# β‐Lactam Ylidenes: An Overlooked Class of *N*‐Heterocyclic Carbenes

**DOI:** 10.1002/chem.202501320

**Published:** 2025-05-08

**Authors:** Leonard Karl, Daniel Deißenbeck, Jan Meisner, Christian Ganter

**Affiliations:** ^1^ Institute for Inorganic Chemistry and Structural Research Heinrich Heine University Düsseldorf Universitätsstraße 1 40225 Düsseldorf Germany; ^2^ Institute for Physical Chemistry Heinrich Heine University Düsseldorf Universitätsstraße 1 40225 Düsseldorf Germany

**Keywords:** β‐lactams, *N*‐heterocyclic carbene, DFT calculations

## Abstract

In this study, the underappreciated class of β‐lactam carbenes (BLCs) is revisited and systematically explored. The required precursors are available in a highly modular synthetic approach from simple starting materials. Two routes may be followed for the generation of the free BLCs: thermal fragmentation of spiro‐fused oxadiazoles (110 °C, toluene), or deprotonation of carbene HCl‐adducts at −80 °C with a strong base. TEP values (2065–2072 cm^−1^), ^77^Se NMR shifts of selenium‐adducts (812–995 ppm), as well as DFT calculations reveal moderate σ‐donor and strong π‐acceptor character of BLCs, leading to a pronounced ambiphilic reactivity. The formation of a wide variety of products under thermal conditions in the cause of oxadiazole fragmentation (110 °C, toluene) is rationalized by a combination of computational reaction discovery and experimental validation. Products include olefinic dimers as well as unprecedented N_2_‐bridged dimers arising from bimolecular reactions, while sterically more demanding precursors are converted in a monomolecular fragmentation reaction via CO release to form ketenimines as intermediates, which finally form amides. With substituents providing appropriate steric protection, a persistent BLC is available via the low temperature route, characterized by its ^13^C NMR shift of 287 ppm at −20 °C.

## Graphical abstract

A highly modular synthetic approach toward four‐membered β‐lactam carbenes (BLCs) is presented. BLCs feature moderate σ‐donor and strong π‐acceptor character, leading to a pronounced ambiphilic reactivity which includes the formation of olefinic dimers as well as unprecedented N_2_‐bridged dimers, oxirane formation with acetone, and fragmentation to ketenimines. Experimental findings are rationalized by DFT calculations.

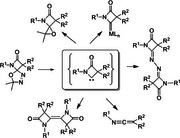

## 1 Introduction

**Scheme 1:**
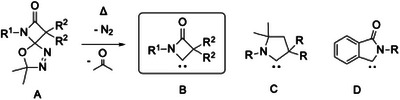
Carbene structures relevant to this contribution.

Due to their ease of synthetic availability via a multitude of routes, NHCs with a large number of different structural motifs have been published in the last three decades^[^ ^1^, ^2^, ^3^, ^4^ ^]^ and have found widespread applications.^[^ ^5^, ^6^, ^7^ ^]^ Remarkably, while a plethora of especially five‐ and six‐membered NHCs have been described, the diversity of four‐membered NHCs reported so far is scarce.^[^ ^8^, ^9^, ^10^, ^11^, ^12^ ^]^ Based on our recent report on the chemistry of carbenes with a urea‐type backbone,^[^ ^13^ ^]^ we wondered which other structural motifs for four‐membered NHCs might be envisioned. Surprisingly, β‐lactam ylidenes have been known as reactive intermediates since 1991^[^ ^14^ ^]^ and their potential for organic synthesis was investigated in some depth. First described by Warkentin in 1991, the precursors to these carbenes are spiro‐fused β‐lactam 1,3,4‐oxadiazoles **A** (Scheme 1).^[^ ^14^ ^]^ These spiro compounds can undergo a 1,3‐dipolar cycloreversion on heating, giving access to β‐lactam ylidenes (BLCs) **B** with the concomitant release of nitrogen and acetone.^[^ ^15^, ^16^, ^17^ ^]^ Although 1,3,4‐oxadiazoles are well‐established since the 1970s as precursors providing in situ access not only to β‐lactam ylidenes but also to other transient carbenes, for example, dialkoxy carbenes,^[^ ^18^, ^19^, ^20^ ^]^ this fragmentation method has obviously never been considered in a systematic fashion as an approach to four‐membered NHCs. This may be due to the fact that the cycloreversion requires high temperatures of up to 110 °C, generally incompatible with the limited thermal stability of the released carbenes or their metal complexes.^[^ ^21^ ^]^

Warkentin explicitly stated that he had never studied the fate of the in situ prepared β‐lactam ylidenes, nor did he observe spectroscopic evidence of their formation. However, he probed their reactivity by cyclopropanations with olefins or H─O activations with alcohols.^[^ ^14^, ^22^ ^]^ In addition, Cheng reported in 2007, trapping reactions with alkyl and aryl isonitriles as well as aryl isocyanates.^[^ ^23^, ^24^, ^25^ ^]^ These reactions remain the only evidence for the in situ formation of carbene species. No electronic or steric characterization has been carried out, nor have any attempts been made to characterize the free carbenes. In this paper, we report on our efforts to stabilize β‐lactam ylidenes by selecting appropriate substituents adjacent to the carbene center and to characterize these novel four‐membered NHCs. This class of stabilized β‐lactam ylidenes nicely complements the well‐established and closely related classes of cyclic (alkyl) (amino) carbenes (CAACs, **C**)^[^ ^26^ ^]^ and cyclic (aryl) (amido) carbenes (CArAmC, **D**).^[^ ^27^ ^]^ We present a highly modular synthetic protocol toward the 1,3,4‐oxadiazole precursors and elucidate their unexpectedly diverse chemistry.

## 2 Results and Discussion

**Scheme 2:**
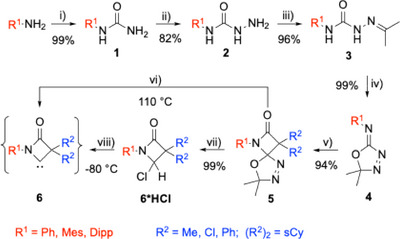
Synthetic pathway toward β‐lactam ylidenes **6**. Reagents and conditions: i) KOCN, HOAc/H_2_O, 90 °C; ii) N_2_H_4_*H_2_O, NaOH, EtOH, reflux; iii) H_3_C(CO)CH_3_, HOAc, EtOH, reflux; iv) Pb(OAc)_4_, DCM, 0 °C to at; v) R^2^_2_HCC(O)Cl, NEt_3_, DCM, 0 °C to at; vi) toluene, 110 °C; vii) HCl/1,4‐dioxane (4 M), toluene, 110 °C; viii) NaHMDS, THF, −80 °C. sCy = spiro‐cyclohexylidene, at = ambient temperature.

The multistep preparation of the desired spiro‐fused β‐lactam oxadiazoles **5** as precursors is based on literature‐known procedures (Scheme 2).^[^ ^28^, ^29^, ^30^ ^]^ However, careful optimization resulted in good to excellent yields in every individual step (see the Supporting Information for details). For example, the yield of the oxidative cyclisation with Pb(OAc)_4_ (step iv) could be significantly improved by an optimized workup procedure.

**Scheme 3:**
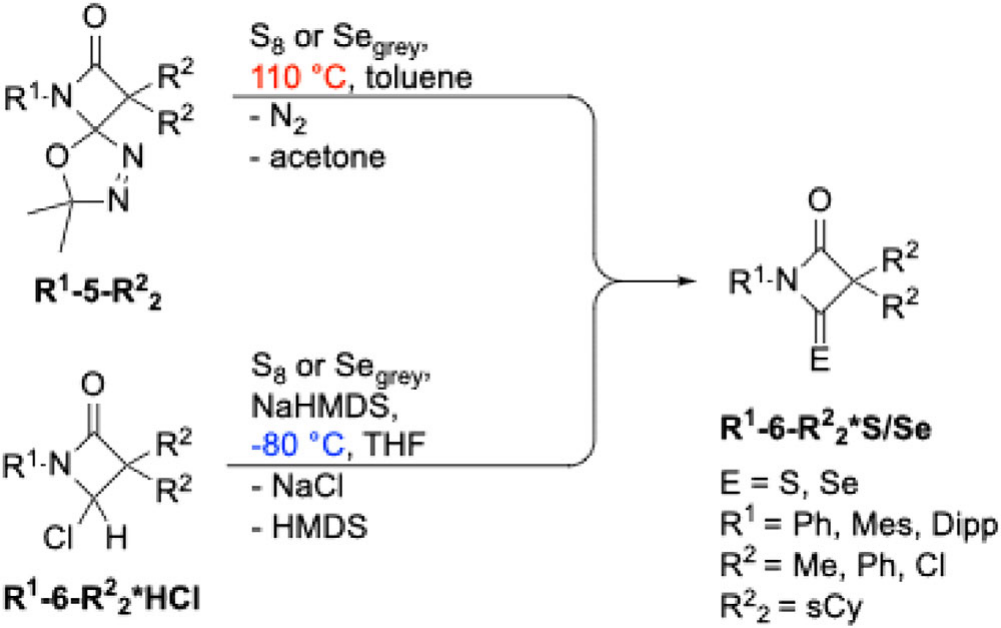
Reactions of carbenes 6 with chalcogenes.

The β‐lactam unit is assembled by a [2+2] cycloaddition of a ketene R^2^_2_C═C═O to the C─N double bond of the oxadiazole (Staudinger synthesis, step v) resulting in the spiro‐fused NHC‐precursors **5**. Substituents R^1^ and R^2^ can be selected broadly and independently of each other, thus warranting a highly modular synthetic approach. While R^1^ is introduced via a primary amine, the groups R^2^ stem from a ketene, which can be obtained from a secondary carboxylic acid chloride upon treatment with triethylamine. Thermal fragmentation of the spiro compounds **5** in refluxing toluene provides the respective carbenes **6** in situ, which are not stable under these conditions but can be trapped with appropriate reagents (vide infra). However, we have developed an alternative route to the sought‐after carbenes by conducting the thermolysis of **5** in the presence of HCl in dioxane, providing the HCl adducts of the carbenes 6*HCl. Subsequent deprotonation of the latter by sodium bis(trimethylsilyl)amide (NaHMDS) at −80 °C opens a “cold” access to the corresponding NHCs **6**, thus obviating any problems due to thermal instability. To demonstrate the applicability of the “cold” and “hot” approaches, we first synthesized thiourea and selenourea derivatives which were obtained in good‐to‐excellent yields following both routes (Scheme 3). Crystal structures of selenoureas Ph‐6‐Ph_2_*Se and Mes‐6‐sCy*Se, and thiourea Mes‐6‐sCy*S proved the successful transformations (Supporting Information).

Having established the straightforward access to carbene precursors **5**, we set out to investigate the thermal decomposition pathway of the oxadiazole precursors in detail. As Warkentin had already investigated the reaction kinetics and the influence of substituents on the reaction rate of the 1,3‐dipolar cycloreversion for the release of carbenes,^[^ ^17^ ^]^ we have not quantified these for our systems. However, we found that for a 1 M **Ph‐5‐Ph_2_** toluene solution thermolysis at 110 °C was complete after four hours, while a 1 M solution of **Ph‐5‐sCy** in toluene required 10 hours for complete conversion. As there are no disadvantages to prolonged heating, thermolysis was routinely carried out for 16 hours to ensure complete conversion.

**Scheme 4:**
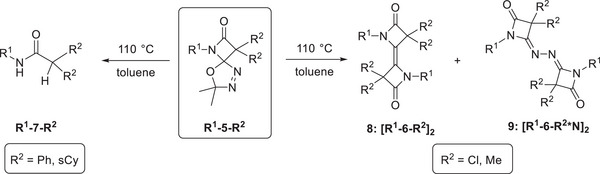
Products observed in precursor thermolysis reactions.

**Figure 1:**
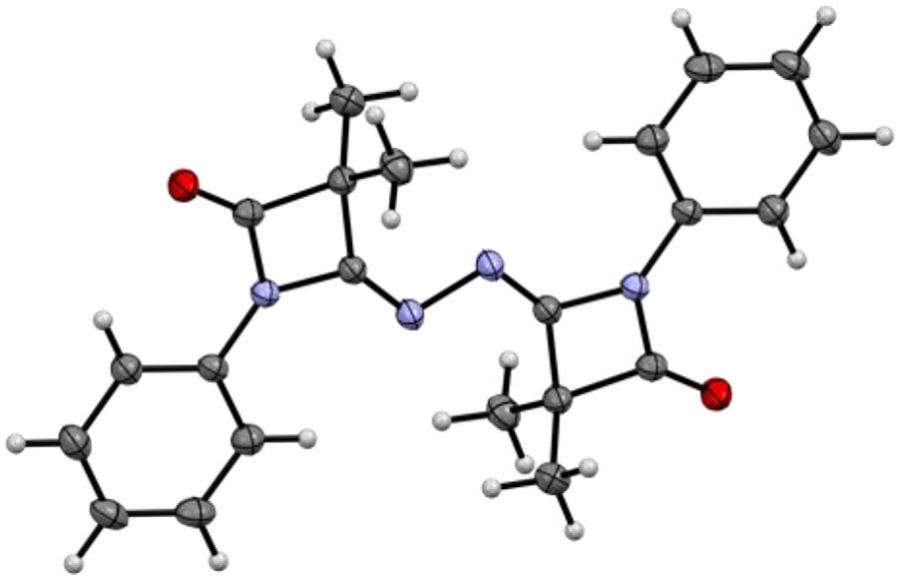
Molecular structure of [Ph‐6‐Me_2_*N]_2_. Thermal ellipsoids are shown with 50% probability.

Structurally quite diverse products were identified in these thermolytic fragmentations, with the particular outcome being governed by the nature of the substituents R^2^, while the *N*‐bonded substituent R^1^ does not play a significant role (Scheme 4). For example, precursors **R^1^‐5‐Cl_2_** and **R^1^‐5‐Me_2_** lead to the formation of dimeric species **8** (olefinic dimer) and **9** (N_2_‐bridged dimer) as main products, the ratio of the two compounds, **8**:**9**, ranging from 91:2 to 38:53. The molecular structures of [Ph‐6‐Me_2_*N]_2_ and **[Ph‐6‐Cl_2_]_2_** were established by single crystal X‐ray diffraction (SCXRD, Figure 1, Supporting Information). Additionally, the amides **7** were obtained as side products in low yield (<5%) for these two precursors. On the other hand, the precursors **R^1^‐5‐sCy** and **R^1^‐5‐Ph_2_** gave the respective amides **7** in >90% yield as major products after chromatographic workup, while the respective N_2_‐bridged species **9** were only present in small amounts. The identity of amides **7** has been established by NMR spectroscopy and SCXRD (Supporting Information).

**Figure 2:**
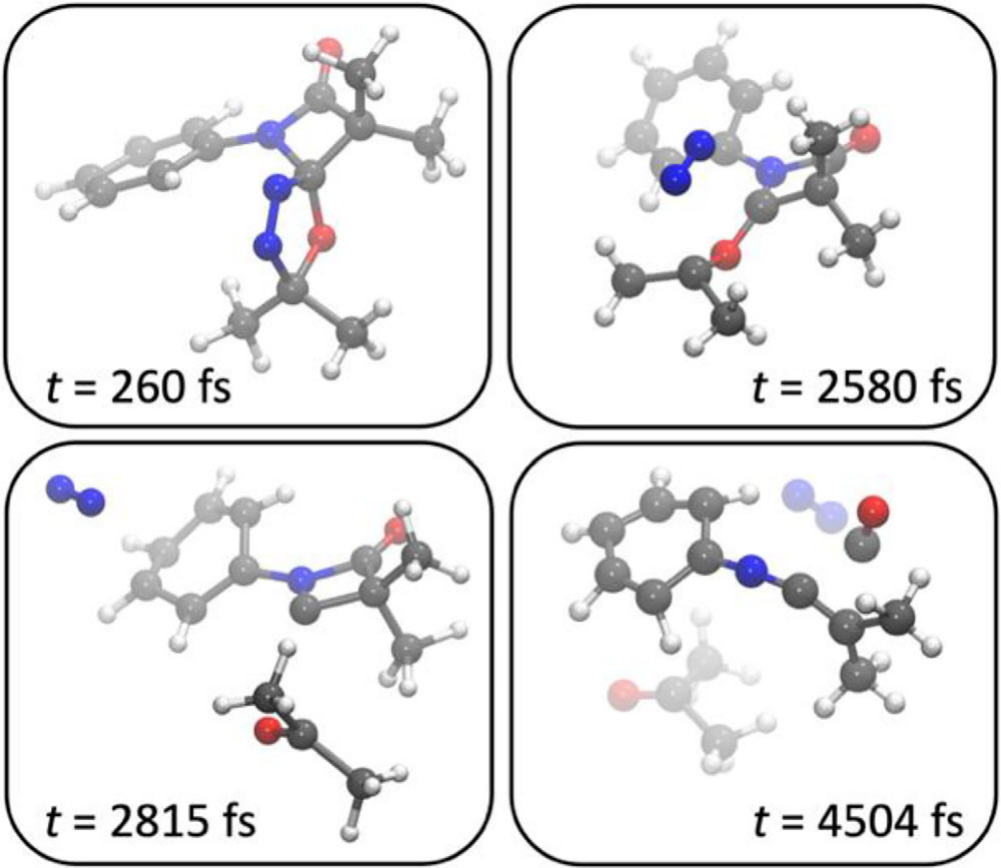
Snapshots of high‐temperature molecular dynamics simulations starting from **Ph‐5‐Me_2_**.

While the formation of olefins as a result of carbene dimerization is well known for ambiphilic NHCs,^[^ ^2^ ^]^ the identification of amides **7** and N_2_‐bridged dimers **9** was surprising at first glance. In order to shed light on the mechanism under which these species are formed, we performed high‐temperature ab initio molecular dynamics (MD) simulations using density functional theory starting from **Ph‐5‐Me_2_** to investigate the formation mechanism as well as the fate of carbene **Ph‐6‐Me_2_**. These high‐temperature MD simulations aim at providing an exploration tool for chemical reactivity, while quantitative results were obtained by subsequent optimization of the discovered elementary reaction. Snapshots of one example trajectory are depicted in Figure 2, further computational details can be found in the Supporting Information.

**Scheme 5:**
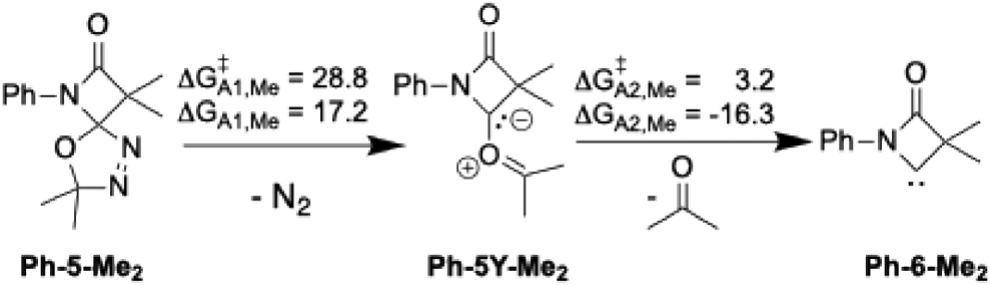
Calculated reaction pathway (kcal/mol) found by MD for the formation of **Ph‐6‐Me_2_**. Free energy barriers and reaction heats are evaluated at 110 °C.

In most MD simulations, N_2_ release takes place as first reaction step, leading to the carbonyl ylide intermediate **Ph‐5Y‐Me_2_** (second snapshot in Figure 2), in line with earlier reports in the literature.^[^ ^31^ ^]^ The intermediate swiftly releases acetone to yield **Ph‐6‐Me_2_** (third snapshot in Figure 2). The N_2_ release step turns out to be rate‐determining with a free energy barrier of 28.8 kcal/mol, which is accessible within the reaction time at the employed temperature of 110 °C. The reaction energetics are shown exemplarily for **Ph‐5‐Me_2_** in Scheme 5 and for other substrates in the Supporting Information. For completeness, we also investigated the alternative reaction sequence, in which acetone is released first, and then N_2_. This alternative mechanism has a significantly higher barrier of 37.1 kcal/mol (see Supporting Information).

**Scheme 6:**
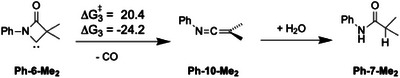
Calculated reaction pathway for the formation of amide **7** via the ketenimine **10** (kcal/mol) at 110 °C.

The computations also provide a convincing explanation for the mechanism under which the in situ‐formed carbenes **6** convert to the related amides **7**: the MD simulations show that extrusion of a CO molecule from **Ph‐6‐Me_2_** forming a ketenimine species **Ph‐10‐Me_2_** (fourth snapshot in Figure 2) can proceed via a free energy activation barrier of 20.4 kcal/mol (Scheme 6). As eventually turned out, the amide **7** forms from the ketenimine **10** by addition of water in the subsequent chromatographic workup, where the water is provided by the silica. Indeed, NMR spectroscopic analysis of the crude reaction solution after thermolysis showed no signals for the amide, but resonances in accord with the presence of the ketenimine, notably ^13^C resonances around 190 ppm. Under rigorous anhydrous conditions a couple of ketene imines could even be isolated. The essential role of water in the amide formation has been further established by running the thermolysis of **Dipp‐5‐sCy_2_** in the presence of D_2_O, where the doubly deuterated analog **Dipp‐7‐sCy_2_(D_2_)** was formed with the two deuterium nuclei being located at the PhND and CDMe_2_ positions, respectively.

**Scheme 7:**
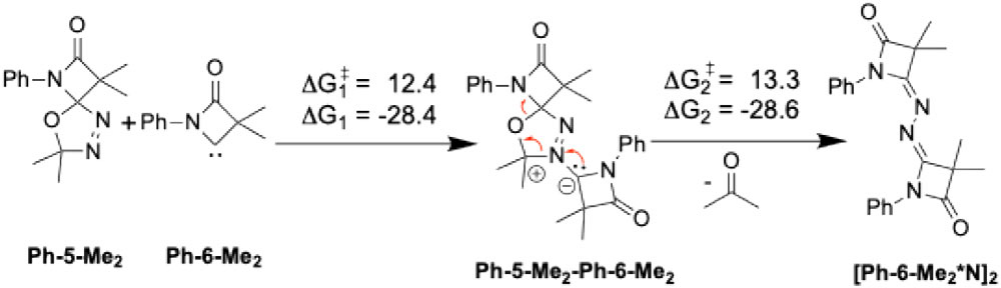
Calculated reaction pathway for the formation of [Ph‐6‐Me_2_*N]_2_. Free energy barriers and reaction heats (kcal/mol) are evaluated at 110 °C.

Once the carbene **Ph‐6‐Me_2_** has formed, a couple of subsequent follow‐up reactions are conceivable other than the abovementioned pyrolysis to ketenimine **Ph‐10‐Me_2_**. For example, according to our calculations, formation of [Ph‐6‐Me_2_*N]_2_ is initiated by the attack of the carbene **Ph‐6‐Me_2_** to a nitrogen atom of the oxadiazole unit of another precursor molecule **Ph‐5‐Me_2_** with a free energy barrier of 12.4 kcal/mol, leading to **Ph‐5‐Me_2_‐Ph‐6‐Me_2_** as an intermediate (Scheme 7). Finally, the release of acetone under concomitant formation of [Ph‐6‐Me_2_*N]_2_ proceeds via a reaction barrier of 13.3 kcal/mol. Considering the overall process from the precursor **Ph‐5‐Me_2_** to the N_2_‐bridged dimer [Ph‐6‐Me_2_*N]_2_, the first step (i.e., carbene formation) appears to have the largest activation barrier. Thus, once **Ph‐6‐Me_2_** has formed, it is prone to react with another precursor molecule under the reaction conditions, ultimately resulting in the formation of [Ph‐6‐Me_2_*N]_2_.

**Scheme 8:**
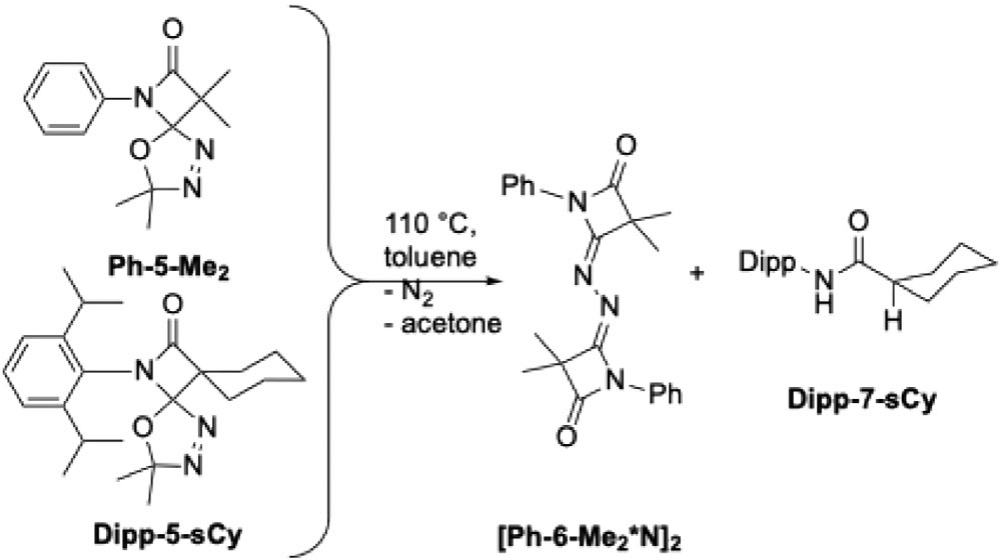
Main products of the thermolysis of precursors with methyl and spiro‐cyclohexylidene substitution.

Alternatively, two molecules of the carbene **Ph‐6‐Me_2_** may combine to form the olefinic carbene dimer **[Ph‐6‐Me_2_]_2_**. Our calculations suggest that this reaction proceeds with a free energy barrier of 7.1 kcal/mol at 110 °C, almost similar to the barrier for the attack of an oxadiazole on the way to the N_2_‐bridged species (12.4 kcal/mol at 110 °C, vide supra). The product ratio between the olefinic dimer **[R^1^‐6‐R^2^_2_]_2_** and the N_2_‐bridged dimers [R^1^‐6‐R^2^_2_*N]_2_ does not depend solely on the relative free energy barriers, since the formation of [R^1^‐6‐R^2^_2_*N]_2_ involves one carbene molecule **6** and one molecule of the oxadiazole precursor **5**, the latter being present in excess in the early stage of the reaction. The formation of **[R^1^‐6‐R^2^_2_]_2_** on the other hand requires the encounter of two carbene molecules **6**, which is in general less likely as carbenes can react further to, for example, amide **7** via the ketenimine intermediate **R^1^‐10‐Me_2_**. Different and varying concentrations of oxadiazole precursor **5** and carbenes **6** can therefore have a strong influence on the product ratio. It should be emphasized that the formation of dimers **[Ph‐6‐R^2^_2_]_2_** and N_2_‐bridged dimers [Ph‐6‐R^2^_2_*N]_2_ as main reaction products under thermal conditions is experimentally observed only for the isostructural precursors with R_2_ = Me and Cl, respectively. As stated above, oxadiazole precursors **5** with R^2^ = Ph or R^2^_2_ = sCy lead preferentially to the formation of the amides **7** as main products (Scheme 6). We attribute this difference in reactivity to the steric demand of the substituents R^2^ at the C3 carbon atom of the β‐lactam moiety, which obviously impedes the bimolecular reaction in the case of Ph and sCy. Thus, refluxing an equimolar mixture of **Ph‐5‐Me_2_** and **Dipp‐5‐sCy** in toluene yielded [Ph‐6‐Me_2_*N]_2_ and **[Ph‐6‐Me_2_]_2_** (roughly in a 5:3 ratio, vide supra) and the amide **Dipp‐7‐sCy** as products and no traces of the cross‐product Ph‐6‐Me_2_*N_2_*Dipp‐6‐sCy were observed (Scheme 8).

The fact that the olefinic dimer is obtained as one product of precursor thermolysis points to a certain degree of ambiphilic character of the intermediate β‐lactam ylidene, as dimerizations are usually not observed for purely nucleophilic NHCs like imidazolylidenes.^[^ ^2^ ^]^ Likewise, the cyclo‐propanations described earlier by Warkentin point into the same direction. It was therefore of interest to quantify the donor and acceptor properties of the involved carbenes. Thus, the selenoureas R^1^‐6‐R^2^_2_*Se (Scheme 3) were characterized by ^77^Se NMR spectroscopy. Chemical shifts in the range of 812–995 ppm (Table 1) reveal the strong π‐accepting character of BLCs, comparable to diamido carbenes (DACs), and ranging between cyclic (aryl) (amido) carbenes (CArAmC) and cyclic (alkyl) (amino) carbenes (CAACs) (Table 2). Notably, the effect of two chlorine atoms as R^2^ (950 ppm) is quite similar to two phenyl substituents (929–995 ppm).

**Table 1:** ^77^Se‐{^1^H}‐NMR chemical shift of selenoureas R^1^‐6‐R^2^_2_*Se in ppm.^[a]^ ^)^

R^1^	R^2^ = Me	R^2^_2_ = sCy	R^2^ = Ph	R^2^ = Cl
Ph	877	899	995	–
Mes	812	843	929	950
Dipp	818	848	936	–

^[a]^ Recorded in acetone‐d_6_ with KSeCN in D_2_O (2 M) as external standard (referenced to 349 ppm) at 298 K.

**Scheme 9:**
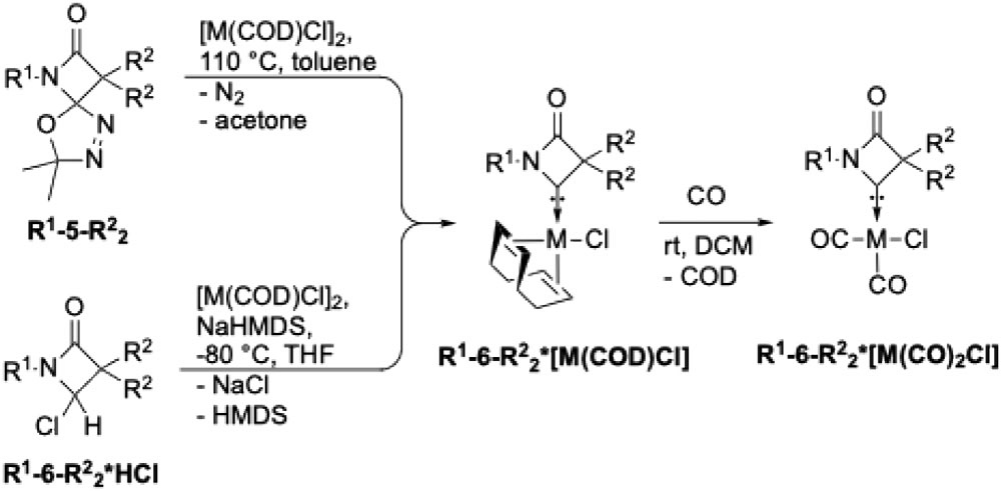
Synthesis of iridium and rhodium COD and carbonyl complexes.

In order to gauge the overall donor capabilities via the TEP‐values, iridium and rhodium CO complexes of carbenes **6** were synthesized (Scheme 9) by way of the M(COD)Cl derivatives, which could be obtained via both synthetic approaches, providing yields above 90% in every case, indicating a high thermal stability of the resulting complexes.

**Figure 3:**
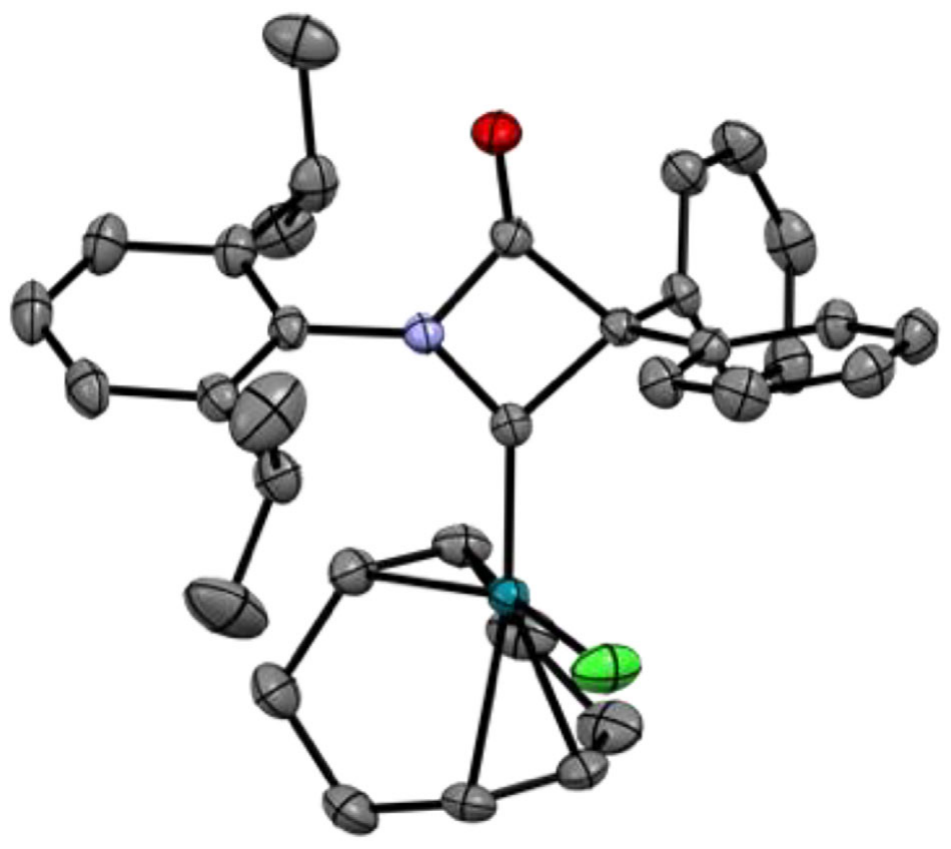
Molecular structure of Dipp‐6‐Ph_2_*[Rh(COD)Cl]. Thermal ellipsoids are shown with 50% probability. Hydrogen atoms are omitted for clarity.

**Figure 4:**
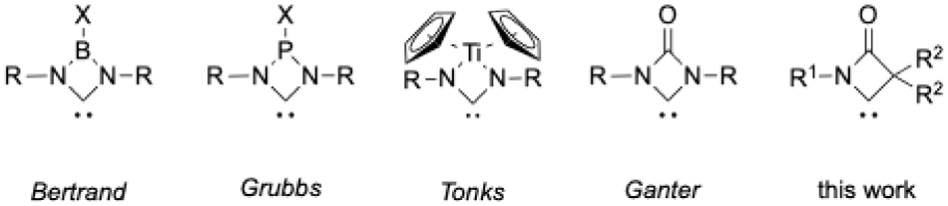
Molecular structures of four‐membered carbenes.

**Figure 5:**
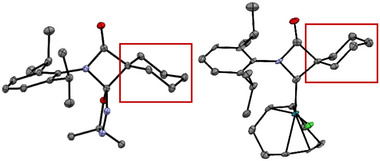
Molecular structures of precursor **Dipp‐5‐sCy** (left, %Vbur = 32.4% and complex Dipp‐6‐sCy*[Rh(COD)Cl] (right, %Vbur = 31.4%). Thermal ellipsoids are shown with 50% probability. Hydrogen atoms are omitted for clarity.

All iridium and rhodium COD complexes were characterized by SCXRD (see Figures 3 and 5 for examples and the Supporting Information for further details), allowing the calculation of the buried volume %Vbur.^[^ ^32^ ^]^ Generally, the complexes feature very short M─C_NHC_ bond lengths^[^ ^33^ ^]^ (well below 200 pm in all cases, Tables 2 and 3), which is even ca. 5 pm shorter than in related complexes with five‐membered diamido carbenes.^[^ ^34^ ^]^ The NCC angle at the carbene center varies between 86° and 94° and is much more acute than in the known four‐membered NHCs reported by Bertrand (94.0(16)°),^[^ ^11^ ^]^ Grubbs (96.7(13)°),^[^ ^9^ ^]^ and Tonks (122.7(9)°) (Figure 4).^[^ ^8^ ^]^ In all complexes and also in all precursors, the nitrogen atom is in a trigonal planar environment (sum of angles 360°), consistent with the planarity of the nitrogen atom in β‐propiolactam.^[^ ^35^ ^]^ Other geometrical parameters like N─C_CO_ or C─O bond lengths are close to values observed for related compounds.^[^ ^13^, ^35^, ^36^ ^]^ Compared to other β‐lactam derivatives, the compounds synthesized in this work appear to be relatively unstrained according to SCXRD,^[^ ^35^ ^]^ which may explain the thermal stability of the β‐lactam moiety upon thermolysis of the precursors **5**. Specifically, the Woodward‐h parameter^[^ ^37^ ^]^ serves as a quantitative measure of strain‐related reactivity in β‐lactams by indicating to which extent the *N* atom is lifted out of the plane defined by the four‐membered lactam ring. In fact, in all compounds characterized by X‐ray diffraction the lactam rings are perfectly planar (Woodward‐h = 0 pm) indicating high stability and low sensitivity to hydrolysis.^[^ ^37^ ^]^ The Woodward‐h also explains why most of the HCl adducts of the NHCs **6** do not hydrolyze and can be handled under standard atmosphere. Only the HCl adducts of spiro‐cyclohexylidene derivatives are susceptible toward hydrolysis, presumably because of the higher strain due to the spiro anellation. Furthermore, in spiro‐cyclohexylidene compounds the cyclohexylidene moiety in precursors **5**, as well as in complexes of **6**, is present in a chair conformation and is always inclined toward the precarbenic carbon atom in the precursor, but bent away in carbene complexes or compounds resulting from carbene trapping reactions (Figure 4). However, the buried volumes %Vbur of the two conformations differ only by one percentage point. A similar, but much more pronounced effect has been described for spiro‐fused CAACs.^[^ ^38^ ^]^

**Table 2:** Properties of selected NHCs.

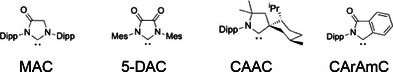				
	MAC	5‐DAC	CAAC	CArAmC
δ ^77^Se (ppm)^[a]^	184^[^ ^33^ ^]^	856^[^ ^48^ ^]^	635^[b]^ ^[^ ^49^ ^]^	1204^[b]^ ^[^ ^27^ ^]^
TEP (cm^−1^)^[c]^	2059^[e]^ ^[^ ^33^ ^]^	2068^[e]^ ^[^ ^34^ ^]^	2042^[d]^ ^[^ ^39^ ^]^	2064^[d]^ ^[^ ^27^ ^]^
%Vbur (%)^[f]^	32.9^[e]^	36.0^[e]^	52.5^[e]^	30.5^[d]^
M–C_NHC_ (ppm)^[g]^	204.4(4)^[e]^ ^[^ ^33^ ^]^	193.1(9)^[e]^ ^[^ ^34^ ^]^	193.9(1)^[e]^ ^[^ ^39^ ^]^	193.7(2)^[d]^ ^[^ ^27^ ^]^
∡NCN/ NCC^[g]^	102.8(3)°^[e]^	105.1(4)°^[d]^	106.5(1)^[h]^	105.3(7)^[d]^

^[a]^ Obtained for the Se‐adducts of the respective carbenes in acetone‐d_6_;

^[b]^ CDCl_3_;

^[c]^ Determined according to literature^[^ ^40^, ^41^, ^42^ ^]^ for complexes of type [M(NHC) (CO)_2_] with M = Ir or Rh. Solvent: DCM;

^[d]^ M = Ir;

^[e]^ M = Rh;

^[f]^ Calculated using SambVca 2 ^[^ ^50^ ^]^ (Bondi radii scaled by 1.17, sphere radius = 3.5 Å, distance of the coordination point from the centre of sphere = 0.0 Å, mesh spacing = 0.10 Å);

^[g]^ Obtained for complexes of type [M(NHC) (COD)Cl] with M = Ir or Rh;

^[h]^ Obtained from crystal structure of free carbene.

**Table 3:** M─C_NHC_ bond length of complexes R^1^‐6‐R^2^_2_*[M(COD)Cl] in pm (first row) and calculated Buried Volumes %Vbur^[a]^ (second row).

R^1^	R^2^ = Me	R^2^_2_ = sCy	R^2^ = Ph
Ph	–	–	190.5(3)^[b]^/190.0(3)^[c]^ 32.9^[b]^/32.7^[c]^
Mes	–	192.5(1)^[b]^ 27.7^[b]^	191.4(1)^[b]^ 32.2^[b]^
Dipp	189.8(3)^[c]^ 29.4^[c]^	190.5(7)^[c]^ 31.4^[c]^	189.3(2)^[c]^ 34.6^[c]^

^[a]^ Calculated using SambVca 2^41^ (Bondi radii scaled by 1.17, sphere radius = 3.5 Å, distance of the coordination point from the center of sphere = 0.0 Å, mesh spacing = 0.10 Å);

^[b]^ M = Ir;

^[c]^ M = Rh.

The buried volumes are generally small, in the range of 28%–35%, and are mainly a consequence of the acute NCC angles of 86°–94°, which causes all the shielding substituents to stand further apart and away from the carbene center. Consequently, in five‐membered CAACs with identical substituents considerably larger buried volumes are found due to the increased NCC angles (Table 2). This structural feature reduces the protecting effect of sterically demanding substituents around the carbene center in the case of the BLCs **6** which might affect the outcome of complexation reactions.^[^ ^39^ ^]^

All COD complexes were converted to the corresponding carbonyl derivatives R^1^‐6‐R^2^_2_*[M(CO)_2_Cl] by treatment with CO in dichloromethane solution (Scheme 9) and their IR spectra were recorded. According to the resulting high TEP values of 2065–2071 cm^−1^, (Table 4)^[^ ^40^, ^41^, ^42^ ^]^ the β‐lactam NHCs **6** are weaker overall donors than CArAmC^[^ ^27^ ^]^ (Table 2) and in the same range as DACs.^[^ ^34^ ^]^ This confirms Grubbs’ observation that the acute angle at the carbene center in four‐membered heterocycles has a diminishing effect on the σ‐donor ability.^[^ ^43^ ^]^ The carbonyl complexes R^1^‐6‐R^2^_2_*[M(CO)_2_Cl] are air‐sensitive and decompose over time, making elemental analysis impossible. This lability is in line with the poor donor and strong acceptor character of the BLCs **6** and has also been observed for carbenes with similar donor/acceptor characteristics.^[^ ^33^, ^42^, ^44^, ^45^, ^46^, ^47^ ^]^

**Table 4:** TEP values of BLCs **R^1^‐6‐R^2^_2_** determined from their [M(CO)_2_Cl] complexes (M = Rh, Ir) in cm^−1^.^[a]^

R^1^	R^2^ = Me	R^2^_2_ = sCy	R^2^ = Ph
Ph	–	–	2071^[b]^/2070^[c]^
Mes	–	2066^[b]^	2071^[b]^
Dipp	2068^[c]^	2065^[c]^	2069^[c]^

^[a]^ Calculated according to literature.^[^ ^40^, ^41^, ^42^ ^]^ DCM as solvent;

^[b]^ M = Ir;

^[c]^ M = Rh.

**Scheme 10:**
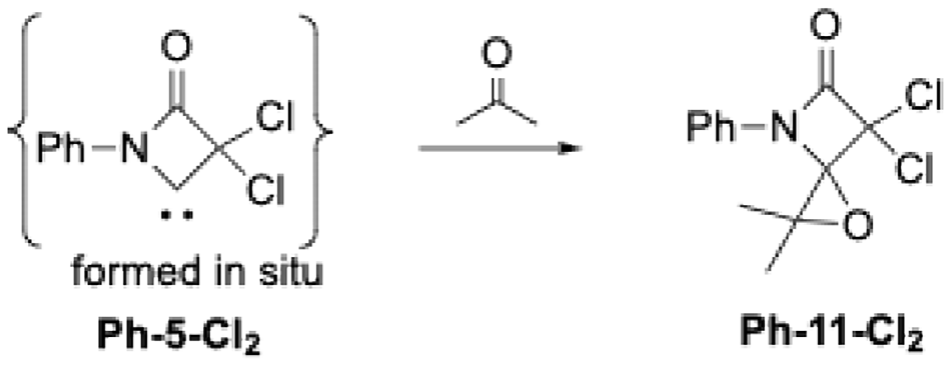
Formation of epoxides 11 with Ph‐5‐Cl_2_ as an example.

DFT calculations were carried out to rationalize the experimentally derived ligand properties of the BLCs. The energies of the filled σ‐type and the unfilled π*‐type orbitals located on the carbene C atom were computed together with the singlet‐triplet energy gaps for representative BLCs and structurally related NHCs (Table 5). The σ‐donor ability and π‐acceptor strength of the **Dipp‐6‐sCy** is the highest among the BLCs investigated (lowest π*, highest σ). Their calculated singlet‐triplet gaps of 1.70–1.80 eV are similar to the energy gaps obtained for monoamido carbenes (MACs). Additionally, the calculated σ/π*‐energy difference of 4.4–4.8 eV is between the values calculated for MACs and CArAmCs. Clearly, from these data, a pronounced degree of ambiphilic character is to be expected for the BLCs. Indeed, formation of olefinic dimers is in accord with this expectation, as are the cyclopropanations reported by Warkentin.^[^ ^14^ ^]^ Additional evidence for the ambiphilicity is provided by the reaction of the BLCs with acetone which leads to the formation of epoxides via a formal [2+1]‐cycloaddition (Scheme 10).

**Table 5:** Comparison of characteristic data for selected carbenes. All energies are given in eV. R = Dipp.^[a]^

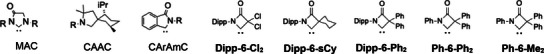								
	MAC	CAAC	CArAmC	Dipp‐6‐Cl_2_	Dipp‐6‐sCy	Dipp‐6‐Ph_2_	Ph‐6‐Ph_2_	Ph‐6‐Me_2_
E(π*‐MO)^[a]^	−2.00	−0.99	−0.33	−2.25	−1.61	−1.77	−1.97	−1.86
E(σ‐MO)^[a]^	−6.06	−6.19	−5.14	−7.07	−6.21	−6.37	−6.41	−6.38
E(π*)‐E(σ)	4.06	5.20	4.81	4.82	4.59	4.60	4.43	4.52
ΔE(S‐T)	1.89	2.79	2.05	1.80	1.78	1.78	1.70	1.70

^[a]^ E(π*‐MO) and E(σ‐MO) are the energies of the unfilled π*‐type and the filled σ‐type orbitals located on the carbene atom, respectively.

**Figure 6:**
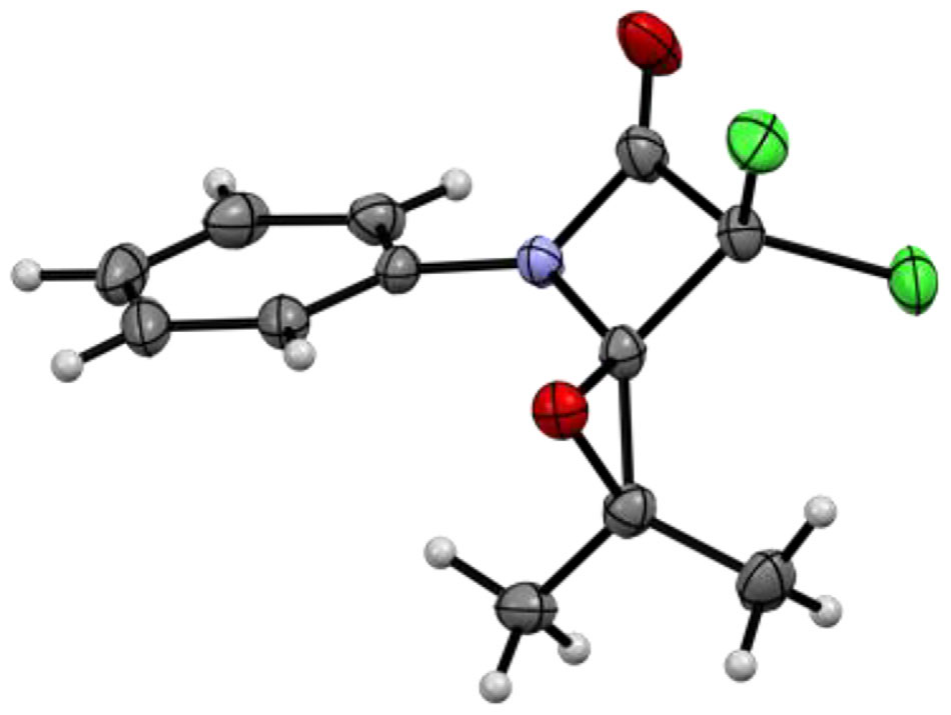
Molecular structure of epoxide Ph‐11‐Cl_2_. Thermal ellipsoids are shown with 50% probability.

Epoxides **11** are formed as side products both under thermal conditions (9%–13% yield) as well as along the cold route (15–21% yield) if thermolysis of precursors **5** or deprotonation of HCl‐adducts 6*HCl are carried out in the presence of acetone, respectively. To the best of our knowledge, the formation of epoxides involving NHCs has been described only by Bielawski^[^ ^51^ ^]^ for the reaction of DACs with aldehydes, while the transformation involving ketones appears to be without precedence. The reaction profile was evaluated by DFT calculations for the BLC **Ph‐5‐Cl_2_** as a representative example and is best described as a concerted process with a free energy activation barrier of 19.6 kcal/mol at 110 °C, 16.1 kcal/mol at 25 °C, and 11.8 kcal/mol at −80 °C. The molecular structure of **Ph‐11‐Cl_2_** has been determined by SCXRD (Figure 6).

**Scheme 11:**
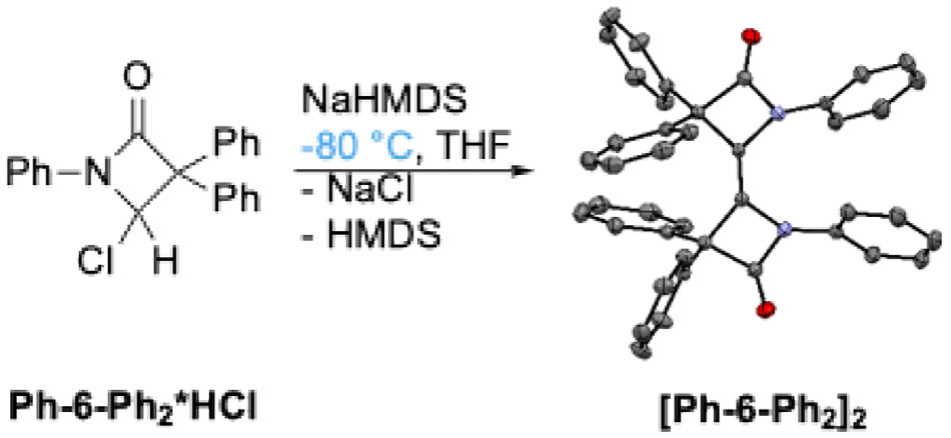
Formation of olefinic carbene dimer **[Ph‐6‐Ph_2_]_2_**. Thermal ellipsoids are shown with 50% probability. Hydrogen atoms are omitted for clarity.

As the reactions carried out under thermal conditions clearly do not provide access to the free carbenes, the focus was shifted to the cold approach, that is, deprotonation of the HCl adducts 6*HCl at low temperature. However, upon treatment of Ph‐6‐Ph_2_*HCl with NaHMDS at −80 °C, no low‐field signal in the ^13^C NMR spectrum could be observed that would hint to the formation of the free carbene. Instead, the dimer **[Ph‐6‐Ph_2_]_2_** was obtained in good yield after workup (Scheme 11). This is a noteworthy result, because under thermal conditions, the precursor **Ph‐5‐Ph_2_** did not yield a dimer as the main product but instead led to the formation of the amide **Ph‐7‐Ph_2_** (vide supra). At −80 °C, the free energy barrier of 21.1 kcal/mol of the carbene decomposition cannot be overcome and therefore the amide formation is suppressed.

The structure of the dimer was determined by SCXRD. Interestingly, although sterically hindered, all four phenyl substituents of the two CPh_2_ units are on the same side of the dimer's double bond. Weak T‐shaped C‐H‐π bonding interactions (*d* = 257(8) pm) between the two pairs of CPh_2_ phenyl groups are observed in the solid state. The overall conformation of the dimer features the two planar β‐lactam rings forming an interplanar angle of 2.2(9)° and a small but significant deviation of the *N* atoms from a trigonal‐planar arrangement (angle sum 353.9(3)°).

**Scheme 12:**
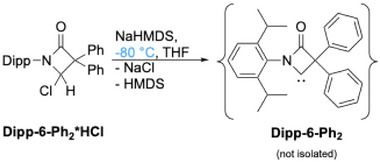
Formation of persistent BLC **Dipp‐6‐Ph_2_** at low temperature.

In order to prevent the dimerization of the carbene formed in situ, we resorted to the precursor of the BLC with the highest buried volume, Dipp‐6‐Ph_2_*HCl. Gratifyingly, the persistent BLC **Dipp‐6‐Ph_2_** was successfully synthesized at −80 °C by deprotonation of the HCl adduct with NaHMDS (Scheme 12). The carbene is neither temperature nor air stable and can only be handled in an inert atmosphere at temperatures below −20 °C, which prohibited elemental analysis and mass spectrometric characterization.

Thus, the carbene was characterized exclusively by ^1^H‐ and ^13^C‐{^1^H}‐NMR spectroscopy, where a chemical shift of 287.2 ppm in the ^13^C spectrum is close to the values of ca. 280 ppm observed for DACs.^[^ ^52^ ^]^ Obviously, sterically demanding substituents on the nitrogen atom are required to achieve a notable degree of stabilization. The isopropyl groups of the Dipp substituent are particularly useful in this regard because they protrude above and below the plane of the carbene carbon atom. Experiments with sterically even more demanding *N*‐substituents are currently underway.

## 3 Conclusion

The class of β‐lactam ylidenes (BLCs) has been investigated in a systematic approach. The required precursors are available according to a highly modular synthetic approach from simple starting materials. Two routes may be followed for the generation of the free BLCs: thermal fragmentation from spiro‐fused oxadiazoles, or deprotonation of carbene HCl‐adducts at low temperature. TEP values, ^77^Se NMR shifts, as well as DFT calculations reveal moderate σ‐donor and strong π‐acceptor character, leading to a pronounced ambiphilic reactivity. The formation of diverse products under thermal conditions in the cause of oxadiazole fragmentation could be rationalized by DFT calculations. Thus, olefinic dimers and N_2_‐bridged dimers arise from bimolecular reactions, while sterically more demanding precursors are converted in a monomolecular fragmentation via CO release to intermediate ketenimines which give rise to the formation of amides upon chromatographic workup. Due to their versatile reactivity and straightforward availability, BLCs are ideally suited as a platform for further chemical studies.

## Supporting Information

The authors have cited additional references within the Supporting Information.^[^ ^40^, ^41^, ^42^, ^53^, ^54^, ^55^, ^56^, ^57^, ^58^, ^59^, ^60^, ^61^, ^62^, ^63^, ^64^, ^65^, ^66^, ^67^, ^68^, ^69^, ^70^, ^71^ ^]^

## Conflict of Interests

The authors declare no conflict of interest.

## Supporting information



Supporting Information

## Data Availability

The data that support the findings of this study are available in the supporting information of this article.
