# Supplementary material for: β‐Lactam Ylidenes: An Overlooked Class of N‐Heterocyclic Carbenes
Source: Chemistry. 2025 May 8;31(31):e202501320. doi: 10.1002/chem.202501320 (PMC12133631; doi:10.1002/chem.202501320)
Supplement: Supplementary file 1 — Supporting Information [file CHEM-31-e202501320-s001.pdf]

Supporting Information

β-Lactam Ylidenes: an Overlooked Class of *N*-heterocyclic Carbenes

Leonard Karl[a], Daniel Deißenbeck[b], Jan Meisner[b], Christian Ganter[a*]*

[a] Institute of Inorganic Chemistry and Structural Research,
Heinrich Heine University Düsseldorf, Universitätsstraße 1, 40225 Düsseldorf, Germany

[b] Institute of Physical Chemistry,
Heinrich Heine University Düsseldorf, Universitätsstraße 1, 40225 Düsseldorf, Germany

Table of Contents

I.	General Considerations	S2
II.	Synthetic Procedures and NMR spectra	S3
a.	Syntheses of ureas **1**	S3
b.	Syntheses of semicarbazides **2**	S8
c.	Syntheses of semicarbazones **3**	S13
d.	Syntheses of 2-imino-Δ^3-1,3,4-oxadiazoles **4**	S18
e.	Syntheses of precursors **5**	S23
f.	Thermolysis of precursors **5**	S41
g.	Syntheses of precursors **6*HCl**	S70
h.	Syntheses of selenium adducts **6*Se**	S82
i.	Syntheses of sulfur adducts **6*S**	S102
j.	Syntheses of iridium/rhodium 1,5-COD-complexes **6*[Ir/Rh(COD)Cl]**	S112
k.	Syntheses of iridium/rhodium carbonyl-complexes **6*[Ir/Rh(CO)$_2$Cl]**	S123
l.	Syntheses of epoxides **11**	S127
m.	Reactions of precursors **6*HCl** with NaHMDS	S131
III.	Crystallographic Data	S134
IV.	Computational Details	S177
V.	References	S312

I. General Considerations

All manipulations, with the exception of the syntheses of compound classes **1**, **2** and **3**, were carried out in a dry, oxygen-free nitrogen atmosphere using standard Schlenk techniques. Glassware was dried at 200 °C for 16 h prior to use. Toluene, *n*-hexane and Tetrahydrofuran (THF) were dried over sodium and distilled or distilled from sodium-benzophenone ketyl under nitrogen prior to use, respectively. Dichloromethane (DCM) was dried over calcium hydride and distilled under nitrogen prior to use. Acetone was dried over Drierit® and distilled. Deuterated solvents were dried over aluminum oxide super I, filtered and stored over 3 Å molecular sieve under dry, oxygen-free nitrogen. Silica, *Celite 535*® and aluminum oxide 90 active neutral (activity stage I) were heated at 200 °C under $1{\cdot}10^{-3}$ mbar for 16 h and stored under dry, oxygen-free nitrogen. All air-sensitive compounds were handled in a *MBraun labmaster 130* glovebox under dry, oxygen-free argon (H_2O/O_2 < 0.01 ppm). All commercially available chemicals were used without further purification. Thermolysis experiments, unless otherwise stated, were carried out in a sealed tube.

NMR spectra were recorded with a *Bruker Avance III – 300* (^{1}H 300 MHz, ^{13}C{^{1}H} 75 MHz), *Bruker Avance III – 600* (^{77}Se{^{1}H} 114 MHz) or *Bruker Avance NEO EVO – 600* (^{1}H 600 MHz, ^{13}C{^{1}H} 150 MHz) spectrometer at 298.0 K, unless otherwise stated. NMR spectra of air and moisture sensitive compounds were recorded using *J. Young* valve NMR tubes. Chemical shifts are reported in delta (δ) expressed in parts per million ppm (ppm) downfield from tetramethyl silane in ^{1}H and ^{13}C{^{1}H} spectra using the residual peak of deuterated solvents as internal standard (^{1}H: $CDCl_2$ 5.32 ppm, $CDCl_3$ 7.26 ppm, C_6D_6 7.16 ppm, DMSO-d_6 2.50 ppm, THF-d_8 3.58; ^{13}C{^{1}H}: $CDCl_2$ 53.8 ppm, $CDCl_3$ 77.2 ppm, C_6D_6 128.1 ppm, DMSO-d_6 39.5, THF-d_8 67.6 ppm). ^{77}Se{^{1}H} spectra were recorded in acetone-d_6 and referenced to external KSeCN in D_2O (0.25 mol/l) with a chemical shift of -329 ppm. For dimers, the monomeric unit is described by the integrals in ^{1}H NMR for clarity. Mass spectra were recorded with a *JEOL JMS-Q1600GC single-quad*-spectrometer (EI) and a *Bruker Daltonics UHR-QTOF maXIS 4G* spectrometer (HR-ESI) equipped with a *Dionex Ultimate 3000 RS* UHPLC-system (LC-MS). Elemental analyses were performed using a *Elementar Vario MICRO cube*. Infrared spectra of the dicarbonyl complexes of the general form NHC-M-$(CO)_2$Cl (M = Ir, Rh) were recorded using a *Shimadzu IRAffinity-1* FTIR spectrometer with IR-transparent liquid cells made of sodium chloride glass with a total volume of

100 µl. The TEP values of the dicarbonyl complexes of the general form NHC-M-$(CO)_2Cl$ (M = Ir, Rh) were calculated according to the literature.[1-3]

Crystals suitable for X-ray analysis were selected by means of a polarization microscope and fixed with NVH oil on a loop glass fiber. Crystal structures were determined at temperatures between 100.0 K and 250.0 K using a *Rigaku XtaLAB-Synergy S* diffractometer equipped with a *HyPix 6000* detector and a *Photon Jet* X-ray source with monochromated Cu-K_{α} or Mo-K_{α} (λ = 0.71073 Å) radiation with generator setting of 50 kV and 1 mA. The data collection was determined and raw data was processed using *CrysAlis^{Pro} v43.98a* (*Rigaku Corporation*).

Crystal structures were solved by direct methods and subsequent ΔF syntheses using *SHELXT*[4] and *OLEX2*.[5] Approximate positions of the H atoms were found in different stages of converging refinements by full-matrix least-squares calculations on F^2 using *SHELXL*[6] and *OLEX2*.[5] Anisotropic displacement parameters were refined for all atoms heavier than hydrogens. The riding model was applied for corresponding H atoms assuming idealized bond lengths and angles for all the CH, CH_2 and CH_3 groups. The isotropic displacement parameters of these H atoms were constrained to 120, 120 and 150% of the equivalent isotropic displacement parameters of the parent carbon atoms and H atoms of CH_3 groups were allowed to rotate around the neighboring C—C bonds. All graphics were created employing *Mercury v.2021.2.2* (*CCDC*). Deposition numbers under section "III. Crystallographic Data" contain the supplementary crystallographic data for this paper (numbers 2423702–2423744). These data are provided free of charge by the joint CCDC and FIZ Karlsruhe Access Service.

II. Synthetic procedures and NMR spectra

a. Syntheses of ureas 1

The corresponding primary amine (0.5 mol, 1 eq.) was dissolved at 90 °C in a mixture of 400 ml acetic acid and 100 ml water. Potassium cyanate (122 g, 1.5 mol, 3 eq.) was suspended in 400 ml of 60 °C hot water and slowly added at 90 °C. The urea precipitated immediately, was filtered off and recrystallized from ethanol to give the desired product as colorless, needle-shaped, air-stable crystals after drying in vacuo.

1-Ph: Amine used: aniline (46 ml, 0.5 mol). Colorless, needle-shaped, air-stable crystals. Yield: 67 g, 99%. ^{1}H NMR (300 MHz, DMSO-d$_6$, 298.0 K): δ = 8.54 (s, 1H; NH), 7.45–7.38 (m, 2H; CH$_{\text{aryl-ortho}}$), 7.27–7.17 (m, 2H; CH$_{\text{aryl-meta}}$), 6.93–6.85 (m, 1H; CH$_{\text{aryl-para}}$), 5.89 (s, 2 H; NH$_2$) ppm. ^{13}C{^{1}H} NMR (75 MHz, DMSO-d$_6$, 298.0 K): δ = 156.2 (s; C=O), 140.6 (s; NC$_{\textit{aryl}}$), 128.7 (s; C$_{\text{aryl-meta}}$), 121.2 (s; C$_{\text{aryl-para}}$), 117.9 (s; C$_{\text{aryl-ortho}}$) ppm. MS (EI, 70 eV, 80 °C) *m/z* (%): 136 (33) [M]$^{+\cdot}$, 141 (49), 85 (100), 58 (67). Elemental analysis calcd for $C_7H_8N_2O$: C 61.75, H 5.92, N 20.58, found: C 61.49, H 6.12, N 20.74.

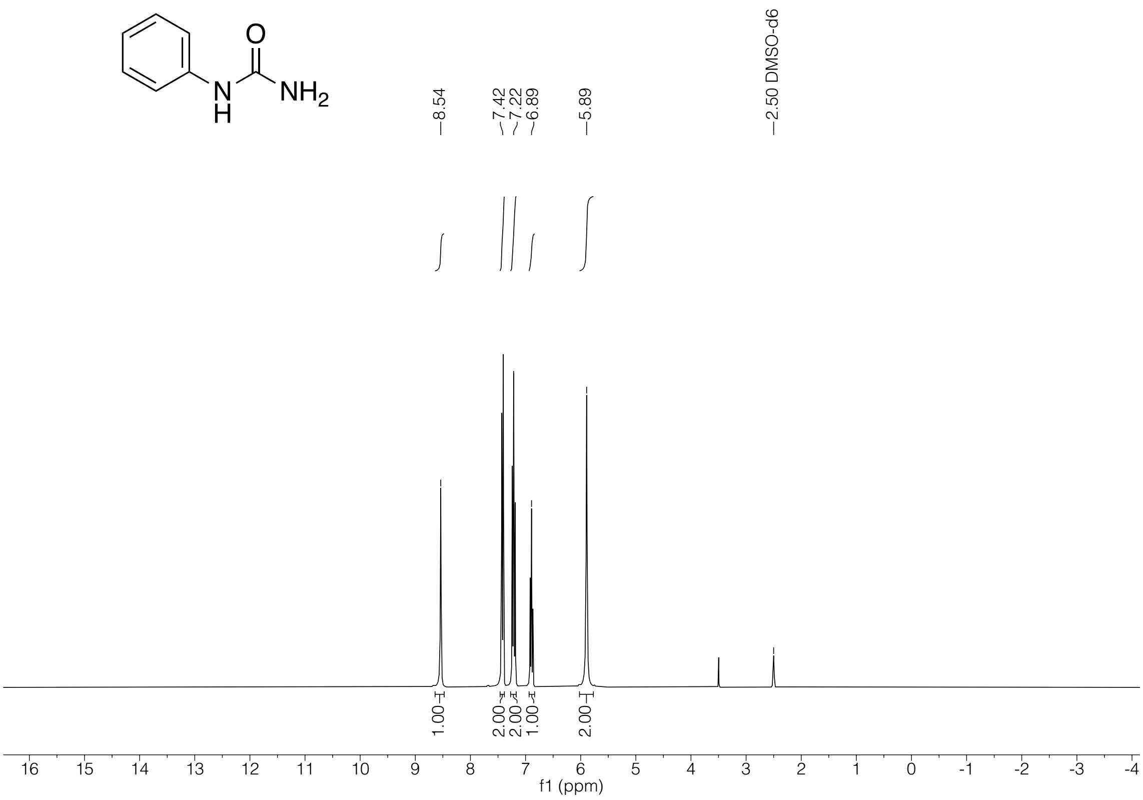

Figure SF1. ^{1}H NMR (300 MHz, DMSO-d$_6$, 298.0 K) spectrum of **1-Ph**.

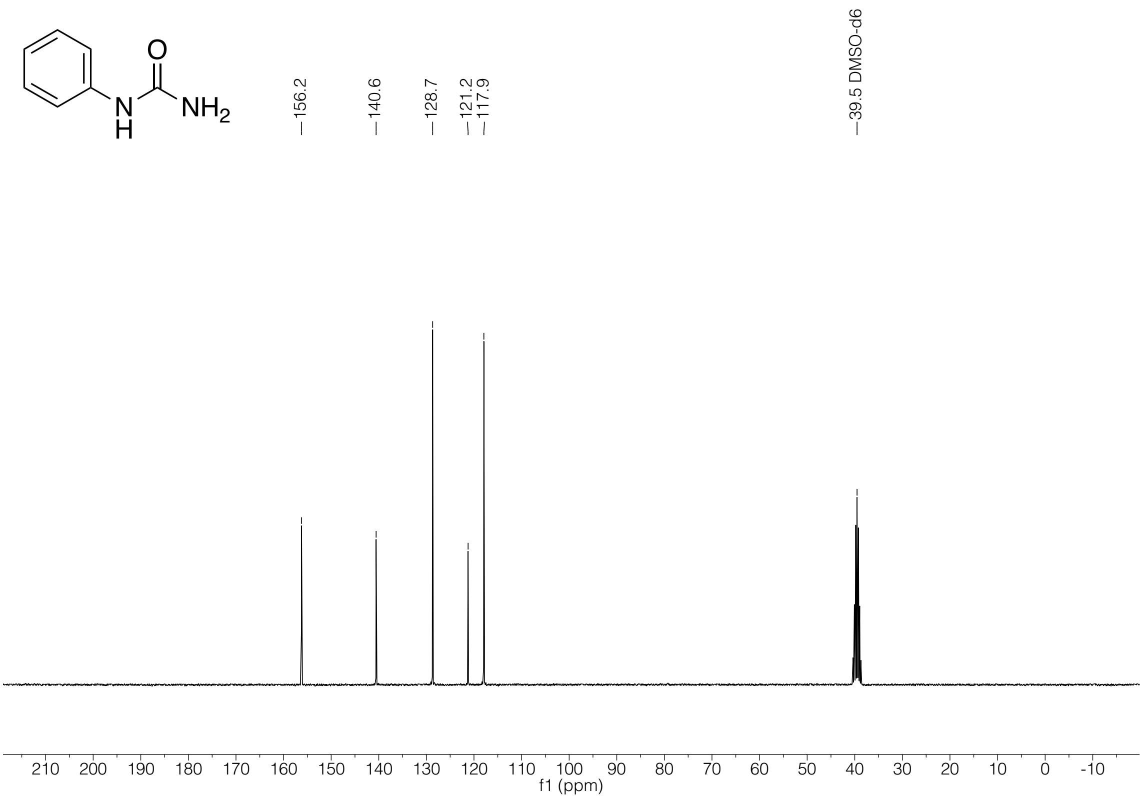

Figure SF2. $^{13}C\{^1H\}$ NMR (75 MHz, DMSO-d_6, 298.0 K) spectrum of **1-Ph**.

1-Mes: Amine used: 2,4,6-trimethylaniline (70 ml, 0.5 mol). Colorless, needle-shaped, air-stable crystals. Yield: 88 g, 99%. ^1H NMR (300 MHz, DMSO-d_6, 298.0 K): δ = 7.39 (s, 1H; NH), 6.83 (appears as s due to broadening, 2H; CH_{meta}), 5.61 (s, 2 H; NH_2), 2.20 (s, 3H; $CH_{3\text{-}para}$), 2.12 (s, 6H; $CH_{3\text{-}ortho}$) ppm. $^{13}C\{^1H\}$ NMR (75 MHz, DMSO-d_6, 298.0 K): δ = 156.8 (s; C=O), 135.3 (s; NC_{aryl}), 134.5 (s; $C_{aryl\text{-}meta}$), 133.5 (s; $C_{aryl\text{-}para}$), 128.2 (s; $C_{aryl\text{-}ortho}$), 20.5 (s; $CH_{3\text{-}para}$), 18.1 (s; $CH_{3\text{-}ortho}$) ppm. MS (EI, 70 eV, 100 °C) *m/z* (%): 178 (63) $[M]^{+\cdot}$, 161 (19), 135 (83), 120 (100), 91 (20). Elemental analysis calcd for $C_{10}H_{14}N_2O$: C 67.39, H 7.92, N 15.72, found: C 67.29, H 8.01, N 15.64.

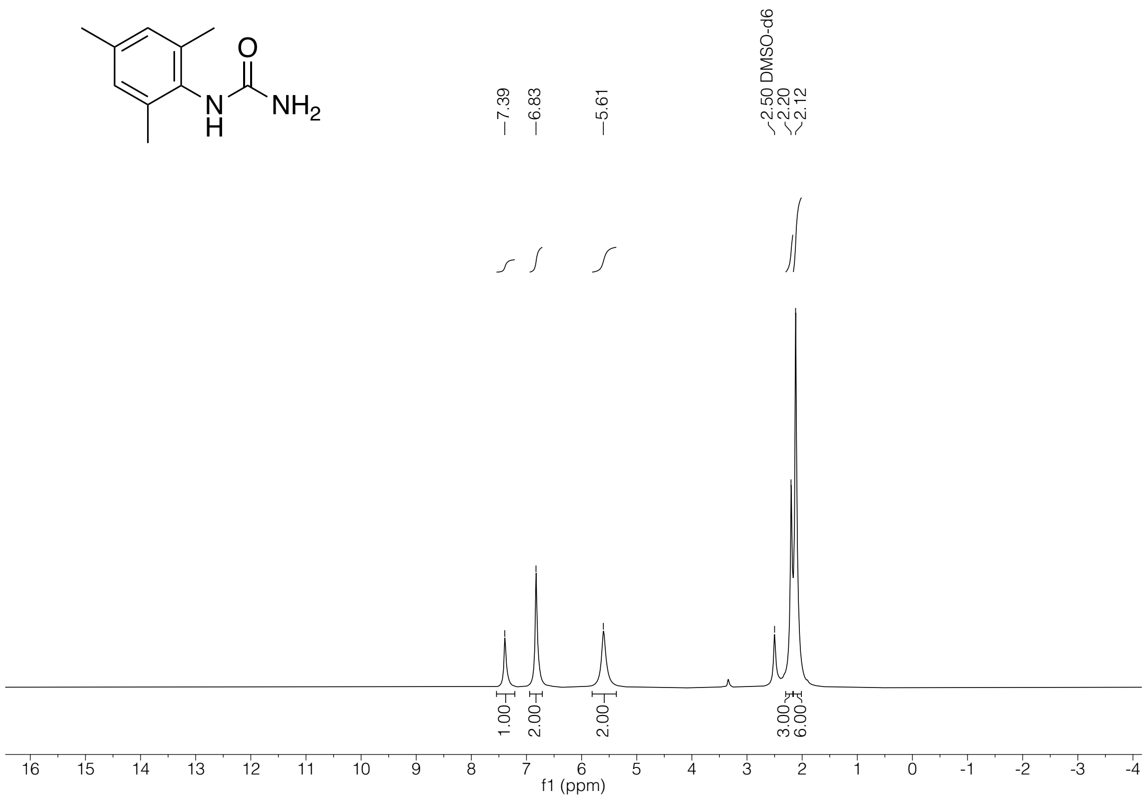

Figure SF3. ^{1}H NMR (300 MHz, DMSO-d_6, 298.0 K) spectrum of **1-Mes**.

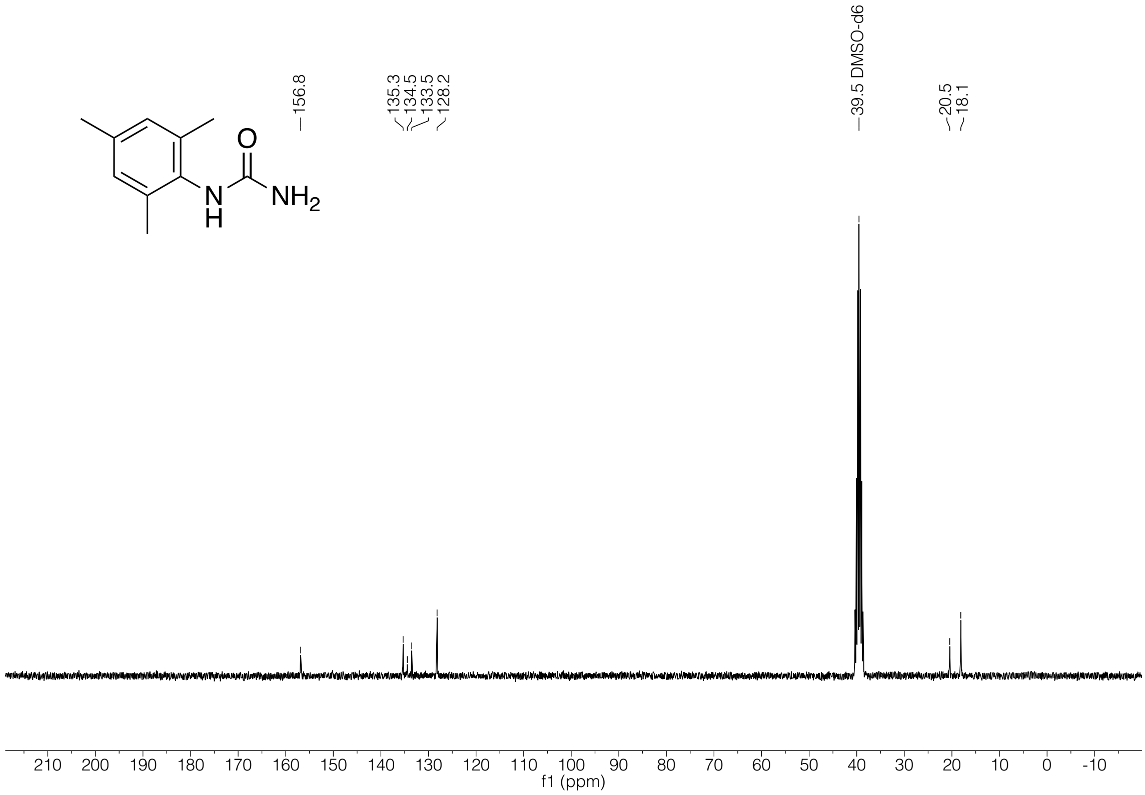

Figure SF4. $^{13}C\{^{1}H\}$ NMR (75 MHz, DMSO-d_6, 298.0 K) spectrum of **1-Mes**.

1-Dipp: Amine used: 2,6-diisopropylaniline (94 ml, 0.5 mol). Colorless, needle-shaped, air-stable crystals. Yield: 109 g, 99%. ^{1}H NMR (300 MHz, DMSO-d$_6$, 298.0 K): δ = 7.46 (s, 1H; NH), 7.23–7.17 (m, 1H; $CH_{aryl\text{-}para}$), 7.13–7.07 (m, 2H; $CH_{aryl\text{-}meta}$), 5.69 (s, 2 H; NH_2), 3.17 (sept, J = 7 Hz, 2H; CH_{iPr}), 1.13 (d, J = 7 Hz, 12H; $CH_{3\text{-}iPr}$) ppm. ^{13}C{^{1}H} NMR (75 MHz, DMSO-d$_6$, 298.0 K): δ = 157.8 (s; C=O), 146.7 (s; $C_{aryl\text{-}ortho}$), 133.3 (s; NC_{aryl}), 126.8 (s; $C_{aryl\text{-}para}$), 122.7 (s; $C_{aryl\text{-}meta}$), 27.8 (s; CH_{iPr}), 23.4 (s; $CH_{3\text{-}iPr}$) ppm. MS (EI, 70 eV, 80 °C) *m/z* (%): 220 (18) $[M]^{+\cdot}$, 203 (100), 188 (98), 176 (66), 160 (34), 134 (28), 120 (22), 106 (16), 91 (12). Elemental analysis calcd for $C_{13}H_{20}N_2O$: C 70.87, H 9.15, N 12.72, found: C 70.72, H 9.07, N 12.70.

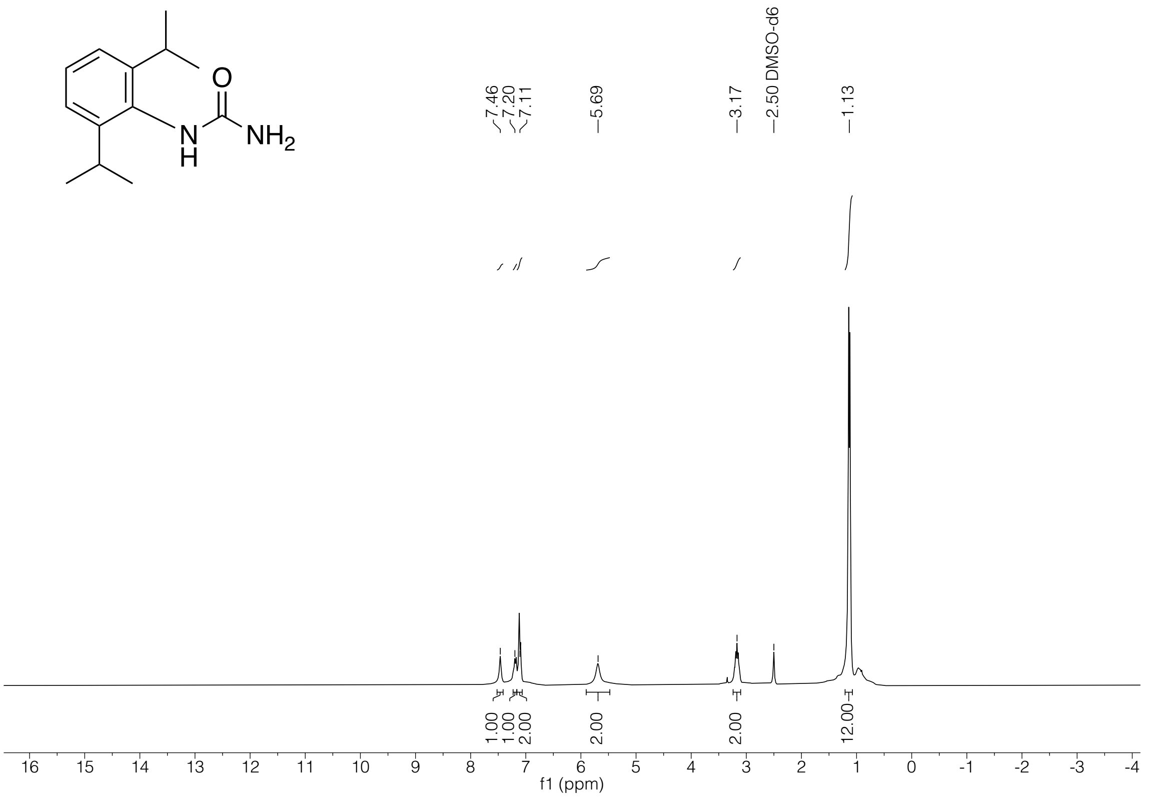

Figure SF5. ^{1}H NMR (300 MHz, DMSO-d$_6$, 298.0 K) spectrum of **1-Dipp**.

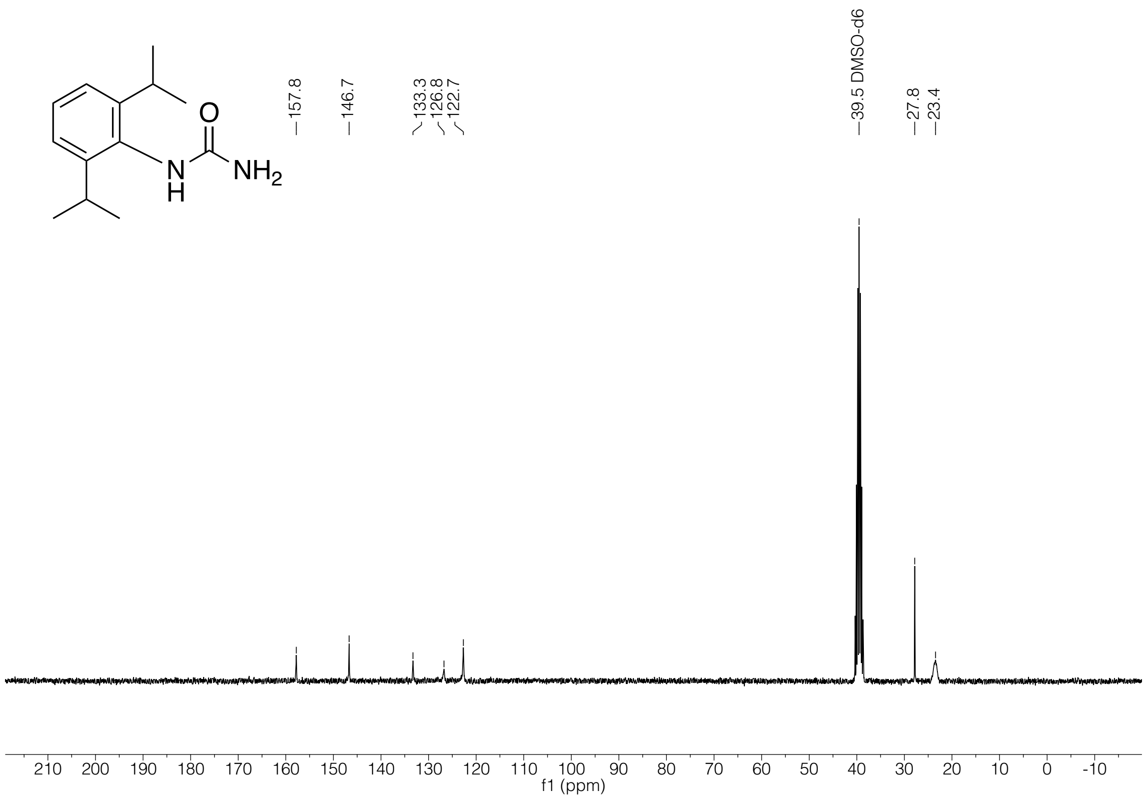

Figure SF6. $^{13}C\{^{1}H\}$ NMR (75 MHz, DMSO-d_6, 298.0 K) spectrum of **1-Dipp**.

b. Syntheses of semicarbazides 2

The corresponding urea **1** (0.45 mol, 1 eq.), hydrazine monohydrate (90 ml, 1.8 mol, 4 eq.) and sodium hydroxide (7.2 g, 0.18 mol, 0.4 eq.) were suspended in 250 ml ethanol and refluxed for 48 h. After cooling at 5 °C the desired product crystallized as colorless, needle-shaped, air-stable crystals. The crystals were filtered off, washed with ice-cold ethanol and hexane and dried in vacuo.

An alternative synthesis can be carried out in a microwave: The corresponding urea **1** (0.05 mol, 1 eq.), hydrazine monohydrate (9 ml, 0.18 mol, 4 eq.) and sodium hydroxide (0.72 g, 0.02 mol, 0.4 eq.) were suspended in 25 ml ethanol and reacted at a maximum of 300 W at 120 °C for 8 h. The product precipitates again as needle shaped, air-stable crystals upon cooling. The yield does not differ from conventional heating.

2-Ph: Urea used: **1-Ph** (61 g, 0.45 mol). Colorless, needle-shaped, air-stable crystals. Yield: 56 g, 82%. ^{1}H NMR (300 MHz, DMSO-d$_6$, 298.0 K): δ = 8.60 (s, 1H; C$_{aryl}$NH), 7.56–7.48 (m, 2H; CH$_{aryl-ortho}$), 7.38 (s, 1H; H$_2$NN*H*), 7.27–7.18 (m, 2H; CH$_{aryl-meta}$), 6.95–6.87 (m, 1H; CH$_{aryl-para}$), 4.33 (s, 2 H; NH$_2$) ppm. ^{13}C{^{1}H} NMR (75 MHz, DMSO-d$_6$, 298.0 K): δ = 157.4 (s; C=O), 140.0 (s; NC$_{aryl}$), 128.6 (s; C$_{aryl-meta}$), 121.4 (s; C$_{aryl-para}$), 118.2 (s; C$_{aryl-ortho}$) ppm. MS (EI, 70 eV, 70 °C) *m/z* (%): 151 (27) $[M]^{+\cdot}$, 136 (1), 85 (100). Elemental analysis calcd for $C_7H_9N_3O$: C 55.65, H 6.00, N 27.86, found: C 55.69, H 6.10, N 20.69.

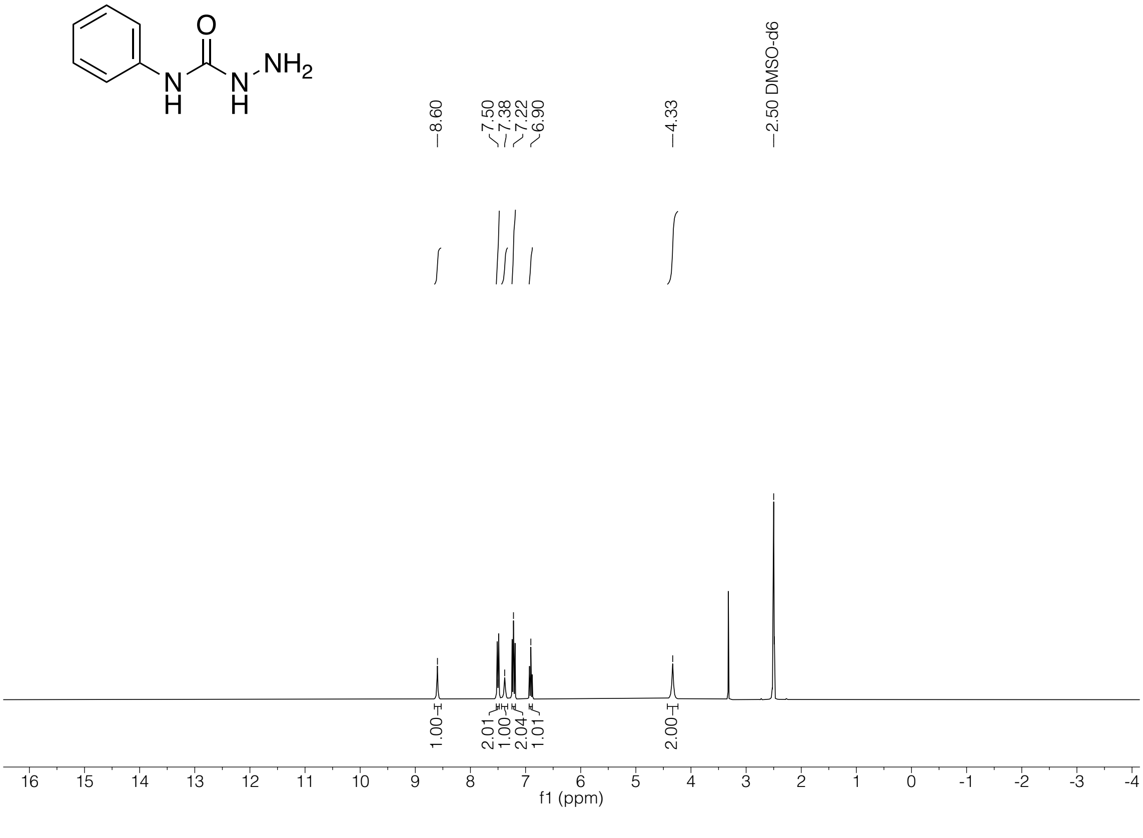

Figure SF7. ^{1}H NMR (300 MHz, DMSO-d$_6$, 298.0 K) spectrum of **2-Ph**.

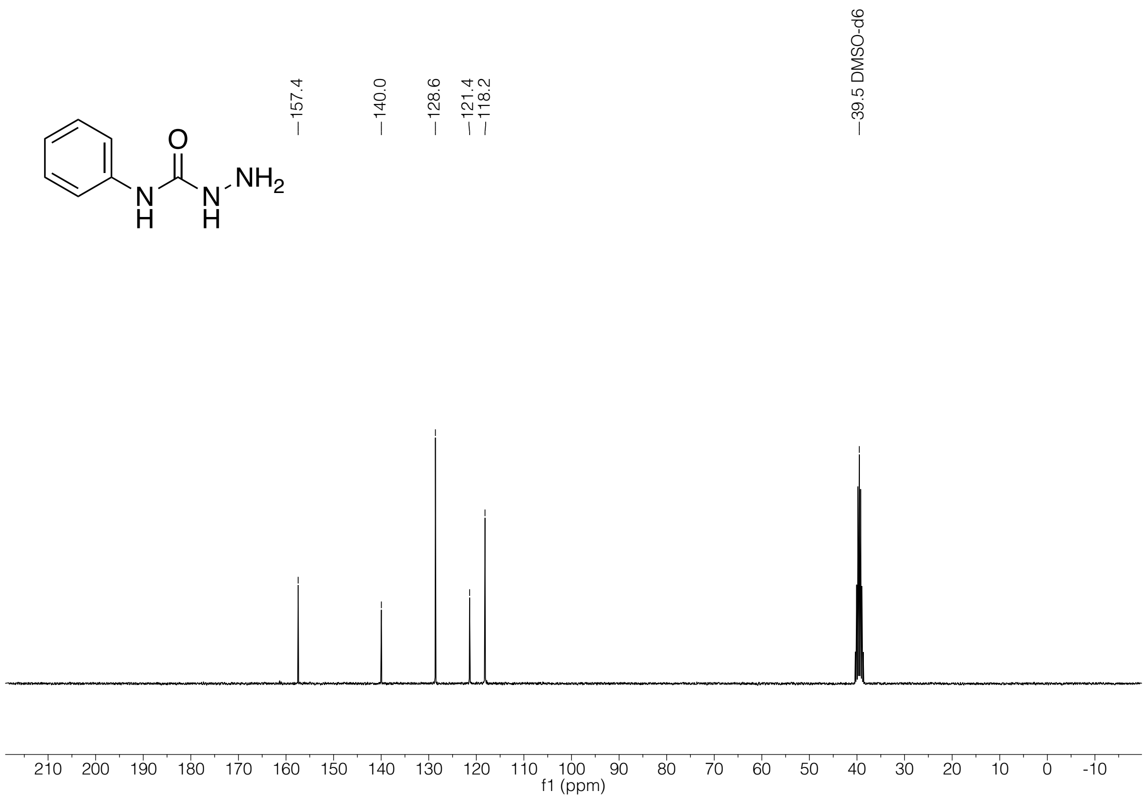

Figure SF8. $^{13}C\{^1H\}$ NMR (75 MHz, DMSO-d_6, 298.0 K) spectrum of **2-Ph**.

2-Mes: Urea used: **1-Mes** (80 g, 0.45 mol). Colorless, needle-shaped, air-stable crystals. Yield: 73 g, 84%. ^1H NMR (300 MHz, DMSO-d_6, 298.0 K): δ = 7.72 (s, 1H; C_{aryl}NH), 7.16 (s, 1H; H_2NN*H*), 6.83 (d, J = 1 Hz, 2H; CH_{meta}), 4.28 (s, 2 H; NH_2), 2.21 (s, 3H; $CH_{3\text{-}para}$), 2.11 (s, 6H; $CH_{3\text{-}ortho}$) ppm. $^{13}C\{^1H\}$ NMR (75 MHz, DMSO-d_6, 298.0 K): δ = 158.3 (s; C=O), 135.2 (s; NC_{aryl}), 134.3 (s; $C_{aryl\text{-}meta}$), 133.3 (s; $C_{aryl\text{-}para}$), 128.1 (s; $C_{aryl\text{-}ortho}$), 20.5 (s; $CH_{3\text{-}para}$), 18.2 (s; $CH_{3\text{-}ortho}$) ppm. MS (EI, 70 eV, 75 °C) *m/z* (%): 193 (9) $[M]^{+\cdot}$,178 (12), 161 (100), 146 (46), 133 (56), 120 (37), 91 (45). Elemental analysis calcd for $C_{10}H_{15}N_3O$: C 62.15, H 7.82, N 21.74, found: C 61.94, H 8.01, N 22.00.

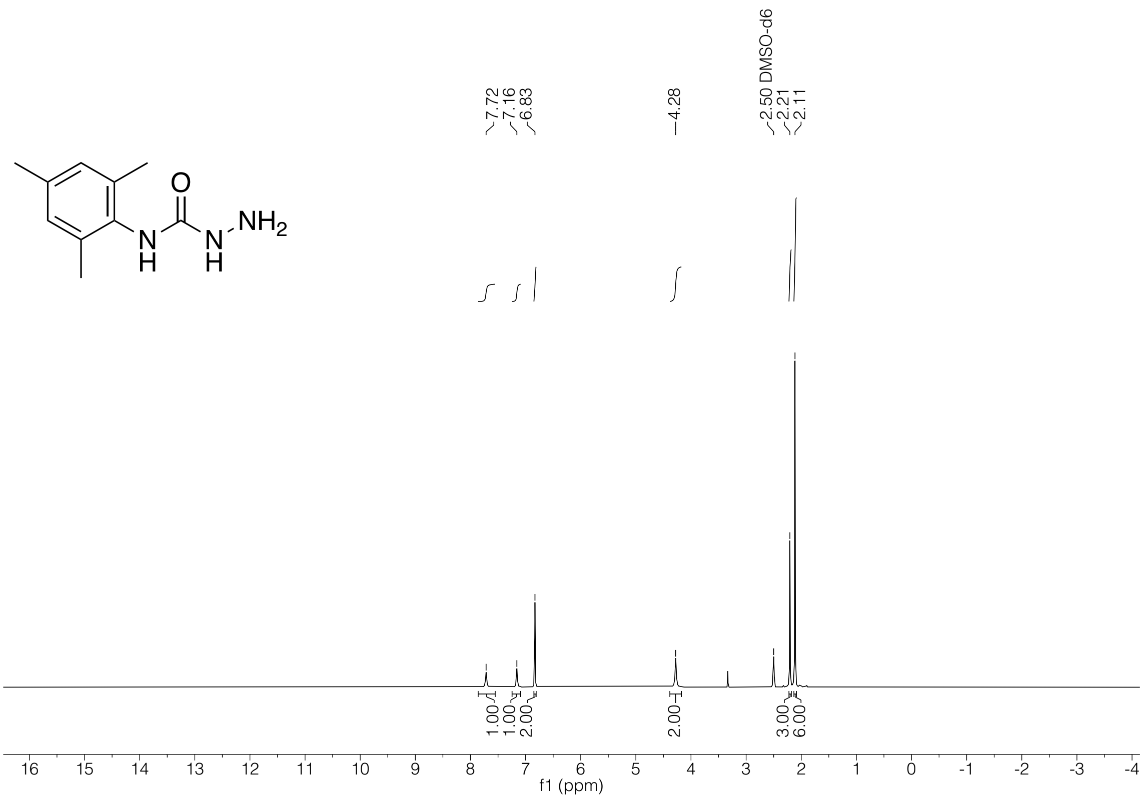

Figure SF9. ^{1}H NMR (300 MHz, DMSO-d$_6$, 298.0 K) spectrum of **2-Mes**.

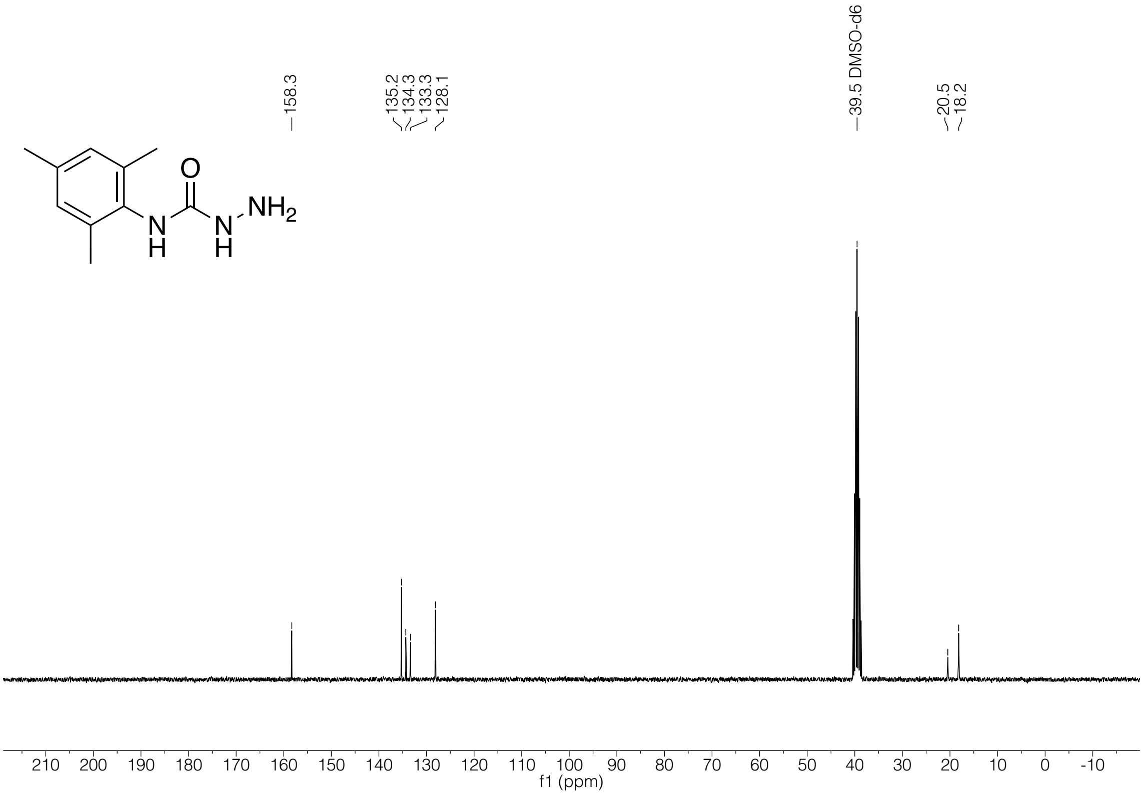

Figure SF10. ^{13}C{^{1}H} NMR (75 MHz, DMSO-d$_6$, 298.0 K) spectrum of **2-Mes**.

2-Dipp: Urea used: **1-Dipp** (99 g, 0.45 mol). Colorless, needle-shaped, air-stable crystals. Yield: 84 g, 79%. ^{1}H NMR (300 MHz, DMSO-d$_6$, 298.0 K): δ = 7.78 (s, 1H; NH), 7.24–7.18 (m, 1H; CH$_{aryl\text{-}para}$), 7.18 (s, 1H; H$_2$NN*H*), 7.14–7.08 (m, 2H; CH$_{aryl\text{-}meta}$), 4.33 (s, 2 H; NH$_2$), 3.15 (sept, J = 7 Hz, 2H; CH$_{iPr}$), 1.13 (d, J = 7 Hz, 12H; CH$_{3\text{-}iPr}$) ppm. ^{13}C{^{1}H} NMR (75 MHz, DMSO-d$_6$, 298.0 K): δ = 159.1 (s; C=O), 146.5 (s; C$_{aryl\text{-}ortho}$), 133.1 (s; N*C$_{aryl}$*), 126.7 (s; C$_{aryl\text{-}para}$), 122.6 (s; C$_{aryl\text{-}meta}$), 27.8 (s; CH$_{iPr}$), 23.5 (s; CH$_{3\text{-}iPr}$) ppm. MS (EI, 70 eV, 90 °C) *m/z* (%): 235 (4) [M]$^{+\cdot}$, 204 (100), 188 (21), 160 (9), 146 (17), 91 (7). Elemental analysis calcd for $C_{13}H_{21}N_3O$: C 66.35, H 8.99, N 17.86, found: C 66.14, H 8.72, N 17.54.

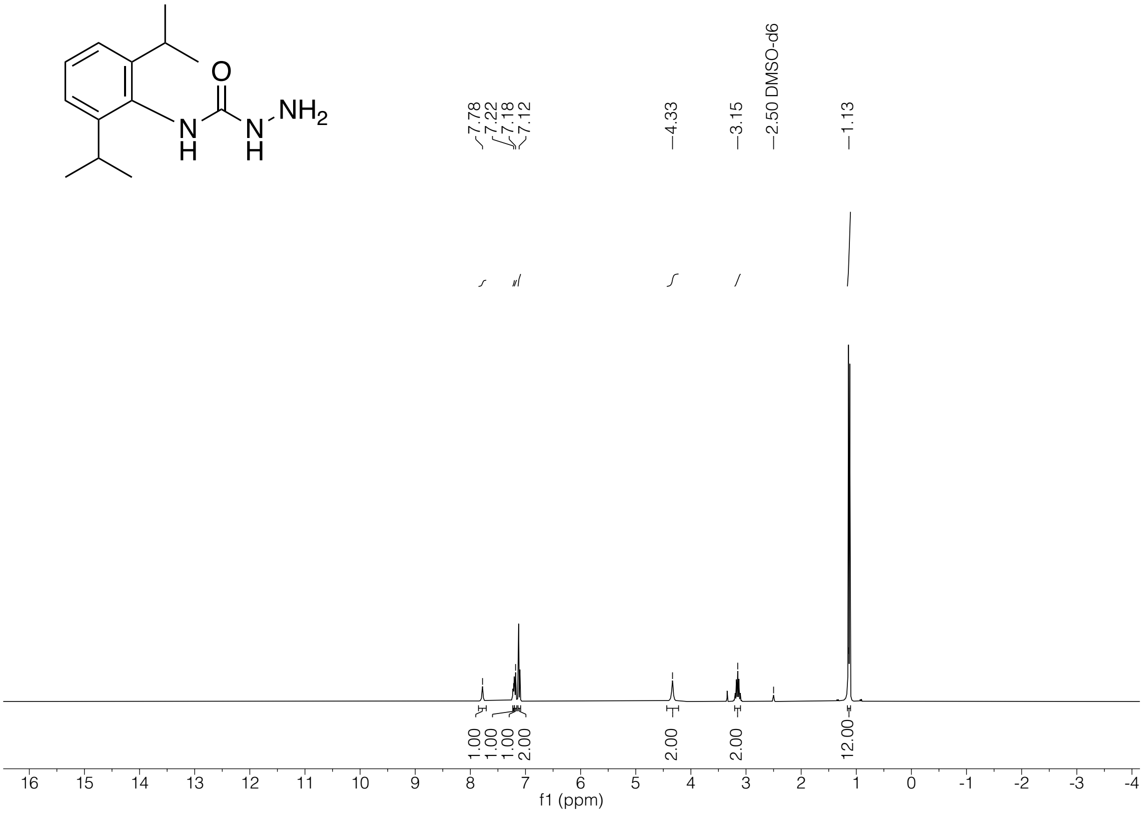

Figure SF11. ^{1}H NMR (300 MHz, DMSO-d$_6$, 298.0 K) spectrum of **2-Dipp**.

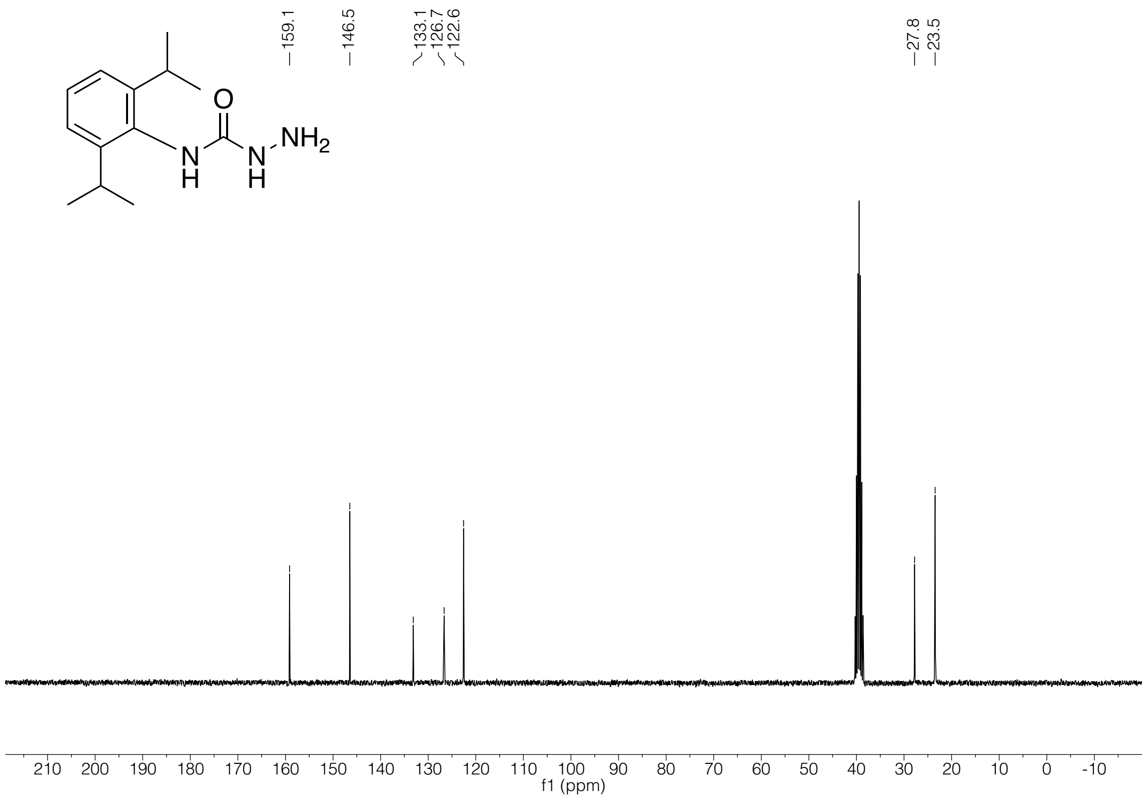

Figure SF12. $^{13}C\{^{1}H\}$ NMR (75 MHz, DMSO-d$_6$, 298.0 K) spectrum of **2-Dipp**.

c. Syntheses of semicarbazones 3

The corresponding semicarbazide **2** (0.3 mol, 1 eq.), acetone (44 ml, 0.6 mol, 2 eq.) and acetic acid (17 ml, 0.3 mol, 1 eq.) were suspended in 400 ml ethanol and refluxed for 3 h. After cooling at 5 °C the desired product crystallized as colorless, needle-shaped, air-stable crystals. The crystals were filtered off, washed with ice-cold ethanol and dried in vacuo.

An alternative synthesis can be carried out in a microwave: The corresponding semicarbazide **2** (0.1 mol, 1 eq.), acetone (15 ml, 0.2 mol, 2 eq.) and acetic acid (0.6 ml, 0.03 mol, 0.3 eq.) were suspended in 65 ml ethanol and reacted at a maximum of 300 W at 120 °C for 45 min. The product precipitates again as needle shaped, air-stable crystals upon cooling. The yield does not differ from conventional heating.

3-Ph: Semicarbazide used: **2-Ph** (45 g, 0.3 mol). Colorless, needle-shaped, air-stable crystals. Yield: 55 g, 96%. ^{1}H NMR (300 MHz, DMSO-d$_6$, 298.0 K): δ = 9.38 (s, 1H; C_{aryl}NH), 8.68 (s, 1H; *H*NN(CH_3)$_2$), 7.62–7.55 (m, 2H; $CH_{aryl\text{-}ortho}$), 7.30–7.22 (m, 2H; $CH_{aryl\text{-}meta}$), 7.02–6.93 (m, 1H; $CH_{aryl\text{-}para}$), 1.97 (s, 3 H; CH_3), 1.87 (s, 3 H; CH_3) ppm. ^{13}C{^{1}H} NMR (75 MHz, DMSO-d$_6$, 298.0 K): δ = 153.6 (s; C=O), 148.3 (s; C=N), 139.9 (s; NC$_{aryl}$), 128.5 (s; $C_{aryl\text{-}meta}$), 122.1 (s; $C_{aryl\text{-}para}$), 119.1 (s; $C_{aryl\text{-}ortho}$), 24.9 (s; N=C(CH_3)$_2$), 17.1 (s; N=C(CH_3)$_2$) ppm. MS (EI, 70 eV, 90 °C) *m/z* (%): 191 (62) $[M]^{+\cdot}$, 176 (18), 93 (37), 77 (28), 72 (100). Elemental analysis calcd for $C_{10}H_{13}N_3O$: C 62.81, H 6.85, N 21.97, found: C 62.98, H 6.71, N 22.15.

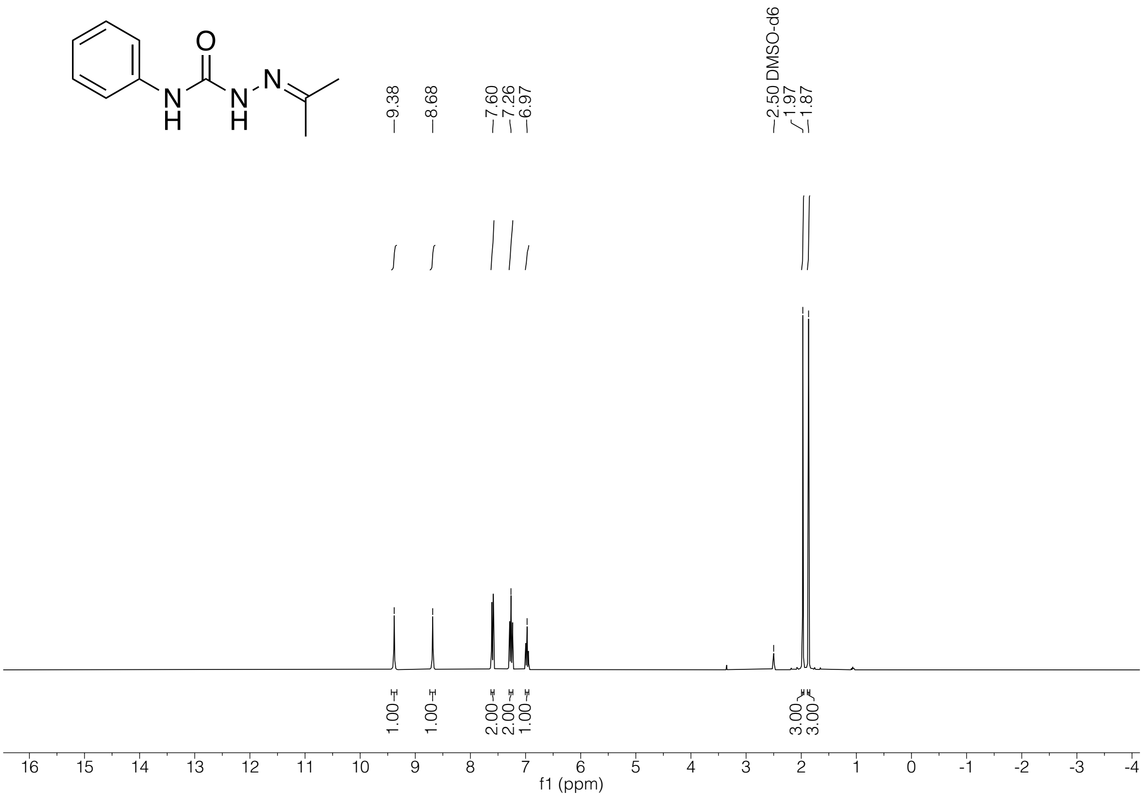

Figure SF13. ^{1}H NMR (300 MHz, DMSO-d$_6$, 298.0 K) spectrum of **3-Ph**.

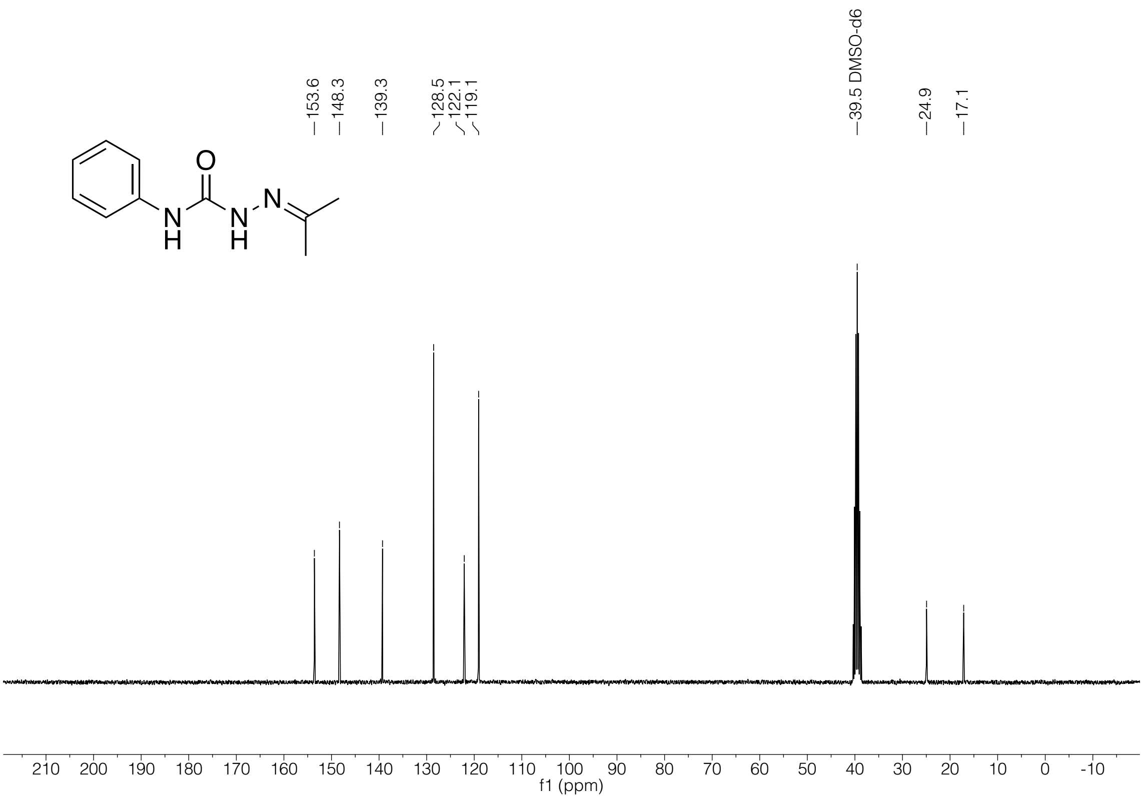

Figure SF14. $^{13}C\{^1H\}$ NMR (75 MHz, DMSO-d_6, 298.0 K) spectrum of **3-Ph**.

3-Mes: Semicarbazide used: **2-Mes** (58 g, 0.3 mol). Colorless, needle-shaped, air-stable crystals. Yield: 66 g, 94%. ^1H NMR (300 MHz, DMSO-d_6, 298.0 K): δ = 9.34 (s, 1H; $C_{aryl}NH$), 8.12 (s, 1H; *H*NN(CH_3)2), 6.86 (d, J = 1 Hz, 2H; CH_{meta}), 2.23 (s, 3H; $CH_{3\text{-para}}$), 2.13 (s, 6H; $CH_{3\text{-ortho}}$), 1.94 (s, 3 H; CH_3), 1.86 (s, 3 H; CH_3) ppm. $^{13}C\{^1H\}$ NMR (75 MHz, DMSO-d_6, 298.0 K): δ = 154.5 (s; C=O), 147.1 (s; C=N), 135.5 (s; NC_{aryl}), 134.7 (s; $C_{aryl\text{-meta}}$), 133.1 (s; $C_{aryl\text{-para}}$), 128.1 (s; $C_{aryl\text{-ortho}}$), 25.0 (s; N=C(CH_3)$_2$), 20.5 (s; $CH_{3\text{-para}}$), 18.2 (s; $CH_{3\text{-ortho}}$), 17.0 (s; N=C(CH_3)$_2$) ppm. MS (EI, 70 eV, 100 °C) *m/z* (%): 233 (38) $[M]^{+\cdot}$,161 (13), 135 (28), 72 (100). Elemental analysis calcd for $C_{10}H_{13}N_3O$: C 62.81, H 6.85, N 21.97, found: C 62.81, H 6.70, N 22.11.

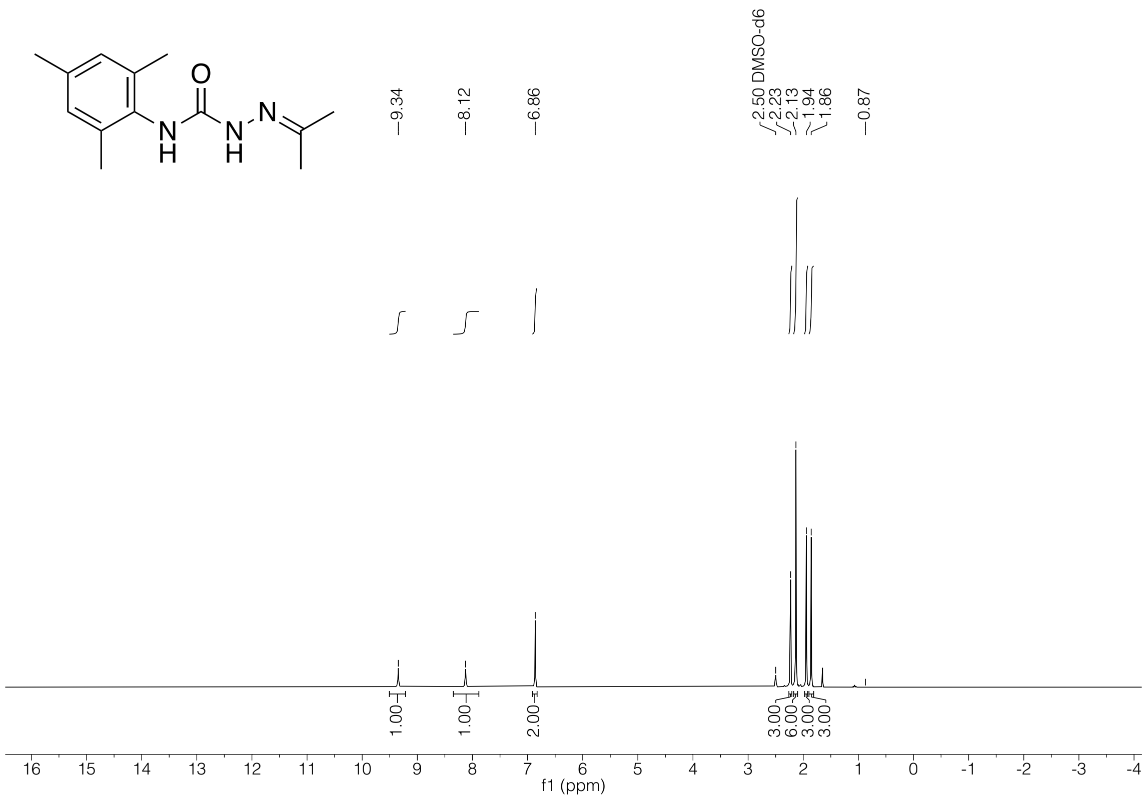

Figure SF15. ^{1}H NMR (300 MHz, DMSO-d$_6$, 298.0 K) spectrum of **3-Mes**.

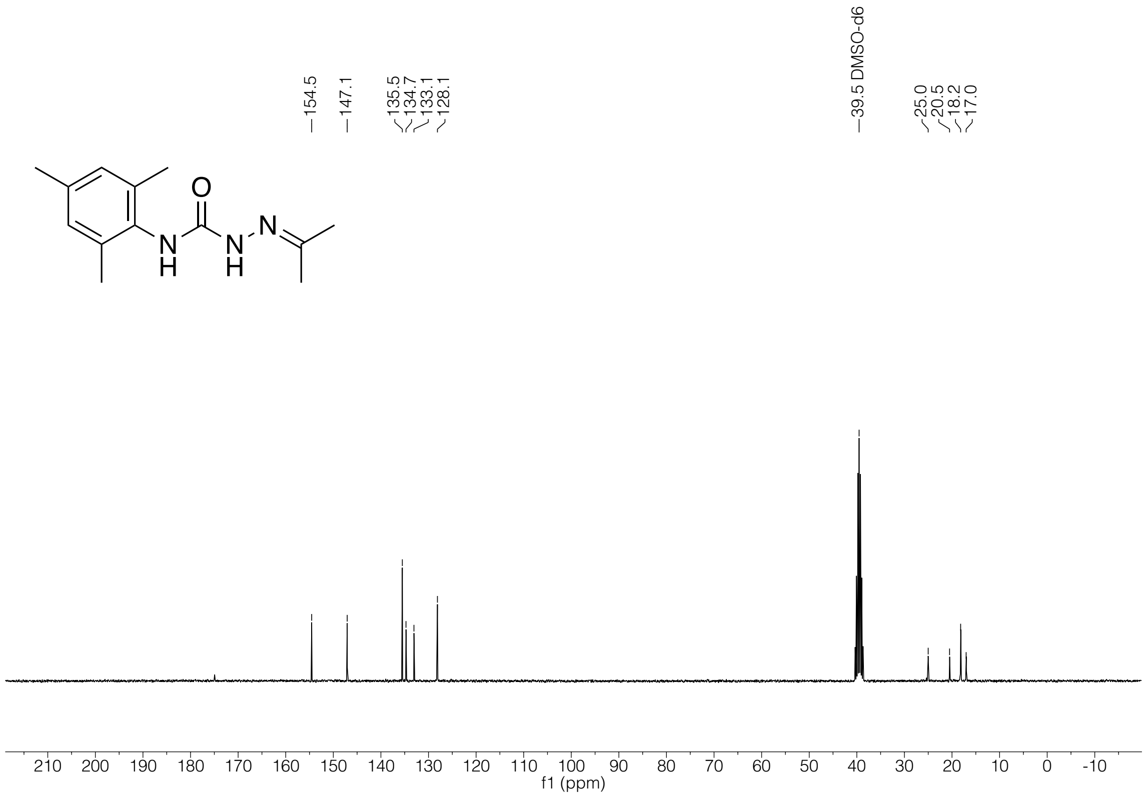

Figure SF16. ^{13}C{^{1}H} NMR (75 MHz, DMSO-d$_6$, 298.0 K) spectrum of **3-Mes**.

3-Dipp: Semicarbazide used: **2-Dipp** (70 g, 0.3 mol). Colorless, needle-shaped, air-stable crystals. Yield: 80 g, 97%. ^{1}H NMR (300 MHz, DMSO-d$_6$, 298.0 K): δ = 9.24 (s, 1H; C$_{aryl}$NH), 8.03 (s, 1H; *H*NN(CH$_3$)$_2$), 7.28–7.19 (m, 1H; CH$_{aryl\text{-}para}$), 7.18–7.09 (m, 2H; CH$_{aryl\text{-}meta}$), 3.13 (sept, *J* = 7 Hz, 2H; CH$_{iPr}$), 1.96 (s, 6H; CH$_{3\text{-}iPr}$), 1.86 (s, 6H; CH$_{3\text{-}iPr}$) ppm. ^{13}C{^{1}H} NMR (75 MHz, DMSO-d$_6$, 298.0 K): δ = 155.4 (s; C=O), 147.1 (s; C=N), 146.6 (s; C$_{aryl\text{-}ortho}$), 132.8 (s; N*C$_{aryl}$*), 127.0 (s; C$_{aryl\text{-}para}$), 122.7 (s; C$_{aryl\text{-}meta}$), 28.0 (s; CH$_{iPr}$), 25.1 (s; N=C(CH$_3$)$_2$), 23.5 (s; CH$_{3\text{-}iPr}$), 17.0 (s; N=C(CH$_3$)$_2$) ppm. MS (EI, 70 eV, 80 °C) *m/z* (%): 275 (20) [M]$^{+\cdot}$, 204 (100), 188 (27), 176 (14), 117 (15), 91 (23), 72 (77). Elemental analysis calcd for $C_{16}H_{25}N_3O$: C 69.78, H 9.15, N 15.26, found: C 69.53, H 8.99, N 15.17.

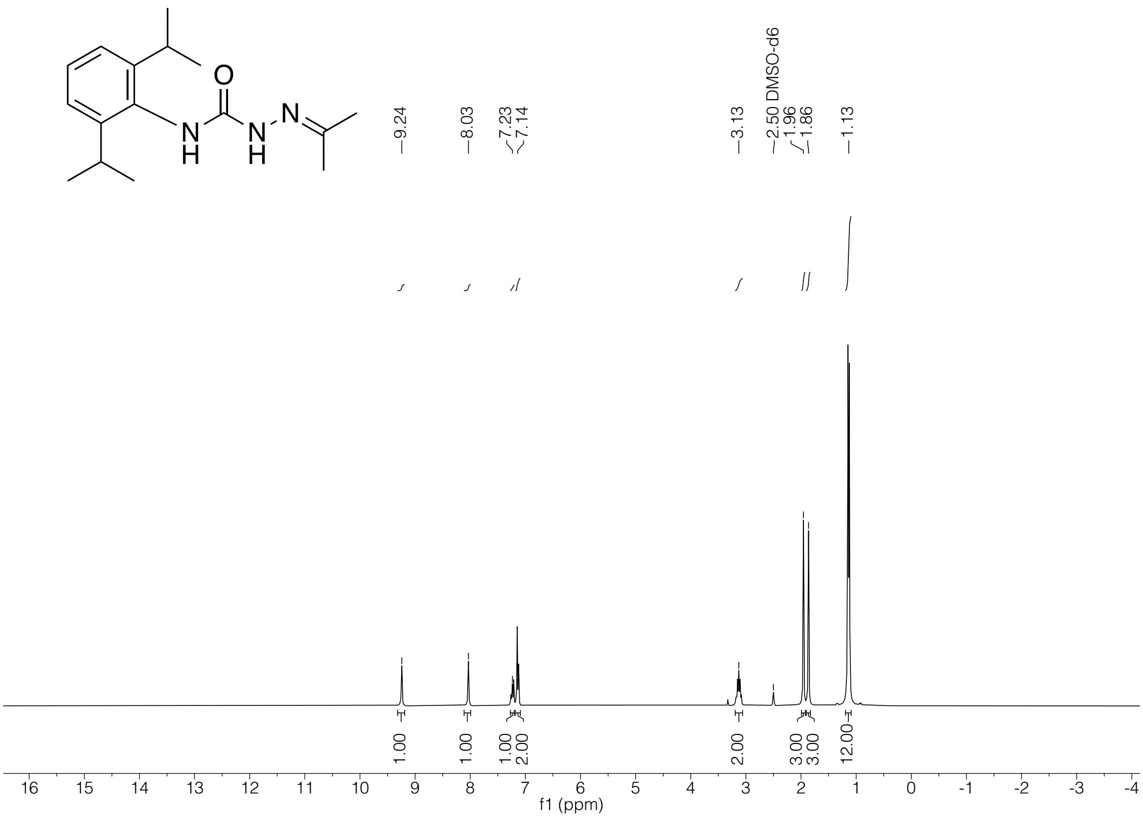

Figure SF17. ^{1}H NMR (300 MHz, DMSO-d$_6$, 298.0 K) spectrum of **3-Dipp**.

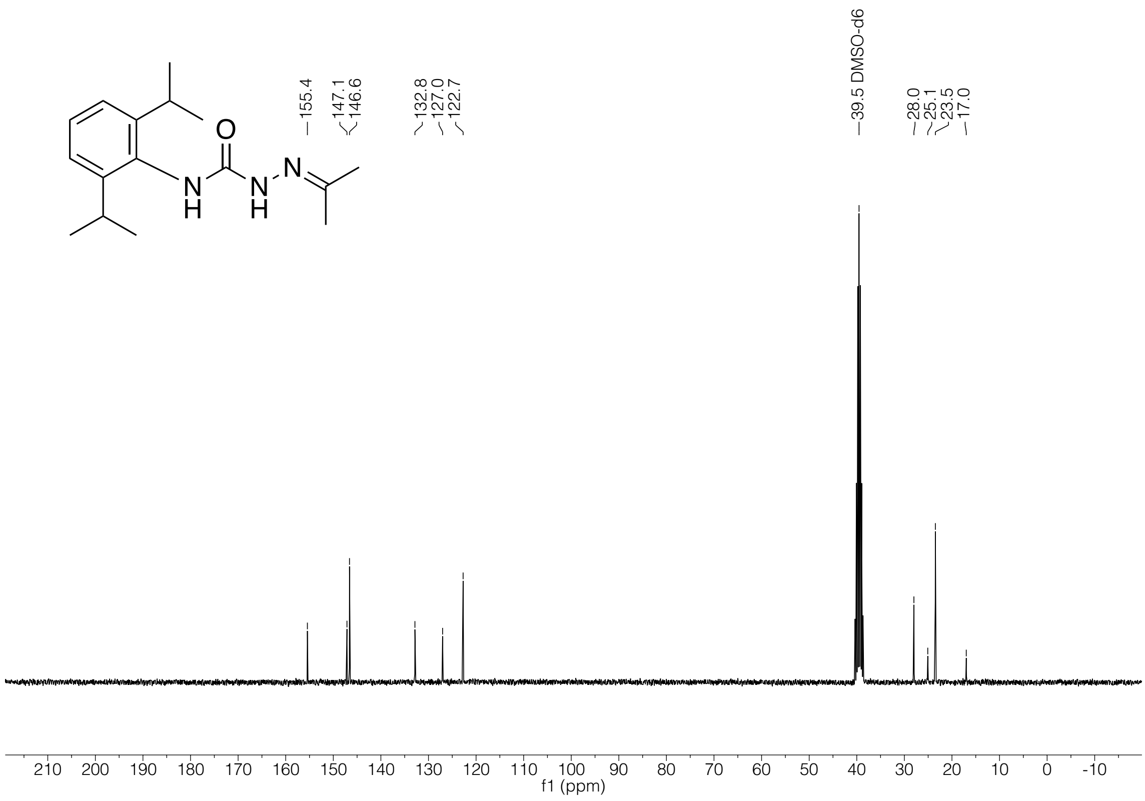

Figure SF18. $^{13}C\{^1H\}$ NMR (75 MHz, DMSO-d_6, 298.0 K) spectrum of **3-Dipp**.

d. Syntheses of 2-imino-Δ^3-1,3,4-oxadiazoles 4

The corresponding semicarbazone (0.25 mol, 1 eq.) was dissolved in 400 ml of dichloromethane and a suspension of lead(IV) acetate (133 g, 0.3 mol, 1.2 eq.) in 500 ml dichloromethane was slowly added at 0 °C over a period of 30 min. After addition of the lead(IV) acetate, the cold bath was removed and the reaction mixture was stirred for 2 h at ambient temperature. Subsequently, 7 ml of ethylene glycol (0.13 mol, 0.5 eq.) were slowly added and after 15 min. of stirring at ambient temperature the yellow solution was extracted two times with 250 ml of water, three times with 250 ml of a saturated aqueous solution of sodium hydrogencarbonate and finally two times with 200 ml of brine. Drying over magnesium sulfate and evaporation of the solvent afforded a yellow oil, that immediately crystallized in the cold.

4-Ph: Semicarbazone used: **3-Ph** (34 g, 0.25 mol). Yellow, block-shaped, air-stable crystals. Yield: 55 g, 99%. ^{1}H NMR (300 MHz, C_6D_6, 298.0 K): δ = 7.39–7.24 (m, 2H; $CH_{aryl\text{-}ortho}$), 7.15–7.09 (m, 2H; $CH_{aryl\text{-}meta}$), 6.96 (m, 1H; $CH_{aryl\text{-}para}$), 0.87 (s, 6 H; $C(CH_3)_2$) ppm. $^{13}C\{^1H\}$ NMR (75 MHz, C_6D_6, 298.0 K): δ = 160.3 (s; O-C=N), 144.3 (s; NC_{aryl}), 129.3 (s; $C_{aryl\text{-}meta}$), 126.7 (s; $C_{aryl\text{-}para}$), 125.1 (s; $C_{aryl\text{-}ortho}$), 121.9 (s; *C*$(CH_3)_2$), 22.9 C(*C*$H_3)_2$ ppm. MS (EI, 70 eV, 100 °C) *m/z* (%): 189 (2) $[M]^{+\cdot}$, 133 (21), 119 (53), 103 (100), 91 (41), 77 (27). Elemental analysis calcd for $C_{10}H_{11}N_3O$: C 63.48, H 5.86, N 22.21, found: C 63.76, H 5.81, N 22.00.

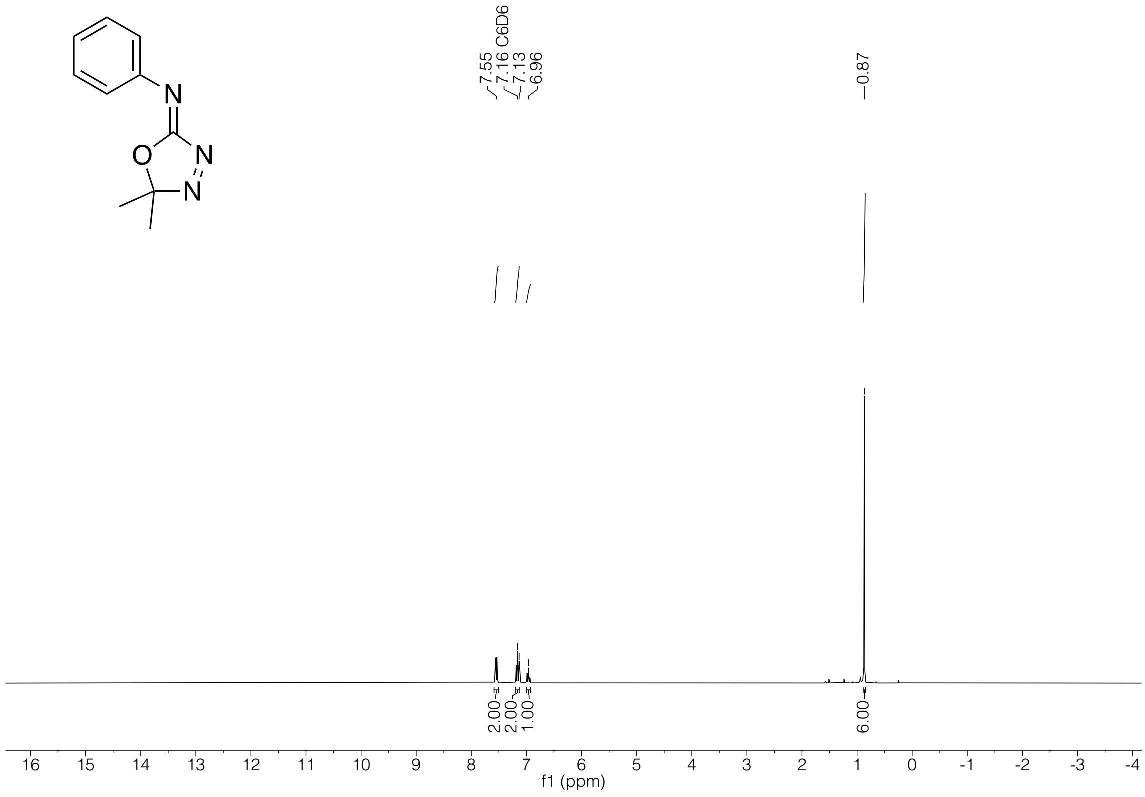

Figure SF19. ^{1}H NMR (300 MHz, C_6D_6, 298.0 K) spectrum of **4-Ph**.

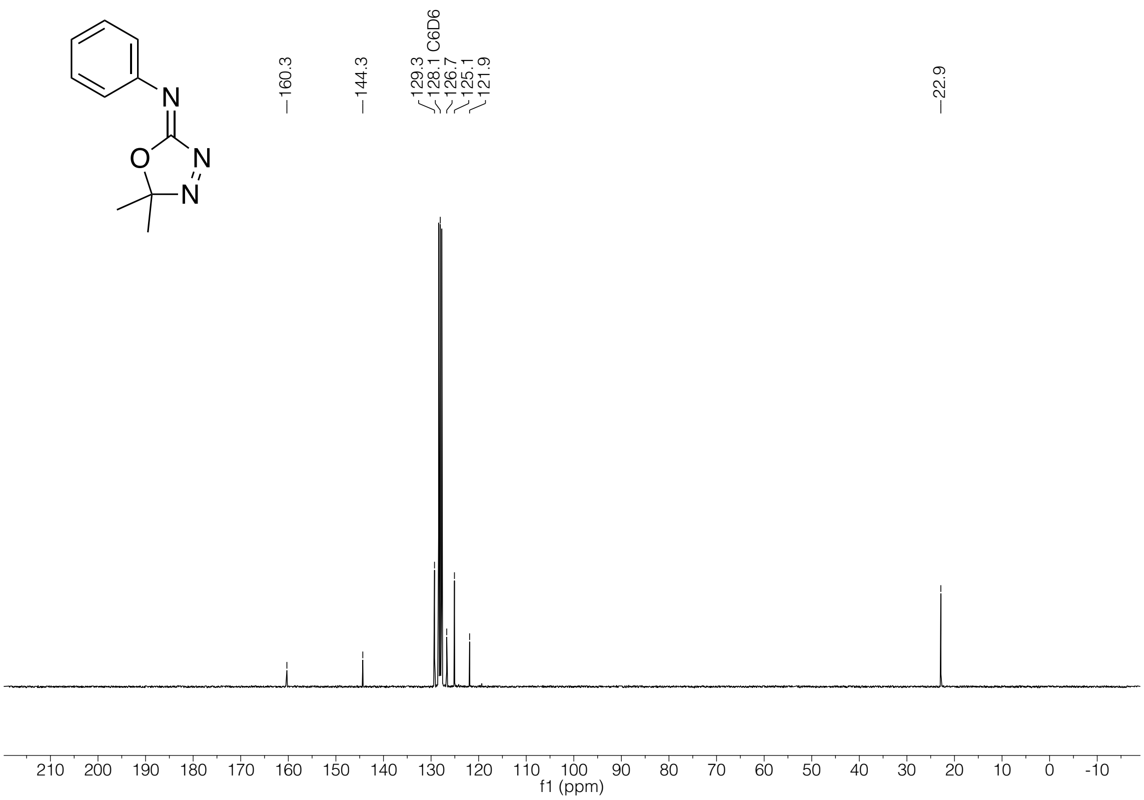

Figure SF20. $^{13}C\{^{1}H\}$ NMR (75 MHz, C_6D_6, 298.0 K) spectrum of **4-Ph**.

4-Mes: Semicarbazone used: **3-Mes** (45 g, 0.25 mol). Yellow, block-shaped, air-stable crystals. Yield: 66 g, 99%. ^{1}H NMR (300 MHz, C_6D_6, 298.0 K): δ = 6.79 (d, J = 1 Hz, 2H; CH_{meta}), 2.15 (s, 3H; $CH_{3\text{-}para}$), 2.12 (s, 6H; $CH_{3\text{-}ortho}$), 1.00 (s, 6 H; $C(CH_3)_2$) ppm. $^{13}C\{^{1}H\}$ NMR (75 MHz, C_6D_6, 298.0 K): δ = 159.9 (s; O-C=N), 141.2 (s; NC_{aryl}), 133.4 (s; $C_{aryl\text{-}meta}$), 129.0 (s; $C_{aryl\text{-}para}$), 127.4 (s; $C_{aryl\text{-}ortho}$), 121.0 (s; $\mathit{C}(CH_3)_2$), 23.3 (s; $C(\mathit{C}H_3)_2$), 20.8 (s; $CH_{3\text{-}para}$), 18.3 (s; $CH_{3\text{-}ortho}$) ppm. MS (EI, 70 eV, 110 °C) *m/z* (%): 231 (25) $[M]^{+\cdot}$,175 (45), 145 (45), 130 (100), 117 (12), 103 (17), 91 (35), 77 (31). Elemental analysis calcd for $C_{13}H_{17}N_3O$: C 67.51, H 7.41, N 18.17, found: C 67.82, H 7.67, N 18.39.

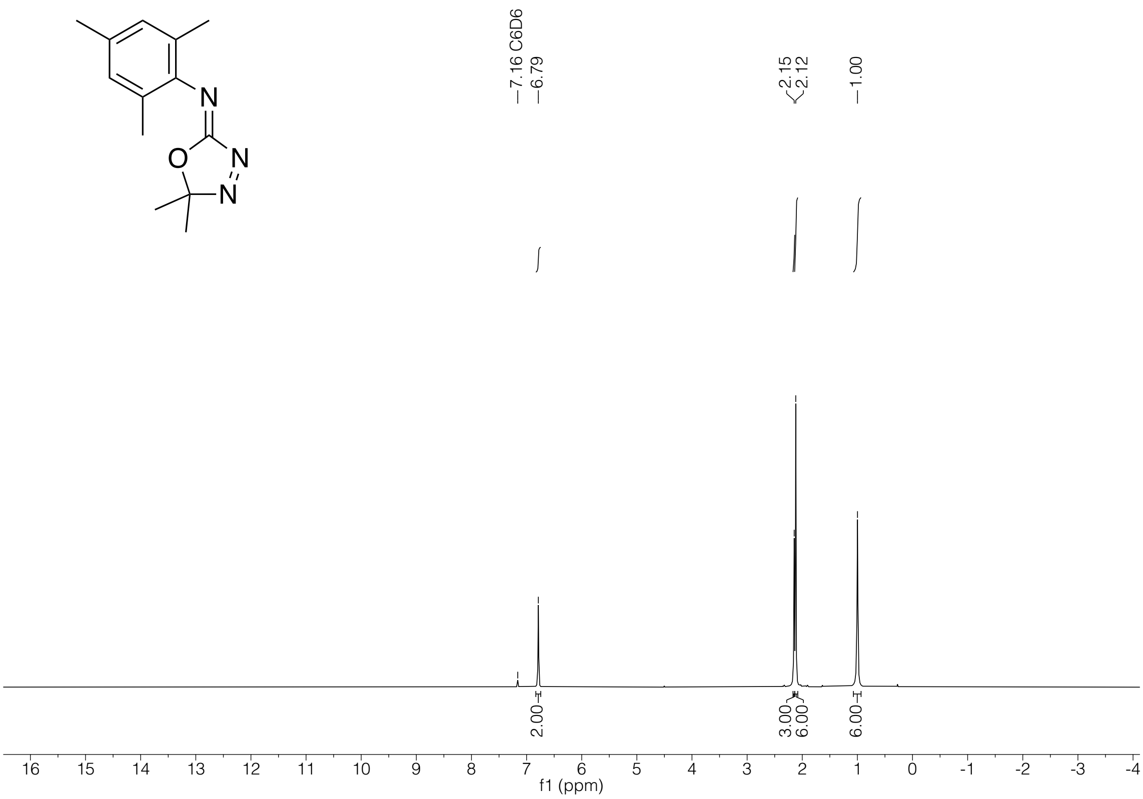

Figure SF21. ^{1}H NMR (300 MHz, C_6D_6, 298.0 K) spectrum of **4-Mes**.

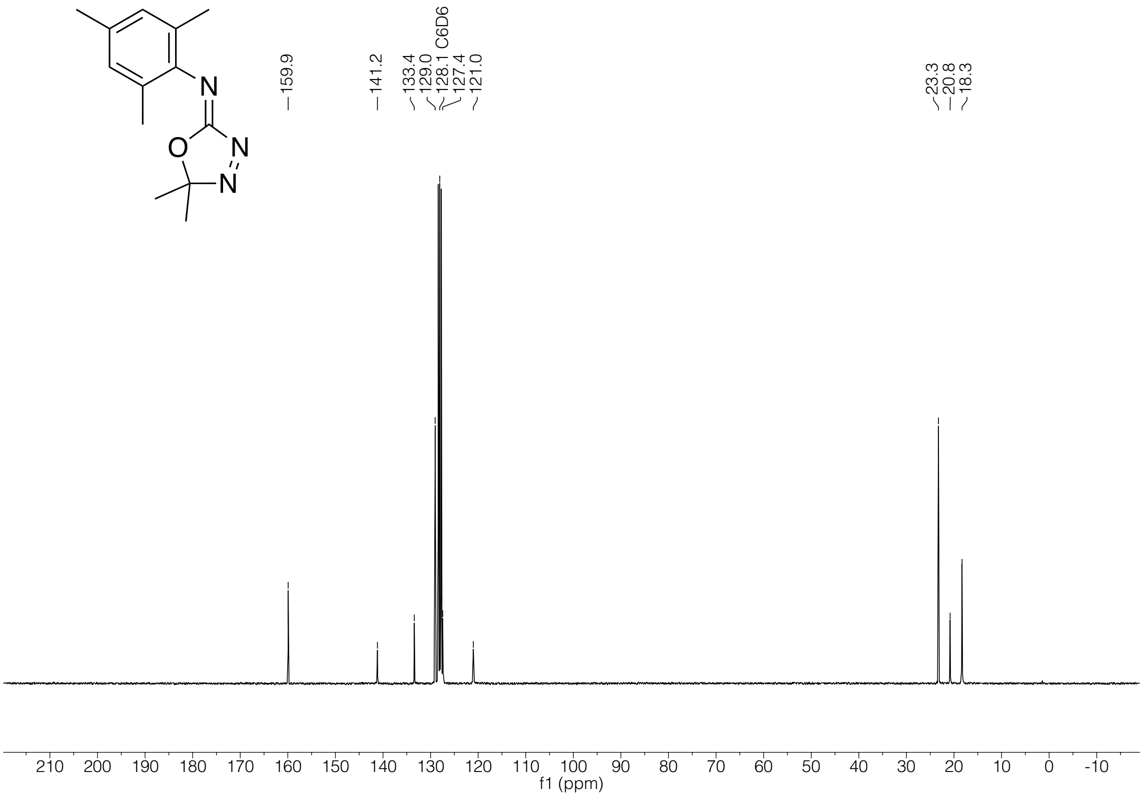

Figure SF22. $^{13}C\{^{1}H\}$ NMR (75 MHz, C_6D_6, 298.0 K) spectrum of **4-Mes**.

4-Dipp: Semicarbazone used: **3-Dipp** (55 g, 0.25 mol). Yellow, block-shaped, air-stable crystals. Yield: 80 g, 99%. ^{1}H NMR (300 MHz, C_6D_6, 298.0 K): δ = 7.20–7.16 (m, 1H; $CH_{aryl\text{-}para}$), 7.14–7.09 (m, 2H; $CH_{aryl\text{-}meta}$), 3.13 (sept, J = 7 Hz, 2H; CH_{iPr}), 1.21 (d, J = 7 Hz, 12H; $CH_{3\text{-}iPr}$), 1.02 (s, 6 H; $CH_{3\text{-}iPr}$) ppm. ^{13}C{^{1}H} NMR (75 MHz, C_6D_6, 298.0 K): δ = 159.1 (s; O-C=N), 141.2 (s; NC_{aryl}), 133.4 (s; $C_{aryl\text{-}meta}$), 129.0 (s; $C_{aryl\text{-}para}$), 127.4 (s; $C_{aryl\text{-}ortho}$), 121.0 (s; $C(CH_3)_2$), 23.3 (s; CH_3-iPr), 20.8 (s; CH_{iPr}), 18.3 (s; $CH_{3\text{-}iPr}$) ppm. MS (EI, 70 eV, 110 °C) m/z (%): 235 (4) $[M]^{+\cdot}$, 204 (100), 188 (21), 146 (17), 128 (7), 91 (7). Elemental analysis calcd for $C_{16}H_{23}N_3O$: C 70.30, H 8.48, N 15.37, found: C 70.57, H 8.31, N 15.08.

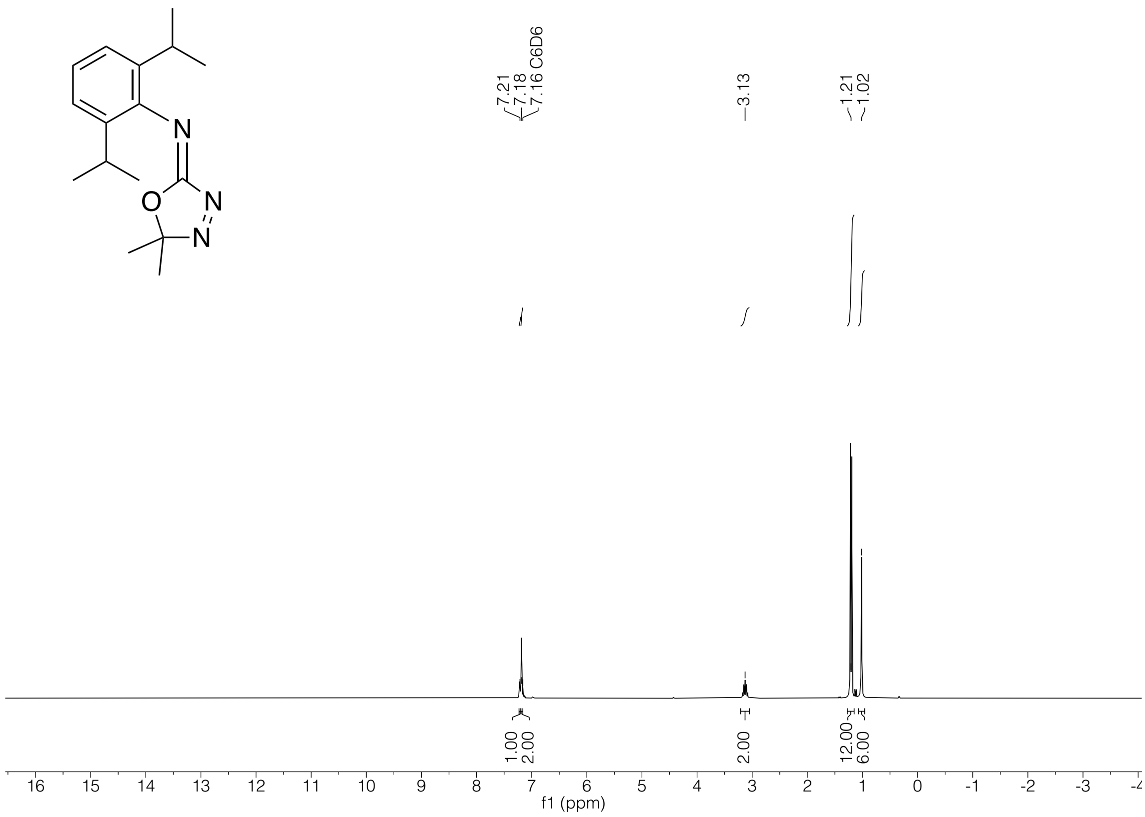

Figure SF23. ^{1}H NMR (300 MHz, C_6D_6, 298.0 K) spectrum of **4-Dipp**.

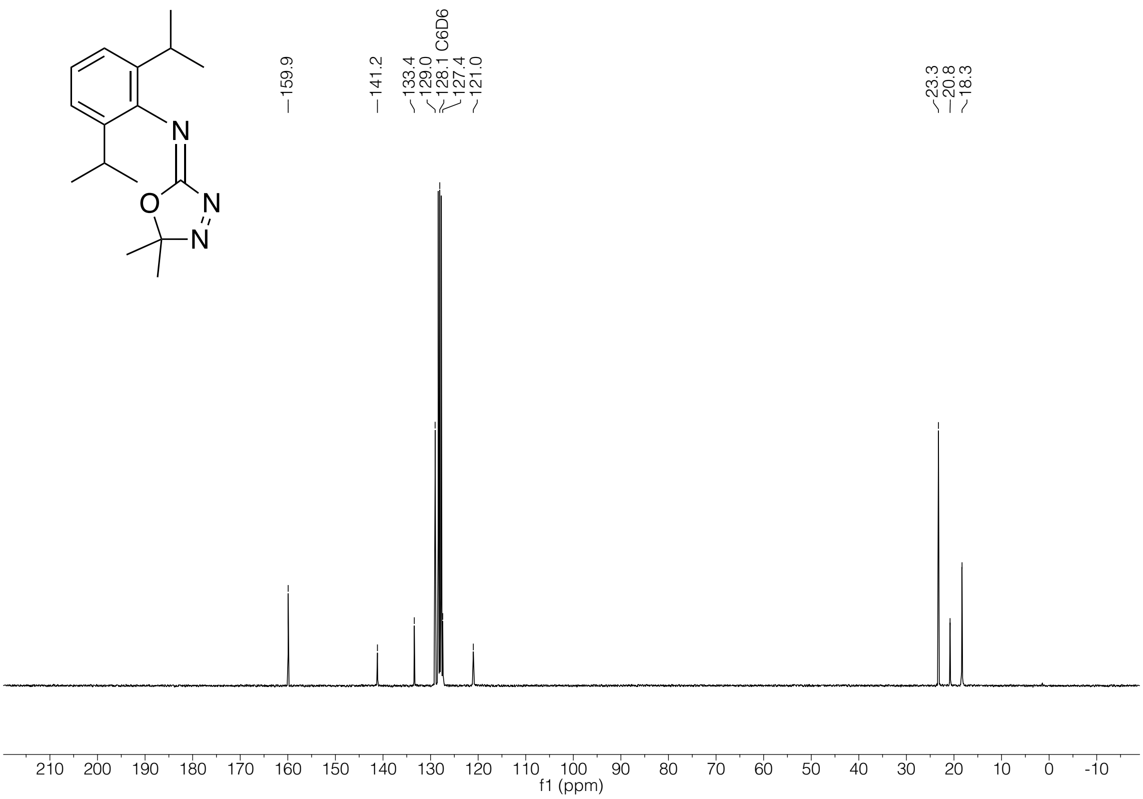

Figure SF24. $^{13}C\{^{1}H\}$ NMR (75 MHz, C_6D_6, 298.0 K) spectrum of **4-Dipp**.

e. Syntheses of precursors 5

The corresponding 1,3,4-oxadiazole (20 mmol, 1 eq.) was dissolved in 75 ml dichloromethane, 14 ml triethylamine (100 mmol, 5 eq.) was added and the solution was cooled to -80 °C. After addition of the corresponding acyl chloride (30 mmol, 1.5 eq.) at -80 °C over a period of 10 min, the cold bath was removed and the solution was stirred at ambient temperature for 15 h. The reaction was quenched by adding 100 ml of water. The organic phase was separated and extracted twice with 100 ml of water, then twice with 250 ml of a saturated aqueous solution of sodium bicarbonate and twice with 100 ml of brine. Drying over magnesium sulphate and evaporation of the solvent afforded a colorless oil which crystallized immediately in the cold at 0 °C. Though not necessary to improve the purity, all precursors **5** can be sublimed at 100 °C and $1*10^{-3}$ mbar, dynamic vacuum.

Ph-5-Me$_2$: 1,3,4-oxadiazole used: **4-Ph** (3.78 g, 20 mmol), acyl chloride used: isobutyryl chloride (3.1 ml, 30 mmol). Colorless, block-shaped, air-stable crystals. Yield: 5.03 g, 97%. ^{1}H NMR (300 MHz, C_6D_6, 298.0 K): δ = 7.34–7.30 (m, 2H; $CH_{aryl\text{-}ortho}$), 6.98–6.94 (m, 2H; $CH_{aryl\text{-}meta}$), 6.83–6.79 (m, 1H; $CH_{aryl\text{-}para}$), 1.33 (s, 3H; $OC(CH_3)_2$), 1.26 (s, 3H; $C(C(CH_3)_2)C$), 1.11 (s, 3H; $C(C(CH_3)_2)C$), 0.98 (s, 3H; $OC(CH_3)_2$) ppm. $^{13}C\{^1H\}$ NMR (75 MHz, C_6D_6, 298.0 K): δ = 169.8 (s; C=O), 135.8 (s; NC_{aryl}), 129.4 (s; $C_{aryl\text{-}meta}$), 125.8 (s; $C_{aryl\text{-}para}$), 125.2 (s; N*C*$C(CH_3)_2$), 120.9 (s; O*C*$(CH_3)_2$), 119.9 (s; $C_{aryl\text{-}ortho}$), 60.9 (s; C(*C*$(CH_3)_2$)C), 25.4 (s; OC(*C*$H_3)_2$), 23.2 (s; OC(*C*$H_3)_2$), 18.6(s; C(C(*C*$H_3)_2$)C), 17.1 (s; C(C(*C*$H_3)_2$)C) ppm [Some of the expected signals for aryl carbon atoms are not observed due to isochrony as well as overlapping with the solvent signal.]. MS (EI, 70 eV, 40 °C) *m/z* (%): 259 (6) $[M]^{+\cdot}$, 203 (8), 145 (100), 130 (23), 77 (10). Elemental analysis calcd for $C_{14}H_{17}N_3O_2$: C 64.85, H 6.61, N 16.20, found: C 64.89, H 6.50, N 16.18.

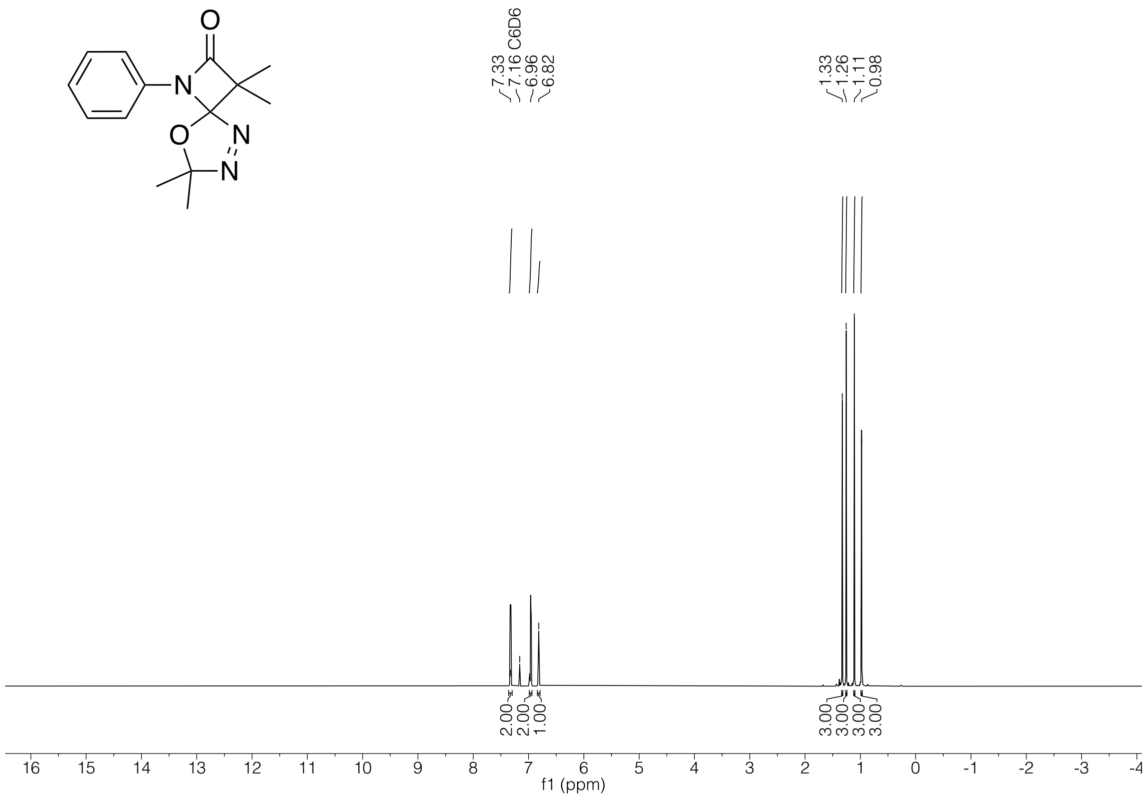

Figure SF25. ^{1}H NMR (300 MHz, C_6D_6, 298.0 K) spectrum of **Ph-5-Me$_2$**.

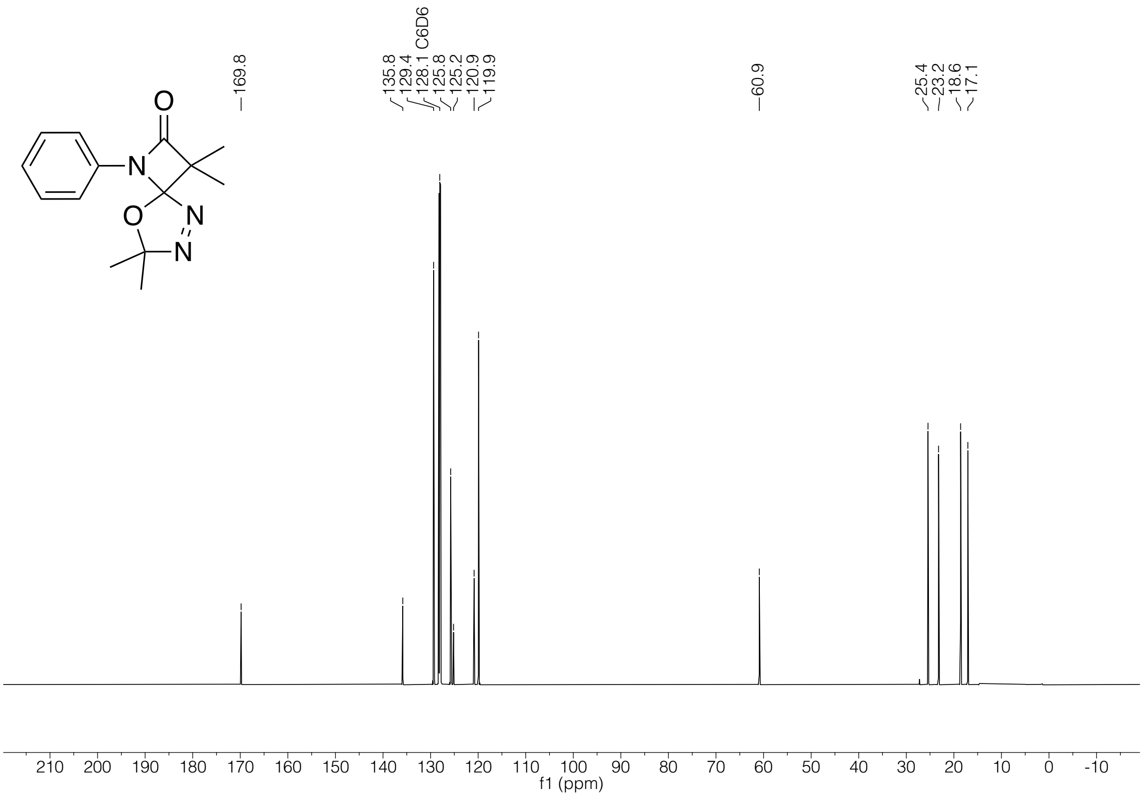

Figure SF26. $^{13}C\{^1H\}$ NMR (75 MHz, C_6D_6, 298.0 K) spectrum of **Ph-5-Me₂**.

Ph-5-Cl₂: 1,3,4-oxadiazole used: **4-Ph** (3.78 g, 20 mmol), acyl chloride used: dichloroacetyl chloride (2.9 ml, 30 mmol). Colorless, block-shaped, air-stable crystals. Yield: 5.94 g, 99%. ^1H NMR (300 MHz, C_6D_6, 298.0 K): δ = 7.10–7.06 (m, 2H; $CH_{aryl\text{-}ortho}$), 6.87–6.83 (m, 2H; $CH_{aryl\text{-}meta}$), 6.81–6.78 (m, 1H; $CH_{aryl\text{-}para}$), 1.24 (s, 3H; CH_3), 1.14 (s, 3H; CH_3) ppm. $^{13}C\{^1H\}$ NMR (75 MHz, C_6D_6, 298.0 K): δ = 158.1 (s; C=O), 133.4 (s; NC_{aryl}), 129.6 (s; $C_{aryl\text{-}meta}$), 127.5 (s; $C_{aryl\text{-}para}$), 124.8 (s; O*C*(C(CH_3))), 121.8 (s; N*C*C(Cl)₂), 120.7 (s; $C_{aryl\text{-}ortho}$), 87.0 (s; C(*C*(Cl)₂)C), 24.9 (s; OC(*C*H_3)₂), 22.7 (s; OC(*C*H_3)₂) ppm [Some of the expected signals for aryl carbon atoms are not observed due to isochrony as well as overlapping with the solvent signal.]. MS (EI, 70 eV, 35 °C) *m/z* (%): 299 (4) $[M]^{+\cdot}$,271 (7), 185 (46), 161 (99), 119 (100), 77 (42). Elemental analysis calcd for $C_{12}H_{11}Cl_2N_3O_2$: C 48.02, H 3.69, N 14.00, found: C 48.44, H 3.78, N 14.05.

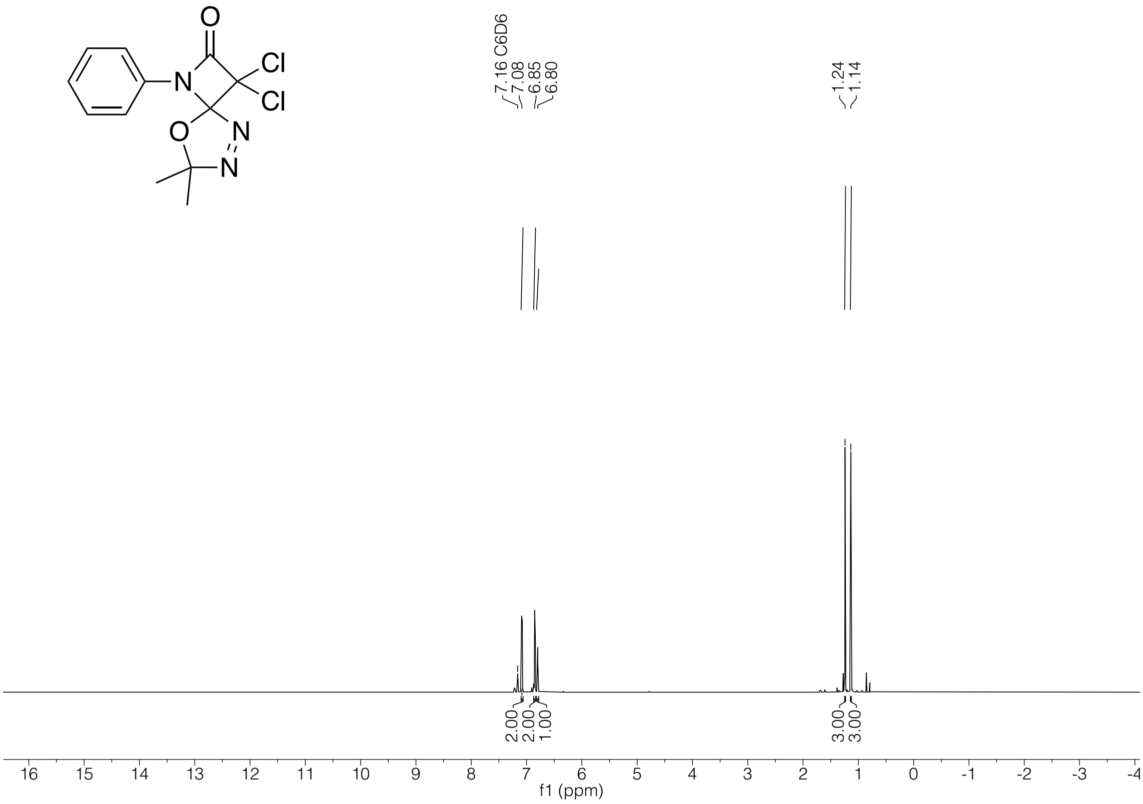

Figure SF27. ^{1}H NMR (300 MHz, C_6D_6, 298.0 K) spectrum of **Ph-5-Cl$_2$**.

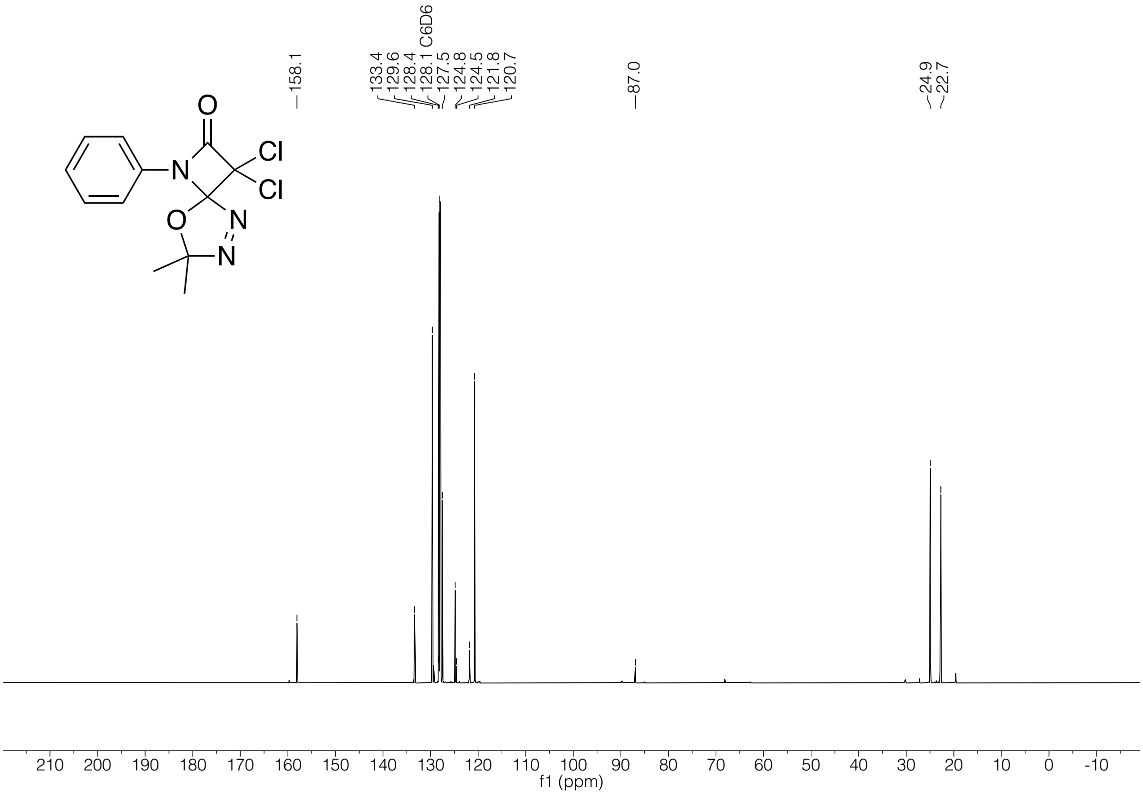

Figure SF28. $^{13}C\{^{1}H\}$ NMR (75 MHz, C_6D_6, 298.0 K) spectrum of **Ph-5-Cl$_2$**.

Ph-5-Ph$_2$: 1,3,4-oxadiazole used: **4-Ph** (3.78 g, 20 mmol), acyl chloride used: diphenylacetyl chloride (6.92 g, 30 mmol) dissolved in 20 ml of dichloromethane. Colorless, prism-shaped, air-stable crystals. Yield: 7.28 g, 95%. ^{1}H NMR (300 MHz, C_6D_6, 298.0 K): δ = 7.75–7.70 (m, 2H; CH_{aryl}), 7.62–7.75 (m, 2H; CH_{aryl}), 7.32–7.27 (m, 2H; CH_{aryl}), 7.15–6.99 (m, 6H; CH_{aryl}), 6.92–6.78 (m, 3H; CH_{aryl}), 1.04 (s, 3H; CH_3), 1.03 (s, 3H; CH_3) ppm. ^{13}C{^{1}H} NMR (75 MHz, C_6D_6, 298.0 K): δ = 166.6 (s; C=O), 137.4 (s; NC_{aryl}), 135.4 (s; C-C_{aryl}), 134.2 (s; C-C_{aryl}), 129.4 (s; C_{aryl}), 129.3 (s; C_{aryl}), 128.9 (s; C_{aryl}), 128.8 (s; C_{aryl}), 128.6 (s; C_{aryl}), 127.2 (s; C_{aryl}), 125.8 (s; N*C*C(Ph)$_2$), 123.1 (s; C_{aryl}), 122.2 (s; O*C*(CH_3)$_2$), 76.9 (s; C(*C*(Ph)$_2$)C), 24.0 (s; OC(*C*H_3)$_2$), 23.8 (s; OC(C(*C*H_3)$_2$ ppm [Some of the expected signals for aryl carbon atoms are not observed due to isochrony as well as overlapping with the solvent signal.]. MS (EI, 70 eV, 60 °C) *m/z* (%): 383 (6) $[M]^{+\cdot}$, 212 (25), 167 (100), 86 (31). Elemental analysis calcd for $C_{24}H_{21}N_3O_2$: C 75.18, H 5.52, N 10.96, found: C 75.60, H 5.41, N 10.94.

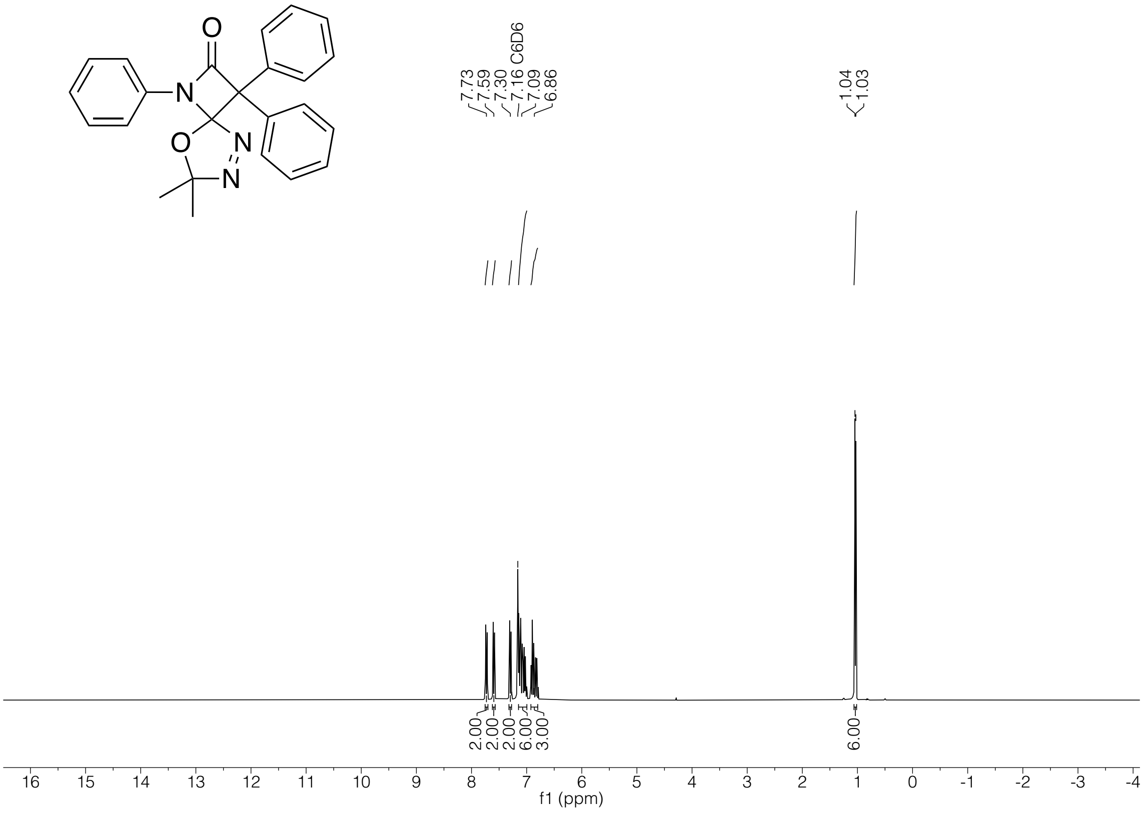

Figure SF29. ^{1}H NMR (300 MHz, C_6D_6, 298.0 K) spectrum of **Ph-5-Ph$_2$**.

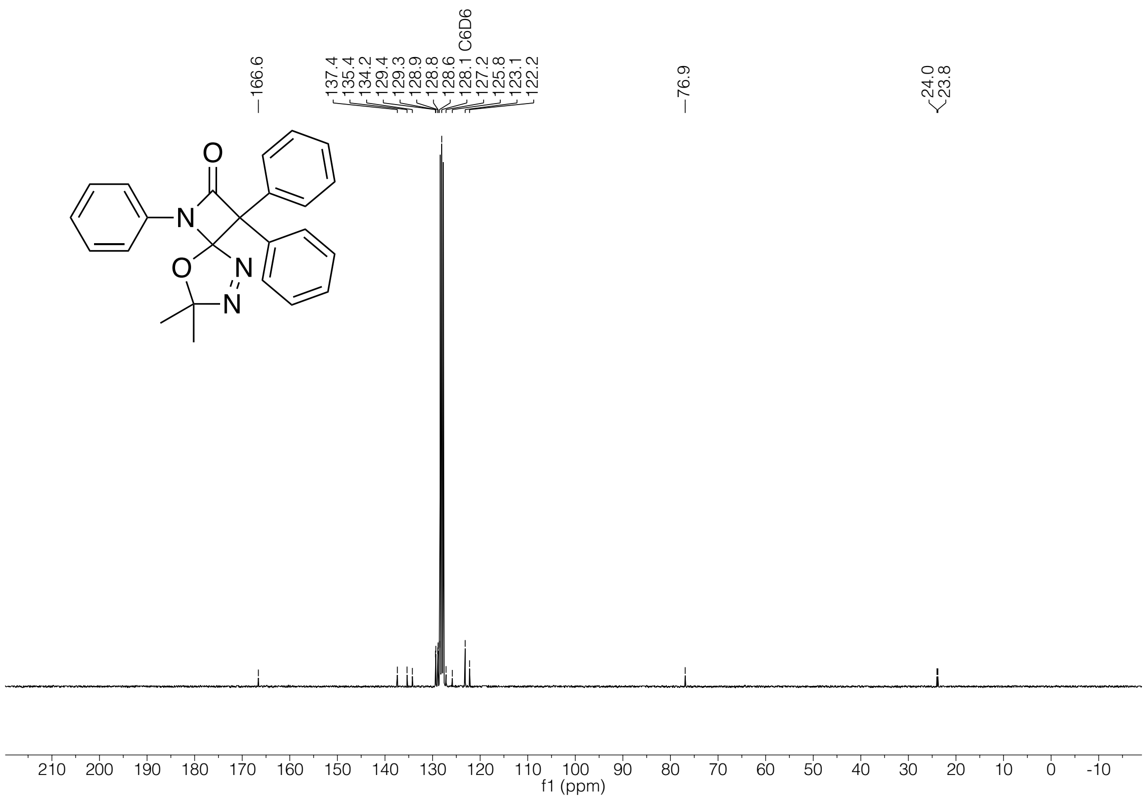

Figure SF30. $^{13}C\{^1H\}$ NMR (75 MHz, C_6D_6 , 298.0 K) spectrum of **Ph-5-Ph$_2$**.

Ph-5-sCy: 1,3,4-oxadiazole used: **4-Ph** (3.78 g, 20 mmol), acyl chloride used: cyclohexanecarbonyl chloride (4.0 ml, 30 mmol). Colorless, plate-shaped, air-stable crystals. Yield: 5.87 g, 98%. ^1H NMR (300 MHz, C_6D_6, 298.0 K): δ = 7.28–7.25 (m, 2H; $CH_{aryl\text{-}ortho}$), 7.00–6.95 (m, 2H; $CH_{aryl\text{-}meta}$), 6.86–6.82 (m, 1H; $CH_{aryl\text{-}para}$), 2.46–2.40 (m, 1H; CH_{aliph}), 2.03–1.97 (m, 1H; CH_{aliph}), 1.92–1.79 (m, 2H; CH_{aliph}), 1.52–1.44 (m, 2H; CH_{aliph}), 1.39–1.31 (m, 2H; CH_{aliph}), 1.30 (s, 3H; CH_3), 1.28–1.22 (m, 1H; CH_{aliph}), 1.13 (s, 3H; CH_3), 1.02–0.93 (m, 1H; CH_{aliph}) ppm. $^{13}C\{^1H\}$ NMR (75 MHz, C_6D_6, 298.0 K): δ = 169.9 (s; C=O), 135.5 (s; NC_{aryl}), 129.3 (s; $C_{aryl\text{-}meta}$), 126.0 (s; $C_{aryl\text{-}para}$), 125.5 (s; $N\mathit{C}C_{sCy}$), 121.1 (s; $O\mathit{C}(CH_3)_2$), 120.8 (s; $C_{aryl\text{-}ortho}$), 64.8 (s; $C(\mathit{C}_{sCy})C$), 29.5 (s; C_{aliph}), 28.3 (s; C_{aliph}), 25.8 (s; C_{aliph}), 25.6 (s; $OC(\mathit{C}H_3)_2$), 23.6 (s; $OC(\mathit{C}H_3)_2$), 23.4 (s; C_{aliph}), 23.2 (s; C_{aliph}) ppm [Some of the expected signals for aryl carbon atoms are not observed due to isochrony as well as overlapping with the solvent signal.]. MS (EI, 70 eV, 50 °C) *m/z* (%): 299 (4) $[M]^{+\cdot}$, 185 (100), 77 (12). Elemental analysis calcd for $C_{17}H_{21}N_3O_2$: C 68.20, H 7.07, N 14.04, found: C 68.26, H 6.93, N 13.89.

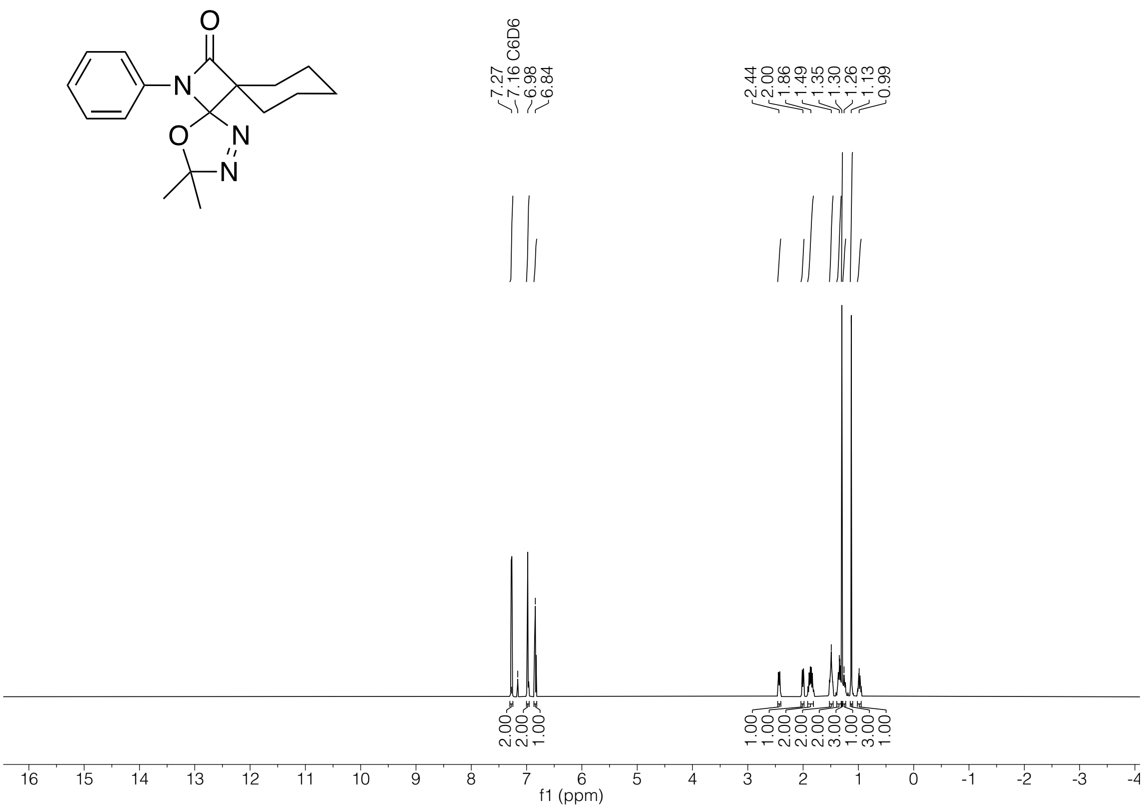

Figure SF31. ^{1}H NMR (300 MHz, C_6D_6, 298.0 K) spectrum of **Ph-5-sCy**.

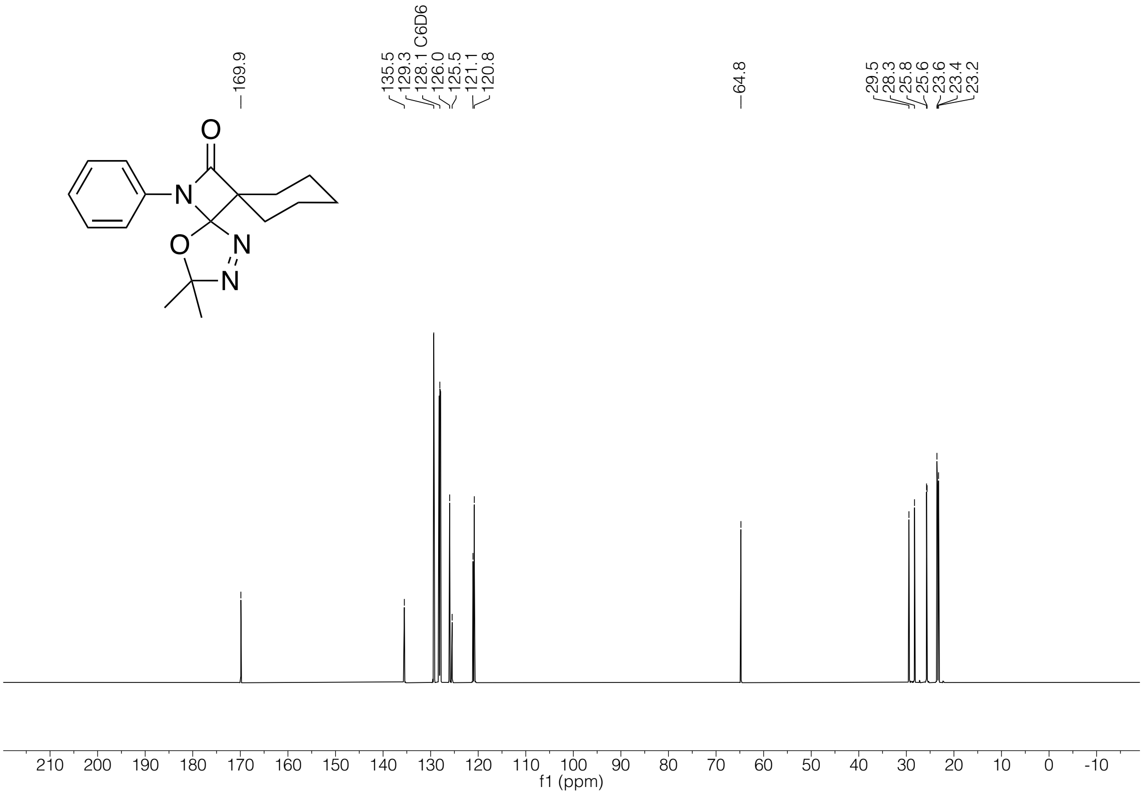

Figure SF32. ^{13}C{^{1} H} NMR (75 MHz, C_6D_6 , 298.0 K) spectrum of **Ph-5-sCy**.

Mes-5-Me$_2$: 1,3,4-oxadiazole used: **4-Mes** (4.63 g, 20 mmol), acyl chloride used: isobutyryl chloride (3.1 ml, 30 mmol). Colorless, block-shaped, air-stable crystals. Yield: 5.67 g, 94%. ^{1}H NMR (300 MHz, C_6D_6, 298.0 K): δ = 6.63–6.61 (m, 1H; CH_{meta}), 6.59–6.57 (m, 1H; CH_{meta}), 2.42 (s, 3H; $CH_{3\text{-}ortho}$), 2.25 (s, 3H; $CH_{3\text{-}ortho}$), 1.99 (s, 3H; $CH_{3\text{-}para}$), 1.48 (s, 3H; $C(C(CH_3)_2)C$), 1.29 (s, 3H; $C(C(CH_3)_2)C$), 1.09 (s, 3H; $OC(CH_3)_2$), 0.86 (s, 3H; $OC(CH_3)_2$) ppm. ^{13}C{^{1}H} NMR (75 MHz, C_6D_6, 298.0 K): δ = 171.1 (s; C=O), 138.8 (s; $C_{aryl\text{-}para}$), 138.3 (s; NC_{aryl}), 129.7 (s; $C_{aryl\text{-}meta}$), 129.2 (s; $C_{aryl\text{-}ortho}$), 127.4 (s; N*C*C(CH$_3$)$_2$), 120.6 (s; O*C*(CH$_3$)$_2$), 59.7 (s; C(*C*(CH$_3$)$_2$)C), 24.9 (s; OC(*C*H$_3$)$_2$), 24.4 (s; OC(*C*H$_3$)$_2$), 21.0 (s; $CH_{3\text{-}para}$), 19.1 (s; C(C(*C*H$_3$)$_2$)C), 18.8 (s; $CH_{3\text{-}ortho}$), 18.7 (s; $CH_{3\text{-}ortho}$), 17.8 (s; C(C(*C*H$_3$)$_2$)C) ppm [Some of the expected signals for aryl carbon atoms are not observed due to isochrony as well as overlapping with the solvent signal.]. MS (EI, 70 eV, 40 °C) *m/z* (%): 301 (4) $[M]^{+\cdot}$, 187 (100), 172 (61), 161 (50), 72 (6). Elemental analysis calcd for $C_{17}H_{23}N_3O_2$: C 67.75, H 7.69, N 13.94, found: C 67.96, H 7.56, N 13.67.

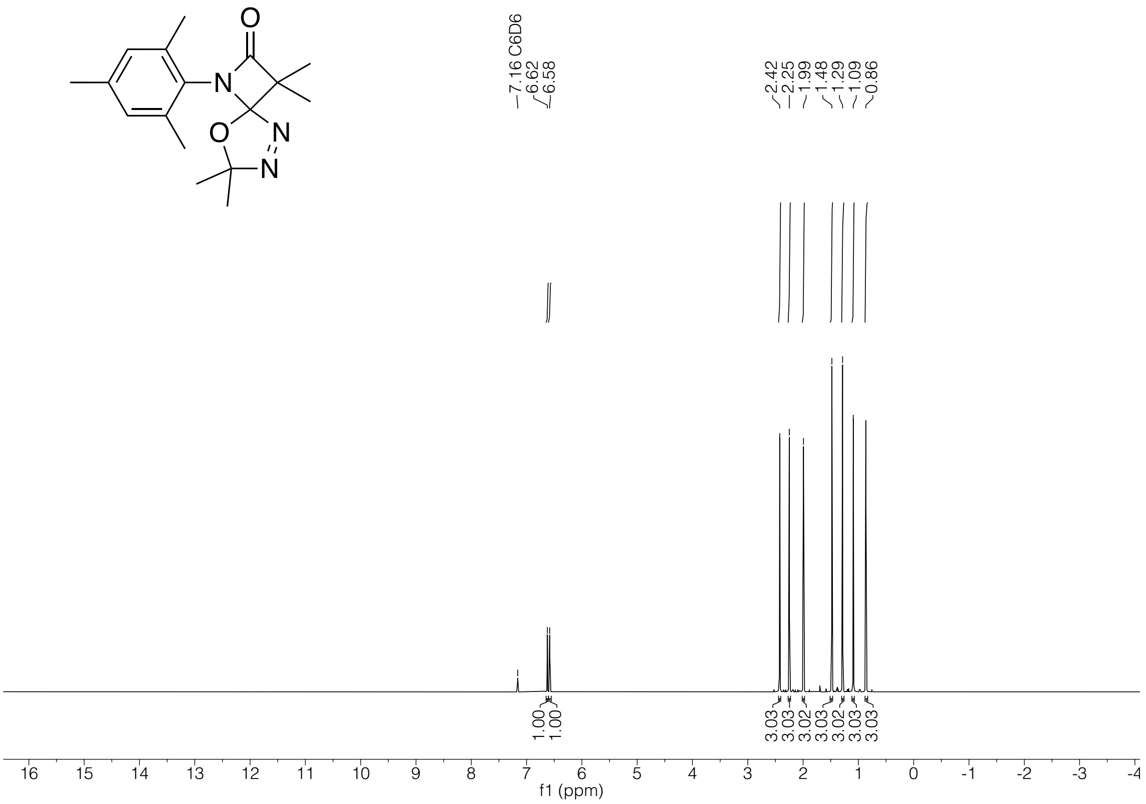

Figure SF33. ^{1}H NMR (300 MHz, C_6D_6, 298.0 K) spectrum of **Mes-5-Me$_2$**.

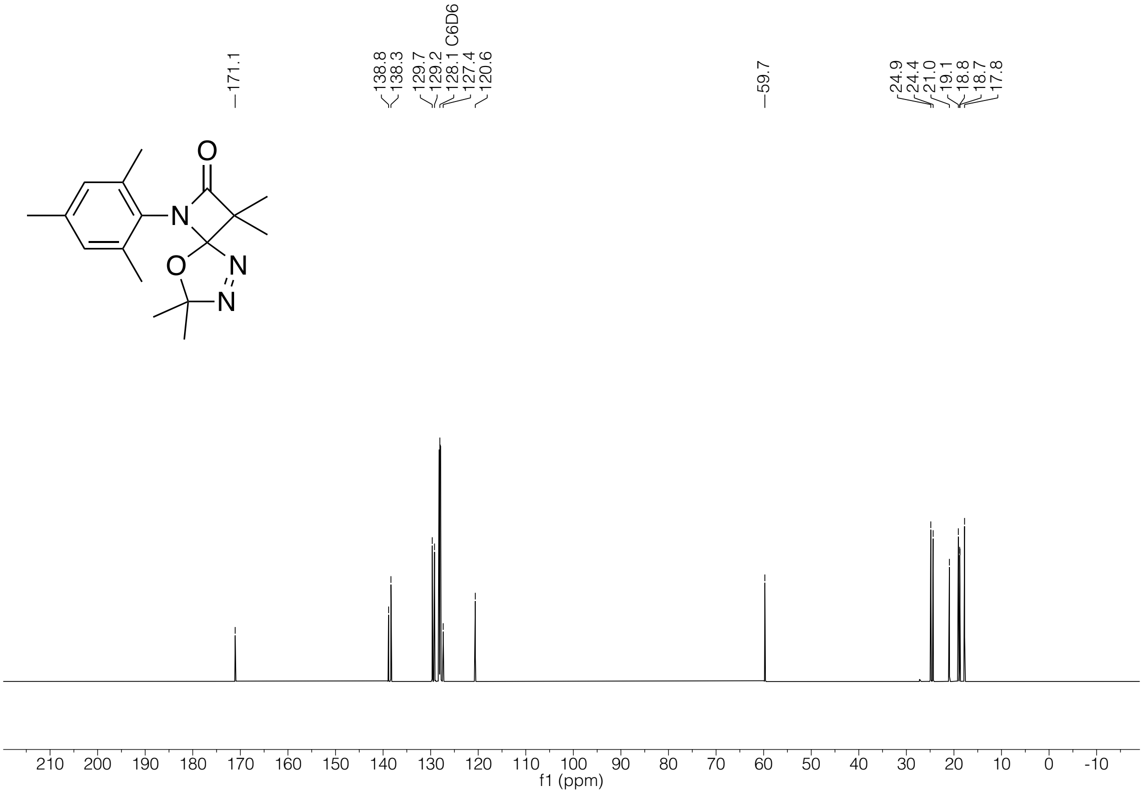

Figure SF34. $^{13}C\{^{1}H\}$ NMR (75 MHz, C_6D_6, 298.0 K) spectrum of **Mes-5-Me$_2$**.

Mes-5-Cl$_2$: 1,3,4-oxadiazole used: **4-Mes** (4.63 g, 20 mmol), acyl chloride used: dichloroacetyl chloride (2.9 ml, 30 mmol). Colorless, block-shaped, air-stable crystals. Yield: 6.64 g, 97%. ^{1}H NMR (300 MHz, C_6D_6, 298.0 K): δ = 6.55–6.52 (m, 1H; CH_{meta}), 6.50–6.46 (m, 1H; CH_{meta}), 2.38 (s, 3H; $CH_{3\text{-ortho}}$), 2.19 (s, 3H; $CH_{3\text{-ortho}}$), 1.94 (s, 3H; $CH_{3\text{-para}}$), 1.11 (s, 3H; $OC(CH_3)_2$), 0.85 (s, 3H; $OC(CH_3)_2$) ppm. $^{13}C\{^{1}H\}$ NMR (75 MHz, C_6D_6, 298.0 K): δ = 160.1 (s; C=O), 140.1 (s; $C_{aryl\text{-}para}$), 138.2 (s; NC_{aryl}), 129.7 (s; $C_{aryl\text{-}meta}$), 126.1 (s; $C_{aryl\text{-}ortho}$), 124.6 (s; $O\mathit{C}(CH_3)_2$), 124.2 (s; $N\mathit{C}C(Cl)_2$), 59.7 (s; $C(\mathit{C}(Cl)_2)C$), 24.3 (s; $OC(\mathit{C}H_3)_2$), 24.7 (s; $OC(\mathit{C}H_3)_2$), 20.9 (s; $CH_{3\text{-para}}$), 18.5 (s; $CH_{3\text{-ortho}}$), 18.3 (s; $CH_{3\text{-ortho}}$) ppm [Some of the expected signals for aryl carbon atoms are not observed due to isochrony as well as overlapping with the solvent signal.]. MS (EI, 70 eV, 50 °C) *m/z* (%): 341 (2) $[M]^{+\cdot}$, 313 (9), 298 (11), 192 (42), 161 (100), 146 (16), 91 (10). Elemental analysis calcd for $C_{15}H_{17}Cl_2N_3O_2$: C 52.65, H 5.01, N 12.28, found: C 52.25, H 4.81, N 11.89.

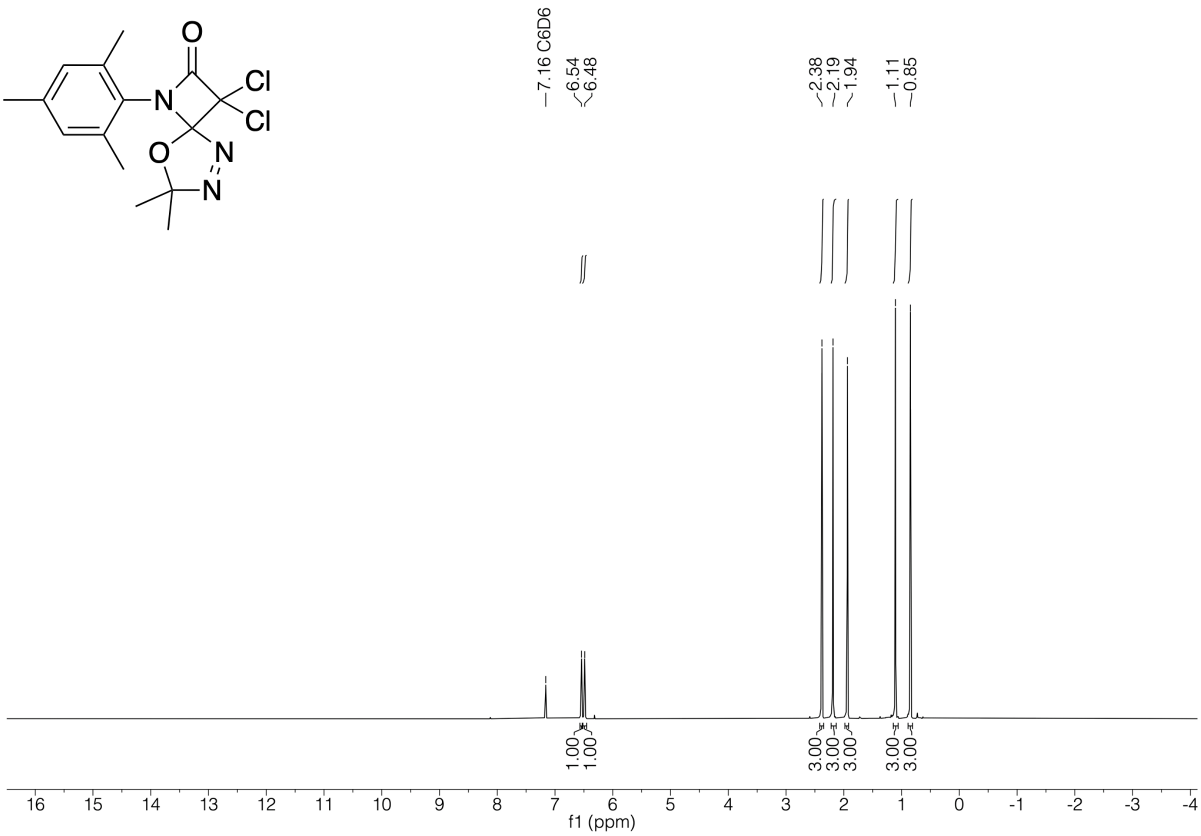

Figure SF35. ^{1}H NMR (300 MHz, C_6D_6, 298.0 K) spectrum of **Mes-5-Cl₂**.

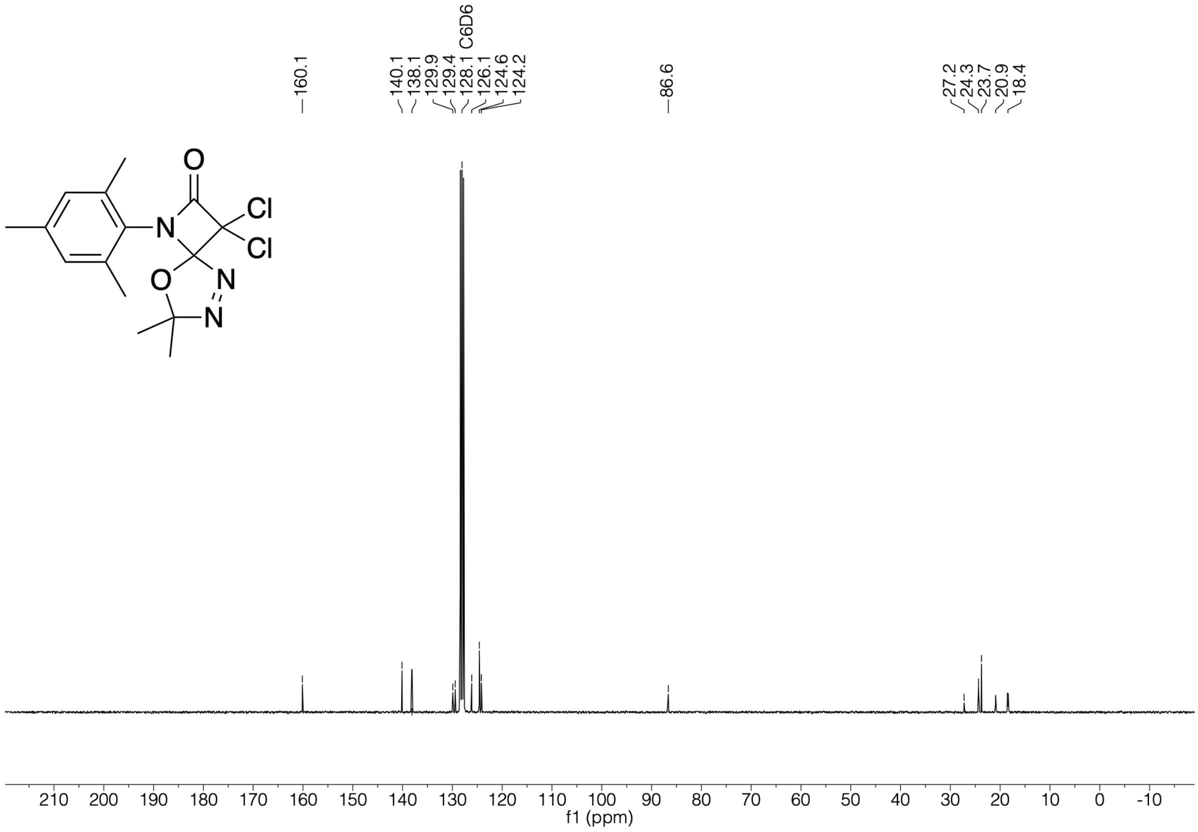

Figure SF36. $^{13}C\{^{1}H\}$ NMR (75 MHz, C_6D_6, 298.0 K) spectrum of **Mes-5-Cl$_2$**.

Mes-5-Ph$_2$: 1,3,4-oxadiazole used: **4-Mes** (4.63 g, 20 mmol), acyl chloride used: diphenylacetyl chloride (6.92 g, 30 mmol) dissolved in 20 ml of dichloromethane. Colorless, block-shaped, air-stable crystals. Yield: 7.91 g, 92%. ^{1}H NMR (300 MHz, C_6D_6, 298.0 K): δ = 7.86–7.79 (m, 4H; CH_{aryl}), 7.22–7.15 (m, 4H; CH_{aryl}), 7.06–6.99 (m, 2H; CH_{aryl}), 6.55–6.53 (m, 1H; $CH_{Mes\text{-}meta}$), 6.49–6.45 (m, 1H; $CH_{Mes\text{-}meta}$), 2.39 (s, 3H; $CH_{3\text{-}ortho}$), 2.25 (s, 3H; $CH_{3\text{-}ortho}$), 1.93 (s, 3H; $CH_{3\text{-}para}$), 1.15 (s, 3H; $OC(CH_3)_2$), 0.76 (s, 3H; $OC(CH_3)_2$) ppm. ^{13}C{^{1}H} NMR (75 MHz, C_6D_6, 298.0 K): δ = 167.6 (s; C=O), 139.2 (s; $NC_{Mes\text{-}aryl}$), 138.9 (s; C-C_{aryl}), 138.5 (s; C-C_{aryl}), 138.4 (s; C_{aryl}), 137.3 (s; C_{aryl}), 129.9 (s; C_{aryl}), 129.8 (s; C_{aryl}), 129.2 (s; C_{aryl}), 129.1 (s; C_{aryl}), 128.8 (s; C_{aryl}), 128.7 (s; C_{aryl}), 128.2 (s; N*C*C(Ph)$_2$), 127.8 (s; C_{aryl}), 127.1 (s; O*C*(CH$_3$)$_2$), 75.1 (s; C(*C*(Cl)$_2$)C), 24.5 (s; OC(*C*H$_3$)$_2$), 23.9 (s; OC(*C*H$_3$)$_2$), 20.9 (s; $CH_{3\text{-}para}$), 19.1 (s; $CH_{3\text{-}ortho}$), 19.0 (s; $CH_{3\text{-}ortho}$) ppm [Some of the expected signals for aryl carbon atoms are not observed due to isochrony as well as overlapping with the solvent signal.]. MS (EI, 70 eV, 80 °C) *m/z* (%): 425 (2) [M]$^{+\cdot}$, 397 (7), 382 (9), 311 (100), 236 (13), 194 (42), 165 (52), 91 (5). Elemental analysis calcd for $C_{27}H_{27}N_3O_2$: C 76.21, H 6.40, N 9.87, found: C 76.42, H 6.55, N 9.93.

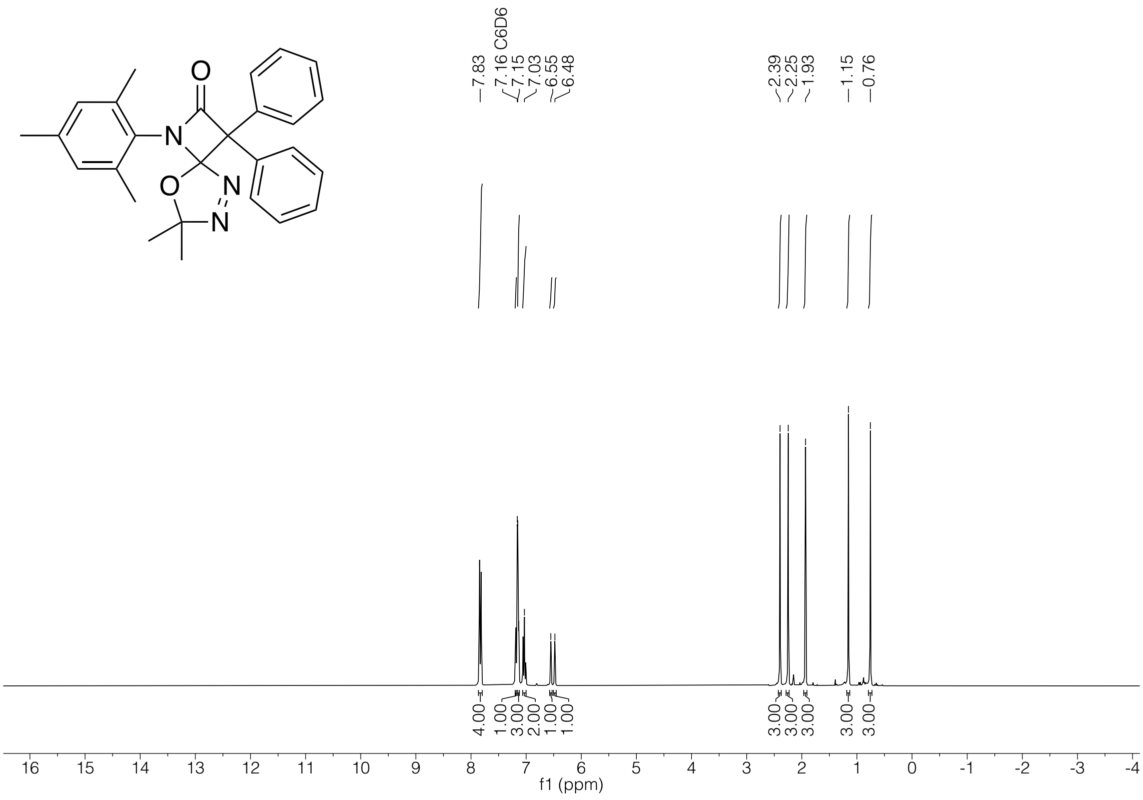

Figure SF37. ^{1}H NMR (300 MHz, C_6D_6, 298.0 K) spectrum of **Mes-5-Ph$_2$**.

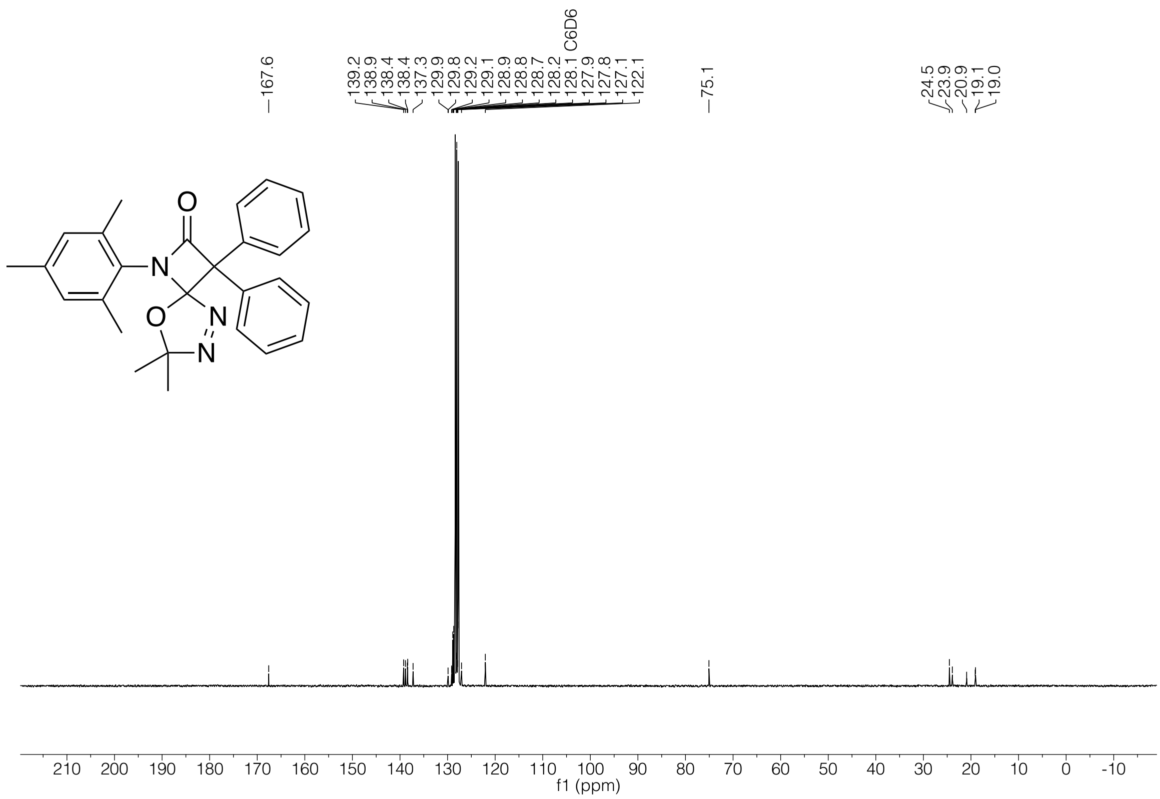

Figure SF38. $^{13}C\{^{1}H\}$ NMR (75 MHz, C_6D_6, 298.0 K) spectrum of **Mes-5-Ph₂**.

Mes-5-sCy: 1,3,4-oxadiazole used: **4-Mes** (4.63 g, 20 mmol), acyl chloride used: cyclohexanecarbonyl chloride (4.0 ml, 30 mmol). Colorless, block-shaped, air-stable crystals. Yield: 6.76 g, 99%. ^{1}H NMR (300 MHz, C_6D_6, 298.0 K): δ = 6.63–6.59 (m, 1H; CH_{meta}), 6.56–6.54 (m, 1H; CH_{meta}), 2.48 (s, 3H; $CH_{3\text{-}ortho}$), 2.46–2.39 (m, 1H; CH_{aliph}), 2.30 (s, 3H; $CH_{3\text{-}ortho}$), 2.18–2.00 (m, 2H; CH_{aliph}), 1.98 (s, 3H; $CH_{3\text{-}para}$), 1.93–1.75 (m, 3H; CH_{aliph}), 1.51–1.71 (m, 4H; CH_{aliph}), 1.14 (s, 3H; $OC(CH_3)_2$), 0.82 (s, 3H; $OC(CH_3)_2$) ppm. $^{13}C\{^{1}H\}$ NMR (75 MHz, C_6D_6, 298.0 K): δ = 171.0 (s; C=O), 138.8 (s; $C_{aryl\text{-}para}$), 138.5 (s; NC_{aryl}), 129.7 (s; $C_{aryl\text{-}meta}$), 129.1 (s; $C_{aryl\text{-}ortho}$), 127.6 (s; $O\mathit{C}(CH_3)_2$), 120.6 (s; $N\mathit{C}C_{sCy}$), 63.7 (s; $C(\mathit{C}_{sCy})C$), 29.4 (s; C_{aliph}), 28.5 (s; C_{aliph}), 25.6 (s; C_{aliph}), 25.3 (s; C_{aliph}), 24.7 (s; $OC(\mathit{C}H_3)_2$), 24.5 (s; C_{aliph}), 23.4 (s; $OC(\mathit{C}H_3)_2$), 23.3 (s; $CH_{3\text{-}para}$), 21.0 (s; $CH_{3\text{-}ortho}$), 18.8 (s; $CH_{3\text{-}ortho}$) ppm [Some of the expected signals for aryl carbon atoms are not observed due to isochrony as well as overlapping with the solvent signal.]. MS (EI, 70 eV, 50 °C) *m/z* (%): 341 (7) $[M]^{+\cdot}$, 227 (100), 212 (31), 161 (29), 110 (26), 91 (7). Elemental analysis calcd for $C_{20}H_{27}N_3O_2$: C 70.35, H 7.97, N 12.31, found: C 70.60, H 7.91, N 11.87.

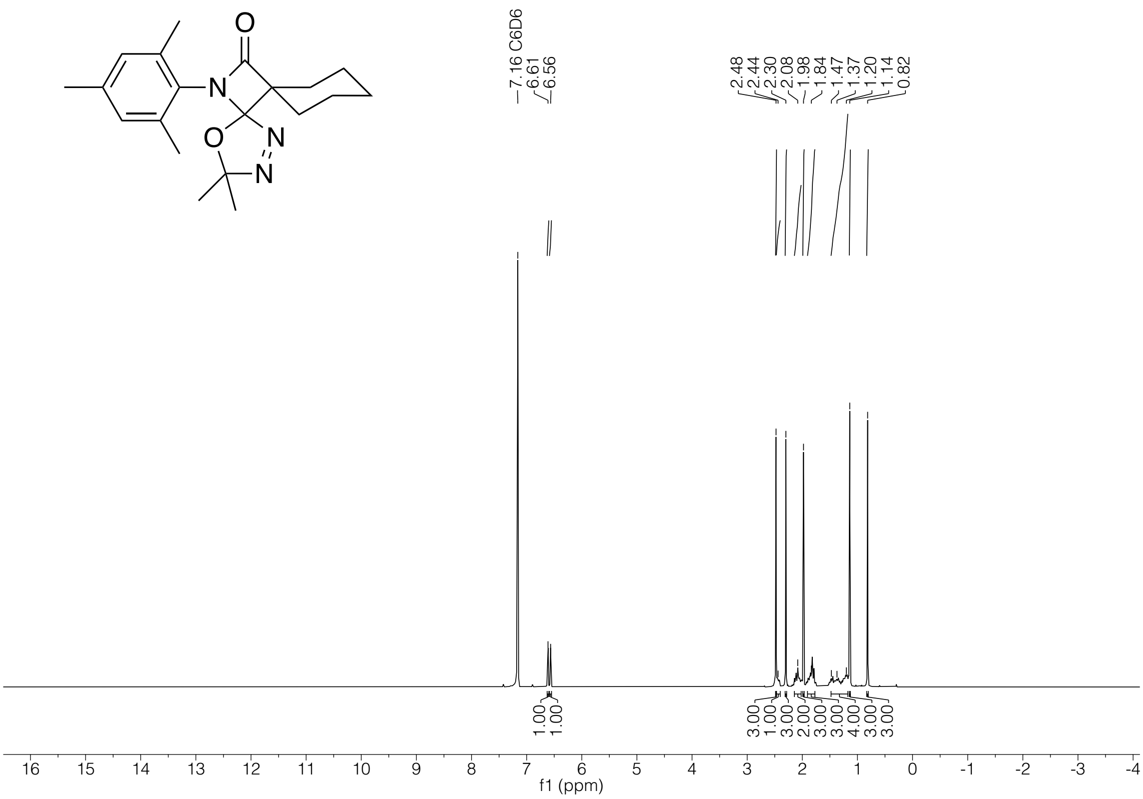

Figure SF39. ^{1}H NMR (300 MHz, C_6D_6, 298.0 K) spectrum of **Mes-5-sCy**.

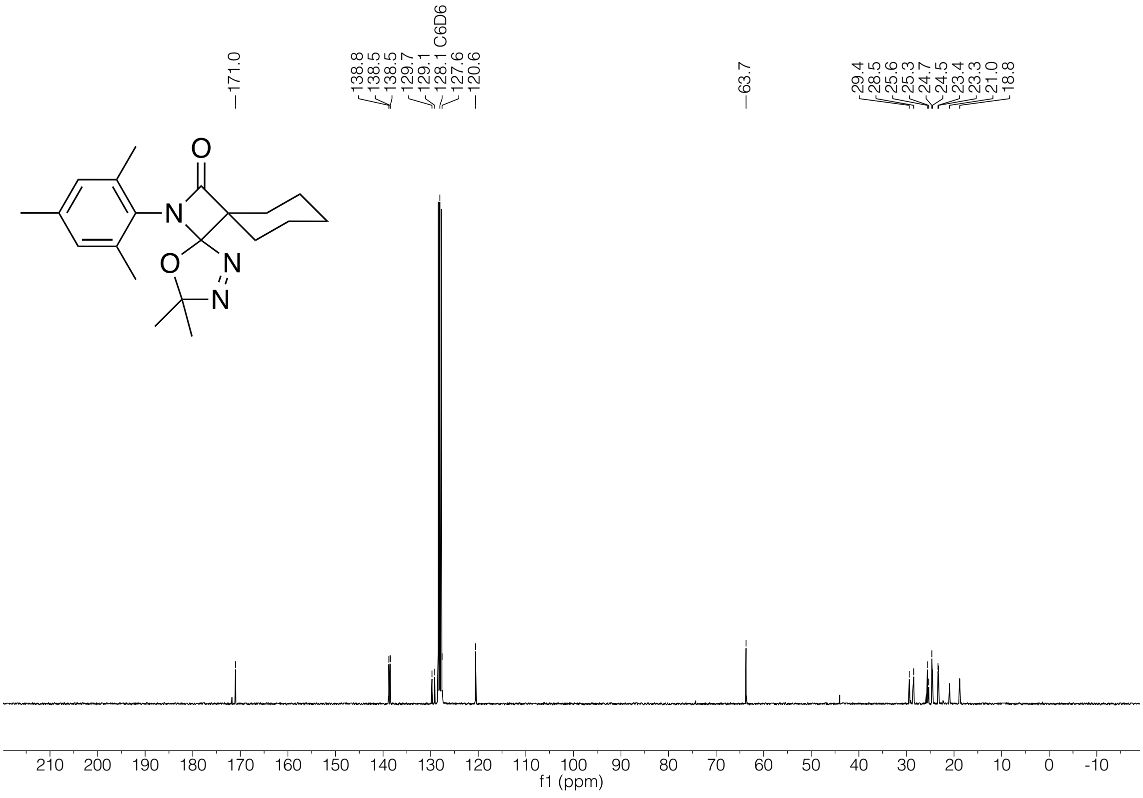

Figure SF40. $^{13}C\{^{1}H\}$ NMR (75 MHz, C_6D_6, 298.0 K) spectrum of **Mes-5-sCy**.

Dipp-5-Me$_2$: 1,3,4-oxadiazole used: **4-Dipp** (5.46 g, 20 mmol), acyl chloride used: isobutyryl chloride (3.1 ml, 30 mmol). Colorless, block-shaped, air-stable crystals. Yield: 6.73 g, 98%. ^{1}H NMR (300 MHz, C_6D_6, 298.0 K): δ = 7.14–7.10 (m, 1H; $CH_{aryl\text{-}para}$), 7.03–6.99 (m, 2H; $CH_{aryl\text{-}meta}$), 3.91 (sept, J = 7 Hz, 1H; CH_{iPr}), 3.52 (sept, J = 7 Hz, 1H; CH_{iPr}), 1.50 (s, 3H; $C(C(CH_3)_2)C$), 1.30 (d, J = 7 Hz, 3H; $CH_{3\text{-}iPr}$), 1.29 (s, 3H; $C(C(CH_3)_2)C$), 1.24 (d, J = 7 Hz, 3H; $CH_{3\text{-}iPr}$), 1.23 (d, J = 7 Hz, 3H; $CH_{3\text{-}iPr}$), 1.18 (d, J = 7 Hz, 3H; $CH_{3\text{-}iPr}$), 1.05 (s, 3H; $OC(CH_3)_2$), 0.79 (s, 3H; $OC(CH_3)_2$) ppm. $^{13}C\{^1H\}$ NMR (75 MHz, C_6D_6, 298.0 K): δ = 172.1 (s; C=O), 149.6 (s; NC_{aryl}), 149.5 (s; NCC$(CH_3)_2$), 130.2 (s; $C_{aryl\text{-}para}$), 127.3 (s; $C_{aryl\text{-}ortho}$), 126.8 (s; $C_{aryl\text{-}ortho}$), 124.5 (s; $C_{aryl\text{-}meta}$), 123.6 (s; $C_{aryl\text{-}meta}$), 120.6 (s; O$C$$(CH_3)_2$), 59.9 (s; C($C$$(CH_3)_2$)C), 29.1 (s; CH_{iPr}), 29.0 (s; CH_{iPr}), 25.6 (s; $CH_{3\text{-}iPr}$), 25.4 (s; $CH_{3\text{-}iPr}$), 24.8 (s; OC($C$$H_3)_2$), 24.7 (s; OC($C$$H_3)_2$), 23.6 (s; $CH_{3\text{-}iPr}$), 22.5 (s; $CH_{3\text{-}iPr}$), 19.0 (s; C(C($C$$H_3)_2$)C), 17.8 (s; C(C($C$$H_3)_2$)C) ppm. MS (EI, 70 eV, 60 °C) *m/z* (%): 343 (3) $[M]^{+\cdot}$, 272 (36), 244 (100), 214 (30), 203 (44), 186 (32), 173 (61), 146 (13), 91 (6). Elemental analysis calcd for $C_{20}H_{29}N_3O_2$: C 69.94, H 8.51, N 12.23, found: C 70.09, H 8.46, N 11.89.

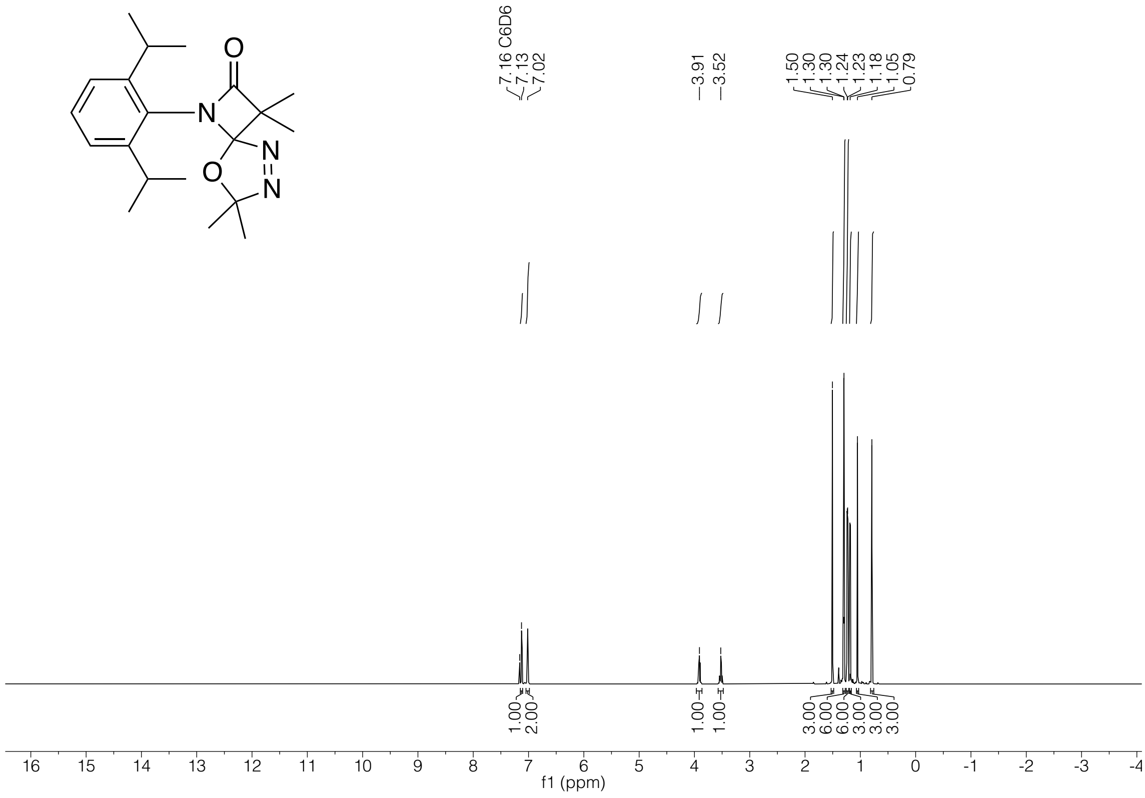

Figure SF41. ^{1}H NMR (300 MHz, C_6D_6, 298.0 K) spectrum of **Dipp-5-Me$_2$**.

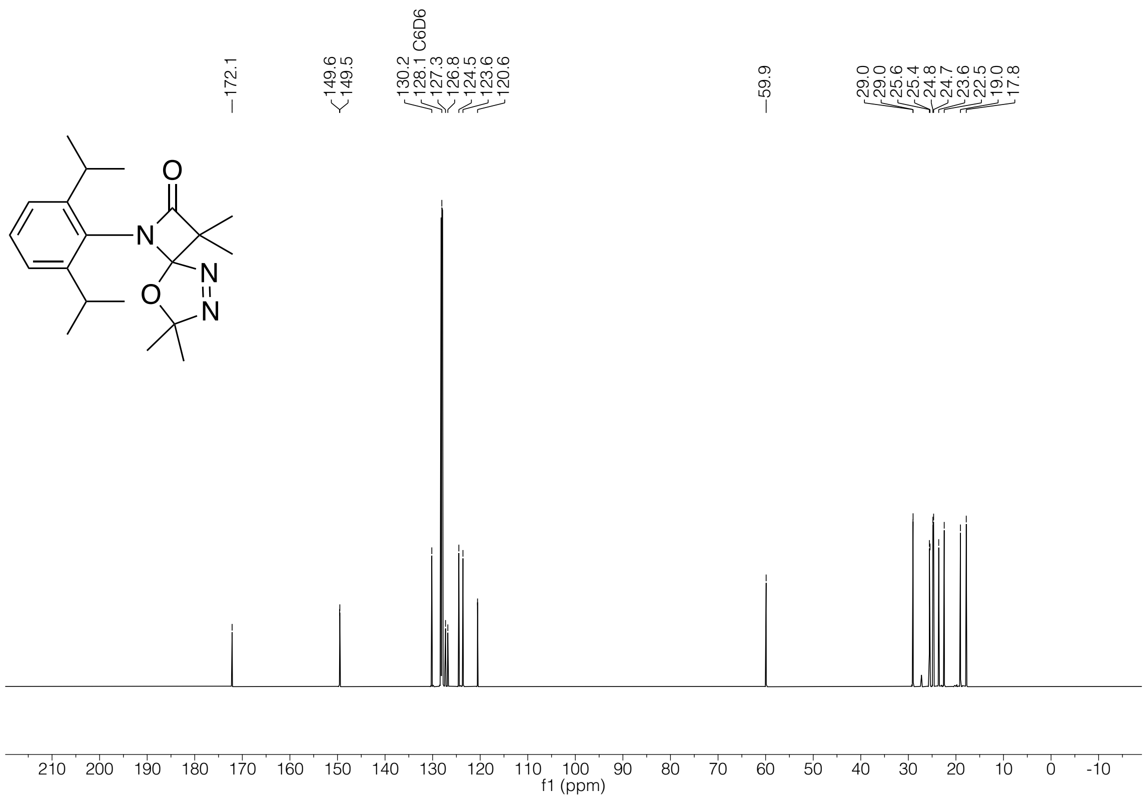

Figure SF42. $^{13}C\{^{1}H\}$ NMR (75 MHz, C_6D_6, 298.0 K) spectrum of **Dipp-5-Me₂**.

Dipp-5-Cl_2: 1,3,4-oxadiazole used: **4-Dipp** (5.46 g, 20 mmol), acyl chloride used: dichloroacetyl chloride (2.9 ml, 30 mmol). Colorless, block-shaped, air-stable crystals. Yield: 6.46 g, 94%. ^{1}H NMR (300 MHz, C_6D_6, 298.0 K): δ = 7.10–7.07 (m, 1H; $CH_{aryl\text{-}para}$), 6.98–9.94 (m, 2H; $CH_{aryl\text{-}meta}$), 3.85 (sept, J = 7 Hz, 1H; CH_{iPr}), 3.42 (sept, J = 7 Hz, 1H; CH_{iPr}), 1.23 (d, J = 7 Hz, 3H; $CH_{3\text{-}iPr}$), 1.18 (d, J = 7 Hz, 3H; $CH_{3\text{-}iPr}$), 1.17 (d, J = 7 Hz, 3H; $CH_{3\text{-}iPr}$), 1.13 (d, J = 7 Hz, 3H; $CH_{3\text{-}iPr}$), 1.08 (s, 3H; $OC(CH_3)_2$), 0.79 (s, 3H; $OC(CH_3)_2$) ppm. $^{13}C\{^{1}H\}$ NMR (75 MHz, C_6D_6, 298.0 K): δ = 161.2 (s; C=O), 149.5 (s; NC_{aryl}), 149.4 (s; N*C*C(Cl)₂), 131.2 (s; $C_{aryl\text{-}para}$), 125.2 (s; $C_{aryl\text{-}ortho}$), 124.8 (s; $C_{aryl\text{-}ortho}$), 124.6 (s; $C_{aryl\text{-}meta}$), 124.1 (s; $C_{aryl\text{-}meta}$), 123.6 (s; O*C*$(CH_3)_2$), 93.9 (s; C(*C*(Cl)₂)C), 29.2 (s; CH_{iPr}), 29.0 (s; CH_{iPr}), 25.4 (s; $CH_{3\text{-}iPr}$), 25.3 (s; $CH_{3\text{-}iPr}$), 24.4 (s; OC(*C*H_3)₂), 24.0 (s; OC(*C*H_3)₂), 23.5 (s; $CH_{3\text{-}iPr}$), 22.7 (s; $CH_{3\text{-}iPr}$) ppm. MS (EI, 70 eV, 70 °C) *m/z* (%): 383 (4) $[M]^{+\cdot}$, 312 (69), 298 (62), 234 (30), 203 (100), 188 (76), 146 (26), 91 (12). Elemental analysis calcd for $C_{18}H_{22}Cl_2N_3O_2$: C 56.26, H 6.03, N 10.93, found: C 56.33, H 6.06, N 10.44.

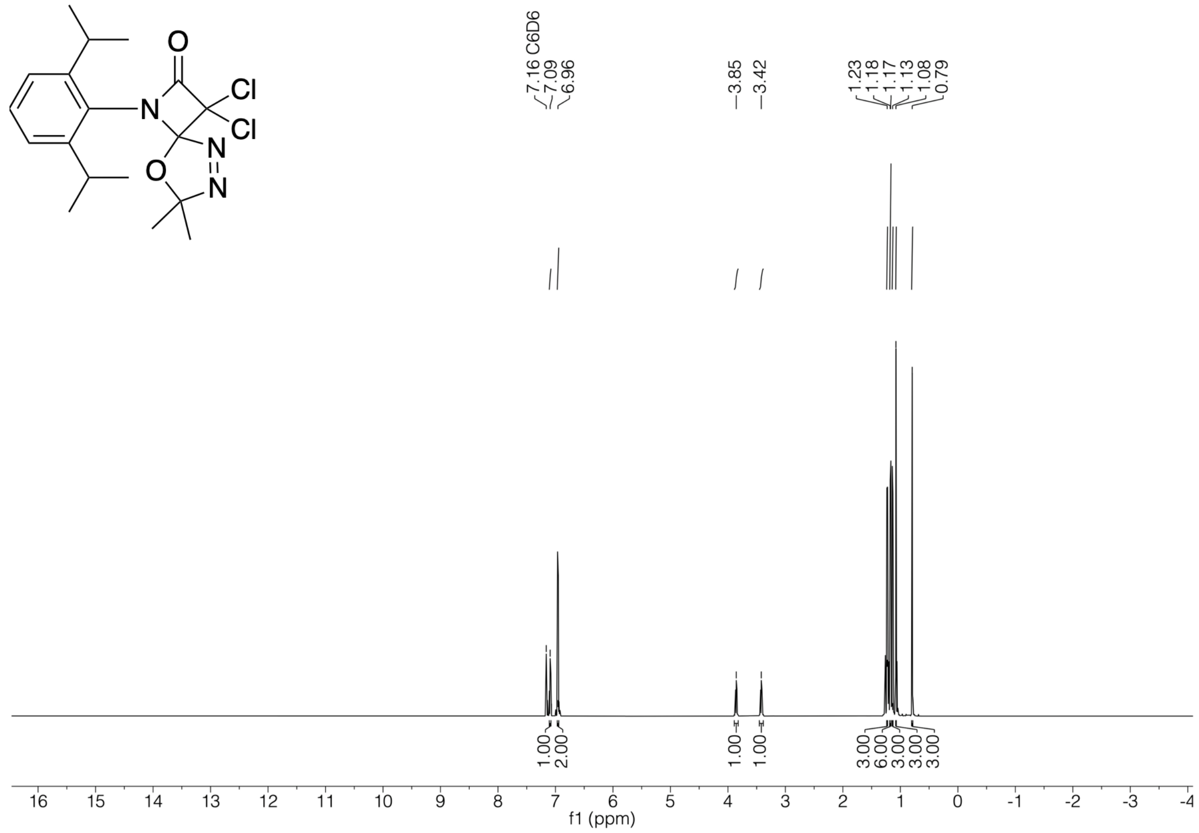

Figure SF43. ^{1}H NMR (300 MHz, C_6D_6, 298.0 K) spectrum of **Dipp-5-Cl$_2$**.

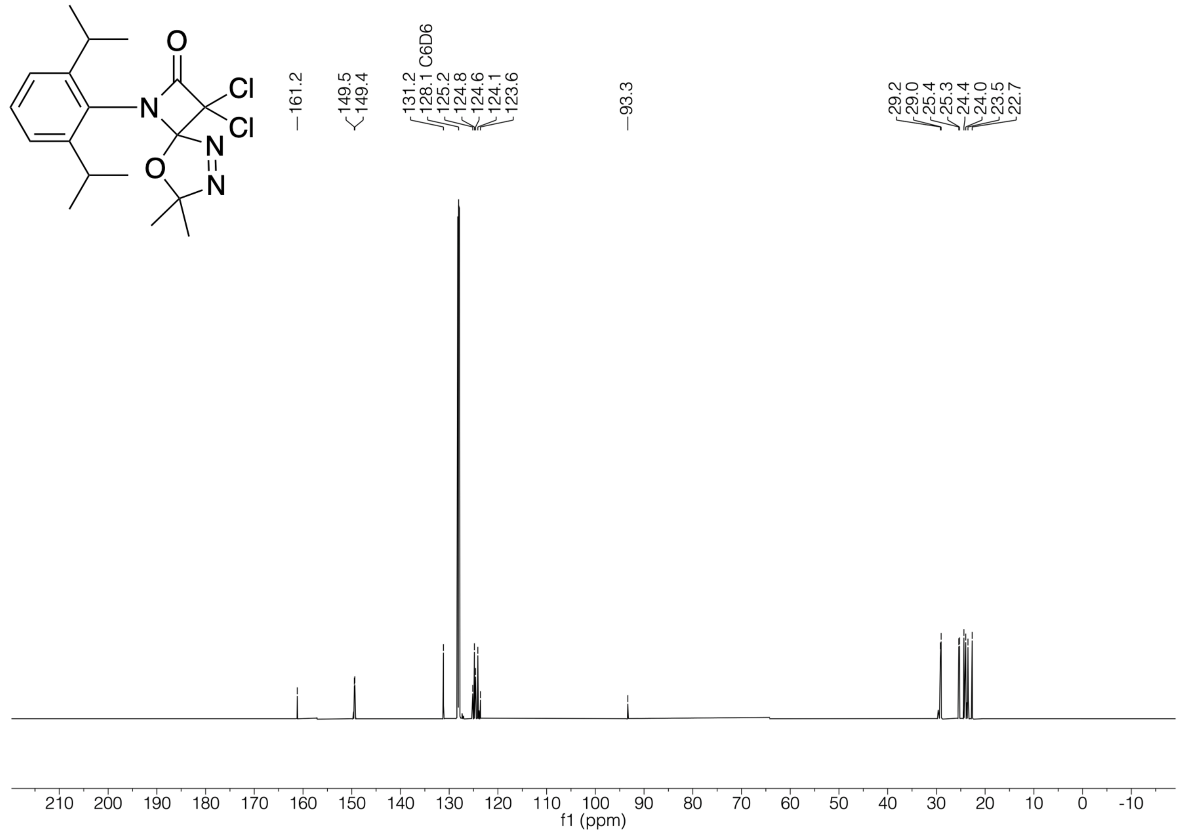

Figure SF44. $^{13}C\{^1H\}$ NMR (75 MHz, C_6D_6, 298.0 K) spectrum of **Dipp-5-Cl$_2$**.

Dipp-5-Ph$_2$: 1,3,4-oxadiazole used: **4-Dipp** (5.46 g, 20 mmol), acyl chloride used: diphenylacetyl chloride (6.92 g, 30 mmol) dissolved in 20 ml of dichloromethane. Colorless, block-shaped, air-stable crystals. Yield: 8.51 g, 91%. ^{1}H NMR (300 MHz, C_6D_6, 298.0 K): δ = 7.89–7.77 (m, 4H; CH_{aryl}), 7.19–6.95 (m, 9H; CH_{aryl}), 3.81 (sept, J = 7 Hz, 1H; CH_{iPr}), 3.47 (sept, J = 7 Hz, 1H; CH_{iPr}), 1.29 (d, J = 7 Hz, 3H; $CH_{3\text{-}iPr}$), 1.28 (d, J = 7 Hz, 3H; $CH_{3\text{-}iPr}$), 1.18 (s, 3H; $OC(CH_3)_2$), 1.09 (d, J = 7 Hz, 3H; $CH_{3\text{-}iPr}$), 1.03 (d, J = 7 Hz, 3H; $CH_{3\text{-}iPr}$), 0.70 (s, 3H; $OC(CH_3)_2$) ppm. ^{13}C{^{1}H} NMR (75 MHz, C_6D_6, 298.0 K): δ = 168.7 (s; C=O), 150.0 (s; NC_{aryl}), 149.6 (s; N*C*C(Ph)$_2$), 138.4 (s; C_{aryl}), 137.4 (s; C_{aryl}), 130.5 (s; C_{aryl}), 128.9 (s; C_{aryl}), 128.6 (s; C_{aryl}), 127.9 (s; C_{aryl}), 127.4 (s; C_{aryl}), 126.4 (s; C_{aryl}), 124.8 (s; C_{aryl}), 123.6 (s; C_{aryl}), 122.2 (s; O*C*(CH$_3$)$_2$), 74.9 (s; C(*C*(Ph)$_2$)C), 29.1 (s; CH_{iPr}), 28.9 (s; CH_{iPr}), 26.1 (s; $CH_{3\text{-}iPr}$), 25.5 (s; $CH_{3\text{-}iPr}$), 24.9 (s; OC(*C*H$_3$)$_2$), 23.9 (s; OC(*C*H$_3$)$_2$), 23.4 (s; $CH_{3\text{-}iPr}$), 21.9 (s; $CH_{3\text{-}iPr}$) ppm [Some of the expected signals for aryl carbon atoms are not observed due to isochrony as well as overlapping with the solvent signal.]. MS (EI, 70 eV, 60 °C) *m/z* (%): 467 (2) $[M]^{+\cdot}$, 396 (20), 386 (52), 236 (25), 194 (100), 165 (72), 146 (5), 91 (6). Elemental analysis calcd for $C_{30}H_{33}N_3O_2$: C 77.06, H 7.11, N 8.99, found: C 76.87, H 7.31, N 9.05.

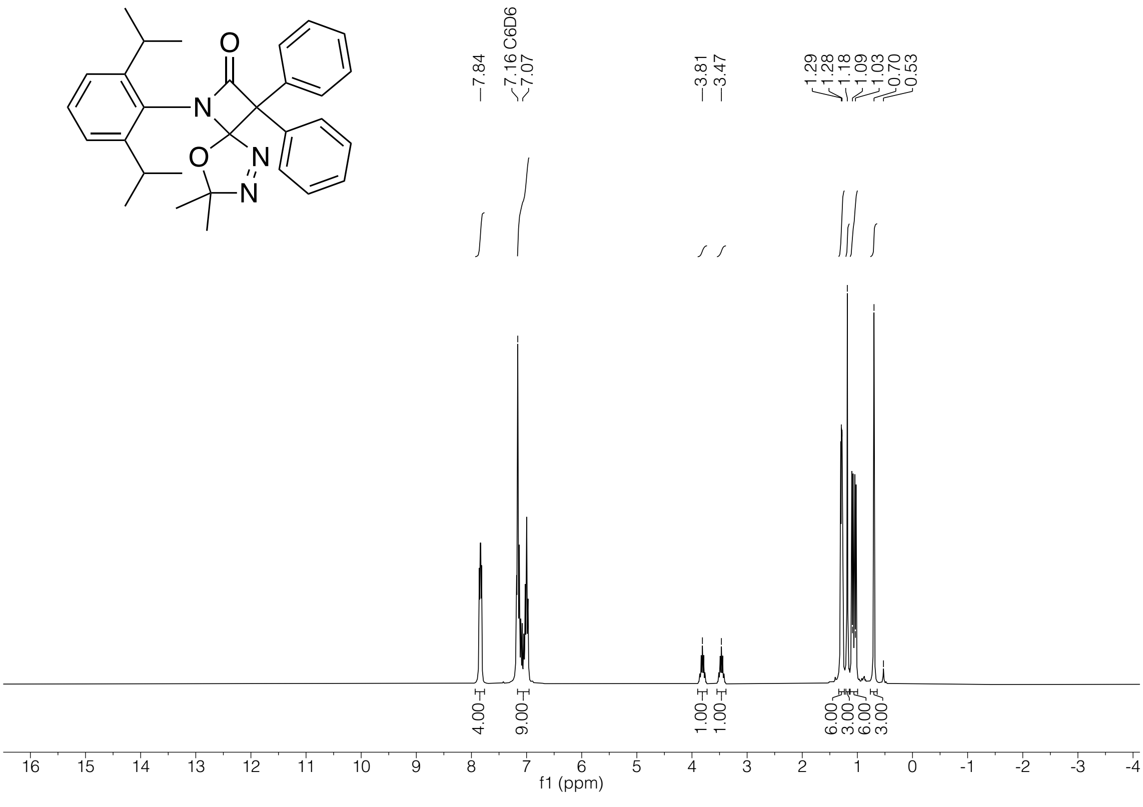

Figure SF45. ^{1}H NMR (300 MHz, C_6D_6, 298.0 K) spectrum of **Dipp-5-Ph$_2$**.

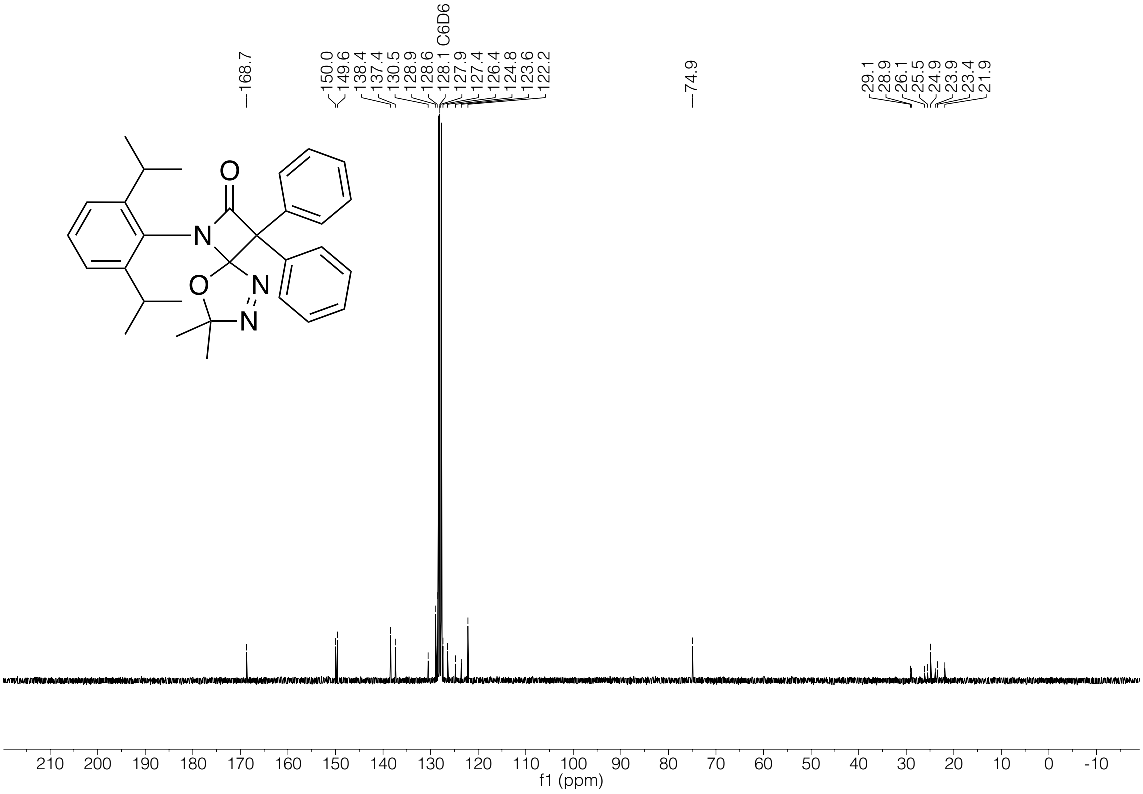

Figure SF46. $^{13}C\{^1H\}$ NMR (75 MHz, C_6D_6, 298.0 K) spectrum of **Dipp-5-Ph$_2$**.

Dipp-5-sCy: 1,3,4-oxadiazole used: **4-Dipp** (5.46 g, 20 mmol), acyl chloride used: cyclohexanecarbonyl chloride (4.0 ml, 30 mmol). Colorless, block-shaped, air-stable crystals. Yield: 7.59 g, 99%. ^{1}H NMR (300 MHz, C_6D_6, 298.0 K): δ = 7.15–7.09 (m, 1H; $CH_{aryl\text{-}para}$), 7.04–6.98 (m, 2H; $CH_{aryl\text{-}meta}$), 3.93 (sept, J = 7 Hz, 1H; CH_{iPr}), 3.57 (sept, J = 7 Hz, 1H; CH_{iPr}), 2.48–2.38 (m, 1H; CH_{aliph}), 2.18–2.05 (m, 2H; CH_{aliph}), 1.91–1.71 (m, 4H; CH_{aliph}), 1.55–1.36 (m, 2H; CH_{aliph}), 1.30 (d, J = 7 Hz, 3H; $CH_{3\text{-}iPr}$), 1.27 (d, J = 7 Hz, 3H; $CH_{3\text{-}iPr}$), 1.25 (d, J = 7 Hz, 3H; $CH_{3\text{-}iPr}$), 1.19 (d, J = 7 Hz, 3H; $CH_{3\text{-}iPr}$), 1.15 (s, 3H; $OC(CH_3)_2$), 1.03–0.86 (m, 1H; CH_{aliph}), 0.75 (s, 3H; $OC(CH_3)_2$) ppm. $^{13}C\{^1H\}$ NMR (75 MHz, C_6D_6, 298.0 K): δ = 172.0 (s; C=O), 149.7 (s; NC_{aryl}), 145.5 (s; NCC_{sCy}), 130.1 (s; $C_{aryl\text{-}para}$), 127.4 (s; $C_{aryl\text{-}ortho}$), 127.0 (s; $C_{aryl\text{-}ortho}$), 124.5 (s; $C_{aryl\text{-}meta}$), 123.6 (s; $C_{aryl\text{-}meta}$), 120.6 (s; $OC(CH_3)_2$), 63.0 (s; $C(C_{sCy})C$), 29.3 (s; CH_{iPr}), 29.1 (s; CH_{iPr}), 29.0 (s; C_{aliph}), 28.5 (s; C_{aliph}), 25.7 (s; $CH_{3\text{-}iPr}$), 25.5 (s; $CH_{3\text{-}iPr}$), 25.0 (s; $OC(CH_3)_2$), 24.5 (s; C_{aliph}), 24.4 (s; C_{aliph}), 23.6 (s; $OC(CH_3)_2$), 23.3 (s; $CH_{3\text{-}iPr}$), 23.2 (s; C_{aliph}), 22.4 (s; $CH_{3\text{-}iPr}$) ppm. MS (EI, 70 eV, 50 °C) m/z (%): 383 (10) $[M]^{+\cdot}$, 286 (10), 272 (37), 244 (25), 146 88), 91 (100). Elemental analysis calcd for $C_{23}H_{33}N_3O_2$: C 72.03, H 8.67, N 10.96, found: C 72.20, H 8.62, N 11.09.

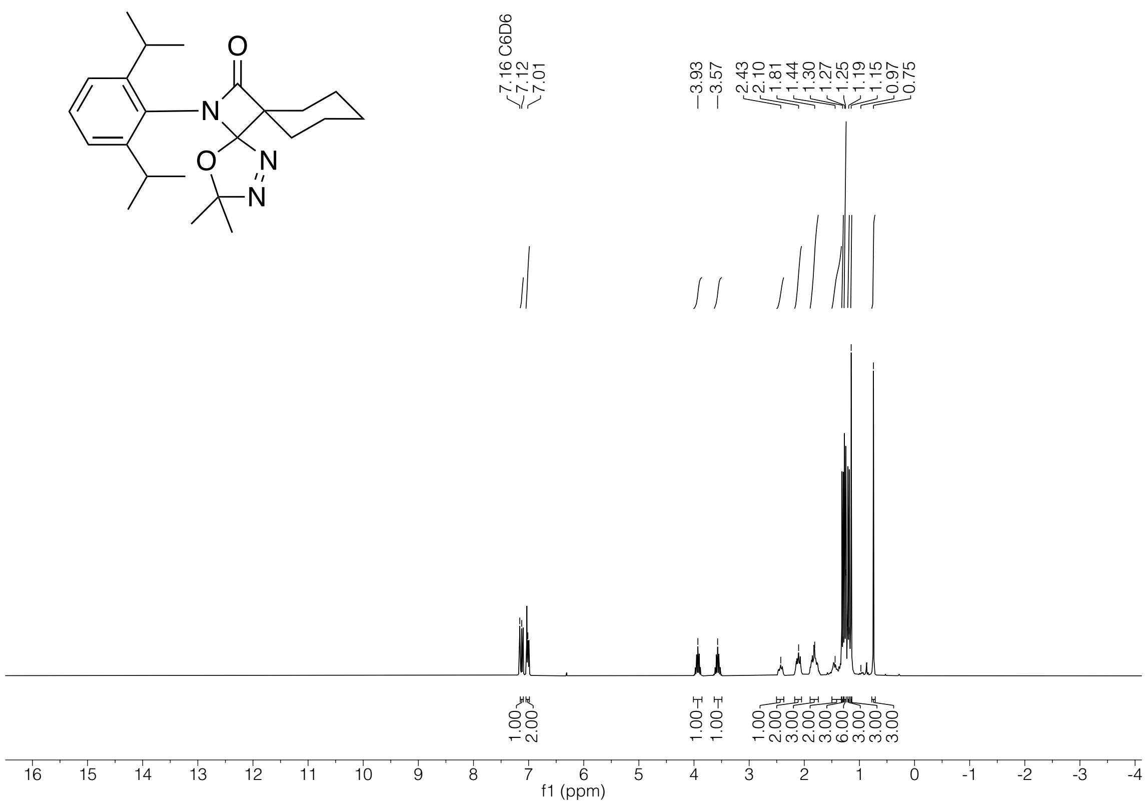

Figure SF47. ^{1}H NMR (300 MHz, C_6D_6, 298.0 K) spectrum of **Dipp-5-sCy**.

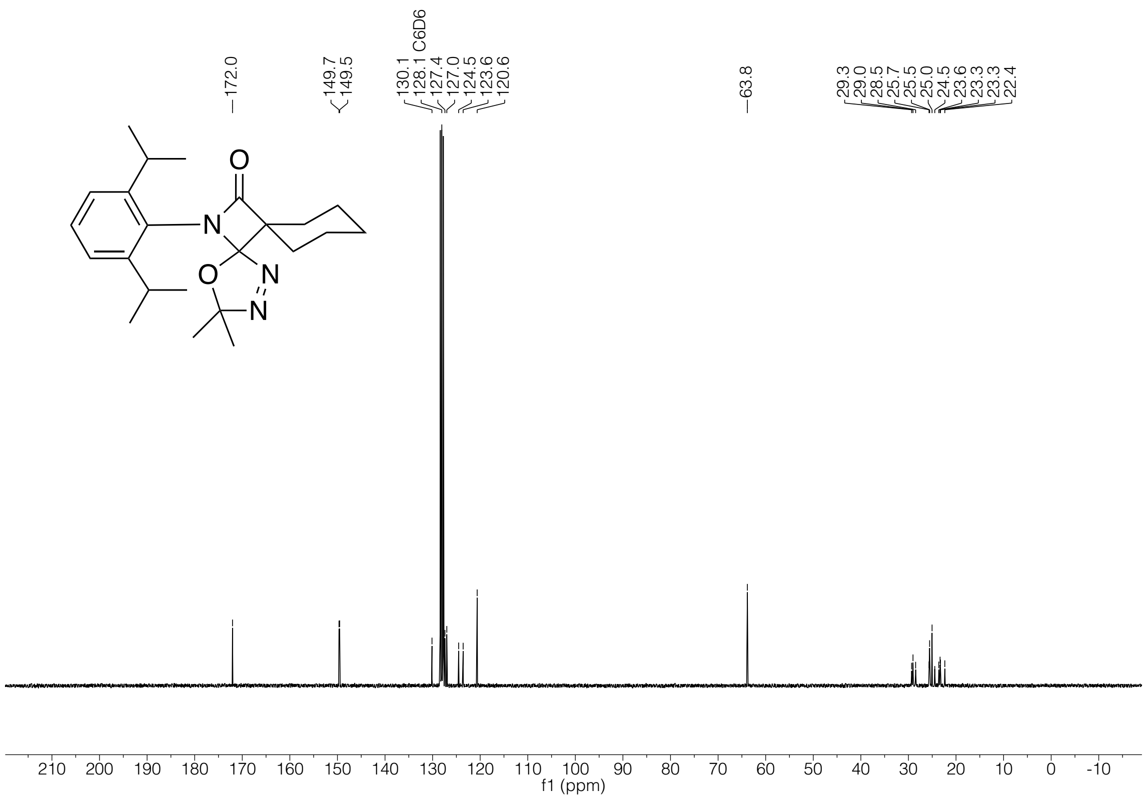

Figure SF48. $^{13}C\{^{1}H\}$ NMR (75 MHz, C_6D_6, 298.0 K) spectrum of **Dipp-5-sCy**.

f. Thermolysis of precursors 5

The corresponding precursor **5** was heated to 110 °C for 16 h in toluene (200 mg precursor, 1.65 M) and all thermolysis products were separated by column chromatography (diethyl ether/cyclohexane 1:5, silica). Table 1 summarizes all the thermolysis products obtained from a precursor and the corresponding yields. All compounds described in Table 1, with the exception of the ketenimines **R_1-10-R^2_2** (colorless oils), are colorless, air-stable powders. The yields of the ketazines did not differ when the thermolysis was carried out in an argon atmosphere. Only when R^2 is an aromatic substituent, the corresponding ketenimine can be easily isolated and is less susceptible to hydrolysis. The ketenimines **Mes-10-Ph_2** and **Dipp-10-Ph_2** are not hydrolyzed by air humidity.

Table ST1. Thermolysis of precursors **5** and yields of resulting thermolysis products.

Precursor **5** 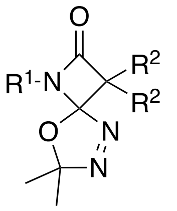	Dimer **[R^1-6-R^2_2]$_2$** 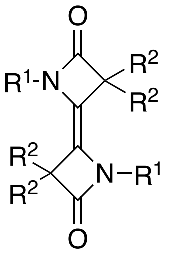	Ketazine **[R^1-6-R^2_2*N]$_2$** 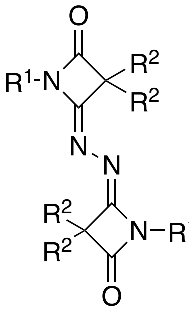	Amide **R^1-7-R^2_2** 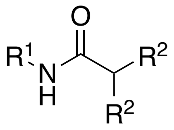 or Ketenimine **R^1-10-R^2_2** 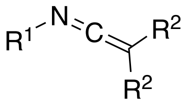
R^1 = Ph<br>R^2 = Me	Yield: 56 mg, 42%.	Yield: 72 mg, 50%.	Yield: 8 mg, 6%.<br>**Ph-7-Me_2**
R^1 = Ph<br>R^2 = Cl	Yield: 80 mg, 56%.	Yield: 56 mg, 37%.	Yield: 10 mg, 7%.<br>**Ph-7-Cl_2**
R^1 = Ph<br>R^2 = Ph	Yield: 6 mg, 4%.	Yield: 10 mg, 6%.	Yield: 123 mg, 82%.<br>**Ph-7-Ph_2** (after hydrolysis)<br>Yield: 129 mg 92%<br>**Ph-10-Ph_2**
R^1 = Ph<br>R^2 = sCy	not obtained	not obtained	Yield: 125 mg, 92%.<br>**Ph-7-sCy**
R^1 = Mes<br>R^2 = Me	Yield: 106 mg, 72%.	Yield: 3 mg, 2%.	Yield: 15 mg, 11%.<br>**Mes-7-Me_2**
R^1 = Mes<br>R^2 = Ph	not obtained	not obtained	Yield: 138 mg, 94%.<br>**Mes-10-Ph_2**

R^1 = Dipp<br>R^2 = Me	Yield: 150 mg, 85%.	Yield: 4 mg, 3%.	Yield: 7 mg, 5%.<br>**Dipp-7-Me₂**
R^1 = Dipp<br>R^2 = Ph	not obtained	not obtained	Yield: 144 mg, 91%.<br>**Dipp-10-Ph₂**
R^1 = Dipp<br>R^2 = sCy	not obtained	not obtained	Yield: 142 mg, 95%<br>**Dipp-7-sCy**

[Ph-6-Me$_2$]$_2$: ^{1}H NMR (300 MHz, C_6D_6, 298.0 K): δ = 8.33–8.29 (m, 2H; CH_{aryl}), 7.06–7.00 (m, 2H; CH_{aryl}), 6.90–6.88 (m, 1H; CH_{aryl}), 1.01 (s, 6H; CH_3) ppm [Integrals are given for monomeric unit.]. $^{13}C\{^1H\}$ NMR (75 MHz, C_6D_6, 298.0 K): δ = 174.4 (s; C=O), 136.0 (s; NC_{aryl}), 129.7 (s; C=C), 129.2 (s; C_{aryl}), 127.5 (s; C_{aryl}), 120.2 (s; C_{aryl}), 67.6 (s; C(*C*(Me)$_2$)C), 19.0 (s; CH_3) ppm. MS (ESI) *m/z* (%): 347.19 [M-H]$^+$. Elemental analysis calcd for $C_{22}H_{22}N_2O_2$: C 76.28, H 6.40, N 8.09, found: C 75.98, H 6.58, N 8.14.

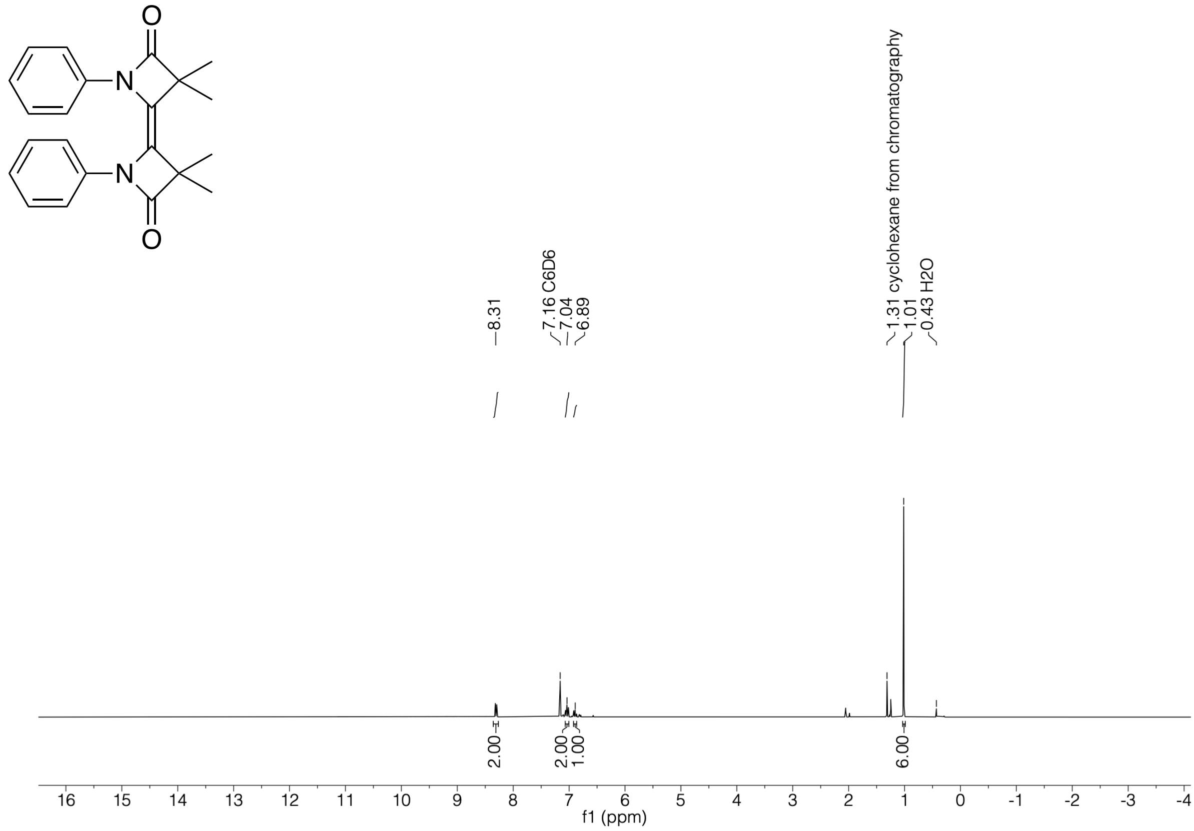

Figure SF49. ^{1}H NMR (300 MHz, C_6D_6, 298.0 K) spectrum of **[Ph-6-Me$_2$]$_2$**.

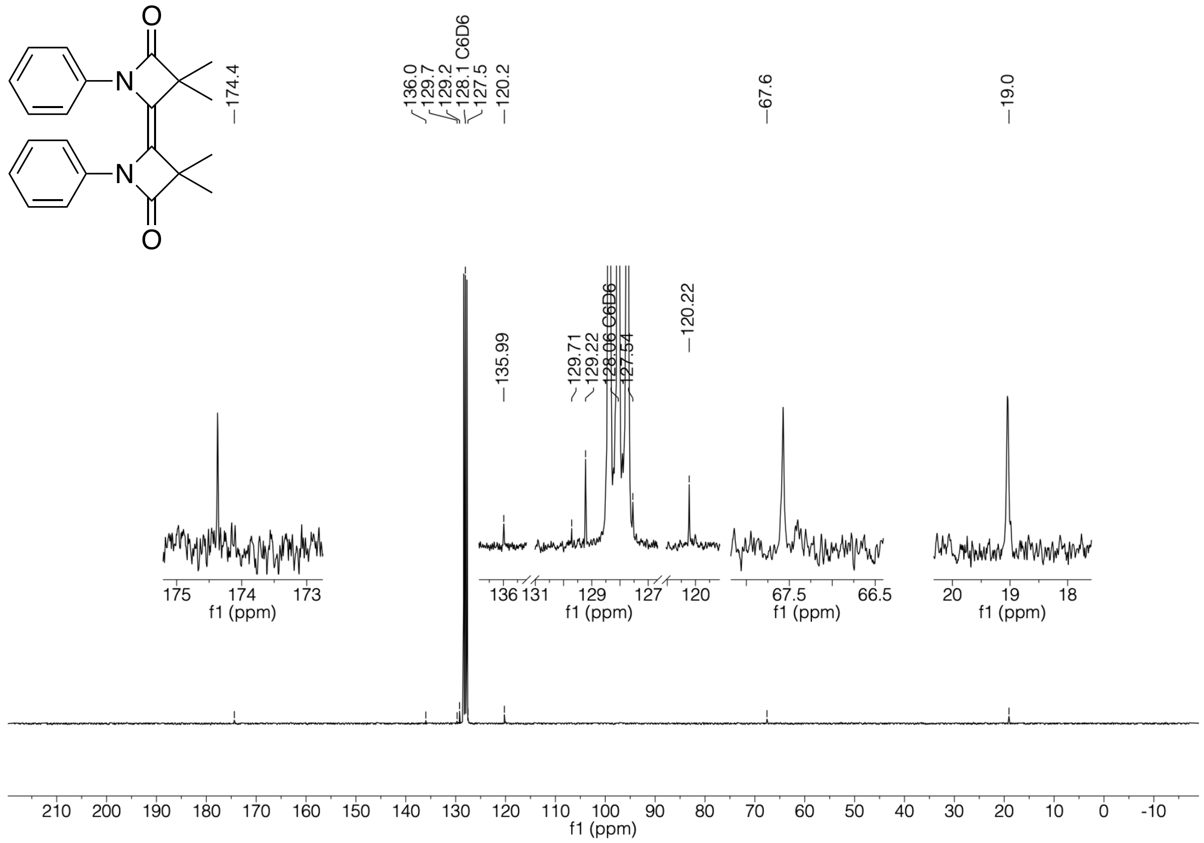

Figure SF50. $^{13}C\{^1H\}$ NMR (75 MHz, C_6D_6, 298.0 K) spectrum of **[Ph-6-Me$_2$]$_2$**.

[Ph-6-Me$_2$*N]$_2$: ^1H NMR (300 MHz, $CDCl_3$, 298.0 K): δ = 8.04–7.98 (m, 2H; CH_{aryl}), 7.46–7.41 (m, 2H; CH_{aryl}), 7.24–7.21 (m, 1H; CH_{aryl}), 1.68 (s, 6H; CH_3) ppm [Integrals are given for monomeric unit.]. $^{13}C\{^1H\}$ NMR (75 MHz, $CDCl_3$, 298.0 K): δ = 174.4 (s; C=O), 162.9 (s; C=N), 136.2 (s; NC_{aryl}), 129.2 (s; C_{aryl}), 125.7 (s; C_{aryl}), 119.1 (s; C_{aryl}), 59.9 (s; C(*C*(Me)$_2$)C), 19.3 (s; CH_3) ppm. MS (ESI) *m/z* (%): 375.19 [M-H]$^+$. Elemental analysis calcd for $C_{22}H_{22}N_4O_2$: C 70.57, H 5.92, N 14.96, found: C 70.81, H 6.14, N 15.44.

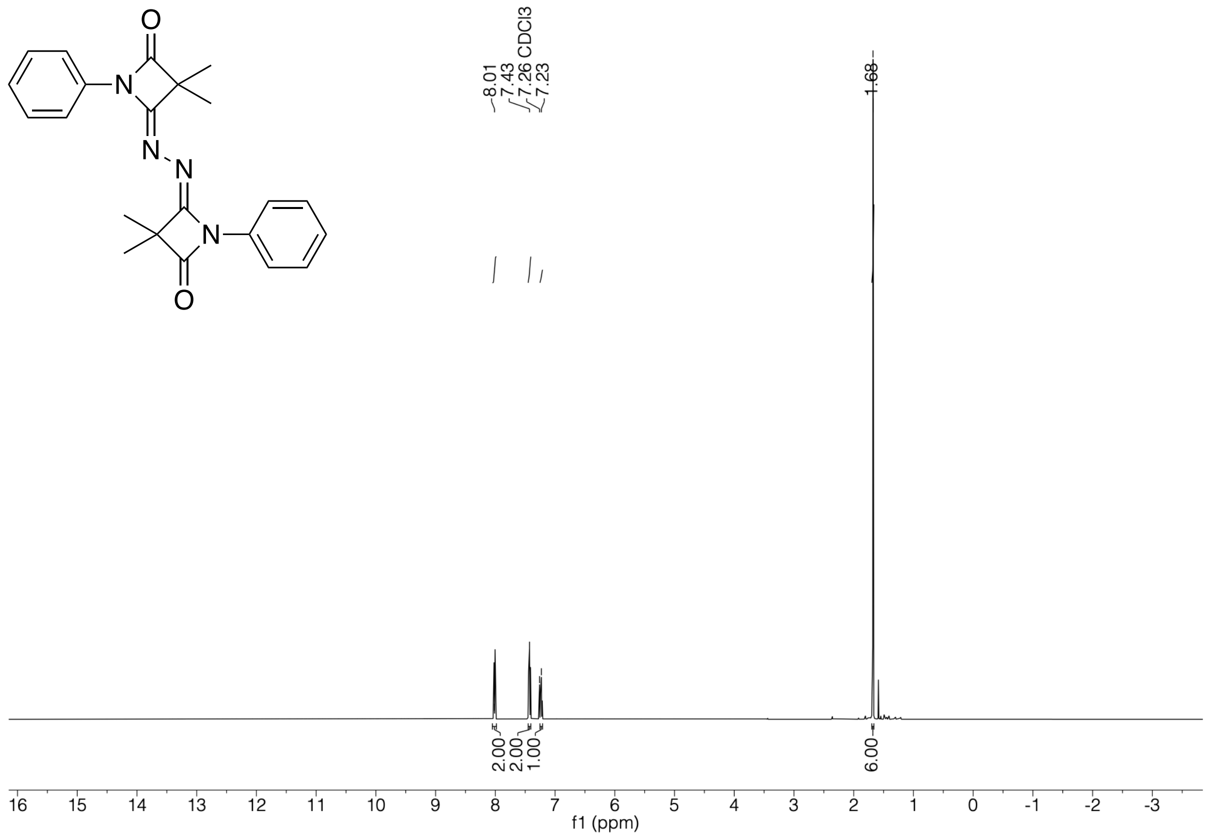

Figure SF51. ^{1}H NMR (300 MHz, $CDCl_3$, 298.0 K) spectrum of **[Ph-6-Me$_2$*N]$_2$**.

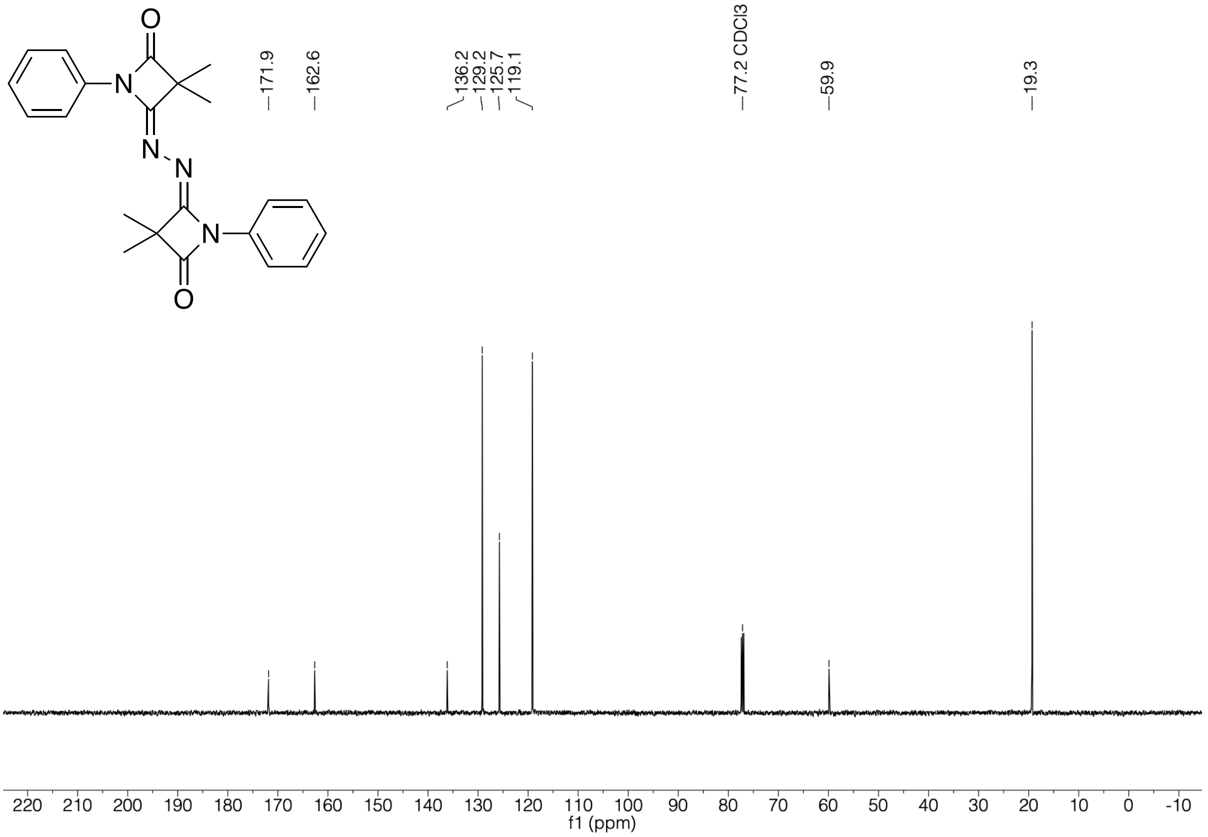

Figure SF52. $^{13}C\{^{1}H\}$ NMR (75 MHz, $CDCl_3$, 298.0 K) spectrum of **[Ph-6-Me$_2$*N]$_2$**.

Ph-7-Me₂: ^{1}H NMR (300 MHz, $CDCl_3$, 298.0 K): δ = 7.57–7.51 (m, 2H; CH_{aryl}), 7.34–7.29 (m, 2H; CH_{aryl}), 6.97 (s, 1H; NH), 6.99–6.96 (m, 1H; CH_{aryl}), 2.51 (sept, J = 7 Hz, 1H; CH), 1.25 (d, J = 7 Hz, 6H; CH_3) ppm. No $^{13}C\{^1H\}$ NMR was recorded due to low amount of sample. MS (ESI) m/z (%): 164.11 $[M-H]^+$. Elemental analysis calcd for $C_{10}H_{13}NO$: C 73.59, H 8.03, N 8.58, found: C 73.68, H 8.12, N 8.55.

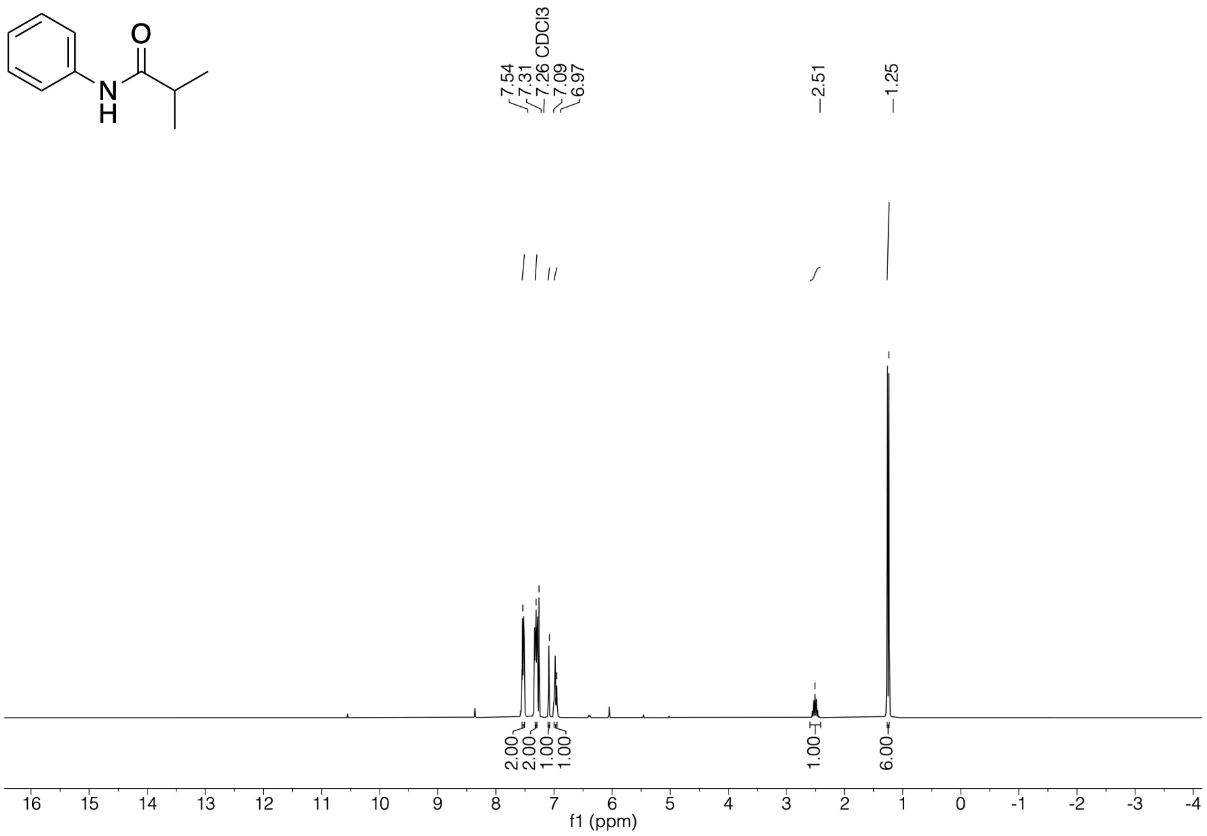

Figure SF53. ^{1}H NMR (300 MHz, $CDCl_3$, 298.0 K) spectrum of **Ph-7-Me₂**.

[Ph-6-Cl₂]₂: ^{1}H NMR (300 MHz, $CDCl_3$, 298.0 K): δ = 7.71–7.33 (m, 10H; CH_{aryl}) ppm. $^{13}C\{^1H\}$ NMR (75 MHz, $CDCl_3$, 298.0 K): δ = 164.7 (s; C=O), 137.9 (s; C_{aryl}), 136.6 (s; C_{aryl}), 136.0 (s; C_{aryl}), 129.4 (s; C_{aryl}), 129.1 (s; C_{aryl}), 129.1 (s; C_{aryl}), 128.5 (s; C_{aryl}), 128.3 (s; C_{aryl}), 127.5 (s; C_{aryl}), 125.5 (s; C_{aryl}), 117.7 (s; C_{aryl}), 74.8 (s; C(*C*(Cl)₂)C), 74.8 (s; C(*C*(Cl)₂)C), 73.8 (s; C=C) ppm [Some of the expected signals for aryl carbon atoms, carbonyl carbon atoms and C=C carbon atoms are not observed due to isochrony.]. MS (ESI) m/z (%): 428.96 $[M-H]^+$. Elemental analysis calcd for $C_{18}H_{10}Cl_4N_2O_2$: C 50.50, H 2.35, N 6.54, found: C 50.61, H 2.19, N 6.67.

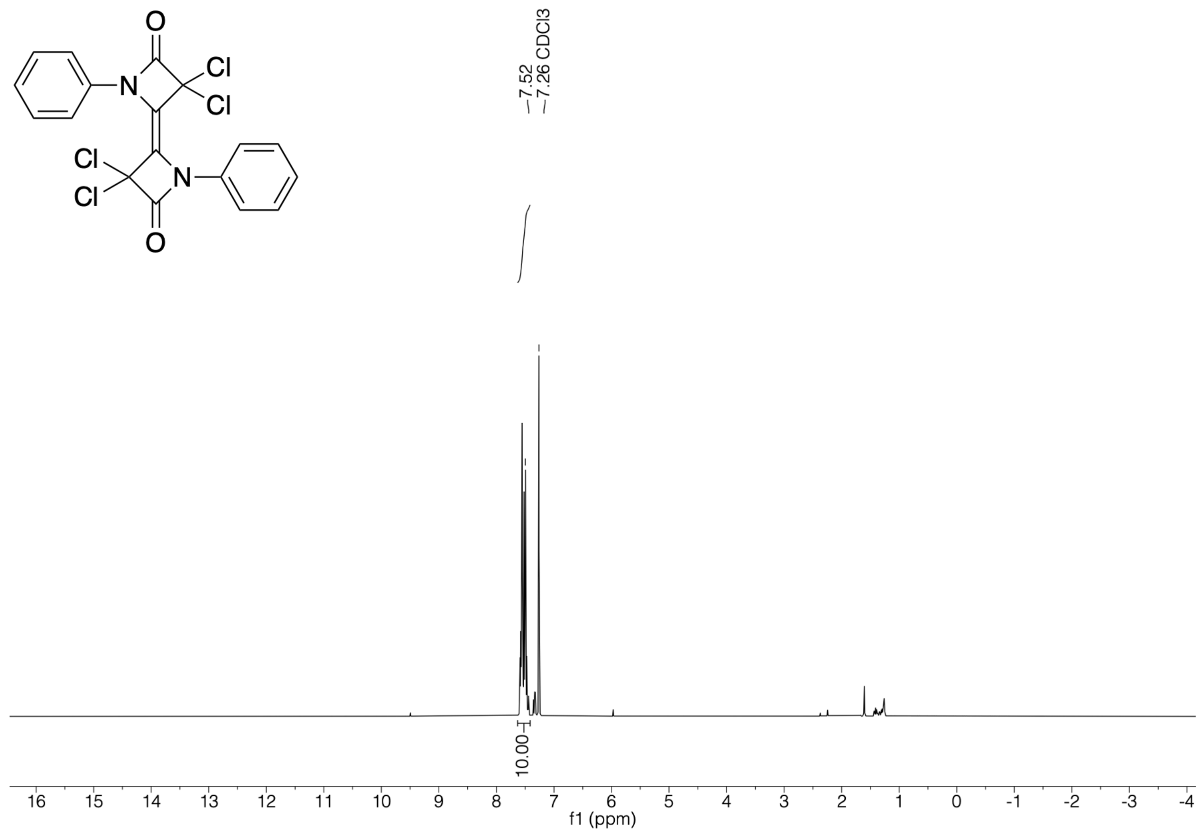

Figure SF54. ^{1}H NMR (300 MHz, $CDCl_3$, 298.0 K) spectrum of **$[Ph\text{-}6\text{-}Cl_2]_2$**.

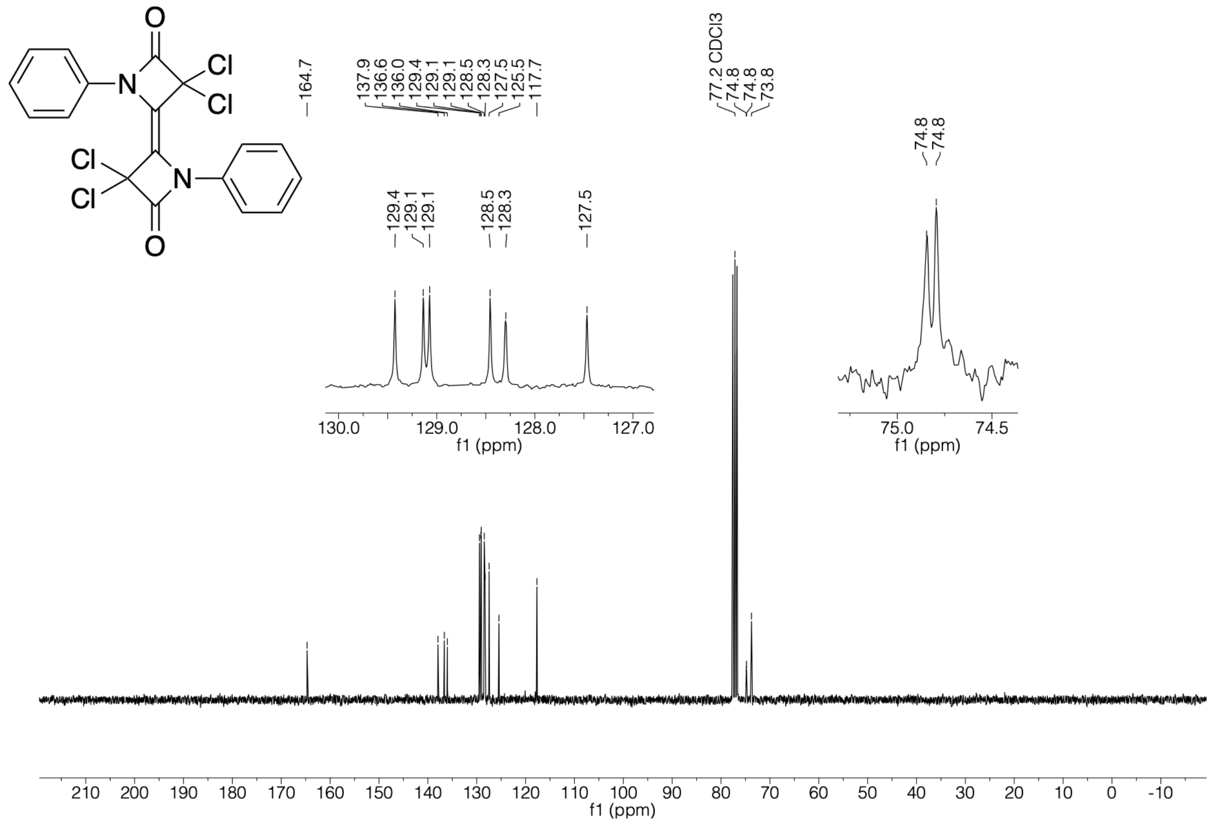

Figure SF55 $^{13}C\{^{1}H\}$ NMR (75 MHz, $CDCl_3$, 298.0 K) spectrum of **$[Ph\text{-}6\text{-}Cl_2]_2$**.

[Ph-6-Cl$_2$*N]$_2$: ^{1}H NMR (300 MHz, CDCl$_3$, 298.0 K): δ = 7.59–7.44 (m, 8H; CH$_{aryl}$), 7.20–7.15 (m, 2H; CH$_{aryl}$) ppm. ^{13}C{^{1}H} NMR (75 MHz, CDCl$_3$, 298.0 K): δ = 163.7 (s; C=O), 159.5 (s; C=N), 147.8 (s; NC$_{aryl}$), 130.7 (s; C$_{aryl}$), 126.4 (s; C$_{aryl}$), 124.5 (s; C$_{aryl}$), 83.5 (s; C(*C*(Cl)$_2$)C) ppm. MS (ESI) *m/z* (%): 456.96 [M-H]$^+$. Elemental analysis calcd for $C_{18}H_{10}Cl_4N_4O_2$: C 47.40, H 2.21, N 12.88, found: C 47.51, H 2.26, N 12.69.

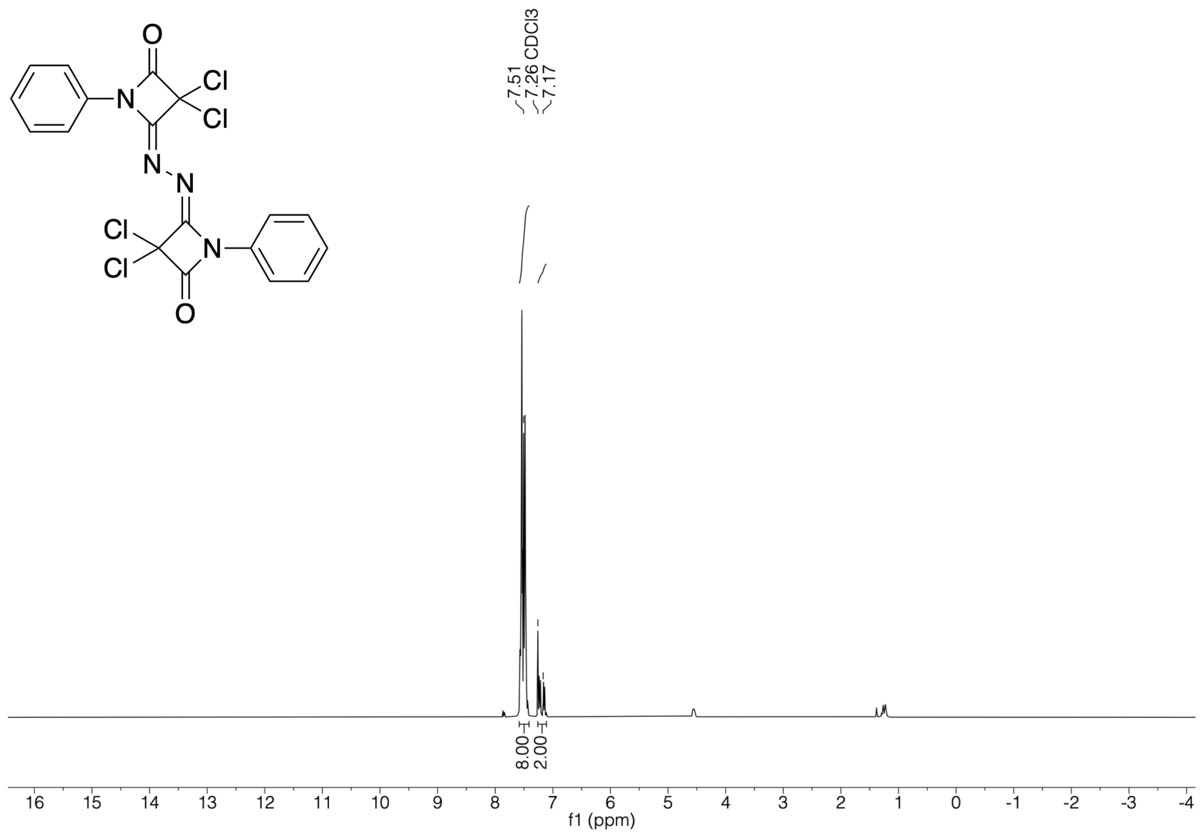

Figure SF56. ^{1}H NMR (300 MHz, CDCl$_3$, 298.0 K) spectrum of **[Ph-6-Cl$_2$*N]$_2$**.

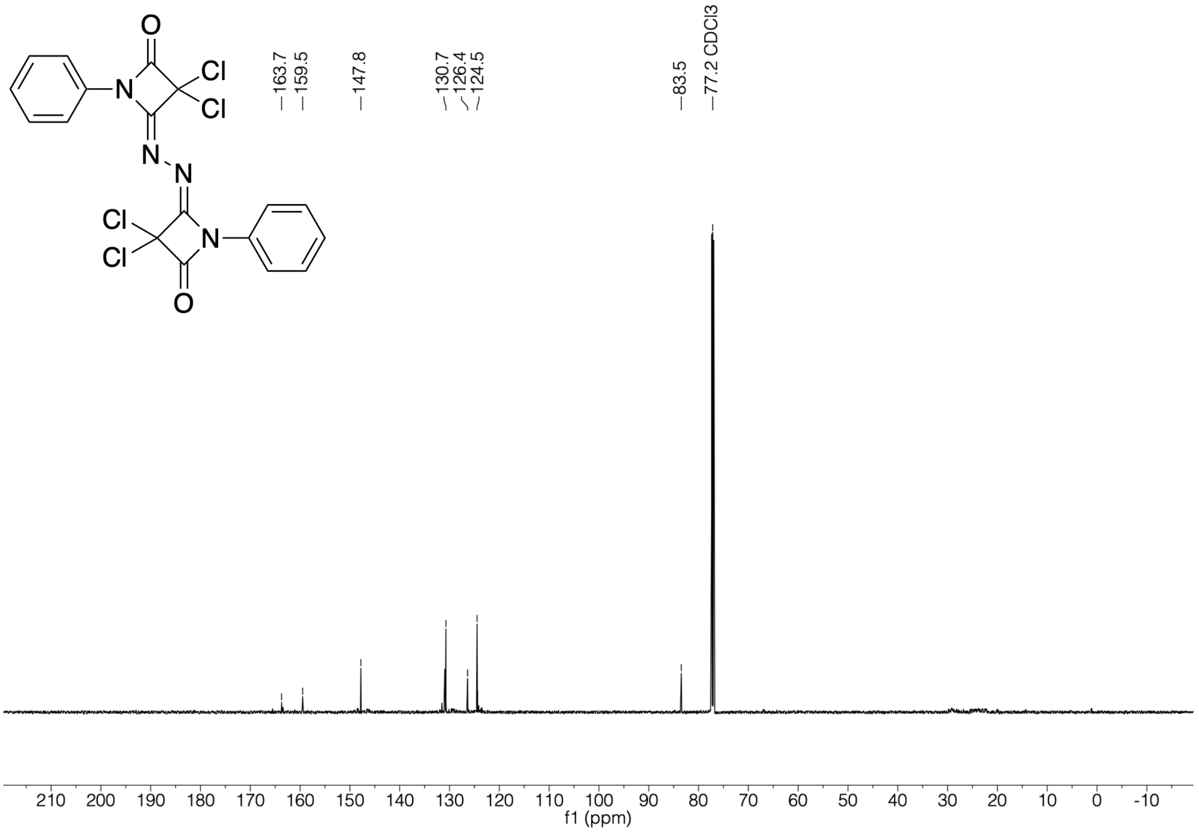

Figure SF57. $^{13}C\{^{1}H\}$ NMR (75 MHz, $CDCl_3$, 298.0 K) spectrum **[Ph-6-Cl$_2$*N]$_2$**.

Ph-7-Cl$_2$: ^{1}H NMR (300 MHz, $CDCl_3$, 298.0 K): δ = 7.50–7.44 (m, 2H; CH_{aryl}), 7.25–7.20 (m, 2H; CH_{aryl}), 7.11–7.09 (m, 1H; CH_{aryl}), 7.03 (s, 1H; NH), 3.02 (s, 1H; CH) ppm. No $^{13}C\{^{1}H\}$ NMR was recorded due to low amount of sample. MS (ESI) *m/z* (%): 204.01 $[M-H]^+$. Elemental analysis calcd for $C_8H_7Cl_2NO$: C 47.09, H 3.46, N 6.86, found: C 47.42, H 3.12, N 7.04.

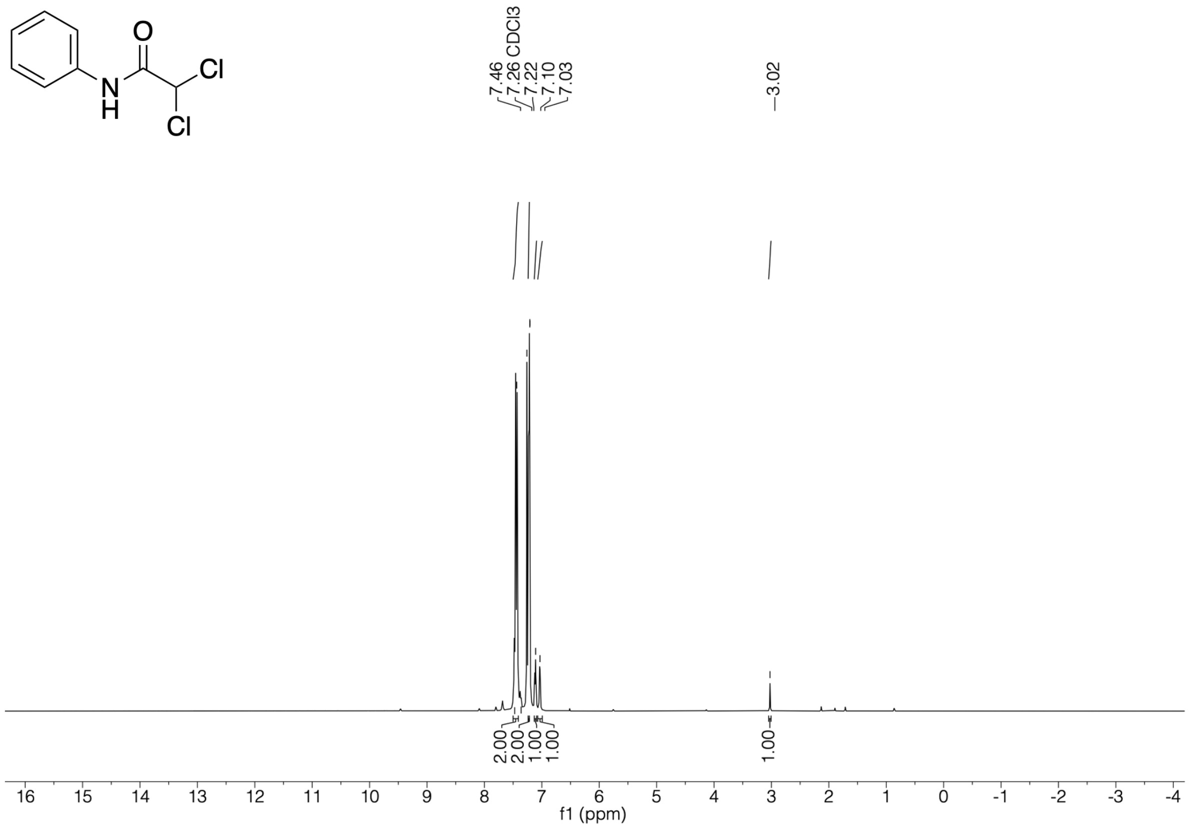

Figure SF58. ^{1}H NMR (300 MHz, $CDCl_3$, 298.0 K) spectrum of **Ph-7-Cl$_2$**.

[Ph-6-Ph$_2$]$_2$: ^{1}H NMR (300 MHz, $CDCl_3$, 298.0 K): δ = 7.48–7.44 (m, 2H; CH_{aryl}), 7.39–7.32 (m, 6H; CH_{aryl}), 7.32–7.28 (m, 6H; CH_{aryl}), 7.11–7.09 (m, 1H; CH_{aryl}), ppm [Integrals are given for monomeric unit.]. $^{13}C\{^1H\}$ NMR (75 MHz, $CDCl_3$, 298.0 K): δ = 170.2 (s; C=O), 139.2 (s; C_{aryl}), 137.8 (s; C_{aryl}), 129.1 (s; C_{aryl}), 129.1 (s; C_{aryl}), 127.7 (s; C_{aryl}), 124.7 (s; C_{aryl}), 119.9 (s; C=C), 60.3 (s; C(*C*(Ph)$_2$)C) ppm [Some of the expected signals for aryl carbon atoms are not observed due to isochrony.]. MS (ESI) *m/z* (%): 595.24 [M-H]$^+$. Elemental analysis calcd for $C_{42}H_{30}N_2O_2$: C 84.82, H 5.08, N 4.71, found: C 84.62, H 4.85, N 5.12.

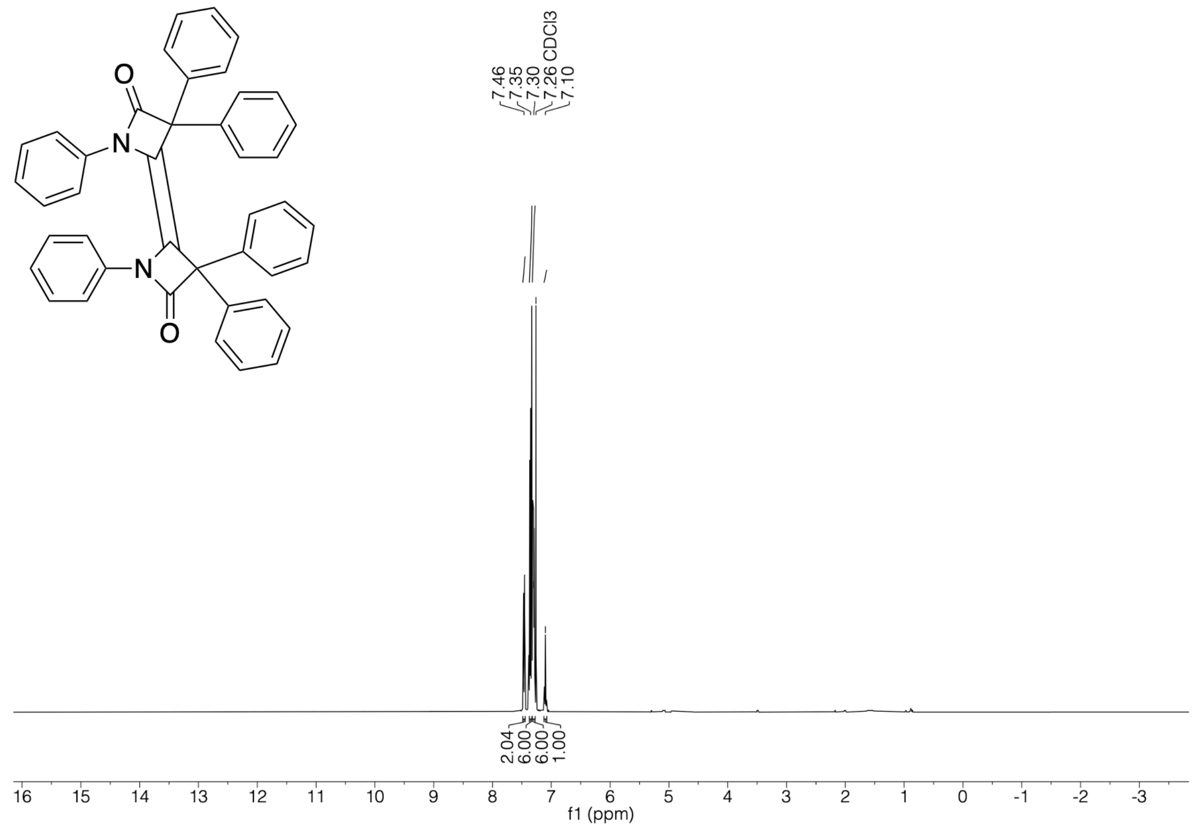

Figure SF59. ^{1}H NMR (300 MHz, $CDCl_3$, 298.0 K) spectrum of **[Ph-6-Ph$_2$]$_2$**.

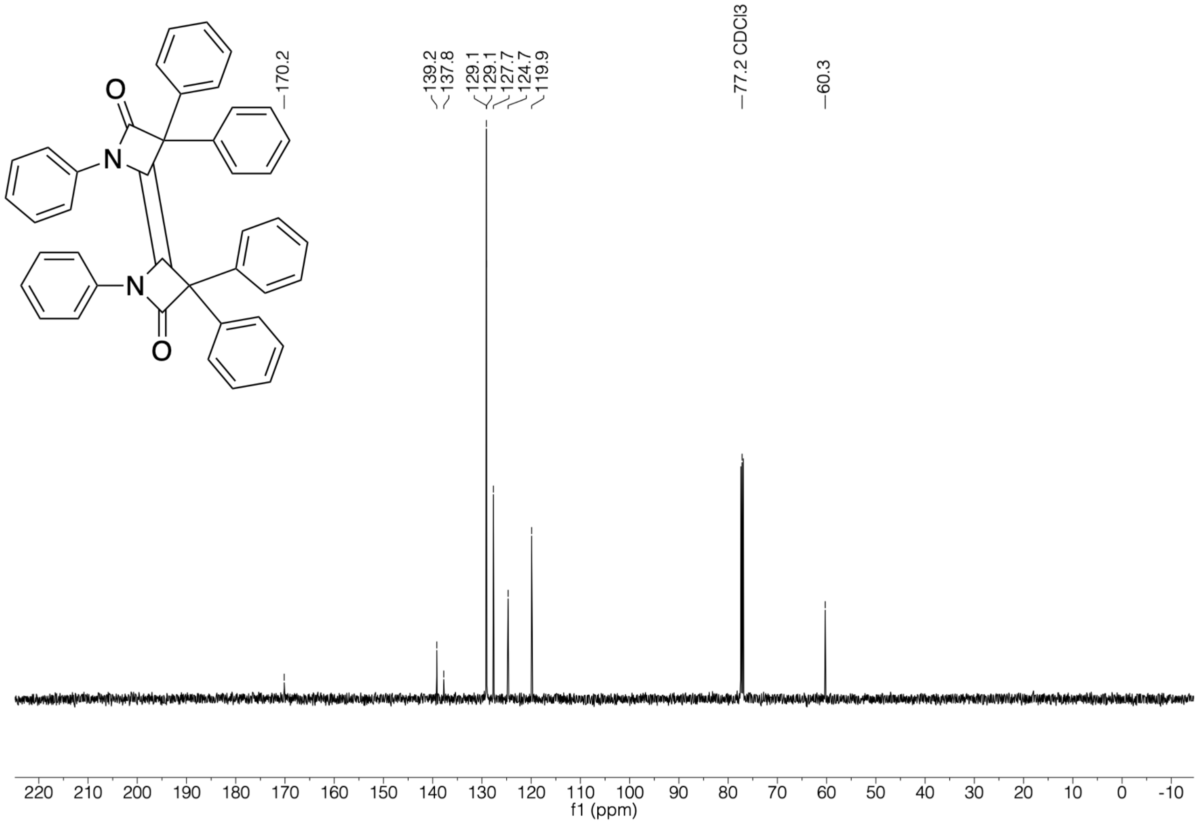

Figure SF60. $^{13}C\{^{1}H\}$ NMR (75 MHz, $CDCl_3$, 298.0 K) spectrum of **[Ph-6-Ph$_2$]$_2$**.

[Ph-6-Ph$_2$*N]$_2$: ^{1}H NMR (300 MHz, C_6D_6, 298.0 K): δ = 8.10–8.08 (m, 1H; CH_{aryl}), 7.90–7.88 (m, 1H; CH_{aryl}), 7.77–7.74 (m, 2H; CH_{aryl}), 7.68–7.57 (m, 7H; CH_{aryl}), 7.13–6.86 (m, 16H; CH_{aryl}), 6.87–6.79 (m, 3H; CH_{aryl}) ppm. $^{13}C\{^1H\}$ NMR (75 MHz, C_6D_6, 298.0 K): δ = 168.5 (s; C=O), 157.6 (s; C=N), 136.7 (s; C_{aryl}), 135.9 (s; C_{aryl}), 129.5 (s; C_{aryl}), 129.4 (s; C_{aryl}), 129.2 (s; C_{aryl}), 127.0 (s; C_{aryl}), 121.1 (s; C_{aryl}), 73.7 (s; C(*C*(Ph)$_2$)C) ppm. MS (ESI) *m/z* (%): 623.26 [M-H]$^+$. Elemental analysis calcd for $C_{42}H_{30}N_4O_2$: C 81.01, H 4.86, N 9.00, found: C 80.89, H 4.83, N 9.11.

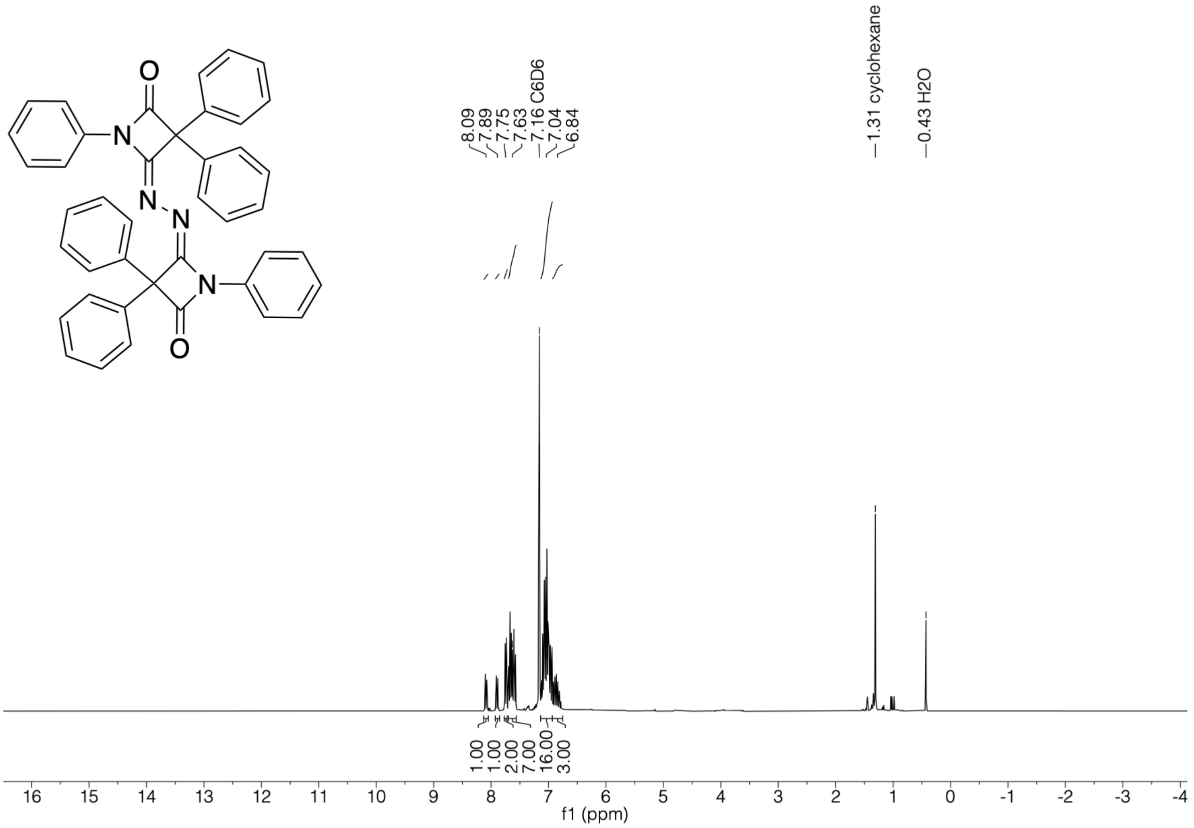

Figure SF61. ^{1}H NMR (300 MHz, C_6D_6, 298.0 K) spectrum of **[Ph-6-Ph$_2$*N]$_2$**.

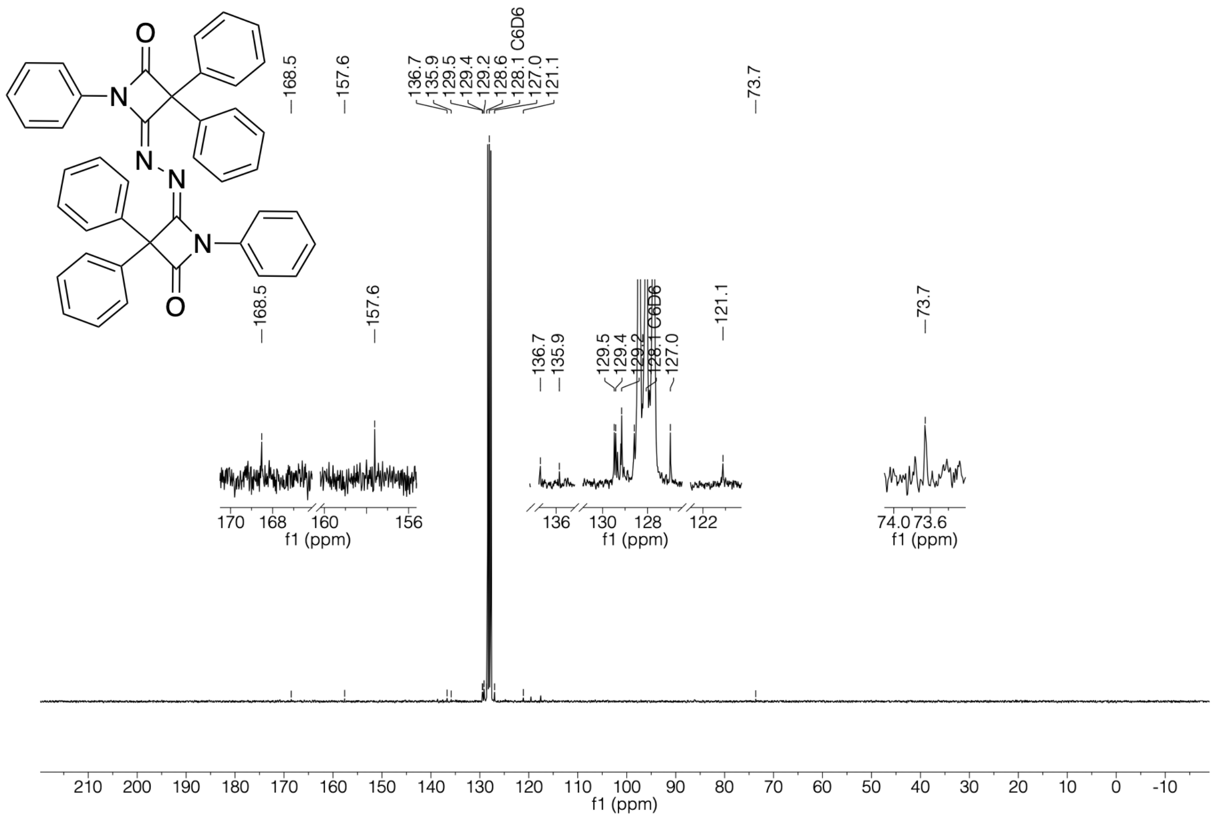

Figure SF62. $^{13}C\{^1H\}$ NMR (75 MHz, C_6D_6, 298.0 K) spectrum of **[Ph-6-Ph$_2$*N]$_2$**.

Ph-7-Ph$_2$: ^1H NMR (300 MHz, $CDCl_3$, 298.0 K): δ = 7.69–7.65 (m, 2H; CH_{aryl}), 7.60–7.56 (m, 2H; CH_{aryl}), 7.48–7.39 (m, 10H; CH_{aryl}), 7.35 (s, 1H; NH), 7.23–7.19 (m, 1H; CH_{aryl}), 6.62 (s, 1H; CH) ppm. $^{13}C\{^1H\}$ NMR (75 MHz, $CDCl_3$, 298.0 K): δ = 164.7 (s; C=O), 137.9 (s; C_{aryl}), 136.6 (s; C_{aryl}), 136.0 (s; C_{aryl}), 129.4 (s; C_{aryl}), 129.1 (s; C_{aryl}), 128.5 (s; C_{aryl}), 128.3 (s; C_{aryl}), 127.5 (s; C_{aryl}), 125.5 (s; C_{aryl}), 117.1 (s; C_{aryl}), 73.8 (s; C(*C*(Ph)$_2$)) ppm. MS (ESI) *m/z* (%): 288.13 [M-H]$^+$. Elemental analysis calcd for $C_{20}H_{17}NO$: C 83.59, H 5.96, N 4.87, found: C 83.46, H 5.88, N 5.12.

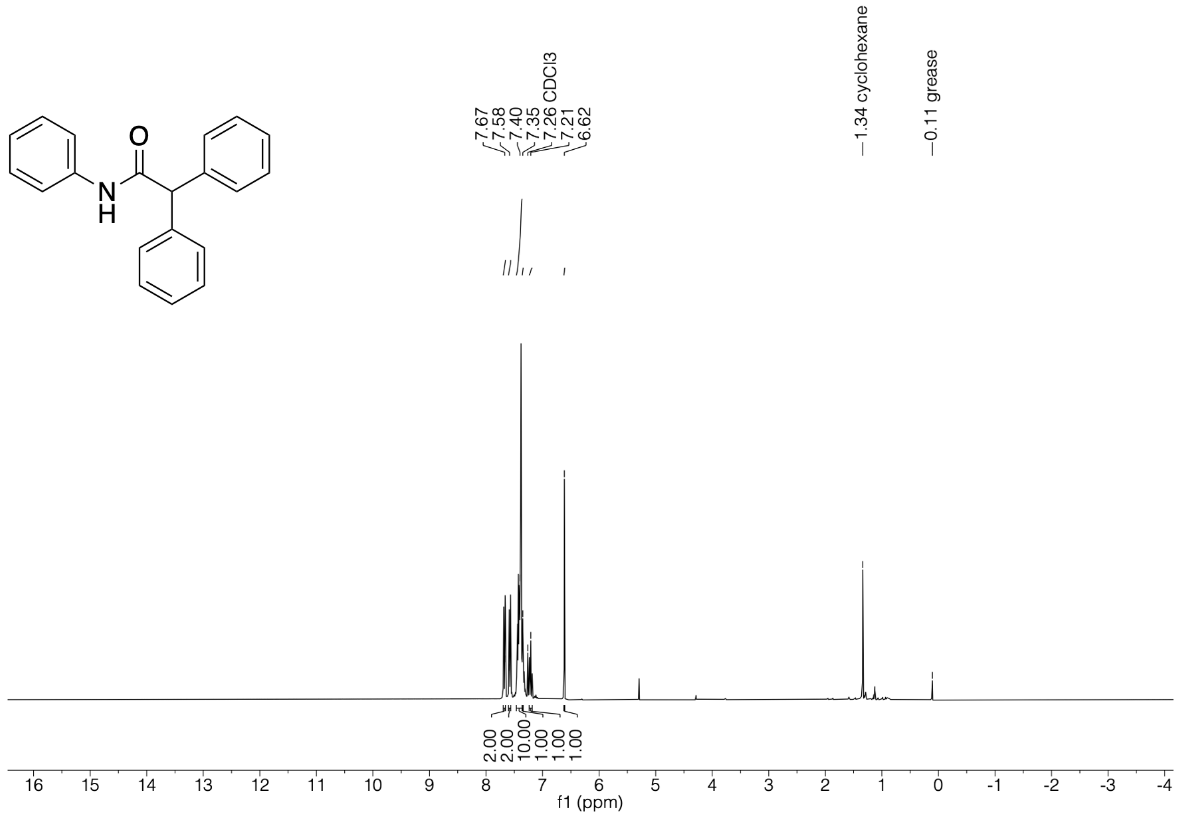

Figure SF63. ^{1}H NMR (300 MHz, $CDCl_3$, 298.0 K) spectrum of **Ph-7-Ph₂**.

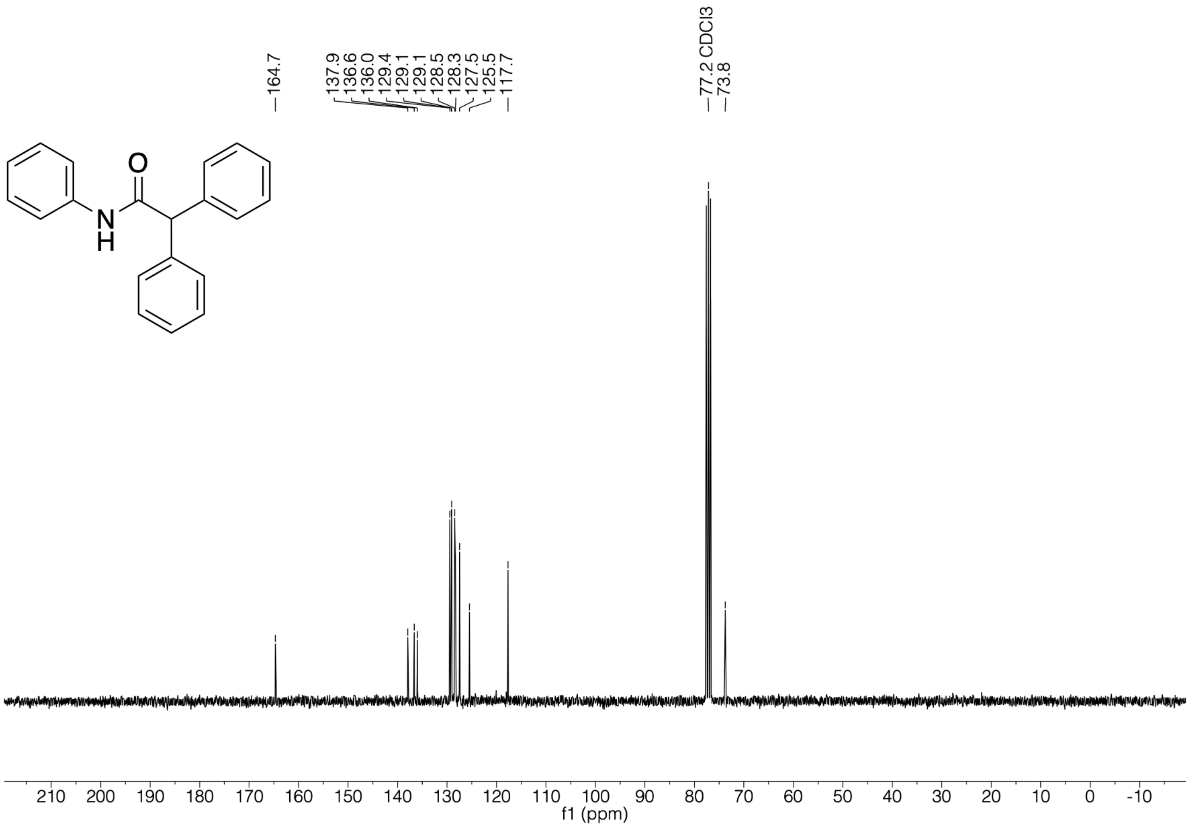

Figure SF64. $^{13}C\{^{1}H\}$ NMR (75 MHz, $CDCl_3$, 298.0 K) spectrum of **Ph-7-Ph₂**.

Ph-10-Ph$_2$: ^{1}H NMR (300 MHz, $CDCl_3$, 298.0 K): δ = 7.44–7.32 (m, 12H; CH_{aryl}), 7.32–7.30 (m, 1H; CH_{aryl}), 7.26–7.23 (m, 2H; CH_{aryl}) ppm. $^{13}C\{^1H\}$ NMR (75 MHz, $CDCl_3$, 298.0 K): δ = 190.7 (s; C=N), 140.8 (s; C_{aryl}), 134.1 (s; C_{aryl}), 129.7 (s; C_{aryl}), 129.0 (s; C_{aryl}), 128.0 (s; C_{aryl}), 126.7 (s; C_{aryl}), 124.2 (s; C_{aryl}), 78.2 (s; C(*C*(Ph)$_2$)) ppm. No MS-analysis could be performed due to the sensitivity of the compound towards moisture. Elemental analysis calcd for $C_{20}H_{15}N$: C 89.19, H 5.61, N 5.20, found: C 88.98, H 5.49, N 5.13. Analytical data are in accordance with results reported in literature.[7]

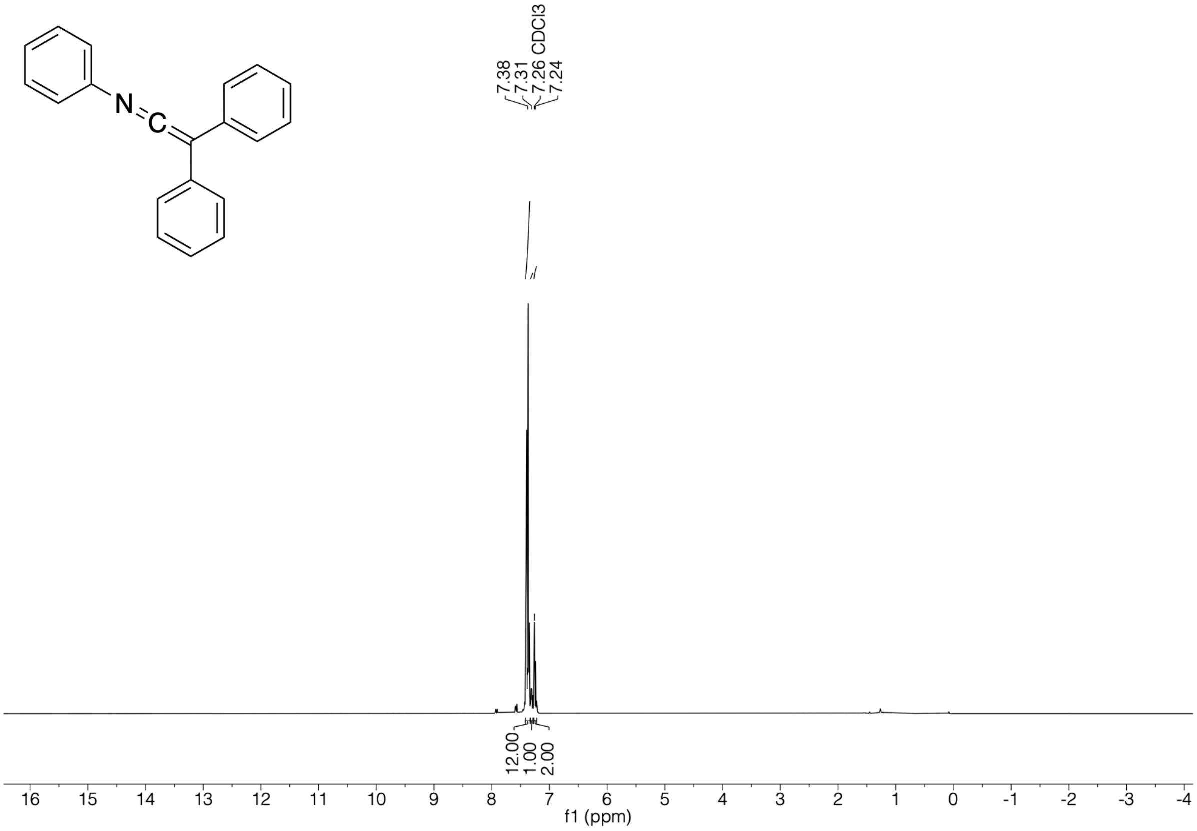

Figure SF65. ^{1}H NMR (300 MHz, $CDCl_3$, 298.0 K) spectrum of **Ph-10-Ph$_2$**.

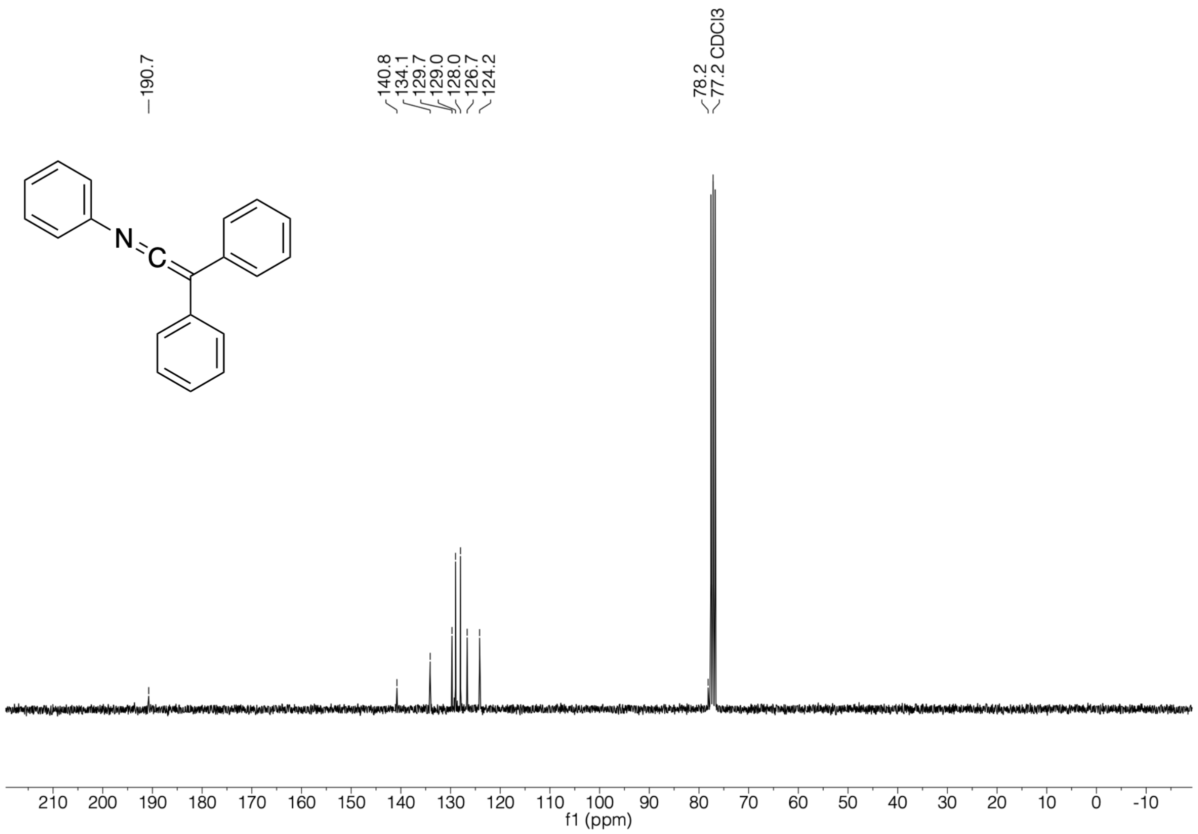

Figure SF66. $^{13}C\{^1H\}$ NMR (75 MHz, $CDCl_3$, 298.0 K) spectrum of **Ph-10-Ph$_2$**.

Ph-7-sCy: ^1H NMR (300 MHz, $CDCl_3$, 298.0 K): δ = 7.81 (br s, 1H; NH), 7.39–7.35 (m, 2H; CH_{aryl}), 7.16–7.14 (m, 2H; CH_{aryl}), 6.98–6.96 (m, 1H; CH_{aryl}), 2.10–1.82 (m, 4H; CH_{aliph}), 1.76–1.42 (m, 3H; CH_{aliph}), 1.41–1.34 (m, 2H; CH_{aliph}), 1.28–1.20 (m, 2H; CH_{aliph}) ppm. $^{13}C\{^1H\}$ NMR (75 MHz, $CDCl_3$, 298.0 K): δ = 167.9 (s; C=O), 137.3 (s; C_{aryl}), 128.7 (s; C_{aryl}), 124.5 (s; C_{aryl}), 120.4 (s; C_{aryl}), 29.5 (s; C_{aliph}), 26.7 (s; C_{aliph}), 24.8 (s; C_{aliph}), 22.3 (s; C_{aliph}) ppm. MS (ESI) *m/z* (%): 204.14 $[M-H]^+$. Elemental analysis calcd for $C_{20}H_{17}NO$: C 76.81, H 8.43, N 6.89, found: C 76.92, H 6.61, N 6.44.

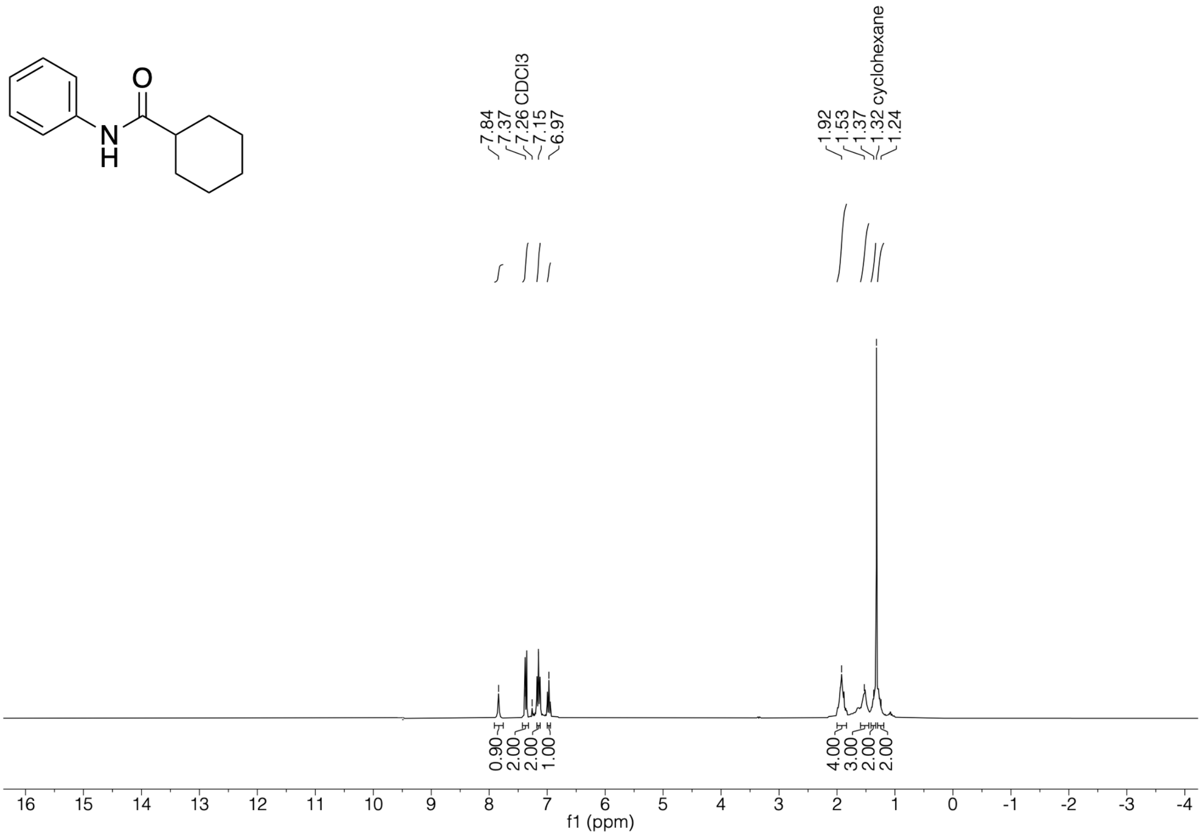

Figure SF67. ^{1}H NMR (300 MHz, $CDCl_3$, 298.0 K) spectrum of **Ph-7-sCy**.

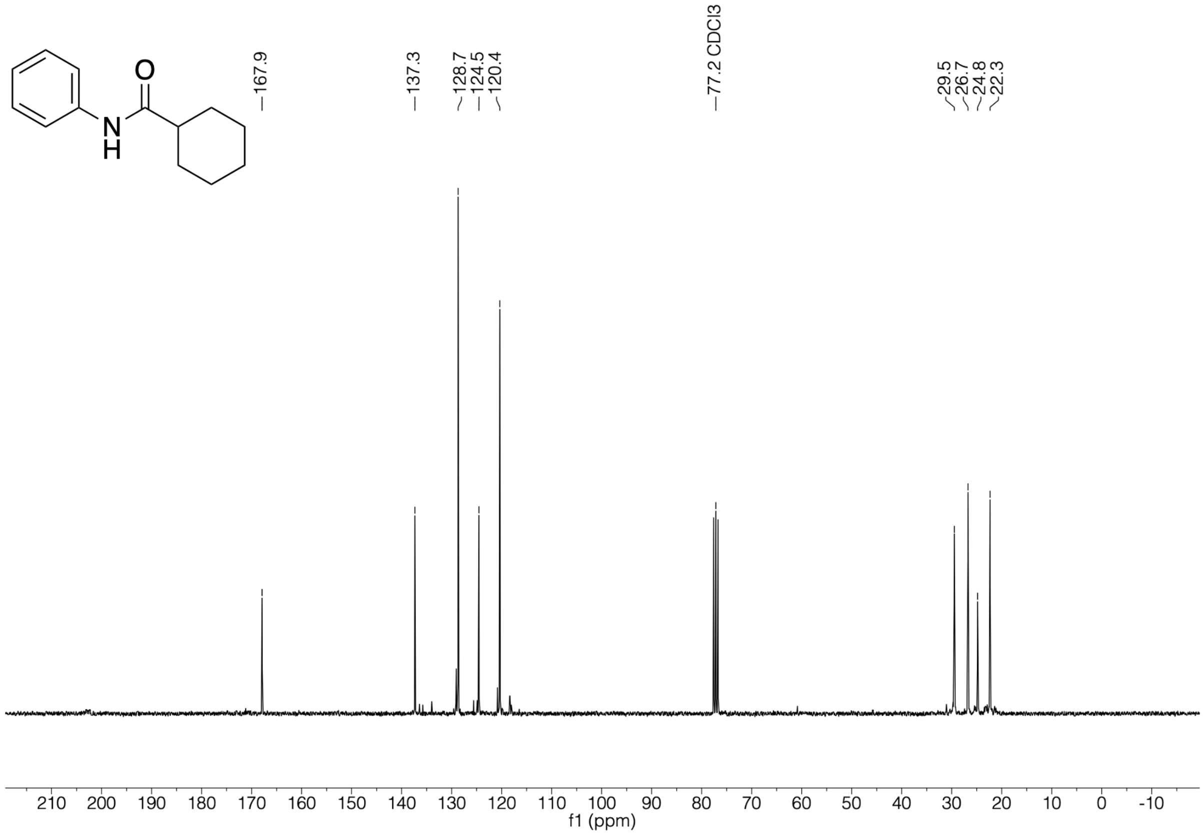

Figure SF68. ^{13}C{^{1}H} NMR (75 MHz, $CDCl_3$, 298.0 K) spectrum of **Ph-7-sCy**.

[Mes-6-Me$_2$]$_2$: ^{1}H NMR (300 MHz, $CDCl_3$, 298.0 K): δ = 6.68 (d, J = 1 Hz, 2 H; CH_{meta}), 2.15 (s, 6H; $CH_{3\text{-}ortho}$), 2.09 (s, 3H; $CH_{3\text{-}para}$), 1.35 (s, 6H; CH_3) ppm. [Integrals are given for monomeric unit.]. MS (ESI) *m/z* (%): 431.27 [M-H]$^+$.

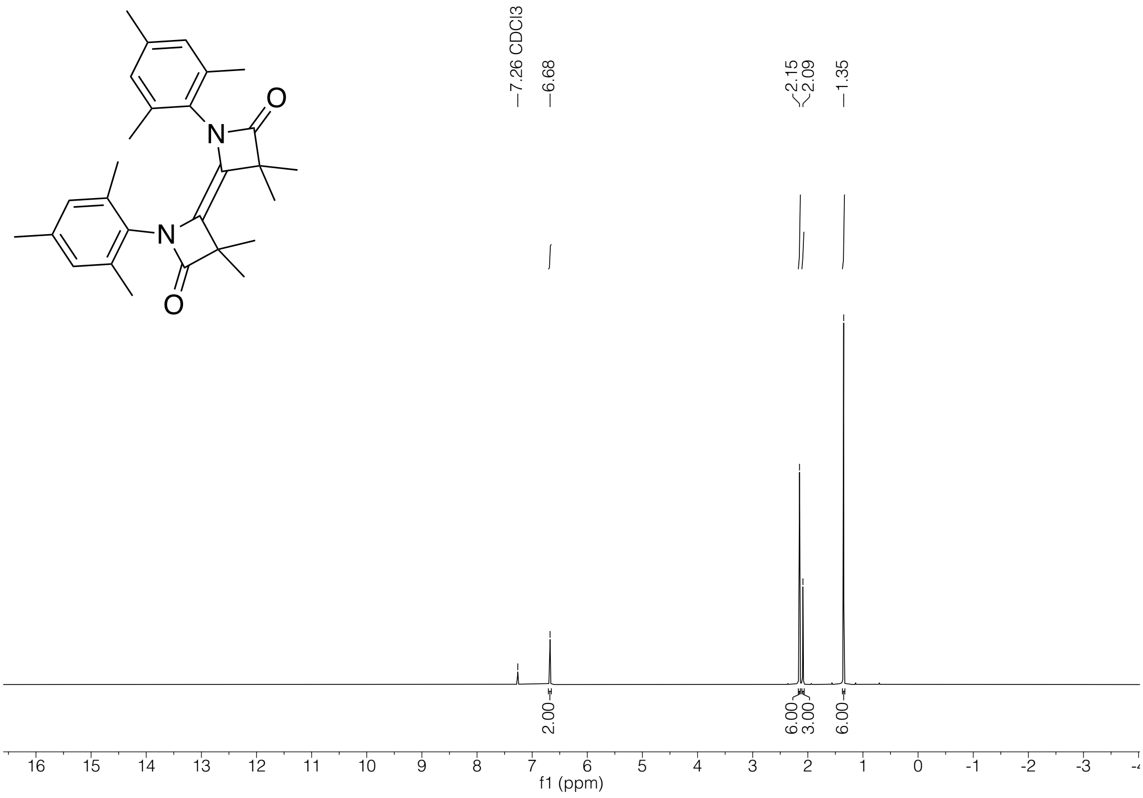

Figure SF69. ^{1}H NMR (300 MHz, $CDCl_3$, 298.0 K) spectrum of **[Mes-6-Me$_2$]$_2$**.

[Mes-6-Me$_2$*N]$_2$: Due to small amount of sample, only MS-analysis was performed. MS (ESI) *m/z* (%): 458.28 [M-H]$^+$. The same type of compound is obtained if the isostructural precursor **Mes-5-Cl$_2$** is used, yielding **[Mes-6-Cl$_2$*N]$_2$**. However, the latter could not be sufficiently purified. Thus, **[Mes-6-Cl$_2$*N]$_2$** was only characterized by single crystal diffraction (see section III. Crystallographic Data). Overall the findings show the same outcome of the described thermolysis reactions for same type of substituents R^2.

Mes-7-Me$_2$: ^{1}H NMR (300 MHz, $CDCl_3$, 298.0 K): δ = 7.01 (br s, 1H; NH), 6.82 (s, 2H; CH_{aryl}), 2.55 (sept, J = 7 Hz, 1H; CH), 2.24 (s, 3H; CH_{para}), 2.10 (s, 6H; CH_{ortho}), 1.22 (d, J = 7 Hz, 6H; CH_3) ppm. $^{13}C\{^{1}H\}$ NMR (75 MHz, $CDCl_3$, 298.0 K): δ = 175.7 (s; C=O), 136.5 (s; C_{aryl}), 135.2 (s; C_{aryl}), 131.3 (s; C_{aryl}), 128.7 (s; C_{aryl}), 35.6 (s; C_{aliph}), 21.0 (s; C_{aliph}), 19.9 (s; C_{aliph}), 18.2 (s; C_{aliph}) ppm. MS (ESI) m/z (%): 206.15 $[M-H]^{+}$. Elemental analysis calcd for $C_{13}H_{19}NO$: C 76.06, H 9.33, N 6.82, found: C 75.85, H 9.17, N 6.76.

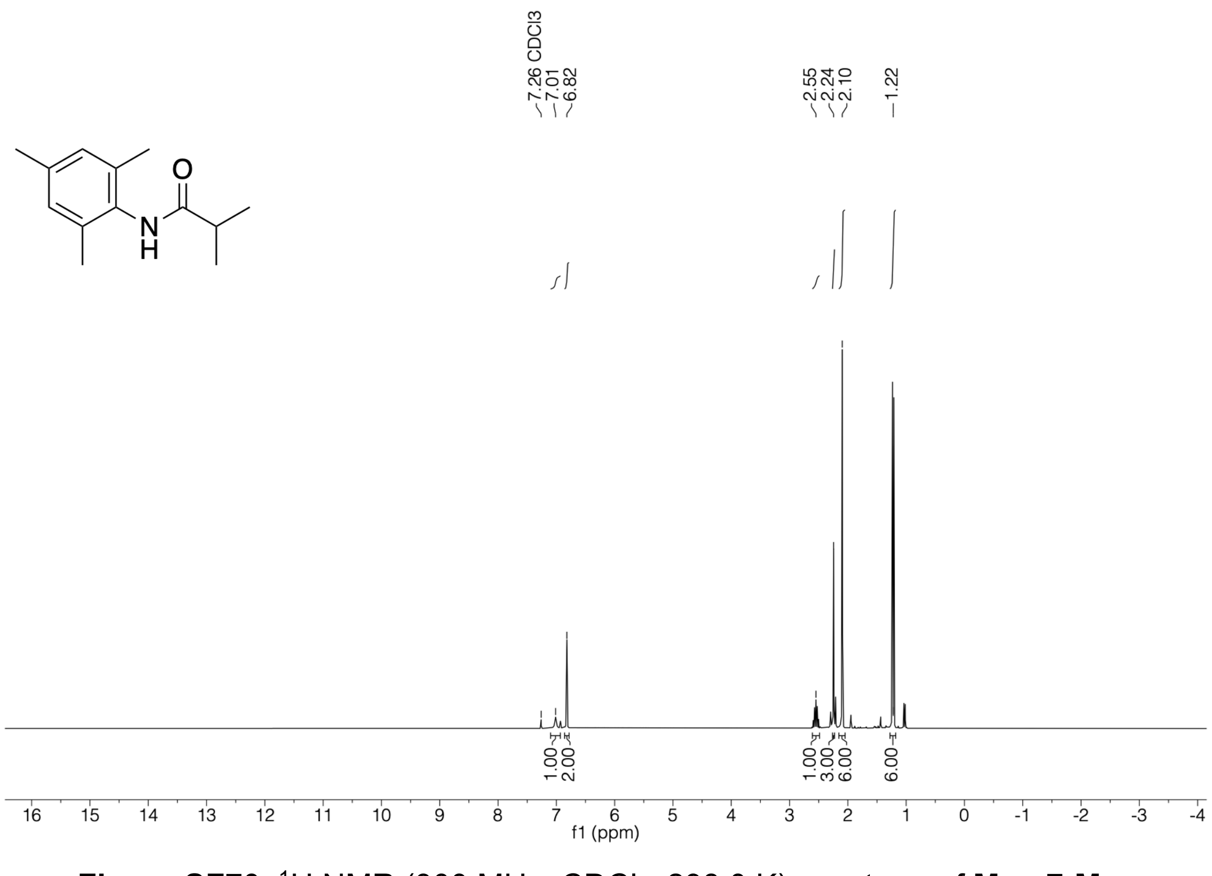

Figure SF70. ^{1}H NMR (300 MHz, $CDCl_3$, 298.0 K) spectrum of **Mes-7-Me$_2$**.

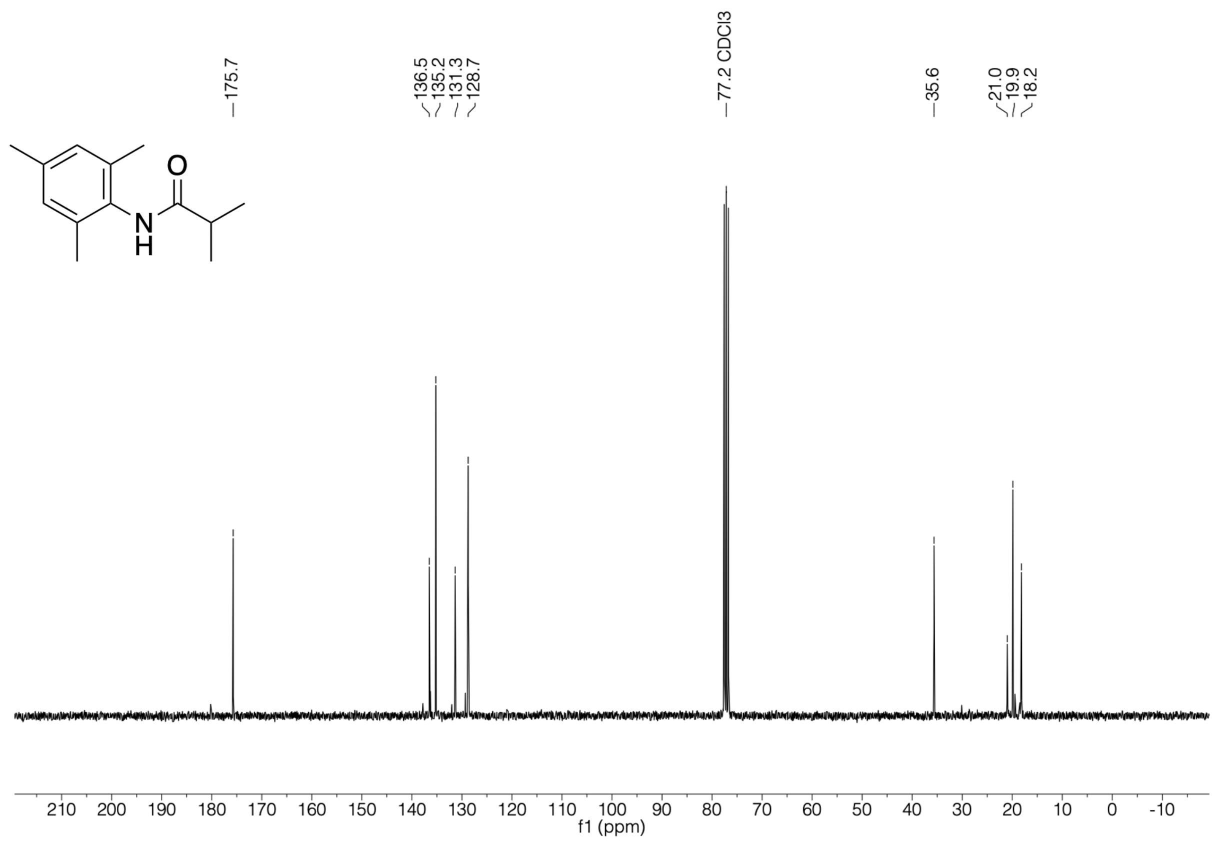

Figure SF71. $^{13}C\{^1H\}$ NMR (75 MHz, $CDCl_3$, 298.0 K) spectrum of **Mes-7-Me$_2$**.

Mes-10-Ph$_2$: ^1H NMR (300 MHz, $CDCl_3$, 298.0 K): δ = 7.37–7.33 (m, 8H; CH_{aryl}), 7.24–7.18 (m, 2H; CH_{aryl}), 6.90 (br s, 2 H; CH_{ortho}), 2.31–2.29 (m, 9H; CH_{aliph}) ppm. $^{13}C\{^1H\}$ NMR (75 MHz, $CDCl_3$, 298.0 K): δ = 184.5 (s; C=N), 136.3 (s; C_{aryl}), 135.5 (s; C_{aryl}), 135.2 (s; C_{aryl}), 132.0 (s; C_{aryl}), 129.4 (s; C_{aryl}), 128.9 (s; C_{aryl}), 127.9 (s; C_{aryl}), 125.9 (s; C_{aryl}), 78.2 (s; C(*C*(Ph)$_2$)), 21.0 (s; C_{para}), 19.0 (s; C_{ortho}) ppm. Elemental analysis calcd for $C_{23}H_{12}N$: C 88.71, H 6.80, N 4.50, found: C 88.68, H 6.73, N 4.39.

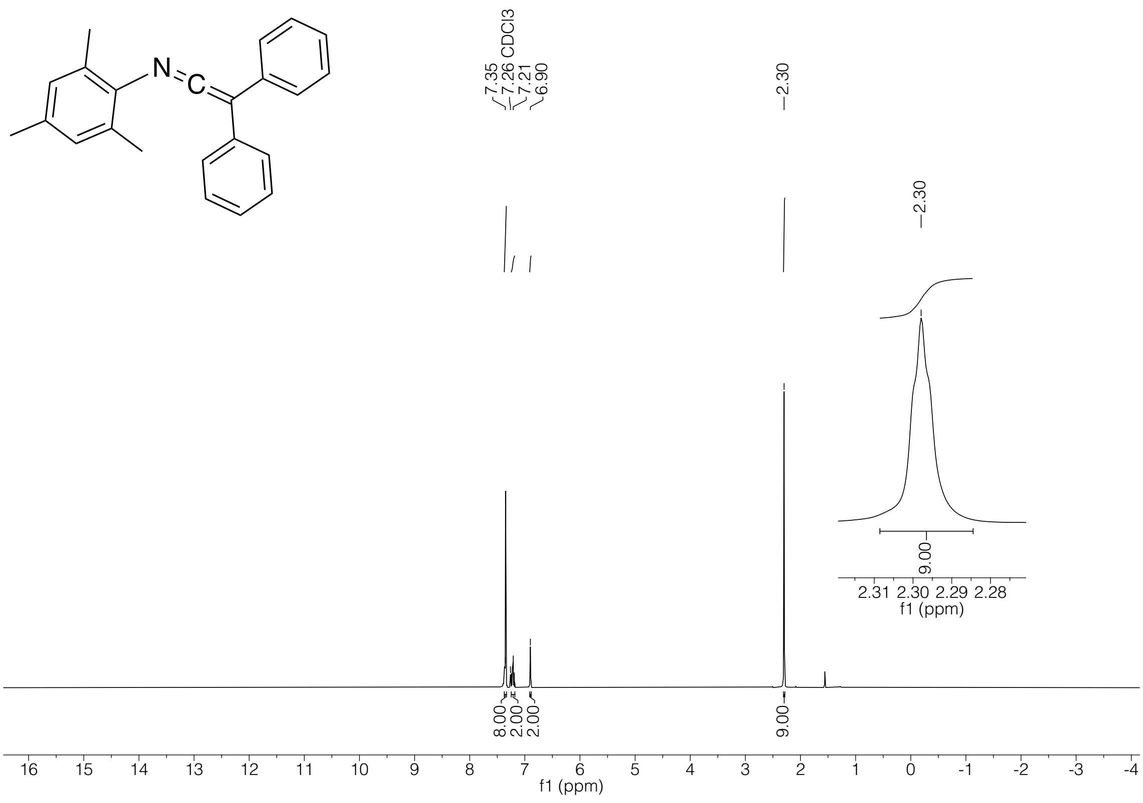

Figure SF72. ^{1}H NMR (300 MHz, $CDCl_3$, 298.0 K) spectrum of **Mes-10-Ph$_2$**.

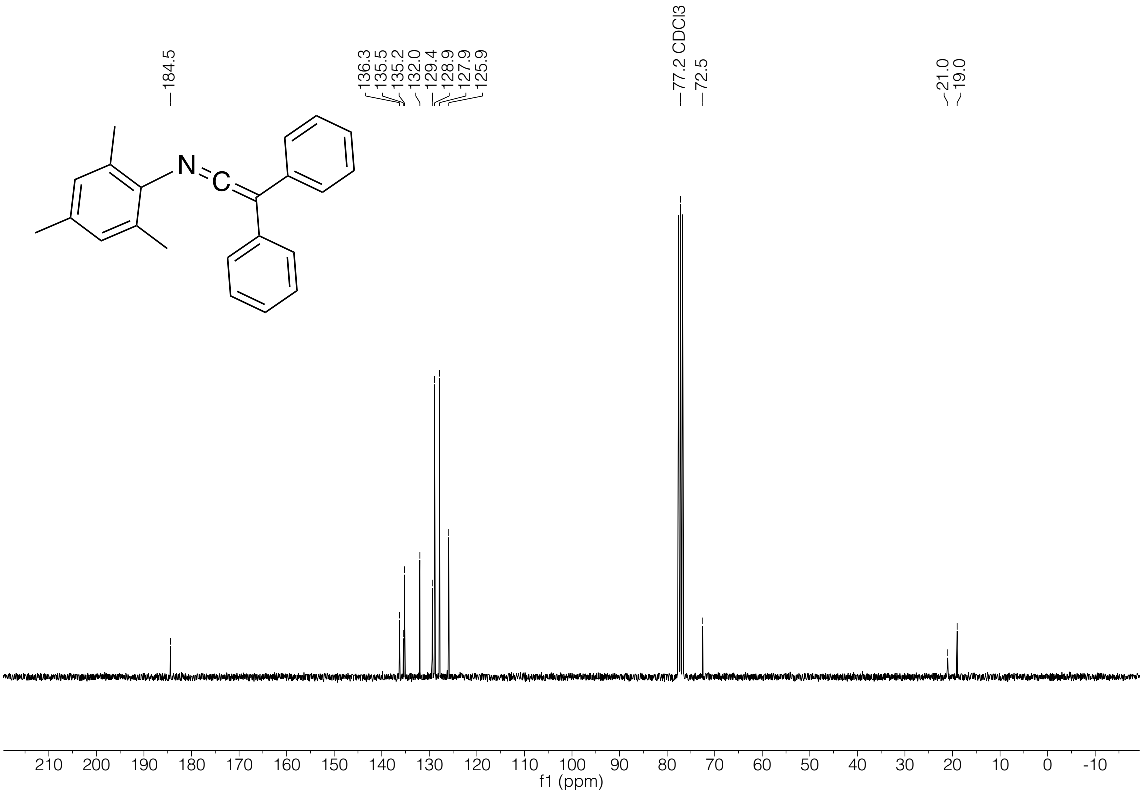

Figure SF73. $^{13}C\{^{1}H\}$ NMR (75 MHz, $CDCl_3$, 298.0 K) spectrum of **Mes-10-Ph$_2$**.

[Dipp-6-Me$_2$]$_2$: ^{1}H NMR (300 MHz, CD_2Cl_2, 298.0 K): δ = 7.426–7.40 (m, 1H; CH_{para}), 7.26–7.22 (m, 2H; CH_{meta}), 2.89 (sept, J = 7 Hz, 2H; CH_{Dipp}), 1.52 (s, 6H; CH_3), 1.22 (d, J = 7 Hz, 12H; CH_{3Dipp}) ppm. $^{13}C\{^1H\}$ NMR (75 MHz, CD_2Cl_2, 298.0 K): δ = 175.6 (s; C=O), 146.9 (s; C_{aryl}), 130.6 (s; C_{aryl}), 125.8 (s; C_{aryl}), 124.1 (s; C_{aryl}), 59.9 (s; C=C), 29.6 (s; C_{aliph}), 23.7 (s; C_{aliph}), 18.1 (s; C_{aliph}) ppm. MS (ESI) m/z (%): 515.37 $[M-H]^+$. Elemental analysis calcd for $C_{34}H_{46}N_2O_2$: C 79.33, H 9.01, N 5.44, found: C 78.95, H 8.63, N 5.21.

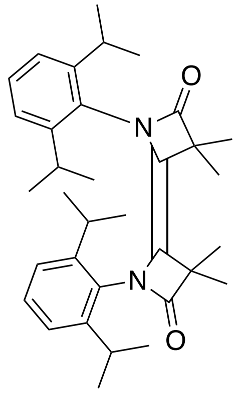

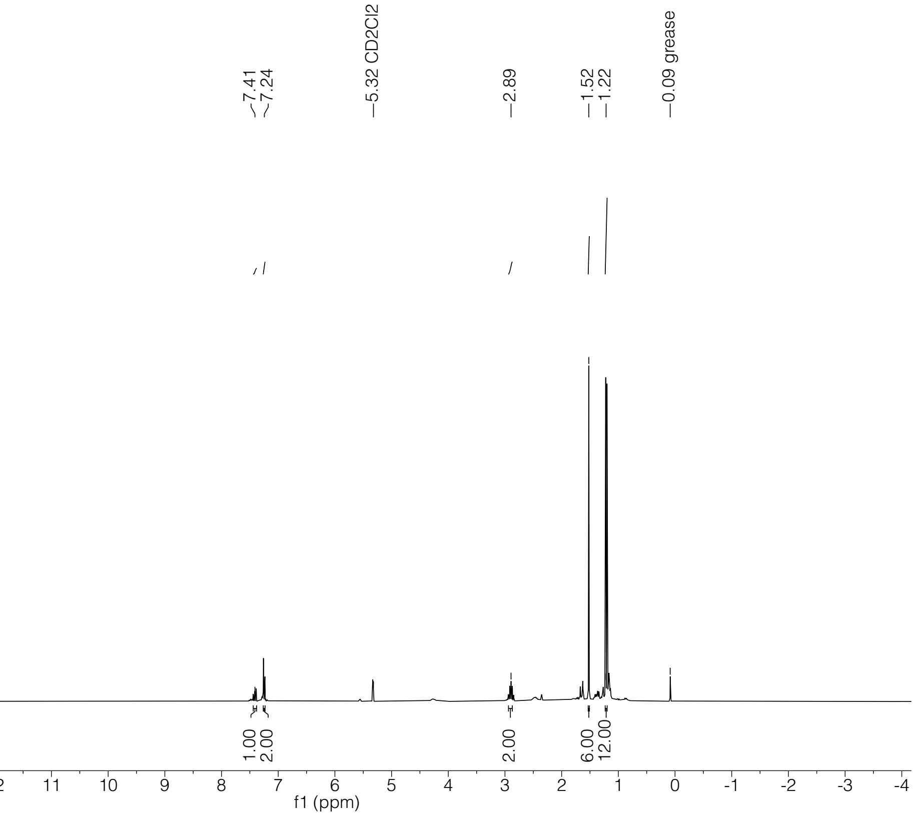

Figure SF74. ^{1}H NMR (300 MHz, CD_2Cl_2, 298.0 K) spectrum of **[Dipp-6-Me$_2$]$_2$**.

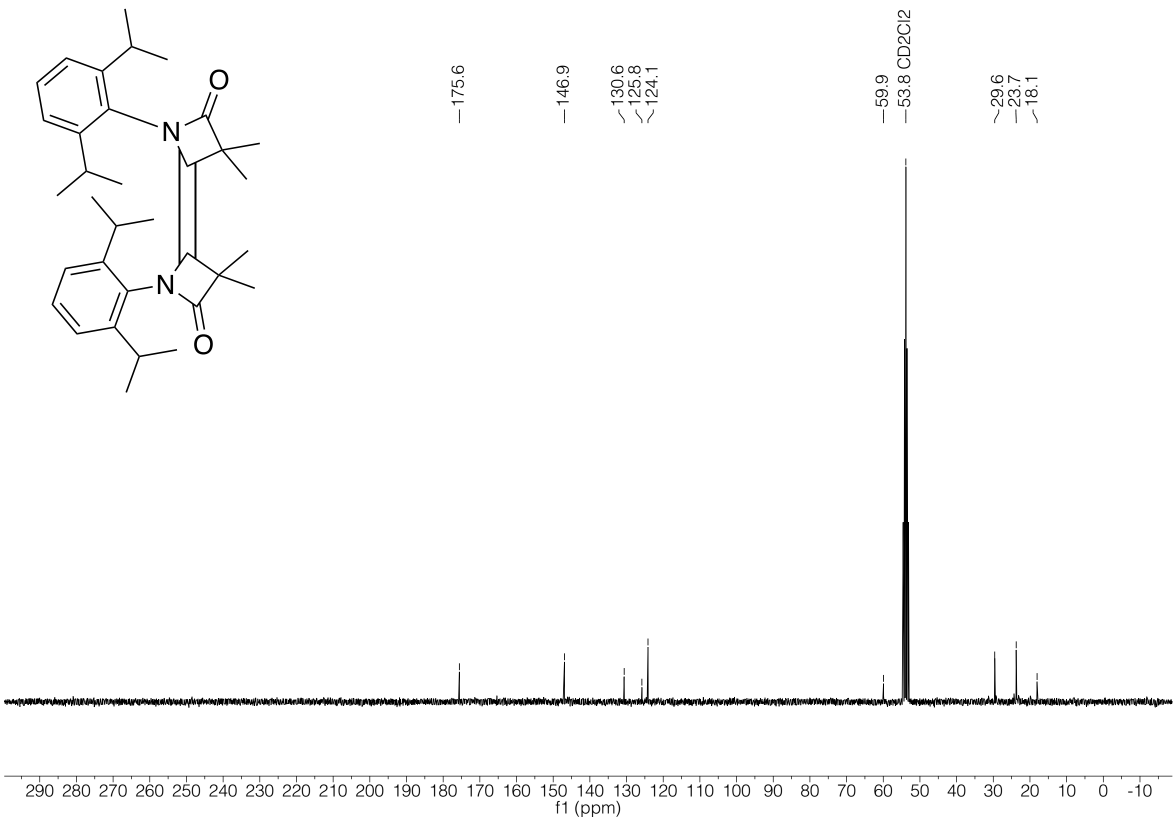

Figure SF75. $^{13}C\{^1H\}$ NMR (75 MHz, CD_2Cl_2, 298.0 K) spectrum of **[Dipp-6-Me$_2$]$_2$**.

[Dipp-6-Me$_2$*N]$_2$: Due to small amount of sample, only MS-analysis was performed. MS (ESI) *m/z* (%): 543.37 $[M\text{-}H]^+$.

Dipp-7-Me$_2$: ^1H NMR (300 MHz, $CDCl_3$, 298.0 K): δ = 7.30–7.28 (m, 1H; CH_{para}), 7.20–7.13 (m, 2H; CH_{meta}), 3.05 (sept, J = 7 Hz, 2H; CH_{Dipp}), 2.63 (sept, J = 7 Hz, 1H; CH), 1.31 (d, J = 7 Hz, 6H; CH_3), 1.19 (d, J = 7 Hz, 6H; CH_3) ppm. $^{13}C\{^1H\}$ NMR (75 MHz, $CDCl_3$, 298.0 K): δ = 176.1 (s; C=O), 146.4 (s; C_{aryl}), 131.2 (s; C_{aryl}), 128.4 (s; C_{aryl}), 123.5 (s; C_{aryl}), 36.2 (s; C_{aliph}), 28.8 (s; C_{aliph}), 23.7 (s; C_{aliph}), 19.9 (s; C_{aliph}) ppm. MS (ESI) *m/z* (%): 248.20 $[M\text{-}H]^+$. Elemental analysis calcd for $C_{16}H_{25}NO$: C 77.68, H 10.19, N 5.66, found: C 77.54, H 9.82, N 6.01.

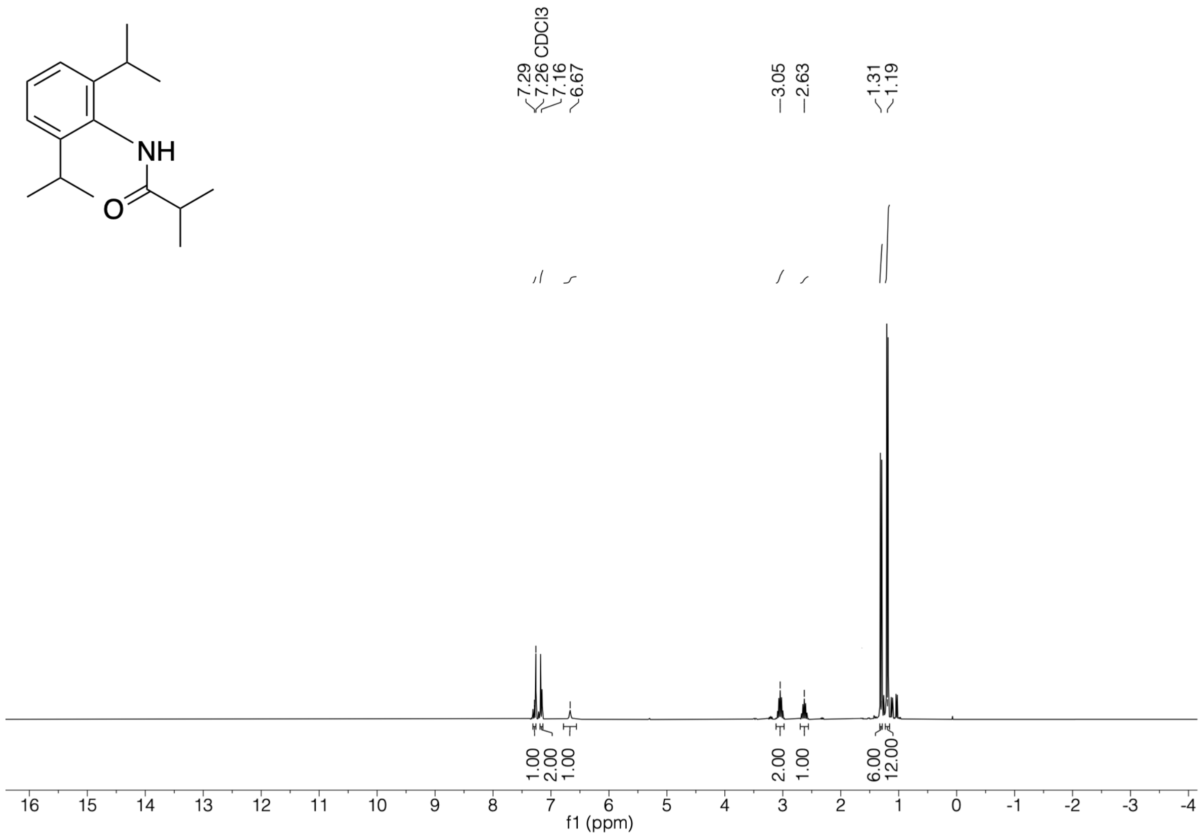

Figure SF76. ^{1}H NMR (300 MHz, $CDCl_3$, 298.0 K) spectrum of **Dipp-7-Me$_2$**.

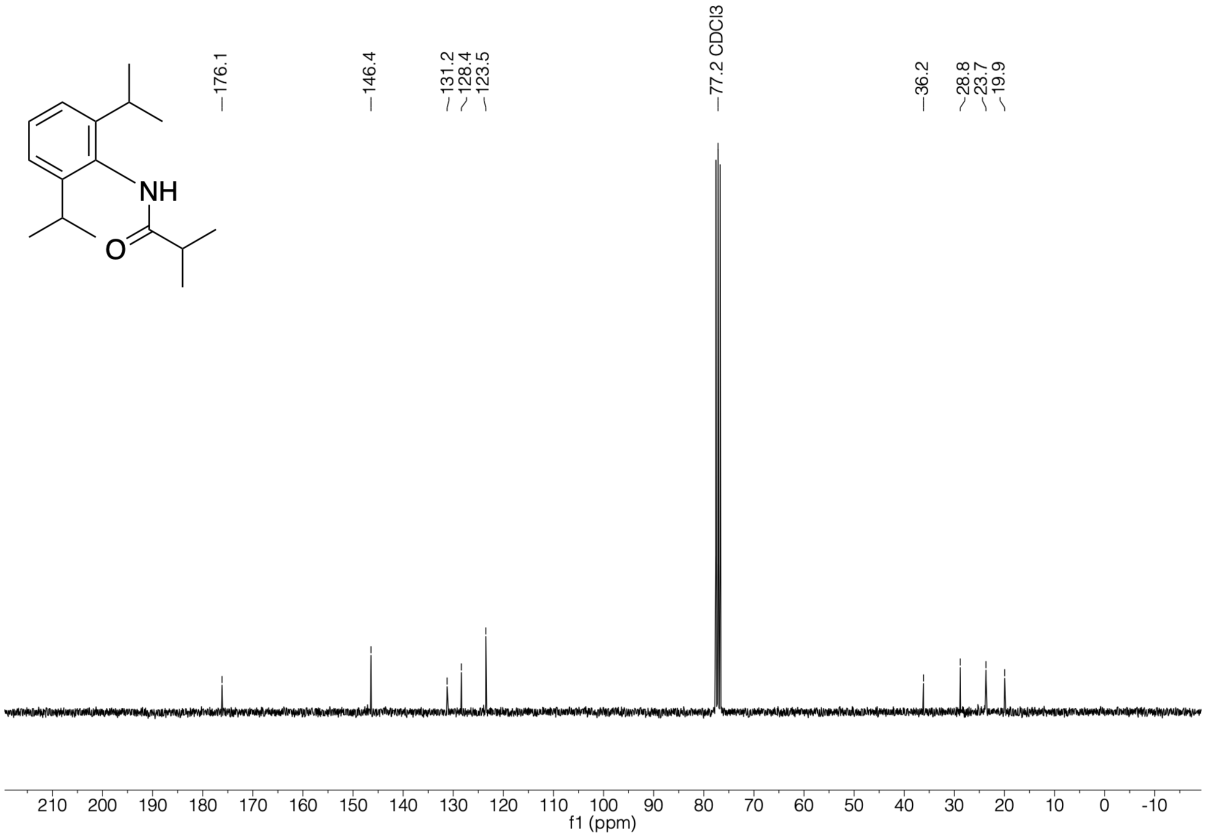

Figure SF77. $^{13}C\{^{1}H\}$ NMR (75 MHz, $CDCl_3$, 298.0 K) spectrum of **Dipp-7-Me$_2$**.

Dipp-10-Ph$_2$: ^{1}H NMR (300 MHz, $CDCl_3$, 298.0 K): δ = 8.11–7.92 (m, 4H; CH_{aryl}), 7.32–6.97 (m, 9H; CH_{aryl}), 3.11 (sept, J = 7 Hz, 2H; CH_{Dipp}), 2.63 (sept, J = 7 Hz, 1H; CH), 1.14 (d, J = 7 Hz, 12H; CH_3) ppm. $^{13}C\{^1H\}$ NMR (75 MHz, $CDCl_3$, 298.0 K): δ = 189.4 (s; C=N), 140.4 (s; C_{aryl}), 138.2 (s; C_{aryl}), 130.7 (s; C_{aryl}), 129.9 (s; C_{aryl}), 129.0 (s; C_{aryl}), 127.3 (s; C_{aryl}), 123.5 (s; C_{aryl}), 77.4 (s; C(*C*(Ph)$_2$)), 28.6 (s; C_{aliph}), 23.7 (s; C_{aliph}) ppm. Elemental analysis calcd for $C_{26}H_{27}N$: C 88.34, H 7.70, N 3.96, found: C 88.04, H 7.34, N 4.21.

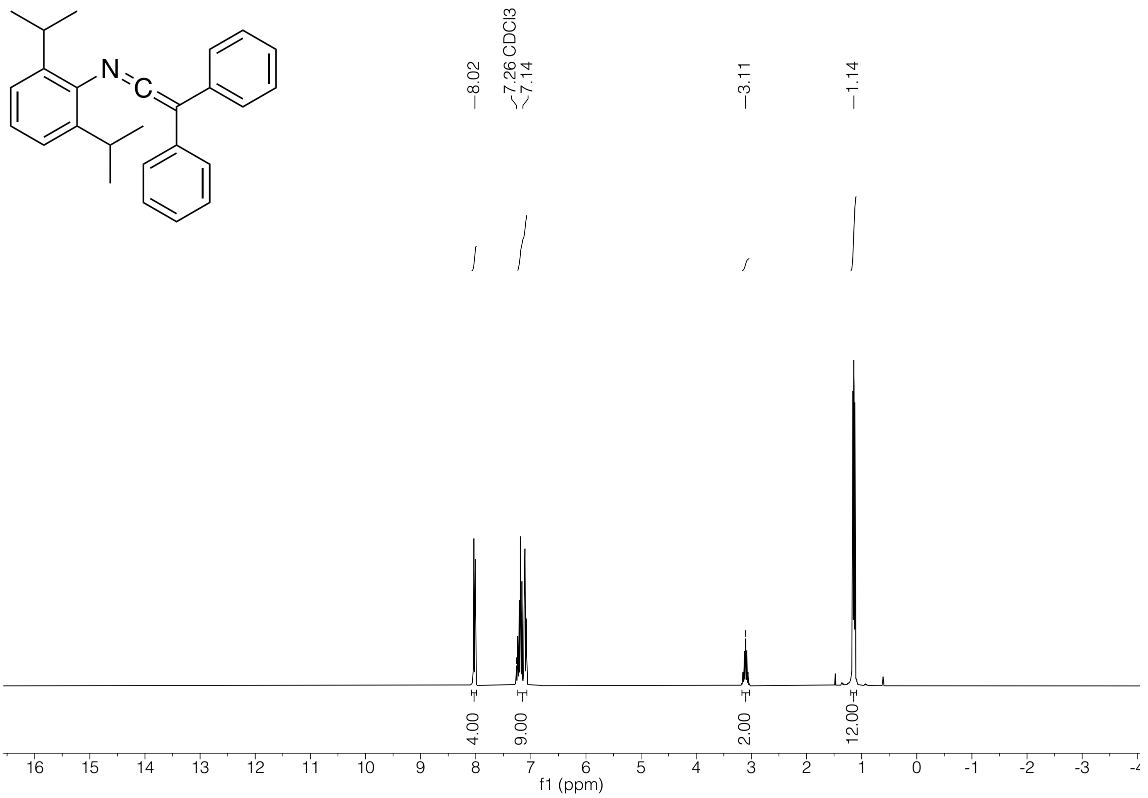

Figure SF78. ^{1}H NMR (300 MHz, $CDCl_3$, 298.0 K) spectrum of **Dipp-10-Ph$_2$**.

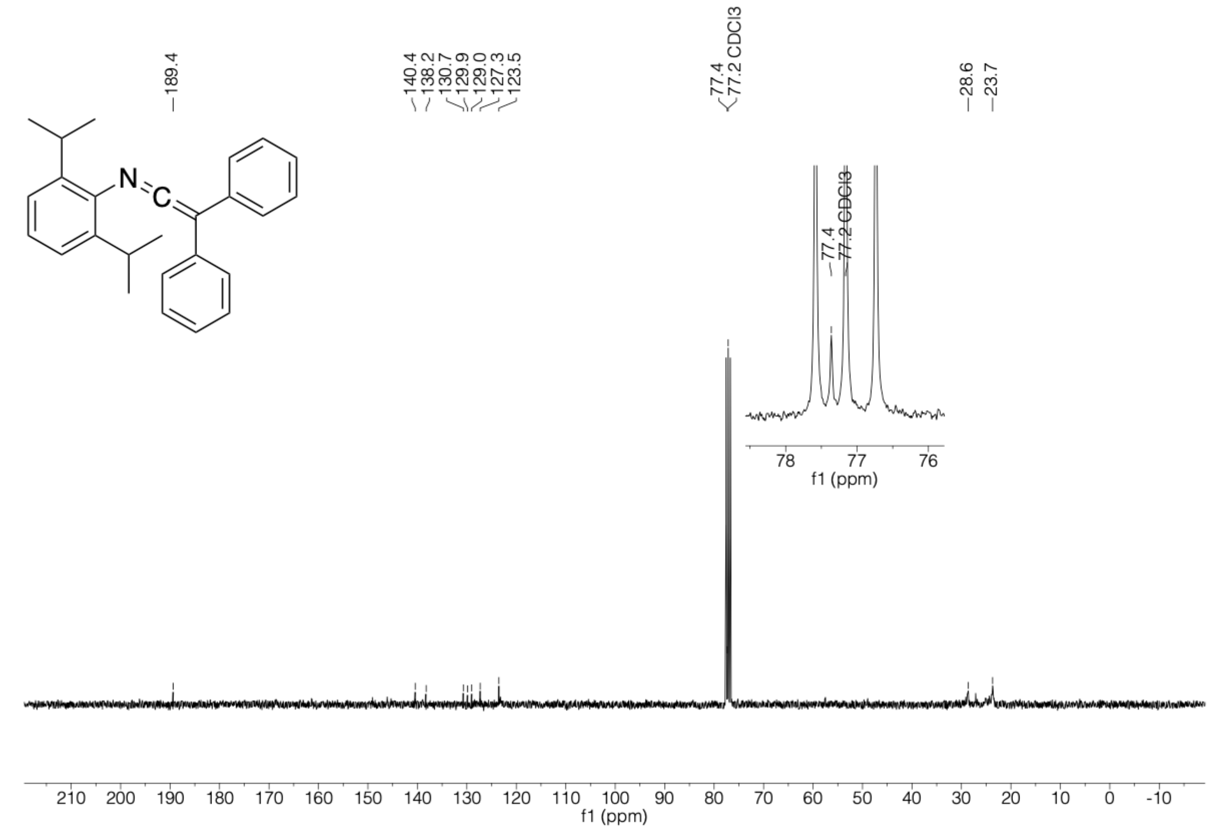

Figure SF79. $^{13}C\{^1H\}$ NMR (75 MHz, $CDCl_3$, 298.0 K) spectrum of **Dipp-10-Ph$_2$**.

Dipp-7-sCy: ^1H NMR (300 MHz, $CDCl_3$, 298.0 K): δ = 7.33–7.28 (m, 1H; CH_{para}), 7.19–7.13 (m, 2H; CH_{meta}), 7.05 (br s, 1H; NH), 2.91 (sept, J = 7 Hz, 2H; CH_{Dipp}), 2.23–2.19 (m, 2H; CH_{aliph}), 2.10–2.06 (m, 2H; CH_{aliph}), 1.78–1.72 (m, 3H; CH_{aliph}), 1.56–1.40 (m, 4H; CH_{aliph}), 1.17 (d, J = 7 Hz, 12H; CH_3) ppm. $^{13}C\{^1H\}$ NMR (75 MHz, $CDCl_3$, 298.0 K): δ = 175.5 (s; C=O), 136.8 (s; C_{aryl}), 135.3 (s; C_{aryl}), 131.3 (s; C_{aryl}), 128.9 (s; C_{aryl}), 36.0 (s; C_{aliph}), 21.05 (s; C_{aliph}), 21.05 (s; C_{aliph}), 20.0 (s; C_{aliph}), 18.32 (s; C_{aliph}), 18.29 (s; C_{aliph}) ppm. MS (ESI) m/z (%): 288.22 $[M-H]^+$. Elemental analysis calcd for $C_{19}H_{29}NO$: C 79.39, H 10.17, N 4.87, found: C 79.45, H 10.23, N 4.68.

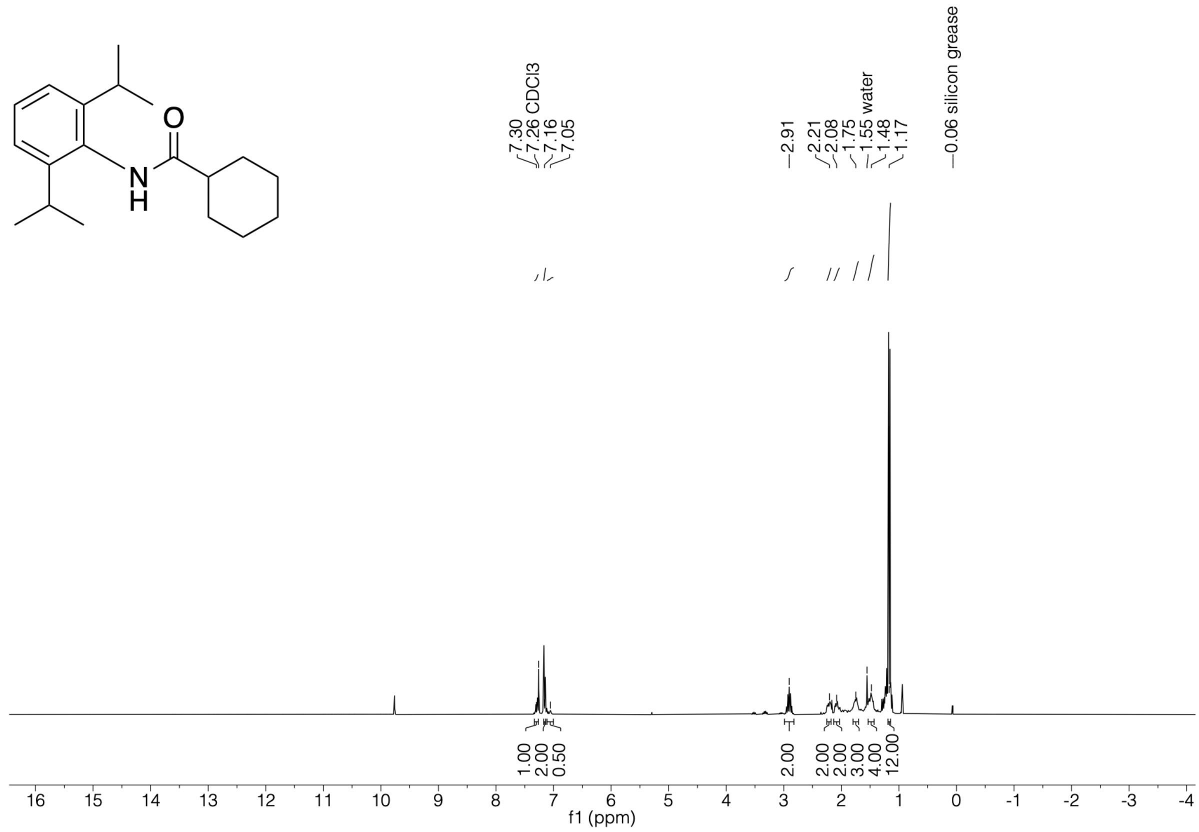

Figure SF80. ^{1}H NMR (300 MHz, $CDCl_3$, 298.0 K) spectrum of **Dipp-7-sCy**.

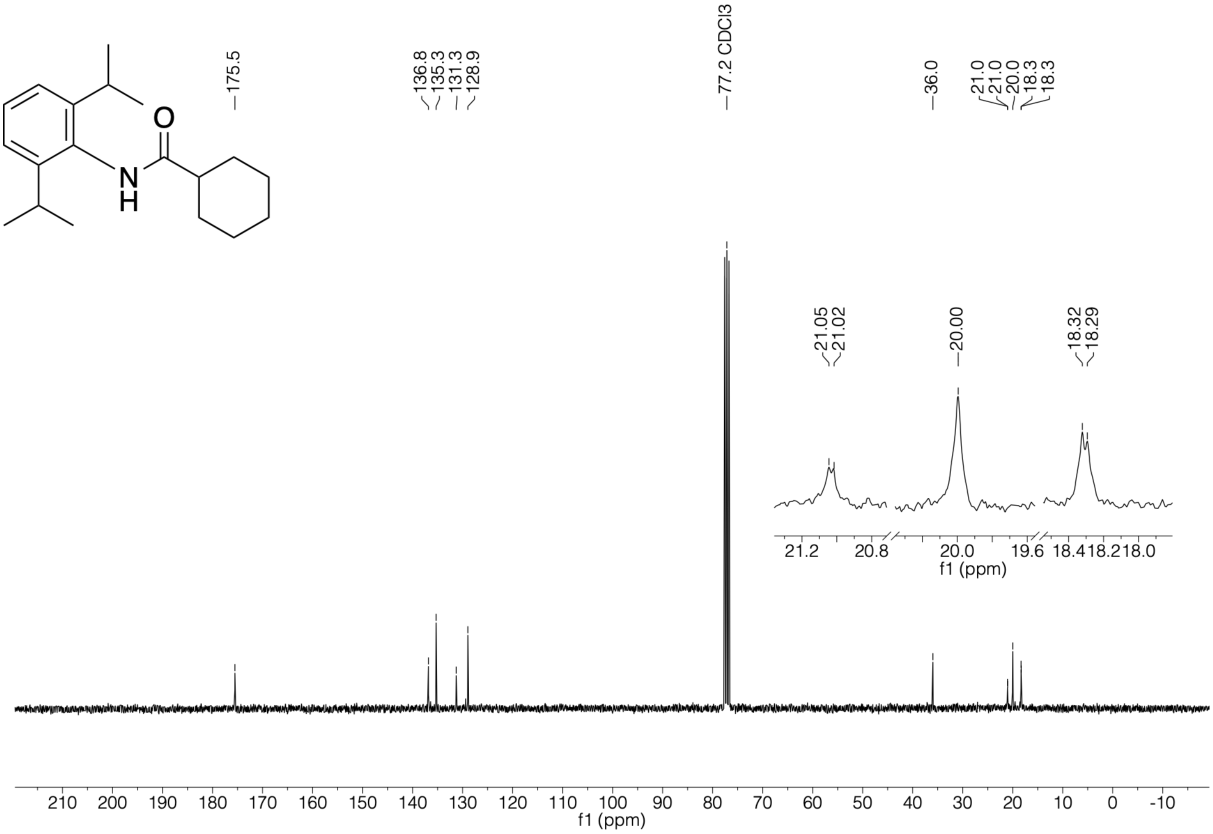

Figure SF81. $^{13}C\{^{1}H\}$ NMR (75 MHz, $CDCl_3$, 298.0 K) spectrum of **Dipp-7-sCy**.

As the amide **Dipp-7-sCy** is obtained in high yields after thermolysis of the precursor and workup and is supposed to be hydrolysis products of a ketenimine, which resulted from decomposition of the generated carbene under the concomitant release of carbon monoxide, it was tried to observe the ketenimine by NMR spectroscopy of a toluene-d8 solution in a sealed NMR tube after thermolysis was also performed at 110 °C for 16 h in that sealed NMR tube. In the NMR a mixture of different products was observed and it was impossible to clearly assign peaks of the NMR spectrum to the desired ketenimine. After adding D_2O and column chromatography (diethyl ether/*n*-hexane 1:5, silica), the deuterated amide was obtained.

Dipp-7-sCy-d$_2$: Precursor **Dipp-5-sCy** (100 mg, 0.26 mmol) was dissolved in 0.6 ml of toluene-d8 and heated in a sealed NMR tube at 105 °C for 16 h. After recording NMR spectra, 0.1 ml of D_2O was added, the desired product was isolated by column chromatography (diethyl ether/*n*-hexane 1:5, silica) and obtained as colorless, air-stable powder. Yield: 69 mg, 92%. ^{1}H NMR (300 MHz, $CDCl_3$, 298.0 K): δ = 7.30–7.27 (m, 1H; CH_{para}), 7.18–7.13 (m, 2H; CH_{meta}), 3.04 (sept, J = 7 Hz, 2H; CH_{Dipp}), 2.08–1.94 (m, 2H; CH_{aliph}), 1.92–1.83 (m, 2H; CH_{aliph}), 1.73–1.69 (m, 1H; CH_{aliph}), 1.59–1.52 (m, 2H; CH_{aliph}), 1.42–1.30 (m, 3H; CH_{aliph}), 1.19 (d, J = 7 Hz, 12H; CH_3) ppm. ^{13}C{^{1}H} NMR (75 MHz, $CDCl_3$, 298.0 K): δ = 175.4 (s; C=O), 146.4 (s; C_{aryl}), 131.1 (s; C_{aryl}), 128.4 (s; C_{aryl}), 123.5 (s; C_{aryl}), 30.0 (br s; C_{aliph}), 28.8 (s; C_{aliph}), 25.9 (br s; C_{aliph}), 23.7 (br s; C_{aliph}) ppm [Some of the expected signals for aliphatic carbon atoms are not observed due to isochrony as well as overlapping of the signals. Because of this, no CD-coupling and multiplicity is observed. MS-Data, CHNS-Analysis and SCXRD confirm the constitution of **Dipp-7-sCy-d$_2$**]. MS (ESI) *m/z* (%): 290.25 $[M\text{-}H]^+$. Elemental analysis calcd for $C_{19}H_{27}D_2NO$: C 78.84, H 10.79, N 4.84, found: C 78.61, H 10.57, N 4.64.

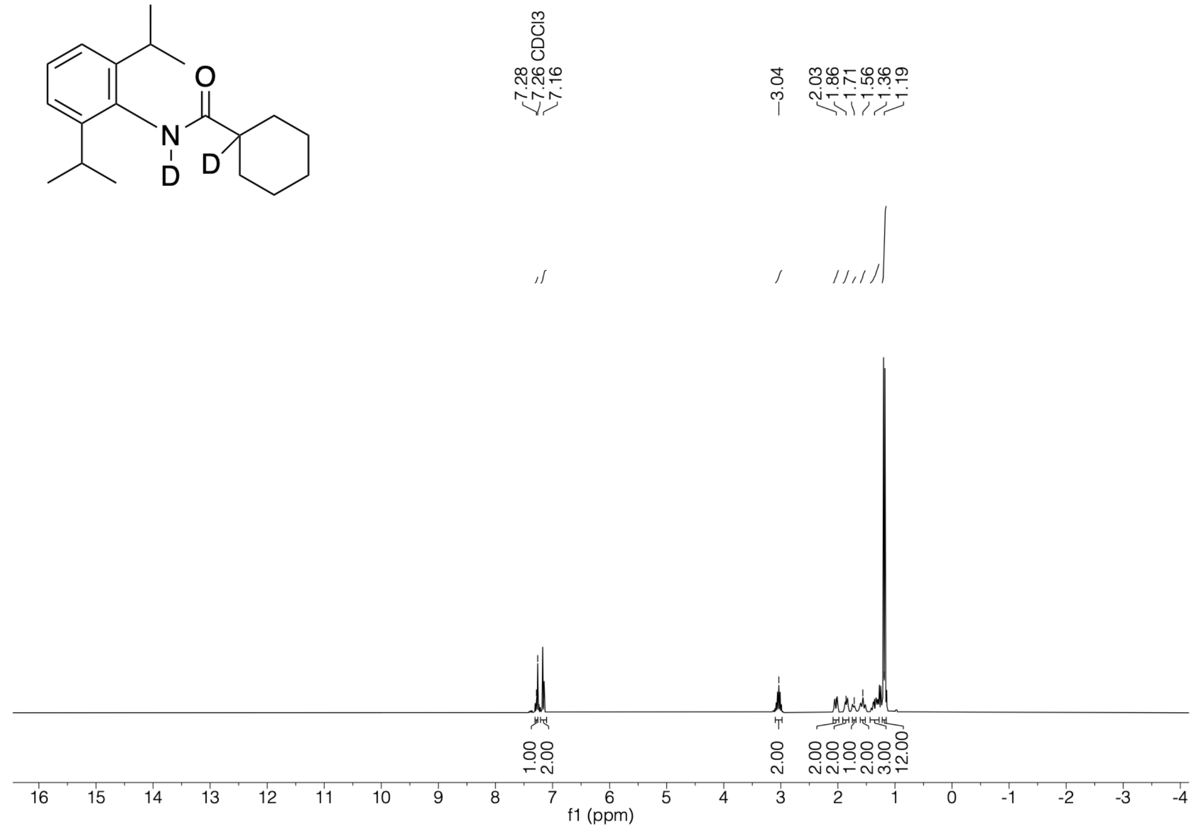

Figure SF82. ^{1}H NMR (300 MHz, $CDCl_3$, 298.0 K) spectrum of **Dipp-7-sCy-d$_2$**.

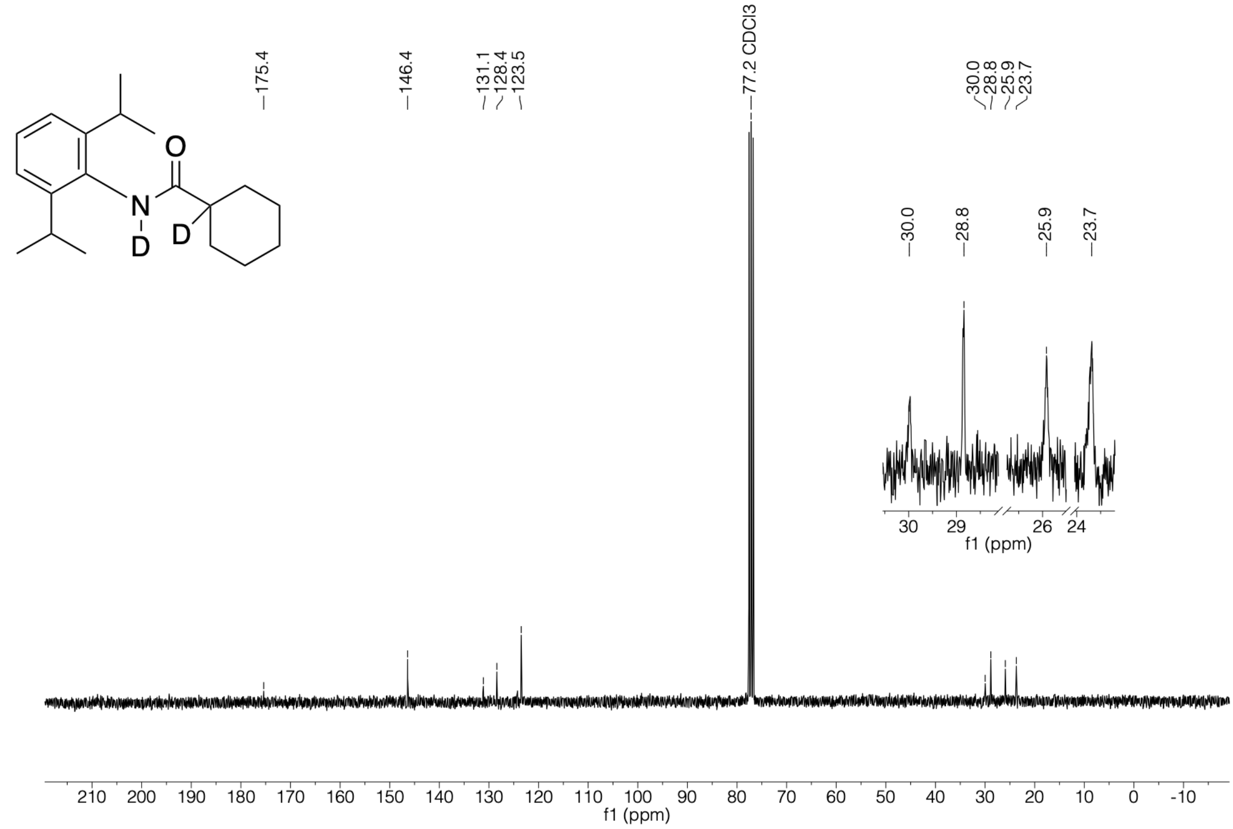

Figure SF83. $^{13}C\{^{1}H\}$ NMR (75 MHz, $CDCl_3$, 298.0 K) spectrum of **Dipp-7-sCy-d$_2$**.

Thermolysis of two different precursors bearing substituents with different steric demands showed a selective reaction of the precursors. The reaction of precursor **Dipp-5-sCy** and **Ph-5-Me2** yield selectively the known thermolysis products of the precursors and not a cross product. **Dipp-5-sCy** (192 mg, 0.5 mmol, 1 eq.) and **Ph-5-Me2** (130 mg, 0.5 mmol, 1 eq.) were dissolved in 2 ml toluene and heated to 110 °C for 16 h. The products were separated by column chromatography (diethyl ether/*n*-hexane 1:5, silica) and obtained as colorless, air-stable powders. Products obtained are **Dipp-7-sCy** (134 mg, 93%), **[Ph-6-Me$_2$]$_2$** (28 mg, 32%), **[Ph-6-Me$_2$*N]$_2$** (52 mg, 56%) and **Ph-7-Me$_2$** (6 mg, 7%). The yields are given based on the corresponding precursors as the reaction was highly selective.

g. Synthesis of precursors 6*HCl

The corresponding 1,3,4-oxadiazole-based precursor **5** (5 mmol, 1 eq.) was dissolved in a mixture of 3 ml of toluene and 5 ml of HCl in 1,4-dioxane (4 M: 20 mmol HCl, 4 eq. of HCl) at ambient temperature. After heating at 110 °C for 16 h, the solvent was removed in vacuo to give a colorless powder.

Ph-6-Me$_2$*HCl: 1,3,4-oxadiazole-based precursor used: **Ph-5-Me$_2$** (1.30 g, 5 mmol). Colorless, air-stable powder. Yield: 1.04 g, 99%. ^{1}H NMR (300 MHz, C_6D_6, 298.0 K): δ = 7.63–7.53 (m, 2H; $CH_{aryl\text{-}ortho}$), 7.11–7.05 (m, 2H; $CH_{aryl\text{-}meta}$), 6.91–6.82 (m, 1H; $CH_{aryl\text{-}para}$), 5.06 (s, 1H; NC(H)(Cl)C), 1.17 (s, 3H; $C(C(CH_3)_2)C$), 0.87 (s, 3H; $C(C(CH_3)_2)C$) ppm. $^{13}C\{^1H\}$ NMR (75 MHz, C_6D_6, 298.0 K): δ = 167.9 (s; C=O), 136.9 (s; NC_{aryl}), 129.4 (s; $C_{aryl\text{-}meta}$), 124.8 (s; $C_{aryl\text{-}para}$), 117.5 (s; $C_{aryl\text{-}ortho}$), 75.6 (s; N*C*(H)(Cl)C), 57.5 (s; C(*C*(CH$_3$)$_2$)C), 20.6 (s; C(C(*C*H$_3$)$_2$)C), 18.3 (s; C(C(*C*H$_3$)$_2$)C) ppm [Some of the expected signals for aryl carbon atoms are not observed due to isochrony as well as overlapping with the solvent signal.]. MS (EI, 70 eV, 50 °C) *m/z* (%): 209 (100) $[M]^{+\cdot}$, 146 (29), 119 (72), 104 (55), 90 (11), 77 (27). Elemental analysis calcd for $C_{11}H_{12}ClNO$: C 63.01, H 5.77, N 6.68, found: C 62.87, H 5.63, N 6.72.

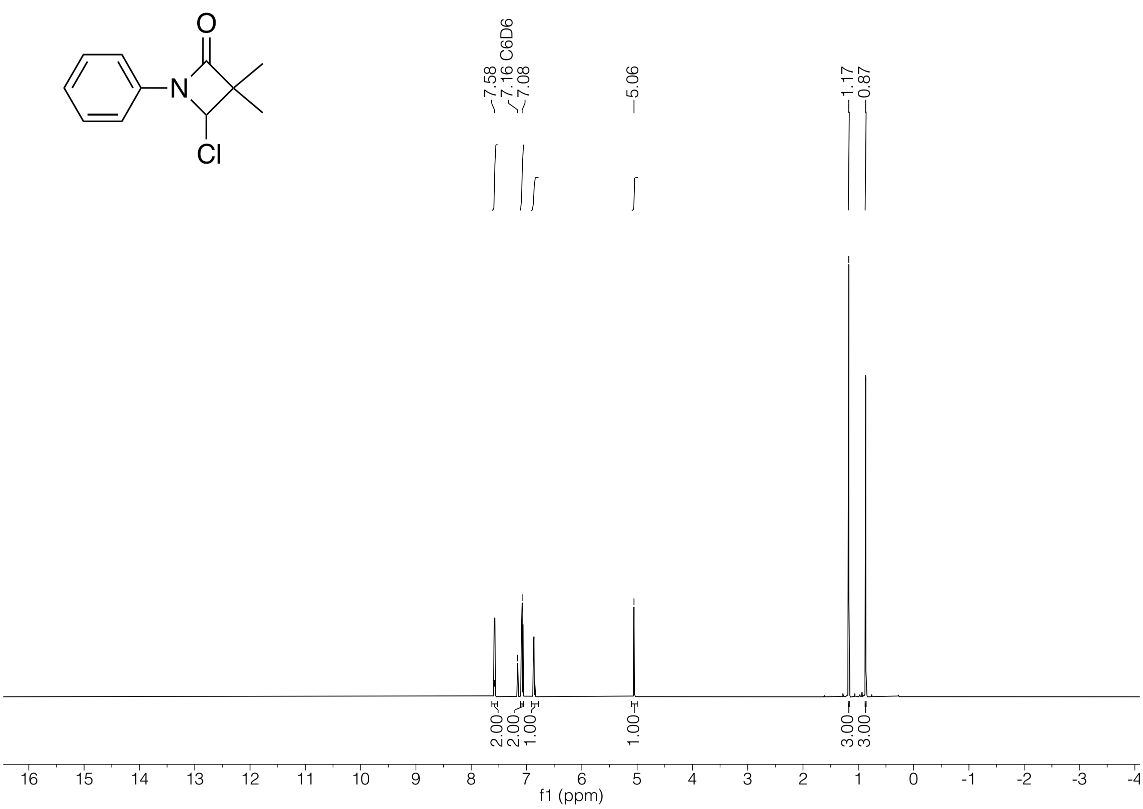

Figure SF84. ^{1}H NMR (300 MHz, C_6D_6, 298.0 K) spectrum of **Ph-6-Me$_2$*HCl**.

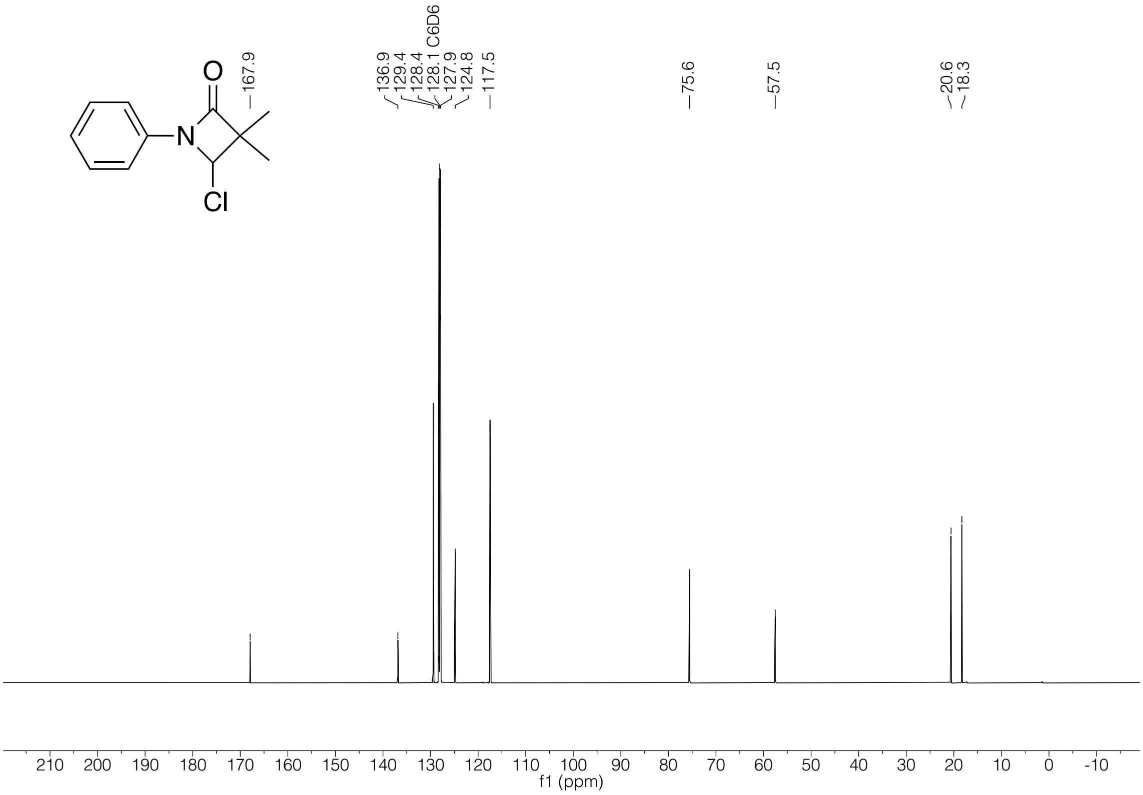

Figure SF85. ^{13}C{^{1}H} NMR (75 MHz, C_6D_6, 298.0 K) spectrum of **Ph-6-Me$_2$*HCl**.

Ph-6-Cl₂*HCl: 1,3,4-oxadiazole-based precursor used: **Ph-5-Cl₂** (1.50 g, 5 mmol). Colorless, air-stable powder. Yield: 1.24 g, 99%. ^{1}H NMR (300 MHz, $CDCl_3$, 298.0 K): δ = 7.58–7.49 (m, 2H; $CH_{aryl\text{-}ortho}$), 7.46–7.40 (m, 2H; $CH_{aryl\text{-}meta}$), 7.33–7.28 (m, 1H; $CH_{aryl\text{-}para}$), 6.19 (s, 1H; NC(H)(Cl)C) ppm. ^{13}C{^{1}H} NMR (75 MHz, $CDCl_3$, 298.0 K): δ = 155.9 (s; C=O), 134.2 (s; NC_{aryl}), 129.7 (s; $C_{aryl\text{-}meta}$), 126.9 (s; $C_{aryl\text{-}para}$), 118.3 (s; $C_{aryl\text{-}ortho}$), 84.5 (s; C(*C*(Cl)$_2$)C), 78.5 (s; N*C*(H)(Cl)C) ppm [Some of the expected signals for aryl carbon atoms are not observed due to isochrony.]. MS (EI, 70 eV, 60 °C) *m/z* (%): 249 (80) $[M]^{+\cdot}$, 119 (100), 90 (21), 77 (35). Elemental analysis calcd for $C_9H_6Cl_3NO$: C 43.15, H 2.41, N 5.59, found: C 42.94, H 2.58, N 5.41.

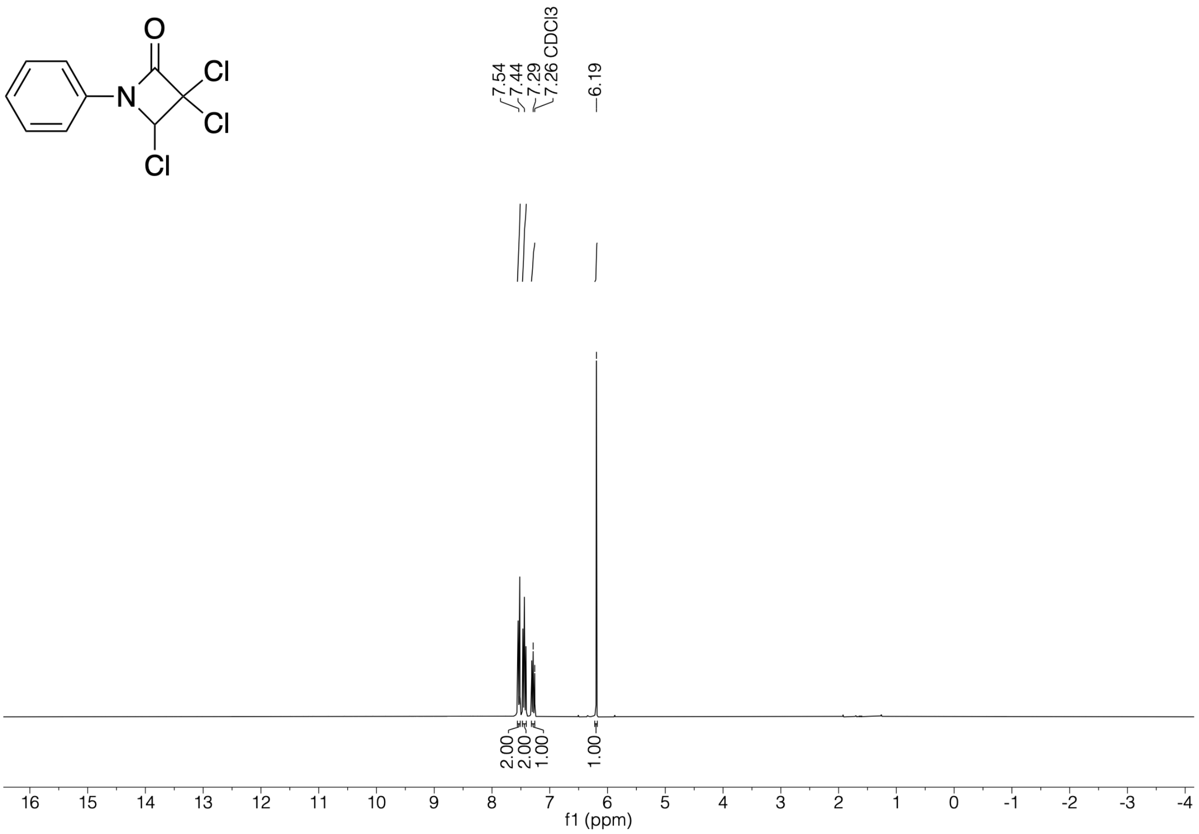

Figure SF86. ^{1}H NMR (300 MHz, $CDCl_3$, 298.0 K) spectrum of **Ph-6-Cl₂*HCl**.

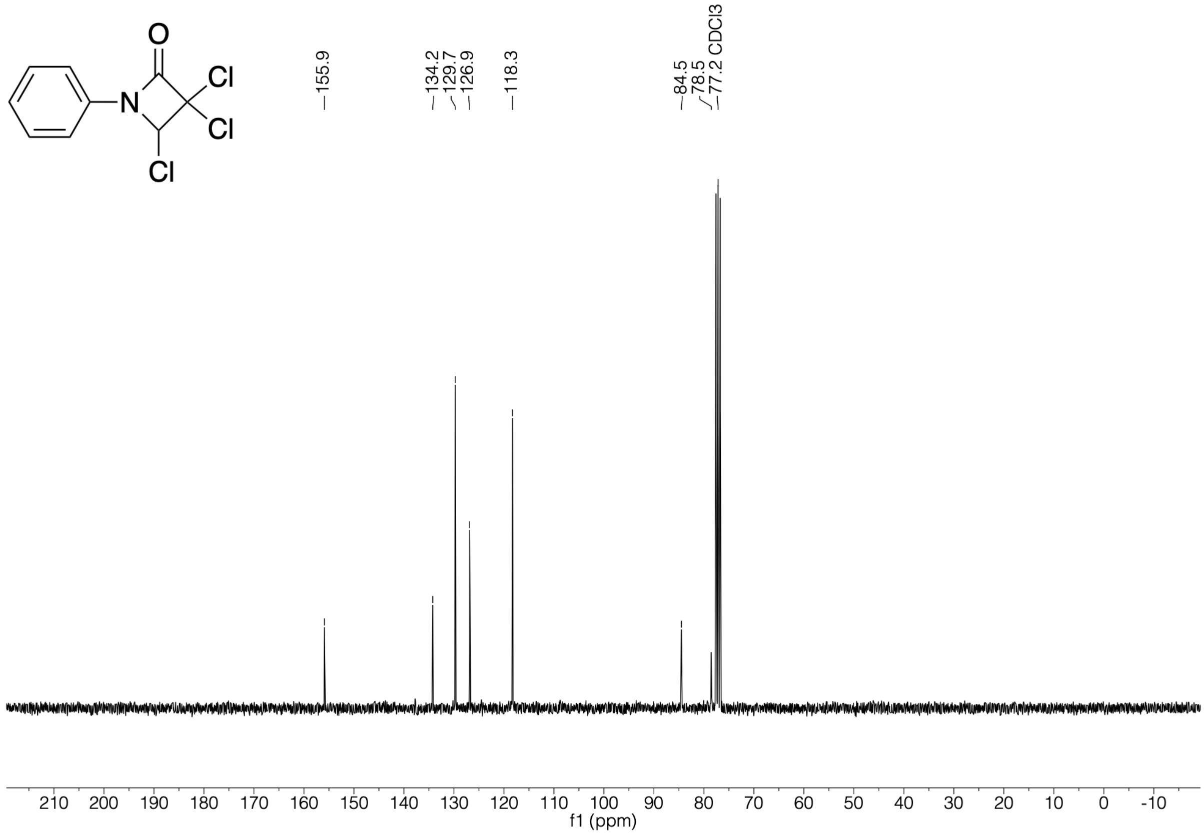

Figure SF87. $^{13}C\{^1H\}$ NMR (75 MHz, $CDCl_3$, 298.0 K) spectrum of **Ph-6-Cl$_2$*HCl**.

Ph-6-Ph$_2$*HCl: 1,3,4-oxadiazole-based precursor used: **Ph-5-Ph$_2$** (1.92 g, 5 mmol). Colorless, air-stable powder. Yield: 1.65 g, 99%. ^{1}H NMR (300 MHz, C_6D_6, 298.0 K): δ = 7.66–7.63 (m, 2H; CH_{aryl}), 7.47–7.42 (m, 4H; CH_{aryl}), 7.10–6.98 (m, 8H; CH_{aryl}), 6.88–6.84 (m, 1H; CH_{aryl}), 6.19 (s, 1H; NC(H)(Cl)C) ppm. $^{13}C\{^1H\}$ NMR (75 MHz, C_6D_6, 298.0 K): δ = 164.6 (s; C=O), 138.5 (s; NC_{aryl}), 137.3 (s; C-C_{aryl}), 137.3 (s; C-C_{aryl}), 136.5 (s; C_{aryl}), 129.5 (s; C_{aryl}), 129.4 (s; C_{aryl}), 129.2 (s; C_{aryl}), 128.6 (s; C_{aryl}), 128.4 (s; C_{aryl}), 128.3 (s; C_{aryl}), 128.2 (s; C_{aryl}), 127.8 (s; C_{aryl}), 125.2 (s; C_{aryl}), 117.9 (s; C_{aryl}), 75.1 (s; N*C*(H)(Cl)C), 74.3 (s; C(*C*(Ph)$_2$)C) ppm [Some of the expected signals for aryl carbon atoms are not observed due to isochrony as well as overlapping with the solvent signal.]. MS (EI, 70 eV, 50 °C) *m/z* (%): 334 (4) $[M]^{+\cdot}$, 297 (13), 270 (11), 214 (100), 194 (17), 179 (36), 174 (6), 164 (8), 77 (8). Elemental analysis calcd for $C_{21}H_{16}ClNO$: C 75.56, H 4.83, N 4.20, found: C 75.81, H 4.94, N 4.38.

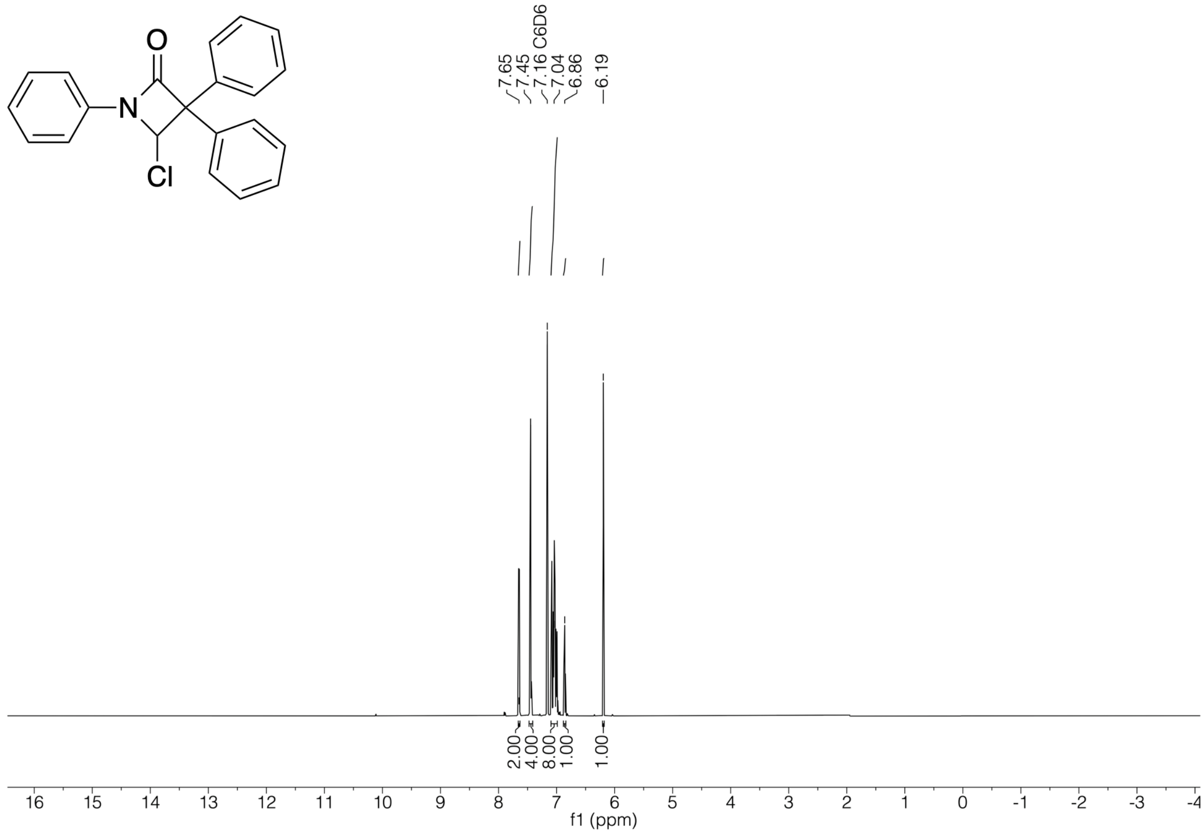

Figure SF88. ^{1}H NMR (300 MHz, C_6D_6, 298.0 K) spectrum of **Ph-6-Ph$_2$*HCl**.

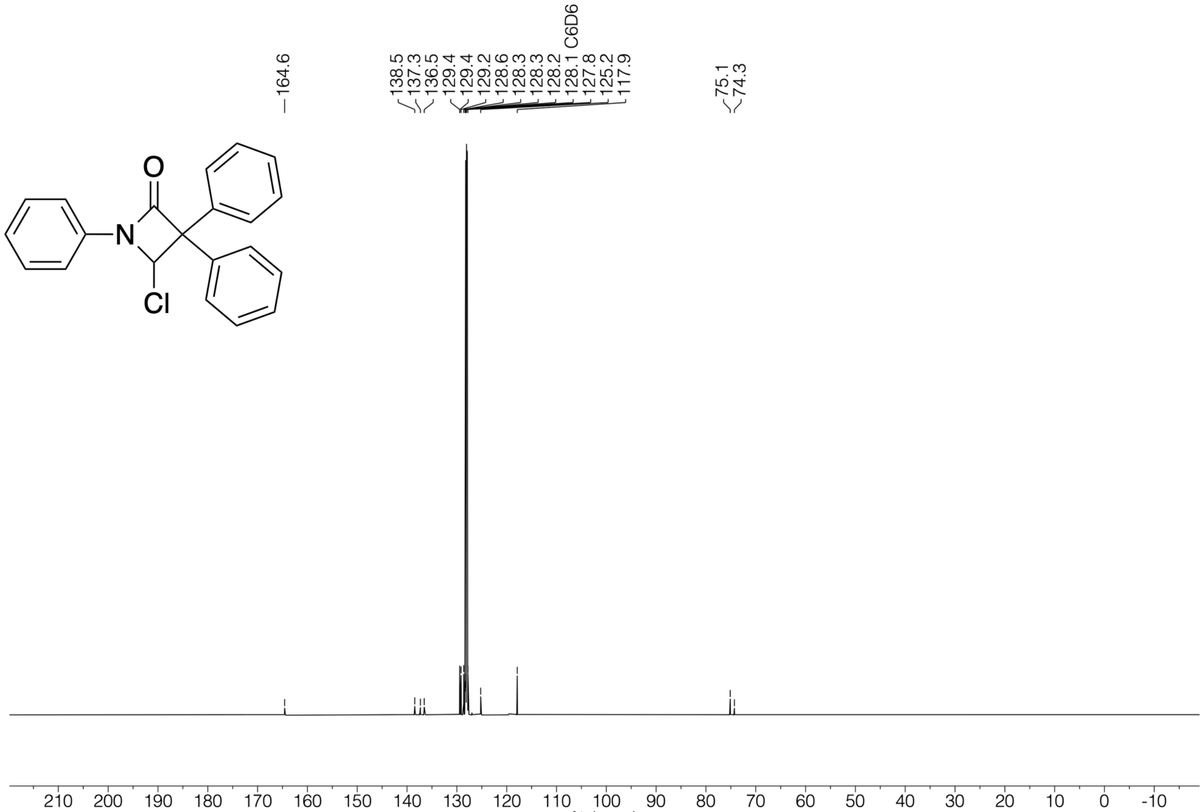

Figure SF89. ^{13}C{^{1}H} NMR (75 MHz, C_6D_6, 298.0 K) spectrum of **Ph-6-Ph$_2$*HCl**.

Mes-6-Me$_2$*HCl: 1,3,4-oxadiazole-based precursor used: **Mes-5-Me$_2$** (1.51 g, 5 mmol). Colorless, air-stable powder. Yield: 1.25 g, 99%. ^{1}H NMR (300 MHz, C_6D_6, 298.0 K): δ = 6.69–6.54 (m, 2H; CH_{meta}), 5.40 (s, 1H; NC(H)(Cl)C), 2.03 (s, 3H; $CH_{3\text{-}ortho}$), 1.51 (s, 3H; $CH_{3\text{-}ortho}$), 1.36 (s, 3H; $CH_{3\text{-}para}$), 1.29 (s, 3H; C(C(CH_3)$_2$)C), 1.09 (s, 3H; C(C(CH_3)$_2$)C) ppm. ^{13}C{^{1}H} NMR (75 MHz, C_6D_6, 298.0 K): δ = 169.2 (s; C=O), 138.5 (s; NC_{aryl}), 129.7 (s; $C_{aryl\text{-}meta}$), 129.2 (s; $C_{aryl\text{-}meta}$), 128.9 (s; $C_{aryl\text{-}ortho}$), 128.4 (s; $C_{aryl\text{-}ortho}$), 120.5 (s; $C_{aryl\text{-}para}$), 80.0 (s; N*C*(H)(Cl)C), 57.5 (s; C(*C*(CH_3)$_2$)C), 24.8 (s; $CH_{3\text{-}ortho}$), 24.4 (s; $CH_{3\text{-}ortho}$), 21.2 (s; $CH_{3\text{-}para}$), 20.9 (s; C(C(*C*H_3)$_2$)C), 19.0 (s; C(C(*C*H_3)$_2$)C) ppm [Some of the expected signals for aryl carbon atoms are not observed due to isochrony as well as overlapping with the solvent signal.]. MS (EI, 70 eV, 60 °C) *m/z* (%): 251 (83) $[M]^{+\cdot}$, 194 (100), 119 (31), 77 (23). Elemental analysis calcd for $C_{14}H_{18}ClNO$: C 66.79, H 7.21, N 5.56, found: C 68.13, H 7.37, N 5.39.

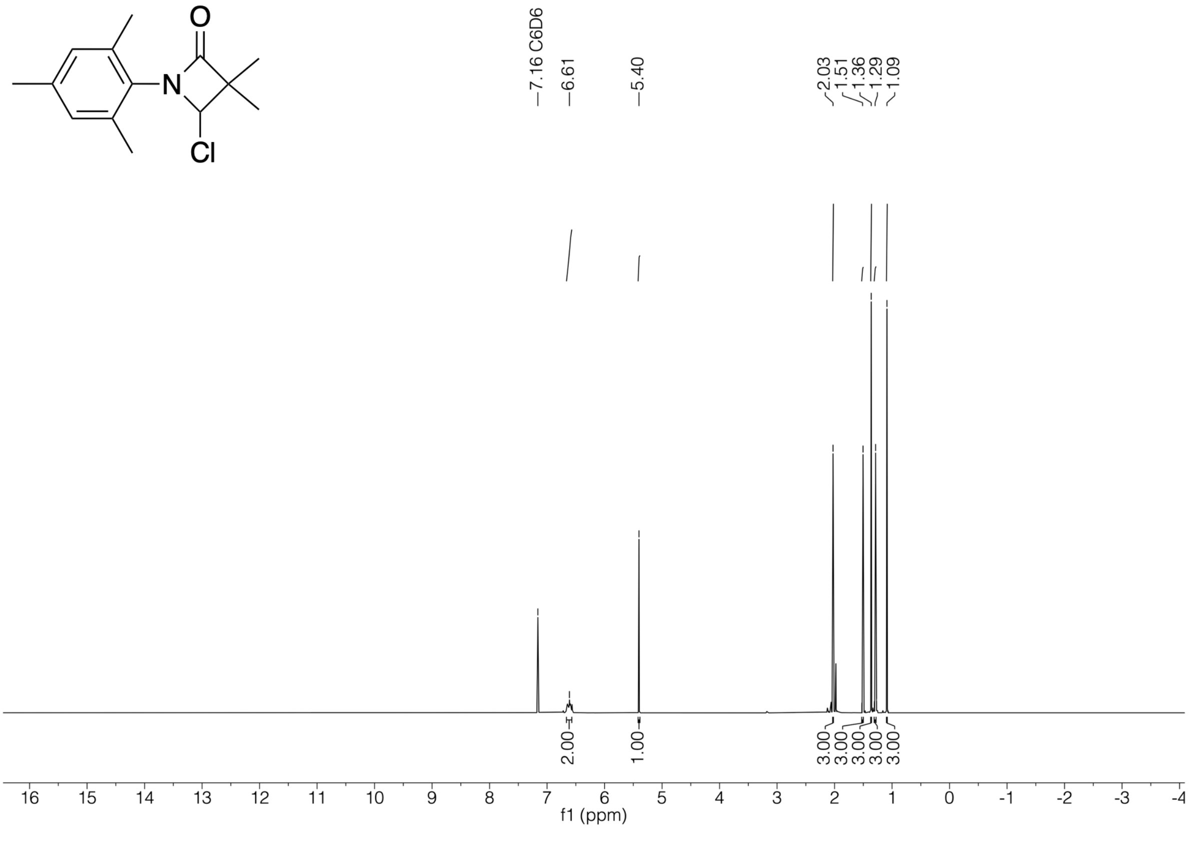

Figure SF90. ^{1}H NMR (300 MHz, C_6D_6, 298.0 K) spectrum of **Mes-6-Me$_2$*HCl**.

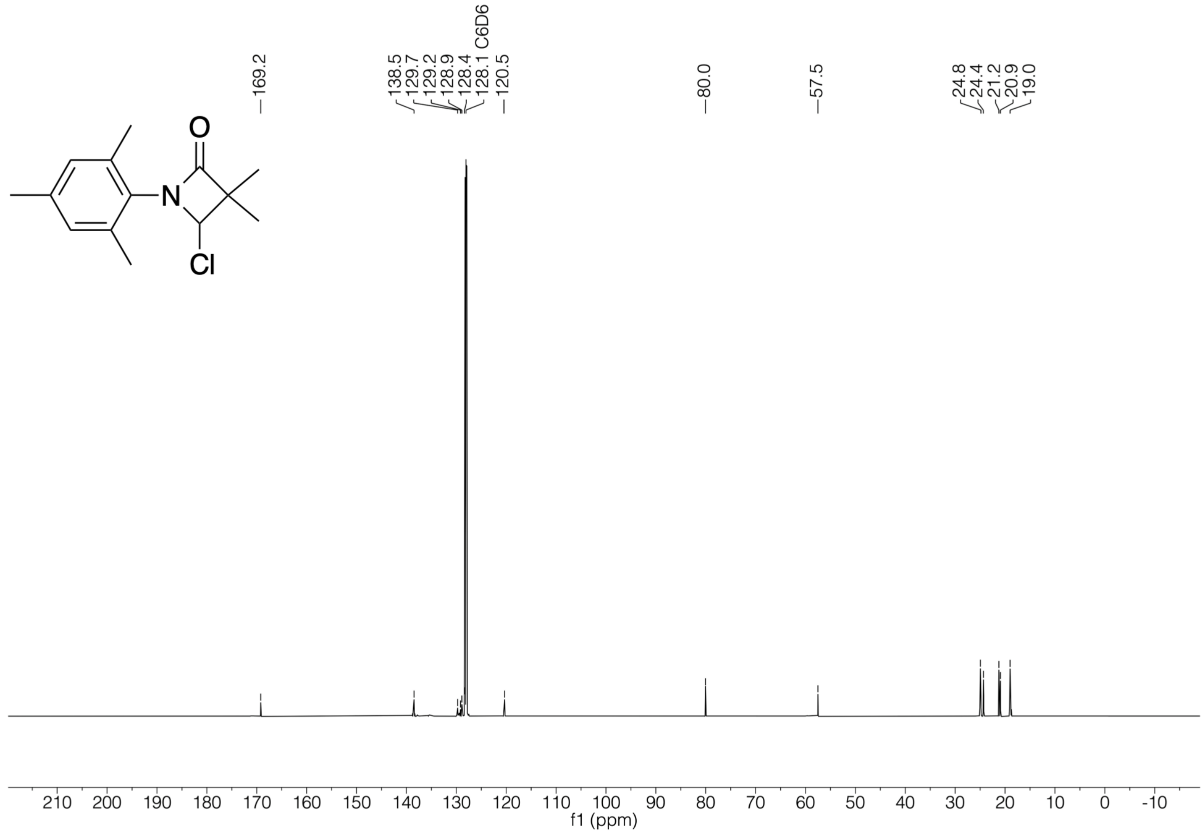

Figure SF91. $^{13}C\{^1H\}$ NMR (75 MHz, C_6D_6, 298.0 K) spectrum of **Mes-6-Me$_2$*HCl**.

Mes-6-Ph$_2$*HCl: 1,3,4-oxadiazole-based precursor used: **Mes-5-Ph$_2$** (2.13 g, 5 mmol). Colorless, air-stable powder. Yield: 1.86 g, 99%. ^1H NMR (300 MHz, C_6D_6, 298.0 K): δ = 7.69–7.65 (m, 2H; CH_{aryl}), 7.58–7.54 (m, 2H; CH_{aryl}), 7.21–7.17 (m, 2H; CH_{aryl}), 7.09–7.05 (m, 3H; CH_{aryl}), 7.02–6.99 (m, 1H; CH_{aryl}), 6.68–6.61 (m, 1H; CH_{aryl}), 6.56 (s, 1H; NC(H)(Cl)C), 6.51–6.48 (m, 1H; CH_{aryl}), 2.52 (s, 3H; $CH_{3\text{-}ortho}$), 2.00 (s, 3H; $CH_{3\text{-}ortho}$), 1.76 (s, 3H; $CH_{3\text{-}para}$) ppm. $^{13}C\{^1H\}$ NMR (75 MHz, C_6D_6, 298.0 K): δ = 166.4 (s; C=O), 139.6 (s; NC_{aryl}), 139.0 (s; C-C_{aryl}), 138.4 (s; C-C_{aryl}), 137.7 (s; C_{aryl}), 136.0 (s; C_{aryl}), 129.9 (s; C_{aryl}), 129.3 (s; C_{aryl}), 129.2 (s; C_{aryl}), 129.1 (s; C_{aryl}), 128.8 (s; C_{aryl}), 128.4 (s; C_{aryl}), 128.2 (s; C_{aryl}), 127.6 (s; C_{aryl}), 127.1 (s; C_{aryl}), 79.2 (s; N*C*(H)(Cl)C), 74.5 (s; C(*C*(Ph)$_2$)C), 27.2 (s; $CH_{3\text{-}para}$), 20.9 (s; $CH_{3\text{-}ortho}$), 19.4 (s; $CH_{3\text{-}ortho}$) ppm [Some of the expected signals for aryl carbon atoms are not observed due to isochrony as well as overlapping with the solvent signal.]. MS (EI, 70 eV, 60 °C) *m/z* (%): 375 (4) $[M]^{+\cdot}$, 339 (27), 214 (100), 161 (75), 146 (19). Elemental analysis calcd for $C_{24}H_{22}ClNO$: C 76.69, H 5.90, N 3.73, found: C 76.82, H 6.11, N 3.97.

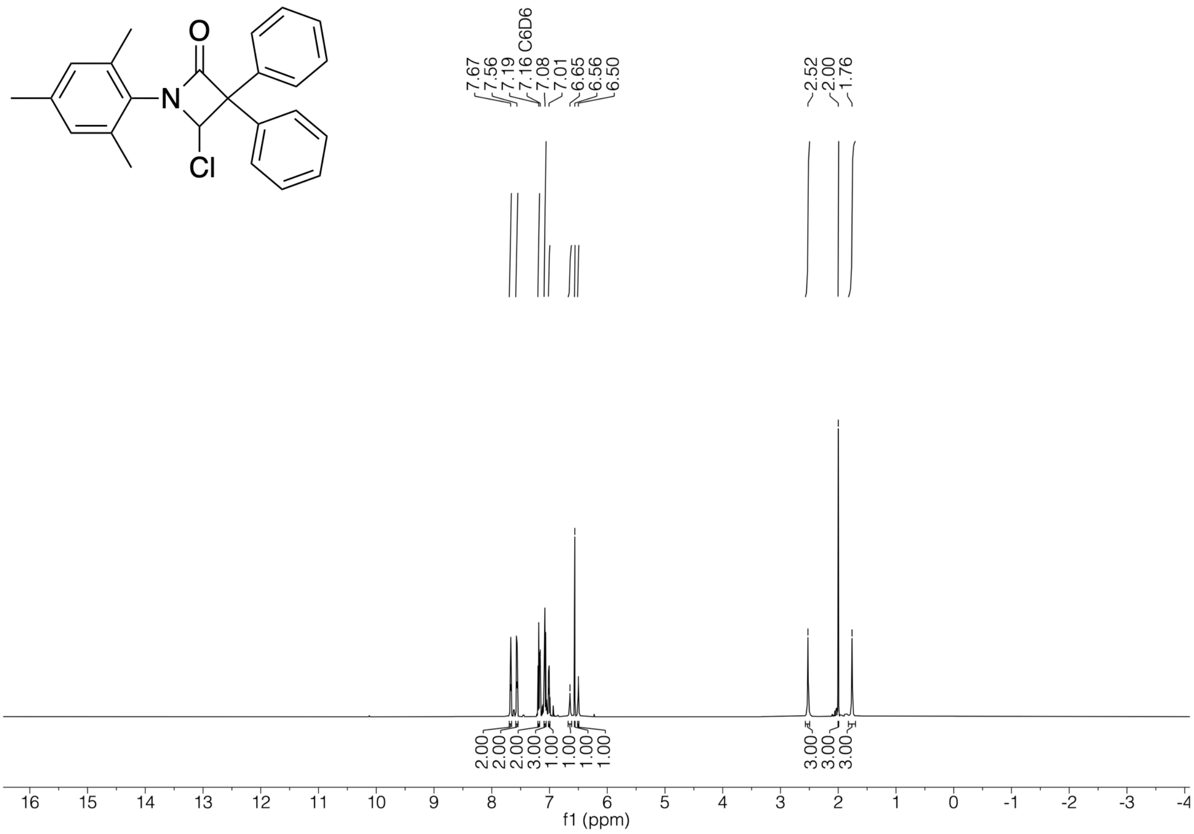

Figure SF92. ^{1}H NMR (300 MHz, C_6D_6, 298.0 K) spectrum of **Mes-6-Ph₂*HCl**.

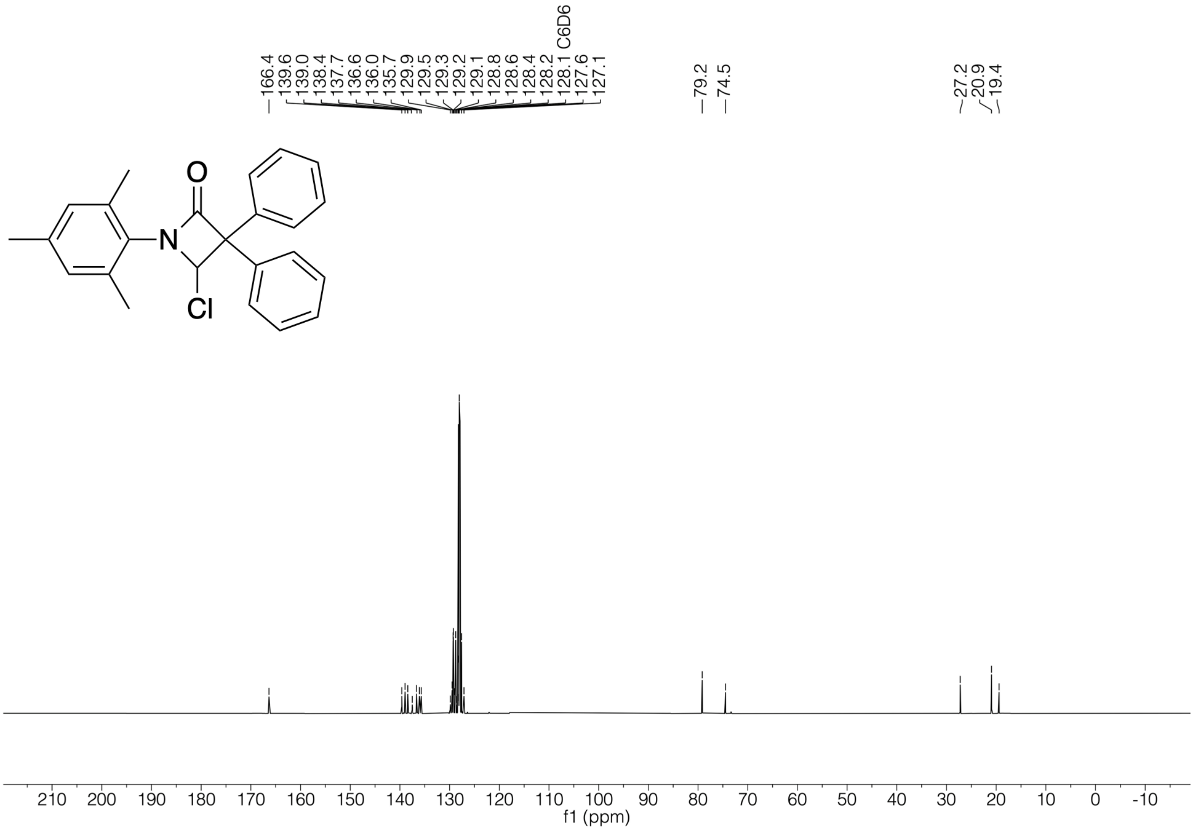

Figure SF93. $^{13}C\{^{1}H\}$ NMR (75 MHz, C_6D_6, 298.0 K) spectrum of **Mes-6-Ph$_2$*HCl**.

Dipp-6-Me$_2$*HCl: 1,3,4-oxadiazole-based precursor used: **Dipp-5-Me$_2$** (1.72 g, 5 mmol). Colorless, air-stable powder. Yield: 1.45 g, 99%. ^{1}H NMR (300 MHz, C_6D_6, 298.0 K): δ = 7.16–7.14 (m, 1H; $CH_{aryl\text{-}para}$), 7.10–7.08 (m, 1H; $CH_{aryl\text{-}meta}$), 6.98–6.95 (m, 1H; $CH_{aryl\text{-}meta}$), 5.46 (s, 1H; NC(H)(Cl)C), 3.52 (sept, *J* = 7 Hz, 1H; CH_{iPr}), 2.89 (sept, *J* = 7 Hz, 1H; CH_{iPr}), 1.37 (s, 3H; C(C(CH_3)$_2$)C), 1.34 (d, *J* = 7 Hz, 3H; $CH_{3\text{-}iPr}$), 1.31 (d, *J* = 7 Hz, 3H; $CH_{3\text{-}iPr}$), 1.12 (s, 3H; C(C(CH_3)$_2$)C), 1.08 (d, *J* = 7 Hz, 3H; $CH_{3\text{-}iPr}$), 1.07 (d, *J* = 7 Hz, 3H; $CH_{3\text{-}iPr}$) ppm. ^{13}C{^{1}H} NMR (75 MHz, C_6D_6, 298.0 K): δ = 170.4 (s; C=O), 149.5 (s; $C_{aryl\text{-}ortho}$), 147.2 (s; $C_{aryl\text{-}ortho}$), 130.1 (s; $C_{aryl\text{-}para}$), 128.4 (s; NC_{aryl}), 124.7 (s; $C_{aryl\text{-}meta}$), 123.8 (s; $C_{aryl\text{-}meta}$), 81.6 (s; N*C*(H)(Cl)C), 57.7 (s; C(*C*(CH_3)$_2$)C), 29.3 (s; CH_{iPr}), 29.2 (s; CH_{iPr}), 25.1 (s; $CH_{3\text{-}iPr}$), 24.3 (s; $CH_{3\text{-}iPr}$), 23.9 (s; $CH_{3\text{-}iPr}$), 23.5 (s; $CH_{3\text{-}iPr}$), 21.0 (s; C(C(*C*H_3)$_2$)C), 19.0 (s; C(C(*C*H_3)$_2$)C) ppm. MS (EI, 70 eV, 70 °C) *m/z* (%): 293 (13) $[M]^{+\cdot}$, 258 (17), 203 (98), 188 (100), 172 (13), 160 (14), 146 (25). Elemental analysis calcd for $C_{17}H_{24}ClNO$: C 69.49, H 8.23, N 4.77, found: C 69.23, H 7.94, N 4.66.

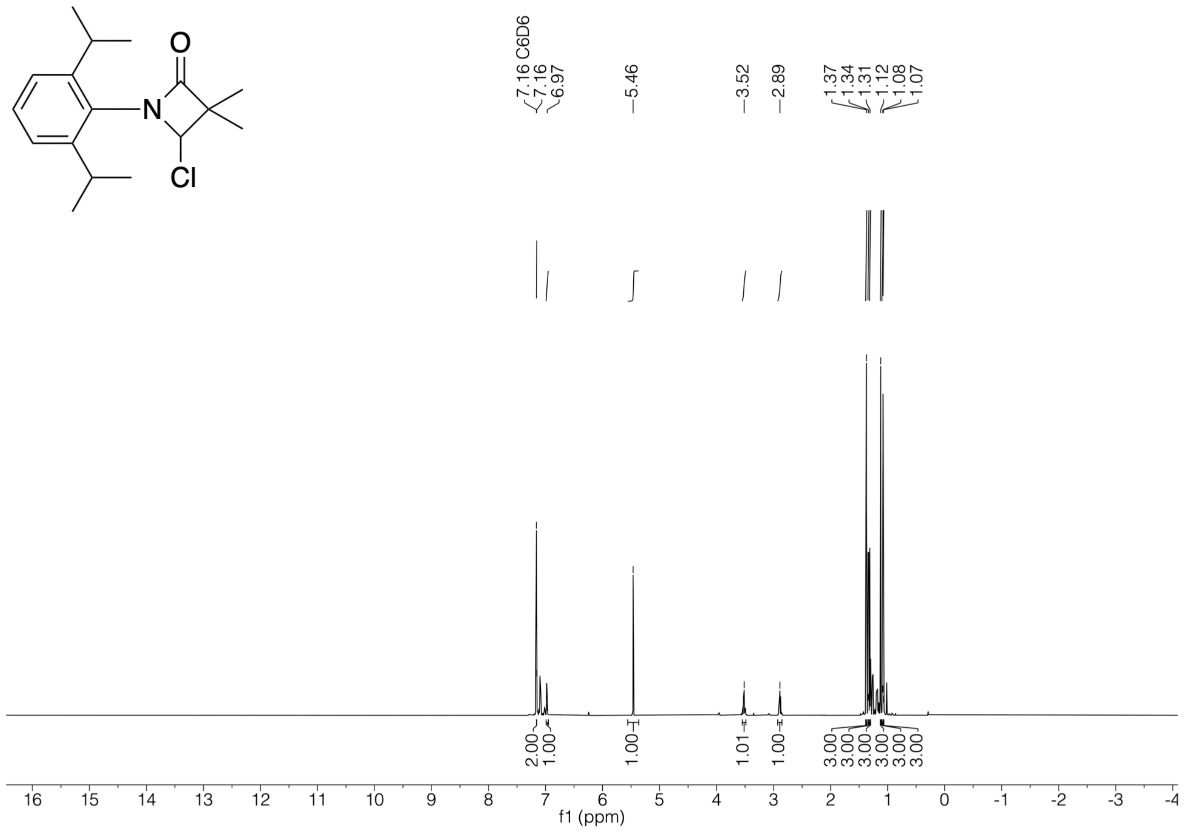

Figure SF94. ^{1}H NMR (300 MHz, C_6D_6, 298.0 K) spectrum of **Dipp-6-Me$_2$*HCl**.

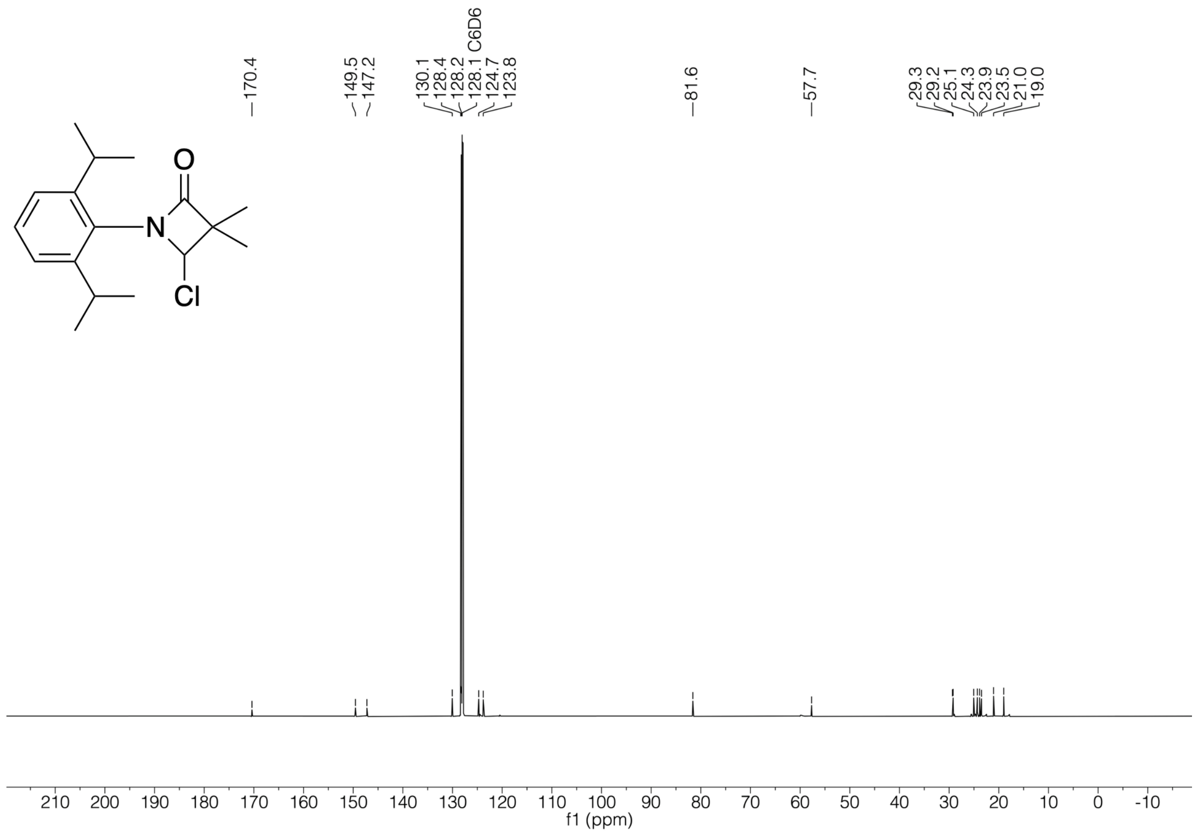

Figure SF95. $^{13}C\{^1H\}$ NMR (75 MHz, C_6D_6, 298.0 K) spectrum of **Dipp-6-Me$_2$*HCl**.

Dipp-6-Ph$_2$*HCl: 1,3,4-oxadiazole-based precursor used: **Dipp-5-Ph$_2$** (2.34 g, 5 mmol). Colorless, air-stable powder. Yield: 2.07 g, 99%. ^{1}H NMR (300 MHz, C_6D_6, 298.0 K): δ = 7.71–7.66 (m, 2H; CH_{aryl}), 7.59–7.54 (m, 2H; CH_{aryl}), 7.21–7.17 (m, 2H; CH_{aryl}), 7.12–7.03 (m, 5H; CH_{aryl}), 7.01–6.94 (m, 2H; CH_{aryl}), 6.59 (s, 1H; NC(H)(Cl)C), 3.77 (sept, J = 7 Hz, 1H; CH_{iPr}), 2.69 (sept, J = 7 Hz, 1H; CH_{iPr}), 1.45 (d, J = 7 Hz, 3H; $CH_{3\text{-}iPr}$),1.23 (d, J = 7 Hz, 3H; $CH_{3\text{-}iPr}$), 0.97 (d, J = 7 Hz, 3H; $CH_{3\text{-}iPr}$), 0.87 (d, J = 7 Hz, 3H; $CH_{3\text{-}iPr}$). $^{13}C\{^1H\}$ NMR (75 MHz, C_6D_6, 298.0 K): δ = 167.8 (s; C=O), 149.4 (s; C_{aryl}), 147.8 (s; C_{aryl}), 139.5 (s; C_{aryl}), 138.4 (s; C_{aryl}), 132.1 (s; C_{aryl}), 130.5 (s; C_{aryl}), 130.2 (s; C_{aryl}), 129.2 (s; C_{aryl}), 129.1 (s; C_{aryl}), 128.8 (s; C_{aryl}), 128.7 (s; C_{aryl}), 128.3 (s; C_{aryl}), 127.5 (s; C_{aryl}), 124.6 (s; C_{aryl}), 124.1 (s; C_{aryl}), 80.8 (s; N*C*(H)(Cl)C), 74.7 (s; C(*C*(Ph)$_2$)C), 29.6 (s; CH_{iPr}), 28.9 (s; CH_{iPr}), 25.4 (s; $CH_{3\text{-}iPr}$), 24.2 (s; $CH_{3\text{-}iPr}$), 23.5 (s; $CH_{3\text{-}iPr}$), 23.4 (s; $CH_{3\text{-}iPr}$) ppm [Some of the expected signals for aryl carbon atoms are not observed due to isochrony as well as overlapping with the solvent signal.]. MS (EI, 70 eV, 70 °C) *m/z* (%): 417 (5) $[M]^{+\cdot}$, 214 (100), 194 (47), 162 (52), 105 (10). Elemental analysis calcd for $C_{27}H_{28}ClNO$: C 77.59, H 6.75, N 3.35, found: C 77.62, H 6.98, N 3.47.

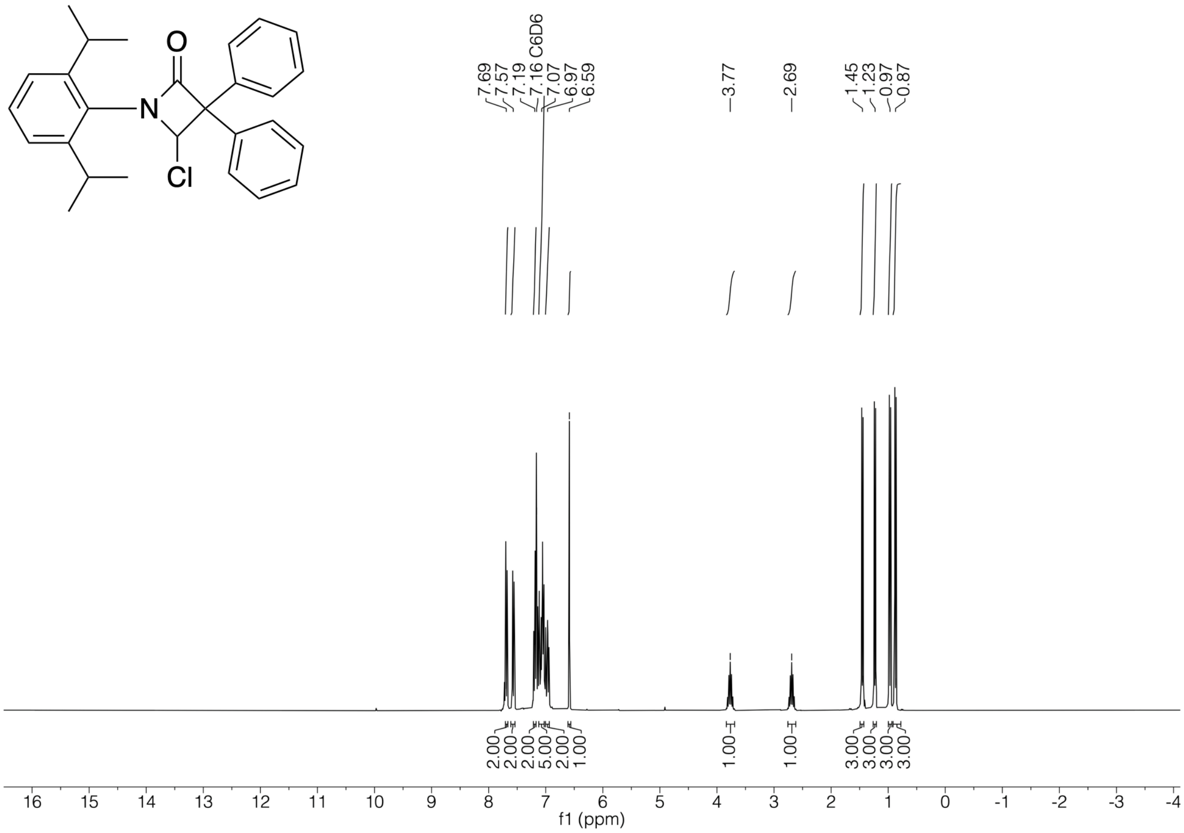

Figure SF96. ^{1}H NMR (300 MHz, C_6D_6, 298.0 K) spectrum of **Dipp-6-Ph$_2$*HCl**.

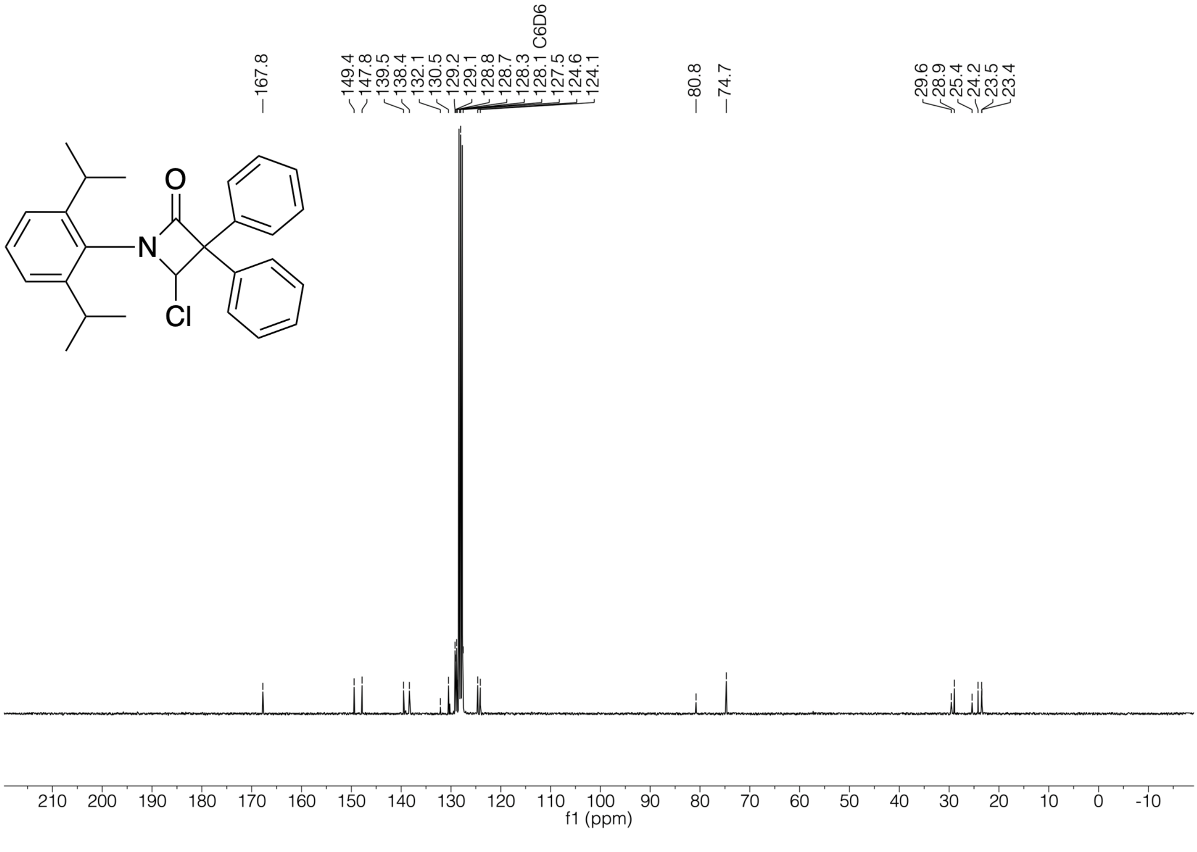

Figure SF97. ^{13}C{^{1}H} NMR (75 MHz, C_6D_6, 298.0 K) spectrum of **Dipp-6-Ph$_2$*HCl**.

Dipp-6-sCy*HCl: 1,3,4-oxadiazole-based precursor used: **Dipp-5-sCy** (192 mg, 0.5 mmol). Colorless, non-air-stable powder. Yield: 7 mg, 4%. Due to the low yield no $^{13}C\{^1H\}$ NMR spectra could be recorded. ^1H NMR (300 MHz, C_6D_6, 298.0 K): δ = 7.12–7.02 (m, 1H; $CH_{aryl\text{-}para}$), 7.00–6.97 (m, 2H; $CH_{aryl\text{-}meta}$), 5.57 (s, 1H; NC(H)(Cl)C), 3.54 (sept, J = 7 Hz, 1H; CH_{iPr}), 2.98 (sept, J = 7 Hz, 1H; CH_{iPr}), 2.18–2.05 (m, 1H; CH_{aliph}), 1.95–1.90 (m, 2H; CH_{aliph}), 1.83–1.70 (m, 4H; CH_{aliph}), 1.65–1.59 (m, 2H; CH_{aliph}), 1.50–1.45 (m, 1H; CH_{aliph}), 1.35 (d, J = 7 Hz, 3H; $CH_{3\text{-}iPr}$), 1.32 (d, J = 7 Hz, 3H; $CH_{3\text{-}iPr}$), 1.10 (d, J = 7 Hz, 3H; $CH_{3\text{-}iPr}$), 1.08 (d, J = 7 Hz, 3H; $CH_{3\text{-}iPr}$) ppm. MS (EI, 70 eV, 60 °C) *m/z* (%): 333 (11) $[M]^{+\cdot}$, 298 (26), 284 (23), 203 (100), 188 (82), 162 (25), 146 (26), 110 (24). Elemental analysis calcd for $C_{20}H_{28}ClNO$: C 79.94, H 8.54, N 4.19, found: C 80.05, H 8.68, N 4.52.

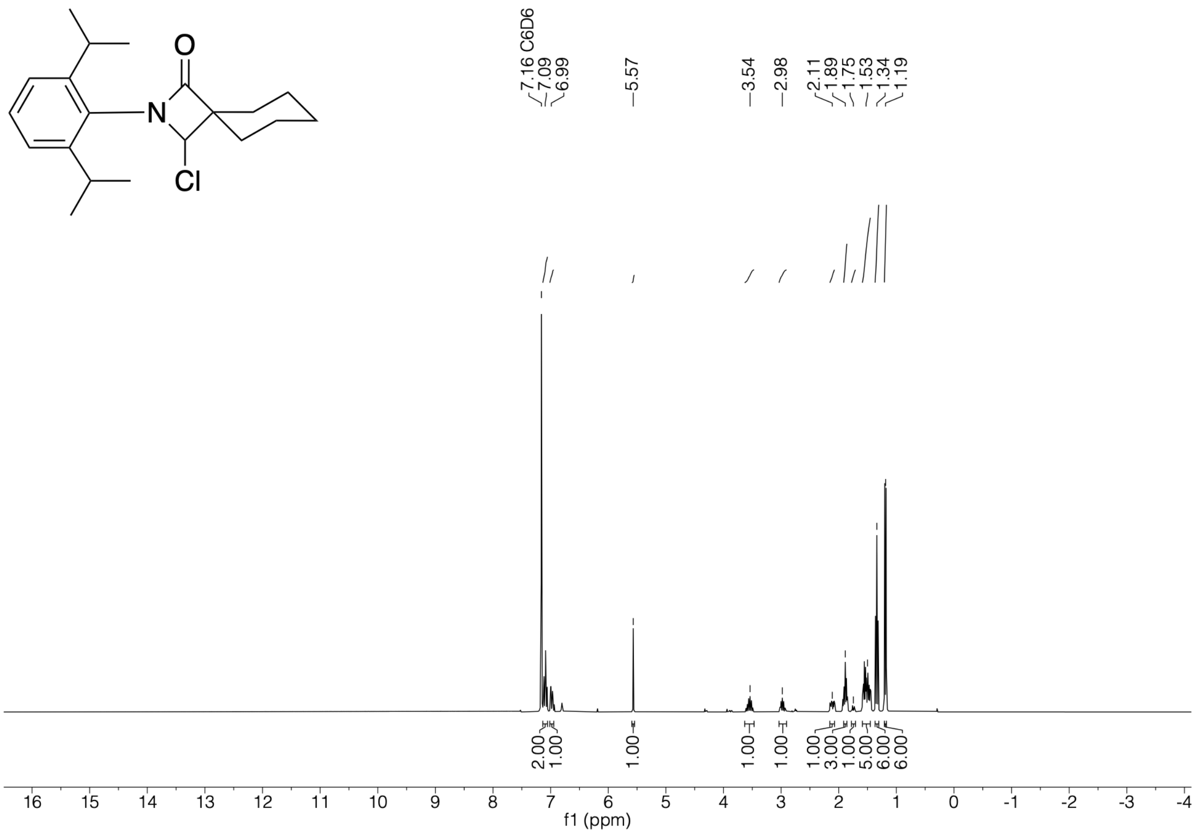

Figure SF98. ^1H NMR (300 MHz, C_6D_6, 298.0 K) spectrum of **Dipp-6-sCy*HCl**.

h. Synthesis of selenium adducts 6*Se

Method A: The corresponding 1,3,4-oxadiazole-based precursor **5** (0.5 mmol, 1 eq.) and grey selenium (78 mg, 1 mmol, 2 eq.) were suspended in 2 ml toluene at ambient temperature. After heating at 110 °C for 16 h, the grey suspension was filtered over silica, eluted with cyclohexane/diethyl ether 1:1 and the orange fraction was collected. After removal of all volatiles, an orange powder was obtained.

Method B: Alternatively, the selenium adducts **6*Se** can be synthesized by reaction of grey selenium with the carbenes generated in situ using the precursors **6*HCl** and NaHMDS as a strong base. The precursors of type **6*HCl** (0.5 mmol, 1 eq.) and grey selenium (78 mg, 1 mmol, 2 eq.) were suspended in 10 ml of tetrahydrofuran followed by the addition 0.6 ml of NaHMDS in THF (1 M, 0.6 mmol, 1.2 eq.) under stirring at. -80 °C. After 2 h the cold bath was removed and stirring was continued at ambient temperature for 14 h. All volatiles were removed in vacuo and the resulting brown powder was suspended in 2 ml of diethyl ether, filtered over silica, eluted with cyclohexane/diethyl ether 1:1 and the orange fraction was collected. After removal of all volatiles, an orange powder was obtained.

Ph-6-Me$_2$*Se: Precursor used: **Ph-5-Me$_2$** (130 mg, 0.5 mmol) for method A and **Ph-6-Me$_2$*HCl** (105 mg, 0.5 mmol) method B. Orange, air-stable powder. Yield: 121 mg, 96% (method A) / 116 mg, 92% (method B). ^{1}H NMR (300 MHz, C_6D_6, 298.0 K): δ = 8.43–8.35 (m, 2H; CH_{aryl}), 7.09–7.03 (m, 2H; CH_{aryl}), 7.00–6.93 (m, 1H; $CH_{aryl\text{-}para}$), 1.04 (s, 6H; $C(C(CH_3)_2)C$) ppm. $^{13}C\{^1H\}$ NMR (75 MHz, C_6D_6, 298.0 K): δ = 218.1 (s; C=Se), 174.3 (s; C=O), 136.4 (s; NC_{aryl}), 129.2 (s; $C_{aryl\text{-}meta}$), 127.9 (s; $C_{aryl\text{-}para}$), 120.3 (s; $C_{aryl\text{-}ortho}$), 71.3 (s; $C(\mathit{C}(CH_3)_2)C$), 19.4 (s; $C(C(\mathit{C}H_3)_2)C$) ppm. $^{77}Se\{^1H\}$ NMR (114 MHz, acetone-d_6, 298.0 K): δ = 877 (s; C=Se) ppm. MS (EI, 70 eV, 70 °C) *m/z* (%): 253 (12) $[M]^{+\cdot}$, 145 (100), 130 (13), 77 (10). Elemental analysis calcd for $C_{11}H_{11}NOSe$: C 52.39, H 4.40, N 5.55, found: C 52.47, H 4.61, N 5.23.

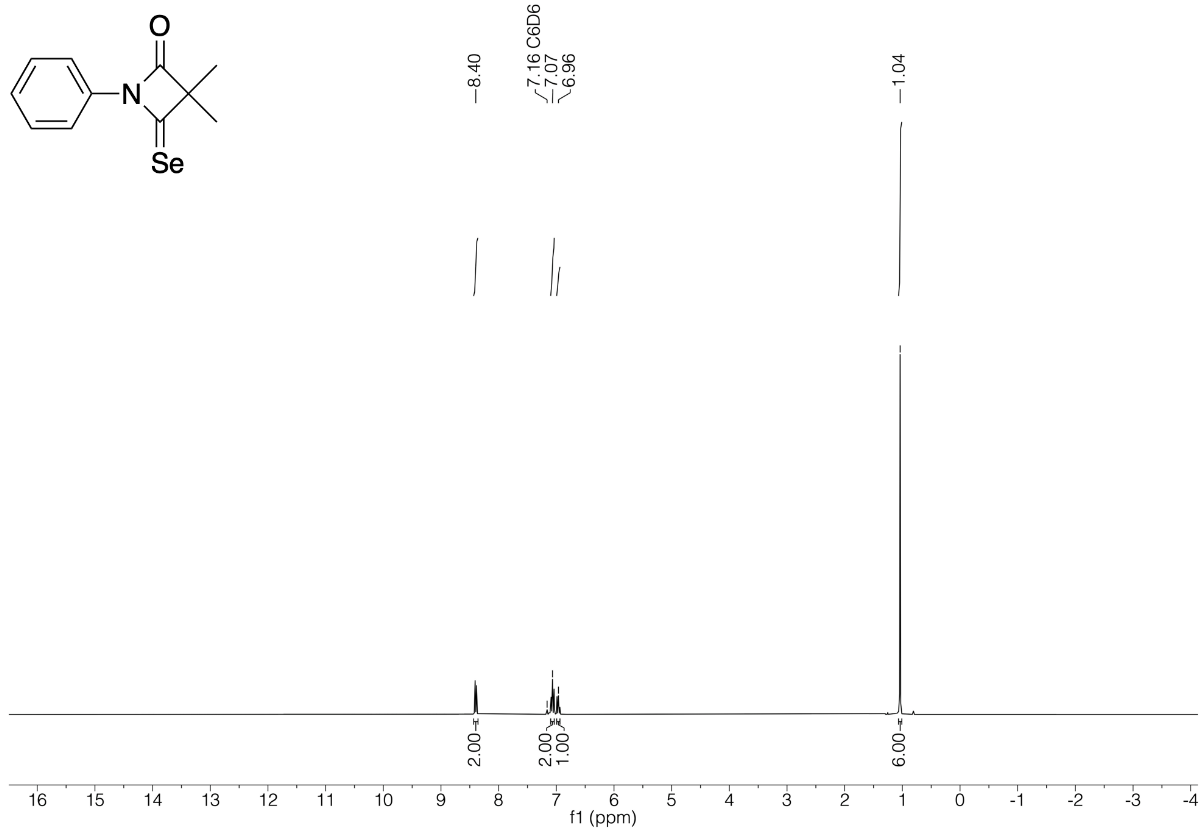

Figure SF99. ^{1}H NMR (300 MHz, C_6D_6, 298.0 K) spectrum of **Ph-6-Me$_2$*Se**.

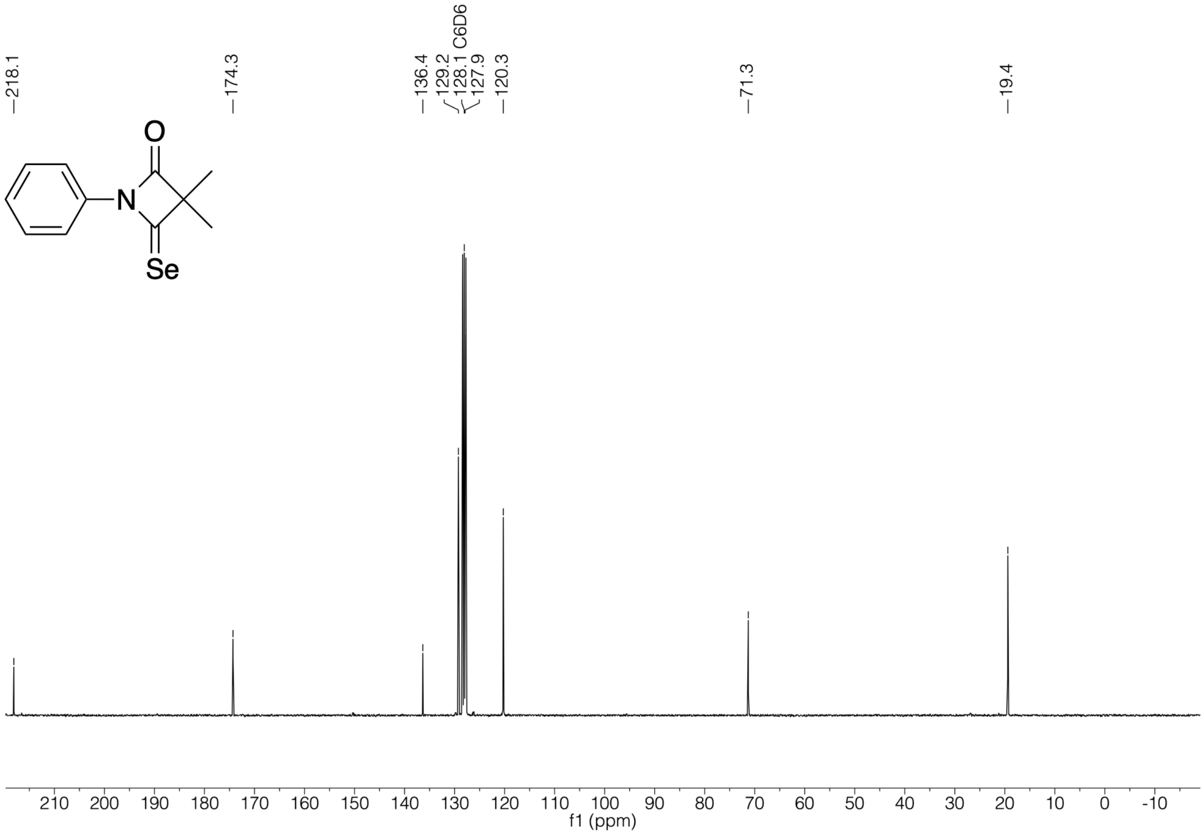

Figure SF100. $^{13}C\{^{1}H\}$ NMR (75 MHz, C_6D_6, 298.0 K) spectrum of **Ph-6-Me$_2$*Se**.

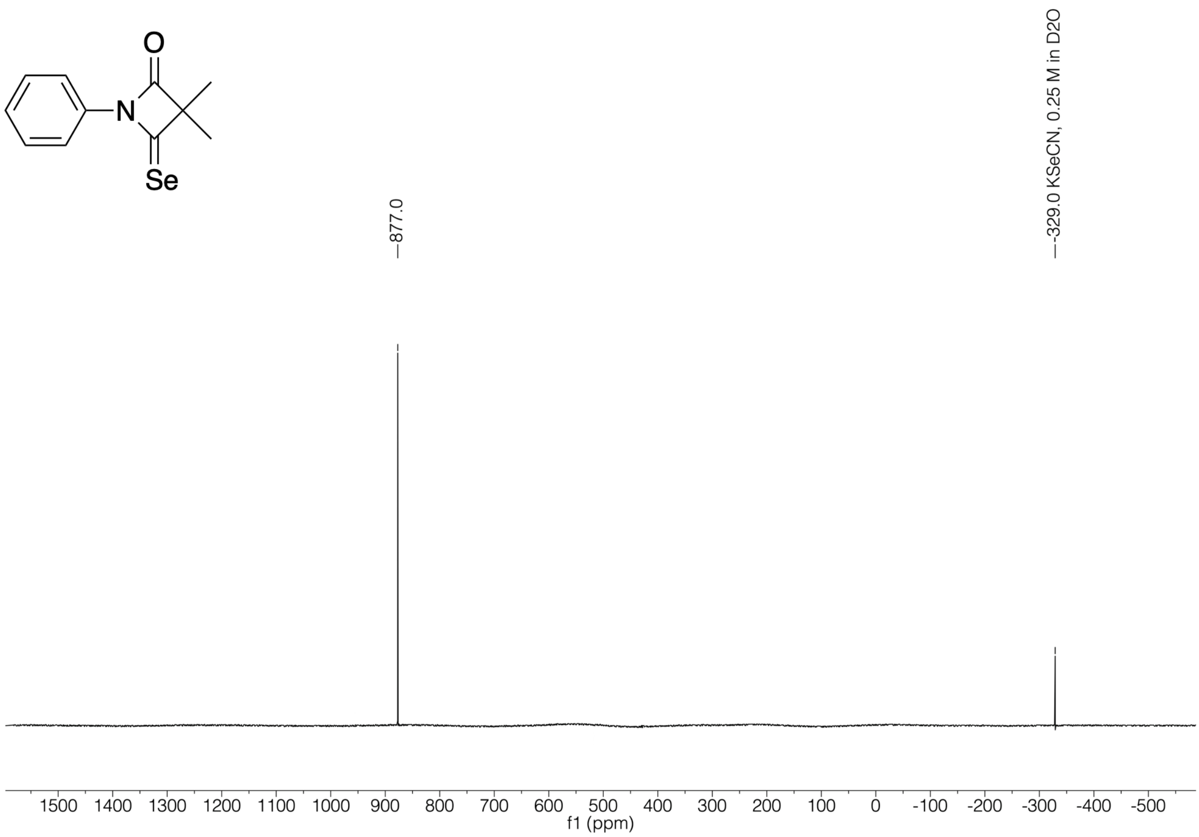

Figure SF101. ^{77}Se{^{1}H} NMR (114 MHz, acetone-d$_6$, 298.0 K) spectrum of **Ph-6-Me$_2$*Se**.

Ph-6-Ph$_2$*Se: Precursor used: **Ph-5-Ph$_2$** (192 mg, 0.5 mmol) for method A and **Ph-6-Ph$_2$*HCl** (167 mg, 0.5 mmol) method B. Orange, air-stable powder. Yield: 184 mg, 98% (method A) / 171 mg, 91% (method B). ^{1}H NMR (300 MHz, C$_6$D$_6$, 298.0 K): δ = 8.23–8.16 (m, 2H; CH$_{aryl}$), 7.82–7.74 (m, 4H; CH$_{aryl}$), 7.12–6.89 (m, 9H; CH$_{aryl}$) ppm. ^{13}C{^{1}H} NMR (75 MHz, C$_6$D$_6$, 298.0 K): δ = 211.0 (s; C=Se), 171.1 (s; C=O), 136.5 (s; C-*C*$_{aryl}$), 135.4 (s; NC$_{aryl}$), 129.2 (s; C$_{aryl}$), 129.1 (s; C$_{aryl}$), 128.6 (s; C$_{aryl}$), 127.9 (s; C$_{aryl}$), 83.9 (s; C(*C*(Ph)$_2$)C) ppm. ^{77}Se{^{1}H} NMR (114 MHz, acetone-d$_6$, 298.0 K): δ = 995 (s; C=Se) ppm [Some of the expected signals for aryl carbon atoms are not observed due to isochrony as well as overlapping with the solvent signal.]. MS (EI, 70 eV, 80 °C) *m/z* (%): 377 (2) [M]$^{+\cdot}$, 269 (10), 246 (6), 194 (100), 165 (36). Elemental analysis calcd for $C_{21}H_{15}NOSe$: C 67.02, H 4.02, N 3.72, found: C 66.86, H 4.15, N 4.08.

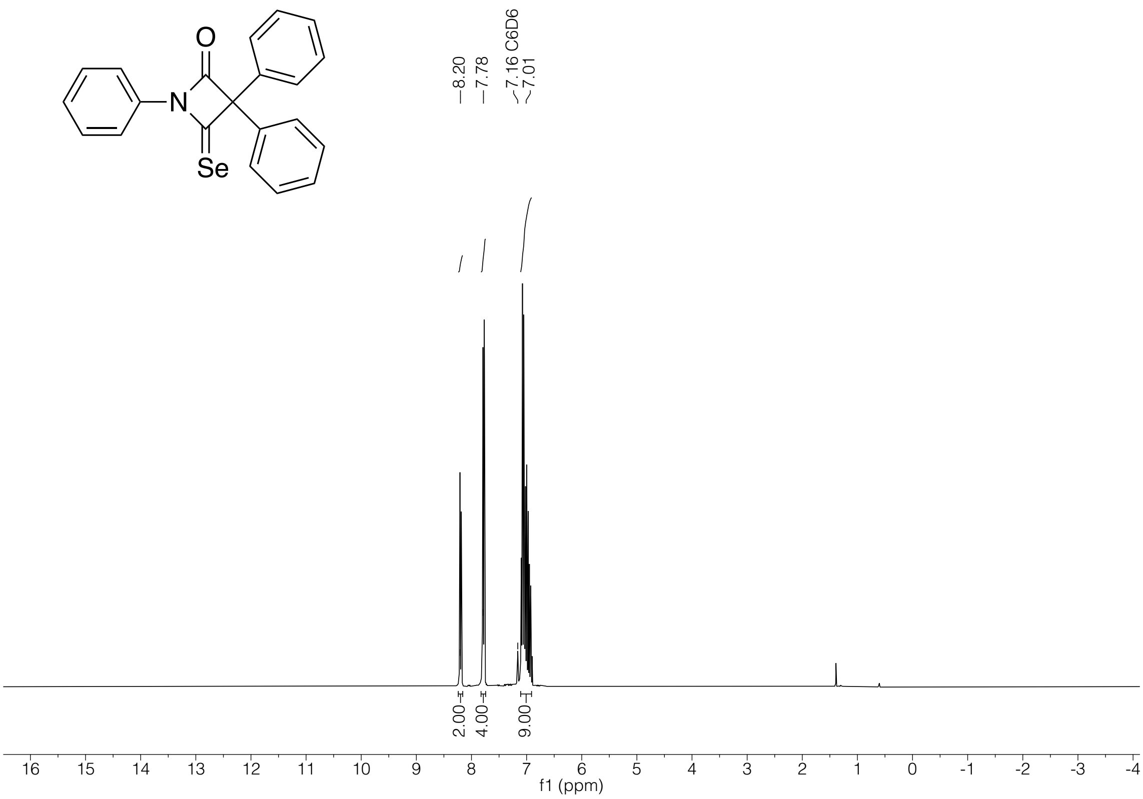

Figure SF102. ^{1}H NMR (300 MHz, C_6D_6, 298.0 K) spectrum of **Ph-6-Ph$_2$*Se**.

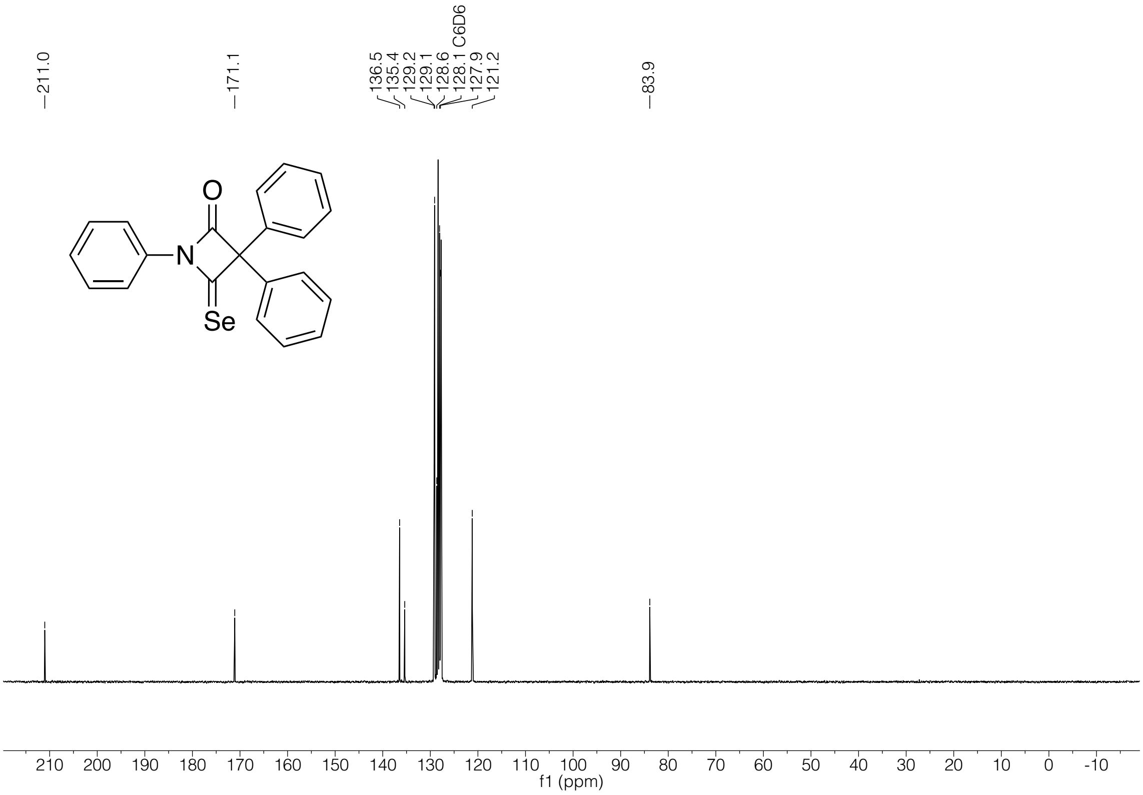

Figure SF103. ^{13}C{^{1}H} NMR (75 MHz, C_6D_6, 298.0 K) spectrum of **Ph-6-Ph$_2$*Se**.

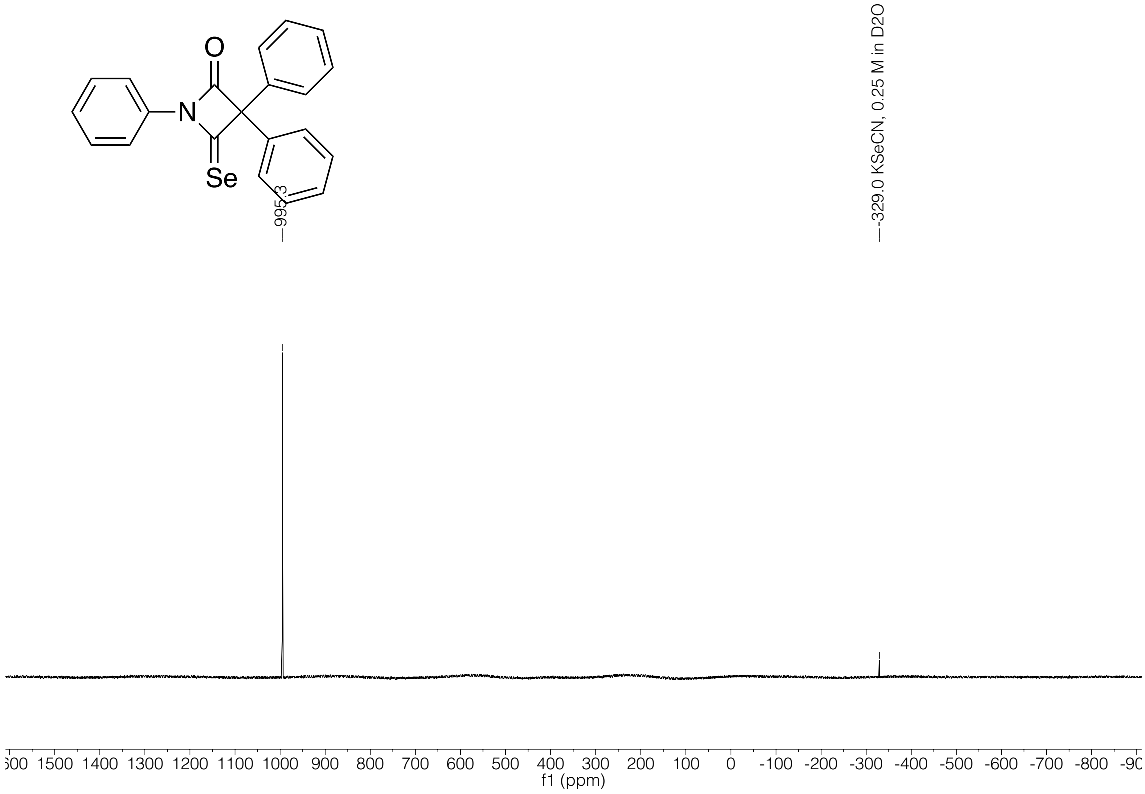

Figure SF104. ^{77}Se{^{1}H} NMR (114 MHz, acetone-d$_6$, 298.0 K) spectrum of **Ph-6-Ph$_2$*Se**.

Ph-6-sCy*Se: Precursor used: **Ph-5-sCy** (150 mg, 0.5 mmol) for method A. Orange, air-stable powder. Yield: 133 mg, 91% (method A). ^{1}H NMR (300 MHz, C_6D_6, 298.0 K): δ = 8.44–8.39 (m, 2H; CH_{aryl}), 7.10–7.03 (m, 2H; CH_{aryl}), 6.99–6.93 (m, 1H; CH_{aryl}), 1.66–1.57 (m, 8H; CH_{aliph}), 1.39–1.43 (m, 1H; CH_{aliph}), 1.06–0.97 (m, 1H; CH_{aliph}) ppm. ^{13}C{^{1}H} NMR (75 MHz, C_6D_6, 298.0 K): δ = 217.9 (s; C=Se), 174.6 (s; C=O), 136.7 (s; NC_{aryl}), 129.5 (s; $C_{aryl\text{-}meta}$), 128.2 (s; $C_{aryl\text{-}para}$), 120.8 (s; $C_{aryl\text{-}ortho}$), 75.3 (s; C(C_{sCy})C), 30.5 (s; C_{aliph}), 25.4 (s; C_{aliph}), 23.4 (s; C_{aliph}) ppm. ^{77}Se{^{1}H} NMR (114 MHz, acetone-d$_6$, 298.0 K): δ = 899 (s; C=Se) ppm. MS (EI, 70 eV, 70 °C) *m/z* (%): 293 (39) $[M]^{+\cdot}$, 185 (100), 145 (38), 77 (9). Elemental analysis calcd for $C_{14}H_{17}NOSe$: C 57.14, H 5.82, N 4.76, found: C 56.87, H 6.02, N 4.55.

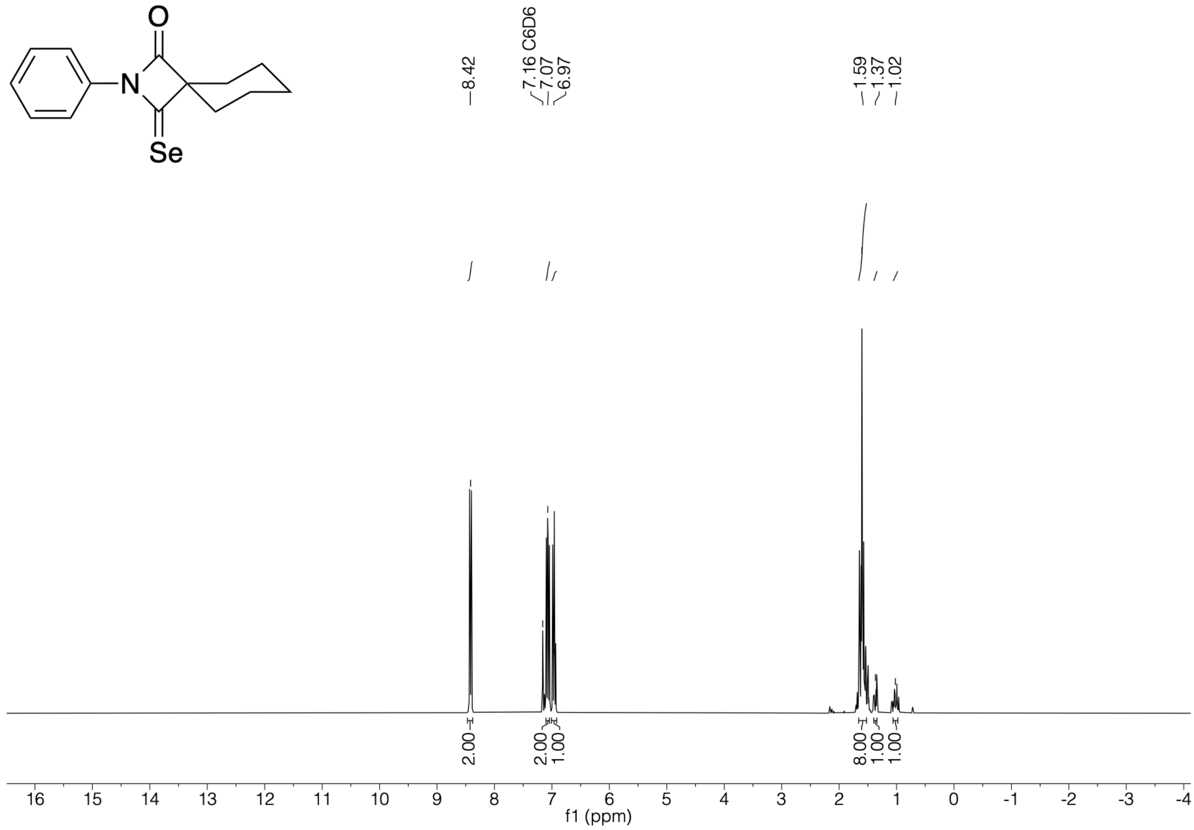

Figure SF105. ^{1}H NMR (300 MHz, C_6D_6, 298.0 K) spectrum of **Ph-6-sCy*Se**.

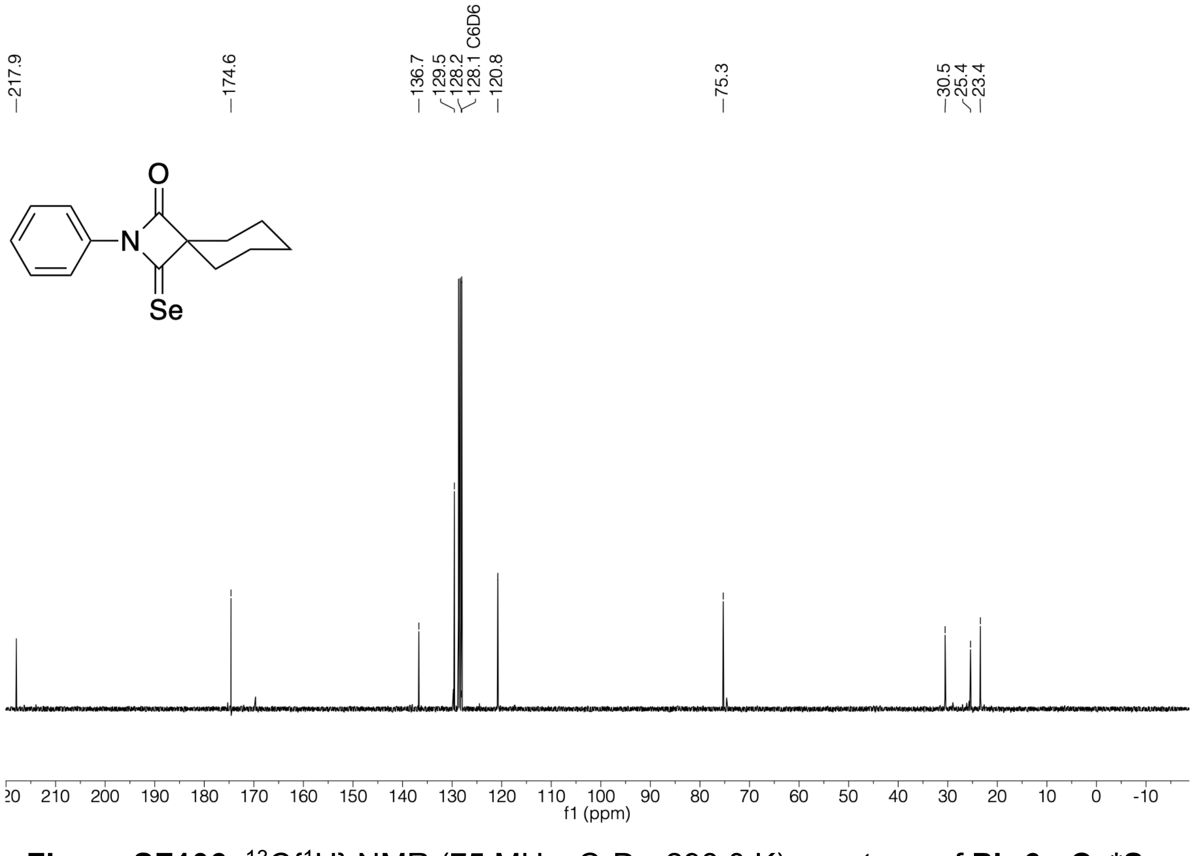

Figure SF106. $^{13}C\{^{1}H\}$ NMR (75 MHz, C_6D_6, 298.0 K) spectrum of **Ph-6-sCy*Se**.

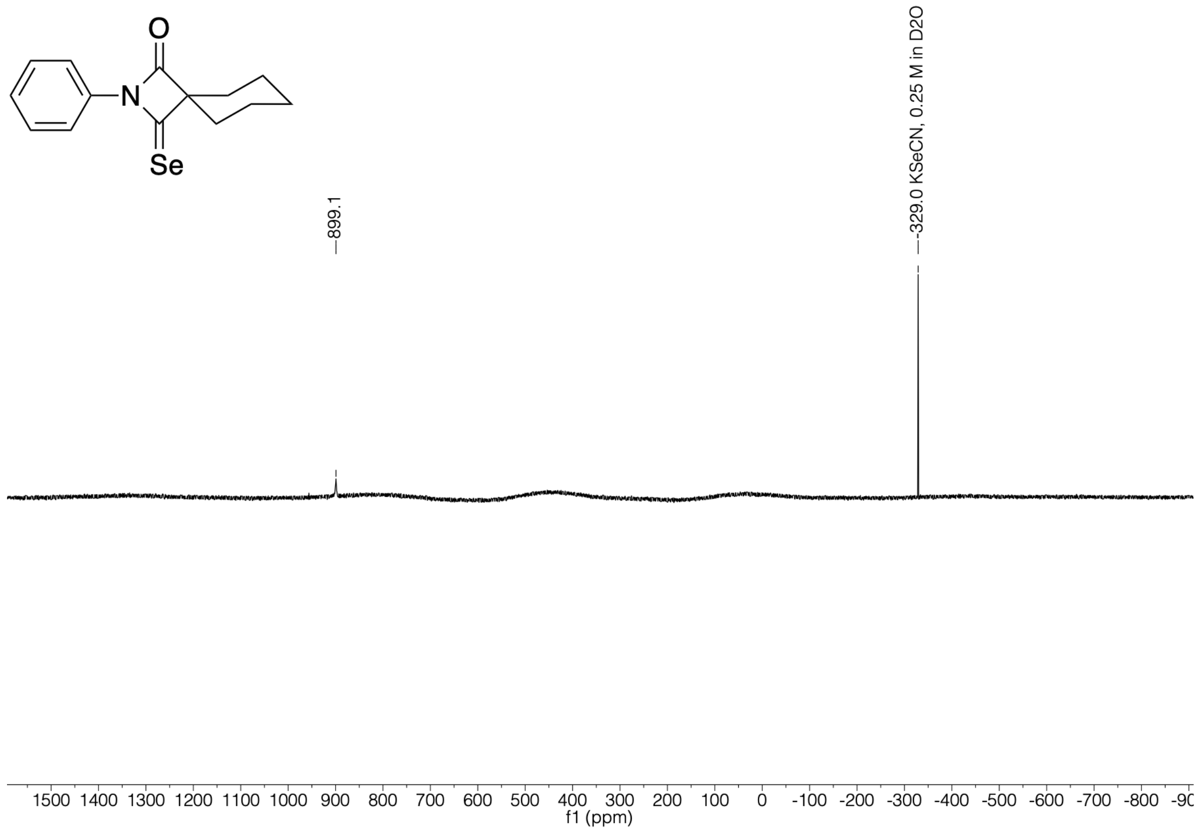

Figure SF107. ^{77}Se{^{1}H} NMR (114 MHz, acetone-d$_6$, 298.0 K) spectrum of **Ph-6-sCy*Se**.

Mes-6-Me$_2$*Se: Precursor used: **Mes-5-Me$_2$** (151 mg, 0.5 mmol) for method A and **Mes-6-Me$_2$*HCl** (126 mg, 0.5 mmol) method B. yellow, air-stable powder. Yield: 144 mg, 98% (method A) / 137 mg, 93% (method B). ^{1}H NMR (300 MHz, C_6D_6, 298.0 K): δ = 6.58 (d, J = 1 Hz, 2H; CH_{meta}), 2.06 (s, 6H; $CH_{3\text{-}ortho}$), 1.93 (s, 3H; $CH_{3\text{-}para}$), 1.24 (s, 6H; $C(C(CH_3)_2)C$) ppm. ^{13}C{^{1}H} NMR (75 MHz, C_6D_6, 298.0 K): δ = 219.1 (s; C=Se), 175.4 (s; C=O), 139.8 (s; NC_{aryl}), 135.3 (s; $C_{aryl\text{-}para}$), 129.5 (s; $C_{aryl\text{-}ortho}$), 129.5 (s; $C_{aryl\text{-}meta}$), 69.7 (s; $C(\mathit{C}(CH_3)_2)C$), 21.0 (s; $CH_{3\text{-}para}$), 19.9 (s; $CH_{3\text{-}ortho}$), 17.9 (s; $C(C(\mathit{C}H_3)_2)C$) ppm. ^{77}Se{^{1}H} NMR (114 MHz, acetone-d$_6$, 298.0 K): δ = 812 (s; C=Se) ppm. MS (EI, 70 eV, 90 °C) *m/z* (%): 295 (8) $[M]^{+\cdot}$, 225 (32), 187 (100), 172 (41), 145 (35), 91 (14), 77 (8). Elemental analysis calcd for $C_{14}H_{17}NOSe$: C 57.14, H 5.82, N 4.76, found: C 57.23, H 6.09, N 4.44.

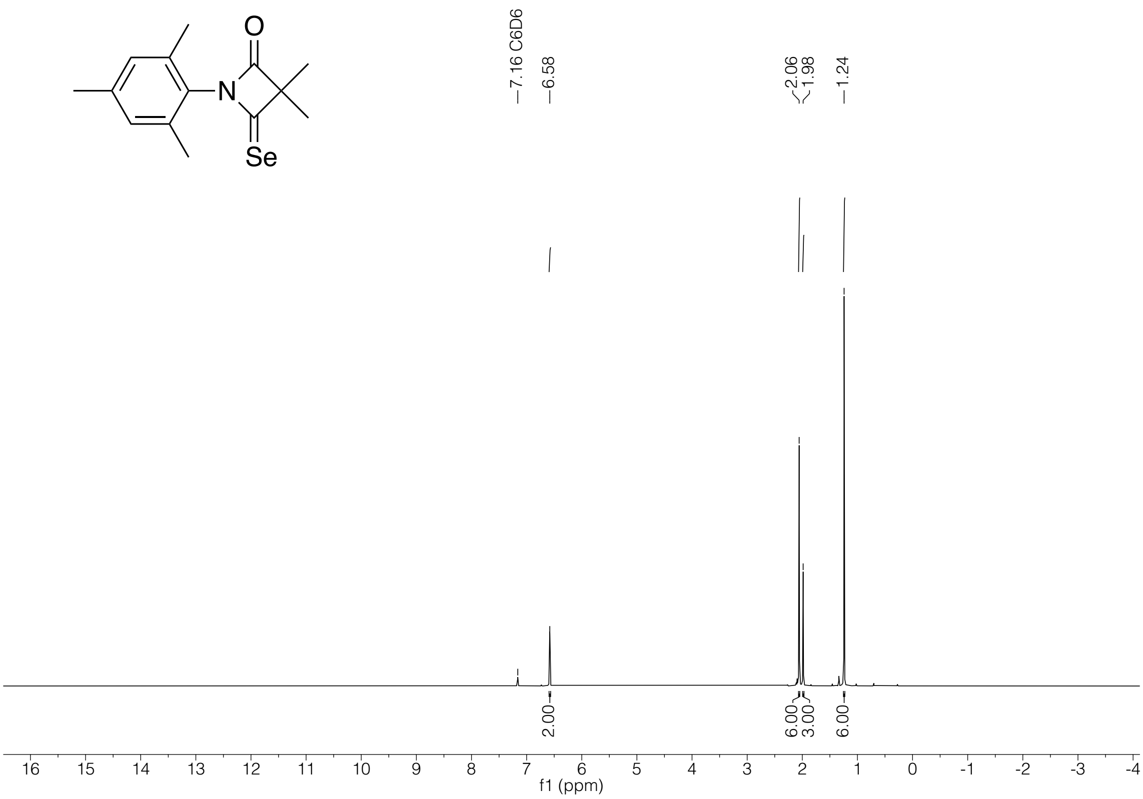

Figure SF108. ^{1}H NMR (300 MHz, C_6D_6, 298.0 K) spectrum of **Mes-6-Me$_2$*Se**.

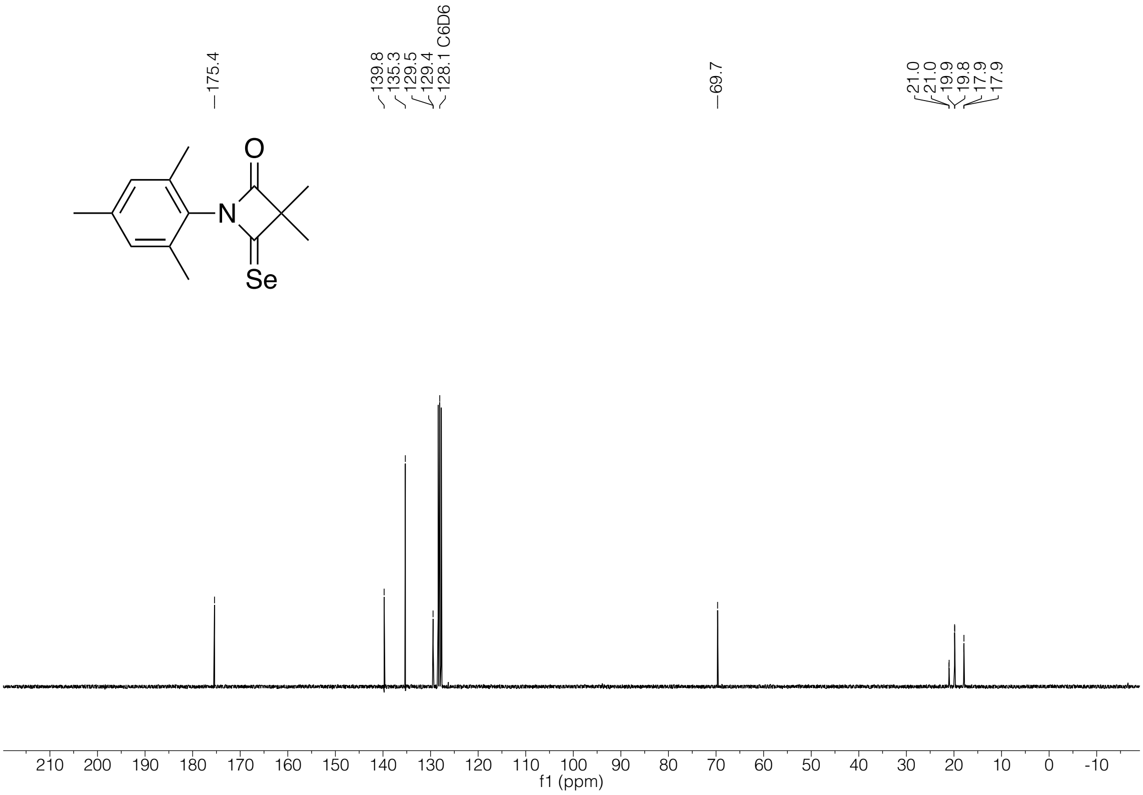

Figure SF109. ^{13}C{^{1}H} NMR (75 MHz, C_6D_6, 298.0 K) spectrum of **Mes-6-Me$_2$*Se**.

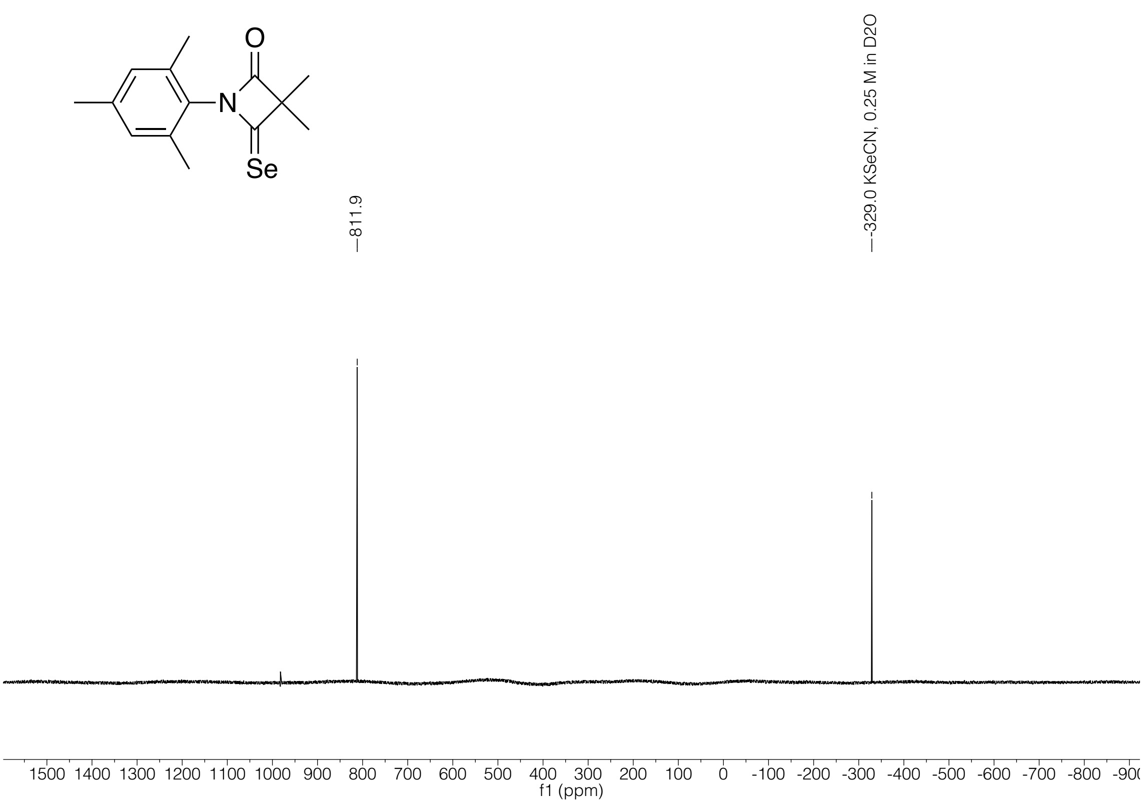

Figure SF110. ^{77}Se{^{1}H} NMR (114 MHz, acetone-d$_6$, 298.0 K) spectrum of **Mes-6-Me$_2$*Se**.

Mes-6-Cl$_2$*Se Precursor used: **Mes-5-Cl$_2$** (171 mg, 0.5 mmol) for method A. Orange, air-stable powder. Yield: 70 mg, 42% (method A). ^{1}H NMR (300 MHz, C$_6$D$_6$, 298.0 K): δ = 6.44 (d, J = 1 Hz, 2H; CH$_{meta}$), 1.94 (s, 6H; CH$_{3\text{-ortho}}$), 1.90 (s, 3H; CH$_{3\text{-para}}$) ppm. ^{13}C{^{1}H} NMR (75 MHz, C$_6$D$_6$, 298.0 K): δ = 204.2 (s; C=Se), 164.7 (s; C=O), 140.9 (s; NC$_{aryl}$), 135.1 (s; C$_{aryl\text{-para}}$), 129.7 (s; C$_{aryl\text{-ortho}}$), 126.5 (s; C$_{aryl\text{-meta}}$), 91.2 (s; C(*C*(Cl)$_2$)C), 20.9 (s; CH$_{3\text{-para}}$), 17.5 (s; CH$_{3\text{-ortho}}$) ppm. ^{77}Se{^{1}H} NMR (114 MHz, acetone-d$_6$, 298.0 K): δ = 950 (s; C=Se) ppm. MS (EI, 70 eV, 80 °C) *m/z* (%): 335 (4) [M]$^{+\cdot}$, 211 (24), 162 (100), 134 (52), 91 (26), 77 (23). Elemental analysis calcd for C$_{12}$H$_{11}$Cl$_2$NOSe: C 43.01, H 3.31, N 4.18, found: C 42.84, H 3.41, N 4.36.

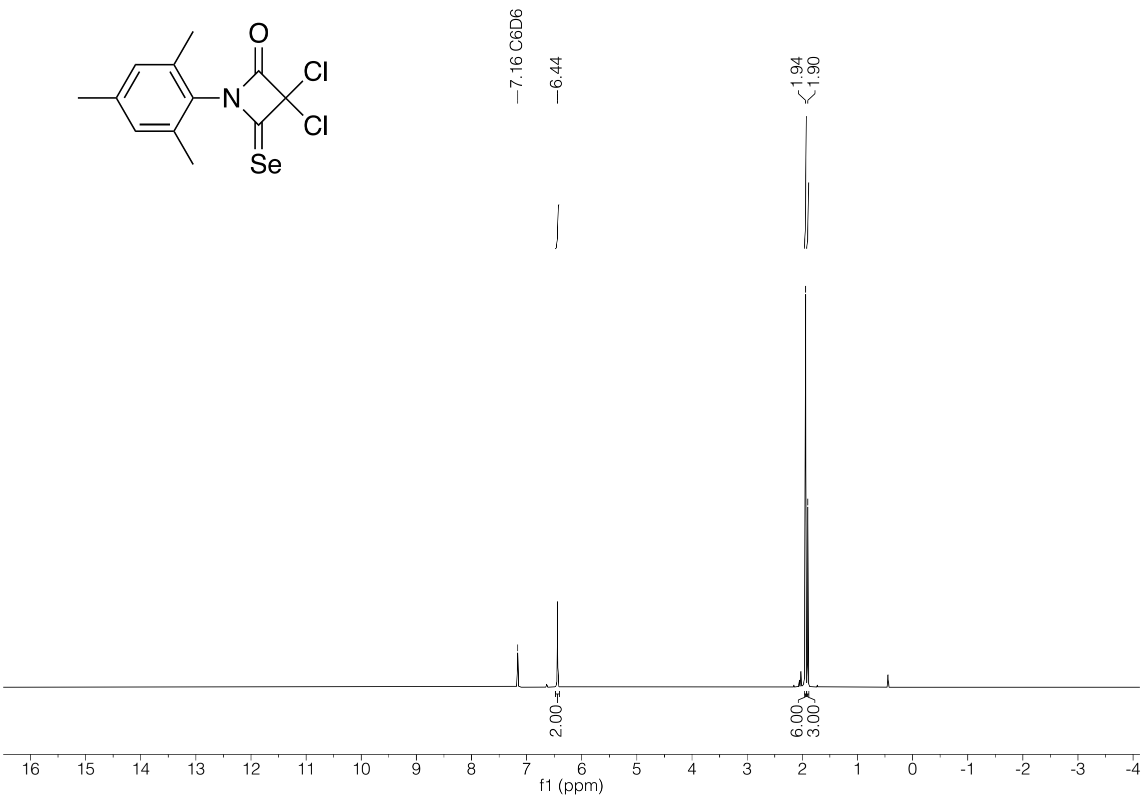

Figure SF111. ^{1}H NMR (300 MHz, C_6D_6, 298.0 K) spectrum of **Mes-6-Cl$_2$*Se**.

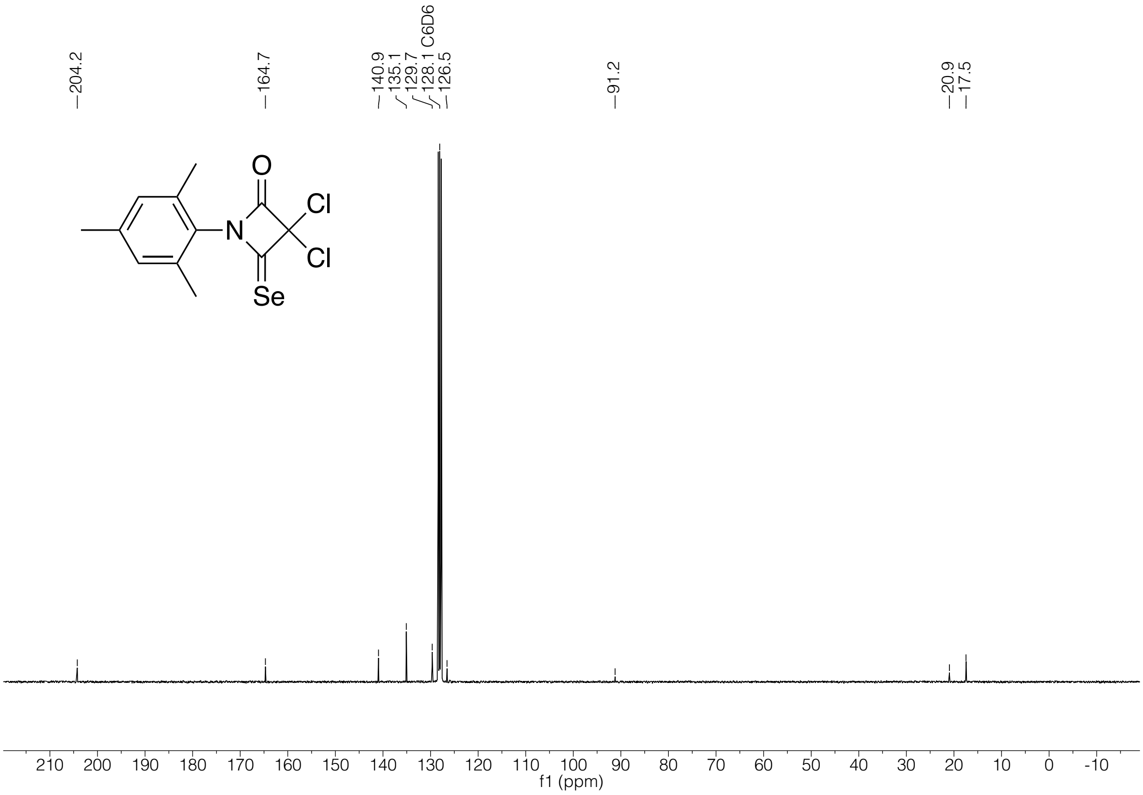

Figure SF112. $^{13}C\{^{1}H\}$ NMR (75 MHz, C_6D_6, 298.0 K) spectrum of **Mes-6-Cl$_2$*Se**.

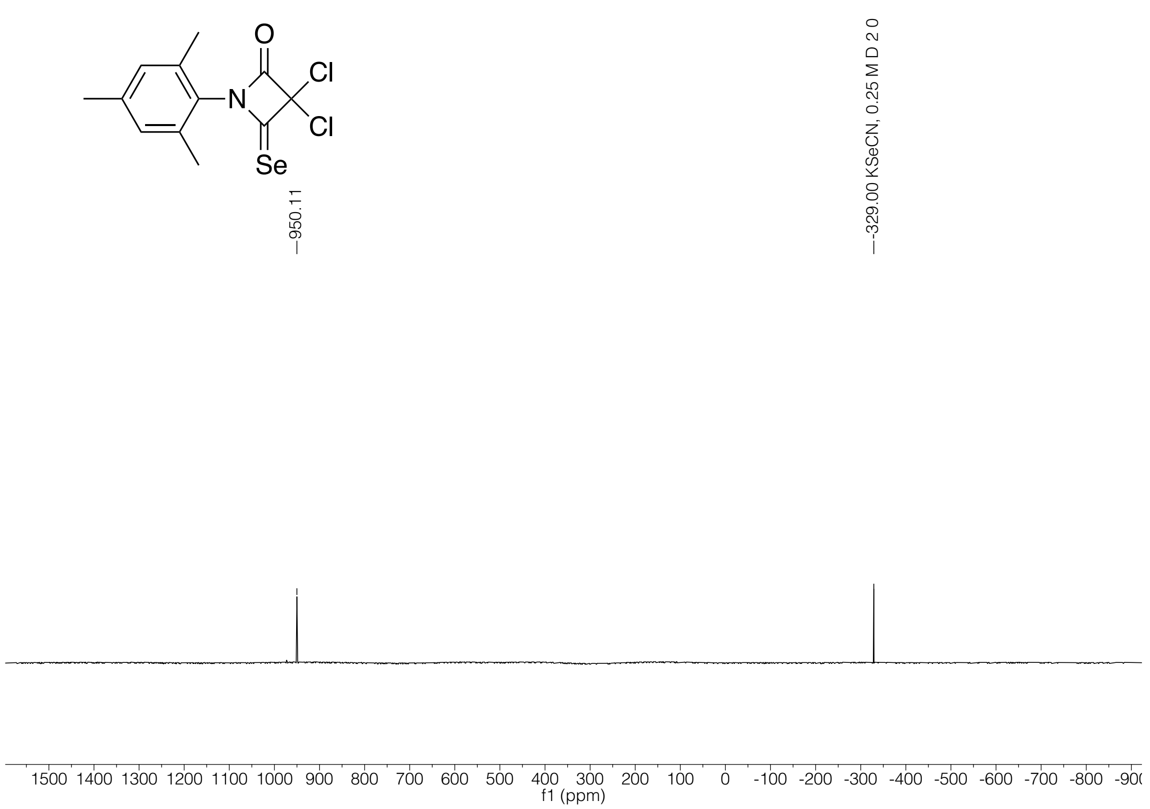

Figure SF113. $^{77}Se\{^{1}H\}$ NMR (114 MHz, acetone-d$_6$, 298.0 K) spectrum of **Mes-6-Cl$_2$*Se**.

Mes-6-Ph$_2$*Se: Precursor used: **Mes-5-Ph$_2$** (213 mg, 0.5 mmol) for method A and **Mes-6-Ph$_2$*HCl** (188 mg, 0.5 mmol) method B. Yellow, air-stable powder. Yield: 207 mg, 99% (method A) / 190 mg, 91% (method B). ^{1}H NMR (300 MHz, C_6D_6, 298.0 K): δ = 7.96–7.90 (m, 4H; CH_{aryl}), 7.14–7.01 (m, 6H; CH_{aryl}), 6.53 (d, J = 1 Hz, 2H; CH_{meta}), 2.05 (s, 6H; $CH_{3\text{-ortho}}$), 1.53 (s, 3H; $CH_{3\text{-para}}$) ppm. $^{13}C\{^{1}H\}$ NMR (75 MHz, C_6D_6, 298.0 K): δ = 215.3 (s; C=Se), 172.3 (s; C=O), 140.2 (s; NC_{aryl}), 137.2 (s; C-C_{aryl}), 135.5 (s; C-C_{aryl}), 129.6 (s; C_{aryl}), 129.5 (s; C_{aryl}), 129.2 (s; C_{aryl}), 128.6 (s; C_{aryl}), 127.6 (s; C_{aryl}), 82.5 (s; C(*C*(Ph)$_2$)C), 21.0 (s; $CH_{3\text{-para}}$), 18.2 (s; $CH_{3\text{-ortho}}$) ppm. $^{77}Se\{^{1}H\}$ NMR (114 MHz, acetone-d$_6$, 298.0 K): δ = 929 (s; C=Se) ppm. MS (EI, 70 eV, 100 °C) *m/z* (%): 419 (1) $[M]^{+\cdot}$, 311 (4), 258 (8), 194 (100), 166 (29). Elemental analysis calcd for $C_{24}H_{21}NOSe$: C 68.90, H 5.06, N 3.35, found: C 68.68, H 5.17, N 3.40.

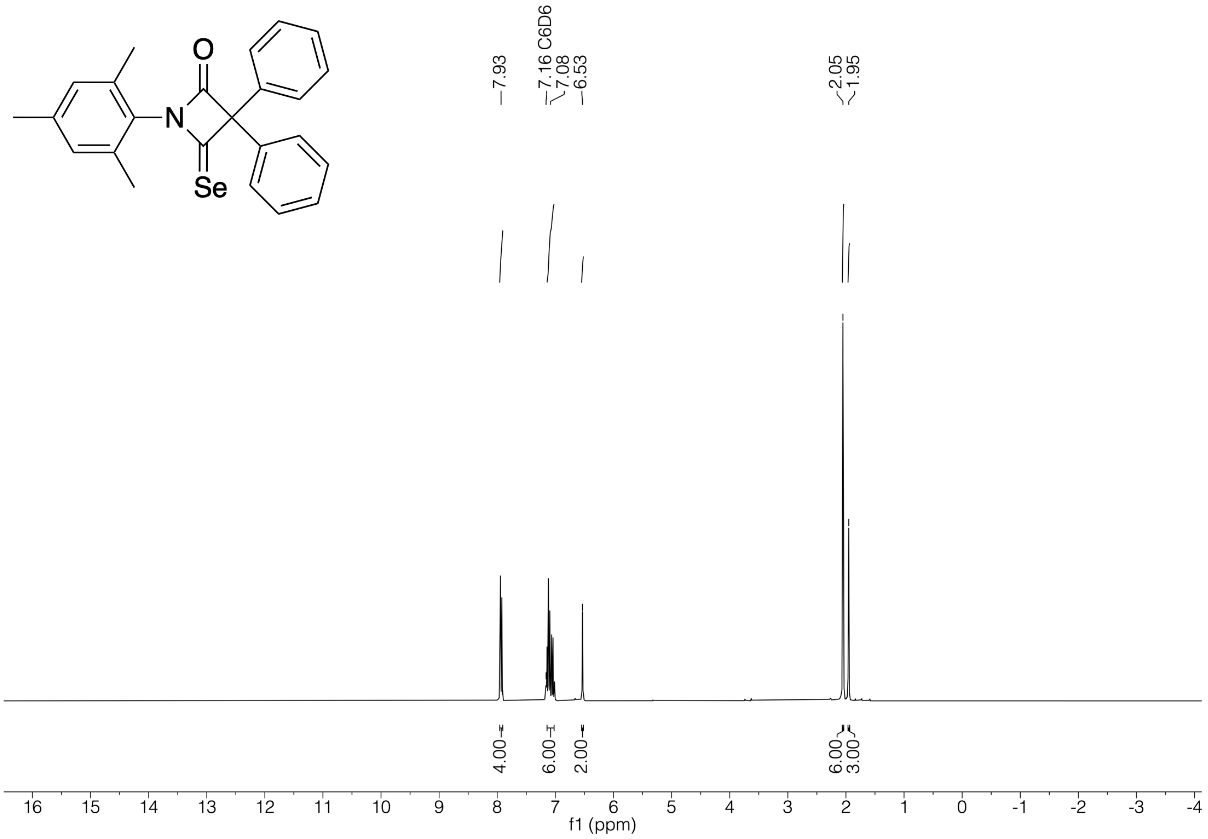

Figure SF114. ^{1}H NMR (300 MHz, C_6D_6, 298.0 K) spectrum of **Mes-6-Ph$_2$*Se**.

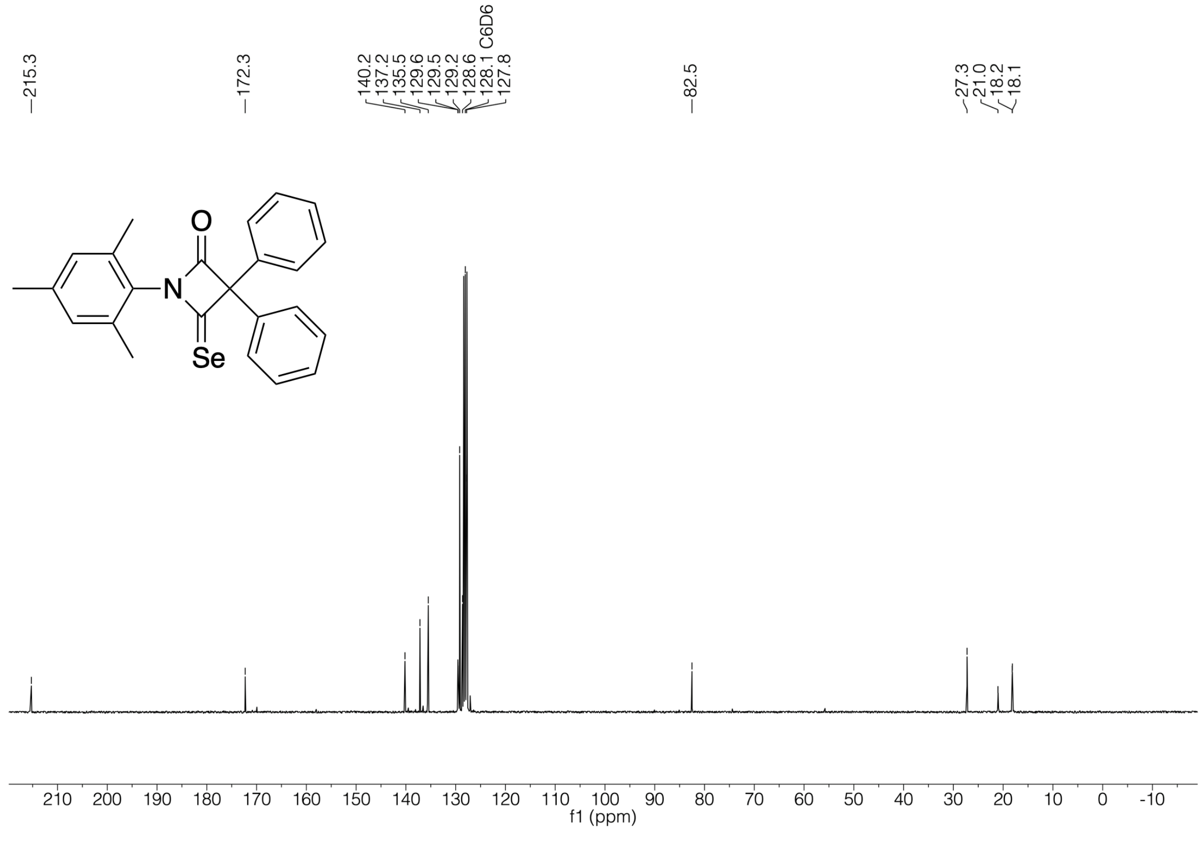

Figure SF115. $^{13}C\{^{1}H\}$ NMR (75 MHz, C_6D_6, 298.0 K) spectrum of **Mes-6-Ph$_2$*Se**.

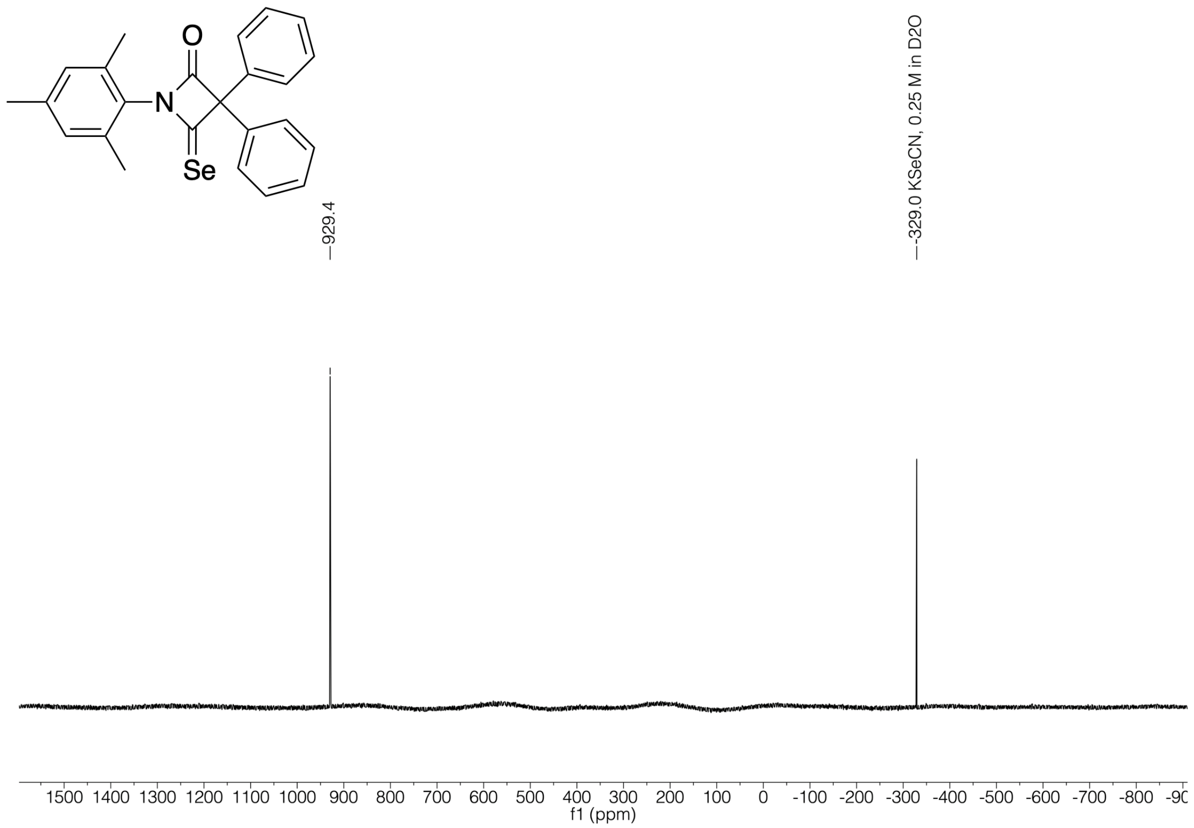

Figure SF116. ^{77}Se{^{1}H} NMR (114 MHz, acetone-d$_6$, 298.0 K) spectrum of **Mes-6-Ph$_2$*Se**.

Mes-6-sCy*Se: Precursor used: **Mes-5-sCy** (171 mg, 0.5 mmol) for method. Yellow, air-stable powder. Yield: 167 mg, 89% (method A). ^{1}H NMR (300 MHz, C_6D_6, 298.0 K): δ = 6.59 (d, J = 1 Hz, 2H; CH_{meta}), 2.09 (s, 6H; $CH_{3\text{-}ortho}$), 1.99 (s, 3H; $CH_{3\text{-}para}$), 1.89–1.82 (m, 4H; CH_{aliph}), 1.76–1.59 (m, 4H; CH_{aliph}), 1.45–1.35 (m, 1H; CH_{aliph}), 1.20–1.10 (m, 1H; CH_{aliph}) ppm. ^{13}C{^{1}H} NMR (75 MHz, C_6D_6, 298.0 K): δ = 213.7 (s; C=Se), 176.5 (s; C=O), 146.5 (s; NC_{aryl}), 130.9 (s; $C_{aryl\text{-}meta}$), 128.0 (s; $C_{aryl\text{-}para}$), 124.2 (s; $C_{aryl\text{-}ortho}$), 73.4 (s; C(C_{sCy})C), 29.7 (s; C_{aliph}), 25.2 (s; C_{aliph}), 24.2 (s; C_{aliph}), 23.7 (s; $CH_{3\text{-}para}$), 23.07 (s; $CH_{3\text{-}ortho}$) ppm. ^{77}Se{^{1}H} NMR (114 MHz, acetone-d$_6$, 298.0 K): δ = 843 (s; C=Se) ppm. MS (EI, 70 eV, 90 °C) *m/z* (%): 335 (22) $[M]^{+\cdot}$, 227 (100), 212 (30), 146 (15), 91 (11). Elemental analysis calcd for $C_{17}H_{21}NOSe$: C 61.07, H 6.33, N 4.19, found: C 60.95, H 6.56, N 4.50.

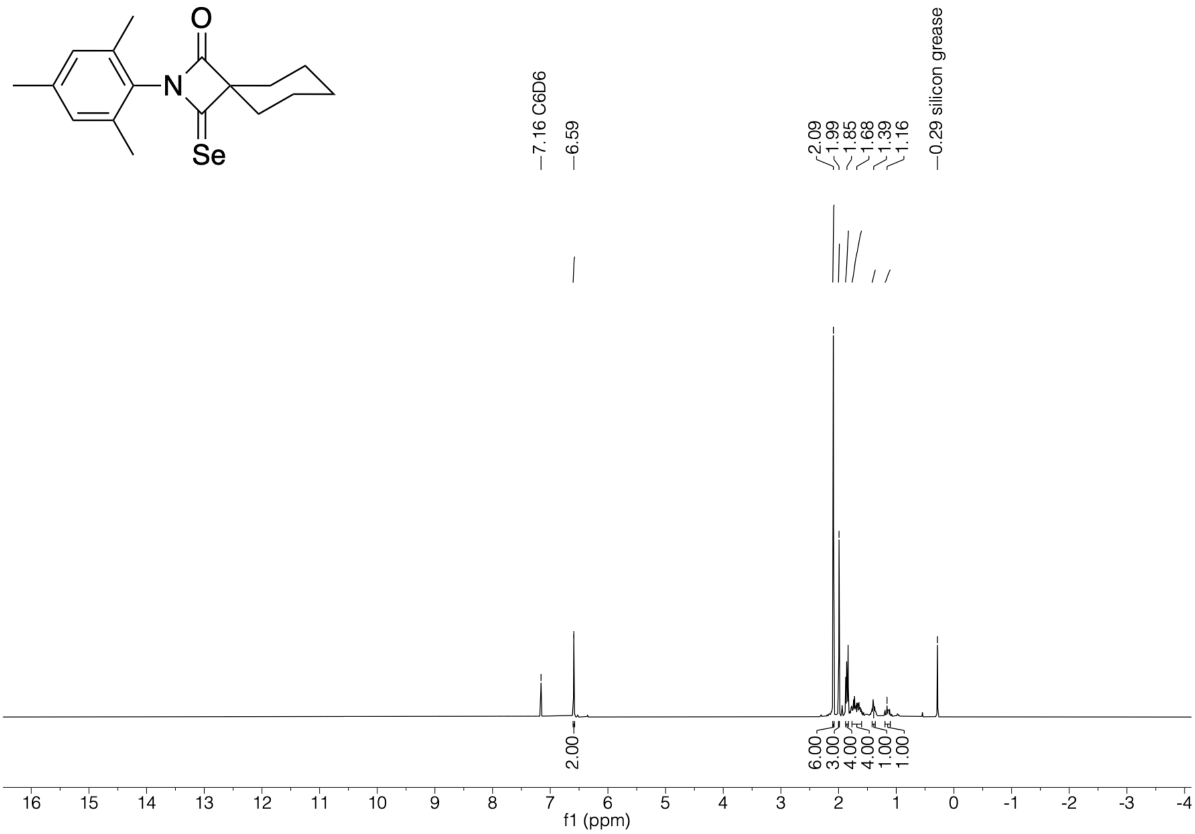

Figure SF117. ^{1}H NMR (300 MHz, C_6D_6, 298.0 K) spectrum of **Mes-6-sCy*Se**.

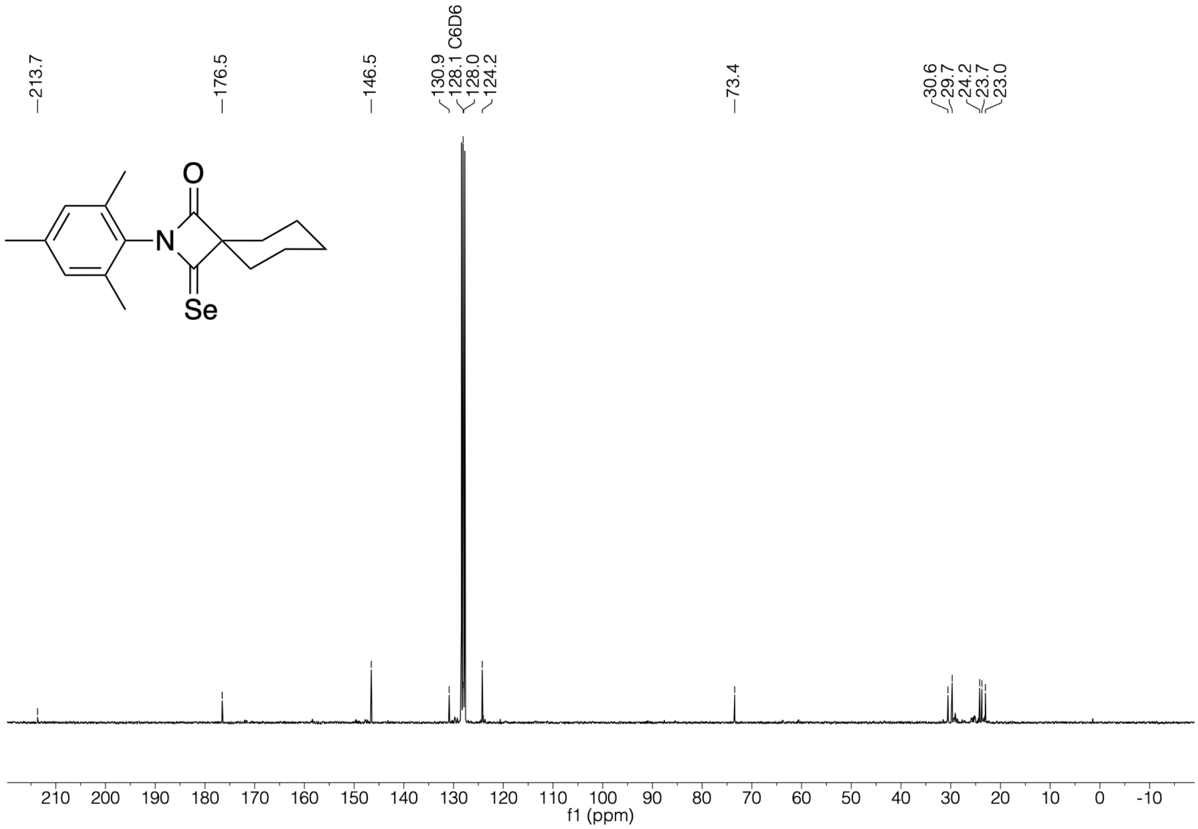

Figure SF118. $^{13}C\{^{1}H\}$ NMR (75 MHz, C_6D_6, 298.0 K) spectrum of **Mes-6-sCy*Se**.

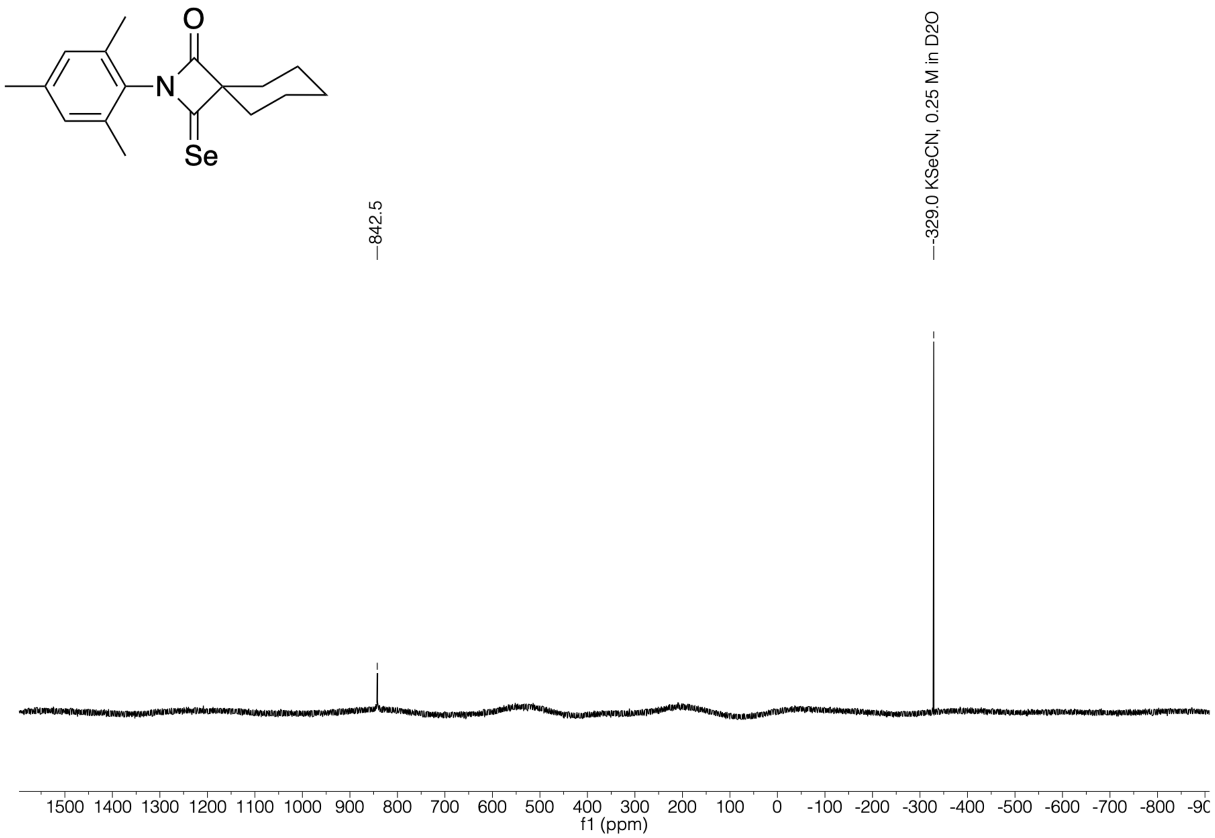

Figure SF119. ^{77}Se{^{1}H} NMR (114 MHz, acetone-d$_6$, 298.0 K) spectrum of **Mes-6-sCy*Se**.

Dipp-6-Me$_2$*Se: Precursor used: **Dipp-5-Me$_2$** (172 mg, 0.5 mmol) for method A and **Dipp-6-Me$_2$*HCl** (147 mg, 0.5 mmol) method B. Yellow, air-stable powder. Yield: 163 mg, 97% (method A) / 158 mg, 94% (method B). ^{1}H NMR (300 MHz, C_6D_6, 298.0 K): δ = 7.15–7.12 (m, 1H; $CH_{aryl\text{-}para}$), 7.04–6.99 (m, 2H; $CH_{aryl\text{-}meta}$), 3.01 (sept, J = 7 Hz, 2H; CH_{iPr}), 1.27 (s, 6H; $C(C(CH_3)_2)C$), 1.15 (d, J = 7 Hz, 6H; $CH_{3\text{-}iPr}$), 1.10 (d, J = 7 Hz, 6H; $CH_{3\text{-}iPr}$) ppm. ^{13}C{^{1}H} NMR (75 MHz, C_6D_6, 298.0 K): δ = 216.0 (s; C=Se), 176.5 (s; C=O), 146.4 (s; $C_{aryl\text{-}ortho}$), 130.9 (s; NC_{aryl}), 127.9 (s; $C_{aryl\text{-}para}$), 124.2 (s; $C_{aryl\text{-}meta}$), 69.8 (s; $C(C(CH_3)_2)C$), 29.7 (s; CH_{iPr}), 24.1 (s; $CH_{3\text{-}iPr}$), 23.7 (s; $CH_{3\text{-}iPr}$), 19.6 (s; $C(C(CH_3)_2)C$) ppm. ^{77}Se{^{1}H} NMR (114 MHz, acetone-d$_6$, 298.0 K): δ = 818 (s; C=Se) ppm. MS (EI, 70 eV, 80 °C) *m/z* (%): 337 (7) $[M]^{+\cdot}$, 266 (51), 229 (67), 214 (48), 186 (69), 173 (100), 146 (12), 91 (10), 77 (6). Elemental analysis calcd for $C_{17}H_{23}NOSe$: C 60.71, H 6.89, N 4.16, found: C 61.02, H 7.15, N 4.07.

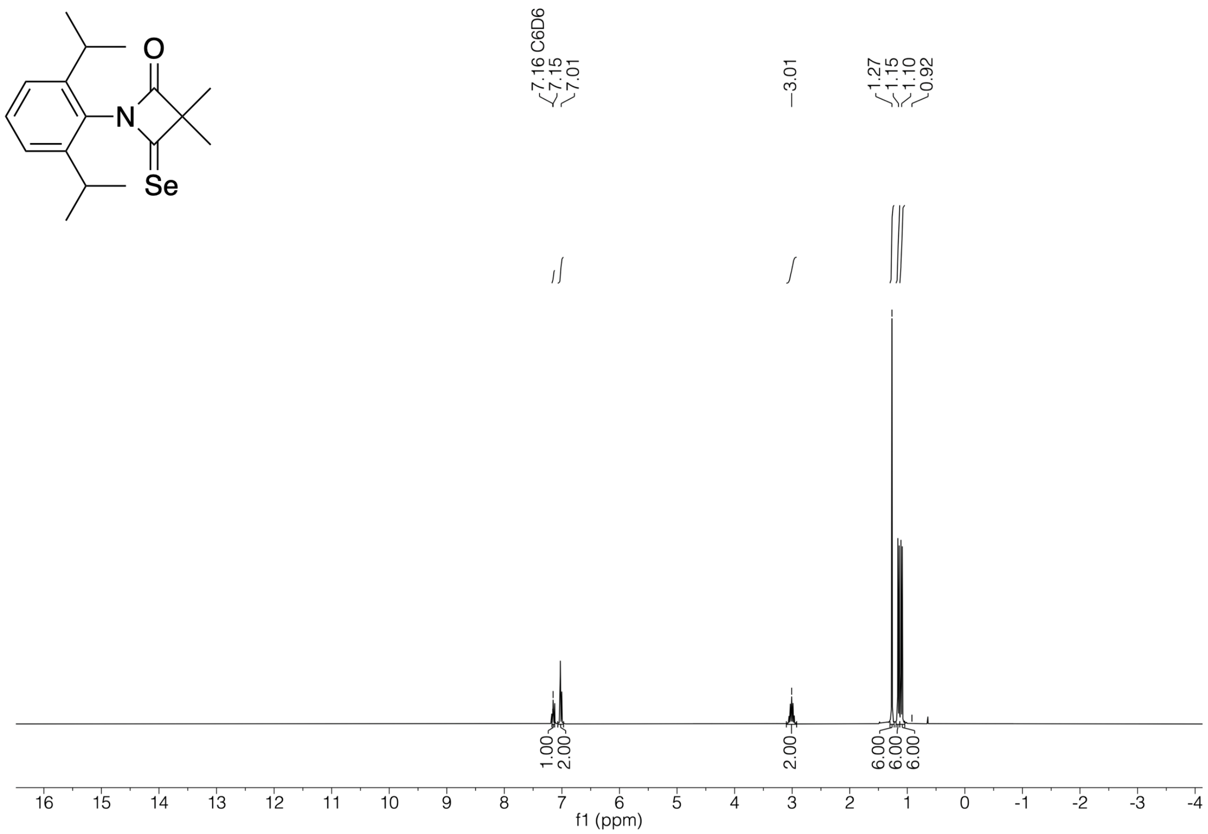

Figure SF120. ^{1}H NMR (300 MHz, C_6D_6, 298.0 K) spectrum of **Dipp-6-Me$_2$*Se**.

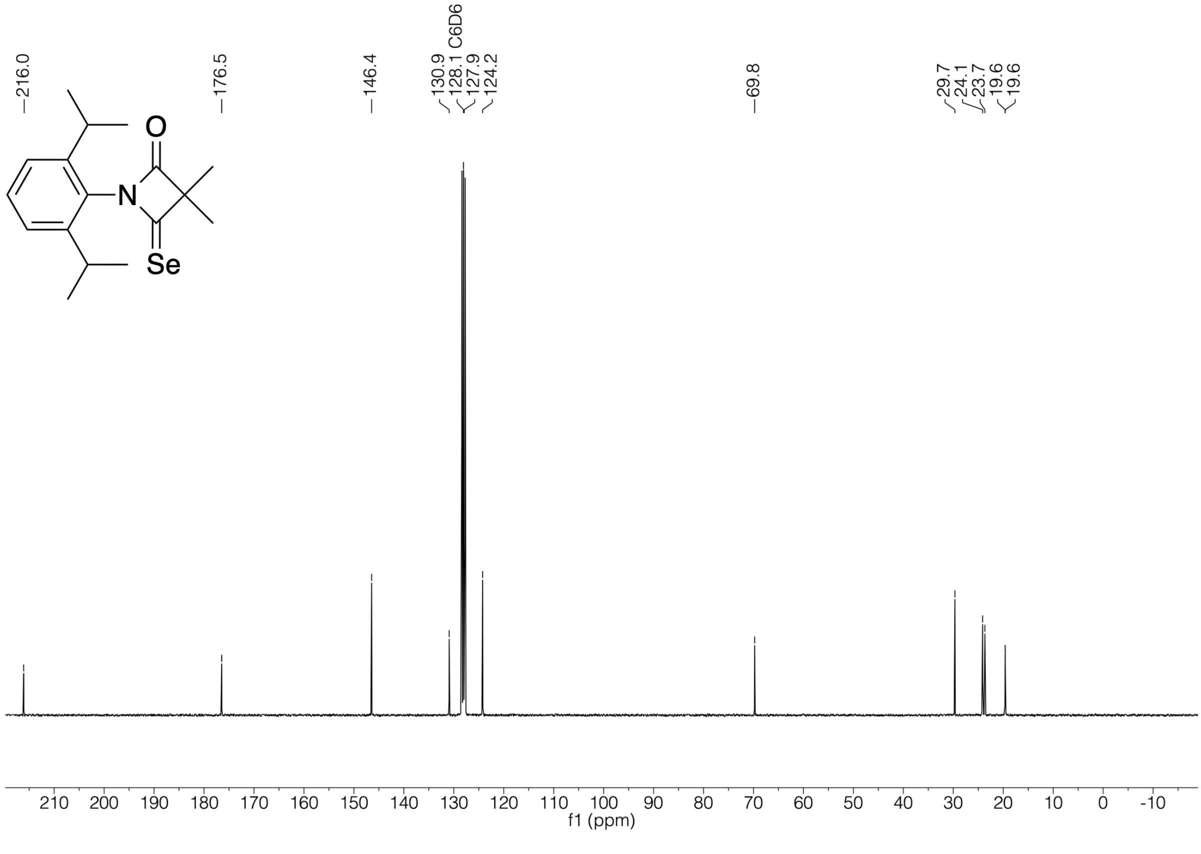

Figure SF121. $^{13}C\{^{1}H\}$ NMR (75 MHz, C_6D_6, 298.0 K) spectrum of **Dipp-6-Me$_2$*Se**.

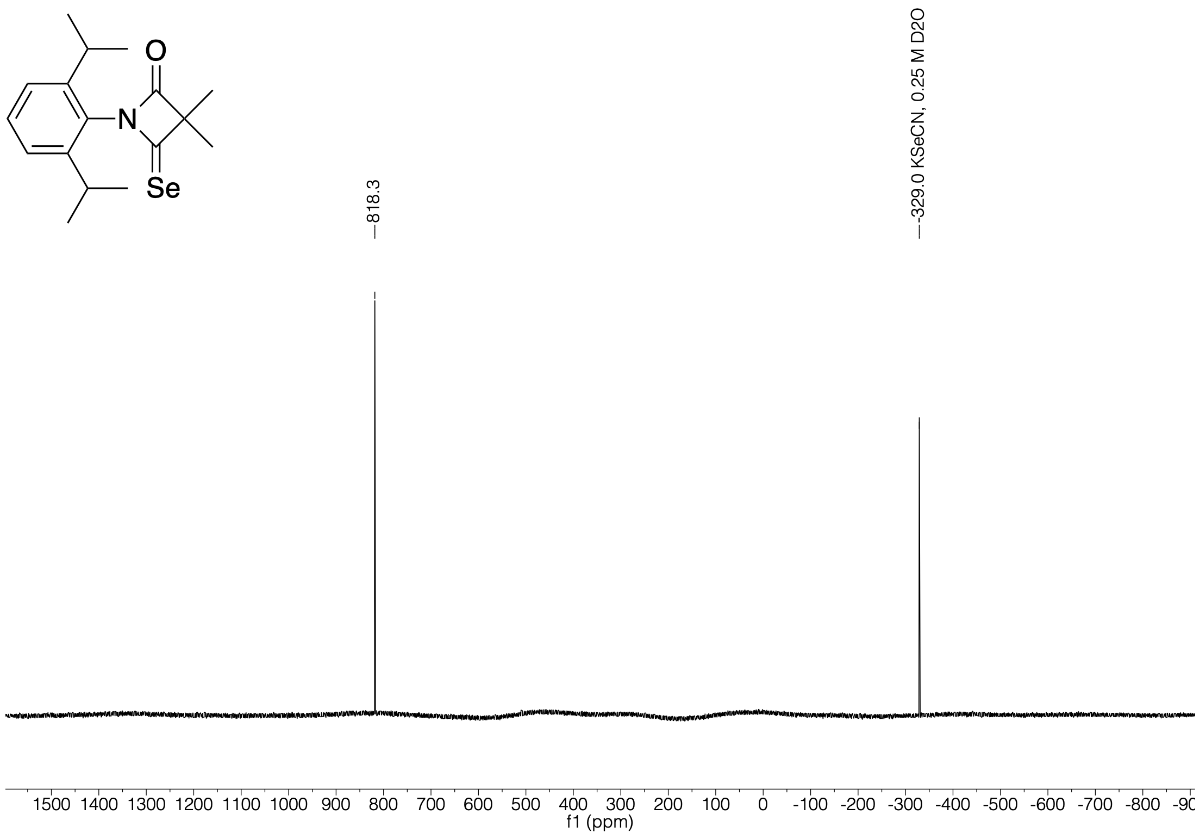

Figure SF122. $^{77}Se\{^1H\}$ NMR (114 MHz, acetone-d_6, 298.0 K) spectrum of **Dipp-6-Me$_2$*Se**.

Dipp-6-Ph$_2$*Se: Precursor used: **Dipp-5-Ph$_2$** (234 mg, 0.5 mmol) for method A and **Dipp-6-Ph$_2$*HCl** (209 mg, 0.5 mmol) method B. Yellow, air-stable powder. Yield: 222 mg, 96% (method A) / 186 mg, 81% (method B). ^1H NMR (300 MHz, C_6D_6, 298.0 K): δ = 7.98–7.91 (m, 4H; CH_{aryl}), 7.14–6.98 (m, 9H; CH_{aryl}), 3.03 (sept, J = 7 Hz, 2H; CH_{iPr}), 1.08 (d, J = 7 Hz, 6H; $CH_{3\text{-}iPr}$), 1.05 (d, J = 7 Hz, 6H; $CH_{3\text{-}iPr}$) ppm. $^{13}C\{^1H\}$ NMR (75 MHz, C_6D_6, 298.0 K): δ = 217.4 (s; C=Se), 173.4 (s; C=O), 146.7 (s; $C_{Dipp\text{-}ortho}$), 137.0 (s; C_{aryl}), 131.0 (s; C_{aryl}), 129.1 (s; C_{aryl}), 128.8 (s; C_{aryl}), 127.8 (s; C_{aryl}), 124.3 (s; C_{aryl}), 82.6 (s; C(*C*(Ph)$_2$)C), 29.6 (s; CH_{iPr}), 24.3 (s; $CH_{3\text{-}iPr}$), 23.6 (s; $CH_{3\text{-}iPr}$) ppm [Some of the expected signals for aryl carbon atoms are not observed due to isochrony as well as overlapping with the solvent signal.]. $^{77}Se\{^1H\}$ NMR (114 MHz, acetone-d_6, 298.0 K): δ = 936 (s; C=Se) ppm. MS (EI, 70 eV, 100 °C) *m/z* (%): 460 (2) $[M]^{+\cdot}$, 390 (6), 258 (11), 194 (100), 165 (32). Elemental analysis calcd for $C_{27}H_{27}NOSe$: C 70.42, H 5.91, N 3.04, found: C 70.52, H 6.18, N 2.96.

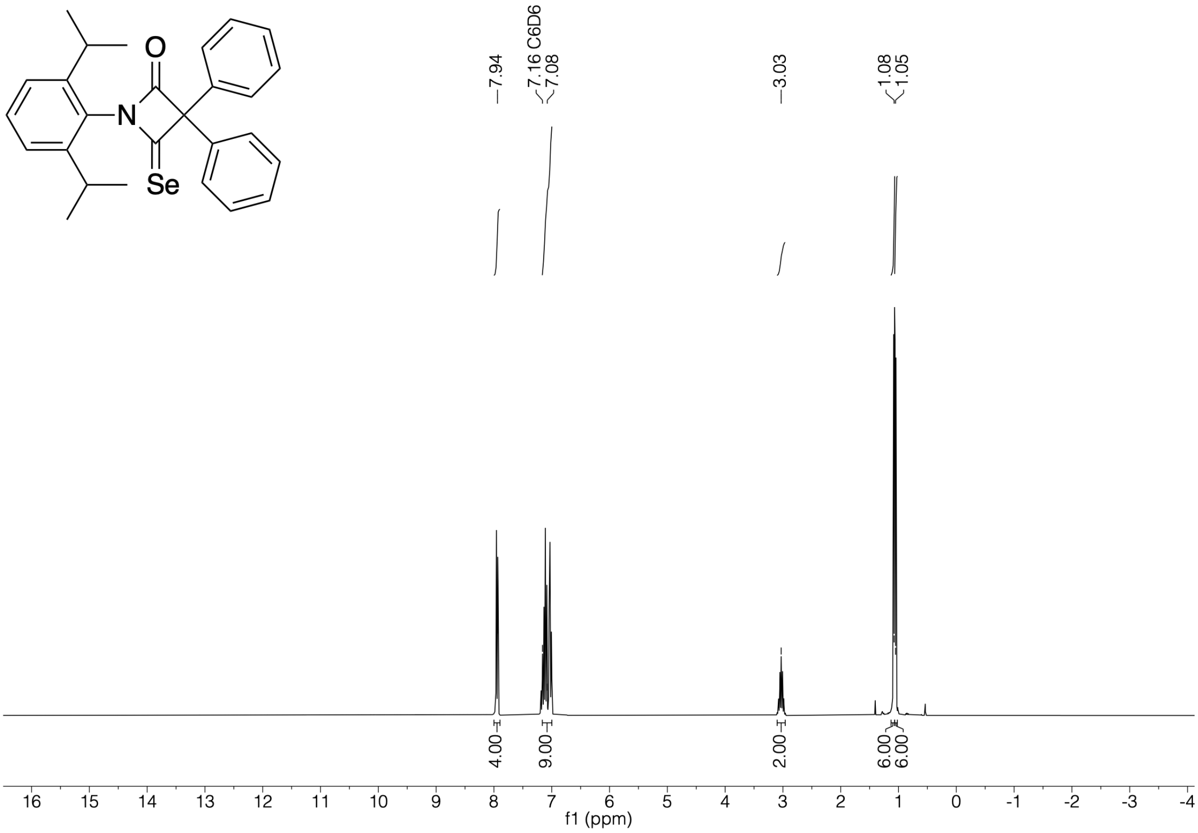

Figure SF123. ^{1}H NMR (300 MHz, C_6D_6, 298.0 K) spectrum of **Dipp-6-Ph$_2$*Se**.

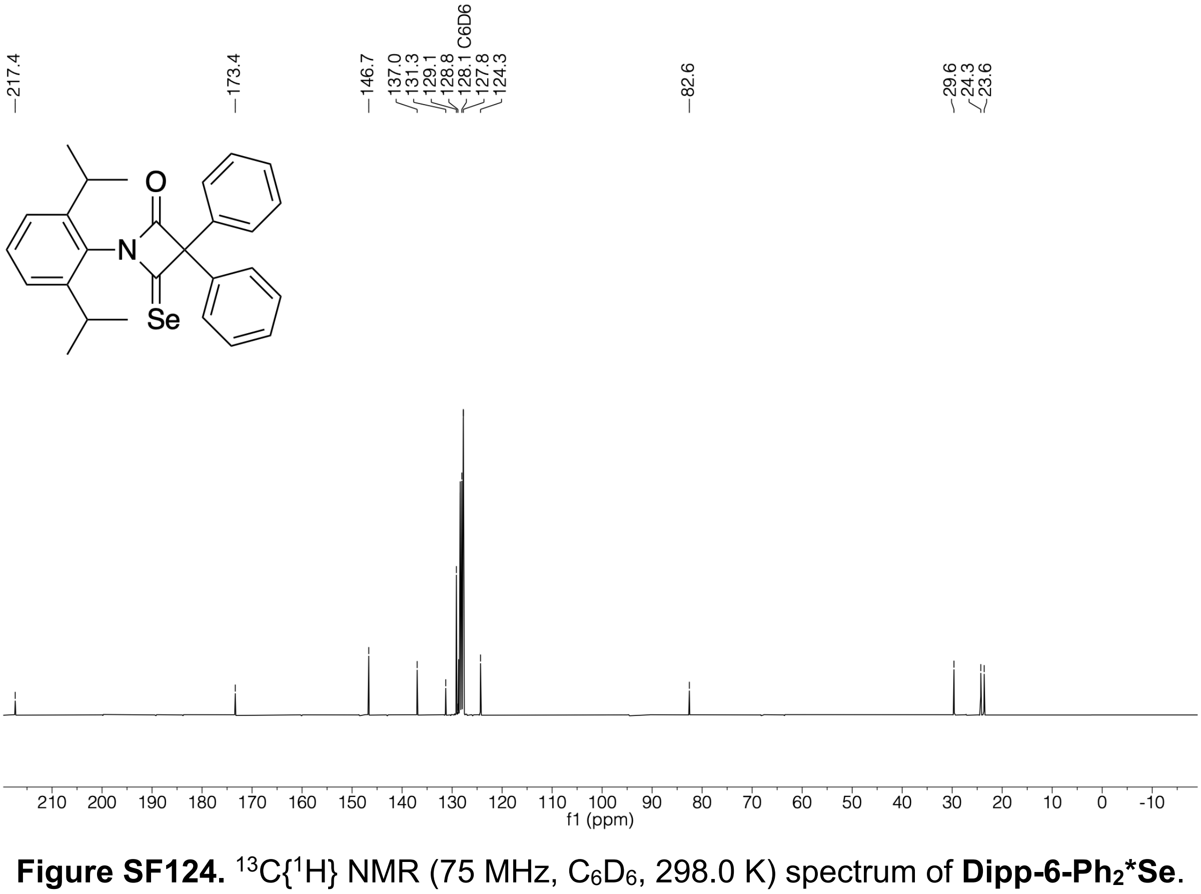

Figure SF124. $^{13}C\{^{1}H\}$ NMR (75 MHz, C_6D_6, 298.0 K) spectrum of **Dipp-6-Ph$_2$*Se**.

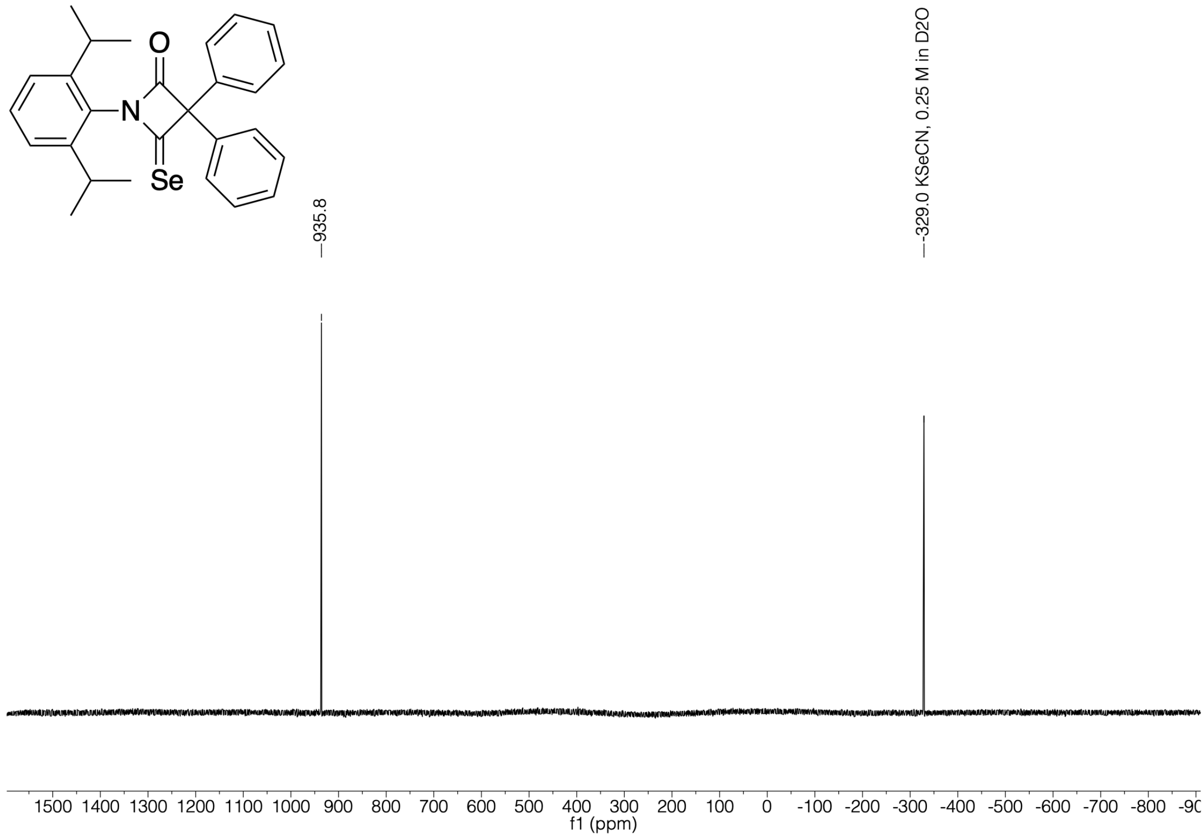

Figure SF125. $^{77}Se\{^1H\}$ NMR (114 MHz, acetone-d$_6$, 298.0 K) spectrum of **Dipp-6-Ph$_2$*Se**.

Dipp-6-sCy*Se: Precursor used: **Dipp-5-sCy** (192 mg, 0.5 mmol) for method A. Yellow, air-stable powder. Yield: 168 mg, 89% (method A). ^1H NMR (300 MHz, C_6D_6, 298.0 K): δ = 7.15–7.11 (m, 1H; $CH_{aryl\text{-}para}$), 7.05–6.99 (m, 2H; $CH_{ary\text{-}ortho}$), 3.07 (sept, J = 7 Hz, 2H; CH_{iPr}), 2.13–2.00 (m, 1H; CH_{aliph}), 1.78–1.70 (m, 2H; CH_{aliph}), 1.68–1.60 (m, 2H; CH_{aliph}), 1.53–1.44 (m, 2H; CH_{aliph}), 1.37–1.29 (m, 2H; CH_{aliph}), 1.18 (d, J = 7 Hz, 6H; $CH_{3\text{-}iPr}$), 1.12 (d, J = 7 Hz, 6H; $CH_{3\text{-}iPr}$), 1.00–0.89 (m, 1H; CH_{aliph}) ppm. $^{13}C\{^1H\}$ NMR (75 MHz, C_6D_6, 298.0 K): δ = 213.7 (s; C=Se), 176.5 (s; C=O), 146.5 (s; $C_{Dipp\text{-}ortho}$), 130.9 (s; NC_{aryl}), 128.0 (s; $C_{aryl\text{-}para}$), 124.2 (s; $C_{aryl\text{-}meta}$), 73.4 (s; C(C_{sCy})C), 30.6 (s; C_{aliph}), 29.7 (s; CH_{iPr}), 25.2 (s; C_{aliph}), 24.2 (s; $CH_{3\text{-}iPr}$), 23.7 (s; $CH_{3\text{-}iPr}$), 23.0 (s; C_{aliph}) ppm. $^{77}Se\{^1H\}$ NMR (114 MHz, acetone-d$_6$, 298.0 K): δ = 848 (s; C=Se) ppm. MS (EI, 70 eV, 90 °C) m/z (%): 377 (32) $[M]^{+\cdot}$, 306 (31), 269 (76), 186 (49), 173 (100), 146 (13), 110 (27), 91 (11). Elemental analysis calcd for $C_{20}H_{27}NOSe$: C 63.82, H 7.23, N 3.72, found: C 64.02, H 7.36, N 7.89.

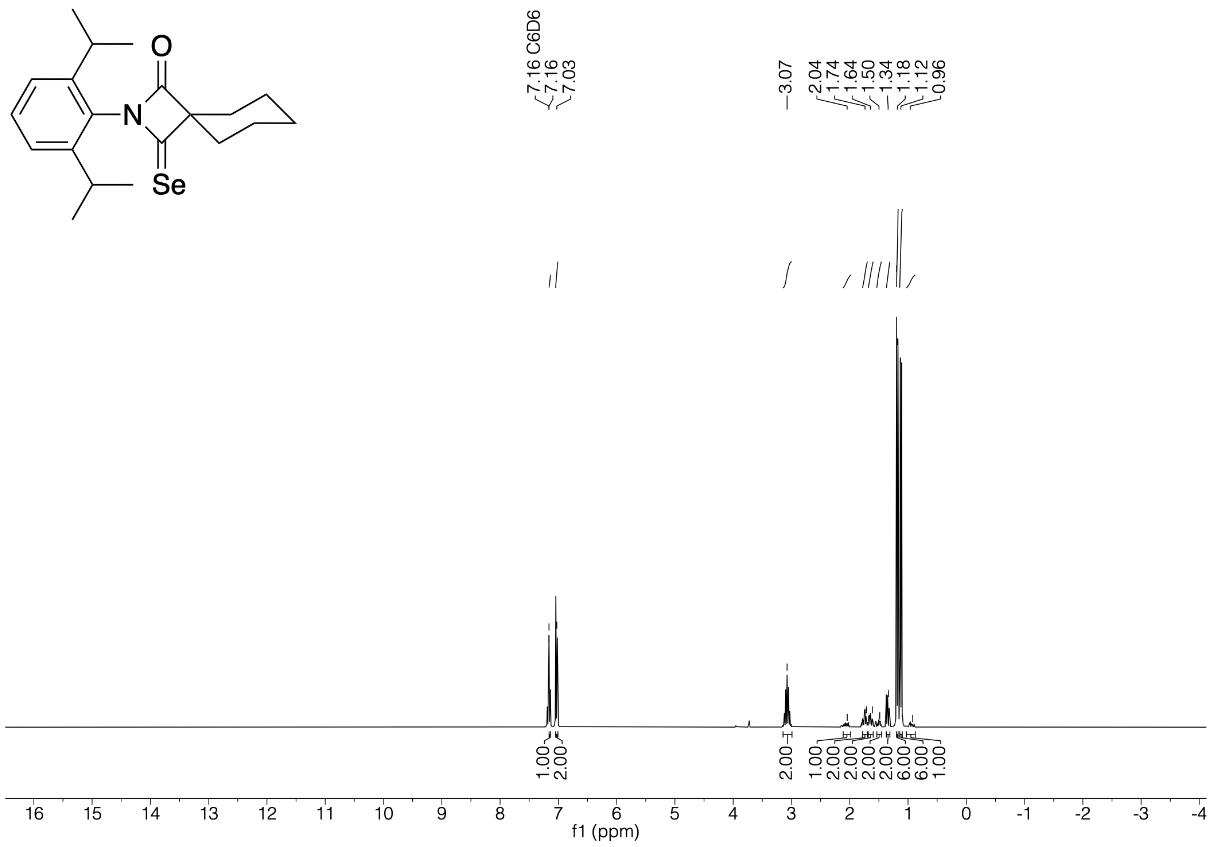

Figure SF126. ^{1}H NMR (300 MHz, C_6D_6, 298.0 K) spectrum of **Dipp-6-sCy*Se**.

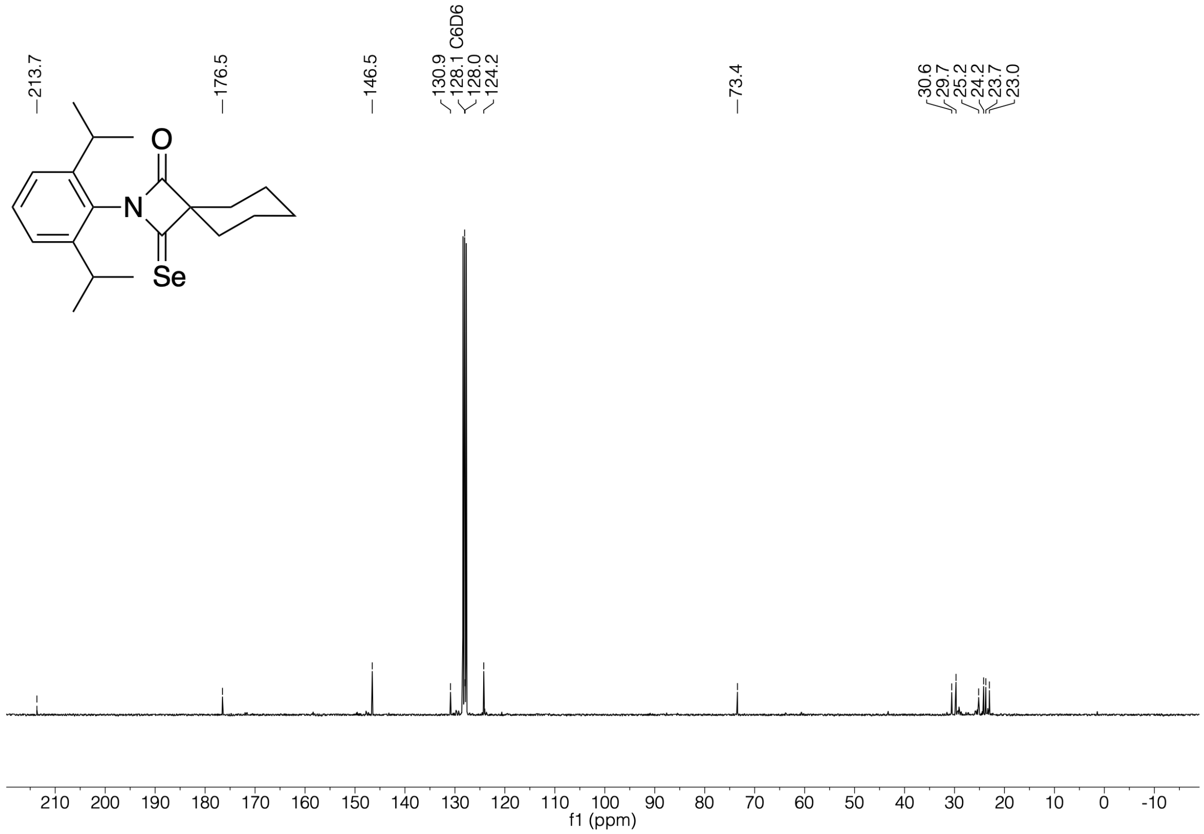

Figure SF137. $^{13}C\{^{1}H\}$ NMR (75 MHz, C_6D_6, 298.0 K) spectrum of **Dipp-6-sCy*Se**.

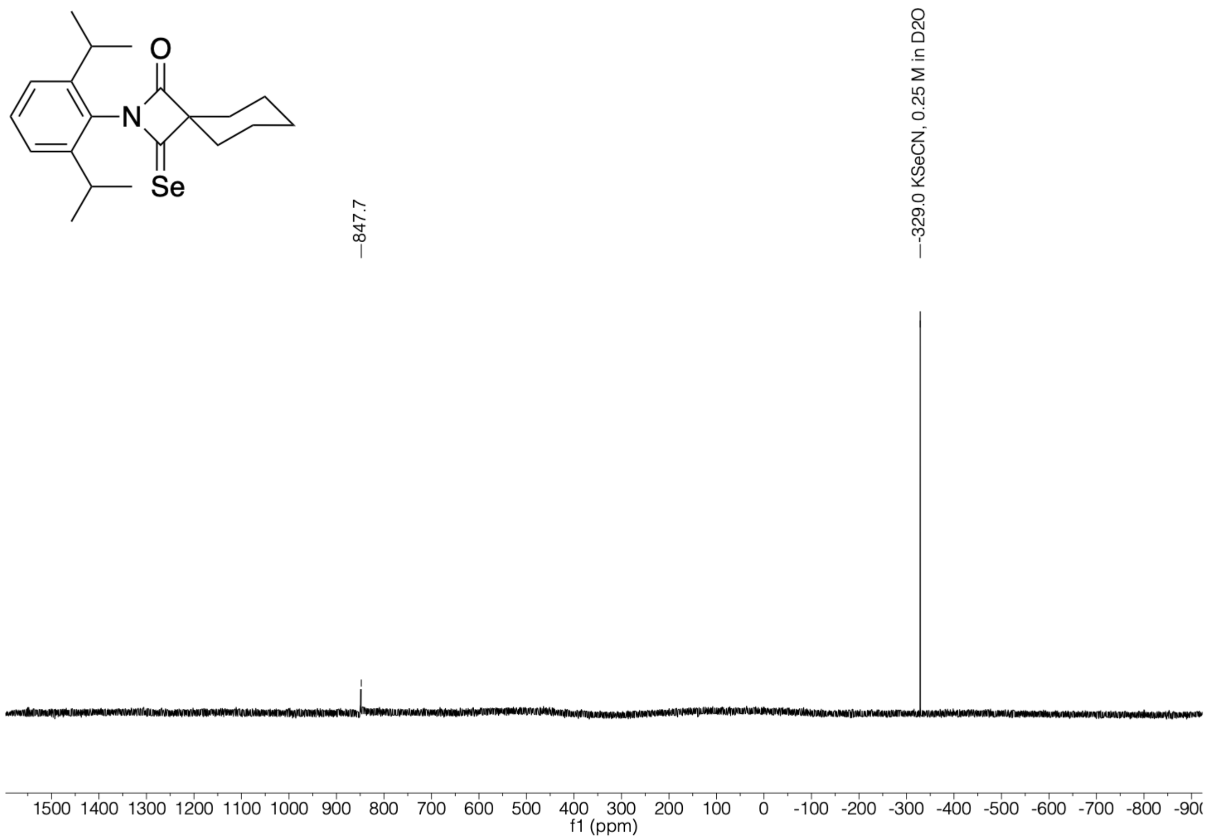

Figure SF138. $^{77}Se\{^{1}H\}$ NMR (114 MHz, acetone-d6, 298.0 K) spectrum of **Dipp-6-sCy*Se**.

i. Synthesis of sulfur adducts 6*S

Method A: The corresponding 1,3,4-oxadiazole-based precursor **5** (0.5 mmol, 1 eq.) and sulfur (32 mg, 1 mmol, 2 eq.) were suspended in 2 ml toluene at ambient temperature. After heating at 110 °C for 16 h, the yellow solution was filtered over silica and eluted with cyclohexane/diethyl ether 1:1. After removal of all volatiles, a bright yellow, air-stable powder was obtained.

Method B: Alternatively, the sulfur adducts **6*S** can be synthesized by reaction of sulfur with the carbenes generated in situ using the precursors **6*HCl** and NaHMDS as a strong base. The precursors of type **6*HCl** (0.5 mmol, 1 eq.) and sulfur (32 mg, 1 mmol, 2 eq.) were suspended in 10 ml of tetrahydrofuran followed by the addition 0.6 ml of NaHMDS in THF (1 M, 0.6 mmol, 1.2 eq.) under stirring at -80 °C. After 2 h the cold bath was removed and stirring was continued at ambient temperature for 14 h.

All volatiles were removed in vacuo and the resulting brown powder was suspended in 2 ml of diethyl ether, filtered over silica and eluted with cyclohexane/diethyl ether 1:1. After removal of all volatiles, a bright yellow, air-stable powder was obtained.

Ph-6-Me$_2$*S: Precursor used: **Ph-5-Me$_2$** (130 mg, 0.5 mmol) for method A and **Ph-6-Me$_2$*HCl** (105 mg, 0.5 mmol) method B. Bright yellow, air-stable powder. Yield: 95 mg, 93% (method A) / 91 mg, 89% (method B). ^{1}H NMR (300 MHz, C_6D_6, 298.0 K): δ = 8.34–8.26 (m, 2H; CH_{aryl}), 7.06–6.99 (m, 2H; CH_{aryl}), 6.92–6.82 (m, 1H; $CH_{aryl\text{-}para}$), 1.01 (s, 6H; $C(C(CH_3)_2)C$) ppm. $^{13}C\{^1H\}$ NMR (75 MHz, C_6D_6, 298.0 K): δ = 211.9 (s; C=S), 174.4 (s; C=O), 136.0 (s; NC_{aryl}), 129.2 (s; $C_{aryl\text{-}meta}$), 127.5 (s; $C_{aryl\text{-}para}$), 120.2 (s; $C_{aryl\text{-}ortho}$), 67.6 (s; $C(\mathit{C}(CH_3)_2)C$), 19.0 (s; $C(C(\mathit{C}H_3)_2)C$) ppm. MS (EI, 70 eV, 40 °C) *m/z* (%): 205 (13) $[M]^{+\cdot}$, 177 (28), 145 (100), 130 (26), 77 (28). Elemental analysis calcd for $C_{11}H_{11}NOS$: C 64.36, H 5.40, N 6.82, S 15.62, found: C 64.51, H 5.45, N 6.96, S 15.73.

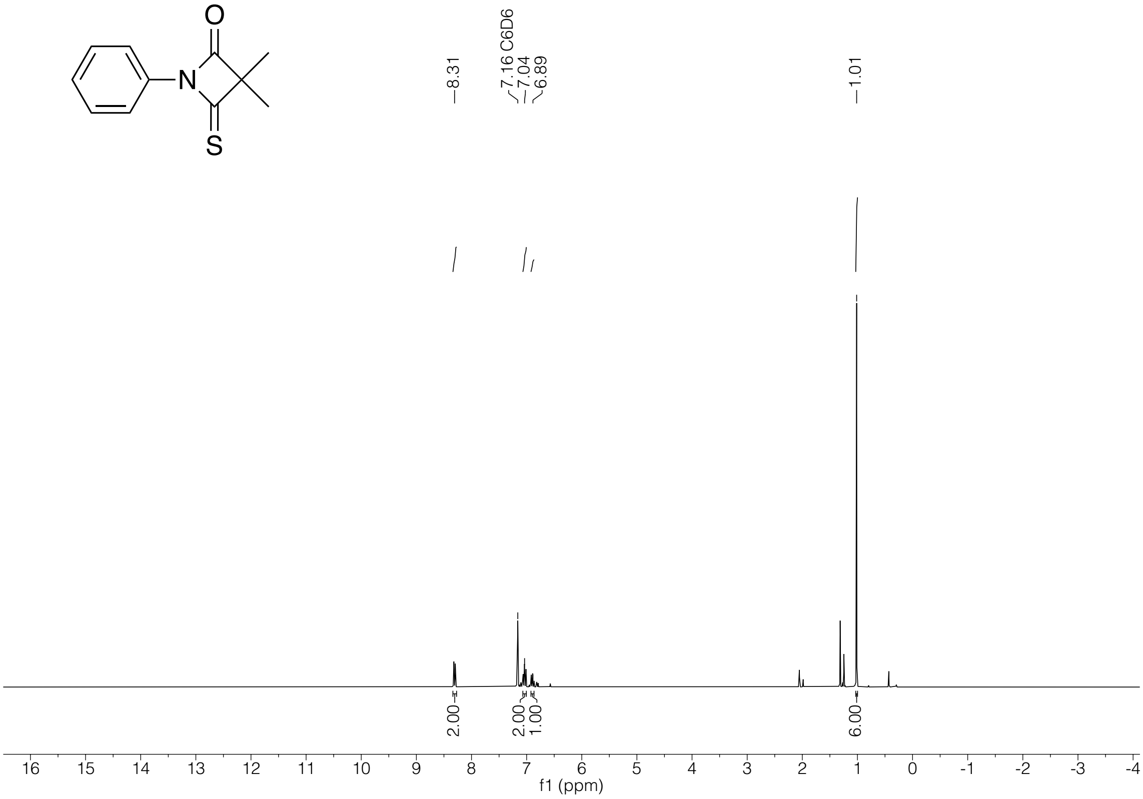

Figure SF129. ^{1}H NMR (300 MHz, C_6D_6, 298.0 K) spectrum of **Ph-6-Me$_2$*S**.

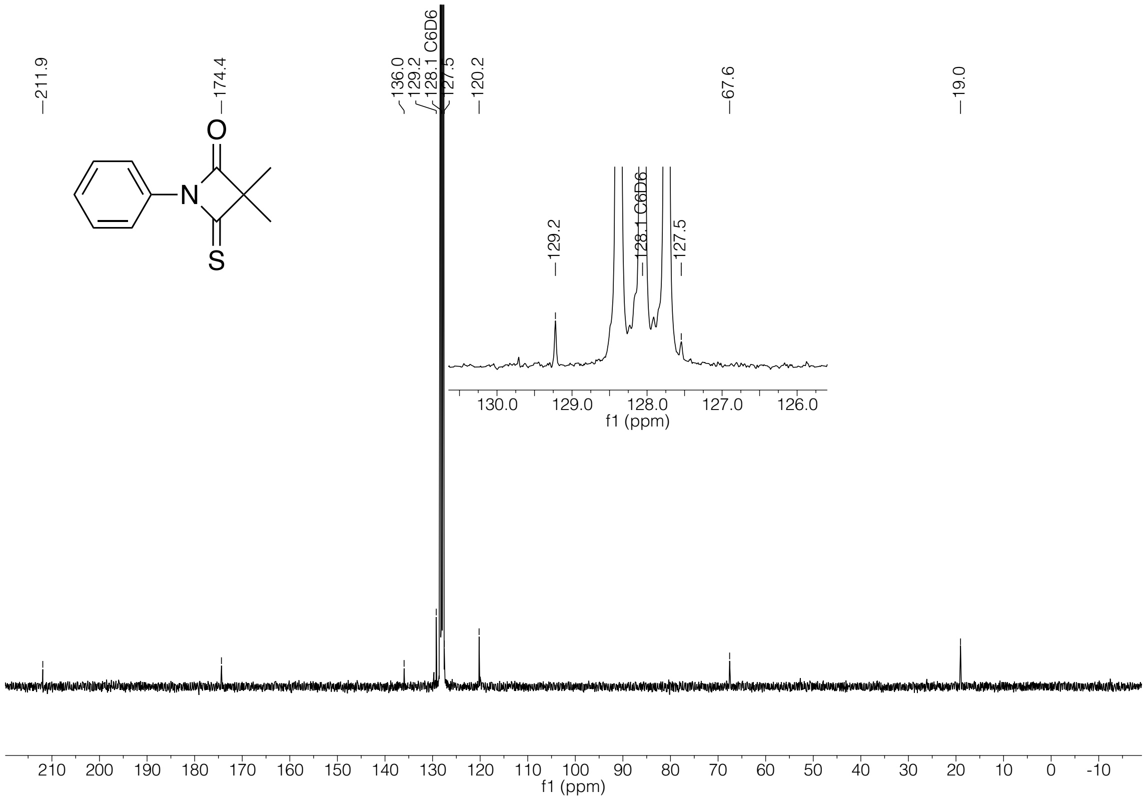

Figure SF130. $^{13}C\{^1H\}$ NMR (75 MHz, C_6D_6, 298.0 K) spectrum of **Ph-6-Me$_2$*S**.

Ph-6-sCy*S: Precursor used: **Ph-5-sCy** (150 mg, 0.5 mmol) for method A. Bright yellow, air-stable powder. Yield: 103 mg, 84% (method A). ^1H NMR (300 MHz, C_6D_6, 298.0 K): δ = 7.65–7.59 (m, 2H; CH_{aryl}), 7.13–7.05 (m, 2H; CH_{aryl}), 6.88–6.81 (m, 1H; CH_{aryl}), 1.83–1.76 (m, 3H; CH_{aliph}), 1.55–1.49 (m, 2H; CH_{aliph}), 1.46–1.34 (m, 2H; CH_{aliph}), 1.22–1.15 (m, 1H; CH_{aliph}), 1.00–0.93 (m, 2H; CH_{aliph}) ppm. $^{13}C\{^1H\}$ NMR (75 MHz, C_6D_6, 298.0 K): δ = 213.7 (s; C=S), 175.0 (s; C=O), 137.7 (s; NC_{aryl}), 129.6 (s; $C_{aryl\text{-}meta}$), 124.4 (s; $C_{aryl\text{-}para}$), 117.0 (s; $C_{aryl\text{-}ortho}$), 74.3 (s; C(C_{sCy})C), 29.0 (s; C_{aliph}), 25.4 (s; C_{aliph}), 23.2 (s; C_{aliph}) ppm. MS (EI, 70 eV, 60 °C) *m/z* (%): 245 (17) $[M]^{+\cdot}$, 231 (19), 203 (33), 185 (76), 119 (100). Elemental analysis calcd for $C_{14}H_{17}NOS$: C 68.54, H 6.16, N 5.71, S 13.07, found: C 68.62, H 6.22, N 5.95, S 12.96.

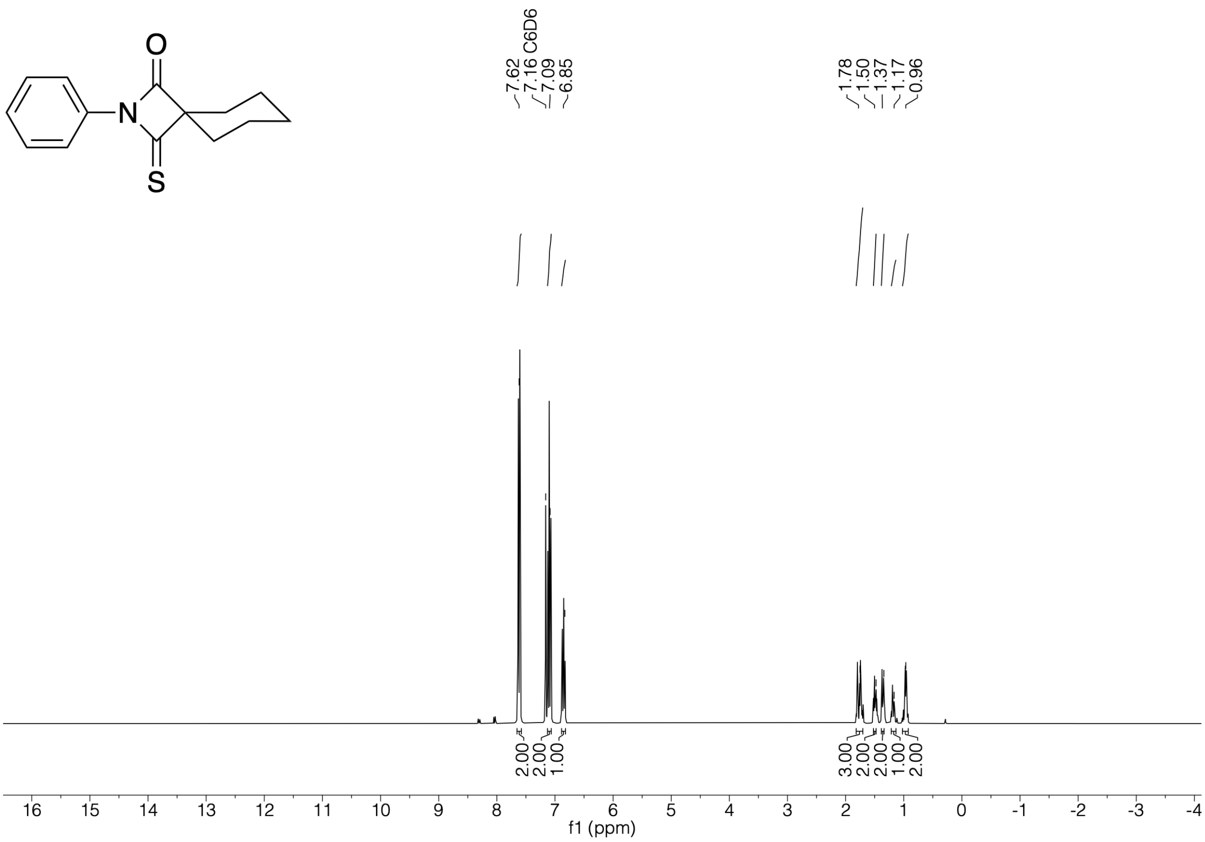

Figure SF131. ^{1}H NMR (300 MHz, C_6D_6, 298.0 K) spectrum of **Ph-6-sCy*S**.

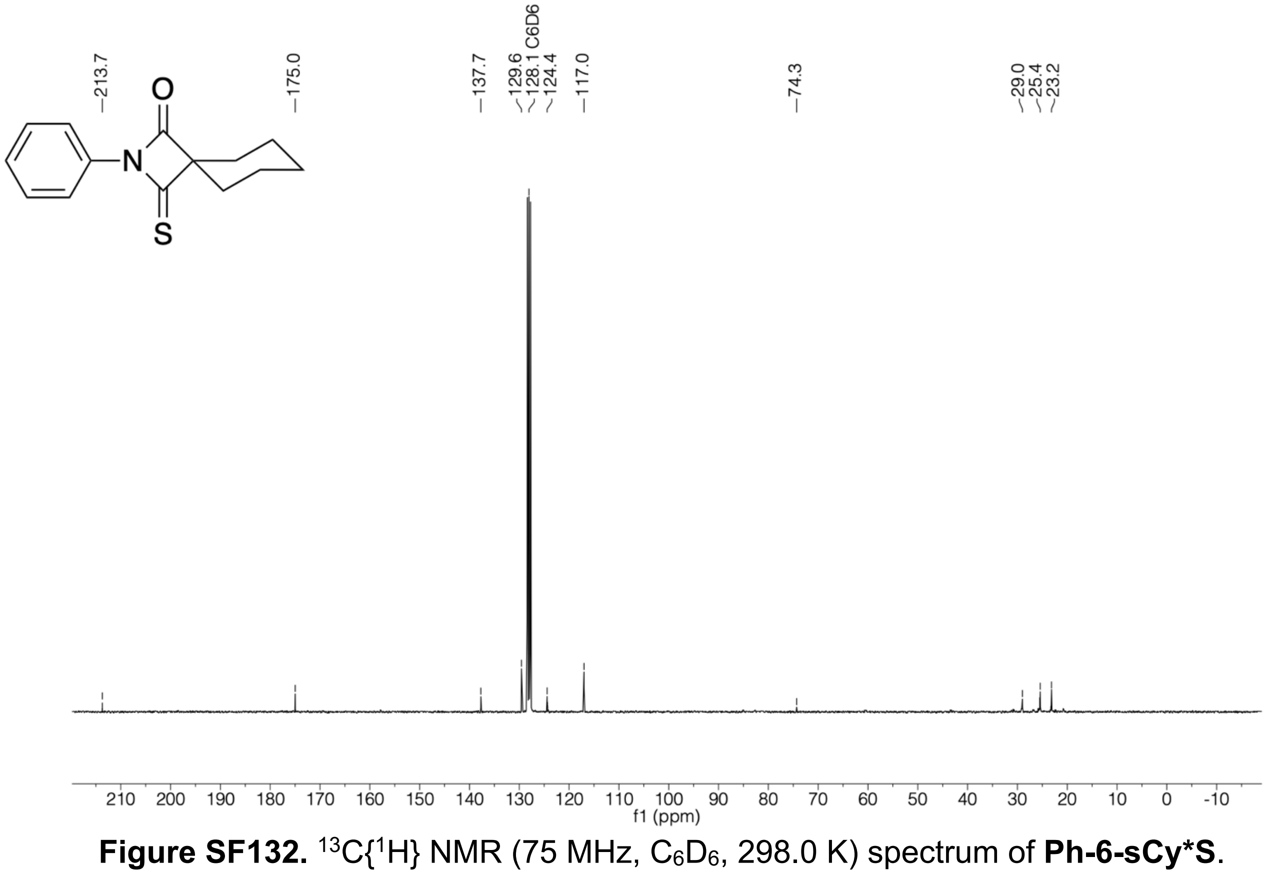

Figure SF132. $^{13}C\{^{1}H\}$ NMR (75 MHz, C_6D_6, 298.0 K) spectrum of **Ph-6-sCy*S**.

Mes-6-Me$_2$*S: Precursor used: **Mes-5-Me$_2$** (151 mg, 0.5 mmol) for method A and **Mes-6-Me$_2$*HCl** (126 mg, 0.5 mmol) method B. Bright yellow, air-stable powder. Yield: 115 mg, 93% (method A) / 109 mg, 88% (method B). ^{1}H NMR (300 MHz, C_6D_6, 298.0 K): δ = 6.58 (d, J = 1 Hz, 2H; CH_{meta}), 2.05 (s, 6H; $CH_{3\text{-}ortho}$), 1.99 (s, 3H; $CH_{3\text{-}para}$), 1.25 (s, 6H; $C(C(CH_3)_2)C$) ppm. $^{13}C\{^{1}H\}$ NMR (75 MHz, C_6D_6, 298.0 K): δ = 215.0 (s; C=S), 175.3 (s; C=O), 139.7 (s; NC_{aryl}), 135.7 (s; $C_{aryl\text{-}para}$), 129.4 (s; $C_{aryl\text{-}ortho}$), 127.2 (s; $C_{aryl\text{-}meta}$), 65.7 (s; $C(\mathit{C}(CH_3)_2)C$), 21.0 (s; $CH_{3\text{-}para}$), 19.6 (s; $CH_{3\text{-}ortho}$), 17.8 (s; $C(C(\mathit{C}H_3)_2)C$) ppm. MS (EI, 70 eV, 80 °C) *m/z* (%): 247 (50) $[M]^{+\cdot}$, 187 (48), 177 (100), 144 (21). Elemental analysis calcd for $C_{14}H_{17}NOS$: C 67.98, H 6.93, N 5.66, S 12.96, found: C 68.04, H 7.10, N 5.58, S 12.88.

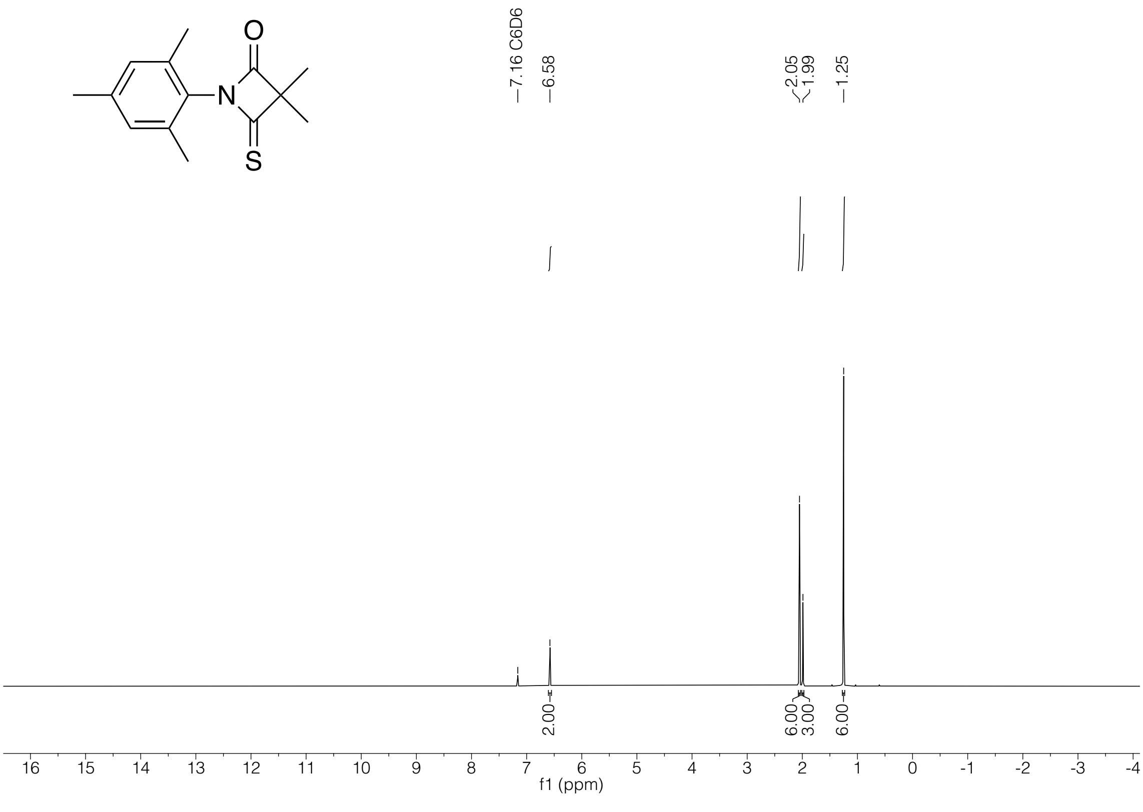

Figure SF133. ^{1}H NMR (300 MHz, C_6D_6, 298.0 K) spectrum of **Mes-6-Me$_2$*S**.

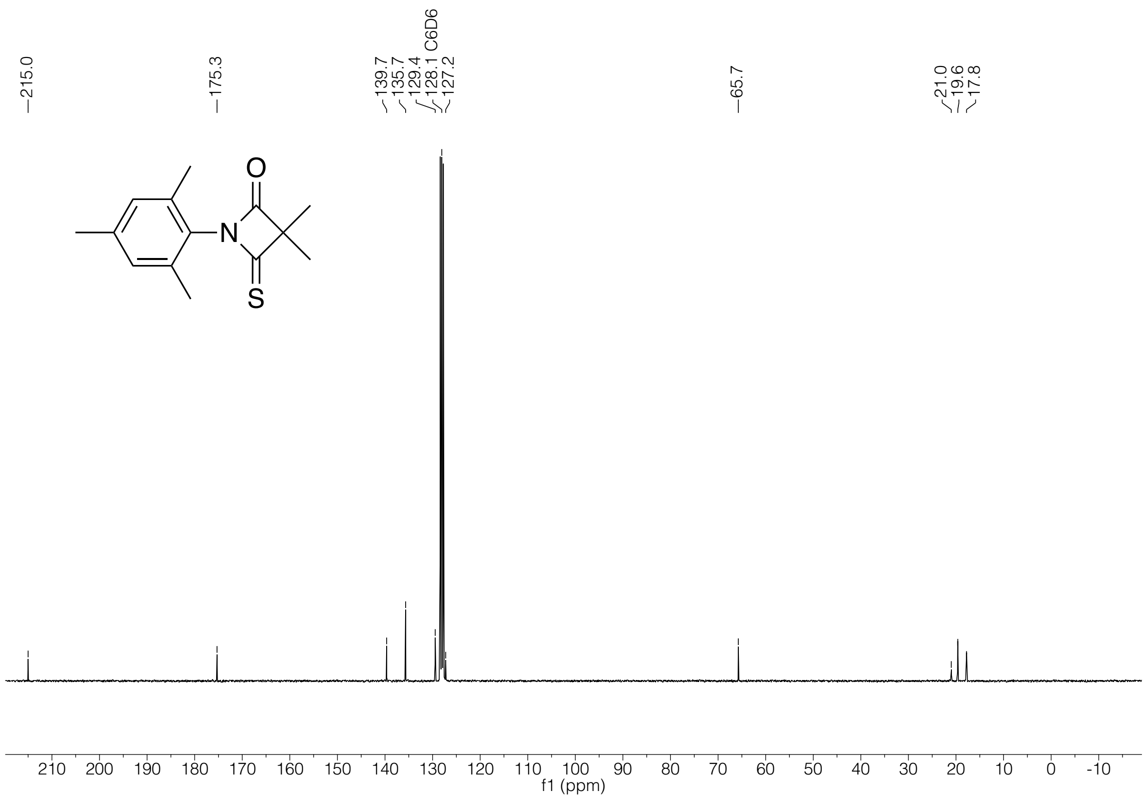

Figure SF134. $^{13}C\{^1H\}$ NMR (75 MHz, C_6D_6, 298.0 K) spectrum of **Mes-6-Me$_2$*S**.

Mes-6-Ph$_2$*S: Precursor used: **Mes-5-Ph$_2$** (213 mg, 0.5 mmol) for method A and **Mes-6-Ph$_2$*HCl** (188 mg, 0.5 mmol) method B. Bright yellow, air-stable powder. Yield: 171 mg, 92% (method A) / 160 mg, 86% (method B). ^{1}H NMR (300 MHz, C_6D_6, 298.0 K): δ = 7.93–7.88 (m, 3H; CH_{aryl}), 7.14–6.98 (m, 7H; CH_{aryl}), 6.51 (d, J = 1 Hz, 2H; CH_{meta}), 2.03 (s, 6H; $CH_{3\text{-ortho}}$), 1.94 (s, 3H; $CH_{3\text{-para}}$) ppm. $^{13}C\{^1H\}$ NMR (75 MHz, C_6D_6, 298.0 K): δ = 208.7 (s; C=S), 171.9 (s; C=O), 140.1 (s; NC_{aryl}), 137.4 (s; C-C_{aryl}), 135.9 (s; C-C_{aryl}), 129.5 (s; C_{aryl}), 129.2 (s; C_{aryl}), 128.6 (s; C_{aryl}), 127.6 (s; C_{aryl}), 127.1 (s; C_{aryl}), 79.2 (s; C(*C*(Ph)$_2$)C), 20.9 (s; $CH_{3\text{-para}}$), 18.0 (s; $CH_{3\text{-ortho}}$) ppm. MS (EI, 70 eV, 100 °C) *m/z* (%): 371 (3) $[M]^{+\cdot}$, 210 (13), 194 (100), 165 (38). Elemental analysis calcd for $C_{24}H_{21}NOS$: C 77.60, H 5.70, N 3.77, S 8.63, found: C 77.72, H 5.58, N 3.83, S 8.52.

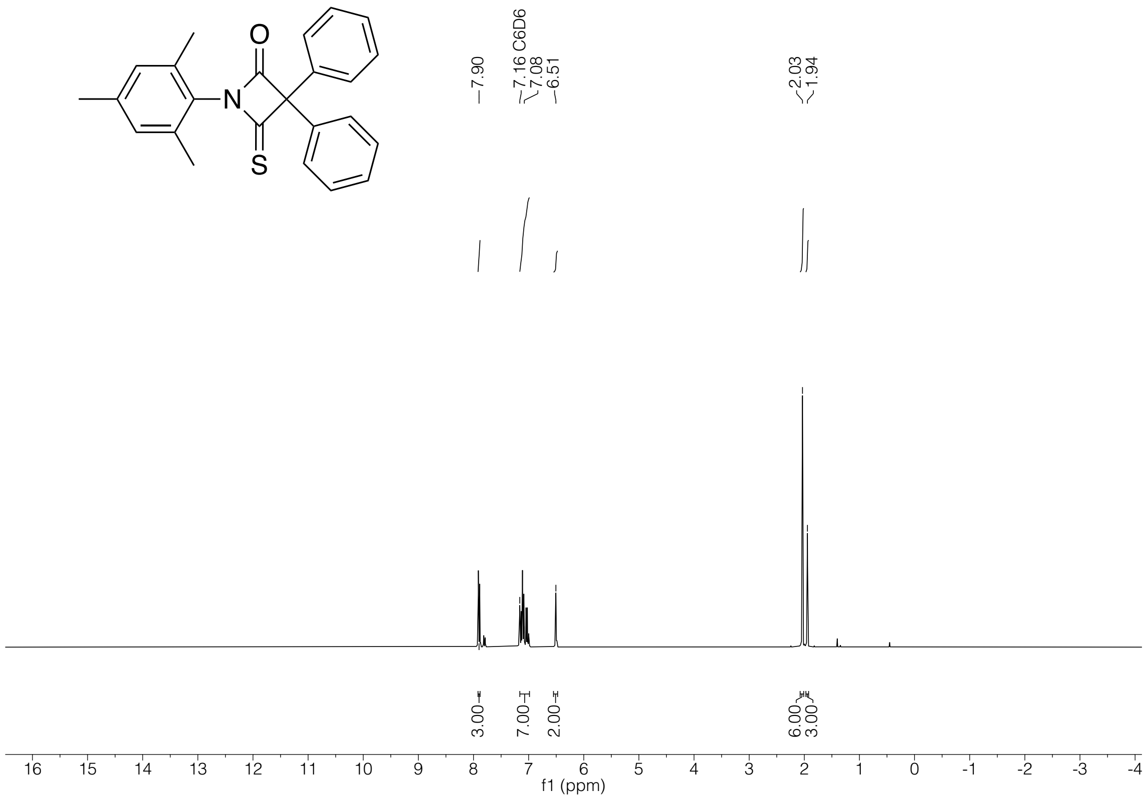

Figure SF135. ^{1}H NMR (300 MHz, C_6D_6, 298.0 K) spectrum of **Mes-6-Ph₂*S**.

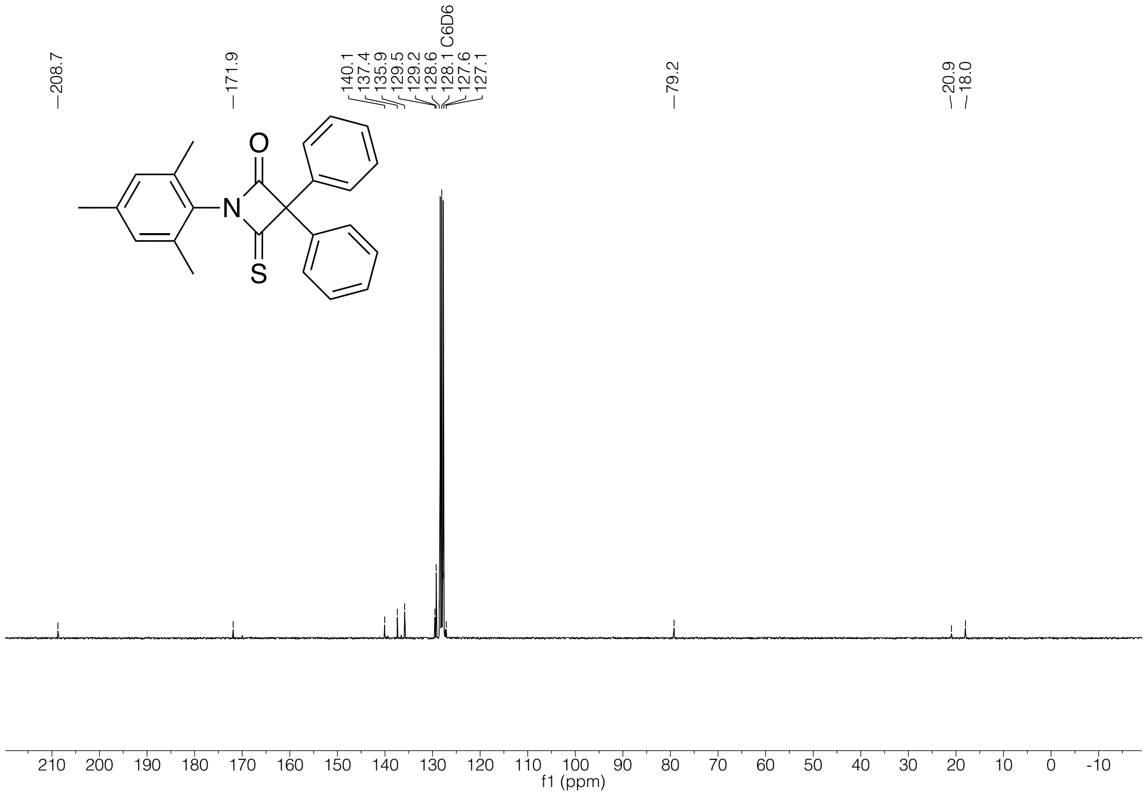

Figure SF136. $^{13}C\{^1H\}$ NMR (75 MHz, C_6D_6, 298.0 K) spectrum of **Mes-6-Ph_2*S**.

Mes-6-sCy*S: Precursor used: **Mes-5-sCy** (171 mg, 0.5 mmol) for method A. Bright yellow, air-stable powder. Yield: 129 mg, 90% (method A). ^{1}H NMR (300 MHz, C_6D_6, 298.0 K): δ = 6.59 (d, J = 1 Hz, 2H; CH_{meta}), 2.08 (s, 6H; $CH_{3\text{-}ortho}$), 2.00 (s, 3H; $CH_{3\text{-}para}$), 1.90–1.79 (m, 4H; CH_{aliph}), 1.73–1.58 (m, 4H; CH_{aliph}), 1.41–1.34 (m, 1H; CH_{aliph}), 1.19–1.09 (m, 1H; CH_{aliph}) ppm. ^{13}C{^{1}H} NMR (75 MHz, C_6D_6, 298.0 K): δ = 214.5 (s; C=S), 175.3 (s; C=O), 139.6 (s; NC_{aryl}), 135.8 (s; $C_{aryl\text{-}meta}$), 129.4 (s; $C_{aryl\text{-}para}$), 127.3 (s; $C_{aryl\text{-}ortho}$), 69.8 (s; C(C_{sCy})C), 30.6 (s; C_{aliph}), 25.2 (s; C_{aliph}), 23.1 (s; C_{aliph}), 21.0 (s; $CH_{3\text{-}para}$), 17.9 (s; $CH_{3\text{-}ortho}$) ppm. MS (EI, 70 eV, 80 °C) *m/z* (%): 287 (63) $[M]^{+\cdot}$, 227 (100), 177 (89), 144 (15), 110 (15). Elemental analysis calcd for $C_{17}H_{21}NOS$: C 71.04, H 7.36, N 4.87, S 11.15, found: C 70.96, H 7.48, N 4.62, S 11.04.

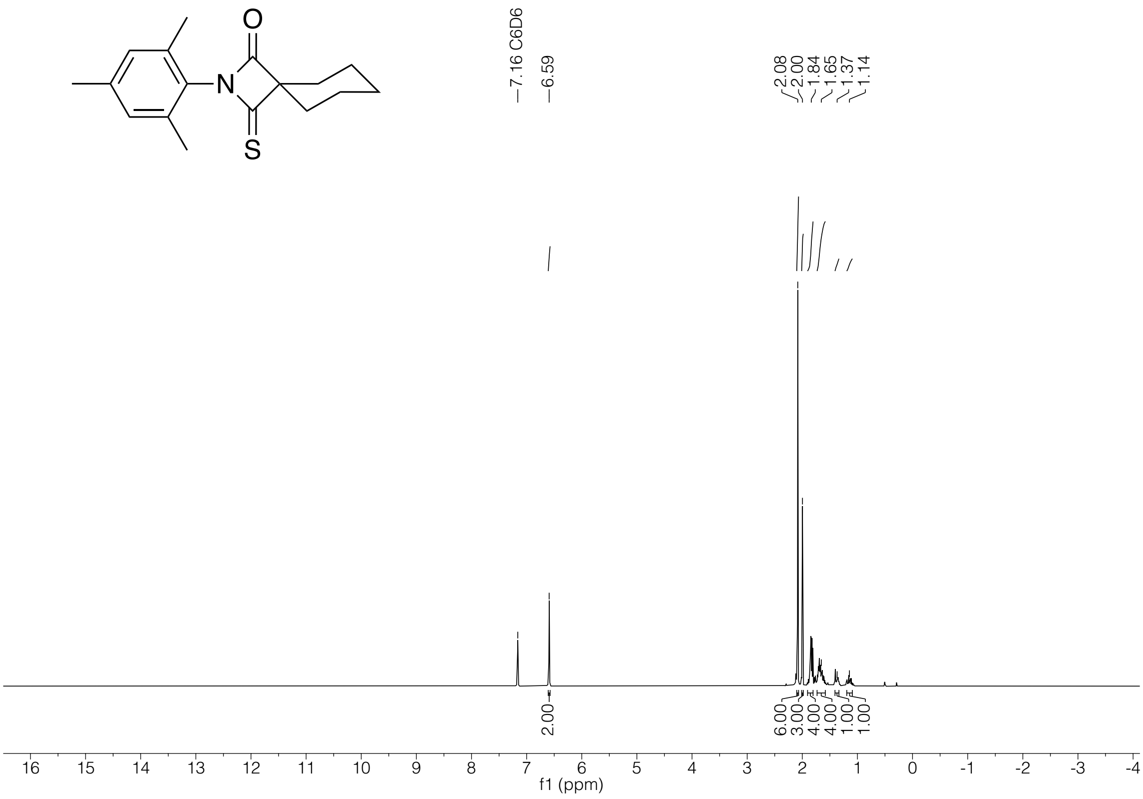

Figure SF137. ^{1}H NMR (300 MHz, C_6D_6, 298.0 K) spectrum of **Mes-6-sCy*S**.

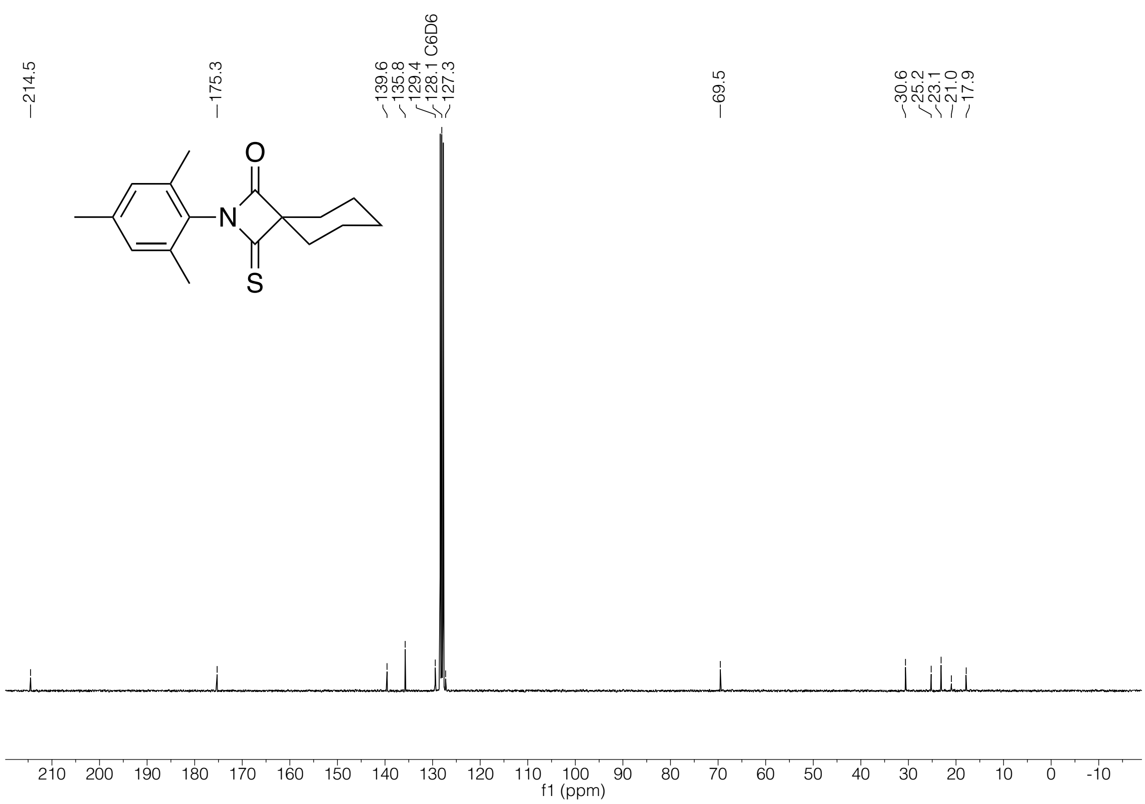

Figure SF138. $^{13}C\{^{1}H\}$ NMR (75 MHz, C_6D_6, 298.0 K) spectrum of **Mes-6-sCy*S**.

Dipp-6-Me$_2$*S: Precursor used: **Dipp-5-Me$_2$** (172 mg, 0.5 mmol) for method A and **Dipp-6-Me$_2$*HCl** (147 mg, 0.5 mmol) method B. Bright yellow, air-stable powder. Yield: 99 mg, 95% (method A) / 95 mg, 91% (method B). ^{1}H NMR (300 MHz, C_6D_6, 298.0 K): δ = 7.16–7.13 (m, 1H; $CH_{aryl\text{-}para}$), 7.04–6.99 (m, 2H; $CH_{aryl\text{-}meta}$), 3.01 (sept, J = 7 Hz, 2H; CH_{iPr}), 1.28 (s, 6H; $C(C(CH_3)_2)C$), 1.15 (d, J = 7 Hz, 6H; $CH_{3\text{-}iPr}$), 1.12 (d, J = 7 Hz, 6H; $CH_{3\text{-}iPr}$) ppm. $^{13}C\{^{1}H\}$ NMR (75 MHz, C_6D_6, 298.0 K): δ = 216.7 (s; C=S), 176.4 (s; C=O), 146.9 (s; $C_{aryl\text{-}ortho}$), 130.8 (s; $NC_{\textit{aryl}}$), 127.1 (s; $C_{aryl\text{-}para}$), 124.2 (s; $C_{aryl\text{-}meta}$), 65.8 (s; $C(\textit{C}(CH_3)_2)C$), 29.6 (s; CH_{iPr}), 24.0 (s; $CH_{3\text{-}iPr}$), 23.6 (s; $CH_{3\text{-}iPr}$), 19.3 (s; $C(C(\textit{C}H_3)_2)C$) ppm. MS (EI, 70 eV, 80 °C) *m/z* (%): 289 (28) $[M]^{+\cdot}$, 261 (21), 229 (99), 218 (100), 204 (48), 186 (42), 173 (14), 144 (7). Elemental analysis calcd for $C_{17}H_{23}NOS$: C 70.55, H 8.01, N 4.84, S 11.08, found: C 70.62, H 8.14, N 5.86, S 10.95.

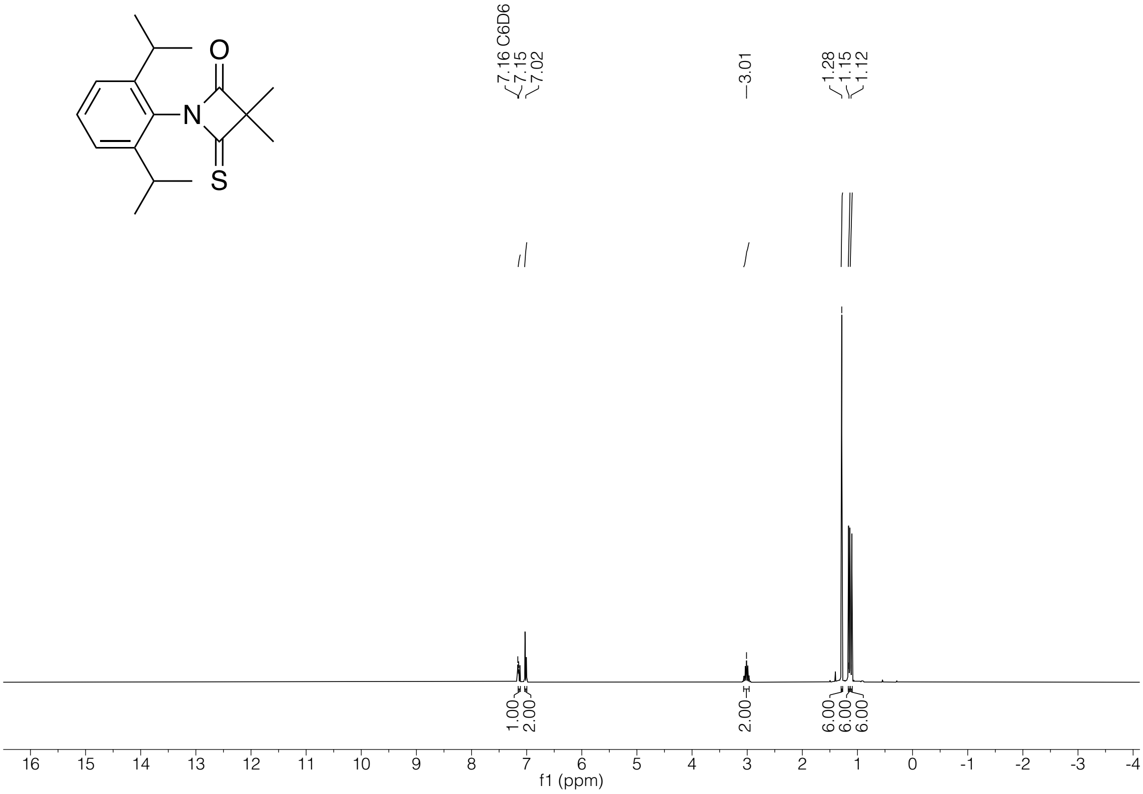

Figure SF139. ^{1}H NMR (300 MHz, C_6D_6, 298.0 K) spectrum of **Dipp-6-Me$_2$*S**.

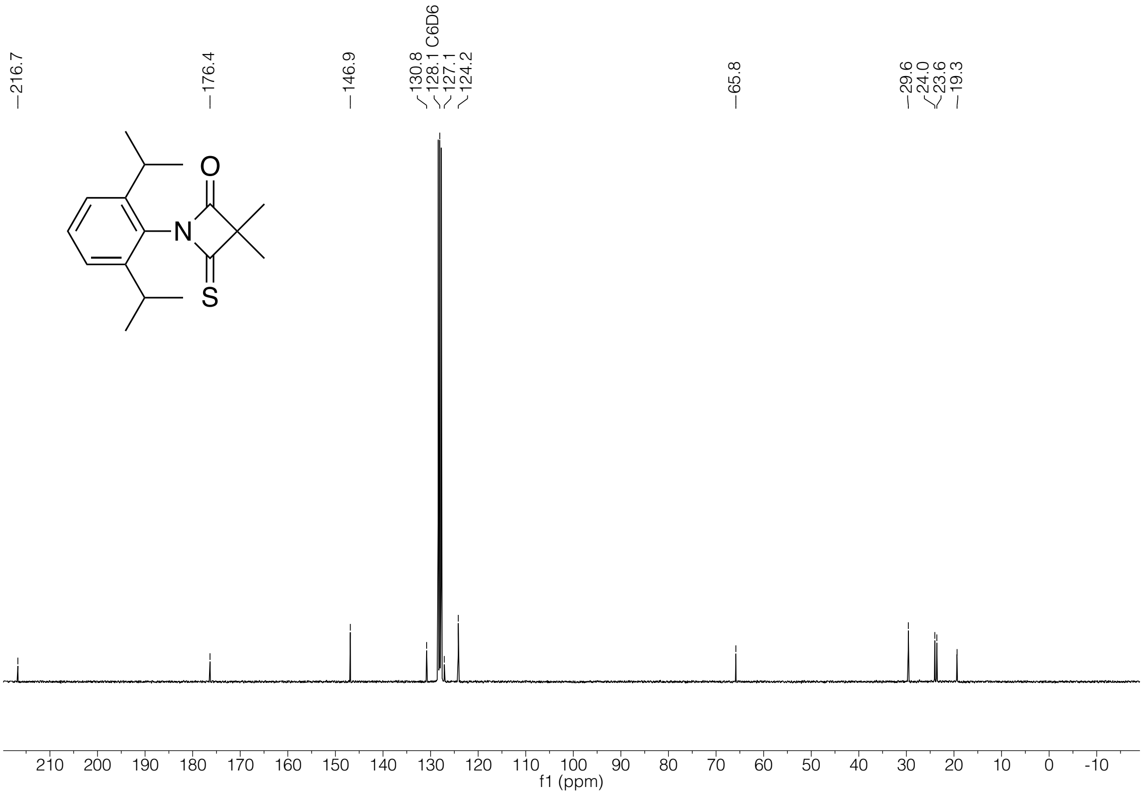

Figure SF140. $^{13}C\{^{1}H\}$ NMR (75 MHz, C_6D_6, 298.0 K) spectrum of **Dipp-6-Me$_2$*S**.

j. Synthesis of iridium/rhodium 1,5-COD-complexes 6*[Ir/Rh(COD)Cl]

Method A: The corresponding 1,3,4-oxadiazole-based precursor **5** (0.5 mmol, 1 eq.) and 1,5-cycloocatadiene iridium chloride dimer (168 mg, 0.25 mmol, 0.5 eq.) or 1,5-cyclooctadiene rhodium dimer (123 mg, 0.25 mmol, 0.5 eq.) were suspended in 2 ml of toluene at ambient temperature. After heating at 110 °C for 16 h, the red solution was filtered through aluminum oxide 90 active neutral (activity stage I) and eluted with dichloromethane. The orange/red fraction was collected. The desired product was obtained by removing all volatiles in vacuo.

Method B: Alternatively, the 1,5-cyclooctadiene complexes **6*[Ir/Rh(COD)Cl]** can be synthesized by reaction of and 1,5-cycloocatadiene iridium/rhodium chloride dimer with the carbenes generated in situ using the precursors **6*HCl** and NaHMDS as a strong base. The precursors of the type **6*HCl** (0.5 mmol, 1 eq.) and 1,5-cycloocatadiene iridium chloride dimer (168 mg, 0.25 mmol, 0.5 eq.) or 1,5-cyclooctadiene rhodium dimer (123 mg, 0. 25 mmol, 0.5 eq.) were suspended in 10 ml tetrahydrofuran and then 0.6 ml NaHMDS in THF (1 M, 0.6 mmol, 1.2 eq.) was added while stirring at -80 °C. After 2 h, the cold bath was removed and stirring continued at ambient temperature for 14 h. All volatiles were removed in vacuo and the resulting brown powder was suspended in 2 ml of dichloromethane, filtered on aluminum oxide 90 active neutral (activity stage I) and eluted with dichloromethane. The orange/red fraction was collected. The desired product was obtained after removal of all volatiles in vacuo.

Ph-6-Ph$_2$*[Ir(COD)Cl]: Precursor used: **Ph-5-Ph$_2$** (192 mg, 0.5 mmol) for method A and **Ph-6-Ph$_2$*HCl** (167 mg, 0.5 mmol) method B. Red, block-shaped, air-stable crystals. Yield: 301 mg, 95% (method A) / 291 mg, 92% (method B). ^{1}H NMR (300 MHz, CD_2Cl_2, 298.0 K): δ = 8.76–8.69 (m, 2H; CH_{aryl}), 7.71–7.64 (m, 4H; CH_{aryl}), 7.60–7.50 (m, 3H; CH_{aryl}), 7.45–7.34 (m, 6H; CH_{aryl}), 5.46–5.36 (m, 2H; $COD_{ol.}$), 2.41–2.22 (m, 3H; $COD_{ol.\&al.}$), 2.20–1.89 (m, 5H; $COD_{al.}$), 1.88–1.71 (m, 2H; $COD_{al.}$) ppm. $^{13}C\{^1H\}$ NMR (75 MHz, CD_2Cl_2, 298.0 K): δ = 168.5 (s; C=O), 136.6 (s; C_{aryl}), 135.2 (s; C_{aryl}), 129.7 (s; C_{aryl}), 129.3 (s; C_{aryl}), 129.1 (s; C_{aryl}), 129.0 (s; C_{aryl}), 128.8 (s; C_{aryl}),128.7 (s; C_{aryl}), 102.9 (s; $COD_{ol.}$), 89.5 (s; C(*C*(Ph)$_2$)C), 59.0 (s; $COD_{ol.}$) ppm [Some of the expected signals for aryl carbon atoms are not observed due to isochrony as well as overlapping with the solvent signal.]. $^{13}C\{^1H\}$ NMR (150 MHz, C_6D_6,

298.0 K): δ = 260.1 (s; $C_{carbene}$), 168.2 (s; C=O), 136.8 (s; C_{aryl}), 129.4 (s; C_{aryl}), 129.1 (s; C_{aryl}), 129.0 (s; C_{aryl}), 121.5 (s; C_{aryl}), 102.9 (s; $COD_{ol.}$), 90.1 (s; C(*C*(Ph)$_2$)C), 58.5 (s; $COD_{ol.}$) ppm [Some of the expected signals for aryl carbon atoms are not observed due to isochrony as well as overlapping with the solvent signal.]. MS (EI, 70 eV, 110 °C) *m/z* (%): 633 (5) $[M]^{+\cdot}$, 439 (78), 401 (100), 269 (21), 165 (40). Elemental analysis calcd for $C_{29}H_{27}ClIrNO$: C 55.01, H 4.30, N 2.21, found: C 55.16, H 4.23, N 2.18.

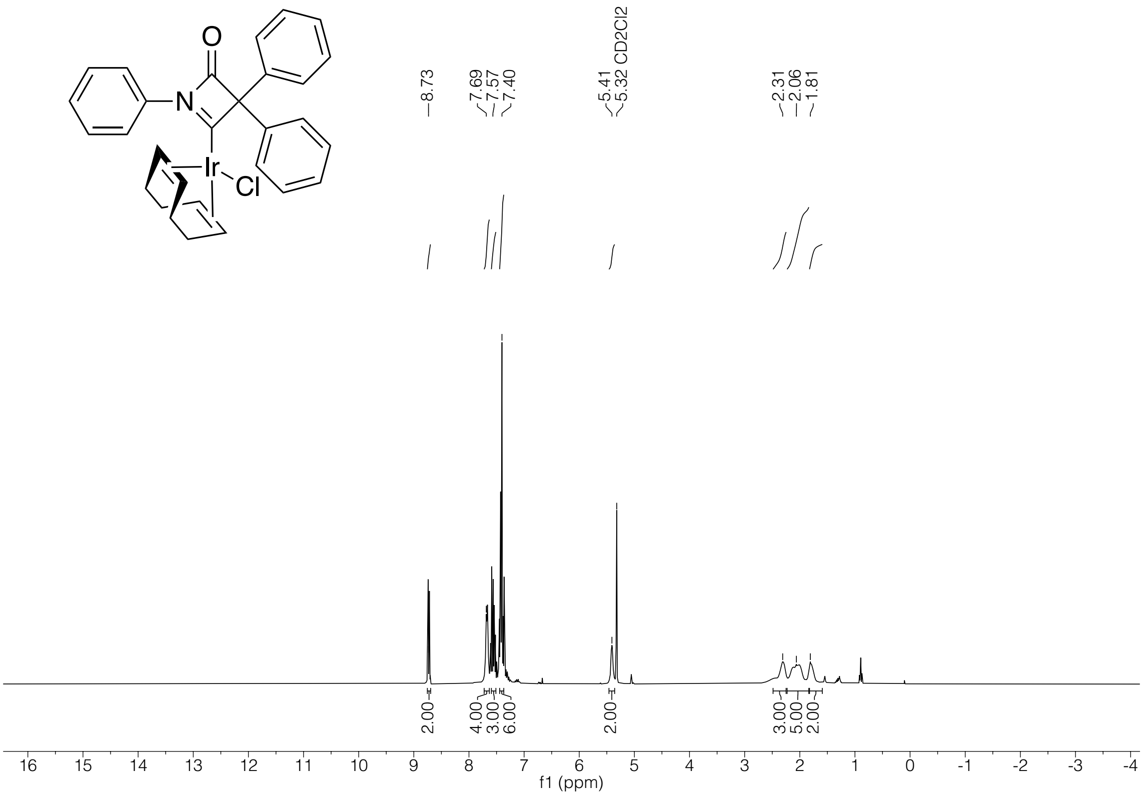

Figure SF141. ^{1}H NMR (300 MHz, C_6D_6, 298.0 K) spectrum of **Ph-6-Ph$_2$*[Ir(COD)Cl]**.

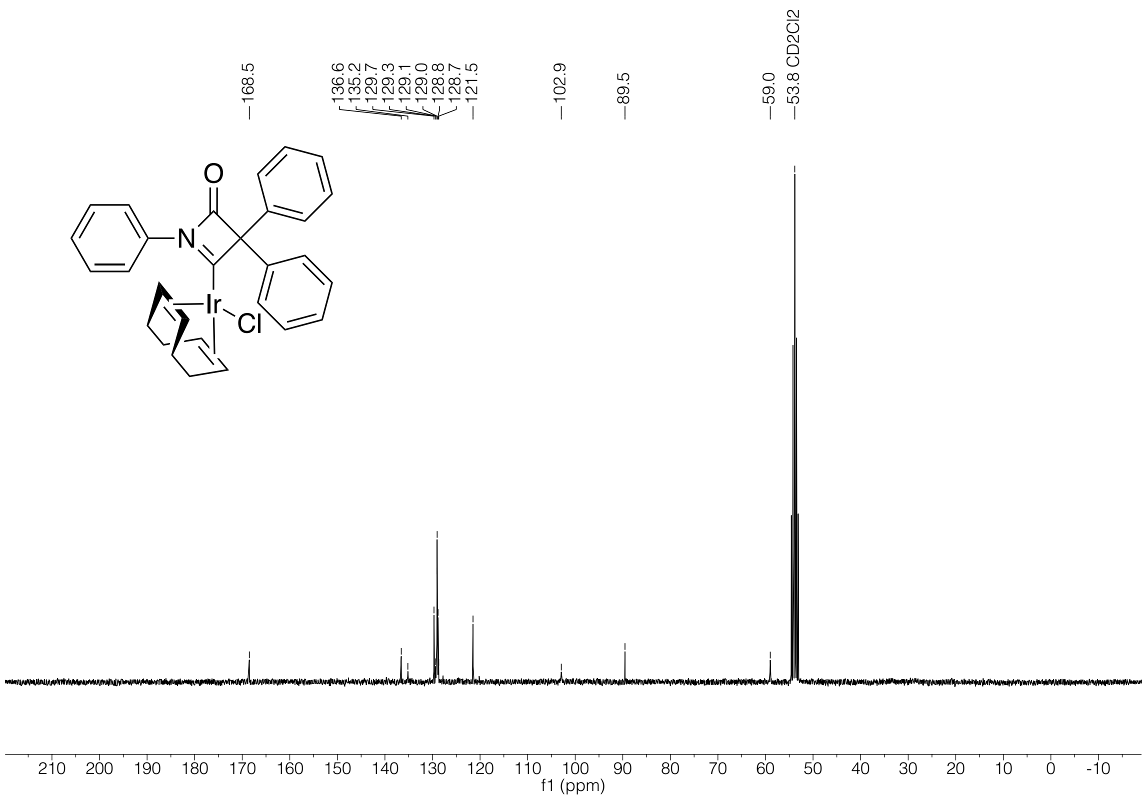

Figure SF142. $^{13}C\{^1H\}$ NMR (75 MHz, CD_2Cl_2, 298.0 K) spectrum of **Ph-6-Ph$_2$*[Ir(COD)Cl]**.

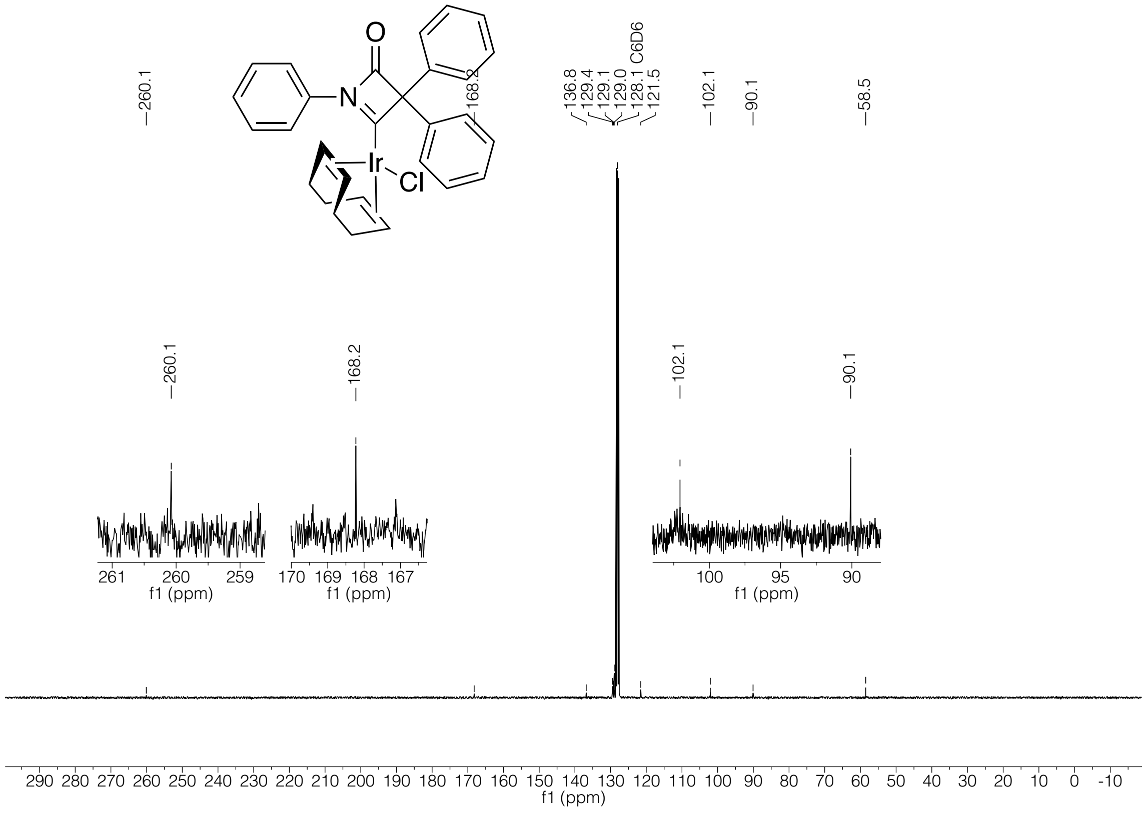

Figure SF143. $^{13}C\{^1H\}$ NMR (150 MHz, C_6D_6, 298.0 K) spectrum of **Ph-6-Ph$_2$*[Ir(COD)Cl]**.

Ph-6-Ph2*[Rh(COD)Cl]: Precursor used: **Ph-5-Ph2** (192 mg, 0.5 mmol) for method A and **Ph-6-Ph2*HCl** (167 mg, 0.5 mmol) method B. Orange, block-shaped, air-stable crystals. Yield: 8 mg, 3% (method A) / 11 mg, 4% (method B). Due to the low yield, there was not sufficient amount of sample for full NMR analysis, so that only ^{1}H NMR was recorded. The obtained yield was used for crystallization, MS and elemental analysis. ^{1}H NMR (600 MHz, $CDCl_3$, 298.0 K): δ = 8.85–8.79 (m, 2H; CH_{aryl}), 7.59–7.50 (m, 3H; CH_{aryl}), 7.45–7.28 (m, 9H; CH_{aryl}), 5.67–5.58 (m, 2H; $COD_{ol.}$), 4.23–4.17 (m, 2H; $COD_{ol.}$), 2.48–2.41 (m, 4H; $COD_{al.}$), 2.15–2.03 (m, 4H; $COD_{al.}$), 1.90–1.82 (m, 2H; $COD_{al.}$) ppm. MS (EI, 70 eV, 110 °C) *m/z* (%): 545 (6) $[M]^{+\cdot}$, 439 (27), 261 (59), 165 (21), 146 (100), 91 (46). Elemental analysis calcd for $C_{29}H_{27}ClNORh$: C 64.04, H 5.00, N 2.58, found: C 64.10, H 4.96, N 2.68.

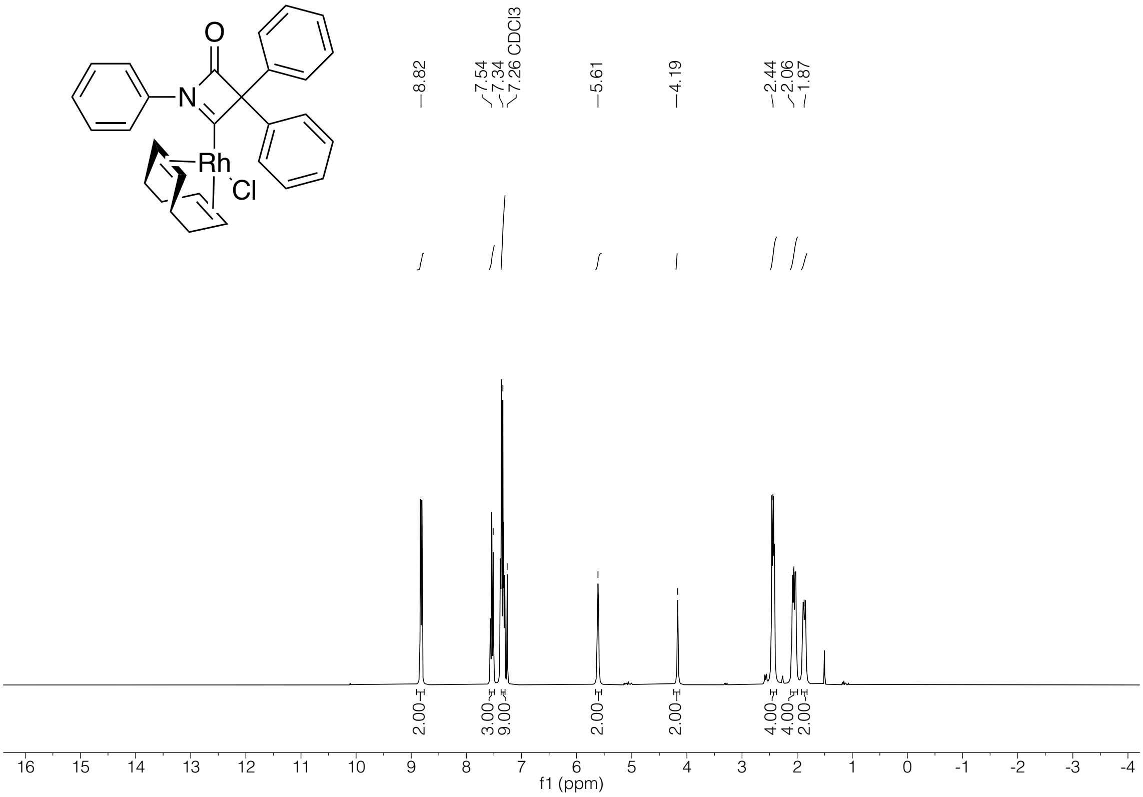

Figure SF144. ^{1}H NMR (600 MHz, $CDCl_3$, 298.0 K) spectrum of **Ph-6-Ph2*[Rh(COD)Cl]**.

Mes-6-Ph_2*[Ir(COD)Cl]: Precursor used: **Mes-5-Ph_2** (213 mg, 0.5 mmol) for method A and **Mes-6-Ph_2*HCl** (188 mg, 0.5 mmol) method B. Red, block-shaped, air-stable crystals. Yield: 324 mg, 96% (method A) / 317 mg, 94% (method B). ^{1}H NMR (600 MHz, CD_2Cl_2, 298.0 K): δ = 7.76–7.69 (m, 4H; CH_{aryl}), 7.47–6.38 (m, 6H; CH_{aryl}), 7.02 (d, J = 1 Hz, 2H; CH_{meta}), 5.31–5.29 (m, 2H; $COD_{ol.}$), 2.61–2.55 (m, 2H; $COD_{ol.}$), 2.37 (s, 6H; $CH_{3\text{-}ortho}$), 2.36 (s, 3H; $CH_{3\text{-}para}$), 2.25–2.13 (m, 2H; $COD_{al.}$), 2.04–1.73 (m, 6H; $COD_{al.}$) ppm. $^{13}C\{^{1}H\}$ NMR (150 MHz, CD_2Cl_2, 298.0 K): δ = 257.7 (s; $C_{carbene}$), 170.8 (C=O), 140.2 (s; NC_{aryl}), 135.3 (s; C-C_{aryl}), 134.7 (s; C-C_{aryl}), 131.7 (s; C_{aryl}), 129.7 (s; C_{aryl}), 129.5 (s; C_{aryl}), 129.0 (s; C_{aryl}), 128.8 (s; C_{aryl}), 105.5 (s; $COD_{ol.}$), 89.7 (s; C(*C*($Ph)_2$)C), 57.4 (s; $COD_{ol.}$), 33.7 (s; $COD_{al.}$), 28.5 (s; $COD_{al.}$), 21.3 (s; $CH_{3\text{-}para}$), 19.6 (s; $CH_{3\text{-}ortho}$) ppm. MS (EI, 70 eV, 120 °C) *m/z* (%): 675 (2) $[M]^{+\cdot}$, 481 (100), 443 (96), 311 (24), 234 (9), 165 (9), 91 (8). Elemental analysis calcd for $C_{32}H_{33}ClIrNO$: C 56.92, H 4.93, N 2.07, found: C 57.05, H 5.12, N 1.88.

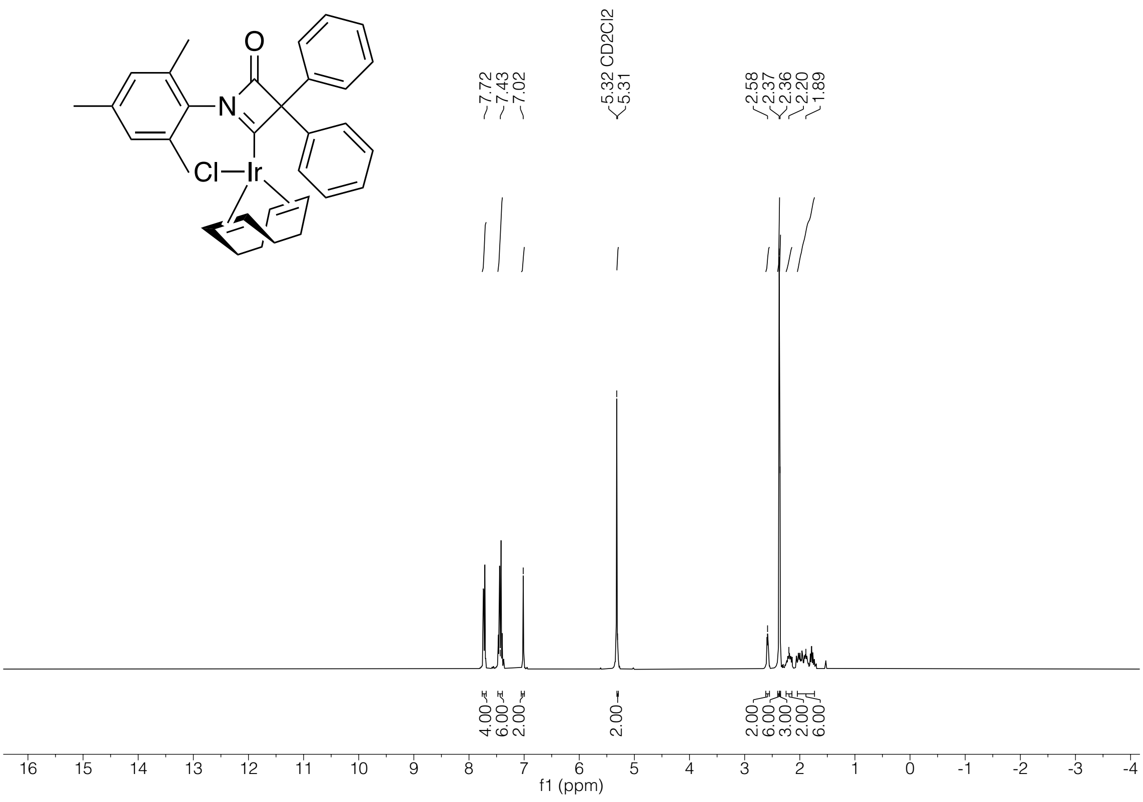

Figure SF145. ^{1}H NMR (600 MHz, CD_2Cl_2, 298.0 K) spectrum of **Mes-6-Ph_2*[Ir(COD)Cl]**.

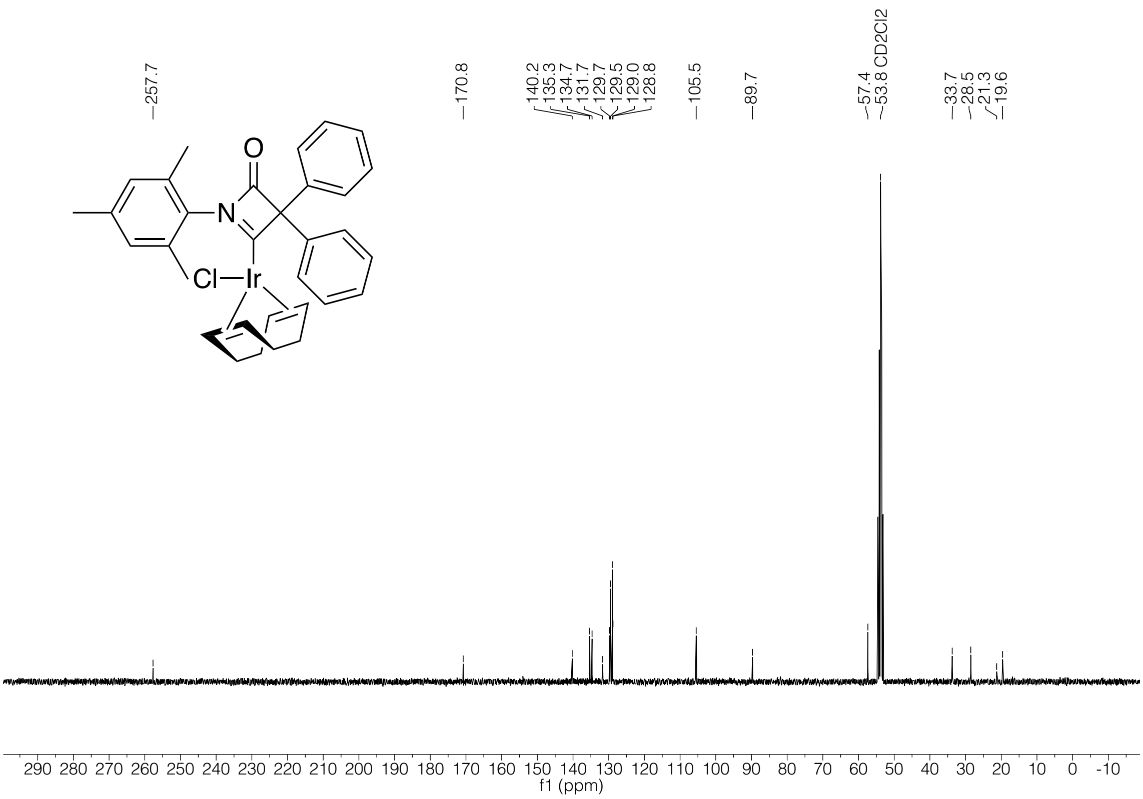

Figure SF146. $^{13}C\{^1H\}$ NMR (150 MHz, CD_2Cl_2, 298.0 K) spectrum of **Mes-6-Ph$_2$*[Ir(COD)Cl]**.

Mes-6-sCy*[Ir(COD)Cl]: Precursor used: **Mes-5-sCy** (171 mg, 0.5 mmol) for method A. Red, block-shaped, air-stable crystals. Yield: 287 mg, 97% (method A). ^1H NMR (600 MHz, C_6D_6, 298.0 K): δ = 6.63 (d, J = 1 Hz, 2H; CH_{meta}), 5.50–5.40 (m, 2H; $COD_{ol.}$), 3.10–3.00 (m, 2H; $COD_{ol.}$), 2.47–2.34 (m, 3H; CH_{aliph}), 2.31 (s, 6H; $CH_{3\text{-}ortho}$), 2.03 (s, 3H; $CH_{3\text{-}para}$), 1.97–1.82 (m, 7H; CH_{aliph}), 1.73–1.60 (m, 4H; $COD_{al.}$), 1.54–1.34 (m, 4H; $COD_{al.}$) ppm. $^{13}C\{^1H\}$ NMR (150 MHz, C_6D_6, 298.0 K): δ = 270.6 (s; $C_{carbene}$), 174.3 (s; C=O), 139.3 (s; NC_{aryl}), 134.7 (s; $C_{aryl\text{-}meta}$), 131.7 (s; $C_{aryl\text{-}para}$), 129.6 (s; $C_{aryl\text{-}ortho}$), 101.4 (s; $COD_{ol.}$), 80.5 (s; C(C_{sCy})C), 53.9 (s; $COD_{ol.}$), 33.7 (s; $COD_{al.}$), 30.2 (s; C_{aliph}), 28.6 (s; $COD_{al.}$), 25.5 (s; C_{aliph}), 23.9 (s; C_{aliph}), 19.2 (s; $CH_{3\text{-}para}$), 19.1 (s; $CH_{3\text{-}ortho}$) ppm. MS (EI, 70 eV, 110 °C) *m/z* (%): 591 (36) $[M]^{+\cdot}$, 481 (46), 443 (100), 227 (72), 212 (17), 146 (10), 91 (6). Elemental analysis calcd for $C_{25}H_{33}ClIrNO$: C 50.79, H 5.63, N 2.37, found: C 51.02, H 5.54, N 2.56.

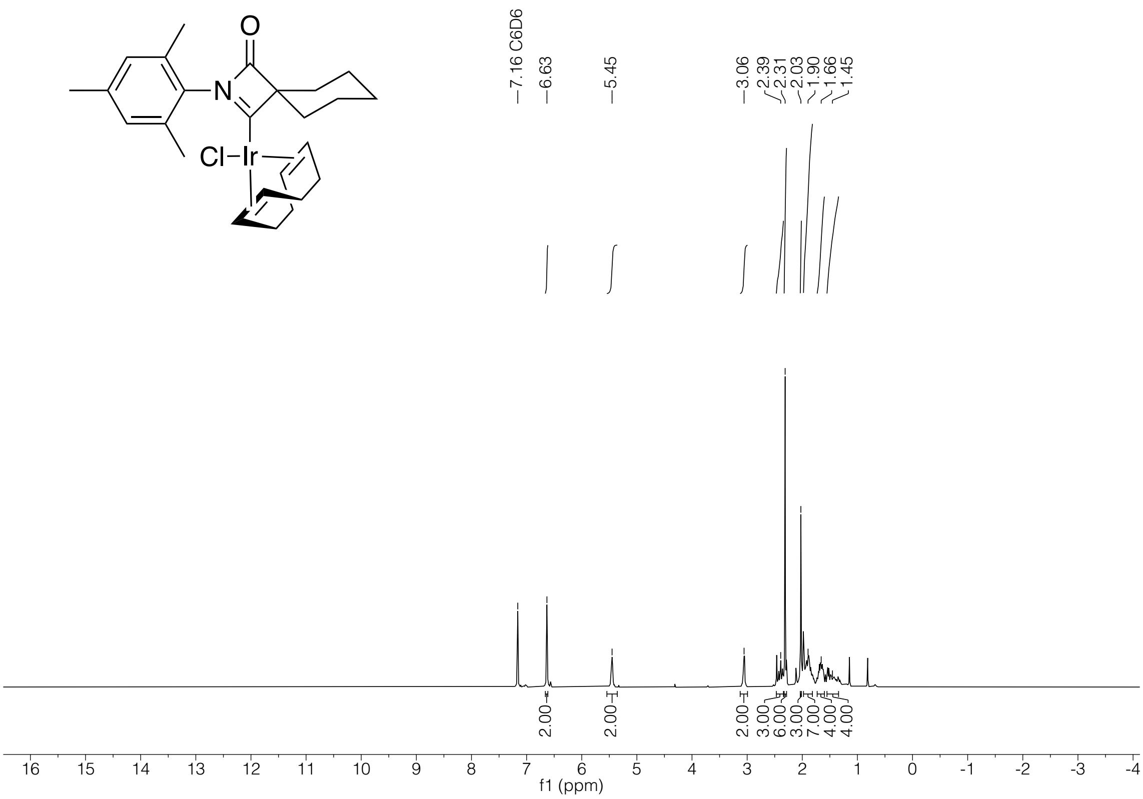

Figure SF157. ^{1}H NMR (600 MHz, C_6D_6, 298.0 K) spectrum of **Mes-6-sCy*[Ir(COD)Cl]**.

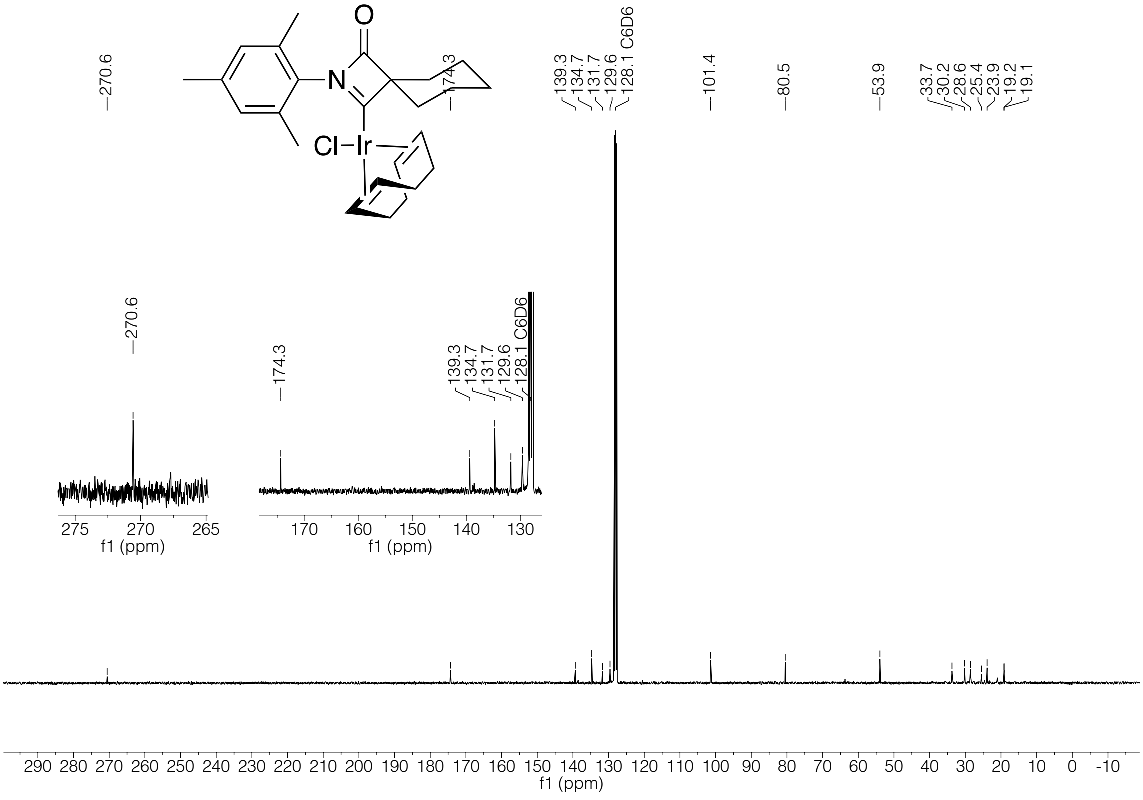

Figure SF158. $^{13}C\{^{1}H\}$ NMR (150 MHz, C_6D_6, 298.0 K) spectrum of **Mes-6-sCy*[Ir(COD)Cl]**.

Dipp-6-Me$_2$*[Rh(COD)Cl]: Precursor used: **Dipp-5-Me$_2$** (172 mg, 0.5 mmol) for method A and **Dipp-6-Me$_2$*HCl** (147 mg, 0.5 mmol) method B. Orange, block-shaped, air-stable crystals. Yield: 247 mg, 98% (method A) / 244 mg, 97% (method B). ^{1}H NMR (600 MHz, C_6D_6, 298.0 K): δ = 7.23–7.19 (m, 1H; $CH_{aryl\text{-}para}$), 7.11–7.06 (m, 2H; $CH_{aryl\text{-}meta}$), 5.75–5.67 (m, 2H; $COD_{ol.}$), 3.40–3.33 (m, 4H; CH_{iPr} and $COD_{ol.}$), 1.97–1.85 (m, 4H; $COD_{al.}$), 1.70–1.62 (m, 4H; $COD_{al.}$), 1.60 (s, 6H; $C(C(CH_3)_2)C$), 1.33 (d, J = 7 Hz, 6H; $CH_{3\text{-}iPr}$), 1.14 (d, J = 7 Hz, 6H; $CH_{3\text{-}iPr}$) ppm. $^{13}C\{^1H\}$ NMR (150 MHz, C_6D_6, 298.0 K): δ = 296.1 (d, $^1J_{C\text{-}Rh}$ = 50 Hz; $C_{carbene}$), 173.1 (d, $^3J_{C\text{-}Rh}$ = 2 Hz; C=O), 145.8 (s; $C_{aryl\text{-}ortho}$), 130.8 (s; NC$_{aryl}$), 124.4 (s; $C_{aryl\text{-}meta}$), 113.1 (d, $^1J_{C\text{-}Rh}$ = 4 Hz; $COD_{ol.}$), 74.3 (d, $^2J_{C\text{-}Rh}$ = 2 Hz; C(C(CH$_3$)$_2$)C), 69.2 (d, $^2J_{C\text{-}Rh}$ = 13 Hz; $COD_{ol.}$), 33.0 (s; CH_{iPr}), 29.2 (s; $COD_{al.}$), 28.0 (s; $CH_{3\text{-}iPr}$), 25.1 (s; $CH_{3\text{-}iPr}$), 23.2 (s; C(C(CH$_3$)$_2$)C), 20.4 (s; $COD_{al.}$) ppm [Some of the expected signals for aryl carbon atoms are not observed due to isochrony as well as overlapping with the solvent signal.]. MS (EI, 70 eV, 120 °C) *m/z* (%): 503 (12) $[M]^{+\cdot}$, 395 (64), 285 (15), 229 (29), 173 (100). Elemental analysis calcd for $C_{25}H_{35}ClNORh$: C 59.59, H 7.00, N 2.78, found: C 59.50, H 6.89, N 2.84.

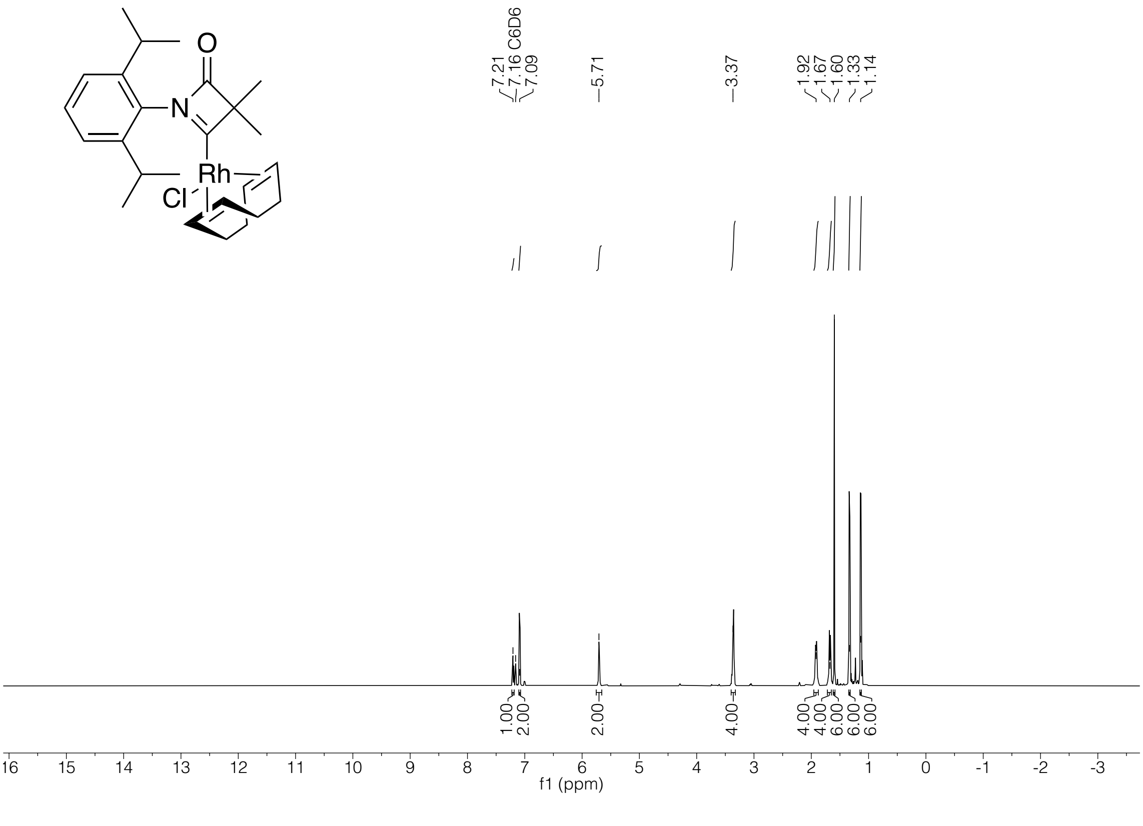

Figure SF159. ^{1}H NMR (600 MHz, C_6D_6, 298.0 K) spectrum of **Dipp-6-Me$_2$*[Rh(COD)Cl]**.

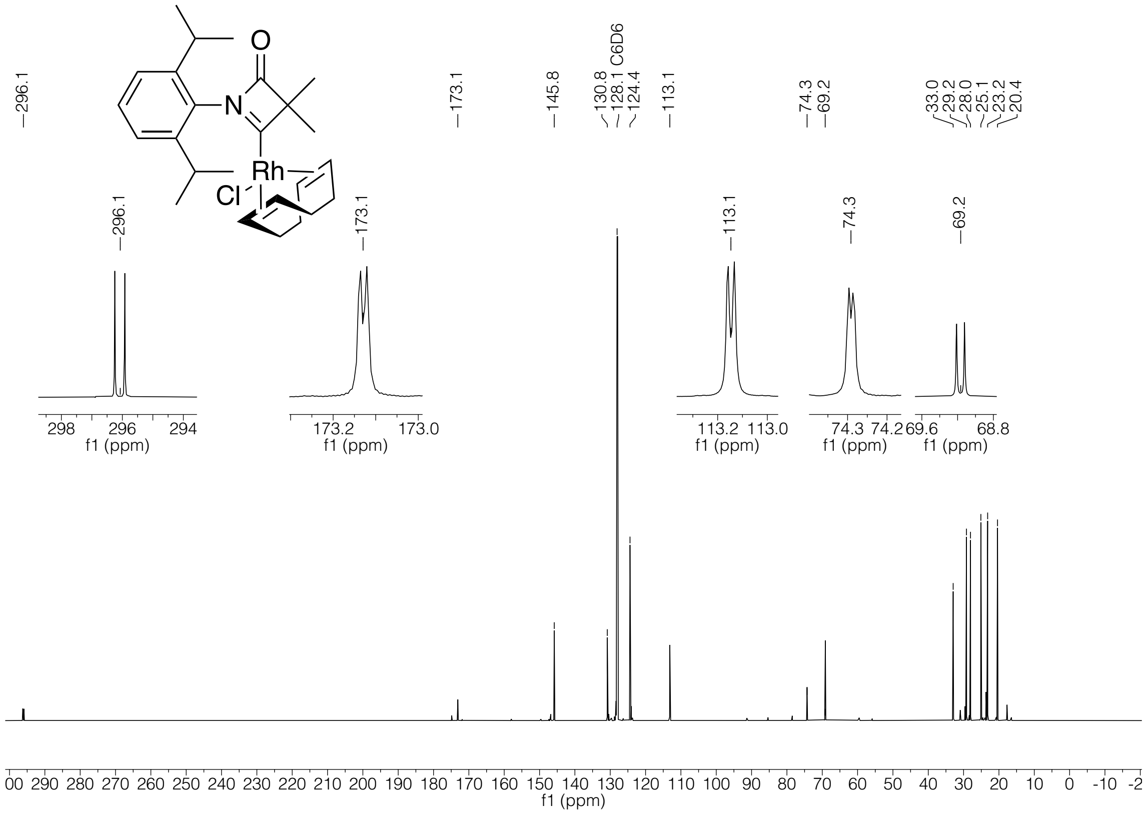

Figure SF160. $^{13}C\{^1H\}$ NMR (150 MHz, C_6D_6, 298.0 K) spectrum of **Dipp-6-Me$_2$*[Rh(COD)Cl]**.

Dipp-6-Ph$_2$*[Rh(COD)Cl]: Precursor used: **Dipp-5-Ph$_2$** (234 mg, 0.5 mmol) for method A and **Dipp-6-Ph$_2$*HCl** (209 mg, 0.5 mmol) method B. Orange, block-shaped, air-stable crystals. Yield: 12 mg, 4% (method A) / 9 mg, 3% (method B). Due to the low yield, there was not sufficient amount of sample for full NMR analysis, so that only ^1H NMR was recorded. The obtained yield was used for crystallization, MS and elemental analysis. ^1H NMR (300 MHz, C_6D_6, 298.0 K): δ = 7.90–7.80 (m, 4H; CH_{aryl}), 7.25–7.17 (m, 5H; CH_{aryl}), 7.13–7.08 (m, 3H; CH_{aryl}), 6.94–6.90 (m, 3H; CH_{aryl}), 5.82–5.75 (m, 2H; $COD_{ol.}$), 3.55 (sept, J = 7 Hz, 2H; CH_{iPr}), 3.08–3.05 (m, 2H; $COD_{ol.}$), 1.88–1.82 (m, 4H; $COD_{al.}$), 1.60–1.53 (m, 4H; $COD_{al.}$), 1.45–1.40 (m, 2H; $COD_{al.}$), 1.37 (d, J = 7 Hz, 6H; $CH_{3\text{-}iPr}$), 1.22 (d, J = 7 Hz, 6H; $CH_{3\text{-}iPr}$) ppm. MS (EI, 70 eV, 140 °C) *m/z* (%): 627 (8) $[M]^{+\cdot}$, 395 (20), 173 (100), 91 (49). Elemental analysis calcd for $C_{35}H_{39}ClNORh$: C 66.93, H 6.26, N 2.23, found: C 67.12, H 6.34, N 1.98.

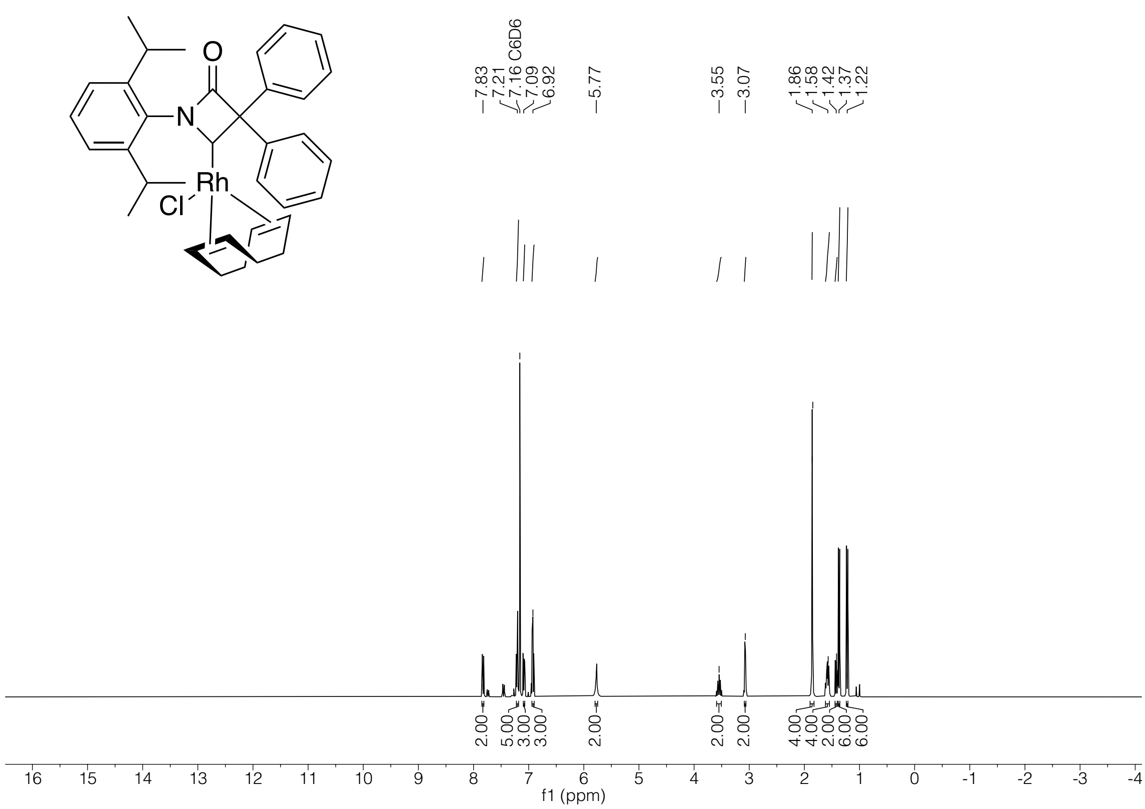

Figure SF161. ^{1}H NMR (300 MHz, C_6D_6, 298.0 K) spectrum of **Dipp-6-Ph$_2$*[Rh(COD)Cl]**.

Dipp-6-sCy*[Rh(COD)Cl]: Precursor used: **Dipp-5-sCy** (192 mg, 0.5 mmol) for method A. Orange, block-shaped, air-stable crystals. Yield: 264 mg, 97% (method A). ^{1}H NMR (300 MHz, C_6D_6, 298.0 K): δ = 7.23–7.18 (m, 1H; $CH_{aryl\text{-}para}$), 7.12–7.08 (m, 2H; $CH_{aryl\text{-}meta}$), 5.79–5.70 (m, 2H; $COD_{ol.}$), 3.47 (sept, J = 7 Hz, 2H; CH_{iPr}), 3.41–3.35 (m, 2H; $COD_{ol.}$), 2.57–2.45 (m, 2H; C_{aliph}), 2.10–1.83 (m, 9H; C_{aliph}), 1.72–1.60 (m, 7H; C_{aliph}), 1.37 (d, J = 7 Hz, 6H; $CH_{3\text{-}iPr}$), 1.17 (d, J = 7 Hz, 6H; $CH_{3\text{-}iPr}$) ppm [The aliphatic carbon atoms cannot be differentiated from each other in ^{1}H NMR due to overlapping signals (multiplets).]. $^{13}C\{^{1}H\}$ NMR (75 MHz, C_6D_6, 298.0 K): δ = 295.6 (s; $C_{carbene}$), 173.1 (s; C=O), 146.0 (s; $C_{aryl\text{-}ortho}$), 130.8 (s; NC_{aryl}), 124.4 (s; $C_{aryl\text{-}meta}$), 112.4 (d, J = 4 Hz; $COD_{ol.}$), 79.5 (s; C(C_{sCy})C), 68.9 (s; $COD_{ol.}$), 32.9 (s; $COD_{al.}$), 30.8 (s; CH_{iPr}), 29.2 (s; C_{aliph}), 28.1 (s; $COD_{al.}$), 25.2 (s; C_{aliph}), 24.0 ($C_{aliph.}$), 23.2 (s; $CH_{3\text{-}iPr}$) ppm [No coupling between rhodium and carbon atoms was observed (Due to low concentration. Some of the expected signals for aryl carbon atoms are not observed due to isochrony as well as overlapping with the solvent signal.]. MS (EI, 70 eV, 130 °C) *m/z* (%): 543 (10) $[M]^{+\cdot}$, 395 (74), 269 (51), 173 (100). Elemental analysis calcd for $C_{28}H_{39}ClNORh$: C 61.82, H 7.23, N 2.57, found: C 61.94, H 7.41, N 2.66.

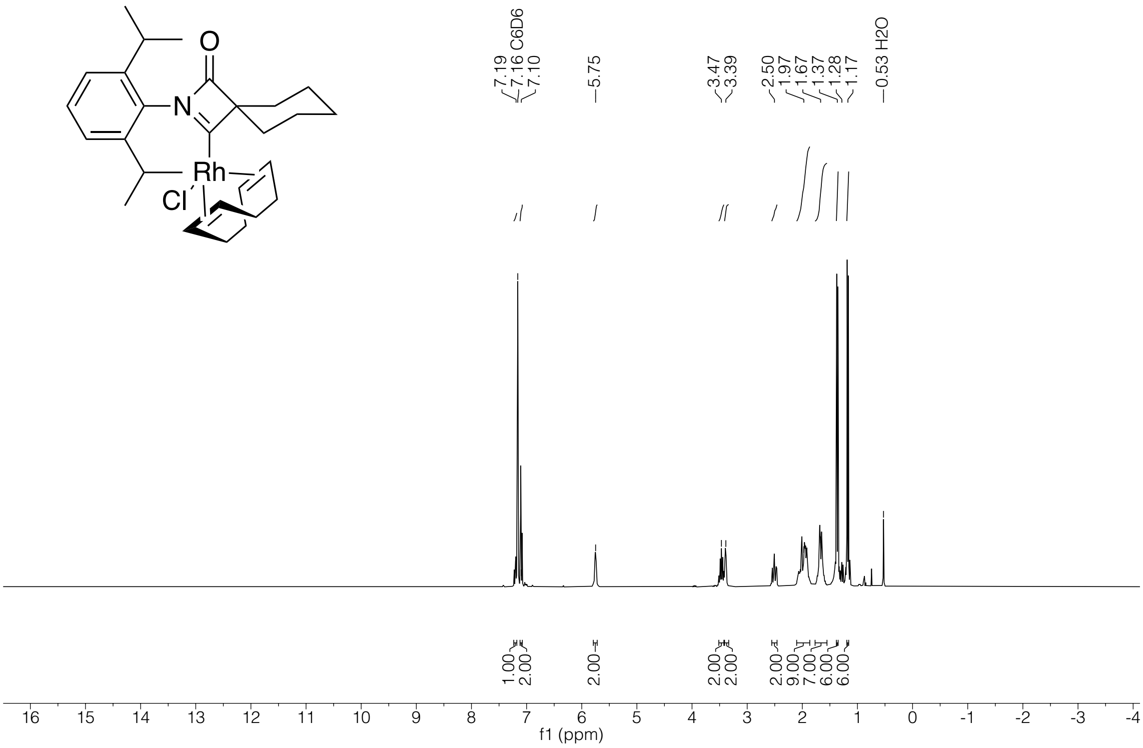

Figure SF162. ^{1}H NMR (300 MHz, C_6D_6, 298.0 K) spectrum of **Dipp-6-sCy*[Rh(COD)Cl]**.

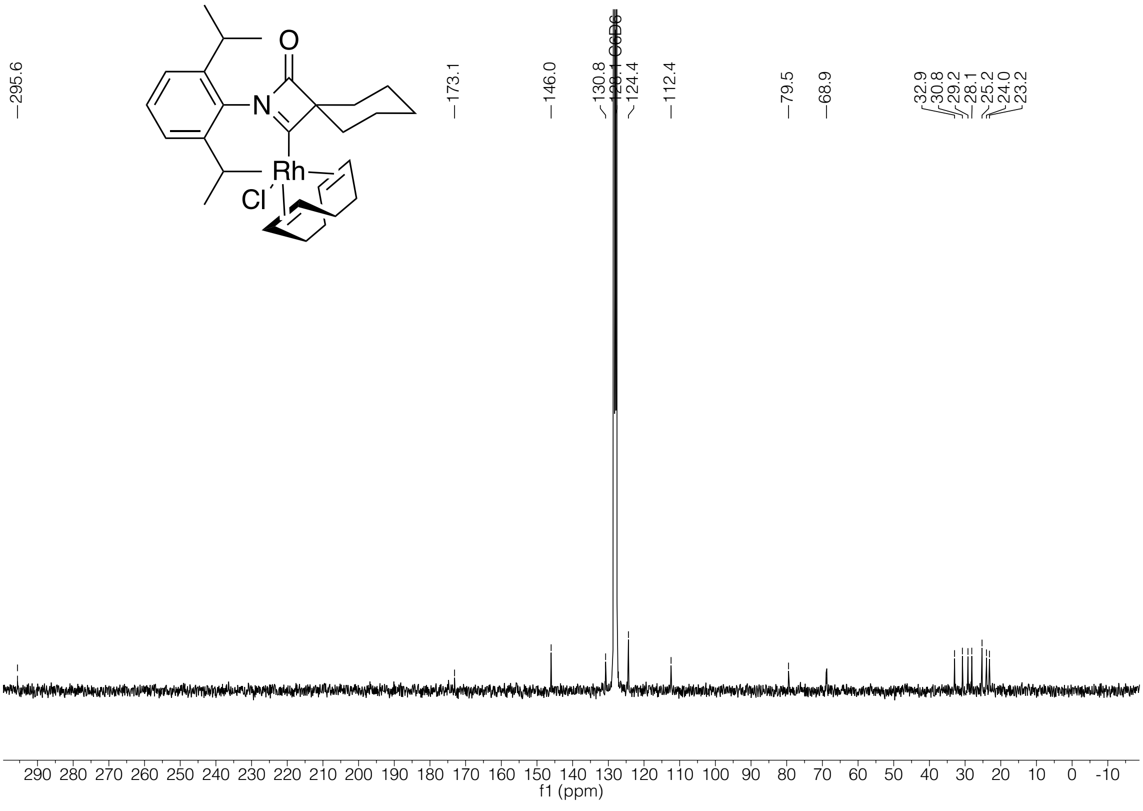

Figure SF163. $^{13}C\{^{1}H\}$ NMR (75 MHz, C_6D_6, 298.0 K) spectrum of **Dipp-6-sCy*[Rh(COD)Cl]**.

k. Synthesis of iridium/rhodium CO-complexes 6*[Ir/Rh(CO)$_2$Cl]

The corresponding 1,5-cyclooctadiene complex **6*[Ir/Rh(COD)Cl]** (0.2 mmol, 1 eq.) was dissolved in 5 ml of dichloromethane and CO was bubbled through the solution, until the color changed to yellow. After removal of all volatiles in vacuo, a yellow powder was obtained that turns black in several minutes indicating decomposition of the product, which was confirmed by NMR analysis. Decomposition products could not be identified. The desired CO complexes seem to be stable only stable under CO atmosphere in dichloromethane. By this, no analysis except IR spectroscopy was possible.

Ph-6-Ph$_2$*[Ir(CO)$_2$Cl]: COD-complex used: **Ph-6-Ph$_2$*[Ir(COD)Cl]** (127 mg, 0.2 mmol). Yellow, air sensitive powder. The product is stable under CO atmosphere in dichloromethane. IR (CH_2Cl_2, at, cm^{-1}): $\tilde{\nu}$ = 2009.9 (s; C=O), 2085.1 (s; C=O). TEP (CH_2Cl_2, at, cm^{-1}): 2071.

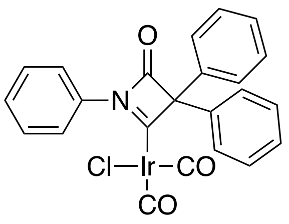

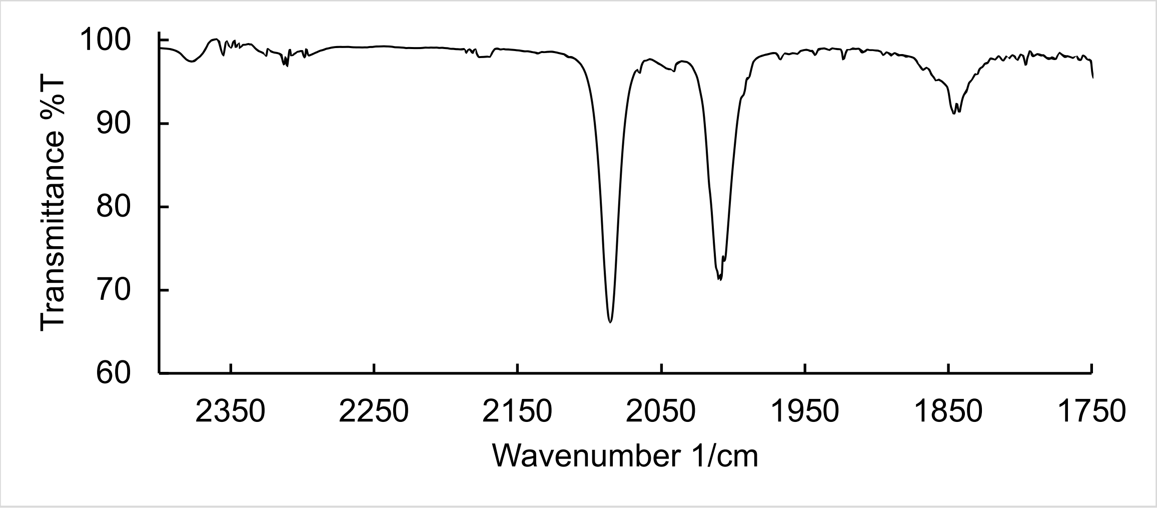

Figure SF164. IR spectrum (NaCl liquid cell, DCM, at) of **Ph-6-Ph$_2$*[Ir(CO)$_2$Cl]**.

Ph-6-Ph$_2$*[Rh(CO)$_2$Cl]: COD-complex used: **Ph-6-Ph$_2$*[Rh(COD)Cl]** (10 mg, 0.02 mmol). Yellow, air sensitive powder. The product is stable under CO atmosphere in dichloromethane. IR (CH_2Cl_2, at, cm^{-1}): $\tilde{\nu}$ = 2032.1 (s; C=O), 2093.3 (s; C=O). TEP (CH_2Cl_2, at, cm^{-1}): 2070.

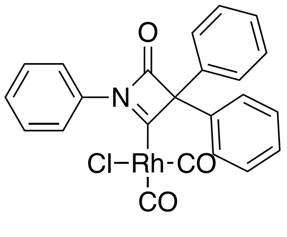

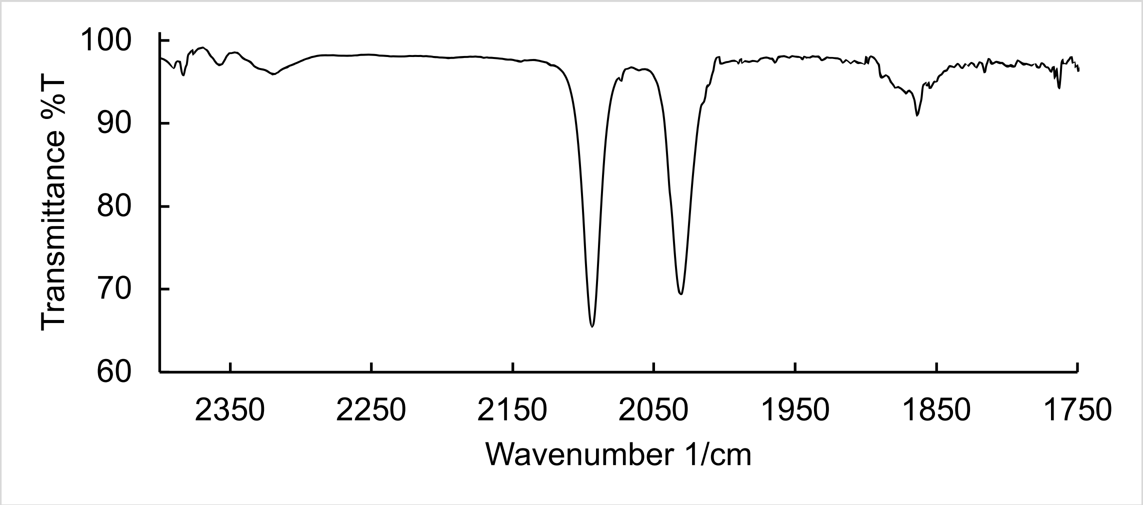

Figure SF165. IR spectrum (NaCl liquid cell, DCM, at) of **Ph-6-Ph$_2$*[Rh(CO)$_2$Cl]**.

Mes-6-Ph$_2$*[Ir(CO)$_2$Cl]: COD-complex used: **Mes-6-Ph$_2$*[Ir(COD)Cl]** (135 mg, 0.2 mmol). Yellow, air sensitive powder. The product is stable under CO atmosphere in dichloromethane. IR (CH_2Cl_2, at, cm^{-1}): $\tilde{\nu}$ = 2007.0 (s; C=O), 2086.1 (s; C=O). TEP (CH_2Cl_2, at, cm^{-1}): 2071.

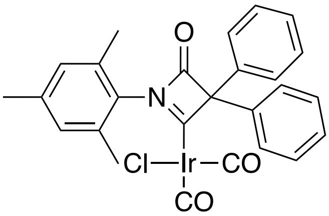

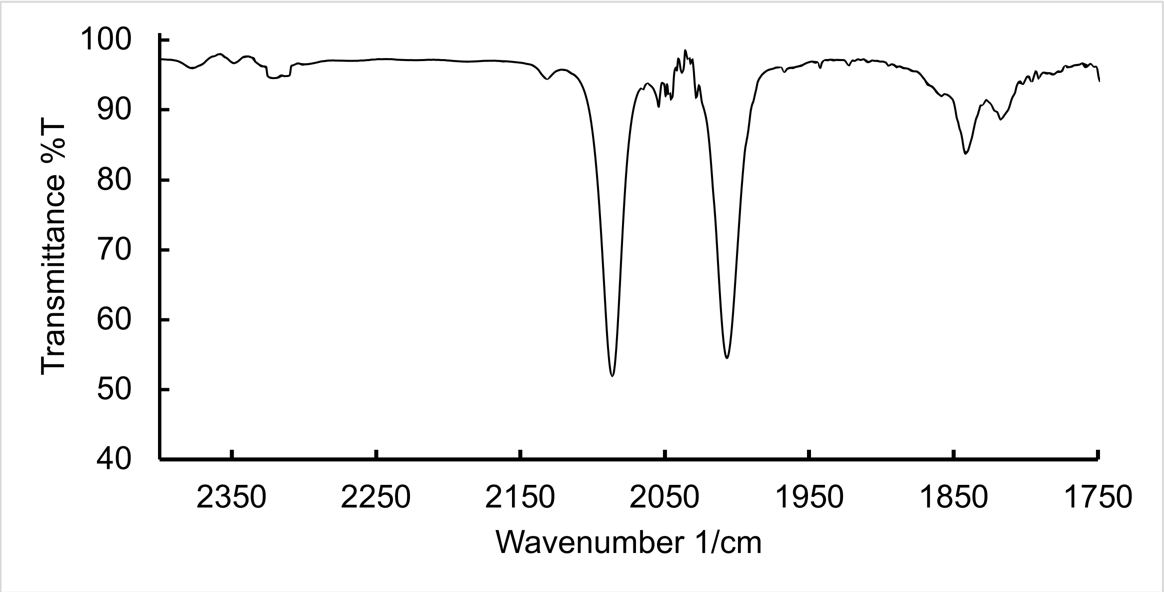

Figure SF166. IR spectrum (NaCl liquid cell, DCM, a.t.) of **Mes-6-Ph$_2$*[Ir(CO)$_2$Cl]**.

Mes-6-sCy*[Ir(CO)$_2$Cl]: COD-complex used: **Mes-6-sCy*[Ir(COD)Cl]** (118 mg, 0.2 mmol). Yellow, air sensitive powder. The product is stable under CO atmosphere in dichloromethane. IR (CH_2Cl_2, at, cm^{-1}): $\tilde{\nu}$ = 2000.3 (s; C=O), 2082.3 (s; C=O). TEP (CH_2Cl_2, at, cm^{-1}): 2066.

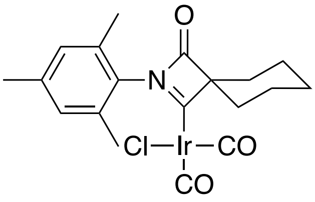

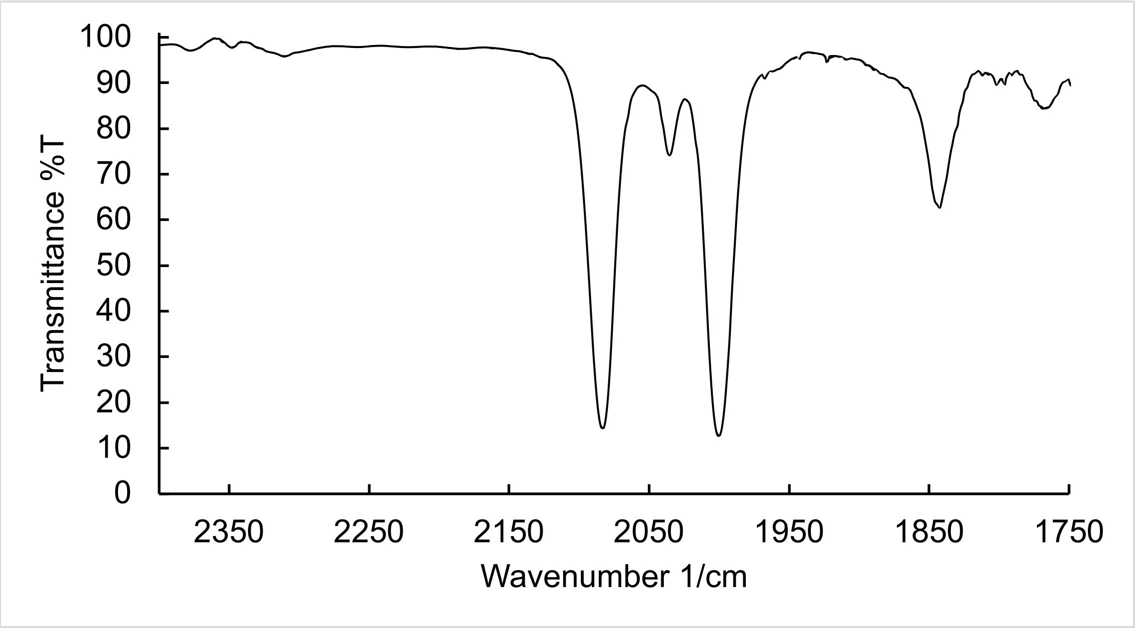

Figure SF167. IR spectrum (NaCl liquid cell, DCM, at) of **Mes-6-sCy*[Ir(CO)$_2$Cl]**.

Dipp-6-Me$_2$*[Rh(CO)$_2$Cl]: COD-complex used: **Dipp-6-Me$_2$*[Rh(COD)Cl]** (101 mg, 0.2 mmol). Yellow, air sensitive powder. The product is stable under CO atmosphere in dichloromethane. IR (CH_2Cl_2, at, cm^{-1}): $\tilde{\nu}$ = 2026.3 (s; C=O), 2093.8 (s; C=O). TEP (CH_2Cl_2, at, cm^{-1}): 2068.

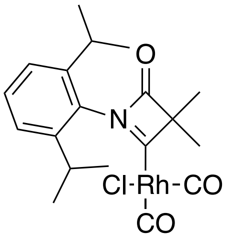

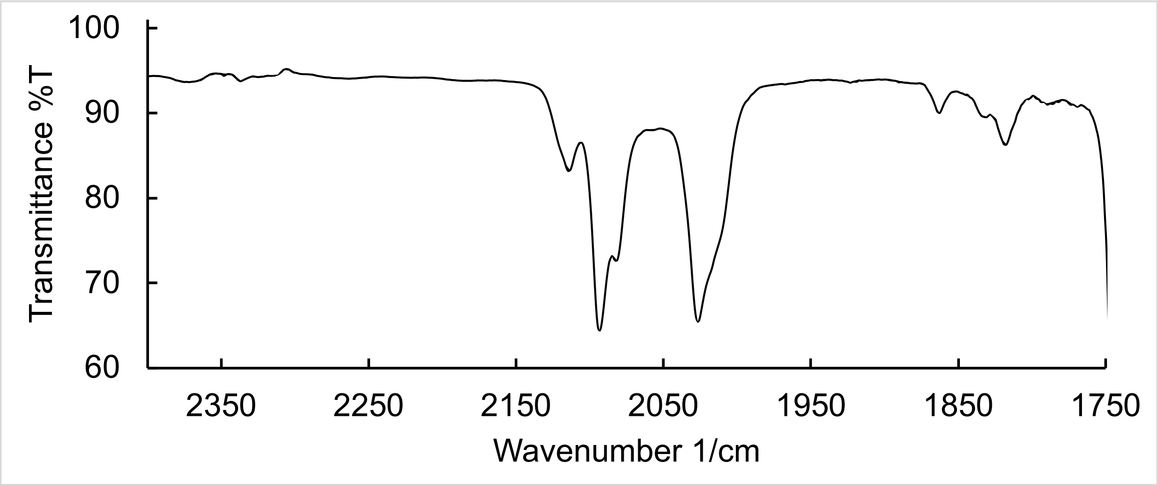

Figure SF168. IR spectrum (NaCl liquid cell, DCM, at) of **Dipp-6-Me$_2$*[Rh(CO)$_2$Cl]**.

Dipp-6-Ph$_2$*[Rh(CO)$_2$Cl]: COD-complex used: **Dipp-6-Ph$_2$*[Rh(COD)Cl]** (10 mg, 0.016 mmol). Yellow, air sensitive powder. The product is stable under CO atmosphere in dichloromethane. IR (CH_2Cl_2, at, cm^{-1}): $\tilde{v}$ = 2034.7 (s; C=O), 2087.3 (s; C=O). TEP (CH_2Cl_2, at, cm^{-1}): 2069.

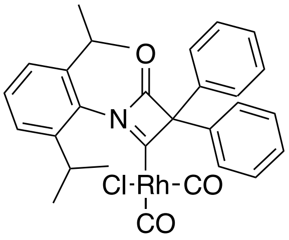

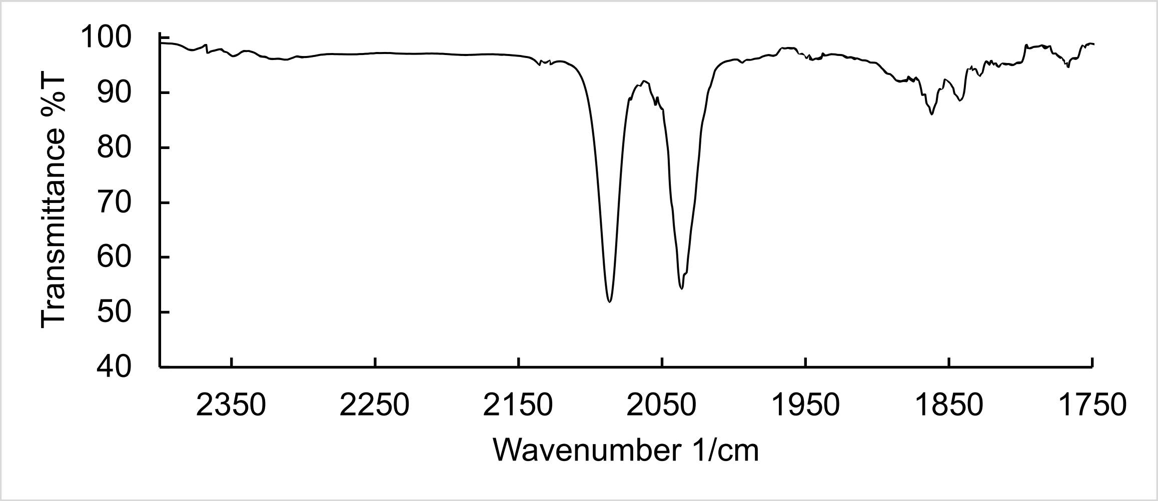

Figure SF169. IR spectrum (NaCl liquid cell, DCM, at) of **Dipp-6-Ph$_2$*[Rh(CO)$_2$Cl]**.

Dipp-6-sCy*[Rh(CO)$_2$Cl]: COD-complex used: **Dipp-6-sCy*[Rh(COD)Cl]** (109 mg, 0.2 mmol). Yellow, air sensitive powder. The product is stable under CO atmosphere in dichloromethane. Yield: 97 mg, 99%. IR (CH_2Cl_2, at, cm^{-1}): $\tilde{v}$ = 2015.7 (s; C=O), 2096.7 (s; C=O). TEP (CH_2Cl_2, at, cm^{-1}): 2065.

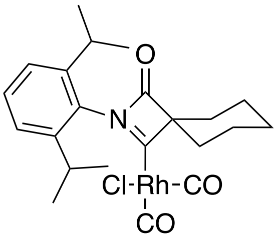

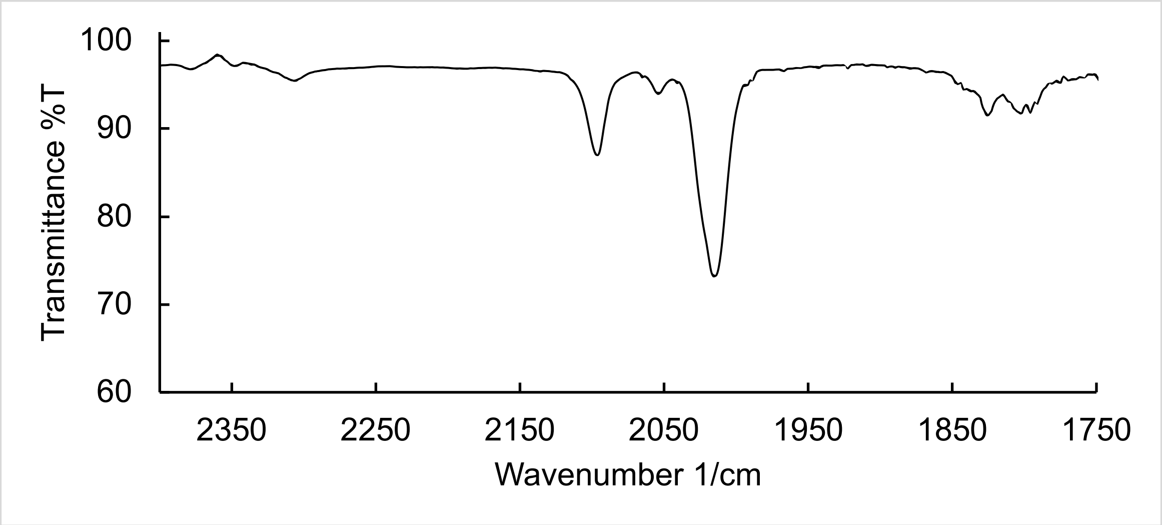

Figure SF170. IR spectrum (NaCl liquid cell, DCM, at) of **Dipp-6-sCy*[Rh(CO)$_2$Cl]**.

I. Synthesis of epoxides 11

Method A: The corresponding 1,3,4-oxadiazole-based precursor **5** (0.5 mmol, 1 eq.) and acetone (0.74 ml, 10 mmol, 20 eq.) were dissolved in 2 ml toluene at ambient temperature. After heating in a sealed glass ampoule at 110 °C for 16 h, the yellow solution was purified by column chromatography. After removal of all volatiles, a colorless, air stable powder was obtained. The by-products are the known products described in the section on thermolysis of precursors **5** without carbene scavengers.

Method B: Alternatively, the epoxides **11** can be synthesized by reaction of acetone with the carbenes generated in situ using the precursors **6*HCl** and NaHMDS as a strong base. The precursors of type **6*HCl** (0.5 mmol, 1 eq.) and acetone (0.74 ml, 10 mmol, 20 eq.) were suspended in 10 ml of tetrahydrofuran followed by the addition 0.6 ml of NaHMDS in THF (1 M, 0.6 mmol, 1.2 eq.) under stirring at -80 °C. After 2 h the cold bath was removed and stirring was continued at ambient temperature for 14 h. All volatiles were removed in vacuo and the resulting brown powder was suspended in 2 ml of dichloromethane, filtered over silica and purified by column chromatography (diethyl ether/*n*-hexane 1:5, silica). After removal of all volatiles, a colorless, air-stable powder was obtained. The main product is the corresponding dimer.

Epoxides **11** cannot be thermolyzed to their corresponding carbenes and acetone even when heated to 200 °C, indicating that the epoxides are not the result of an intermolecular reaction of the ylidic intermediate. Furthermore, the epoxides do not undergo exchange reactions with acetone-d_6.

Ph-11-Cl$_2$: Precursor used: **Ph-5-Cl$_2$** (150 mg, 0.5 mmol) for method A and **Ph-6-Cl$_2$*HCl** (124 mg, 0.5 mmol) method B. Colorless, air-stable powder. Yield: 18 mg, 13% (method A) / 28 mg, 21% (method B). ^{1}H NMR (300 MHz, $CDCl_3$, 298.0 K): δ = 7.71–7.67 (m, 2H; CH_{aryl}), 7.32–7.28 (m, 2H; CH_{aryl}), 7.11–7.09 (m, 1H; CH_{aryl}), 1.00 (appears as s, 6H; CH_3) ppm. $^{13}C\{^1H\}$ NMR (75 MHz, $CDCl_3$, 298.0 K): δ = 161.5 (s; C=O), 136.1 (s; C_{aryl}), 129.5 (s; C_{aryl}), 126.1 (s; C_{aryl}), 120.7 (s; C_{aryl}), 83.7 (s; C_{quart}), 70.6 (s; C_{quart}), 68.5 (s; C_{quart}), 33.3 (s; CH_3) ppm. MS (ESI) *m/z* (%): 272.03 [M–H]$^+$. Elemental analysis calcd for $C_{12}H_{11}Cl_2NO_2$: C 52.97, H 4.07, N 5.15, found: C 53.24, H 4.41, N 4.67.

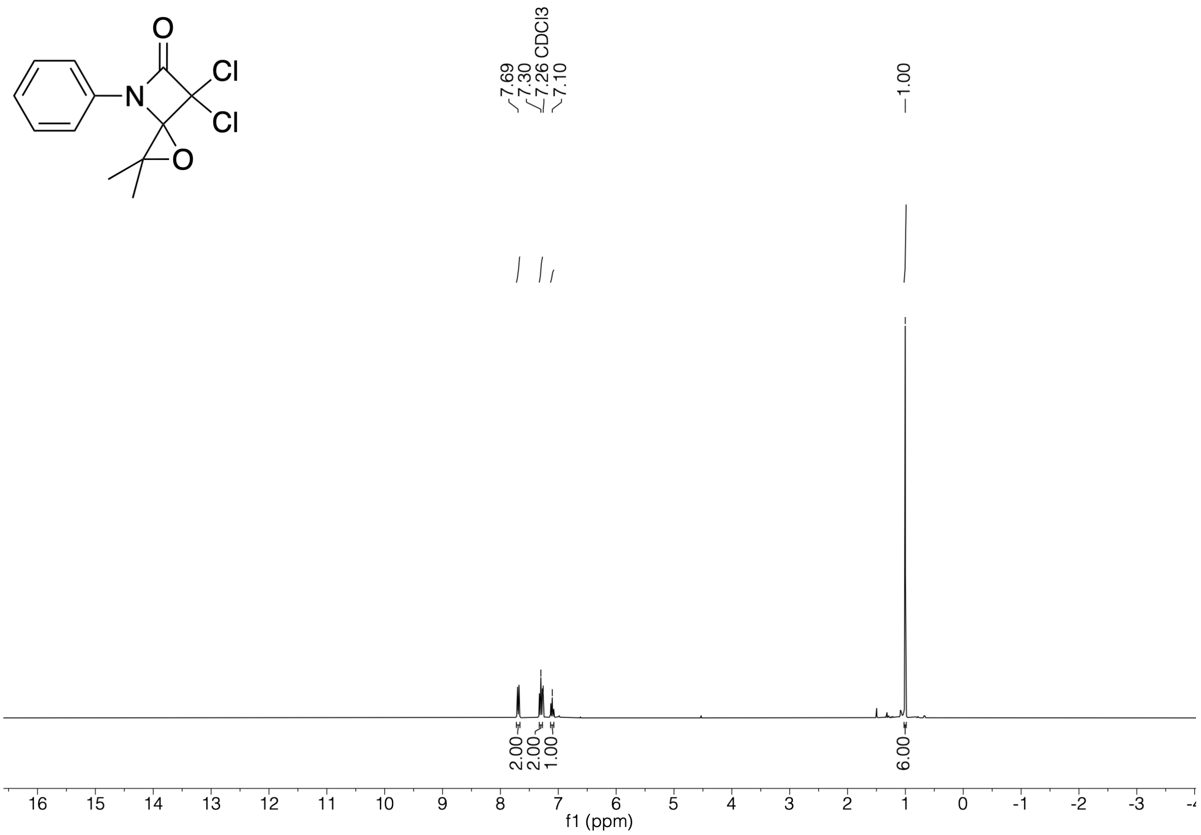

Figure SF171. ^{1}H NMR (300 MHz, $CDCl_3$, 298.0 K) spectrum of **Ph-11-Cl$_2$**.

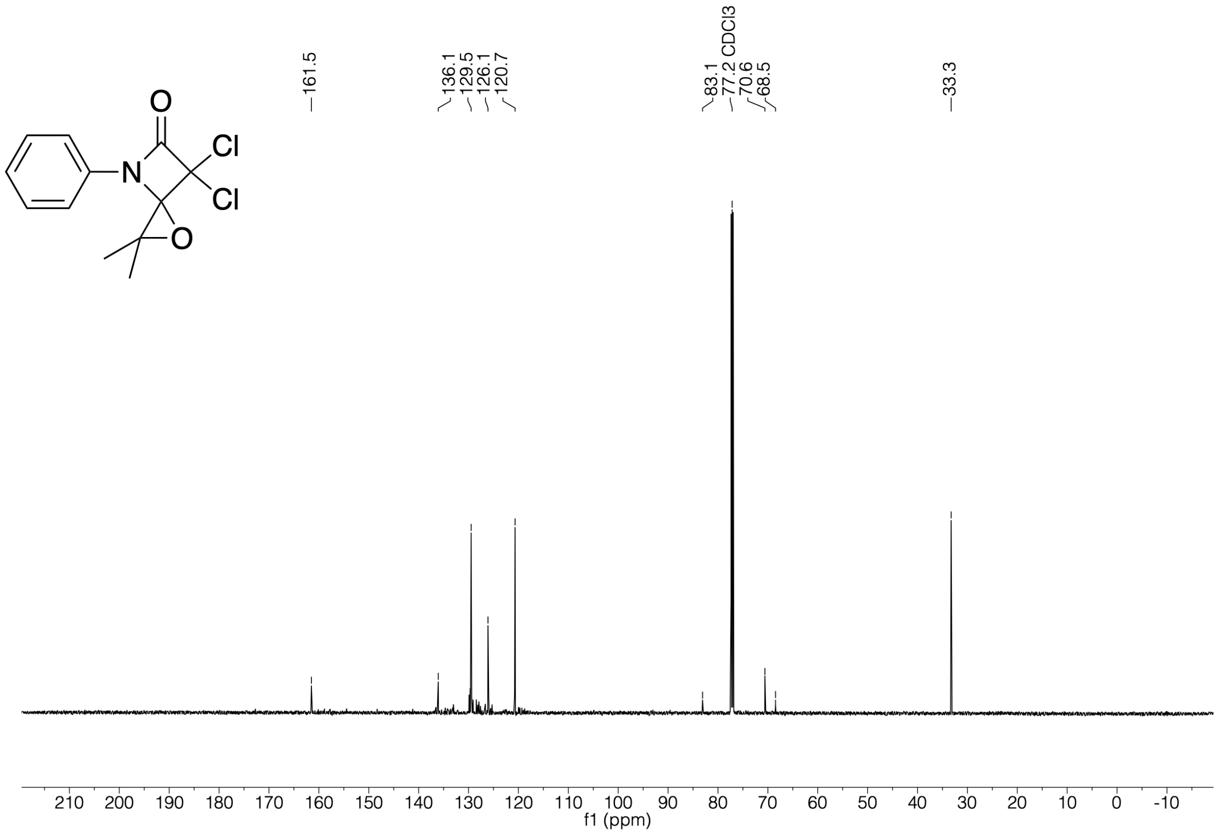

Figure SF172. $^{13}C\{^1H\}$ NMR (75 MHz, $CDCl_3$, 298.0 K) spectrum of **Ph-11-Cl₂**.

Ph-11-Ph$_2$: Precursor used: **Ph-5-Ph$_2$** (192 mg, 0.5 mmol) for method A and **Ph-6-Ph$_2$*HCl** (167 mg, 0.5 mmol) method B. Colorless, air-stable powder. Yield: 16 mg, 9% (method A) / 27 mg, 15% (method B). ^{1}H NMR (300 MHz, $CDCl_3$, 298.0 K): δ = 7.89–7.87 (m, 1H; CH_{aryl}), 7.69–7.67 (m, 1H; CH_{aryl}), 7.56–7.54 (m, 1H; CH_{aryl}), 7.49–7.43 (m, 3H; CH_{aryl}), 7.40–7.25 (m, 7H; CH_{aryl}), 7.20–7.16 (m, 2H; CH_{aryl}), 2.17 (appears as s, 6H; CH_3) ppm. $^{13}C\{^1H\}$ NMR (75 MHz, $CDCl_3$, 298.0 K): δ = 165.6 (s; C=O), 138.3 (s; C_{aryl}), 137.2 (s; C_{aryl}), 135.6 (s; C_{aryl}), 129.5 (s; C_{aryl}), 129.4 (s; C_{aryl}), 129.0 (s; C_{aryl}), 128.9 (s; C_{aryl}), 128.8 (s; C_{aryl}), 128.7 (s; C_{aryl}), 128.4 (s; C_{aryl}), 127.9 (s; C_{aryl}), 127.5 (s; C_{aryl}), 124.9 (s; C_{aryl}), 85.8 (s; C_{quart}), 83.4 (s; C_{quart}), 72.4 (s; C_{quart}), 31.1 (s; CH_3) ppm. MS (ESI) *m/z* (%): 356.16 $[M–H]^+$. Elemental analysis calcd for $C_{24}H_{21}NO_2$: C 81.10, H 5.96, N 3.94, found: C 80.76, H 6.36, N 4.27.

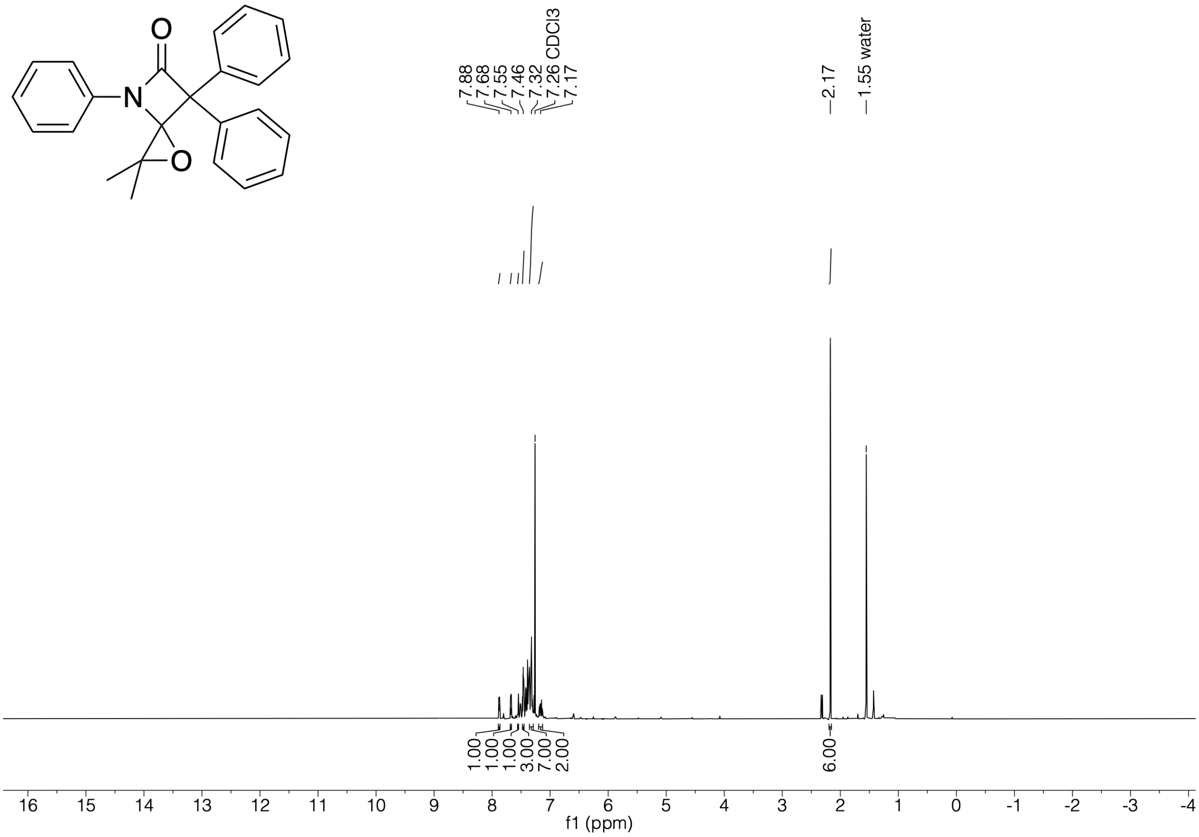

Figure SF173. ^{1}H NMR (300 MHz, $CDCl_3$, 298.0 K) spectrum of **Ph-11-Ph$_2$**.

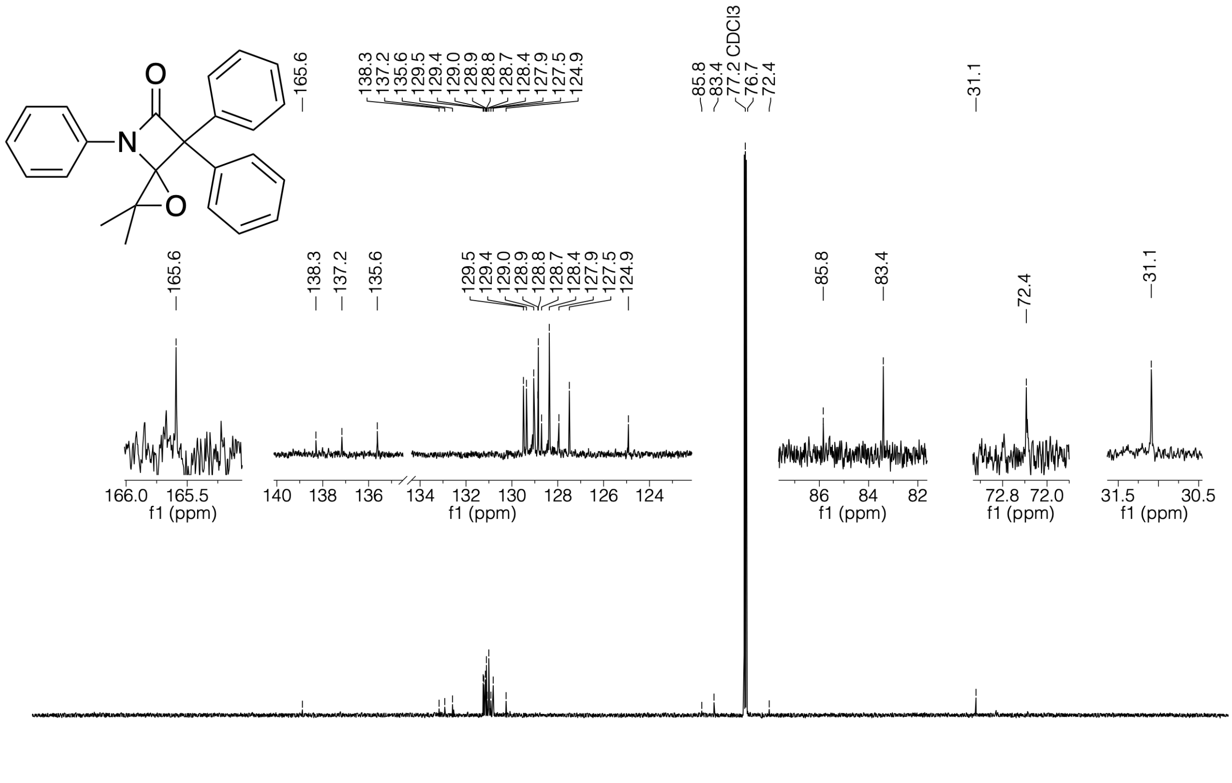

Figure SF174. ^{13}C{^{1}H} NMR (75 MHz, $CDCl_3$, 298.0 K) spectrum of **Ph-11-Ph$_2$**.

m. Reactions of precursors 6*HCl with NaHMDS

Synthesis of Dipp-6-Ph$_2$: 50 mg of **Dipp-6-Ph$_2$*HCl** (0.1 mmol, 1 eq.) and 18.3 mg NaHMDS (0.1 mmol, 1 eq.) were dissolved in 0.6 ml tetrahydrofuran-d$_8$ while stirring at -80 °C. After 2 h, the cold bath was removed and stirring continued at -25 °C for 4 h. Due to the high air sensitivity of the carbene and its sensitivity to temperatures above -20°C, purification was not possible and the reaction solution was immediately analyzed by NMR spectroscopy. Other methods of analysis were therefore not possible. A ^{1}H NMR (600 MHz, THF-d$_8$, 243.0 K) was recorded, but as the solution of raw product was used, no clear assignment of peaks in the ^{1}H NMR is possible. ^{13}C{^{1}H} NMR (150 MHz, THF-d$_8$, 243.0 K): δ = 287.2 (s; C$_{carbene}$), 170.1 (s; C=O), 136.9, 135.9, 130.4, 129.7, 129.3., 129.0, 128.5, 127.9, 86.6, 28.4, 20.5 ppm. A chemical shift δ = 287.2 (s; C$_{carbene}$) ppm clearly indicates the presence of the carbene.

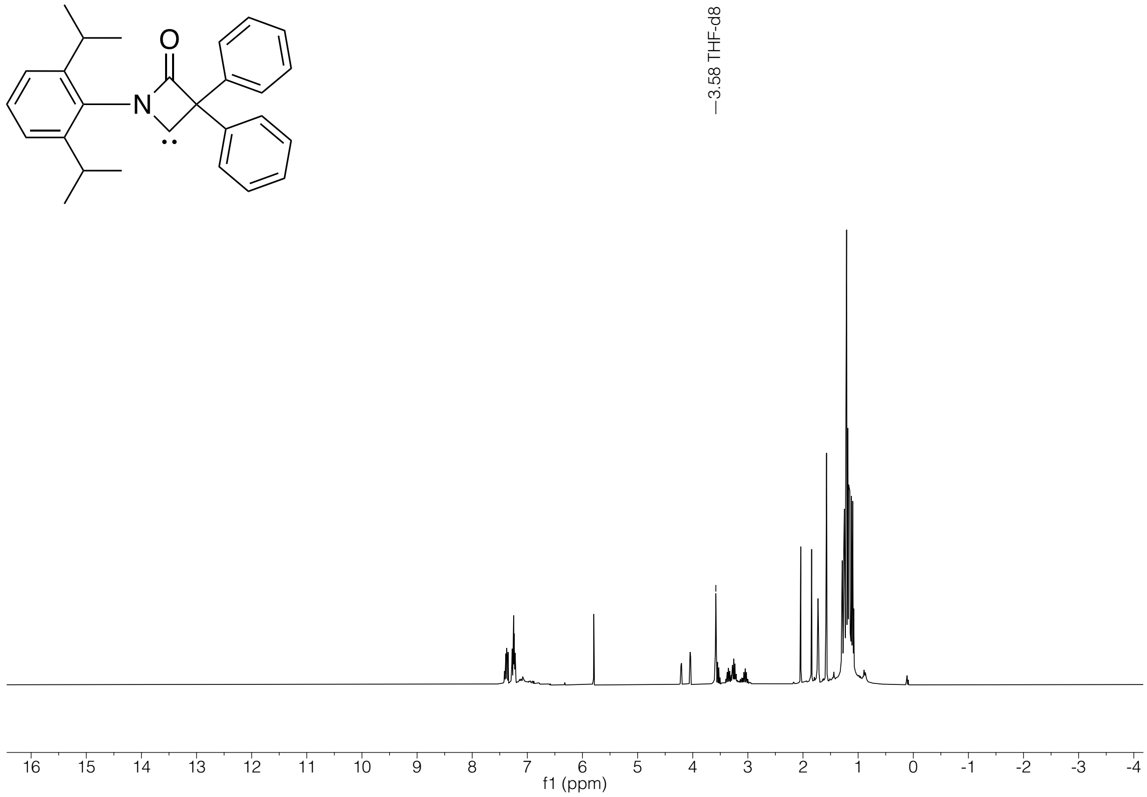

Figure SF175. ^{1}H NMR (600 MHz**,** THF-d$_8$, 243.0 K) spectrum of **Dipp-6-Ph$_2$**.

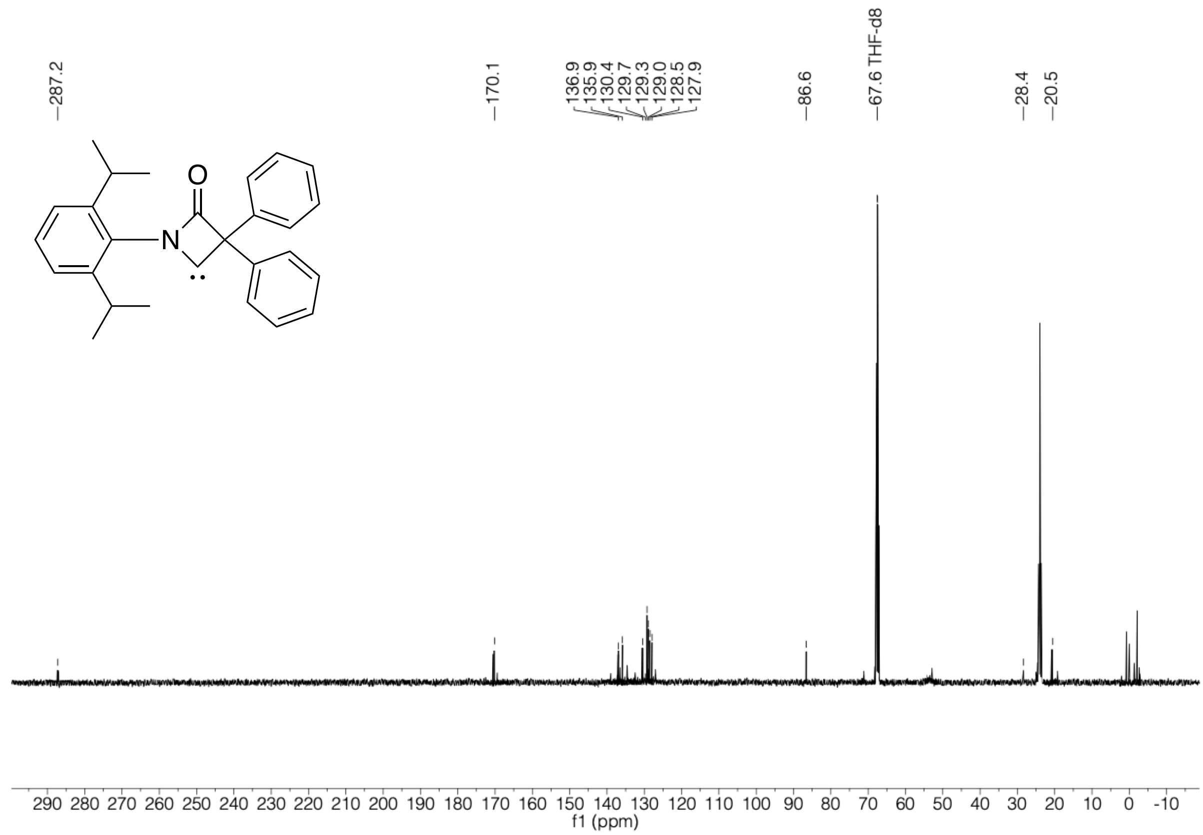

Figure SF176. $^{13}C\{^{1}H\}$ NMR (150 MHz, THF-d_8, 243.0 K) spectrum of **Dipp-6-Ph$_2$**.

Synthesis of dimers [R^1-6-R$^2{}_2$]$_2$: 0.5 mmol (1 eq.) of precursor **R^1-6-R$^2{}_2$*HCl** were dissolved in 10 ml tetrahydrofuran and 0.6 ml NaHMDS in THF (1 M, 0.6 mmol, 1.2 eq.) were added while stirring at -80 °C. After 2 h, the cold bath was removed and stirring continued at ambient temperature for 14 h. All volatiles were removed in vacuo and the resulting colorless powder was suspended in 3 ml of dichloromethane, filtered over silica and eluted with dichloromethane. The desired product was obtained after removal of all volatiles in vacuo as air-stable powder.

[Ph-6-Me$_2$]$_2$: Precursor used: **Ph-6-Me$_2$*HCl** (105 mg, 0.5 mmol). Colorless, air-stable powder. Yield: 83 mg, 96%. Analytical data is matching the data from other synthetic routes, see section *Thermolysis of precursors* ***5***.

[Mes-6-Me$_2$]$_2$: Precursor used: **Mes-6-Me$_2$*HCl** (126 mg, 0.5 mmol). Colorless, air-stable powder. Yield: 105 mg, 98%. Analytical data is matching the data from other synthetic routes, see section *Thermolysis of precursors* ***5***.

[Dipp-6-Me$_2$]$_2$: Precursor used: **Dipp-6-Me$_2$*HCl** (147 mg, 0.5 mmol). Colorless, air-stable powder. Yield: 122 mg, 95%. Analytical data is matching the data from other synthetic routes, see section *Thermolysis of precursors* ***5***.

[Ph-6-Cl$_2$]$_2$: Precursor used: **Ph-6-Cl$_2$*HCl** (125 mg, 0.5 mmol). Colorless, air-stable powder. Yield: 97 mg, 91%. Analytical data is matching the data from other synthetic routes, see section *Thermolysis of precursors* ***5***.

[Ph-6-Ph$_2$]$_2$: Precursor used: **Ph-6-Ph$_2$*HCl** (167 mg, 0.5 mmol). Colorless, air-stable powder. Yield: 146 mg, 98%. Analytical data is matching the data from other synthetic routes, see section *Thermolysis of precursors* ***5***.

III. Crystallographic Data

III.1 Crystal Structure of 1-Mes

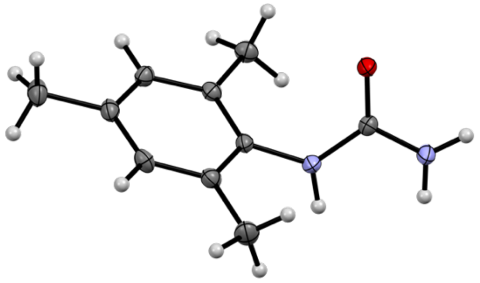

Figure SF177. Molecular structure of 1-Mes. Thermal ellipsoids are shown with 50% probability.

Table ST2 Crystal data and structure refinement for 1-Mes.

CCDC number	2423709
Empirical formula	$C_{10}H_{14}N_2O$
Formula weight	178.23
Temperature/K	99.98(10)
Crystal system	monoclinic
Space group	$P2_1/c$
a/Å	13.9513(2)
b/Å	7.98300(10)
c/Å	8.62130(10)
α/°	90
β/°	95.451(2)
γ/°	90
Volume/Å^3	955.84(2)
Z	4
ρ_{calc}g/cm^3	1.239
μ/mm^{-1}	0.652
F(000)	384.0
Crystal size/mm^3	0.2 × 0.1 × 0.1
Radiation	Cu Kα (λ = 1.54184)
2Θ range for data collection/°	6.364 to 155.77
Index ranges	-16 ≤ h ≤ 16, -8 ≤ k ≤ 10, -10 ≤ l ≤ 10
Reflections collected	7355
Independent reflections	1883 [R_{int} = 0.0224, R_{sigma} = 0.0186]
Data/restraints/parameters	1883/0/133
Goodness-of-fit on F^2	1.091
Final R indexes [I>=2σ (I)]	R_1 = 0.0374, wR_2 = 0.1021
Final R indexes [all data]	R_1 = 0.0393, wR_2 = 0.1037
Largest diff. peak/hole / e Å^{-3}	0.24/-0.27

III.2 Crystal Structure of 1-Dipp(*DMSO)

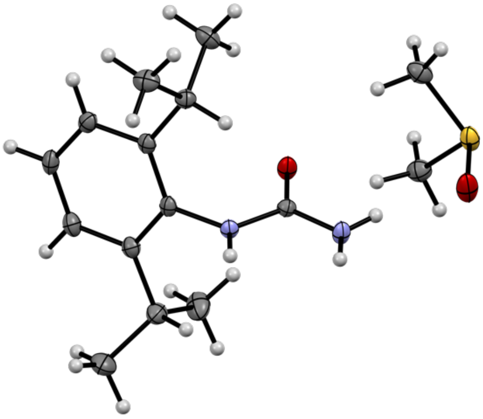

Figure SF178. Molecular structure of 1-Dipp*DMSO. Thermal ellipsoids are shown with 50% probability.

Table ST3 Crystal data and structure refinement for 1-Dipp*DMSO.

CCDC number	2423708
Empirical formula	$C_{15}H_{26}N_2O_2S$
Formula weight	298.44
Temperature/K	100.0(3)
Crystal system	monoclinic
Space group	$P2_1/c$
a/Å	17.09365(18)
b/Å	5.10809(5)
c/Å	20.3518(2)
α/°	90
β/°	111.1620(12)
γ/°	90
Volume/Å^3	1657.20(3)
Z	4
ρ_{calc}g/cm^3	1.196
μ/mm^{-1}	1.759
F(000)	648.0
Crystal size/mm^3	0.3 × 0.1 × 0.1
Radiation	Cu Kα (λ = 1.54184)
2Θ range for data collection/°	5.544 to 155.89
Index ranges	-21 ≤ h ≤ 16, -6 ≤ k ≤ 6, -25 ≤ l ≤ 24
Reflections collected	19308
Independent reflections	3329 [R_{int} = 0.0255, R_{sigma} = 0.0162]
Data/restraints/parameters	3329/0/197
Goodness-of-fit on F^2	1.065
Final R indexes [I>=2σ (I)]	R_1 = 0.0279, wR_2 = 0.0767
Final R indexes [all data]	R_1 = 0.0289, wR_2 = 0.0775
Largest diff. peak/hole / e Å^{-3}	0.27/-0.32

III.3 Crystal Structure of 2-Dipp

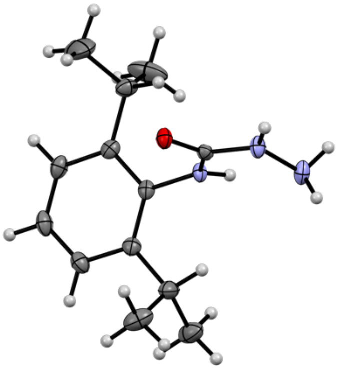

Figure SF179. Molecular structure of 2-Dipp. Thermal ellipsoids are shown with 50% probability.

Table ST4 Crystal data and structure refinement for 2-Dipp.

CCDC number	2423710
Empirical formula	$C_{13}H_{21}N_3O$
Formula weight	235.33
Temperature/K	99.97(17)
Crystal system	trigonal
Space group	R3
a/Å	24.0080(3)
b/Å	24.0080(3)
c/Å	6.16310(10)
α/°	90
β/°	90
γ/°	120
Volume/Å^3	3076.39(9)
Z	9
ρ_{calc}g/cm^3	1.143
μ/mm^{-1}	0.587
F(000)	1152.0
Crystal size/mm^3	0.2 × 0.1 × 0.1
Radiation	Cu Kα (λ = 1.54184)
2Θ range for data collection/°	7.364 to 154.926
Index ranges	-29 ≤ h ≤ 28, -27 ≤ k ≤ 29, -7 ≤ l ≤ 7
Reflections collected	15332
Independent reflections	2689 [R_{int} = 0.0286, R_{sigma} = 0.0177]
Data/restraints/parameters	2689/1/164
Goodness-of-fit on F^2	1.054
Final R indexes [I>=2σ (I)]	R_1 = 0.0285, wR_2 = 0.0714
Final R indexes [all data]	R_1 = 0.0292, wR_2 = 0.0720
Largest diff. peak/hole / e Å^{-3}	0.20/-0.20
Flack parameter	0.04(8)

III.4 Crystal Structure of 3-Ph

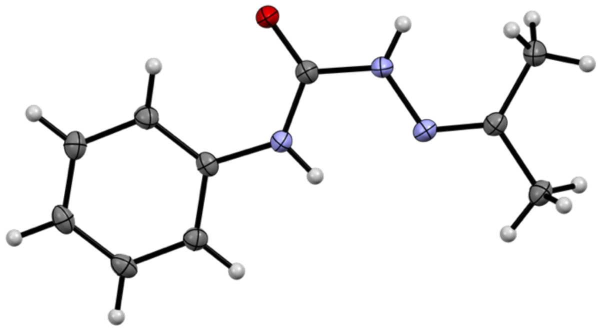

Figure SF180. Molecular structure of 3-Ph. Thermal ellipsoids are shown with 50% probability.

Table ST5 Crystal data and structure refinement for 3-Ph.

CCDC number	2423713
Empirical formula	$C_{10}H_{13}N_3O$
Formula weight	191.23
Temperature/K	99.9(5)
Crystal system	monoclinic
Space group	$P2_1/c$
a/Å	13.29323(14)
b/Å	5.31237(4)
c/Å	15.8552(2)
α/°	90
β/°	114.5435(14)
γ/°	90
Volume/Å^3	1018.51(2)
Z	4
ρ_{calc}g/cm^3	1.247
μ/mm^{-1}	0.680
F(000)	408.0
Crystal size/mm^3	0.3 × 0.2 × 0.2
Radiation	Cu Kα (λ = 1.54184)
2Θ range for data collection/°	7.31 to 155.906
Index ranges	-16 ≤ h ≤ 16, -6 ≤ k ≤ 5, -19 ≤ l ≤ 19
Reflections collected	22113
Independent reflections	2117 [R_{int} = 0.0344, R_{sigma} = 0.0145]
Data/restraints/parameters	2117/0/130
Goodness-of-fit on F^2	1.071
Final R indexes [I>=2σ (I)]	R_1 = 0.0348, wR_2 = 0.0935
Final R indexes [all data]	R_1 = 0.0363, wR_2 = 0.0948
Largest diff. peak/hole / e Å^{-3}	0.21/-0.25

III.5 Crystal Structure of 3-Mes

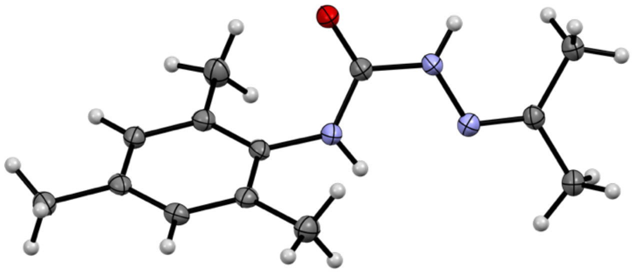

Figure SF181. Molecular structure of 3-Mes. Thermal ellipsoids are shown with 50% probability.

Table ST6 Crystal data and structure refinement for 3-Mes.

CCDC number	2423712
Empirical formula	$C_{13}H_{19}N_3O$
Formula weight	233.31
Temperature/K	99.97(13)
Crystal system	triclinic
Space group	P-1
a/Å	7.7126(2)
b/Å	7.9161(2)
c/Å	11.2551(3)
α/°	98.965(2)
β/°	109.401(2)
γ/°	94.353(2)
Volume/Å^3	634.19(3)
Z	2
ρ_{calc}g/cm^3	1.222
μ/mm^{-1}	0.632
F(000)	252.0
Crystal size/mm^3	0.1 × 0.1 × 0.1
Radiation	Cu Kα (λ = 1.54184)
2Θ range for data collection/°	8.488 to 155.444
Index ranges	-9 ≤ h ≤ 9, -9 ≤ k ≤ 10, -13 ≤ l ≤ 14
Reflections collected	14367
Independent reflections	2551 [R_{int} = 0.0218, R_{sigma} = 0.0127]
Data/restraints/parameters	2551/0/164
Goodness-of-fit on F^2	1.098
Final R indexes [I>=2σ (I)]	R_1 = 0.0349, wR_2 = 0.0941
Final R indexes [all data]	R_1 = 0.0363, wR_2 = 0.0950
Largest diff. peak/hole / e Å^{-3}	0.21/-0.21

III.6 Crystal Structure of 3-Dipp

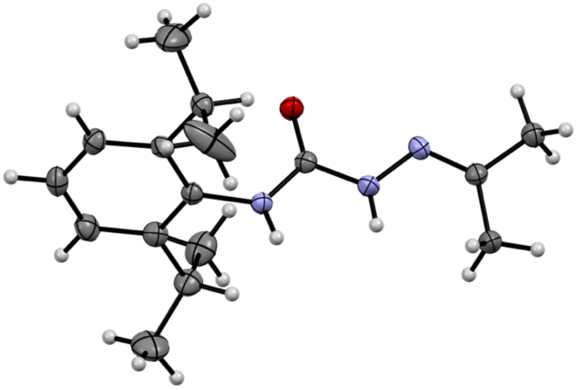

Figure SF182. Molecular structure of 3-Dipp. Thermal ellipsoids are shown with 50% probability.

Table ST7 Crystal data and structure refinement for 3-Dipp.

CCDC number	2423711
Empirical formula	$C_{16}H_{25}N_3O$
Formula weight	275.39
Temperature/K	100.0(4)
Crystal system	orthorhombic
Space group	Pbca
a/Å	15.7405(2)
b/Å	17.0903(2)
c/Å	24.2248(3)
α/°	90
β/°	90
γ/°	90
Volume/Å^3	6516.71(14)
Z	16
ρ_{calc}g/cm^3	1.123
μ/mm^{-1}	0.559
F(000)	2400.0
Crystal size/mm^3	0.3 × 0.05 × 0.05
Radiation	Cu Kα (λ = 1.54184)
2Θ range for data collection/°	7.298 to 155.278
Index ranges	-19 ≤ h ≤ 18, -20 ≤ k ≤ 20, -24 ≤ l ≤ 30
Reflections collected	26926
Independent reflections	6286 [R_{int} = 0.0344, R_{sigma} = 0.0274]
Data/restraints/parameters	6286/0/373
Goodness-of-fit on F^2	1.036
Final R indexes [I>=2σ (I)]	R_1 = 0.0454, wR_2 = 0.1177
Final R indexes [all data]	R_1 = 0.0525, wR_2 = 0.1233
Largest diff. peak/hole / e Å^{-3}	0.49/-0.37

III.7 Crystal Structure of 4-Ph

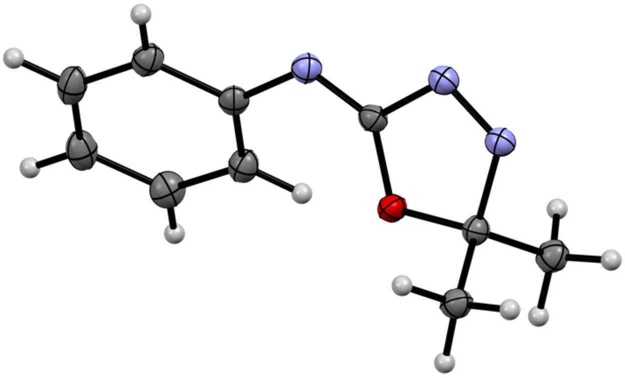

Figure SF183. Molecular structure of 4-Ph. Thermal ellipsoids are shown with 50% probability.

Table ST8 Crystal data and structure refinement for 4-Ph.

CCDC number	2423716
Empirical formula	$C_{10}H_{11}N_3O$
Formula weight	189.22
Temperature/K	102(3)
Crystal system	monoclinic
Space group	$P2_1/c$
a/Å	12.5180(2)
b/Å	6.18379(8)
c/Å	13.6698(2)
α/°	90
β/°	113.579(2)
γ/°	90
Volume/Å^3	969.82(3)
Z	4
ρ_{calc}g/cm^3	1.296
μ/mm^{-1}	0.713
F(000)	400.0
Crystal size/mm^3	0.2 × 0.1 × 0.1
Radiation	Cu Kα (λ = 1.54184)
2Θ range for data collection/°	7.706 to 155.156
Index ranges	-13 ≤ h ≤ 15, -7 ≤ k ≤ 7, -16 ≤ l ≤ 16
Reflections collected	17195
Independent reflections	1995 [R_{int} = 0.0238, R_{sigma} = 0.0108]
Data/restraints/parameters	1995/0/130
Goodness-of-fit on F^2	1.097
Final R indexes [I>=2σ (I)]	R_1 = 0.0330, wR_2 = 0.0830
Final R indexes [all data]	R_1 = 0.0344, wR_2 = 0.0837
Largest diff. peak/hole / e Å^{-3}	0.24/-0.18

III.8 Crystal Structure of 4-Mes

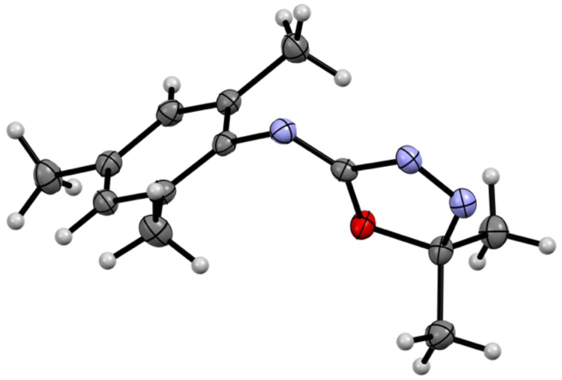

Figure SF184. Molecular structure of 4-Mes. Thermal ellipsoids are shown with 50% probability.

Table ST9 Crystal data and structure refinement for 4-Mes.

CCDC number	2423715
Empirical formula	$C_{13}H_{17}N_3O$
Formula weight	231.29
Temperature/K	105(8)
Crystal system	monoclinic
Space group	$P2_1/n$
a/Å	8.7277(2)
b/Å	18.2507(3)
c/Å	8.9751(2)
α/°	90
β/°	115.951(2)
γ/°	90
Volume/Å^3	1285.46(5)
Z	4
ρ_{calc}g/cm^3	1.195
μ/mm^{-1}	0.623
F(000)	496.0
Crystal size/mm^3	0.3 × 0.1 × 0.05
Radiation	Cu Kα (λ = 1.54184)
2Θ range for data collection/°	9.692 to 155.76
Index ranges	-8 ≤ h ≤ 11, -22 ≤ k ≤ 22, -11 ≤ l ≤ 10
Reflections collected	14540
Independent reflections	2550 [R_{int} = 0.0211, R_{sigma} = 0.0123]
Data/restraints/parameters	2550/0/160
Goodness-of-fit on F^2	1.087
Final R indexes [I>=2σ (I)]	R_1 = 0.0336, wR_2 = 0.0906
Final R indexes [all data]	R_1 = 0.0352, wR_2 = 0.0918
Largest diff. peak/hole / e Å^{-3}	0.23/-0.20

III.9 Crystal Structure of 4-Dipp

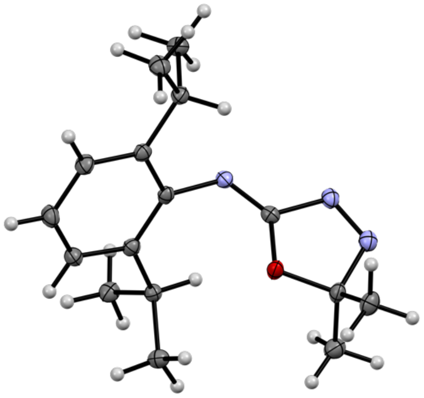

Figure SF185. Molecular structure of 4-Dipp. Thermal ellipsoids are shown with 50% probability.

Table ST10 Crystal data and structure refinement for 4-Dipp.

CCDC number	2423714
Empirical formula	$C_{16}H_{23}N_3O$
Formula weight	273.37
Temperature/K	99.9(5)
Crystal system	triclinic
Space group	P-1
a/Å	8.06400(12)
b/Å	8.38206(12)
c/Å	11.82623(19)
α/°	90.1619(12)
β/°	90.6310(12)
γ/°	109.5132(13)
Volume/Å^3	753.39(2)
Z	2
ρ_{calc}g/cm^3	1.205
μ/mm^{-1}	0.604
F(000)	296.0
Crystal size/mm^3	0.2 × 0.2 × 0.1
Radiation	Cu Kα (λ = 1.54184)
2Θ range for data collection/°	7.476 to 155.508
Index ranges	-10 ≤ h ≤ 10, -9 ≤ k ≤ 10, -14 ≤ l ≤ 14
Reflections collected	16925
Independent reflections	3003 [R_{int} = 0.0199, R_{sigma} = 0.0113]
Data/restraints/parameters	3003/0/188
Goodness-of-fit on F^2	1.067
Final R indexes [I>=2σ (I)]	R_1 = 0.0313, wR_2 = 0.0805
Final R indexes [all data]	R_1 = 0.0324, wR_2 = 0.0813
Largest diff. peak/hole / e Å^{-3}	0.30/-0.18

III.10 Crystal Structure of Ph-5-Me$_2$

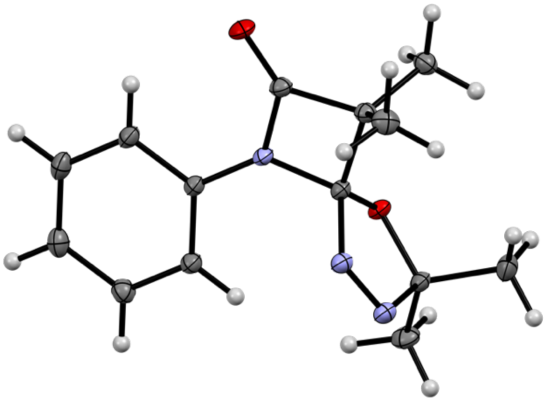

Figure SF186. Molecular structure of Ph-5-Me$_2$. Thermal ellipsoids are shown with 50% probability.

Table ST11 Crystal data and structure refinement for Ph-5-Me$_2$.

CCDC number	2423737
Empirical formula	$C_{14}H_{17}N_3O_2$
Formula weight	259.30
Temperature/K	100.0(2)
Crystal system	triclinic
Space group	P-1
a/Å	9.2091(2)
b/Å	9.5259(2)
c/Å	9.6835(2)
α/°	107.760(2)
β/°	100.859(2)
γ/°	117.094(2)
Volume/Å^3	664.38(3)
Z	2
ρ_{calc}g/cm^3	1.296
μ/mm^{-1}	0.089
F(000)	276.0
Crystal size/mm^3	0.15 × 0.1 × 0.1
Radiation	Mo Kα (λ = 0.71073)
2Θ range for data collection/°	4.788 to 82.384
Index ranges	-16 ≤ h ≤ 16, -17 ≤ k ≤ 17, -17 ≤ l ≤ 17
Reflections collected	34119
Independent reflections	8605 [R_{int} = 0.0382, R_{sigma} = 0.0360]
Data/restraints/parameters	8605/0/176
Goodness-of-fit on F^2	1.058
Final R indexes [I>=2σ (I)]	R_1 = 0.0433, wR_2 = 0.1218
Final R indexes [all data]	R_1 = 0.0596, wR_2 = 0.1309
Largest diff. peak/hole / e Å^{-3}	0.61/-0.25

III.11 Crystal Structure of Ph-5-Cl_2

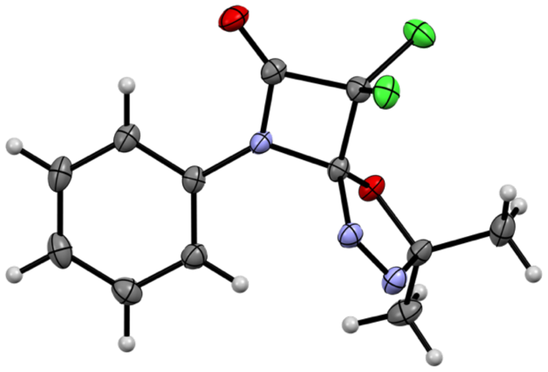

Figure SF187. Molecular structure of Ph-5-Cl_2. Thermal ellipsoids are shown with 50% probability.

Table ST12 Crystal data and structure refinement for Ph-5-Cl_2.

CCDC number	2423736
Empirical formula	$C_{12}H_{11}Cl_2N_3O_2$
Formula weight	300.14
Temperature/K	99.98(10)
Crystal system	triclinic
Space group	P-1
a/Å	9.0755(2)
b/Å	9.5394(2)
c/Å	9.6628(2)
α/°	119.327(2)
β/°	106.348(2)
γ/°	98.950(2)
Volume/Å^3	655.39(3)
Z	2
ρ_{calc}g/cm^3	1.521
μ/mm^{-1}	4.484
F(000)	308.0
Crystal size/mm^3	0.1 × 0.08 × 0.05
Radiation	Cu Kα (λ = 1.54184)
2Θ range for data collection/°	10.708 to 155.964
Index ranges	-11 ≤ h ≤ 11, -11 ≤ k ≤ 12, -12 ≤ l ≤ 10
Reflections collected	12281
Independent reflections	2615 [R_{int} = 0.0299, R_{sigma} = 0.0177]
Data/restraints/parameters	2615/0/174
Goodness-of-fit on F^2	1.037
Final R indexes [I>=2σ (I)]	R_1 = 0.0298, wR_2 = 0.0782
Final R indexes [all data]	R_1 = 0.0304, wR_2 = 0.0787
Largest diff. peak/hole / e Å^{-3}	0.29/-0.38

III.12 Crystal Structure of Ph-5-Ph$_2$

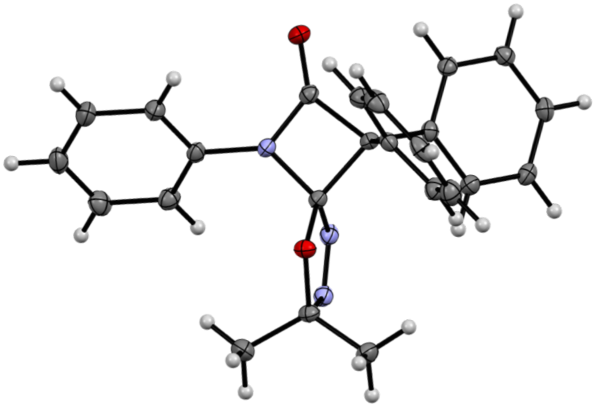

Figure SF188. Molecular structure of Ph-5-Ph$_2$. Thermal ellipsoids are shown with 50% probability.

Table ST13 Crystal data and structure refinement for Ph-5-Ph$_2$.

CCDC number	2423738
Empirical formula	$C_{24}H_{21}N_3O_2$
Formula weight	383.44
Temperature/K	108(4)
Crystal system	monoclinic
Space group	I2/a
a/Å	13.3710(2)
b/Å	16.8234(2)
c/Å	17.8491(2)
α/°	90
β/°	108.8970(10)
γ/°	90
Volume/Å^3	3798.67(9)
Z	8
ρ_{calc}g/cm^3	1.341
μ/mm^{-1}	0.695
F(000)	1616.0
Crystal size/mm^3	0.2 × 0.1 × 0.1
Radiation	Cu Kα (λ = 1.54184)
2Θ range for data collection/°	7.418 to 155.656
Index ranges	-16 ≤ h ≤ 16, -20 ≤ k ≤ 20, -21 ≤ l ≤ 22
Reflections collected	40183
Independent reflections	3795 [R_{int} = 0.0379, R_{sigma} = 0.0129]
Data/restraints/parameters	3795/0/265
Goodness-of-fit on F^2	1.058
Final R indexes [I>=2σ (I)]	R_1 = 0.0334, wR_2 = 0.0832
Final R indexes [all data]	R_1 = 0.0340, wR_2 = 0.0837
Largest diff. peak/hole / e Å^{-3}	0.33/-0.18

III.13 Crystal Structure of Ph-5-sCy

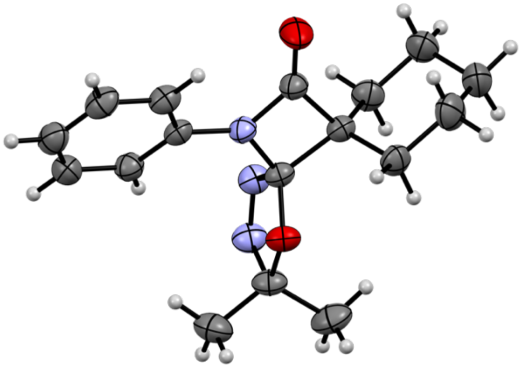

Figure SF189. Molecular structure of Ph-5-sCy. Thermal ellipsoids are shown with 50% probability.

Table ST14 Crystal data and structure refinement for Ph-5-sCy.

CCDC number	2423739
Empirical formula	$C_{17}H_{21}N_3O_2$
Formula weight	299.37
Temperature/K	221(1)
Crystal system	monoclinic
Space group	$P2_1/n$
a/Å	11.24068(8)
b/Å	11.98071(9)
c/Å	12.18436(8)
α/°	90
β/°	103.4641(7)
γ/°	90
Volume/Å^3	1595.79(2)
Z	4
ρ_{calc}g/cm^3	1.246
μ/mm^{-1}	0.669
F(000)	640.0
Crystal size/mm^3	0.2 × 0.15 × 0.1
Radiation	Cu Kα (λ = 1.54184)
2Θ range for data collection/°	9.646 to 153.362
Index ranges	$-11 \le h \le 14$, $-14 \le k \le 14$, $-15 \le l \le 14$
Reflections collected	35384
Independent reflections	3318 [R_{int} = 0.0232, R_{sigma} = 0.0096]
Data/restraints/parameters	3318/0/202
Goodness-of-fit on F^2	1.062
Final R indexes [I>=2σ (I)]	R_1 = 0.0370, wR_2 = 0.0948
Final R indexes [all data]	R_1 = 0.0381, wR_2 = 0.0955
Largest diff. peak/hole / e Å^{-3}	0.26/-0.25

III.14 Crystal Structure of Mes-5-Me_2

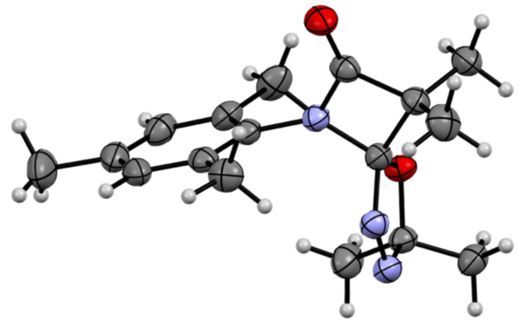

Figure SF190. Molecular structure of Mes-5-Me_2. Thermal ellipsoids are shown with 50% probability.

Table ST15 Crystal data and structure refinement for Mes-5-Me_2.

CCDC number	2423727
Empirical formula	$C_{17}H_{23}N_3O_2$
Formula weight	301.38
Temperature/K	150.00(10)
Crystal system	monoclinic
Space group	$P2_1$
a/Å	7.81910(10)
b/Å	9.09080(10)
c/Å	12.2976(2)
α/°	90
β/°	98.011(2)
γ/°	90
Volume/Å^3	865.61(2)
Z	2
ρ_{calc}g/cm^3	1.156
μ/mm^{-1}	0.617
F(000)	324.0
Crystal size/mm^3	0.1 × 0.06 × 0.04
Radiation	Cu Kα (λ = 1.54184)
2Θ range for data collection/°	7.26 to 158.368
Index ranges	-9 ≤ h ≤ 9, -11 ≤ k ≤ 11, -14 ≤ l ≤ 15
Reflections collected	25594
Independent reflections	3503 [R_{int} = 0.0344, R_{sigma} = 0.0160]
Data/restraints/parameters	3503/1/206
Goodness-of-fit on F^2	1.068
Final R indexes [I>=2σ (I)]	R_1 = 0.0292, wR_2 = 0.0766
Final R indexes [all data]	R_1 = 0.0302, wR_2 = 0.0774
Largest diff. peak/hole / e Å^{-3}	0.14/-0.13
Flack parameter	0.36(8)

III.15 Crystal Structure of Mes-5-sCy

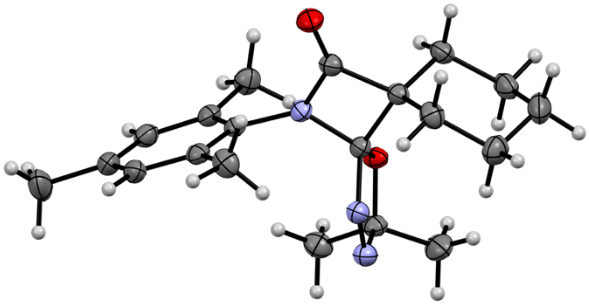

Figure SF191. Molecular structure of Mes-5-sCy. Thermal ellipsoids are shown with 50% probability.

Table ST16 Crystal data and structure refinement for Mes-5-sCy.

CCDC number	2423728
Empirical formula	$C_{20}H_{27}N_3O_2$
Formula weight	341.44
Temperature/K	150.00(10)
Crystal system	orthorhombic
Space group	$Pca2_1$
a/Å	15.83861(7)
b/Å	8.85917(4)
c/Å	13.24275(7)
α/°	90
β/°	90
γ/°	90
Volume/Å^3	1858.183(15)
Z	4
ρ_{calc}g/cm^3	1.221
μ/mm^{-1}	0.633
F(000)	736.0
Crystal size/mm^3	0.2 × 0.1 × 0.1
Radiation	Cu Kα (λ = 1.54184)
2Θ range for data collection/°	9.984 to 155.372
Index ranges	-16 ≤ h ≤ 19, -11 ≤ k ≤ 11, -16 ≤ l ≤ 16
Reflections collected	53263
Independent reflections	3853 [R_{int} = 0.0301, R_{sigma} = 0.0101]
Data/restraints/parameters	3853/1/232
Goodness-of-fit on F^2	1.049
Final R indexes [I>=2σ (I)]	R_1 = 0.0268, wR_2 = 0.0738
Final R indexes [all data]	R_1 = 0.0269, wR_2 = 0.0739
Largest diff. peak/hole / e Å^{-3}	0.20/-0.14
Flack parameter	0.01(3)

III.16 Crystal Structure of Dipp-5-Me₂

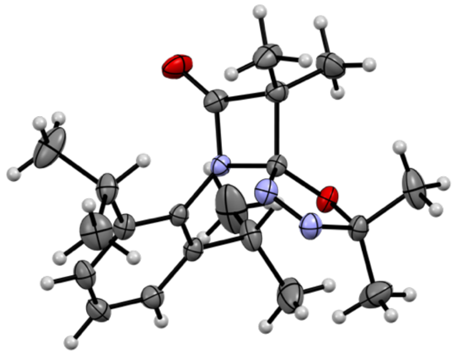

Figure SF192. Molecular structure of Dipp-5-Me$_2$. Thermal ellipsoids are shown with 50% probability.

Table ST17 Crystal data and structure refinement for Dipp-5-Me$_2$.

CCDC number	2423718
Empirical formula	$C_{20}H_{29}N_3O_2$
Formula weight	343.46
Temperature/K	100.00(10)
Crystal system	orthorhombic
Space group	Pbca
a/Å	9.08910(10)
b/Å	16.54480(10)
c/Å	26.2182(2)
α/°	90
β/°	90
γ/°	90
Volume/Å^3	3942.62(6)
Z	8
ρ_{calc}g/cm^3	1.157
μ/mm^{-1}	0.597
F(000)	1488.0
Crystal size/mm^3	0.2 × 0.1 × 0.1
Radiation	Cu Kα (λ = 1.54184)
2Θ range for data collection/°	6.742 to 155.414
Index ranges	-11 ≤ h ≤ 10, -20 ≤ k ≤ 19, -33 ≤ l ≤ 27
Reflections collected	41906
Independent reflections	4032 [R_{int} = 0.0434, R_{sigma} = 0.0152]
Data/restraints/parameters	4032/0/235
Goodness-of-fit on F^2	1.038
Final R indexes [I>=2σ (I)]	R_1 = 0.0577, wR_2 = 0.1463
Final R indexes [all data]	R_1 = 0.0587, wR_2 = 0.1471
Largest diff. peak/hole / e Å^{-3}	0.79/-0.49

III.17 Crystal Structure of Dipp-5-Cl_2

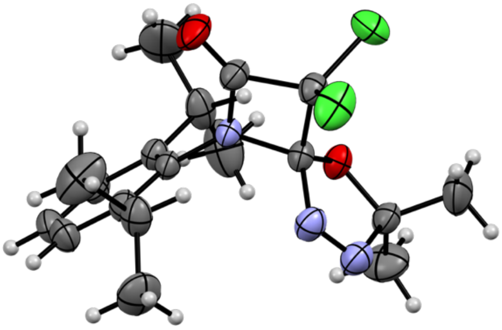

Figure SF193. Molecular structure of Dipp-5-Cl_2. Thermal ellipsoids are shown with 50% probability.

Table ST18 Crystal data and structure refinement for Dipp-5-Cl_2.

CCDC number	2423717
Empirical formula	$C_{18}H_{23}Cl_2N_3O_2$
Formula weight	384.29
Temperature/K	200.0(3)
Crystal system	orthorhombic
Space group	Pbca
a/Å	9.11590(10)
b/Å	16.61420(10)
c/Å	26.1506(2)
α/°	90
β/°	90
γ/°	90
Volume/Å^3	3960.60(6)
Z	8
ρ_{calc}g/cm^3	1.289
μ/mm^{-1}	3.078
F(000)	1616.0
Crystal size/mm^3	0.2 × 0.15 × 0.1
Radiation	Cu Kα (λ = 1.54184)
2Θ range for data collection/°	6.76 to 154.398
Index ranges	$-11 \le h \le 11$, $-21 \le k \le 21$, $-32 \le l \le 30$
Reflections collected	41809
Independent reflections	4110 [R_{int} = 0.0433, R_{sigma} = 0.0167]
Data/restraints/parameters	4110/0/232
Goodness-of-fit on F^2	1.037
Final R indexes [I>=2σ (I)]	R_1 = 0.0500, wR_2 = 0.1356
Final R indexes [all data]	R_1 = 0.0521, wR_2 = 0.1370
Largest diff. peak/hole / e Å^{-3}	0.42/-0.37

III.18 Crystal Structure of Dipp-5-sCy

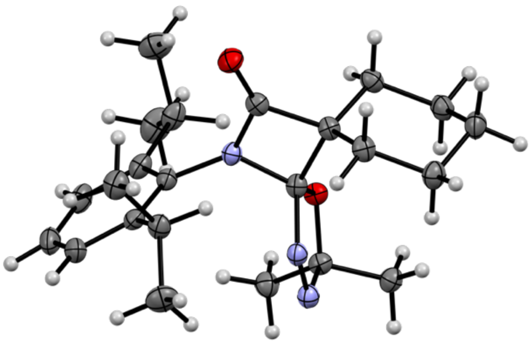

Figure SF194. Molecular structure of Dipp-5-sCy. Thermal ellipsoids are shown with 50% probability.

Table ST19 Crystal data and structure refinement for Dipp-5-sCy.

CCDC number	2423719
Empirical formula	$C_{23}H_{33}N_3O_2$
Formula weight	383.52
Temperature/K	99.97(13)
Crystal system	monoclinic
Space group	Cc
a/Å	14.05800(10)
b/Å	9.29530(10)
c/Å	16.6183(2)
α/°	90
β/°	101.2250(10)
γ/°	90
Volume/Å^3	2130.03(4)
Z	4
ρ_{calc}g/cm^3	1.196
μ/mm^{-1}	0.604
F(000)	832.0
Crystal size/mm^3	0.25 × 0.1 × 0.1
Radiation	Cu Kα (λ = 1.54184)
2Θ range for data collection/°	10.856 to 155.398
Index ranges	-17 ≤ h ≤ 17, -11 ≤ k ≤ 11, -17 ≤ l ≤ 20
Reflections collected	23176
Independent reflections	3965 [R_{int} = 0.0215, R_{sigma} = 0.0134]
Data/restraints/parameters	3965/2/260
Goodness-of-fit on F^2	1.075
Final R indexes [I>=2σ (I)]	R_1 = 0.0234, wR_2 = 0.0613
Final R indexes [all data]	R_1 = 0.0236, wR_2 = 0.0614
Largest diff. peak/hole / e Å^{-3}	0.16/-0.12
Flack parameter	-0.02(4)

III.19 Crystal Structure of Ph-6-Cl_2*HCl

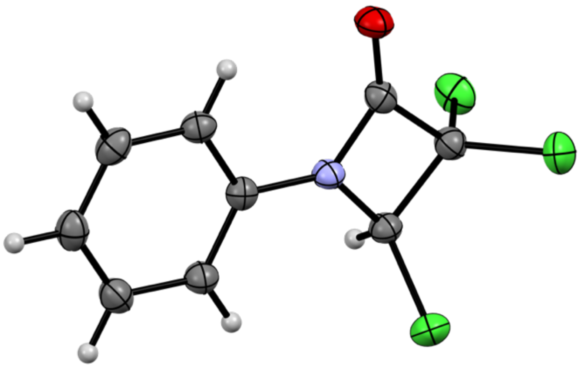

Figure SF195. Molecular structure of Ph-6-Cl_2*HCl. Thermal ellipsoids are shown with 50% probability.

Table ST20 Crystal data and structure refinement for Ph-6-Cl_2*HCl.

CCDC number	2423740
Empirical formula	$C_9H_6Cl_3NO$
Formula weight	250.50
Temperature/K	150.00(10)
Crystal system	monoclinic
Space group	$P2_1/n$
a/Å	12.2088(2)
b/Å	5.83260(10)
c/Å	14.5408(2)
α/°	90
β/°	94.2570(10)
γ/°	90
Volume/Å^3	1032.58(3)
Z	4
ρ_{calc}g/cm^3	1.611
μ/mm^{-1}	7.751
F(000)	504.0
Crystal size/mm^3	0.1 × 0.1 × 0.05
Radiation	Cu Kα (λ = 1.54184)
2Θ range for data collection/°	9.132 to 155.074
Index ranges	-15 ≤ h ≤ 15, -7 ≤ k ≤ 7, -18 ≤ l ≤ 17
Reflections collected	17895
Independent reflections	2079 [R_{int} = 0.0303, R_{sigma} = 0.0160]
Data/restraints/parameters	2079/0/127
Goodness-of-fit on F^2	1.064
Final R indexes [I>=2σ (I)]	R_1 = 0.0257, wR_2 = 0.0646
Final R indexes [all data]	R_1 = 0.0276, wR_2 = 0.0659
Largest diff. peak/hole / e Å^{-3}	0.28/-0.24

III.20 Crystal Structure of $Ph\text{-}6\text{-}Ph_2$*HCl

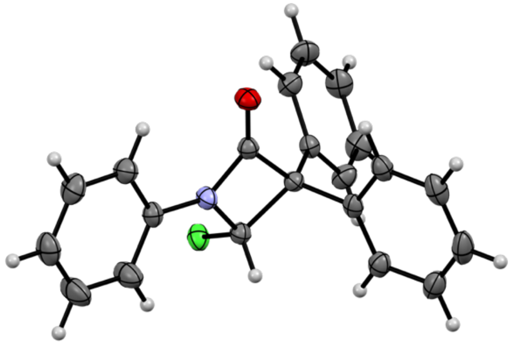

Figure SF196. Molecular structure of $Ph\text{-}6\text{-}Ph_2$*HCl. Thermal ellipsoids are shown with 50% probability.

Table ST21 Crystal data and structure refinement for $Ph\text{-}6\text{-}Ph_2$*HCl.

CCDC number	2423743
Empirical formula	$C_{21}H_{16}ClNO$
Formula weight	333.80
Temperature/K	104(7)
Crystal system	monoclinic
Space group	$P2_1/c$
a/Å	17.0105(2)
b/Å	6.34120(10)
c/Å	15.8200(2)
α/°	90
β/°	102.3660(10)
γ/°	90
Volume/Å^3	1666.86(4)
Z	4
ρ_{calc}g/cm^3	1.330
μ/mm^{-1}	2.067
F(000)	696.0
Crystal size/mm^3	0.2 × 0.1 × 0.1
Radiation	Cu Kα (λ = 1.54184)
2Θ range for data collection/°	5.318 to 155.392
Index ranges	$-21 \le h \le 20$, $-7 \le k \le 7$, $-19 \le l \le 19$
Reflections collected	28399
Independent reflections	3216 [R_{int} = 0.0401, R_{sigma} = 0.0151]
Data/restraints/parameters	3216/0/218
Goodness-of-fit on F^2	1.055
Final R indexes [I>=2σ (I)]	R_1 = 0.0324, wR_2 = 0.0837
Final R indexes [all data]	R_1 = 0.0330, wR_2 = 0.0842
Largest diff. peak/hole / e Å^{-3}	0.28/-0.29

III.21 Crystal Structure of Mes-6-Ph$_2$*HCl

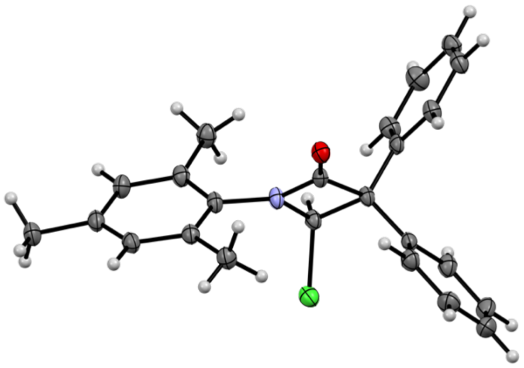

Figure SF197. Molecular structure of Mes-6-Ph$_2$*HCl. Thermal ellipsoids are shown with 50% probability.

Table ST22 Crystal data and structure refinement for Mes-6-Ph$_2$*HCl.

CCDC number	2423730
Empirical formula	$C_{24}H_{22}ClNO$
Formula weight	375.87
Temperature/K	100.00(10)
Crystal system	triclinic
Space group	P-1
a/Å	6.26830(10)
b/Å	8.45260(10)
c/Å	18.3541(2)
α/°	91.0280(10)
β/°	95.3300(10)
γ/°	91.8980(10)
Volume/Å^3	967.51(2)
Z	2
ρ_{calc}g/cm^3	1.290
μ/mm^{-1}	1.838
F(000)	396.0
Crystal size/mm^3	0.2 × 0.05 × 0.05
Radiation	Cu Kα (λ = 1.54184)
2Θ range for data collection/°	4.836 to 155.172
Index ranges	-7 ≤ h ≤ 7, -10 ≤ k ≤ 10, -20 ≤ l ≤ 22
Reflections collected	54558
Independent reflections	3822 [R_{int} = 0.0615, R_{sigma} = 0.0202]
Data/restraints/parameters	3822/0/247
Goodness-of-fit on F^2	1.040
Final R indexes [I>=2σ (I)]	R_1 = 0.0473, wR_2 = 0.1333
Final R indexes [all data]	R_1 = 0.0483, wR_2 = 0.1342
Largest diff. peak/hole / e Å^{-3}	0.85/-0.53

III.22 Crystal Structure of Dipp-6-Me_2*HCl

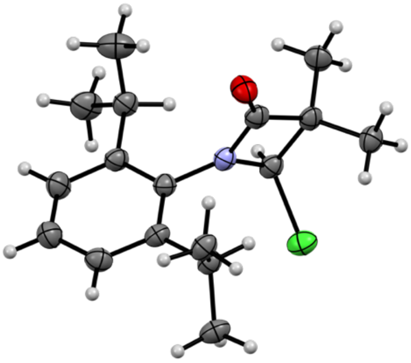

Figure SF198. Molecular structure of Dipp-6-Me_2*HCl. Thermal ellipsoids are shown with 50% probability.

Table ST23 Crystal data and structure refinement for Dipp-6-Me_2*HCl.

CCDC number	2423721
Empirical formula	$C_{17}H_{24}ClNO$
Formula weight	293.82
Temperature/K	100.00(10)
Crystal system	monoclinic
Space group	$P2_1/n$
a/Å	16.9409(4)
b/Å	11.2734(2)
c/Å	19.4773(5)
α/°	90
β/°	115.632(3)
γ/°	90
Volume/Å^3	3353.74(15)
Z	8
ρ_{calc}g/cm^3	1.164
μ/mm^{-1}	1.971
F(000)	1264.0
Crystal size/mm^3	0.1 × 0.05 × 0.05
Radiation	Cu Kα (λ = 1.54184)
2Θ range for data collection/°	9.172 to 155.99
Index ranges	-20 ≤ h ≤ 20, -14 ≤ k ≤ 14, -24 ≤ l ≤ 22
Reflections collected	48521
Independent reflections	6807 [R_{int} = 0.0587, R_{sigma} = 0.0295]
Data/restraints/parameters	6807/0/373
Goodness-of-fit on F^2	1.160
Final R indexes [I>=2σ (I)]	R_1 = 0.0839, wR_2 = 0.2082
Final R indexes [all data]	R_1 = 0.0880, wR_2 = 0.2100
Largest diff. peak/hole / e Å^{-3}	0.43/-0.60

III.23 Crystal Structure of Dipp-6-Ph_2*HCl(*0.5 benzene)

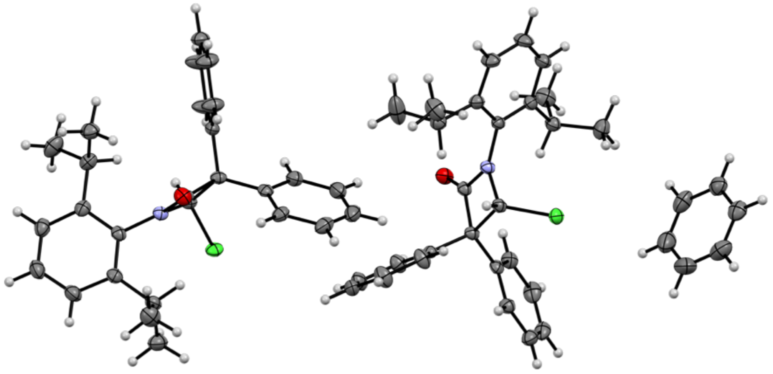

Figure SF199. Molecular structure of Dipp-6-Ph_2*HCl(*0.5 benzene). Thermal ellipsoids are shown with 50% probability.

Table ST24 Crystal data and structure refinement for Dipp-6-Ph_2*HCl.

CCDC number	2423723
Empirical formula	$C_{60}H_{62}Cl_2N_2O_2$
Formula weight	457.01
Temperature/K	100.00(10)
Crystal system	triclinic
Space group	P-1
a/Å	6.21380(10)
b/Å	20.3702(2)
c/Å	21.39920(10)
α/°	68.9940(10)
β/°	81.8690(10)
γ/°	83.2470(10)
Volume/Å^3	2496.64(5)
Z	2
ρ_{calc}g/cm^3	1.216
μ/mm^{-1}	1.512
F(000)	972.0
Crystal size/mm^3	0.2 × 0.05 × 0.05
Radiation	Cu Kα (λ = 1.54184)
2Θ range for data collection/°	4.448 to 156.34
Index ranges	-7 ≤ h ≤ 7, -24 ≤ k ≤ 25, -25 ≤ l ≤ 26
Reflections collected	140592
Independent reflections	9938 [R_{int} = 0.0709, R_{sigma} = 0.0248]
Data/restraints/parameters	9938/0/604
Goodness-of-fit on F^2	1.023
Final R indexes [I>=2σ (I)]	R_1 = 0.0511, wR_2 = 0.1264
Final R indexes [all data]	R_1 = 0.0545, wR_2 = 0.1284
Largest diff. peak/hole / e Å^{-3}	0.31/-0.60

III.24 Crystal Structure of Ph-6-Ph_2*Se

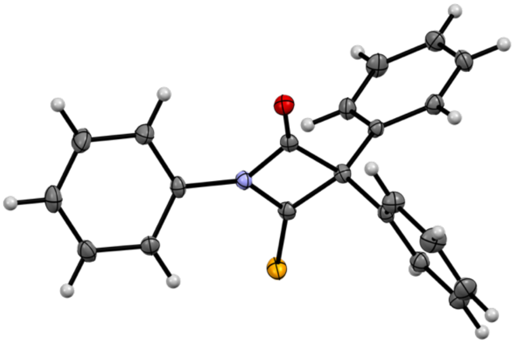

Figure SF200. Molecular structure of Ph-6-Ph_2*Se. Thermal ellipsoids are shown with 50% probability.

Table ST25 Crystal data and structure refinement for Ph-6-Ph_2*Se.

CCDC number	2423744
Empirical formula	$C_{21}H_{15}NOSe$
Formula weight	752.60
Temperature/K	99.8(3)
Crystal system	monoclinic
Space group	C2/c
a/Å	15.14630(10)
b/Å	12.89330(10)
c/Å	34.5630(2)
α/°	90
β/°	97.4350(10)
γ/°	90
Volume/Å^3	6692.91(8)
Z	16
ρ_{calc}g/cm^3	1.494
μ/mm^{-1}	3.078
F(000)	3040.0
Crystal size/mm^3	0.3 × 0.1 × 0.1
Radiation	Cu Kα (λ = 1.54184)
2Θ range for data collection/°	5.156 to 152.982
Index ranges	-18 ≤ h ≤ 14, -15 ≤ k ≤ 15, -43 ≤ l ≤ 43
Reflections collected	49931
Independent reflections	6677 [R_{int} = 0.0399, R_{sigma} = 0.0154]
Data/restraints/parameters	6677/0/433
Goodness-of-fit on F^2	1.088
Final R indexes [I>=2σ (I)]	R_1 = 0.0267, wR_2 = 0.0661
Final R indexes [all data]	R_1 = 0.0269, wR_2 = 0.0663
Largest diff. peak/hole / e Å^{-3}	0.33/-0.51

III.25 Crystal Structure of Mes-6-sCy*Se

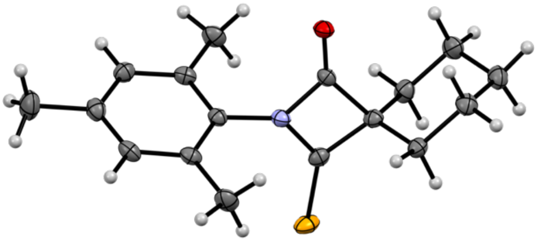

Figure SF201. Molecular structure of Mes-6-sCy*Se. Thermal ellipsoids are shown with 50% probability.

Table ST26 Crystal data and structure refinement for Mes-6-sCy*Se.

CCDC number	2423733
Empirical formula	$C_{17}H_{21}NOSe$
Formula weight	334.31
Temperature/K	100.00(10)
Crystal system	monoclinic
Space group	C2/c
a/Å	19.9021(2)
b/Å	12.54360(10)
c/Å	13.19980(10)
α/°	90
β/°	101.8640(10)
γ/°	90
Volume/Å^3	3224.86(5)
Z	8
ρ_{calc}g/cm^3	1.377
μ/mm^{-1}	3.106
F(000)	1376.0
Crystal size/mm^3	0.2 × 0.05 × 0.05
Radiation	Cu Kα (λ = 1.54184)
2Θ range for data collection/°	8.384 to 153.008
Index ranges	-24 ≤ h ≤ 23, -15 ≤ k ≤ 15, -16 ≤ l ≤ 15
Reflections collected	34942
Independent reflections	3352 [R_{int} = 0.0823, R_{sigma} = 0.0258]
Data/restraints/parameters	3352/0/184
Goodness-of-fit on F^2	1.080
Final R indexes [I>=2σ (I)]	R_1 = 0.0313, wR_2 = 0.0849
Final R indexes [all data]	R_1 = 0.0319, wR_2 = 0.0854
Largest diff. peak/hole / e Å^{-3}	0.39/-0.59

III.26 Crystal Structure of Mes-6-sCy*S

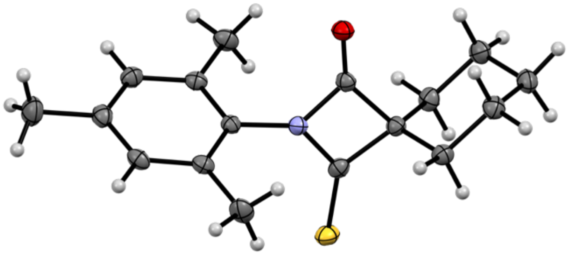

Figure SF202. Molecular structure of Mes-6-sCy*S. Thermal ellipsoids are shown with 50% probability.

Table ST27 Crystal data and structure refinement for Mes-6-sCy*S.

CCDC number	2423732
Empirical formula	$C_{17}H_{21}NOS$
Formula weight	287.41
Temperature/K	100.00(10)
Crystal system	monoclinic
Space group	C2/c
a/Å	19.4974(2)
b/Å	12.45960(12)
c/Å	13.15732(14)
α/°	90
β/°	101.2367(11)
γ/°	90
Volume/Å^3	3135.03(6)
Z	8
ρ_{calc}g/cm^3	1.218
μ/mm^{-1}	1.783
F(000)	1232.0
Crystal size/mm^3	0.15 × 0.15 × 0.1
Radiation	Cu Kα (λ = 1.54184)
2Θ range for data collection/°	8.47 to 154.576
Index ranges	-24 ≤ h ≤ 24, -15 ≤ k ≤ 14, -16 ≤ l ≤ 16
Reflections collected	23413
Independent reflections	3184 [R_{int} = 0.0616, R_{sigma} = 0.0238]
Data/restraints/parameters	3184/0/185
Goodness-of-fit on F^2	1.090
Final R indexes [I>=2σ (I)]	R_1 = 0.0362, wR_2 = 0.1003
Final R indexes [all data]	R_1 = 0.0368, wR_2 = 0.1008
Largest diff. peak/hole / e Å^{-3}	0.31/-0.26

III.27 Crystal Structure of Ph-6-Ph_2*[Ir(COD)Cl]

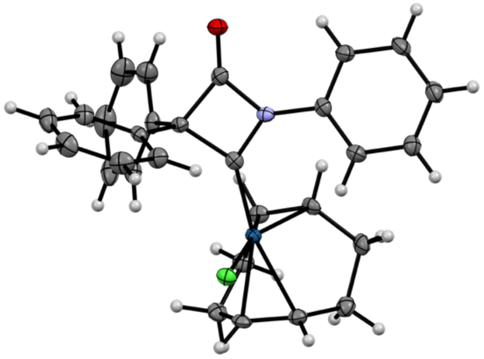

Figure SF203. Molecular structure of Ph-6-Ph_2*[Ir(COD)Cl].Thermal ellipsoids are shown with 50% probability.

Table ST28 Crystal data and structure refinement for Ph-6-Ph_2*[Ir(COD)Cl].

CCDC number	2423741
Empirical formula	$C_{29}H_{27}ClIrNO$
Formula weight	633.16
Temperature/K	100.3(7)
Crystal system	monoclinic
Space group	$P2_1/n$
a/Å	16.3477(2)
b/Å	8.82110(10)
c/Å	16.7246(2)
α/°	90
β/°	104.7480(10)
γ/°	90
Volume/Å^3	2332.31(5)
Z	4
ρ_{calc}g/cm^3	1.803
μ/mm^{-1}	12.304
F(000)	1240.0
Crystal size/mm^3	0.13 × 0.1 × 0.1
Radiation	Cu Kα (λ = 1.54184)
2Θ range for data collection/°	6.75 to 156.14
Index ranges	-20 ≤ h ≤ 20, -10 ≤ k ≤ 11, -20 ≤ l ≤ 14
Reflections collected	22632
Independent reflections	4629 [R_{int} = 0.0225, R_{sigma} = 0.0172]
Data/restraints/parameters	4629/0/298
Goodness-of-fit on F^2	1.068
Final R indexes [I>=2σ (I)]	R_1 = 0.0189, wR_2 = 0.0493
Final R indexes [all data]	R_1 = 0.0206, wR_2 = 0.0501
Largest diff. peak/hole / e Å^{-3}	0.88/-0.93

III.28 Crystal Structure of Ph-6-Ph$_2$*[Rh(COD)Cl]

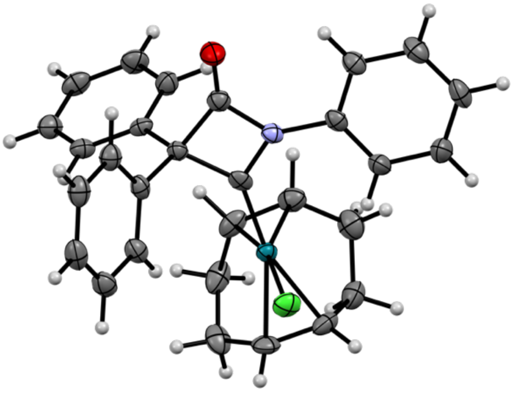

Figure SF204. Molecular structure of Ph-6-Ph$_2$*[Rh(COD)Cl].Thermal ellipsoids are shown with 50% probability.

Table ST29 Crystal data and structure refinement for Ph-6-Ph$_2$*[Rh(COD)Cl].

CCDC number	2423742
Empirical formula	$C_{29}H_{27}ClNORh$
Formula weight	543.87
Temperature/K	99.96(16)
Crystal system	monoclinic
Space group	$P2_1/n$
a/Å	7.9665(2)
b/Å	18.6306(3)
c/Å	16.0614(3)
α/°	90
β/°	90.316(2)
γ/°	90
Volume/Å^3	2383.81(8)
Z	4
ρ_{calc}g/cm^3	1.515
μ/mm^{-1}	6.987
F(000)	1112.0
Crystal size/mm^3	0.08 × 0.03 × 0.003
Radiation	Cu Kα (λ = 1.54184)
2Θ range for data collection/°	7.266 to 155.984
Index ranges	-10 ≤ h ≤ 9, -23 ≤ k ≤ 22, -9 ≤ l ≤ 20
Reflections collected	23438
Independent reflections	4482 [R_{int} = 0.0442, R_{sigma} = 0.0287]
Data/restraints/parameters	4482/0/298
Goodness-of-fit on F^2	1.185
Final R indexes [I>=2σ (I)]	R_1 = 0.0482, wR_2 = 0.1042
Final R indexes [all data]	R_1 = 0.0521, wR_2 = 0.1058
Largest diff. peak/hole / e Å^{-3}	1.36/-1.18

III.29 Crystal Structure of Mes-6-Ph$_2$*[Ir(COD)Cl]

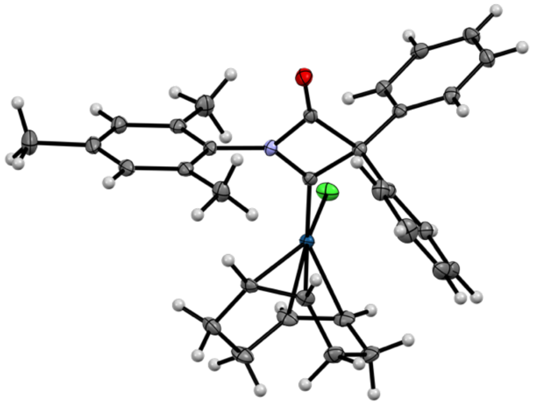

Figure SF205. Molecular structure of Mes-6-Ph$_2$*[Ir(COD)Cl].Thermal ellipsoids are shown with 50% probability.

Table ST30 Crystal data and structure refinement for Mes-6-Ph$_2$*[Ir(COD)Cl].

CCDC number	2423729
Empirical formula	$C_{32}H_{33}ClIrNO$
Formula weight	675.24
Temperature/K	99.99(10)
Crystal system	monoclinic
Space group	$P2_1/n$
a/Å	16.57160(10)
b/Å	10.174
c/Å	17.03790(10)
α/°	90
β/°	112.4550(10)
γ/°	90
Volume/Å^3	2654.75(3)
Z	4
ρ_{calc}g/cm^3	1.689
μ/mm^{-1}	10.851
F(000)	1336.0
Crystal size/mm^3	0.12 × 0.1 × 0.1
Radiation	Cu Kα (λ = 1.54184)
2Θ range for data collection/°	6.33 to 155.496
Index ranges	-21 ≤ h ≤ 20, -12 ≤ k ≤ 12, -18 ≤ l ≤ 21
Reflections collected	77489
Independent reflections	5514 [R_{int} = 0.0652, R_{sigma} = 0.0185]
Data/restraints/parameters	5514/0/329
Goodness-of-fit on F^2	1.155
Final R indexes [I>=2σ (I)]	R_1 = 0.0233, wR_2 = 0.0631
Final R indexes [all data]	R_1 = 0.0234, wR_2 = 0.0632
Largest diff. peak/hole / e Å^{-3}	0.67/-1.11

III.30 Crystal Structure of Mes-6-sCy*[Ir(COD)Cl]

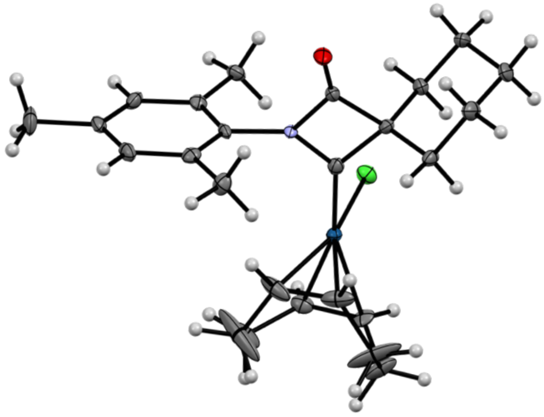

Figure SF206. Molecular structure of Mes-6-sCy*[Ir(COD)Cl].Thermal ellipsoids are shown with 50% probability.

Table ST31 Crystal data and structure refinement for Mes-6-sCy*[Ir(COD)Cl.

CCDC number	2423731
Empirical formula	$C_{25}H_{33}ClIrNO$
Formula weight	591.17
Temperature/K	100.00(10)
Crystal system	orthorhombic
Space group	Pbca
a/Å	13.14280(10)
b/Å	13.04950(10)
c/Å	26.1145(2)
α/°	90
β/°	90
γ/°	90
Volume/Å^3	4478.82(6)
Z	8
ρ_{calc}g/cm^3	1.753
μ/mm^{-1}	12.751
F(000)	2336.0
Crystal size/mm^3	0.2 × 0.1 × 0.05
Radiation	Cu Kα (λ = 1.54184)
2Θ range for data collection/°	6.77 to 155.988
Index ranges	-15 ≤ h ≤ 16, -16 ≤ k ≤ 13, -33 ≤ l ≤ 24
Reflections collected	34306
Independent reflections	4360 [R_{int} = 0.0637, R_{sigma} = 0.0285]
Data/restraints/parameters	4360/0/266
Goodness-of-fit on F^2	1.174
Final R indexes [I>=2σ (I)]	R_1 = 0.0374, wR_2 = 0.0991
Final R indexes [all data]	R_1 = 0.0381, wR_2 = 0.0996
Largest diff. peak/hole / e Å^{-3}	1.42/-1.40

III.31 Crystal Structure of Dipp-6-Me_2*[Rh(COD)Cl]

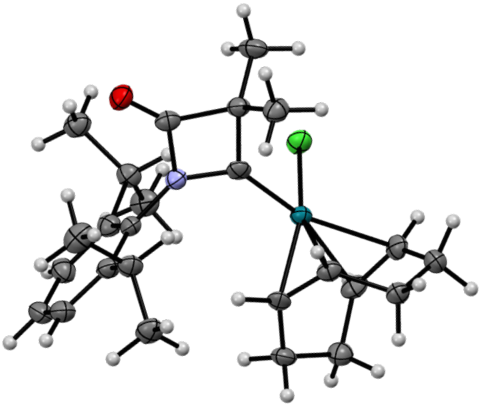

Figure SF207. Molecular structure of Dipp-6-Me_2*[Rh(COD)Cl].Thermal ellipsoids are shown with 50% probability.

Table ST32 Crystal data and structure refinement for Dipp-6-Me_2*[Rh(COD)Cl].

CCDC number	2423720
Empirical formula	$C_{25}H_{35}ClNORh$
Formula weight	503.90
Temperature/K	100.00(10)
Crystal system	monoclinic
Space group	$P2_1/c$
a/Å	10.2601(2)
b/Å	30.4902(4)
c/Å	8.2624(2)
α/°	90
β/°	112.275(2)
γ/°	90
Volume/Å^3	2391.86(9)
Z	4
ρ_{calc}g/cm^3	1.399
μ/mm^{-1}	6.905
F(000)	1048.0
Crystal size/mm^3	0.15 × 0.1 × 0.05
Radiation	Cu Kα (λ = 1.54184)
2Θ range for data collection/°	5.798 to 153.132
Index ranges	$-12 \le h \le 12$, $-35 \le k \le 38$, $-9 \le l \le 10$
Reflections collected	26031
Independent reflections	4786 [R_{int} = 0.0514, R_{sigma} = 0.0246]
Data/restraints/parameters	4786/0/268
Goodness-of-fit on F^2	1.126
Final R indexes [I>=2σ (I)]	R_1 = 0.0411, wR_2 = 0.1035
Final R indexes [all data]	R_1 = 0.0427, wR_2 = 0.1045
Largest diff. peak/hole / e Å^{-3}	1.23/-0.64

III.32 Crystal Structure of Dipp-6-Ph$_2$*[Rh(COD)Cl](*benzene)

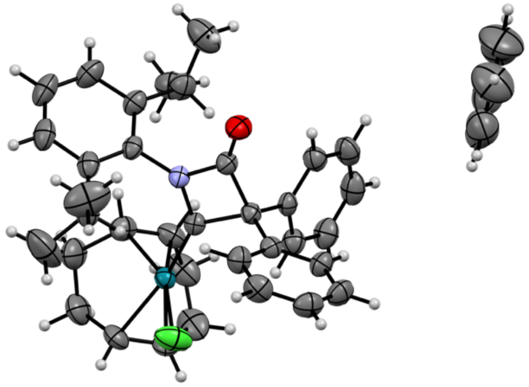

Figure SF208. Molecular structure of Dipp-6-Ph$_2$*[Rh(COD)Cl](*benzene).Thermal ellipsoids are shown with 50% probability.

Table ST33 Crystal data and structure refinement for Dipp-6-Ph$_2$*[Rh(COD)Cl].

CCDC number	2423722
Empirical formula	$C_{41}H_{45}ClNORh$
Formula weight	706.14
Temperature/K	249.99(10)
Crystal system	monoclinic
Space group	$P2_1/c$
a/Å	10.13740(10)
b/Å	17.3493(2)
c/Å	20.18770(10)
α/°	90
β/°	95.2560(10)
γ/°	90
Volume/Å^3	3535.62(6)
Z	4
ρ_{calc}g/cm^3	1.327
μ/mm^{-1}	4.835
F(000)	1472.0
Crystal size/mm^3	0.1 × 0.1 × 0.1
Radiation	Cu Kα (λ = 1.54184)
2Θ range for data collection/°	6.73 to 153.546
Index ranges	-11 ≤ h ≤ 12, -21 ≤ k ≤ 21, -24 ≤ l ≤ 25
Reflections collected	40621
Independent reflections	7220 [R_{int} = 0.0274, R_{sigma} = 0.0181]
Data/restraints/parameters	7220/0/411
Goodness-of-fit on F^2	1.049
Final R indexes [I>=2σ (I)]	R_1 = 0.0296, wR_2 = 0.0798
Final R indexes [all data]	R_1 = 0.0313, wR_2 = 0.0809
Largest diff. peak/hole / e Å^{-3}	0.44/-0.58

III.33 Crystal Structure of Dipp-6-sCy*[Rh(COD)Cl]

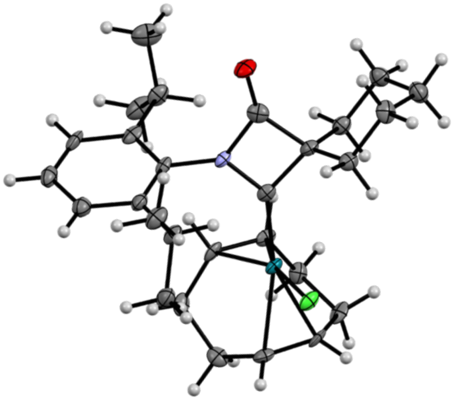

Figure SF209. Molecular structure of Dipp-6-sCy*[Rh(COD)Cl].Thermal ellipsoids are shown with 50% probability.

Table ST34 Crystal data and structure refinement for Dipp-6-sCy*[Rh(COD)Cl].

CCDC number	2423724
Empirical formula	$C_{28}H_{39}ClNORh$
Formula weight	543.96
Temperature/K	100.00(10)
Crystal system	triclinic
Space group	P-1
a/Å	8.0855(2)
b/Å	10.2459(2)
c/Å	16.9469(4)
α/°	91.038(2)
β/°	99.839(2)
γ/°	111.065(2)
Volume/Å^3	1286.01(5)
Z	2
ρ_{calc}g/cm^3	1.405
μ/mm^{-1}	6.463
F(000)	568.0
Crystal size/mm^3	0.4 × 0.04 × 0.03
Radiation	Cu Kα (λ = 1.54184)
2Θ range for data collection/°	5.312 to 152.828
Index ranges	-10 ≤ h ≤ 9, -12 ≤ k ≤ 11, -20 ≤ l ≤ 21
Reflections collected	25109
Independent reflections	5057 [R_{int} = 0.0832, R_{sigma} = 0.0456]
Data/restraints/parameters	5057/0/293
Goodness-of-fit on F^2	1.161
Final R indexes [I>=2σ (I)]	R_1 = 0.0711, wR_2 = 0.1889
Final R indexes [all data]	R_1 = 0.0728, wR_2 = 0.1897
Largest diff. peak/hole / e Å^{-3}	3.54/-1.85

III.34 Crystal Structure of $[Ph\text{-}6\text{-}Cl_2]_2$

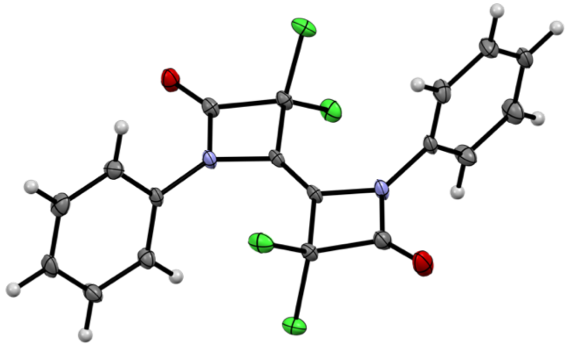

Figure SF210. Molecular structure of $[Ph\text{-}6\text{-}Cl_2]_2$. Thermal ellipsoids are shown with 50% probability.

Table ST35 Crystal data and structure refinement for $[Ph\text{-}6\text{-}Cl_2]_2$.

CCDC number	2423704
Empirical formula	$C_{18}H_{10}Cl_4N_2O_2$
Formula weight	428.08
Temperature/K	99.96(18)
Crystal system	orthorhombic
Space group	Pbca
a/Å	8.34320(10)
b/Å	20.6379(2)
c/Å	20.7487(2)
α/°	90
β/°	90
γ/°	90
Volume/Å^3	3572.64(6)
Z	8
ρ_{calc}g/cm^3	1.592
μ/mm^{-1}	6.167
F(000)	1728.0
Crystal size/mm^3	0.2 × 0.2 × 0.1
Radiation	Cu Kα (λ = 1.54184)
2Θ range for data collection/°	8.524 to 155.05
Index ranges	$-10 \le h \le 10$, $-23 \le k \le 24$, $-26 \le l \le 25$
Reflections collected	32201
Independent reflections	3649 [R_{int} = 0.0250, R_{sigma} = 0.0122]
Data/restraints/parameters	3649/0/236
Goodness-of-fit on F^2	1.090
Final R indexes [I>=2σ (I)]	R_1 = 0.0246, wR_2 = 0.0663
Final R indexes [all data]	R_1 = 0.0252, wR_2 = 0.0666
Largest diff. peak/hole / e Å^{-3}	0.32/-0.23

III.35 Crystal Structure of $[Ph\text{-}6\text{-}Ph_2]_2$

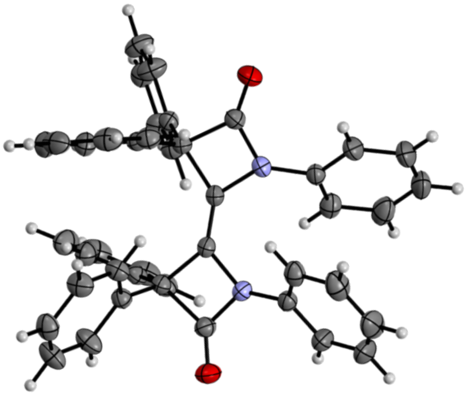

Figure SF211. Molecular structure of $[Ph\text{-}6\text{-}Ph_2]_2$. Thermal ellipsoids are shown with 50% probability.

Table ST36 Crystal data and structure refinement for $[Ph\text{-}6\text{-}Ph_2]_2$.

CCDC number	2423707
Empirical formula	$C_{42}H_{30}N_2O_2$
Formula weight	297.34
Temperature/K	199.9(3)
Crystal system	monoclinic
Space group	I2/a
a/Å	17.7021(2)
b/Å	11.01400(10)
c/Å	16.5775(2)
α/°	90
β/°	104.8490(10)
γ/°	90
Volume/Å^3	3124.19(6)
Z	4
ρ_{calc}g/cm^3	1.264
μ/mm^{-1}	0.608
F(000)	1248.0
Crystal size/mm^3	0.15 × 0.1 × 0.1
Radiation	Cu Kα (λ = 1.54184)
2Θ range for data collection/°	9.55 to 155.506
Index ranges	-22 ≤ h ≤ 19, -13 ≤ k ≤ 13, -19 ≤ l ≤ 21
Reflections collected	18294
Independent reflections	3210 [R_{int} = 0.0292, R_{sigma} = 0.0191]
Data/restraints/parameters	3210/0/209
Goodness-of-fit on F^2	1.054
Final R indexes [I>=2σ (I)]	R_1 = 0.0361, wR_2 = 0.0946
Final R indexes [all data]	R_1 = 0.0398, wR_2 = 0.0971
Largest diff. peak/hole / e Å^{-3}	0.24/-0.15

III.36 Crystal Structure of $[Ph\text{-}6\text{-}Me_2{*}N]_2$

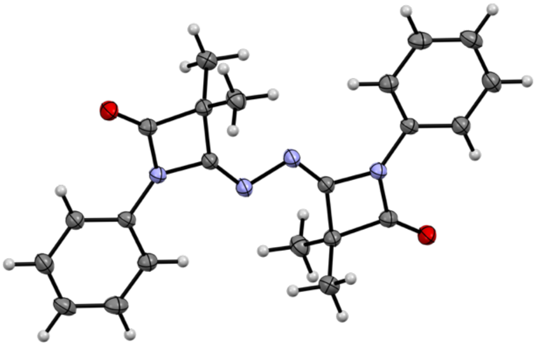

Figure SF212. Molecular structure of $[Ph\text{-}6\text{-}Me_2{*}N]_2$. Thermal ellipsoids are shown with 50% probability.

Table ST37 Crystal data and structure refinement for $[Ph\text{-}6\text{-}Me_2{*}N]_2$.

CCDC number	2423705
Empirical formula	$C_{22}H_{22}N_4O_2$
Formula weight	187.22
Temperature/K	149.99(10)
Crystal system	orthorhombic
Space group	Pmna
a/Å	6.92357(14)
b/Å	8.89165(18)
c/Å	15.6021(3)
α/°	90
β/°	90
γ/°	90
Volume/Å^3	960.49(3)
Z	2
ρ_{calc}g/cm^3	1.295
μ/mm^{-1}	0.686
F(000)	396.0
Crystal size/mm^3	0.15 × 0.12 × 0.1
Radiation	Cu Kα (λ = 1.54184)
2Θ range for data collection/°	9.948 to 154.83
Index ranges	-8 ≤ h ≤ 6, -9 ≤ k ≤ 11, -19 ≤ l ≤ 18
Reflections collected	6239
Independent reflections	1025 [R_{int} = 0.0242, R_{sigma} = 0.0149]
Data/restraints/parameters	1025/0/84
Goodness-of-fit on F^2	1.051
Final R indexes [I>=2σ (I)]	R_1 = 0.0322, wR_2 = 0.0822
Final R indexes [all data]	R_1 = 0.0338, wR_2 = 0.0837
Largest diff. peak/hole / e Å^{-3}	0.29/-0.19

III.37 Crystal Structure of [Ph-6-Cl_2*N]$_2$

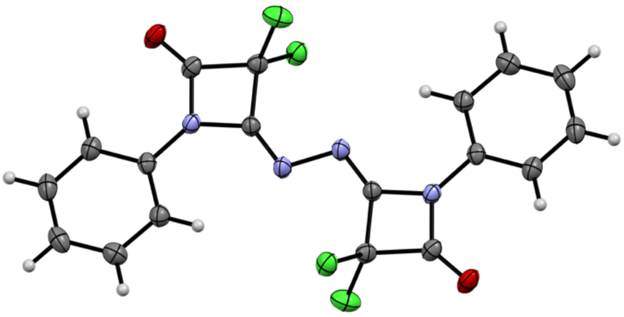

Figure SF213. Molecular structure of [Ph-6-Cl_2*N]$_2$. Thermal ellipsoids are shown with 50% probability.

Table ST38 Crystal data and structure refinement for [Ph-6-Cl_2*N]$_2$.

CCDC number	2423703
Empirical formula	$C_{18}H_{10}Cl_4N_4O_2$
Formula weight	228.05
Temperature/K	150.00(10)
Crystal system	monoclinic
Space group	$P2_1/n$
a/Å	9.55590(10)
b/Å	8.88450(10)
c/Å	11.35690(10)
α/°	90
β/°	100.5500(10)
γ/°	90
Volume/Å^3	947.895(17)
Z	2
ρ_{calc}g/cm^3	1.598
μ/mm^{-1}	5.884
F(000)	460.0
Crystal size/mm^3	0.1 × 0.1 × 0.1
Radiation	Cu Kα (λ = 1.54184)
2Θ range for data collection/°	11.144 to 158.176
Index ranges	-11 ≤ h ≤ 12, -11 ≤ k ≤ 11, -13 ≤ l ≤ 14
Reflections collected	27231
Independent reflections	1998 [R_{int} = 0.0319, R_{sigma} = 0.0111]
Data/restraints/parameters	1998/0/128
Goodness-of-fit on F^2	1.063
Final R indexes [I>=2σ (I)]	R_1 = 0.0249, wR_2 = 0.0659
Final R indexes [all data]	R_1 = 0.0254, wR_2 = 0.0662
Largest diff. peak/hole / e Å^{-3}	0.30/-0.22

III.38 Crystal Structure of [Ph-6-Ph_2*N]$_2$

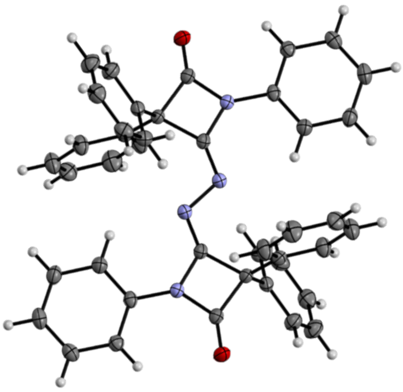

Figure SF214. Molecular structure of [Ph-6-Ph_2*N]$_2$. Thermal ellipsoids are shown with 50% probability.

Table ST39 Crystal data and structure refinement for [Ph-6-Ph_2*N]$_2$.

CCDC number	2423706
Empirical formula	$C_{84}H_{60}N_8O_4$
Formula weight	622.70
Temperature/K	149.99(10)
Crystal system	triclinic
Space group	P-1
a/Å	11.03247(14)
b/Å	11.63096(13)
c/Å	13.06644(14)
α/°	88.9849(9)
β/°	82.7985(10)
γ/°	69.5282(11)
Volume/Å^3	1557.77(3)
Z	1
ρ_{calc}g/cm^3	1.328
μ/mm^{-1}	0.654
F(000)	652.0
Crystal size/mm^3	0.2 × 0.15 × 0.15
Radiation	Cu Kα (λ = 1.54184)
2Θ range for data collection/°	6.822 to 155.276
Index ranges	-13 ≤ h ≤ 13, -14 ≤ k ≤ 13, -16 ≤ l ≤ 15
Reflections collected	55666
Independent reflections	6237 [R_{int} = 0.0367, R_{sigma} = 0.0153]
Data/restraints/parameters	6237/0/434
Goodness-of-fit on F^2	1.033
Final R indexes [I>=2σ (I)]	R_1 = 0.0347, wR_2 = 0.0834
Final R indexes [all data]	R_1 = 0.0369, wR_2 = 0.0848
Largest diff. peak/hole / e Å^{-3}	0.30/-0.17

III.39 Crystal Structure of [Mes-6-Cl_2*N]$_2$

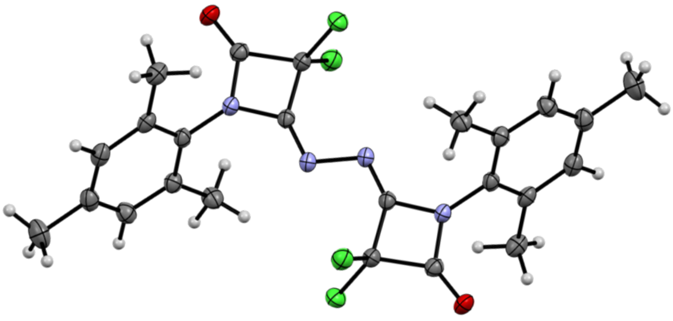

Figure SF215. Molecular structure of [Mes-6-Cl_2*N]$_2$. Thermal ellipsoids are shown with 50% probability.

Table ST40 Crystal data and structure refinement for [Mes-6-Cl_2*N]$_2$.

CCDC number	2423702
Empirical formula	$C_{24}H_{21}Cl_4N_4O_2$
Formula weight	270.13
Temperature/K	100.00(10)
Crystal system	monoclinic
Space group	C2/c
a/Å	13.7686(2)
b/Å	11.4928(2)
c/Å	16.4093(3)
α/°	90
β/°	100.835(2)
γ/°	90
Volume/Å^3	2550.31(8)
Z	4x
ρ_{calc}g/cm^3	1.407
μ/mm^{-1}	4.459
F(000)	1112.0
Crystal size/mm^3	0.1 × 0.05 × 0.05
Radiation	Cu Kα (λ = 1.54184)
2Θ range for data collection/°	10.1 to 152.894
Index ranges	-17 ≤ h ≤ 14, -13 ≤ k ≤ 13, -20 ≤ l ≤ 20
Reflections collected	14317
Independent reflections	2533 [R_{int} = 0.0337, R_{sigma} = 0.0193]
Data/restraints/parameters	2533/0/157
Goodness-of-fit on F^2	1.092
Final R indexes [I>=2σ (I)]	R_1 = 0.0298, wR_2 = 0.0857
Final R indexes [all data]	R_1 = 0.0312, wR_2 = 0.0869
Largest diff. peak/hole / e Å^{-3}	0.31/-0.27

III.40 Crystal Structure of Mes-7-Me$_2$

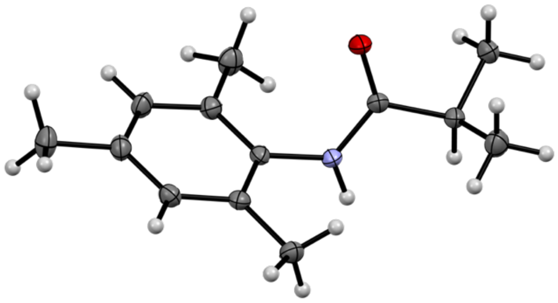

Figure SF216. Molecular structure of Mes-7-Me$_2$. Thermal ellipsoids are shown with 50% probability.

Table ST41 Crystal data and structure refinement for Mes-7-Me$_2$.

CCDC number	2423734
Empirical formula	$C_{13}H_{19}NO$
Formula weight	205.29
Temperature/K	102(4)
Crystal system	monoclinic
Space group	$P2_1/c$
a/Å	4.74830(10)
b/Å	24.3213(3)
c/Å	10.4024(2)
α/°	90
β/°	98.3770(10)
γ/°	90
Volume/Å^3	1188.50(4)
Z	4
ρ_{calc}g/cm^3	1.147
μ/mm^{-1}	0.558
F(000)	448.0
Crystal size/mm^3	0.15 × 0.1 × 0.05
Radiation	Cu Kα (λ = 1.54184)
2Θ range for data collection/°	7.27 to 155.41
Index ranges	$-6 \le h \le 5$, $-24 \le k \le 30$, $-12 \le l \le 13$
Reflections collected	13186
Independent reflections	2339 [R_{int} = 0.0487, R_{sigma} = 0.0238]
Data/restraints/parameters	2339/0/141
Goodness-of-fit on F^2	1.056
Final R indexes [I>=2σ (I)]	R_1 = 0.0434, wR_2 = 0.1176
Final R indexes [all data]	R_1 = 0.0453, wR_2 = 0.1191
Largest diff. peak/hole / e Å^{-3}	0.35/-0.34

III.41 Crystal Structure of Dipp-7-Me$_2$

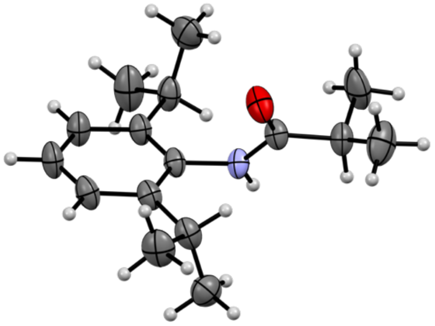

Figure SF217. Molecular structure of Dipp-7-Me$_2$. Thermal ellipsoids are shown with 50% probability.

Table ST42 Crystal data and structure refinement for Dipp-7-Me$_2$.

CCDC number	2423725
Empirical formula	$C_{16}H_{25}NO$
Formula weight	247.37
Temperature/K	150.00(10)
Crystal system	monoclinic
Space group	$P2_1/c$
a/Å	9.41061(18)
b/Å	18.6811(3)
c/Å	9.48545(16)
α/°	90
β/°	111.489(2)
γ/°	90
Volume/Å^3	1551.63(5)
Z	4
ρ_{calc}g/cm^3	1.059
μ/mm^{-1}	0.498
F(000)	544.0
Crystal size/mm^3	0.15 × 0.12 × 0.08
Radiation	Cu Kα (λ = 1.54184)
2Θ range for data collection/°	9.468 to 155.91
Index ranges	$-11 \le h \le 11$, $-22 \le k \le 23$, $-11 \le l \le 11$
Reflections collected	34108
Independent reflections	3213 [R_{int} = 0.0551, R_{sigma} = 0.0171]
Data/restraints/parameters	3213/0/169
Goodness-of-fit on F^2	1.069
Final R indexes [I>=2σ (I)]	R_1 = 0.0619, wR_2 = 0.1776
Final R indexes [all data]	R_1 = 0.0658, wR_2 = 0.1818
Largest diff. peak/hole / e Å^{-3}	0.64/-0.26

III.42 Crystal Structure of Dipp-7-sCy-d2

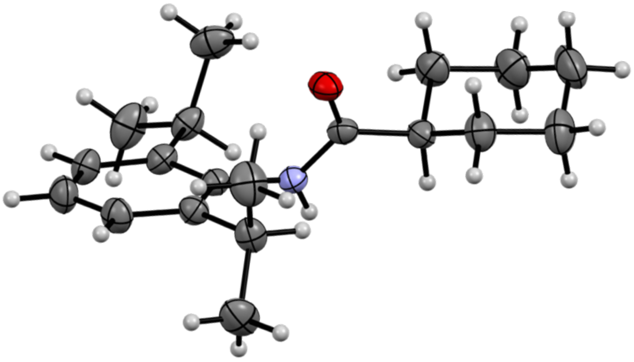

Figure SF218. Molecular structure of Dipp-7-sCy-d2. Thermal ellipsoids are shown with 50% probability.

Table ST43 Crystal data and structure refinement for Dipp-7-sCy-d2.

CCDC number	2423726
Empirical formula	$C_{19}H_{27}D_2NO$
Formula weight	289.44
Temperature/K	150.00(10)
Crystal system	monoclinic
Space group	$P2_1/c$
a/Å	18.1491(2)
b/Å	9.03480(10)
c/Å	23.1406(3)
α/°	90
β/°	111.7430(10)
γ/°	90
Volume/Å^3	3524.49(8)
Z	8
ρ_{calc}g/cm^3	1.091
μ/mm^{-1}	0.500
F(000)	1264.0
Crystal size/mm^3	0.2 × 0.15 × 0.12
Radiation	Cu Kα (λ = 1.54184)
2Θ range for data collection/°	5.242 to 155.656
Index ranges	-22 ≤ h ≤ 22, -11 ≤ k ≤ 10, -28 ≤ l ≤ 29
Reflections collected	74838
Independent reflections	7191 [R_{int} = 0.0323, R_{sigma} = 0.0144]
Data/restraints/parameters	7191/0/387
Goodness-of-fit on F^2	1.060
Final R indexes [I>=2σ (I)]	R_1 = 0.0469, wR_2 = 0.1293
Final R indexes [all data]	R_1 = 0.0520, wR_2 = 0.1334
Largest diff. peak/hole / e Å^{-3}	0.22/-0.18

III.43 Crystal Structure of Ph-11-Cl_2

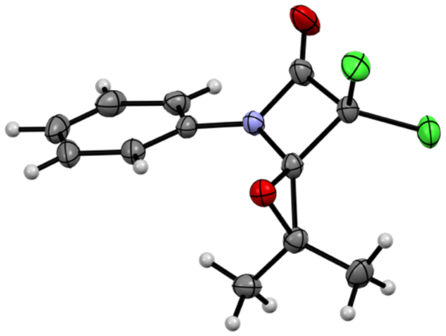

Figure SF219. Molecular structure of Ph-11-Cl_2. Thermal ellipsoids are shown with 50% probability.

Table ST44 Crystal data and structure refinement for Ph-11-Cl_2.

CCDC number	2423735
Empirical formula	$C_{12}H_{11}Cl_2NO_2$
Formula weight	272.12
Temperature/K	149.99(10)
Crystal system	triclinic
Space group	P-1
a/Å	8.37026(10)
b/Å	8.61910(7)
c/Å	9.82879(10)
α/°	101.0419(8)
β/°	106.7696(11)
γ/°	106.3027(9)
Volume/Å^3	622.322(12)
Z	2
ρ_{calc}g/cm^3	1.452
μ/mm^{-1}	4.611
F(000)	280.0
Crystal size/mm^3	0.2 × 0.08 × 0.08
Radiation	Cu Kα (λ = 1.54184)
2Θ range for data collection/°	9.842 to 155.39
Index ranges	-10 ≤ h ≤ 10, -10 ≤ k ≤ 10, -12 ≤ l ≤ 10
Reflections collected	31200
Independent reflections	2459 [R_{int} = 0.0406, R_{sigma} = 0.0147]
Data/restraints/parameters	2459/0/156
Goodness-of-fit on F^2	1.090
Final R indexes [I>=2σ (I)]	R_1 = 0.0290, wR_2 = 0.0775
Final R indexes [all data]	R_1 = 0.0299, wR_2 = 0.0781
Largest diff. peak/hole / e Å^{-3}	0.32/-0.28

III. Computational Details

1. Optimization of molecular and transition state structures

The DL-Find[8] optimization library, which is connected to various quantum chemistry program packages via the ChemShell[9] interface, is used for geometry and transition structure optimization. The Hessian matrices were calculated for all stationary points, to ensure that all minimum structures and transition states have zero or one negative eigenvalue, respectively. Furthermore, all transition state structures were confirmed to correspond to the correct elementary reactions by computing the intrinsic reaction coordinates. The rigid-rotor-harmonic oscillator approximation was used to calculate the free energies: frequencies below 100 cm^{-1} were set to this value to avoid divergence of the entropic term.

All calculations regarding the investigation of chemical reactivity were performed using the B3LYP[10-11] hybrid functional and the def2-SVP[12] basis set with D3[13] dispersion correction and COSMO ($\varepsilon_{Toluene}$ = 2.39)[14-15] in TeraChem version 1.9.[16]

The potential energies, zero-point-energy-corrected potential energies, and Gibbs free energies of the molecular species and transition state structures involved in the reactions studied in the main text are shown in **Tables ST48 and ST49** below.

The reactions investigated were computed with various substituents: Me_2, Cl_2, Ph_2, and sCy, which are collectively referred to as R^2. Reaction 1 describes the nucleophilic attack of the **Ph-6-R^2_2** on **Ph-5-R^2_2**. Subsequently, 2 involves the cleavage of acetone, leading to the formation of **[Ph-6-R^2_2*N]$_2$**. Reaction 3 describes the decomposition of the **Ph-6-R^2_2,** resulting in carbon monoxide release and formation of **Ph-10-R^2_2**. In addition, 4 is the dimerization of two **Ph-6-R^2_2** to **[Ph-6-R^2_2]$_2$**. Reaction 5 forms **Ph-11-R^2_2** starting from **Ph-6-R^2_2** and reacts with acetone. From these various reactions, the following network can be concluded in **Scheme SS1**.

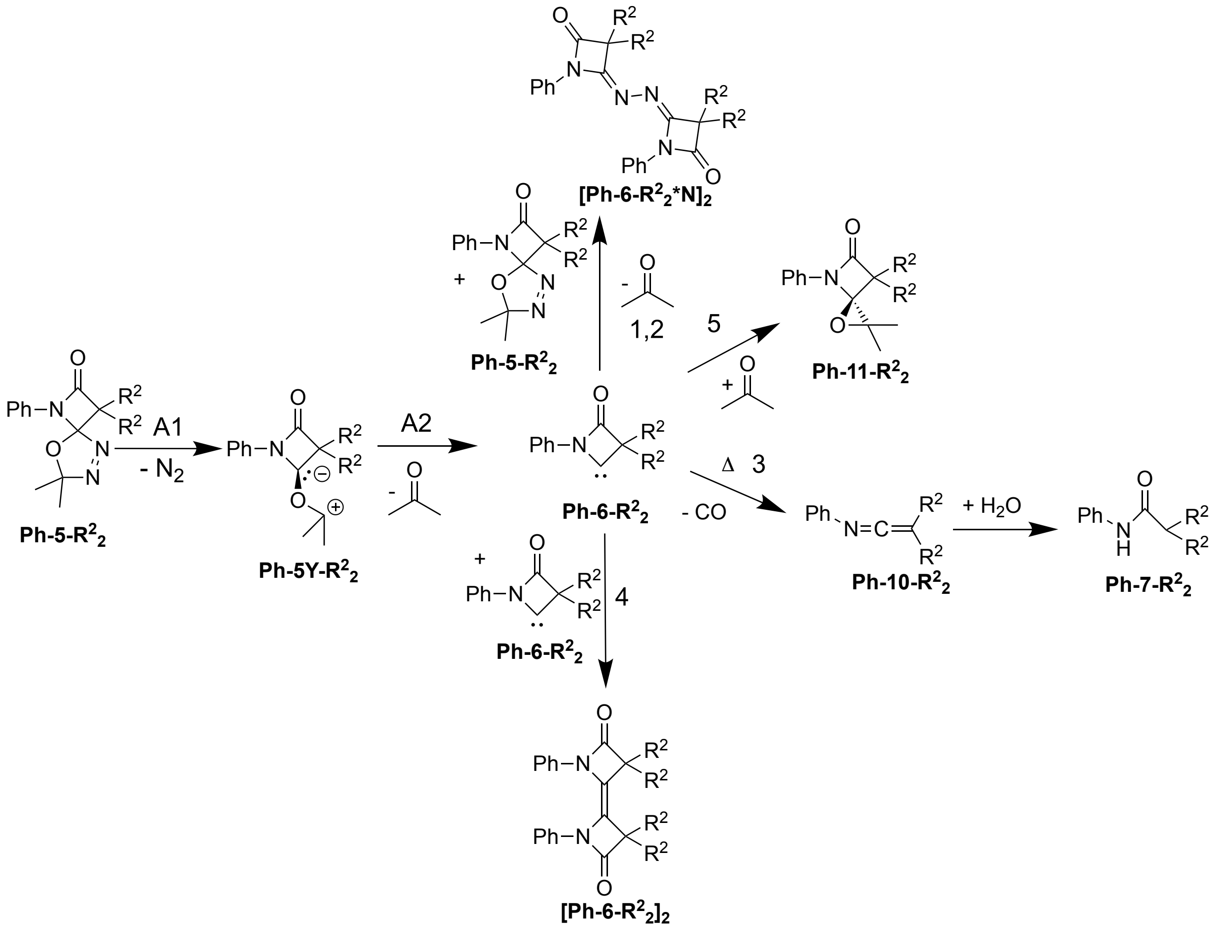

Scheme SS1 Obtained reaction network of all reactions involved in this study.

2. Calculation of HOMO, LUMO and singlet-triplet gaps

The calculations to determine minimum geometries and extracting HOMO, LUMO, singlet and triplet excitation energies of the carbene structures were performed using the B3LYP hybrid functional with D3 dispersion correction and Becke-Johnson[12] damping with the def2-TZVP[17] basis set in Turbomole version 7.2.1, which is interfaced with DL-Find via Chemshell. For the singlet-triplet gaps, Gibbs free energies evaluated at the minimum structures of the respective singlet or triplet potential energy surface are reported. For this, the rigid-rotor-harmonic oscillator approximation was used to calculate the free energies: frequencies below 100 cm^{-1} were set to this value to avoid divergence of the entropic term. The potential energies, zero-point-energy-corrected potential energies, and Gibbs free energies of the calculated species can be found in **Table ST45**, the resulting singlet-triplet gaps, ΔG_{ST}, can be found in **Table ST46.**

Table ST45 Energies of all species regarding the calculation of the physical properties with B3LYP+D3(BJ)/def2-TZVP in Hartree. *V* is the potential energy, *V*+ZPE is the zero-point energy corrected, vibrationally adiabatic potential energy and *G* (298K) is the free energy, i.e. including thermal contributions effects.

Species	*V*	*V+ZPE*	*G* (298K)
**MAC** (Singlet)	-1235.06635446	-1234.49342346	-1234.54653200
**MAC** (Triplet)	-1234.96159698	-1234.39034598	-1234.44386400
**CAAC** (Singlet)	-1148.56253173	-1147.88965873	-1147.94154400
**CAAC** (Triplet)	-1148.48474206	-1147.81389906	-1147.86624900
**CArAmc** (Singlet)	-904.55405322	-904.19540122	-904.23834400
**CArAmc** (Triplet)	-904.52191081	-904.16392181	-904.20684300
**Dipp-6-Cl$_2$** (Singlet)	-1631.93472258	-1631.64858258	-1631.69165300
**Dipp-6-Cl$_2$** (Triplet)	-1631.86748960	-1631.58242360	-1631.62548500
**Dipp-6-Ph$_2$** (Singlet)	-1174.83733533	-1174.37063133	-1174.42001100
**Dipp-6-Ph$_2$** (Triplet)	-1174.77042470	-1174.30514270	-1174.35463800
**Dipp-6-Spiro-Cy** (Singlet)	-908.13914343	-907.71165843	-907.75617900
**Dipp-6-Spiro-Cy** (Triplet)	-908.07270382	-907.64621282	-907.69073400
**Dipp-6-Me$_2$** (Singlet)	-791.42397602	-791.06321002	-791.10610300
**Dipp-6-Me$_2$** (Triplet)	-791.35791849	-790.99794749	-791.04054600
**Ph-6-Ph$_2$** (Singlet)	-939.00789002	-938.70989102	-938.75142800
**Ph-6-Ph$_2$** (Triplet)	-939.00789002	-938.70989102	-938.75142800
**Ph-6-Me$_2$** (Singlet)	-555.59847237	-555.40633137	-555.44058200
**Ph-6-Me$_2$** (Triplet)	-555.53552066	-555.34412966	-555.37819100

Table ST46 Energies of all species regarding the single triplet gap Δ*G* (298K) in kcal/mol is the free energy, i.e. including thermal contributions effects.

Species	ΔG_{ST} (298K)
**MAC**	64.42
**CAAC**	47.25
**CArAmc**	19.77
**Dipp-6-Cl$_2$**	41.52
**Dipp-6-Ph$_2$**	41.02
**Dipp-6-Spiro-Cy**	41.07
**Dipp-6-Me$_2$**	41.14
**Ph-6-Ph$_2$**	39.10
**Ph-6-Me$_2$**	39.15

3. *Ab initio* molecular dynamic and reaction discovery

To investigate the formation of **Ph-6-Me2**, we used high-temperature *ab initio* molecular dynamics (AIMD) simulations. All trajectories start from one **Ph-5-Me2** molecule and the velocity Verlet integration with a time step of 1 fs. A Langevin[18] thermostat with a friction term of γ = 0.41 ps^{-1} was used with various temperatures. AIMD runs with temperature set to 2000 K (5 runs of 1 ps each) and 3000 K (10 runs of 1 ps each) were performed with B3LYP[10-11]+D3/3-21G[13, 19] (Conductor-like Screening Model (COSMO);[14] $\varepsilon_{Toluene}$ = 2.39)[15, 19] and GFN2-xTB.[20] The equations of motion were integrated by an inhouse-developed python code which was interfaced to TeraChem[16] version 1.9 and to the xTB code version 6.4.1, which were used to compute electronic energies and gradients. To stabilize SCF convergence of the DFT computations the DIIS+a[21] algorithm implemented in TeraChem was used.

Table ST47 Results of the high-temperature *ab initio* molecular dynamics (AIMD) simulations.

	T	No. of AIMD runs	$A1_{Me}$	$A2_{Me}$	$B1_{Me}$	$B2_{Me}$	No. of side reactions	Total no. of reactions
B3LYP	2000 K	5	1	1	-	-	4	6
	3000 K	10	7	5	-	-	21	33
GFN2-xTB	2000 K	5	-	-	-	-	-	0
	3000 K	10	-	-	2	1	35	38

In the high-temperature AIMD simulations using B3LYP/3-21G, 39 reactions were observed, with 33 reactions occurring at 3000 K and 6 reactions at 2000 K temperature. Using the GFN2-xTB method, no reactions were found at 2000 K, but 38 reactions were found at 3000 K simulation temperature. Besides several reactions leading to decomposition or pyrolysis of **Ph-5-Me2**, two main formation pathways for **Ph-6-Me2** consisting of two elementary reactions each were found, shown in **Scheme SS2**. In pathway A, N_2 is released first (reaction $A1_{Me}$) and acetone is formed thereafter (reaction $A2_{Me}$). These two reactions were found in the AIMD runs using B3LYP+D3/3-21G in both temperature settings, see **Table ST47**. The calculations using the GFN2-xTB method reveal pathway B where acetone is released first (reaction $B1_{Me}$), then N_2 is formed (reaction $B2_{Me}$).

At first glance, both reaction pathways seem legit. In order to give a quantitative assessment of the pathways A and B, we optimized the stationary points and computed reaction free energies and the corresponding free energy barriers, which are described in the next section.

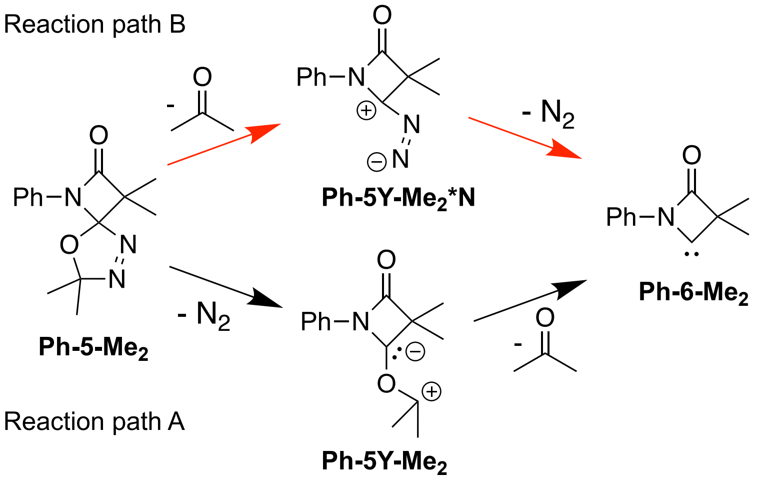

Scheme SS2 Comparison of both reaction pathways A (black, also shown in the main text) and B (red) to form **Ph-6-Me₂**.

Stationary points were computed and reported for all structures involved in pathways A and B. The results of pathway A are shown in in the main text. For pathway B, firstly releasing acetone (step $B1_{Me}$ in **Scheme SS3**) leads to a free energy barrier of 37.1 kcal/mol and a free reaction energy of 17.6 kcal/mol. Subsequently, the reaction proceeding from the intermediate **Ph-5Y-Me$_2$*N** (step $B2_{Me}$ in **Scheme SS3**) has a free reaction barrier of 10.4 kcal/mol and a free reaction energy of -16.7 kcal/mol. As this reaction pathway has a higher free energy barrier than pathway A, it appears less likely.

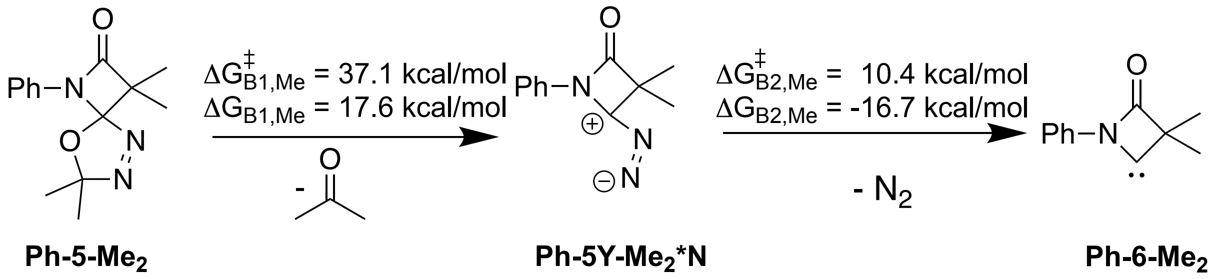

Scheme SS3 Alternative reaction pathway B to form **Ph-6-Me$_2$**.

Table ST48 gives the energies of the involved molecular species and the corresponding transition state structures (TS) and the reaction barriers and reaction heat are in **Table ST49**.

Table ST48 Energies of all *molecular* species and the transition state structures (TS) *involved in this study* in Hartree. *V* is the potential energy, *V+ZPE* is the (zero-point energy corrected) vibrationally adiabatic potential energy and *G* is the free energy, i.e. including thermal contributions effects at different temperatures. Molecular species of carbene formation reaction mechanisms A and B (see **Scheme SS2**), the nucleophilic attack of **Ph-6-R^2$_2$** on **Ph-5-R^2$_2$** (reaction 1), the formation of the N_2-bridged dimer (reaction 2), the carbene decomposition with corresponding intermediates **Ph-10-R^2$_2$** and product carbon monoxide (reaction 3), the dimer formation (reaction 4) and the epoxide formation (reaction 5) are shown.

Species	*V*	*V+ZPE*	*G* (193 K)	*G* (298 K)	*G* (383 K)
N_2	-109.442891	-109.437229	-109.446971	-109.453306	-109.458640
C_3H_6O (Acetone)	-193.033514	-192.950530	-192.965178	-192.975230	-192.984077
Oxadiazoles					
**Ph-5-Me$_2$**	-857.891060	-857.597449	-857.619217	-857.637246	-857.654745
**Ph-5-Cl$_2$**	-1698.237900	-1698.019104	-1698.041281	-1698.059408	-1698.076815
**Ph-5-Ph$_2$**	-1241.145852	-1240.746189	-1240.771116	-1240.792981	-1240.814710
**Ph-5-sCy**	-974.569943	-974.210304	-974.233168	-974.252416	-974.271260
First reaction step from oxadiazoles of carbene formation and ylidic intermediates **Ph-5Y-R^2$_2$**					
$TS_{A1,Me2}$	-857.831558	-857.545841	-857.569337	-857.589488	-857.608920
$TS_{A1,Cl2}$	-1698.192704	-1697.97s8651	-1698.001302	-1698.020121	-1698.038193
$TS_{A1,Ph2}$	-1241.098266	-1240.703592	-1240.729069	-1240.751751	-1240.774265
$TS_{A1,sCy}$	-974.518505	-974.164418	-974.188030	-974.208311	-974.228134
**Ph-5Y-Me$_2$**	-748.390980	-748.111680	-748.133446	-748.151381	-748.168650
**Ph-5Y-Cl$_2$**	-1588.750764	-1588.545825	-1588.567913	-1588.585839	-1588.602926
**Ph-5Y-Ph$_2$**	-1131.655187	-1131.269465	-1131.294287	-1131.315926	-1131.337313

**Ph-5Y-sCy**	-865.069879	-864.723720	-864.746271	-864.765092	-864.783420
Second reaction step from oxadiazoles of carbene formation and ylidic intermediates **Ph-5Y-R^2$_2$**					
$TS_{A2,Me2}$	-748.384106	-748.106537	-748.128353	-748.146304	-748.163560
$TS_{A2,Cl2}$	-1588.741381	-1588.538043	-1588.560248	-1588.578263	-1588.595398
$TS_{A2,Ph2}$	-1131.649070	-1131.265169	-1131.290186	-1131.311980	-1131.333469
$TS_{A2,sCy}$	-865.063047	-864.719229	-864.742022	-864.761052	-864.779535
Alternative pathway from oxadiazoles to carbenes					
$TS_{B1,Me2}$	-857.824557	-857.536100	-857.558525	-857.577385	-857.595653
**Ph-5Y-Me$_2$*N**	-664.794059	-664.591144	-664.611509	-664.627515	-664.642656
$TS_{B2,Me2}$	-664.774743	-664.574479	-664.594861	-664.610885	-664.626025
**Carbenes Ph-6-R^2$_2$**					
**Ph-6-Me$_2$**	-555.354906	-555.162581	-555.181938	-555.196737	-555.210611
**Ph-6-Cl$_2$**	-1395.704188	-1395.585756	-1395.605476	-1395.620193	-1395.633808
**Ph-6-Ph$_2$**	-938.614090	-938.315086	-938.337904	-938.356557	-938.374657
**Ph-6-sCy**	-672.033611	-671.774106	-671.794336	-671.810008	-671.824921
First reaction step to N_2-bridged dimer and corresponding intermediate					
$TS1_{Me2}$	-1413.256603	-1412.768544	-1412.795389	-1412.820371	-1412.845535
$TS1_{Cl2}$	-3093.958211	-3093.619362	-3093.646709	-3093.671670	-3093.696462
$TS1_{Ph2}$	-2179.780995	-2179.080786	-2179.113271	-2179.144827	-2179.178261
$TS1_{sCy}$	-1646.616668	-1645.995894	-1646.024325	-1646.051290	-1646.078765
**Ph-5-Me$_2$-Ph-6-Me$_2$**	-1413.324391	-1412.833779	-1412.860509	-1412.885445	-1412.910601

**Ph-5-Cl_2-Ph-6-Cl_2**	-3094.028815	-3093.686855	-3093.714039	-3093.738871	-3093.763581
**Ph-5-Ph_2-Ph-6-Ph_2**	-2179.846950	-2179.142453	-2179.174207	-2179.205923	-2179.238783
**Ph-5-sCy-Ph-6-sCy**	-1646.682299	-1646.057926	-1646.086062	-1646.112770	-1646.140043
Second reaction step to N_2-bridged dimer and corresponding product					
$TS2_{Me2}$	-1413.299611	-1412.811755	-1412.838756	-1412.863978	-1412.889374
$TS2_{Cl2}$	-3093.997399	-3093.659509	-3093.687186	-3093.712594	-3093.737816
$TS2_{Ph2}$	-2179.823773	-2179.122860	-2179.154788	-2179.186782	-2179.219902
$TS2_{sCy}$	-1646.657575	-1646.036506	-1646.064951	-1646.091994	-1646.119558
**[Ph-6-Me_2*N]$_2$**	-1220.302411	-1219.900766	-1219.926369	-1219.949294	-1219.972028
**[Ph-6-Cl_2*N]$_2$**	-2900.989779	-2900.737213	-2900.763392	-2900.786276	-2900.808615
**[Ph-6-Ph_2*N]$_2$**	-1986.831796	-1986.216855	-1986.247566	-1986.277312	-1986.307799
**[Ph-6-sCy*N]$_2$**	-1453.660653	-1453.125463	-1453.152535	-1453.177221	-1453.202063
Carbene decomposition with corresponding intermediates **Ph-10-R^2_2** and product carbon monoxide					
$TS3_{Me2}$	-555.318365	-555.129377	-555.148949	-555.164008	-555.178137
$TS3_{Cl2}$	-1395.663317	-1395.548676	-1395.568783	-1395.583945	-1395.597977
$TS3_{Ph2}$	-938.576322	-938.281079	-938.304254	-938.323310	-938.341784
$TS3_{sCy}$	-671.995899	-671.739949	-671.760418	-671.776399	-671.791612
**Ph-10-Me_2**	-442.138880	-441.957325	-441.976106	-441.990194	-442.003276
**Ph-10-Cl_2**	-1282.485902	-1282.377870	-1282.397044	-1282.411031	-1282.423839
**Ph-10-Ph_2**	-825.408660	-825.120099	-825.142399	-825.160279	-825.177534
**Ph-10-sCy**	-558.816009	-558.567637	-558.587426	-558.602450	-558.616625
CO	-113.229582	-113.224497	-113.234259	-113.240604	-113.245947

Transition state and product structures of carbene dimerization					
$TS4_{Me2}$	-1110.726624	-1110.340289	-1110.365511	-1110.387894	-1110.409972
$TS4_{Cl2}$	-2791.424878	-2791.187189	-2791.212732	-2791.234807	-2791.256262
$TS4_{Ph2}$	-1877.262068	-1876.649022	-1876.692951	-1876.721842	-1876.751404
$TS4_{sCy}$	-1344.088699	-1343.569216	-1343.595648	-1343.619574	-1343.643591
**[Ph-6-Me$_2$]$_2$**	-1110.887075	-1110.495979	-1110.520605	-1110.542388	-1110.563950
**[Ph-6-Cl$_2$]$_2$**	-2791.584137	-2791.342149	-2791.367419	-2791.389239	-2791.410475
**[Ph-6-Ph$_2$]$_2$**	-1877.415125	-1876.810210	-1876.840024	-1876.868656	-1876.897984
**[Ph-6-sCy]$_2$**	-1344.245145	-1343.720682	-1343.746869	-1343.770495	-1343.794237
Transition state and product structures of epoxide formation					
$TS5_{Me2}$	-748.388376	-748.109943	-748.131633	-748.149529	-748.166747
$TS5_{Cl2}$	-1588.733951	-1588.529928	-1588.551912	-1588.569744	-1588.586723
$TS5_{Ph2}$	-1131.649845	-1131.264572	-1131.289262	-1131.310742	-1131.331960
$TS5_{sCy}$	-865.066933	-864.722302	-864.744854	-864.763698	-864.782034
**Ph-11-Me$_2$**	-748.464141	-748.182330	-748.203683	-748.221216	-748.238119
**Ph-11-Cl$_2$**	-1588.814561	-1588.606985	-1588.628710	-1588.646262	-1588.662996
**Ph-11-Ph$_2$**	-1131.726036	-1131.337323	-1131.361748	-1131.382942	-1131.403916
**Ph-11-sCy**	-865.142631	-864.793985	-864.816202	-864.834674	-864.852677

Table ST49 Free energy barriers G_A and free reaction energies $\Delta_r G$ for all reactions in kcal/mol at -80 °C (193 K), 25 °C (298 K) and 110 °C (383 K). Reactions A1, A2: main carbene formation mechanism. Reactions B1, B2: alternative carbene formation mechanisms. Reactions 1 and 2: nucleophilic attach of **Ph-6-R$^2_2$** on **Ph-5-R$^2_2$** and formation of the N_2-bridged dimer. Reaction 3: carbene decomposition. Reaction 4: dimer formation. Reaction 5: epoxide formation.

Temperature	193 K		298 K		383 K	
Reaction	G_A	$\Delta_r G$	G_A	$\Delta_r G$	G_A	$\Delta_r G$
$A1_{Me2}$	31.30	24.35	29.97	20.43	28.76	17.23
$A1_{Cl2}$	25.09	16.57	24.65	12.72	24.24	9.57
$A1_{Ph2}$	26.39	18.74	25.87	14.90	25.38	11.77
$A1_{sCy}$	28.32	25.05	27.68	21.35	27.06	18.32
$A2_{Me2}$	3.20	-8.58	3.19	-12.92	3.19	-16.34
$A2_{Cl2}$	4.81	-1.72	4.75	-6.01	4.72	-9.39
$A2_{Ph2}$	2.57	-5.52	2.48	-9.95	2.41	-13.44
$A2_{sCy}$	2.67	-8.31	2.54	-12.64	2.44	-16.05
$B1_{Me2}$	38.08	26.69	37.56	21.58	37.08	17.58
$B2_{Me2}$	10.45	-10.92	10.44	-14.14	10.44	-16.69
1_{Me2}	3.62	-37.25	8.77	-32.07	12.44	-28.39
1_{Cl2}	0.03	-42.22	5.18	-36.99	8.89	-33.23
1_{Ph2}	-2.67	-40.90	4.49	-33.85	6.97	-31.01
1_{sCy}	2.00	-36.75	6.66	-31.92	10.93	-27.52
2_{Me2}	13.65	-19.48	13.47	-24.52	13.32	-28.55
2_{Cl2}	16.85	-9.12	16.49	-14.20	16.17	-18.27
2_{Ph2}	12.19	-24.18	12.01	-29.25	11.85	-33.32
2_{sCy}	13.25	-19.86	13.04	-24.90	12.85	-28.93
3_{Me2}	20.70	-17.84	20.53	-21.37	20.38	-24.23
3_{Cl2}	23.03	-16.21	22.75	-19.73	22.48	-22.58
3_{Ph2}	21.12	-24.32	20.86	-27.82	20.63	-30.64
3_{sCy}	21.28	-17.16	21.09	-20.74	20.90	-23.63
4_{Me2}	-1.03	-98.35	3.50	-93.44	7.06	-89.56
4_{Cl2}	-1.12	-98.18	3.50	-93.41	7.13	-89.64
4_{Ph2}	-10.76	-103.05	-5.48	-97.60	-1.31	-93.29
4_{sCy}	-4.38	-99.27	0.28	-94.43	3.92	-90.61
5_{Me2}	9.72	-35.50	14.08	-30.90	17.53	-27.25
5_{Cl2}	11.76	-36.43	16.11	-31.90	19.55	-28.31
5_{Ph2}	8.67	-36.81	13.21	-32.10	16.80	-28.35
5_{sCy}	9.20	-35.57	13.52	-31.02	16.92	-27.41

4. Cartesian coordinates

Compound **Ph-5-Me$_2$**

36

Potential Energy = -857.8910599 Hrt, B3LYP+D3/def2-SVP

O -2.773288 1.076807 -0.389640

C -2.386372 -0.065375 -0.410809

N -1.113110 -0.615585 -0.365623

C -3.051104 -1.451108 -0.501170

C -1.566938 -1.989885 -0.491016

N -1.221732 -2.874690 0.649384

O -1.101313 -2.653753 -1.621300

N -0.758045 -3.943285 0.242310

C -0.697911 -3.968964 -1.244040

C -1.696845 -5.008688 -1.747374

C 0.728413 -4.234024 -1.703939

C 0.176713 -0.055768 -0.337126

C -3.895843 -1.813729 0.718052

C -3.785664 -1.695295 -1.814759

C 1.281598 -0.828802 0.052436

C 2.558812 -0.262737 0.051998

H 1.146017 -1.860351 0.374223

C 2.744088 1.073091 -0.317584

H 3.413818 -0.873261 0.353763

C 1.635885 1.844474 -0.685598

H 3.744571 1.512355 -0.313080

C 0.354941 1.289728 -0.701834

H 1.767412 2.892087 -0.968841

H -0.512019 1.886844 -0.984233

H -1.407986 -6.012971 -1.405380

H -1.711435 -4.994981 -2.847076

H -2.707199 -4.783118 -1.374555

H 1.403882 -3.452290 -1.329092
H 0.762093 -4.230168 -2.803384
H 1.068036 -5.213973 -1.339560
H -3.156623 -1.459608 -2.684771
H -4.686691 -1.063487 -1.851548
H -4.100430 -2.748042 -1.890229
H -4.168076 -2.880949 0.691133
H -4.823123 -1.220037 0.712605
H -3.360824 -1.619993 1.658368

Compound $TS_{B1,Me2}$

36
Potential Energy = -857.824557 Hrt, B3LYP+D3/def2-SVP
O -1.900093 1.756535 0.171754
C -1.992112 0.562686 0.136668
N -1.015029 -0.450388 0.031299
C -3.126497 -0.497611 0.151329
C -1.946210 -1.482769 -0.015139
N -1.830450 -2.685584 0.572243
O -1.931632 -2.473201 -1.961127
N -1.865028 -3.828141 0.275721
C -2.045721 -3.717879 -1.770590
C -3.428798 -4.347843 -1.861618
C -0.873290 -4.632206 -2.094905
C 0.389795 -0.425695 -0.128085
C -3.860410 -0.599365 1.487696
C -4.074103 -0.415401 -1.042201
H -3.158492 -0.636623 2.334609
H -4.482521 -1.507798 1.508166
H -4.518444 0.273954 1.614147
H -3.477248 -5.322555 -1.353861
H -3.679851 -4.502784 -2.926288

H -4.185388 -3.673303 -1.433630
H -0.980257 -5.619880 -1.622703
H 0.075234 -4.181157 -1.770083
H -0.825427 -4.777097 -3.188985
H -3.523734 -0.425686 -1.991687
H -4.670103 0.507766 -0.969529
H -4.759664 -1.276454 -1.034980
C 1.007938 -1.355128 -0.974198
C 2.396920 -1.329472 -1.114722
H 0.382605 -2.051468 -1.533749
C 3.159824 -0.373493 -0.434222
H 2.884022 -2.053399 -1.773011
C 2.527712 0.564845 0.389745
H 4.246154 -0.354440 -0.551679
C 1.140667 0.541872 0.553202
H 3.118570 1.318206 0.916810
H 0.638508 1.266848 1.195512

Compound **Ph-5Y-Me$_2$*N**

26
Potential Energy = -664.7940592 Hrt, B3LYP+D3/def2-SVP
O -1.535009 0.693657 1.358668
C -1.937079 -0.115035 0.559863
N -1.317505 -1.191932 -0.074544
C -3.276820 -0.357284 -0.192750
C -2.479798 -1.494923 -0.862299
N -2.872048 -2.662782 -1.249839
N -3.228059 -3.640921 -1.742311
C -0.004272 -1.679238 -0.090198
C -4.416124 -0.811812 0.718318
C -3.673956 0.798524 -1.108793
H -4.097085 -1.630453 1.381650

H -5.270807 -1.161432 0.117074
H -4.755447 0.030589 1.340828
H -2.842294 1.093262 -1.766467
H -3.966110 1.670237 -0.501935
H -4.531251 0.508987 -1.736465
C 0.380697 -2.638219 -1.041595
C 1.691721 -3.120659 -1.044343
H -0.334589 -3.000990 -1.781096
C 2.623604 -2.654626 -0.111881
H 1.984192 -3.867482 -1.787001
C 2.233418 -1.693590 0.829035
H 3.647432 -3.035983 -0.118180
C 0.928015 -1.202221 0.850210
H 2.954720 -1.320159 1.560690
H 0.619684 -0.450695 1.576761

Compound C_3H_6O

10
Potential Energy = -193.033513750600 Hrt, B3LYP+D3/def2-SVP
O -2.160361 -2.816905 0.015762
C -1.627980 -3.768753 0.550080
C -0.132030 -3.817426 0.791029
C -2.422074 -4.973929 1.013950
H 0.308065 -4.670450 0.247096
H 0.078145 -3.979265 1.861489
H 0.334915 -2.882255 0.453898
H -2.022862 -5.894680 0.556809
H -3.481526 -4.852278 0.751012
H -2.320972 -5.090469 2.106406

Compound $TS_{B2,Me2}$

26

Potential Energy = -664.7747427 Hrt, B3LYP+D3/def2-SVP

O -1.492464 0.799907 1.263145

C -1.919034 -0.025735 0.500723

N -1.306956 -1.110935 -0.158902

C -3.233816 -0.305998 -0.255119

C -2.403795 -1.424080 -0.970612

N -3.023109 -2.961107 -0.544389

N -3.237436 -3.988461 -0.932786

C 0.002234 -1.633746 -0.151049

C -4.389075 -0.746113 0.642992

C -3.643216 0.820405 -1.205834

H -4.068004 -1.494825 1.382863

H -5.201877 -1.179869 0.038845

H -4.790953 0.125332 1.183344

H -2.815085 1.107366 -1.871959

H -3.949385 1.707505 -0.629193

H -4.491492 0.496459 -1.828983

C 0.341760 -2.656286 -1.049780

C 1.633323 -3.185990 -1.031154

H -0.402154 -3.020641 -1.760258

C 2.585686 -2.702337 -0.127000

H 1.897086 -3.982393 -1.731830

C 2.238360 -1.679060 0.762302

H 3.595201 -3.120447 -0.116494

C 0.950229 -1.139631 0.759127

H 2.977525 -1.294374 1.469712

H 0.671689 -0.340106 1.445690

Compound **Ph-6-Me$_2$**

24

Potential Energy = -555.354906327700 Hrt, B3LYP+D3/def2-SVP

H -2.035069 -0.052360 7.508880

H -2.556734 1.651445 9.257860

C -1.373320 0.805479 7.653273

C -1.665865 1.761604 8.634509

C -0.237977 0.939358 6.854295

H 0.010025 0.207160 6.084293

C -0.815058 2.856387 8.813594

H -1.038289 3.605638 9.577316

C 0.607126 2.040750 7.043335

C 0.326950 3.004702 8.020388

N 1.764785 2.176097 6.228648

H 0.995623 3.855316 8.153457

C 2.239200 1.397897 5.231057

C 2.813084 3.170094 6.181607

C 3.446899 2.360434 5.036423

O 3.000174 4.164675 6.815301

H 4.776647 1.222484 6.351530

C 4.797003 1.727806 5.373277

H 2.475717 3.521876 3.455405

C 3.454656 3.072049 3.683569

H 5.056209 0.981575 4.605654

H 3.696342 2.349984 2.887675

H 5.586012 2.496391 5.394197

H 4.215220 3.869020 3.674926

Compound N_2

2

Potential Energy = -109.442891404600 Hrt, B3LYP+D3/def2-SVP

N -1.724039 -2.903293 -2.078672

N -1.408341 -3.908457 -1.762068

Compound $TS_{A1,Me2}$

36

Potential Energy = -857.8315583 Hrt, B3LYP+D3/def2-SVP

O -1.691417 1.642534 0.856695

C -1.915421 0.499344 0.545907

N -1.080958 -0.549573 0.151382

C -3.193773 -0.378413 0.411993

C -2.177334 -1.429728 -0.050292

N -1.421704 -2.921823 2.590672

O -2.047886 -2.686615 -0.263272

N -1.816444 -3.939149 2.425142

C -2.878727 -3.732633 -0.352718

C -4.331577 -3.446302 -0.479201

C -2.230026 -4.928936 -0.968395

C 0.306669 -0.655350 -0.001972

C -3.867190 -0.594004 1.772544

C -4.170310 0.161690 -0.635686

H -4.887195 -4.384288 -0.621603

H -4.544329 -2.791232 -1.355873

H -4.755923 -2.940569 0.404560

H -2.753943 -5.849591 -0.665550

H -1.181093 -5.017037 -0.643102

H -2.232046 -4.907189 -2.081994

C 0.888913 -1.863664 -0.427279

C 2.276278 -1.947085 -0.568286

H 0.261955 -2.726692 -0.643455

C 3.091553 -0.845479 -0.291987

H 2.721636 -2.889355 -0.898554

C 2.504096 0.354497 0.129304

H 4.175956 -0.919216 -0.403985

C 1.121758 0.460041 0.276418

H 3.130432 1.223694 0.347053

H 0.660601 1.391636 0.602820

H -4.715486 -1.288593 1.686330
H -4.251143 0.370925 2.140254
H -3.160569 -0.993067 2.513786
H -3.676106 0.299599 -1.609220
H -4.562936 1.135691 -0.302067
H -5.019381 -0.525103 -0.768088

Compound **Ph-5Y-Me$_2$**

34
Potential Energy = -748.3909804 Hrt, B3LYP+D3/def2-SVP
O -2.371647 1.144738 0.930380
C -2.305556 0.097249 0.325835
N -1.258132 -0.572888 -0.280142
C -3.299125 -1.042199 -0.004029
C -2.024478 -1.781462 -0.534882
O -1.960487 -2.132081 -1.875264
C -2.580786 -3.122470 -2.422704
C -3.431852 -3.973386 -1.570715
C -2.176298 -3.466423 -3.812301
C 0.117214 -0.322053 -0.341888
C -3.970534 -1.636784 1.229481
C -4.307030 -0.597814 -1.068502
H -3.221662 -1.944851 1.974410
H -4.573000 -2.519375 0.962280
H -4.638084 -0.892587 1.693654
H -3.611904 -4.955350 -2.030507
H -4.402359 -3.525129 -1.306805
H -2.881266 -4.056875 -0.600184
H -1.769932 -4.494692 -3.855332
H -1.419691 -2.759335 -4.179055
H -3.045177 -3.444154 -4.493451
H -3.804511 -0.173948 -1.951352

H -4.966345 0.177191 -0.646619
H -4.943742 -1.431749 -1.402763
C 0.984175 -1.305331 -0.850890
C 2.357168 -1.058728 -0.907953
H 0.575672 -2.255171 -1.197047
C 2.881302 0.161791 -0.468155
H 3.023563 -1.830495 -1.303032
C 2.013396 1.137949 0.036240
H 3.956228 0.351693 -0.519329
C 0.638362 0.907808 0.103734
H 2.411034 2.094906 0.385234
H -0.040608 1.661176 0.502863

Compound $TS_{A2,Me2}$

34
Potential Energy =-748.3841059 Hrt, B3LYP+D3/def2-SVP
O -1.681291 1.139431 1.102176
C -1.827617 0.077568 0.542667
N -0.919229 -0.841678 0.022954
C -2.992259 -0.886118 0.258860
C -1.857416 -1.891586 -0.181439
O -1.927863 -1.945933 -1.943872
C -2.456621 -2.915007 -2.516590
C -3.316347 -3.869025 -1.763535
C -2.160965 -3.139767 -3.965244
C 0.484830 -0.857658 -0.035250
C -3.720163 -1.342665 1.523160
C -3.965887 -0.363388 -0.795719
H -3.013566 -1.739625 2.268183
H -4.442304 -2.138624 1.278882
H -4.268305 -0.501407 1.977829
H -2.809005 -4.041701 -0.794308

H -3.505655 -4.794344 -2.323232
H -4.277101 -3.391414 -1.512735
H -1.681188 -4.126998 -4.087850
H -1.507667 -2.350883 -4.359506
H -3.104760 -3.175183 -4.534925
H -3.443747 0.026198 -1.681390
H -4.569701 0.452860 -0.367227
H -4.660993 -1.154871 -1.120113
C 1.147776 -2.014882 -0.474481
C 2.542298 -2.032695 -0.535988
H 0.558942 -2.889184 -0.754643
C 3.285398 -0.905335 -0.166661
H 3.053268 -2.937539 -0.876517
C 2.618163 0.244559 0.270323
H 4.376887 -0.923003 -0.219432
C 1.223499 0.278017 0.339412
H 3.188737 1.129914 0.563832
H 0.696726 1.167043 0.686056

Compound $TS_{1,Me2}$

60
Potential Energy = -1413.256602541400 Hrt, B3LYP+D3/def2-SVP
C -2.540417 -1.766286 7.518140
C -1.571152 -0.773730 7.344924
C -3.410194 -2.066235 6.463786
C -1.468936 -0.083342 6.135090
C -3.323607 -1.383287 5.249318
C -2.348265 -0.384225 5.083312
H -2.615887 -2.304247 8.466130
H -0.880083 -0.531360 8.156402
H -4.170069 -2.842810 6.584520
H -3.995313 -1.616651 4.423733

H -0.700043 0.676474 6.015097
N -2.262632 0.300986 3.858772
C -1.487502 1.453898 3.447866
C -2.895235 0.140832 2.632540
O -3.715915 -0.653564 2.245631
C -2.130621 1.320671 2.008447
C -1.183153 0.892887 0.890678
C -3.012605 2.498679 1.610233
H -2.393414 3.356031 1.304384
H -3.666747 2.818760 2.433505
H -3.640336 2.205566 0.754733
O -1.737539 2.610074 4.188163
N -0.020120 1.264613 3.524909
C -0.503937 3.295929 4.402822
H -1.774072 0.579961 0.016209
H -0.540964 0.055485 1.195558
H -0.534419 1.727037 0.588318
N 0.499651 2.245116 4.062128
C -0.364770 4.466457 3.434194
C -0.365878 3.703480 5.859659
H 0.593513 4.974714 3.595864
H -1.199569 5.162885 3.602772
H -0.398927 4.110272 2.394725
H 0.619934 4.161402 6.022945
H -0.479027 2.834275 6.521838
H -1.145681 4.438700 6.106062
H 1.223598 3.849852 -1.168716
H 1.424543 1.729441 -2.473487
C 1.594476 2.941815 -0.686086
C 1.708293 1.753938 -1.418541
C 1.952444 2.976950 0.662383
H 1.873764 3.892866 1.249279
C 2.187298 0.598683 -0.791775

H 2.278380 -0.332751 -1.356350

C 2.429659 1.812901 1.282687

C 2.551486 0.619357 0.556998

N 2.776490 1.850154 2.649764

H 2.928291 -0.276514 1.051007

C 2.488319 2.803802 3.612172

C 3.559702 1.003748 3.468868

C 3.339185 1.976183 4.644189

O 4.168097 -0.005438 3.236831

H 5.098869 3.190339 4.162880

C 4.612803 2.731242 5.037950

H 1.824692 0.701905 5.576133

C 2.642745 1.376555 5.862542

H 4.365367 3.533307 5.751645

H 2.228484 2.176978 6.495360

H 5.332082 2.046288 5.514693

H 3.367657 0.804102 6.463383

Compound **Ph-5-Me$_2$-Ph-6-Me$_2$**

60

Potential Energy = -1413.324390584000 Hrt, B3LYP+D3/def2-SVP

C -0.490438 -3.730228 4.969028

C 0.297901 -2.579286 4.864607

C -1.825897 -3.685199 4.552869

C -0.236307 -1.393904 4.355610

C -2.378373 -2.506383 4.048202

C -1.583254 -1.349636 3.952995

H -0.066286 -4.655918 5.365689

H 1.346454 -2.603082 5.174087

H -2.451128 -4.579458 4.623091

H -3.416782 -2.465593 3.719534

H 0.391244 -0.512599 4.241175

N -2.132270 -0.160104 3.459631
C -1.632541 1.232098 3.487850
C -3.331722 0.153507 2.853255
O -4.291612 -0.522983 2.559508
C -2.963382 1.640559 2.726184
C -2.738264 2.088379 1.285315
C -3.898714 2.575055 3.484377
H -3.513515 3.607432 3.467229
H -4.016852 2.269748 4.533651
H -4.890861 2.573707 3.005855
O -1.550215 1.687110 4.833938
N -0.381689 1.464377 2.873552
C -0.379296 2.431894 5.029267
H -3.698970 2.120129 0.747609
H -2.054121 1.413596 0.751704
H -2.295489 3.097023 1.267878
N 0.414759 2.016501 3.793642
C -0.659621 3.930694 4.934697
C 0.248373 2.013323 6.344626
H 0.260676 4.517438 5.060137
H -1.369814 4.210500 5.726633
H -1.109593 4.165557 3.959343
H 1.100110 2.650519 6.608387
H 0.557932 0.960568 6.312574
H -0.516624 2.128206 7.125969
H -0.241168 3.008435 -1.269724
H 1.291546 1.741746 -2.780607
C 0.653484 2.527251 -0.866386
C 1.512496 1.817377 -1.713032
C 0.931502 2.634036 0.497054
H 0.266707 3.184037 1.160385
C 2.657486 1.208229 -1.187587
H 3.334716 0.654493 -1.842714

C 2.069438 2.001781 1.018970
C 2.938796 1.292261 0.177775
N 2.394847 2.119730 2.389692
H 3.826039 0.815461 0.596145
C 1.667874 2.308019 3.582306
C 3.622153 2.402156 3.003024
C 2.943223 2.626920 4.383458
O 4.745356 2.446675 2.580382
H 2.782413 4.793330 4.130550
C 3.114471 4.062242 4.883204
H 3.167047 0.561913 5.074570
C 3.420261 1.582487 5.399414
H 2.542232 4.225803 5.808714
H 2.993539 1.751336 6.396428
H 4.177499 4.247174 5.102988
H 4.516068 1.656057 5.483880

Compound $TS_{2,Me2}$

60
Potential Energy = -1413.299546591800 Hrt, B3LYP+D3/def2-SVP
C -0.490438 -3.730228 4.969028
C 0.297901 -2.579286 4.864607
C -1.825897 -3.685199 4.552869
C -0.236307 -1.393904 4.355610
C -2.378373 -2.506383 4.048202
C -1.583254 -1.349636 3.952995
H -0.066286 -4.655918 5.365689
H 1.346454 -2.603082 5.174087
H -2.451128 -4.579458 4.623091
H -3.416782 -2.465593 3.719534
H 0.391244 -0.512599 4.241175
N -2.132270 -0.160104 3.459631

C -1.632541 1.232098 3.487850
C -3.331722 0.153507 2.853255
O -4.291612 -0.522983 2.559508
C -2.963382 1.640559 2.726184
C -2.738264 2.088379 1.285315
C -3.898714 2.575055 3.484377
H -3.513515 3.607432 3.467229
H -4.016852 2.269748 4.533651
H -4.890861 2.573707 3.005855
O -1.550215 1.687110 4.833938
N -0.381689 1.464377 2.873552
C -0.379296 2.431894 5.029267
H -3.698970 2.120129 0.747609
H -2.054121 1.413596 0.751704
H -2.295489 3.097023 1.267878
N 0.414759 2.016501 3.793642
C -0.659621 3.930694 4.934697
C 0.248373 2.013323 6.344626
H 0.260676 4.517438 5.060137
H -1.369814 4.210500 5.726633
H -1.109593 4.165557 3.959343
H 1.100110 2.650519 6.608387
H 0.557932 0.960568 6.312574
H -0.516624 2.128206 7.125969
H -0.241168 3.008435 -1.269724
H 1.291546 1.741746 -2.780607
C 0.653484 2.527251 -0.866386
C 1.512496 1.817377 -1.713032
C 0.931502 2.634036 0.497054
H 0.266707 3.184037 1.160385
C 2.657486 1.208229 -1.187587
H 3.334716 0.654493 -1.842714
C 2.069438 2.001781 1.018970

C 2.938796 1.292261 0.177775

N 2.394847 2.119730 2.389692

H 3.826039 0.815461 0.596145

C 1.667874 2.308019 3.582306

C 3.622153 2.402156 3.003024

C 2.943223 2.626920 4.383458

O 4.745356 2.446675 2.580382

H 2.782413 4.793330 4.130550

C 3.114471 4.062242 4.883204

H 3.167047 0.561913 5.074570

C 3.420261 1.582487 5.399414

H 2.542232 4.225803 5.808714

H 2.993539 1.751336 6.396428

H 4.177499 4.247174 5.102988

H 4.516068 1.656057 5.483880

Compound **[Ph-6-Me$_2$*N]$_2$**

50

Potential Energy = -1220.302411379800 Hrt, B3LYP+D3/def2-SVP

C -2.756051 -1.189767 5.193679

C -2.188507 -0.135816 5.917179

C -2.966683 -1.047495 3.816875

C -1.831612 1.054503 5.279887

C -2.614907 0.133269 3.160613

C -2.045349 1.185297 3.898543

H -3.033448 -2.118358 5.698723

H -2.016392 -0.232262 6.992163

H -3.409665 -1.865538 3.242817

H -2.777800 0.252238 2.089622

H -1.387551 1.878719 5.835433

N -1.692343 2.380431 3.242702

C -1.077019 3.566556 3.665283

C -1.857015 2.844404 1.919952
O -2.341246 2.330879 0.947369
C -1.192680 4.199741 2.273985
C 0.137909 4.448994 1.564644
C -2.142818 5.395188 2.193149
H -1.674363 6.271266 2.664892
H -3.093342 5.188173 2.709103
H -2.361672 5.629048 1.139441
N -0.635607 3.871597 4.820008
H -0.040933 4.668776 0.500392
H 0.801734 3.572857 1.632150
H 0.647720 5.307435 2.025838
N -0.100937 5.162088 4.856250
H -1.323140 2.422959 10.676006
H -0.404423 0.329019 9.674254
C -0.759615 2.463111 9.740471
C -0.244843 1.290158 9.179128
C -0.563844 3.692786 9.105965
H -0.964053 4.612866 9.534926
C 0.478986 1.351256 7.982271
H 0.882590 0.438106 7.538261
C 0.154626 3.744617 7.904321
C 0.688002 2.575318 7.345561
N 0.380743 5.005616 7.297625
H 1.245773 2.630625 6.410562
C 0.310523 5.545826 5.995760
C 0.868952 6.207736 7.852889
C 0.866734 6.892016 6.462546
O 1.152763 6.501216 8.981331
H 2.946592 6.361579 6.042457
C 2.264778 7.219962 5.937687
H -1.102181 7.803465 6.720044
C -0.104468 8.064097 6.334239

H 2.210327 7.492002 4.872106

H -0.206153 8.352601 5.276462

H 2.686434 8.070594 6.495448

H 0.273525 8.930766 6.898577

Compound **Ph-5-Cl$_2$**

30

Potential Energy = -1698.237899760600 Hrt, B3LYP+D3/def2-SVP

O -2.749821 1.069997 -0.372623

C -2.321250 -0.043869 -0.490517

N -1.058584 -0.597112 -0.375992

C -2.929669 -1.428025 -0.816618

C -1.447436 -1.968136 -0.636096

N -1.274771 -2.907140 0.513294

O -0.841526 -2.578499 -1.705577

N -0.857917 -3.989913 0.096948

C -0.680669 -3.966505 -1.379588

C -1.793574 -4.804815 -2.005910

C 0.716511 -4.426113 -1.759357

C 0.196915 -0.037374 -0.068283

Cl -4.119630 -2.012224 0.360204

Cl -3.531601 -1.570512 -2.477157

C 1.322126 -0.863085 0.069969

C 2.560662 -0.295085 0.374120

H 1.236064 -1.940275 -0.054993

C 2.686585 1.087620 0.540682

H 3.433360 -0.944036 0.481792

C 1.558568 1.903675 0.402429

H 3.658469 1.526719 0.778012

C 0.312548 1.353029 0.097828

H 1.644842 2.985373 0.532928

H -0.569167 1.984138 -0.008389

H -1.696144 -5.855176 -1.696493
H -1.719068 -4.741800 -3.101130
H -2.779292 -4.429761 -1.695158
H 1.480868 -3.822209 -1.249494
H 0.849304 -4.319925 -2.845933
H 0.852444 -5.481644 -1.484999

Compound $TS_{A1,Cl2}$

30
Potential Energy = -1698.192703957300 Hrt, B3LYP+D3/def2-SVP
O -1.654231 1.624624 1.058055
C -1.836228 0.479167 0.755621
N -1.013820 -0.538069 0.292331
C -3.050994 -0.503809 0.761349
C -2.017743 -1.554714 0.365657
N -1.712074 -3.214147 1.825178
O -2.100758 -2.428871 -0.635501
N -2.025782 -4.149091 1.268067
C -2.835309 -3.532577 -0.669541
C -4.257196 -3.515817 -0.209795
C -2.381711 -4.527378 -1.682598
C 0.372799 -0.614840 0.083250
Cl -3.847271 -0.692436 2.339524
Cl -4.243880 -0.106685 -0.518090
H -4.580707 -4.548104 -0.010730
H -4.913092 -3.095243 -0.992495
H -4.397390 -2.922214 0.704156
H -2.551826 -5.545511 -1.298099
H -1.312987 -4.398118 -1.903088
H -2.953310 -4.430673 -2.624395
C 0.974155 -1.859073 -0.167885
C 2.354644 -1.925726 -0.365339

H 0.368899 -2.764065 -0.202410
C 3.137582 -0.767382 -0.320958
H 2.820201 -2.895980 -0.556337
C 2.528344 0.468073 -0.072323
H 4.217000 -0.826410 -0.479617
C 1.150419 0.555557 0.129735
H 3.131546 1.378719 -0.033191
H 0.671816 1.513765 0.330792

Compound **Ph-5Y-Cl₂**

28
Potential Energy = -1588.750764299500 Hrt, B3LYP+D3/def2-SVP
O -2.066011 0.852213 1.413296
C -2.103520 0.059350 0.508373
N -1.164105 -0.488148 -0.334925
C -3.210224 -0.823881 -0.142845
C -2.061220 -1.486018 -0.923721
O -2.097929 -1.358679 -2.313831
C -2.894092 -2.015929 -3.076457
C -3.842733 -2.970174 -2.473199
C -2.673623 -1.872854 -4.537344
C 0.234705 -0.409862 -0.349707
Cl -4.128038 -1.842702 0.996445
Cl -4.346271 0.185273 -1.117828
H -4.215658 -3.690710 -3.213338
H -4.697344 -2.470026 -1.990701
H -3.291303 -3.464869 -1.640743
H -2.466481 -2.860610 -4.987633
H -1.841830 -1.185932 -4.741940
H -3.588970 -1.491135 -5.022285
C 0.966890 -1.264897 -1.190669
C 2.361366 -1.196526 -1.200865

H 0.440119 -1.976070 -1.827521
C 3.035006 -0.279784 -0.386651
H 2.925738 -1.866463 -1.855020
C 2.298410 0.571492 0.445993
H 4.126330 -0.227062 -0.401932
C 0.904261 0.515338 0.472322
H 2.815041 1.290434 1.087366
H 0.326453 1.170329 1.124151

Compound $TS_{A2,Cl2}$

28
Potential Energy = -1588.741380844700 Hrt, B3LYP+D3/def2-SVP
O -1.485869 1.174062 0.872734
C -1.306660 0.191805 0.206928
N -0.241994 -0.688971 0.046131
C -2.144837 -0.705494 -0.729514
C -0.875869 -1.636343 -0.798153
O -0.110102 -1.175089 -2.399705
C -0.303067 -1.887371 -3.395170
C -1.372688 -2.927672 -3.411558
C 0.568109 -1.693692 -4.595541
C 1.030098 -0.746283 0.648689
Cl -3.498002 -1.516239 0.110356
Cl -2.734724 0.113086 -2.201025
H -1.365271 -3.437005 -2.432927
H -1.265216 -3.626145 -4.251550
H -2.348394 -2.418050 -3.479439
H 1.099320 -2.636953 -4.811544
H 1.286281 -0.879640 -4.435018
H -0.065635 -1.479831 -5.472693
C 1.879539 -1.822787 0.351679
C 3.140907 -1.886213 0.945126

H 1.536158 -2.597435 -0.334748
C 3.562358 -0.883396 1.826195
H 3.801069 -2.726679 0.715237
C 2.708820 0.187259 2.114261
H 4.552639 -0.935858 2.285360
C 1.441783 0.265008 1.531415
H 3.029913 0.974115 2.801646
H 0.770227 1.094226 1.753902

Compound **Ph-6-Cl₂**

18
Potential Energy = -1395.704188248700 Hrt, B3LYP+D3/def2-SVP
H -1.993135 -0.127147 7.437792
H -2.573172 1.531058 9.210862
C -1.352937 0.742222 7.605956
C -1.677718 1.673531 8.601018
C -0.212394 0.916511 6.823500
H 0.059467 0.203911 6.043492
C -0.856090 2.784686 8.813043
H -1.106519 3.513105 9.587939
C 0.602006 2.034780 7.047174
C 0.291251 2.975195 8.037835
N 1.764935 2.211066 6.248366
H 0.936386 3.839125 8.197497
C 2.261995 1.447873 5.249514
C 2.774182 3.243893 6.244951
C 3.431746 2.455419 5.092012
O 2.941542 4.227900 6.886780
Cl 5.018356 1.767147 5.490639
Cl 3.423311 3.304264 3.532713

Compound **Ph-5-Ph₂**

50

Potential Energy = -1241.145851518000 Hrt, B3LYP+D3/def2-SVP

O -1.857482 3.198868 -2.375456

C -2.286259 2.359560 -1.620218

N -1.693349 1.253247 -1.057863

C -3.634250 2.084368 -0.906681

C -2.807867 0.932390 -0.178676

N -2.479202 1.244436 1.240619

O -3.238872 -0.380563 -0.175791

N -2.697880 0.267454 1.958621

C -3.231034 -0.870707 1.173326

C -4.649615 -1.179181 1.642680

C -2.291143 -2.062258 1.308633

C -0.366622 0.766057 -1.094166

C -0.136829 -0.611138 -1.220142

C 1.174966 -1.091855 -1.241809

H -0.986391 -1.288252 -1.313396

C 2.252842 -0.202954 -1.167273

H 1.354803 -2.166120 -1.333247

C 2.015649 1.172926 -1.067310

H 3.277767 -0.581305 -1.194205

C 0.708686 1.662628 -1.022045

H 2.854681 1.871375 -1.014733

H 0.512354 2.732392 -0.936648

H -4.632035 -1.559670 2.673614

H -5.092104 -1.942696 0.986805

H -5.275081 -0.275598 1.615932

H -1.266098 -1.780900 1.031328

H -2.632776 -2.873207 0.648559

H -2.292333 -2.423331 2.346904

C -3.649938 4.499860 -0.086950

H -2.874478 4.736879 -0.816202

C -4.148743 5.504602 0.750122
C -4.148840 3.193219 -0.000598
H -3.749456 6.519138 0.669727
C -5.148511 5.216340 1.682094
C -5.163765 2.914553 0.927921
C -5.655599 3.914453 1.768037
H -5.534618 6.001642 2.337148
H -5.576747 1.906880 0.996325
H -6.440722 3.676353 2.490425
C -5.667959 0.651105 -1.531235
H -5.616212 0.150756 -0.567665
C -6.686934 0.307859 -2.424188
C -4.713717 1.619791 -1.877536
H -7.418224 -0.452881 -2.138342
C -6.771963 0.929559 -3.673656
C -4.809241 2.245087 -3.131531
C -5.829544 1.901356 -4.022742
H -7.569676 0.659194 -4.370443
H -4.080357 3.008464 -3.408843
H -5.886249 2.398130 -4.994903

Compound $TS_{A1,Ph2}$

50

Potential Energy = -1241.098266124600 Hrt, B3LYP+D3/def2-SVP
O -1.613956 1.559887 0.991265
C -1.866404 0.426810 0.673653
N -1.081816 -0.621493 0.207782
C -3.158469 -0.461248 0.641712
C -2.139084 -1.572323 0.265454
N -1.830192 -3.205333 1.890010
O -2.169141 -2.538026 -0.636166
N -2.141642 -4.172257 1.400639

C -2.934652 -3.621559 -0.675264
C -4.358950 -3.543027 -0.242512
C -2.462728 -4.662776 -1.631024
C 0.302531 -0.755092 0.014267
H -4.747022 -4.559838 -0.083444
H -4.992256 -3.051316 -1.004514
H -4.468349 -2.980013 0.694541
H -2.650998 -5.665522 -1.213416
H -1.385566 -4.554009 -1.821796
H -2.996563 -4.607737 -2.599663
C 0.867065 -2.022890 -0.206713
C 2.246445 -2.137992 -0.391680
H 0.233729 -2.908480 -0.227605
C 3.067828 -1.006212 -0.362334
H 2.680951 -3.126787 -0.560602
C 2.496891 0.252603 -0.140498
H 4.145862 -1.103742 -0.511316
C 1.120957 0.388439 0.047340
H 3.129374 1.143774 -0.112108
H 0.671411 1.364461 0.228042
H -6.265980 1.730248 -3.242212
C -5.646177 1.271573 -2.467485
H -6.290365 2.631698 -0.909421
C -5.662862 1.772615 -1.160780
H -4.787355 -0.205557 -3.794264
C -4.821535 0.187276 -2.774718
C -4.875337 1.181886 -0.172015
C -4.027418 -0.402173 -1.784162
H -4.885549 1.586260 0.841679
C -4.058947 0.074338 -0.465506
H -3.376968 -1.231144 -2.058176
C -3.835393 -0.637376 1.993308
H -5.809676 -1.018594 1.190958

C -5.198976 -0.952184 2.092769
H -2.016499 -0.306289 3.124274
C -3.080201 -0.547847 3.172863
C -5.791565 -1.178431 3.338916
C -3.671029 -0.767770 4.418932
C -5.030151 -1.086272 4.507265
H -6.855219 -1.424902 3.393406
H -3.064735 -0.690251 5.325047
H -5.493228 -1.258713 5.482261

Compound **Ph-5Y-Ph$_2$**

48
Potential Energy = -1131.655186928800 Hrt, B3LYP+D3/def2-SVP
O -1.412537 1.103450 1.580181
C -1.840233 0.542887 0.597500
N -1.235733 0.015390 -0.518787
C -3.248224 0.034669 0.144259
C -2.443588 -0.596568 -1.064905
O -2.685212 -0.040618 -2.326104
C -3.725766 -0.248494 -3.048185
C -4.758020 -1.190141 -2.574682
C -3.717089 0.358300 -4.404375
C 0.109292 -0.146074 -0.872535
H -5.429185 -1.495978 -3.388507
H -5.358225 -0.797922 -1.740045
H -4.202079 -2.056497 -2.151756
H -3.896708 -0.410525 -5.177024
H -2.762819 0.867641 -4.594922
H -4.536589 1.094119 -4.492064
C 0.445763 -0.920986 -1.995827
C 1.786938 -1.086957 -2.347064
H -0.345963 -1.389059 -2.582166

C 2.800814 -0.483598 -1.594751
H 2.040521 -1.693973 -3.220533
C 2.458972 0.288339 -0.477877
H 3.848648 -0.612908 -1.876693
C 1.123950 0.462453 -0.109964
H 3.242381 0.762543 0.119631
H 0.851893 1.055760 0.762728
H -4.155428 4.218356 -1.781778
C -4.563495 3.340436 -1.274006
C -3.706070 2.310259 -0.880803
H -6.608343 4.055718 -1.323222
C -5.935509 3.250397 -1.017215
C -4.196065 1.169179 -0.221662
C -6.436703 2.121875 -0.361511
C -5.574937 1.093722 0.032063
H -7.506556 2.038440 -0.152253
H -5.982264 0.219142 0.541905
H -2.638680 2.393937 -1.097614
H -4.897861 -0.914969 4.410461
C -4.636719 -1.310206 3.425072
C -4.145650 -0.449898 2.441977
H -5.177318 -3.349623 3.921184
C -4.790381 -2.675367 3.152408
C -3.801464 -0.937769 1.168936
C -4.435461 -3.170210 1.894832
C -3.936769 -2.307012 0.911953
H -4.539606 -4.236457 1.675660
H -3.627325 -2.691414 -0.060617
H -4.018413 0.612722 2.659604

Compound $TS_{A2,Ph2}$

48

Potential Energy = -1131.649069545200 Hrt, B3LYP+D3/def2-SVP

O -1.614200 1.332073 0.800388

C -1.415702 0.308008 0.190518

N -0.320402 -0.534836 0.102298

C -2.259458 -0.664971 -0.667540

C -0.946591 -1.570874 -0.649415

O -0.226358 -1.254868 -2.226142

C -0.264974 -2.101239 -3.133528

C -1.122427 -3.313932 -3.027868

C 0.579838 -1.877507 -4.345657

C 0.958013 -0.499185 0.684816

H -1.059314 -3.677516 -1.988005

H -0.851711 -4.079009 -3.767120

H -2.171968 -3.014078 -3.181278

H 1.263976 -2.732984 -4.480332

H 1.146776 -0.941901 -4.261464

H -0.071458 -1.848413 -5.235674

C 1.832377 -1.579684 0.488486

C 3.105567 -1.551301 1.059701

H 1.494689 -2.431770 -0.103093

C 3.517888 -0.452295 1.822495

H 3.782083 -2.396590 0.906513

C 2.640158 0.620618 2.014210

H 4.516819 -0.432936 2.265365

C 1.361666 0.606816 1.451568

H 2.951533 1.482293 2.610846

H 0.670052 1.435798 1.601741

H -4.335044 0.684409 0.399962

H -1.412884 1.613154 -1.977858

H -6.276326 -0.204246 1.679654

H -2.067263 2.403660 -4.227694

C -4.417849 -0.387023 0.596827

C -2.144014 1.043081 -2.554695

C -5.504770 -0.888036 1.315446
C -2.513391 1.489826 -3.826077
C -3.414887 -1.250756 0.126216
C -2.704483 -0.124940 -2.012893
C -5.607282 -2.261757 1.568971
C -3.452304 0.777314 -4.579947
H -6.459803 -2.655076 2.129220
H -3.742946 1.128682 -5.573455
C -3.516198 -2.621848 0.392800
C -3.659420 -0.822089 -2.770261
C -4.610802 -3.125468 1.105865
C -4.026585 -0.380391 -4.044930
H -2.724017 -3.285816 0.043888
H -4.123695 -1.717144 -2.350424
H -4.680976 -4.198778 1.303328
H -4.771280 -0.939414 -4.618172

Compound **Ph-6-Ph$_2$**

38
Potential Energy = -938.614090338600 Hrt, B3LYP+D3/def2-SVP
O -2.389836 2.208307 -2.913933
C -2.693126 2.162984 -1.760859
N -2.014941 1.642290 -0.603100
C -3.829602 2.615209 -0.822744
C -2.923467 2.034501 0.314893
C -0.781312 0.950760 -0.449828
C 0.010663 0.684969 -1.574161
C 1.218158 0.000469 -1.406599
H -0.316154 1.011001 -2.561521
C 1.628766 -0.412519 -0.135698
H 1.840445 -0.209641 -2.280004
C 0.825866 -0.139419 0.979362

H 2.573500 -0.947619 -0.011768
C -0.381607 0.542317 0.829148
H 1.142356 -0.461065 1.974768
H -1.023110 0.764510 1.683334
C -3.924914 4.832068 0.415291
H -3.632539 4.305015 1.325945
C -4.166087 6.210321 0.438560
C -4.055262 4.115400 -0.781633
H -4.064965 6.761406 1.377354
C -4.531346 6.880869 -0.732353
C -4.422691 4.791129 -1.955283
C -4.656498 6.167652 -1.930947
H -4.717321 7.957935 -0.713427
H -4.524873 4.234223 -2.889875
H -4.940013 6.686259 -2.850628
C -5.970412 1.709915 0.143538
H -5.670522 2.150426 1.096992
C -7.180236 1.021115 0.035987
C -5.128572 1.840833 -0.972161
H -7.827716 0.922867 0.911376
C -7.563299 0.457606 -1.187177
C -5.514395 1.275010 -2.195129
C -6.728786 0.587603 -2.300836
H -8.510709 -0.081179 -1.270760
H -4.868873 1.374755 -3.070224
H -7.021219 0.152240 -3.260010

Compound **Ph-5-sCy**

43

Potential Energy = -974.569943132300 Hrt, B3LYP+D3/def2-SVP

H -1.953426 -0.384342 6.775322

H -1.705766 0.117250 9.211625

C -1.125449 0.235790 7.128427
C -0.989246 0.518906 8.491051
C -0.215462 0.751030 6.202263
H -0.351004 0.552711 5.140062
C 0.068256 1.327480 8.922685
H 0.180800 1.561256 9.984633
C 0.853634 1.546105 6.643135
C 0.992981 1.839557 8.010564
N 1.787893 2.057934 5.724989
H 1.816146 2.472432 8.341757
C 2.176180 1.626567 4.392714
C 2.721303 3.082418 5.793414
O 2.988107 3.869044 6.670170
H 4.850521 1.518248 5.082314
H 4.862061 1.838990 3.343878
H 6.703757 3.097273 4.466392
C 4.691360 2.305248 4.329647
C 5.668185 3.468559 4.538388
C 3.237998 2.790328 4.374922
H 5.539140 3.864338 5.559688
H 5.653016 4.223458 2.508577
C 5.426570 4.593092 3.526071
H 3.116967 3.500380 2.350148
H 6.114972 5.432955 3.716623
C 2.990406 3.918387 3.364727
C 3.973401 5.075842 3.577509
H 1.949558 4.268001 3.436454
H 3.777401 5.537538 4.560114
H 3.795942 5.854603 2.817570
O 2.640054 0.316886 4.315045
C 2.073251 -0.299484 3.159620
C 1.400074 -1.611877 3.536658
H 2.160351 -2.316007 3.905822

H 0.906075 -2.050432 2.657880
H 0.654518 -1.453623 4.328704
C 3.120949 -0.459389 2.059580
H 3.546837 0.517012 1.785188
H 2.669850 -0.912251 1.164982
H 3.928750 -1.111372 2.422849
N 1.108905 1.721652 3.364979
N 1.057674 0.684262 2.698989

Compound $TS_{A1,sCy}$

43
Potential Energy = -974.518504459600 Hrt, B3LYP+D3/def2-SVP
O -1.424572 1.530908 0.862320
C -1.729722 0.390499 0.599426
N -0.965115 -0.755955 0.400172
C -2.105130 -1.614394 0.327545
N -2.239248 -3.366952 2.074129
O -2.060010 -2.603920 -0.554944
N -2.497636 -4.296625 1.506702
C -2.881899 -3.602279 -0.797066
C -4.346468 -3.462231 -0.569557
C -2.309710 -4.689096 -1.640358
C 0.409571 -1.014905 0.495972
H -4.797068 -4.459887 -0.456803
H -4.853847 -2.963900 -1.417533
H -4.557376 -2.876310 0.338152
H -2.636086 -5.674043 -1.266280
H -1.211122 -4.649568 -1.628288
H -2.647229 -4.615928 -2.693111
C 0.880810 -2.339326 0.480016
C 2.251488 -2.585551 0.582611
H 0.178518 -3.167328 0.394891

C 3.160230 -1.528301 0.696899
H 2.610086 -3.618313 0.573523
C 2.683505 -0.212101 0.711580
H 4.231728 -1.728061 0.774585
C 1.317245 0.054329 0.611543
H 3.383619 0.622559 0.803694
H 0.940447 1.076697 0.631275
H -5.350000 1.060756 2.599360
H -3.421348 -0.563906 2.551615
C -4.653289 1.053245 1.745020
C -3.986208 -0.323593 1.637361
H -4.758778 -1.104636 1.526859
H -3.880009 1.811175 1.954460
C -5.386041 1.422758 0.450656
H -6.223553 0.718300 0.290994
H -5.831299 2.427699 0.537343
C -3.053768 -0.400632 0.421538
C -4.442774 1.362790 -0.755960
C -3.787180 -0.019207 -0.877778
H -4.566123 -0.772257 -1.075778
H -3.658149 2.130059 -0.646701
H -4.988797 1.593466 -1.685710
H -3.084092 -0.048896 -1.726319

Compound **Ph-5Y-sCy**

41
Potential Energy = -865.069879018400 Hrt, B3LYP+D3/def2-SVP
C -4.878546 -4.704261 1.177249
C -3.569350 -4.254315 1.000171
C -5.512119 -4.597639 2.421262
C -2.877065 -3.684891 2.085979
C -4.818133 -4.032375 3.496790

C 0.479639 -2.734589 1.752565
C -3.508849 -3.574012 3.337244
C -0.633471 -3.407096 0.917036
H -5.409263 -5.145982 0.329351
H -6.537975 -4.951294 2.550591
H -5.300557 -3.944760 4.474246
N -1.562900 -3.231494 1.925353
O -0.698240 -3.950070 -0.166561
H -2.968638 -3.132390 4.175553
H -3.067775 -4.343839 0.036842
C 1.711395 -3.617505 1.979108
C 0.862285 -1.363107 1.168951
C 1.708574 -1.514071 -0.102111
C 2.550339 -3.757192 0.702838
C 2.944760 -2.385204 0.145058
H 1.961201 -4.300497 -0.054661
H 3.448082 -4.363386 0.909208
H 3.520455 -2.502297 -0.788181
H 3.612643 -1.875661 0.865189
H 2.004510 -0.517012 -0.469282
H 1.090123 -1.974991 -0.890359
H 2.324258 -3.163848 2.777672
H 1.390009 -4.603843 2.350087
H -0.048239 -0.778297 0.962086
H 1.435305 -0.795913 1.922786
H 1.222959 -0.789602 5.664020
C 0.942135 -1.266967 4.714385
H 0.795070 -2.371147 4.819647
C -0.600146 -2.717764 2.885619
C -0.347318 -0.758800 4.212085
H -1.132832 0.321673 5.886508
O -1.013659 -1.483437 3.378324
C -1.065786 0.410446 4.785946

H -0.520240 1.348009 4.579379

H -2.076295 0.486303 4.361658

H 1.780494 -1.161222 4.008094

Compound $TS_{A2,sCy}$

41

Potential Energy = -865.063046544100 Hrt, B3LYP+D3/def2-SVP

O -1.878521 0.640223 1.166023

C -1.565601 -0.175691 0.327043

N -0.382846 -0.874936 0.100649

C -2.272047 -0.981007 -0.775180

C -0.929245 -1.799249 -0.832697

O -0.124900 -1.311569 -2.327344

C -0.157645 -2.062486 -3.318721

C -1.134963 -3.182572 -3.391424

C 0.850052 -1.858570 -4.404835

C 0.860714 -0.879415 0.754367

H -1.146803 -3.642378 -2.383286

H -0.898400 -3.897217 -4.190470

H -2.151383 -2.785380 -3.543189

H 1.461775 -2.772773 -4.505511

H 1.492953 -0.996658 -4.185147

H 0.334681 -1.716312 -5.369446

C 1.842241 -1.804406 0.363021

C 3.081717 -1.811908 1.004800

H 1.615858 -2.508332 -0.438541

C 3.356134 -0.901571 2.032255

H 3.841511 -2.535681 0.697170

C 2.373666 0.018202 2.416289

H 4.329512 -0.908335 2.529181

C 1.127254 0.037052 1.786031

H 2.577030 0.731944 3.219244

H 0.354635 0.745988 2.083636

H -4.193459 1.314776 -2.563038

H -3.649326 1.459241 -0.882921

C -3.910535 0.731839 -1.670281

C -2.677041 -0.125796 -1.983531

H -5.953863 0.512125 -0.952709

C -5.087117 -0.125930 -1.192938

H -5.404223 -0.796191 -2.013720

H -4.468337 -0.301667 0.872069

C -4.690705 -0.969795 0.022889

C -3.454594 -1.824655 -0.277728

H -5.527180 -1.617395 0.334968

H -3.688989 -2.562686 -1.066239

H -2.904790 -0.795674 -2.832252

H -1.835129 0.510789 -2.295031

H -3.149302 -2.401086 0.610753

Compound **Ph-6-sCy**

31

Potential Energy = -672.033611107200 Hrt, B3LYP+D3/def2-SVP

H -1.706152 -0.855113 5.818054

H -3.151234 0.675964 7.160555

C -1.338304 0.132678 6.107052

C -2.149046 0.991720 6.860275

C -0.058179 0.528995 5.720050

H 0.590243 -0.124000 5.133726

C -1.671900 2.253619 7.226996

H -2.299550 2.928189 7.814711

C 0.408599 1.796453 6.092949

C -0.390850 2.665457 6.847086

N 1.712255 2.201838 5.697271

H -0.010021 3.646707 7.130490

C 2.657399 1.561720 4.972399

C 2.473944 3.405846 5.938102

O 2.224873 4.397248 6.557675

H 4.655761 2.014976 6.858092

H 5.485240 1.772156 5.310902

H 6.647029 3.536665 6.650961

C 4.897369 2.499449 5.897727

C 5.711471 3.779906 6.120764

C 3.608426 2.780811 5.112225

H 5.141597 4.460272 6.776917

H 6.670450 3.845849 4.181585

C 6.012462 4.488347 4.795133

H 4.472721 2.796880 3.153215

H 6.566791 5.423417 4.979146

C 3.907775 3.500921 3.788887

C 4.723931 4.779052 4.017436

H 2.966888 3.724760 3.259845

H 4.111816 5.502099 4.584044

H 4.954828 5.249464 3.047415

Compound $TS1_{Cl2}$

48

Potential Energy = -3093.958210622100 Hrt, B3LYP+D3/def2-SVP

C -1.936831 -2.482551 7.072197

C -0.902837 -2.297587 6.148963

C -3.125189 -1.756892 6.933754

C -1.048169 -1.394199 5.093929

C -3.282388 -0.841438 5.891360

C -2.236646 -0.659535 4.973230

H -1.819816 -3.193756 7.893353

H 0.025470 -2.867240 6.238387

H -3.941249 -1.899418 7.646593

H	-4.207094	-0.275715	5.777498
H	-0.248133	-1.286736	4.362882
N	-2.392412	0.267914	3.920865
C	-1.402261	0.989838	3.151685
C	-3.506689	0.858157	3.349936
O	-4.684584	0.756330	3.543419
C	-2.611162	1.677859	2.387461
Cl	-2.799841	1.225929	0.682841
Cl	-2.681844	3.428935	2.620936
O	-0.559865	1.791849	3.887801
N	-0.519198	0.148341	2.306496
C	0.765856	1.694921	3.350161
N	0.641994	0.522897	2.447122
C	1.102188	2.928288	2.518058
C	1.764363	1.406931	4.457611
H	2.088505	2.804269	2.052802
H	1.104088	3.806381	3.179757
H	0.349508	3.077100	1.730381
H	2.756818	1.227791	4.021431
H	1.457488	0.523040	5.033118
H	1.813990	2.276238	5.128826
H	-0.467243	3.146187	-2.153691
H	-2.191597	1.783602	-3.338089
C	-0.524442	2.067813	-1.985928
C	-1.490685	1.303636	-2.650854
C	0.366162	1.462081	-1.100568
H	1.121919	2.040881	-0.568425
C	-1.558489	-0.076095	-2.429791
H	-2.311290	-0.677191	-2.945799
C	0.285249	0.079615	-0.883861
C	-0.671760	-0.699089	-1.548293
N	1.169809	-0.520604	0.040523
H	-0.722021	-1.772351	-1.365489

C 2.054499 0.085365 0.920736
C 1.410686 -1.868209 0.381189
C 2.500013 -1.355359 1.352202
O 0.968288 -2.910863 0.005681
Cl 4.149170 -1.700723 0.751100
Cl 2.343187 -1.864643 3.050518

Compound **Ph-5-Cl$_2$-Ph-6-Cl$_2$**

48
Potential Energy = -3094.028814520700 Hrt, B3LYP+D3/def2-SVP
C -1.958455 -2.230073 6.984996
C -0.938972 -1.348531 6.610149
C -3.164796 -2.227557 6.276764
C -1.115775 -0.469014 5.540720
C -3.361317 -1.353039 5.206196
C -2.332581 -0.469288 4.838709
H -1.812112 -2.916642 7.822344
H 0.010573 -1.345489 7.151640
H -3.967480 -2.913852 6.558793
H -4.298269 -1.347372 4.649776
H -0.308932 0.196795 5.241421
N -2.531386 0.420243 3.766634
C -1.692049 1.518929 3.291353
C -3.554017 0.567569 2.855737
O -4.590493 -0.014778 2.674213
C -2.835891 1.755609 2.181662
Cl -2.342666 1.443839 0.507298
Cl -3.718410 3.293090 2.312370
O -1.591689 2.544517 4.243593
N -0.390386 1.156305 2.887655
C -0.357113 3.197826 4.096393
N 0.425885 2.085940 3.369374

C -0.452734 4.392800 3.153314
C 0.174142 3.539400 5.472776
H 0.534527 4.853994 3.015171
H -1.138591 5.130669 3.593169
H -0.851904 4.085492 2.176674
H 1.117144 4.096312 5.419017
H 0.308532 2.632077 6.075469
H -0.575466 4.180097 5.959698
H 0.043816 0.126638 -1.435264
H 1.503012 -1.852040 -1.874381
C 0.883247 -0.096102 -0.772295
C 1.701032 -1.204279 -1.016963
C 1.132503 0.739546 0.317245
H 0.504480 1.607958 0.503866
C 2.775666 -1.479975 -0.163199
H 3.419193 -2.342832 -0.351238
C 2.196573 0.440931 1.178121
C 3.026888 -0.664497 0.941361
N 2.477278 1.280065 2.280287
H 3.855770 -0.878399 1.617399
C 1.704049 2.121165 3.113504
C 3.697702 1.761567 2.756939
C 2.938123 2.706617 3.753965
O 4.837547 1.528737 2.493923
Cl 3.266804 4.439151 3.497187
Cl 3.249725 2.232952 5.452877

Compound $TS2_{Cl2}$

48

Potential Energy = -3093.997399333600 Hrt, B3LYP+D3/def2-SVP

C -4.260597 -2.738168 2.775935
C -3.461819 -2.553530 3.910450

C -4.454950 -1.676539 1.886470
C -2.856603 -1.321403 4.160163
C -3.853725 -0.437235 2.117146
C -3.051846 -0.266270 3.256152
H -4.731499 -3.706076 2.587574
H -3.309374 -3.376725 4.613093
H -5.080248 -1.810169 1.000100
H -4.003366 0.396116 1.431134
H -2.239313 -1.168288 5.043625
N -2.450988 0.986218 3.500042
C -1.387204 1.341967 4.366938
C -2.606950 2.230889 2.908606
O -3.295831 2.639612 2.017336
C -1.546189 2.843189 3.856707
Cl -0.090413 3.486518 3.069344
Cl -2.279918 4.065944 4.915824
O -1.943779 1.347289 6.027485
N -0.327335 0.542314 4.422495
C -1.015028 1.380253 6.910060
N 0.448553 0.749996 5.481674
C -0.368509 2.682729 7.305391
C -1.024005 0.264020 7.919863
H 0.541512 2.509116 7.892303
H -1.089680 3.238143 7.931339
H -0.131263 3.304466 6.435126
H -0.073858 0.185101 8.462010
H -1.262923 -0.690207 7.430837
H -1.821349 0.482201 8.653231
H 1.394987 0.771721 0.215529
H 2.682361 -1.214586 -0.585511
C 1.947216 0.150761 0.925264
C 2.669742 -0.960661 0.477289
C 1.929775 0.485430 2.280094

H 1.377180 1.354575 2.628861
C 3.379835 -1.743296 1.394035
H 3.949182 -2.610903 1.051647
C 2.628919 -0.315080 3.191941
C 3.360668 -1.428807 2.754404
N 2.642774 0.002039 4.573514
H 3.909187 -2.037398 3.474300
C 1.687756 0.465248 5.518061
C 3.678691 -0.097240 5.505619
C 2.723197 0.420338 6.628742
O 4.816348 -0.454197 5.438339
Cl 3.212368 1.992498 7.306230
Cl 2.443050 -0.787435 7.907536

Compound **[Ph-6-Cl$_2$*N]$_2$**

38
Potential Energy = -2900.989778997000 Hrt, B3LYP+D3/def2-SVP
C -0.730406 6.327743 1.382342
C -0.832378 4.941953 1.554758
C -1.301800 7.192276 2.321526
C -1.500725 4.417584 2.661401
C -1.967272 6.678736 3.438051
C -2.055903 5.291713 3.604599
H -0.206914 6.733128 0.512978
H -0.386477 4.263144 0.824189
H -1.227046 8.274370 2.189776
H -2.412512 7.344159 4.179696
H -1.582831 3.339899 2.807130
N -2.764342 4.777864 4.722628
C -2.510864 3.797656 5.701840
C -4.063899 5.062160 5.175640
O -4.895743 5.838905 4.819662

C -3.912577 3.964807 6.278023
Cl -3.967826 4.575289 7.941210
Cl -4.982380 2.566978 6.033028
N -1.540237 3.071990 6.071763
N -0.368486 3.260153 5.349290
H 5.681405 3.110713 2.806940
H 4.604700 4.958863 1.519649
C 4.648146 3.396836 3.018488
C 4.044236 4.433312 2.296636
C 3.942441 2.716204 4.011718
H 4.407327 1.908072 4.575942
C 2.721379 4.791851 2.574429
H 2.236313 5.597089 2.017634
C 2.613961 3.086841 4.280049
C 1.998089 4.126331 3.565988
N 1.893030 2.403125 5.281017
H 0.967904 4.399736 3.785766
C 0.574859 2.503940 5.738133
C 2.247357 1.380408 6.179687
C 0.806384 1.407498 6.777671
O 3.255871 0.768786 6.367142
Cl 0.740609 1.954288 8.464064
Cl -0.113838 -0.085268 6.506093

Compound $TS1_{Ph2}$

88
Potential Energy = -2179.782228629000 Hrt, B3LYP+D3/def2-SVP
C -2.496179 -3.753187 5.928859
C -2.204234 -4.512166 7.067597
C -1.974198 -2.461303 5.811868
C -1.385690 -3.999237 8.075832
C -1.167147 -1.928186 6.819202

C -0.859381 -2.702989 7.947538
H -3.131284 -4.164284 5.140448
H -2.612387 -5.520657 7.173257
H -2.197880 -1.852771 4.933019
H -0.794657 -0.909234 6.730405
H -1.157097 -4.586236 8.964852
N -0.022628 -2.183873 8.956500
C 1.101131 -1.259534 8.878073
C 0.265815 -2.623765 10.235262
O -0.195449 -3.508108 10.910079
C 1.358886 -1.542654 10.440508
O 2.102362 -1.701052 8.021278
N 0.783035 0.107207 8.422409
C 2.488028 -0.643421 7.140610
N 1.532618 0.438828 7.498203
C 3.925856 -0.226021 7.418637
C 2.264531 -1.058761 5.692010
H 4.214270 0.596070 6.753183
H 4.577838 -1.096209 7.251758
H 4.034442 0.103398 8.458728
H 2.501239 -0.224164 5.020535
H 1.221729 -1.360686 5.528275
H 2.923036 -1.909240 5.463926
H 0.102722 4.936126 12.320613
H 1.742221 3.524716 13.561422
C 0.763766 4.257155 11.776217
C 1.685696 3.470063 12.472341
C 0.670471 4.179741 10.384812
H -0.045481 4.788761 9.833635
C 2.523363 2.596989 11.771132
H 3.231780 1.966985 12.313286
C 1.522941 3.308147 9.692893
C 2.449895 2.516305 10.381676

N 1.462944 3.230269 8.279750
H 3.102898 1.855166 9.812405
C 2.270514 2.560714 7.395778
C 0.697485 3.940886 7.322419
C 1.472008 3.219869 6.197494
O -0.127023 4.803953 7.426628
H 5.561072 4.670284 4.378718
C 4.485568 4.840150 4.477192
H 4.486857 6.602352 3.220170
C 3.884354 5.922532 3.828385
C 3.715536 3.971344 5.258704
H 4.180825 3.129853 5.774984
C 2.506513 6.133266 3.963181
C 2.335180 4.174127 5.389552
H 2.029924 6.979888 3.461892
C 1.735987 5.263280 4.737351
H 0.660823 5.425588 4.842599
H 0.728488 0.681721 2.317464
C 0.333823 1.023607 3.277847
H -1.545546 -0.031557 3.064954
C -0.937471 0.621269 3.696584
C 1.104075 1.870713 4.080332
H 2.090609 2.191356 3.740818
C -1.430843 1.071851 4.926527
C 0.623730 2.315863 5.320052
H -2.425777 0.769580 5.262653
C -0.655691 1.908680 5.731259
H -1.056725 2.247275 6.688950
H 1.173175 0.036804 14.838711
C 0.782500 0.132785 13.822142
H -0.677089 1.644461 14.344148
C -0.255082 1.025895 13.548026
C 1.326568 -0.648798 12.799456

H 2.126067 -1.352063 13.034227
C -0.744137 1.125914 12.243172
C 0.848986 -0.549066 11.482400
H -1.549535 1.826598 12.010648
C -0.205031 0.342500 11.221497
H -0.601734 0.437960 10.213358
H 4.641177 -4.892431 10.804298
C 4.408938 -3.824450 10.821050
H 6.465068 -3.217494 11.122040
C 5.430231 -2.887379 10.999485
C 3.084197 -3.403303 10.661883
H 2.294973 -4.144847 10.527475
C 5.118816 -1.522884 11.027302
C 2.765269 -2.039732 10.684860
H 5.908786 -0.780588 11.168365
C 3.795898 -1.105975 10.875132
H 3.552544 -0.043750 10.910219

Compound **Ph-5-Ph$_2$-Ph-6-Ph$_2$**

88
Potential Energy = -2179.846949518300 Hrt, B3LYP+D3/def2-SVP
C 0.705172 -1.826980 8.904135
C 1.364828 -1.570356 7.697270
C -0.693512 -1.796719 8.944538
C 0.636862 -1.285616 6.540084
C -1.433659 -1.496604 7.798760
C -0.765403 -1.233446 6.591504
H 1.277118 -2.056617 9.806580
H 2.456657 -1.604568 7.650384
H -1.218129 -2.003563 9.881112
H -2.523120 -1.476829 7.823099
H 1.133234 -1.121386 5.585250

N -1.499119 -0.938538 5.428445
C -1.214226 -0.050578 4.288174
C -2.792825 -1.232863 5.057416
O -3.672845 -1.837529 5.624101
C -2.650329 -0.521590 3.693724
O -1.227690 1.312302 4.672735
N -0.046914 -0.280773 3.538677
C -0.057503 1.969217 4.249297
N 0.507009 0.905775 3.302831
C -0.416226 3.275994 3.572953
C 0.883227 2.166353 5.436216
H 0.486093 3.829814 3.286112
H -0.975056 3.880373 4.301912
H -1.046472 3.112252 2.694301
H 1.755846 2.774605 5.167716
H 1.211738 1.200268 5.840587
H 0.321933 2.696859 6.219314
H 2.138266 -4.271858 0.022639
H 2.567918 -5.226345 2.289406
C 2.221509 -3.611656 0.889452
C 2.461023 -4.146586 2.160093
C 2.066754 -2.234201 0.719513
H 1.861485 -1.810751 -0.264454
C 2.568148 -3.293308 3.265029
H 2.760862 -3.702062 4.260110
C 2.164810 -1.390484 1.833216
C 2.433235 -1.914108 3.104298
N 2.008092 0.002585 1.658521
H 2.536890 -1.238690 3.953667
C 1.503927 1.049565 2.462936
C 2.488035 0.858944 0.668780
C 1.961517 2.123096 1.449083
O 3.078208 0.670473 -0.356991

H -0.526586 5.789149 -0.205913
C -0.440882 4.704370 -0.102707
H -2.308473 4.296753 -1.120067
C -1.439717 3.870446 -0.612144
C 0.672498 4.160395 0.546915
H 1.434631 4.827797 0.950386
C -1.318780 2.483511 -0.467232
C 0.796828 2.775094 0.703543
H -2.094807 1.818377 -0.853333
C -0.212018 1.942833 0.187506
H -0.150732 0.859310 0.318523
H 5.194115 3.810665 4.421762
C 4.795767 3.802594 3.403968
H 6.291550 5.160902 2.628315
C 5.411597 4.554000 2.400337
C 3.668798 3.028106 3.108264
H 3.210989 2.417163 3.886179
C 4.902927 4.513335 1.096435
C 3.134424 3.006466 1.814889
H 5.390455 5.081106 0.299783
C 3.777133 3.741837 0.803604
H 3.398567 3.699352 -0.219362
H -3.933101 -2.334334 -0.467946
C -3.269317 -2.408068 0.397790
H -2.230559 -4.160188 -0.337307
C -2.322141 -3.431360 0.472402
C -3.380172 -1.469809 1.431174
H -4.130736 -0.683213 1.356187
C -1.495787 -3.518106 1.598814
C -2.542880 -1.532891 2.554831
H -0.748379 -4.311411 1.675913
C -1.610978 -2.585161 2.629317
H -0.954248 -2.670728 3.494261

H -6.489563 2.084783 4.488122

C -5.581565 1.933806 3.898107

H -5.898698 3.687198 2.668707

C -5.252503 2.830715 2.878552

C -4.754716 0.836511 4.171882

H -5.021348 0.134470 4.963502

C -4.090839 2.622055 2.123579

C -3.589224 0.625506 3.424904

H -3.817829 3.312951 1.321606

C -3.272475 1.526745 2.394928

H -2.374707 1.362257 1.798534

Compound $TS2_{Ph2}$

88

Potential Energy = -2179.823772632900 Hrt, B3LYP+D3/def2-SVP

C 0.281708 -3.818077 8.001879

C 1.021570 -2.837002 7.333635

C -1.085595 -3.949720 7.734604

C 0.408333 -1.992602 6.405457

C -1.717313 -3.105584 6.819291

C -0.967532 -2.119242 6.156188

H 0.768260 -4.479873 8.722707

H 2.092191 -2.730406 7.527829

H -1.672747 -4.716033 8.247547

H -2.781028 -3.206250 6.606069

H 0.975219 -1.241565 5.857902

N -1.608672 -1.264584 5.237094

C -1.259357 0.014314 4.720991

C -2.825458 -1.353000 4.577796

O -3.679038 -2.204990 4.586259

C -2.615073 -0.003763 3.847958

O -1.519056 1.204548 5.919528

N -0.006495 0.239874 4.299334
C -0.580221 2.105985 5.975121
N 0.219436 1.554583 4.221499
C -0.968992 3.553965 5.847894
C 0.564635 1.807798 6.918653
H -0.087617 4.185505 5.677914
H -1.424373 3.863024 6.805390
H -1.704533 3.711771 5.056033
H 1.325933 2.597856 6.922782
H 1.022347 0.836135 6.692288
H 0.131542 1.737769 7.931340
H 2.258781 -1.123405 -1.465733
H 2.787172 -3.072015 0.006651
C 2.213637 -1.019925 -0.378760
C 2.507909 -2.110872 0.445276
C 1.843613 0.205658 0.176992
H 1.604502 1.055617 -0.461657
C 2.443493 -1.964690 1.835522
H 2.675050 -2.810673 2.487802
C 1.785931 0.343522 1.571711
C 2.098875 -0.738341 2.406243
N 1.473172 1.616427 2.106100
H 2.049761 -0.622311 3.486795
C 0.899911 2.118711 3.298840
C 1.800227 2.890606 1.623088
C 1.224449 3.572740 2.909478
O 2.323412 3.263860 0.612274
H -1.586160 7.330002 3.435297
C -1.410410 6.374068 2.935385
H -3.240186 6.455434 1.781111
C -2.337168 5.884366 2.010995
C -0.253631 5.644401 3.229905
H 0.459316 6.034831 3.956797

C -2.102882 4.655679 1.384089
C -0.011505 4.414993 2.606437
H -2.824724 4.254547 0.668519
C -0.950302 3.927380 1.681252
H -0.797303 2.959634 1.196087
H 4.398172 3.893847 6.369231
C 3.980944 4.332551 5.459266
H 5.365834 5.994946 5.425681
C 4.523046 5.506299 4.930256
C 2.902073 3.708727 4.822830
H 2.492947 2.783821 5.227398
C 3.986790 6.048913 3.755739
C 2.347186 4.257336 3.660901
H 4.414128 6.959127 3.327336
C 2.908974 5.427908 3.122974
H 2.501590 5.845272 2.200103
H -3.084110 0.964752 -0.757604
C -2.603854 0.347737 0.006717
H -1.442673 -0.805150 -1.412214
C -1.692023 -0.643649 -0.360282
C -2.918534 0.555877 1.355389
H -3.645832 1.322908 1.617010
C -1.101037 -1.431226 0.633357
C -2.314074 -0.211187 2.361964
H -0.382369 -2.207274 0.364546
C -1.404733 -1.213743 1.975510
H -0.914333 -1.828432 2.730988
H -6.832737 1.440918 5.347269
C -5.866172 1.713952 4.915337
H -6.343281 3.820650 4.770216
C -5.593892 3.044861 4.592153
C -4.908456 0.718118 4.684372
H -5.141586 -0.320172 4.922854

C -4.353904 3.376807 4.031554
C -3.662243 1.044344 4.133497
H -4.123636 4.411421 3.766470
C -3.401915 2.383666 3.807735
H -2.442183 2.645977 3.364068

Compound **[Ph-6-Ph$_2$*N]$_2$**

78
Potential Energy = -1986.831795726700 Hrt, B3LYP+D3/def2-SVP
C -2.884404 -2.087888 3.445393
C -1.757503 -1.525950 4.054442
C -3.538000 -1.388504 2.424239
C -1.282848 -0.274420 3.655850
C -3.082259 -0.134319 2.014649
C -1.953568 0.423619 2.638836
H -3.249970 -3.067708 3.762113
H -1.235772 -2.066610 4.848444
H -4.416927 -1.821260 1.939574
H -3.587759 0.415792 1.221279
H -0.403757 0.170001 4.119375
N -1.502905 1.698805 2.245042
C -0.552504 2.590789 2.773760
C -1.901641 2.583594 1.229983
O -2.732190 2.499331 0.367632
C -0.895185 3.672238 1.728765
N 0.213679 2.438345 3.772106
N 1.089067 3.470135 4.033017
H -4.172014 4.208460 7.967962
H -4.344123 1.734409 7.676228
C -3.340407 3.647808 7.533761
C -3.435988 2.260629 7.372268
C -2.191531 4.329469 7.129168

H -2.113409 5.410894 7.239882
C -2.361492 1.550308 6.827165
H -2.423721 0.466830 6.699856
C -1.126040 3.610862 6.564401
C -1.197614 2.217188 6.438237
N 0.018520 4.316315 6.129099
H -0.355230 1.669596 6.020288
C 0.951788 4.179755 5.071942
C 0.617610 5.490138 6.618772
C 1.746699 5.406253 5.547161
O 0.312984 6.227406 7.514609
H -3.440772 5.843987 5.021237
H -2.166255 3.921667 4.158629
C -2.982521 5.906380 4.031926
C -2.251586 4.819888 3.545317
H -3.691395 7.913989 3.637438
C -3.123453 7.061433 3.256501
C -1.639722 4.877639 2.284473
C -2.535533 7.115964 1.988536
C -1.797786 6.032392 1.505679
H -2.646343 8.011303 1.371367
H -1.333269 6.087134 0.519803
H 3.043332 5.961467 0.683263
H 1.097242 5.473889 2.126883
C 2.308190 5.220283 0.361946
C 1.211993 4.946842 1.181004
H 3.320435 4.766734 -1.498918
C 2.462029 4.550296 -0.857537
C 0.254756 3.995528 0.791964
C 1.509872 3.607418 -1.253145
C 0.411015 3.330175 -0.431891
H 1.616338 3.084458 -2.207071
H -0.332433 2.600574 -0.759196

H 5.764803 2.987483 5.924212
H 3.527545 3.402542 4.911155
C 5.150903 3.817514 6.283768
C 3.892439 4.044090 5.715433
H 6.605939 4.468954 7.747755
C 5.622758 4.648039 7.304679
C 3.098610 5.106501 6.167125
C 4.831332 5.710231 7.757295
C 3.576007 5.939992 7.190180
H 5.192648 6.362745 8.556479
H 2.955146 6.766696 7.542853
H -0.379030 8.697623 2.971031
H -0.375108 6.922392 4.673443
C 0.560190 8.202706 3.223570
C 0.566415 7.199861 4.194939
H 1.739399 9.340894 1.808383
C 1.745351 8.558901 2.571983
C 1.760315 6.544161 4.534736
C 2.936875 7.907034 2.902350
C 2.945003 6.906865 3.879749
H 3.868233 8.175450 2.396937
H 3.878290 6.400476 4.129900

Compound $TS1_{sCy}$

74

Potential Energy = -1646.616667923800 Hrt, B3LYP+D3/def2-SVP

H -9.078701 3.023007 4.090540
H -10.964971 1.625038 4.941590
C -8.956783 1.941636 4.193264
C -10.015407 1.158445 4.668748
C -7.740295 1.350371 3.848664
H -6.903514 1.942876 3.476675

C -9.849987 -0.225133 4.794073
H -10.671499 -0.842601 5.166029
C -7.581443 -0.036144 3.985147
C -8.637584 -0.830347 4.453629
N -6.340584 -0.625978 3.663836
H -8.500338 -1.907623 4.549642
C -5.097880 -0.041075 3.484680
C -5.959653 -1.947385 3.335679
O -6.598126 -2.960012 3.220990
H -4.869265 -1.063143 0.998311
H -3.200820 -0.830329 1.541416
H -3.374405 -2.884685 0.131840
C -4.079622 -1.492578 1.636647
C -3.718861 -2.909545 1.179184
C -4.519664 -1.449764 3.111151
H -4.624104 -3.540697 1.201268
H -1.710612 -2.946696 1.984022
C -2.648074 -3.524548 2.085209
H -2.567416 -1.444873 4.009390
H -2.420319 -4.555515 1.767277
C -3.462637 -2.091286 4.017775
C -3.097745 -3.505696 3.549220
H -3.827330 -2.120747 5.054140
H -3.979797 -4.159008 3.665944
H -2.309979 -3.918107 4.201839
H -7.436426 4.461457 9.293144
H -5.358887 3.096244 8.939594
C -7.513770 3.366464 9.191122
H -7.505613 2.946843 10.211291
C -6.295869 2.837408 8.423581
N -5.281124 -0.167833 6.418654
H -6.251568 3.306506 7.425913
N -4.538136 0.217990 5.513359

C -2.902949 -2.222703 11.086959
H -2.844314 -2.473425 12.149361
C -3.874619 -1.318557 10.654170
C -2.008735 -2.801897 10.179670
H -4.574957 -0.867632 11.356802
C -8.822632 2.978462 8.494577
H -9.687110 3.351285 9.068434
H -1.248051 -3.505097 10.527082
O -6.529382 0.327674 10.564203
C -3.959353 -0.991552 9.289492
C -6.050396 0.486203 9.467466
C -2.102140 -2.476636 8.823078
N -4.935074 -0.075812 8.857549
C -6.387567 1.321834 8.223430
C -3.074446 -1.580617 8.372764
C -5.084424 0.670181 7.622616
H -8.865114 3.463593 7.501953
H -1.417018 -2.927266 8.100312
H -3.148307 -1.357723 7.310637
C -3.733848 1.407093 5.915318
C -8.914643 1.460183 8.313427
C -4.248205 2.646927 5.189198
H -8.965621 0.975408 9.302992
H -1.917965 0.253338 6.215412
C -7.703389 0.921785 7.545399
O -4.004558 1.497277 7.314262
C -2.256069 1.146315 5.672139
H -7.696204 1.340534 6.528011
H -2.076585 1.007183 4.596591
H -9.834071 1.188122 7.770800
H -7.758175 -0.171053 7.436817
H -1.674579 2.012380 6.020194
H -4.036509 2.570521 4.115867

H -3.750532 3.531279 5.614106

H -5.334694 2.748108 5.322578

Compound **Ph-5-sCy-Ph-6-sCy**

74

Potential Energy = -1646.682299332800 Hrt, B3LYP+D3/def2-SVP

H -6.071348 1.304763 2.936050

H -5.627863 -0.868961 1.786757

C -5.173383 0.729176 3.174631

C -4.924201 -0.488322 2.531210

C -4.276716 1.222800 4.123135

H -4.457716 2.173147 4.621815

C -3.768785 -1.214849 2.841475

H -3.566382 -2.164289 2.339466

C -3.135080 0.475779 4.446920

C -2.872784 -0.740989 3.802512

N -2.204834 0.970104 5.388745

H -1.975142 -1.306177 4.057254

C -2.287423 1.823805 6.505713

C -0.811963 1.090363 5.300056

O -0.020797 0.644520 4.511260

H -0.733076 3.842186 5.380538

H -0.394496 4.063564 7.107891

H 1.691734 4.368698 5.803037

C -0.199564 3.408739 6.241628

C 1.311374 3.349280 5.979240

C -0.762615 2.012626 6.553196

H 1.492766 2.773650 5.057154

H 1.952003 3.317928 8.047871

C 2.055732 2.688497 7.144476

H -0.182751 1.920861 8.638279

H 3.133764 2.630215 6.922203

C -0.009984 1.343644 7.719338
C 1.497775 1.290857 7.435571
H -0.415943 0.333369 7.890744
H 1.683427 0.635112 6.569278
H 2.014684 0.839513 8.298036
H -8.998301 5.066287 7.939811
H -7.032565 3.957466 9.043486
C -8.747965 4.020706 7.694221
H -9.357726 3.380840 8.354031
C -7.263735 3.763089 7.985294
N -4.504593 1.404802 7.066731
H -6.644229 4.458623 7.391752
N -3.339024 2.031140 7.252618
C -5.772205 -1.586328 11.655885
H -6.414657 -2.050606 12.409095
C -6.325481 -0.669393 10.760016
C -4.411698 -1.909884 11.600437
H -7.384390 -0.413710 10.797056
C -9.091384 3.714022 6.232664
H -10.166655 3.876510 6.049230
H -3.984936 -2.625037 12.308001
O -8.415925 0.868733 8.999697
C -5.508235 -0.066151 9.786884
C -7.340828 1.260721 8.598370
C -3.605528 -1.313820 10.624784
N -6.051509 0.870507 8.899093
C -6.861895 2.333453 7.609802
C -4.144213 -0.401332 9.715270
C -5.406167 1.887304 8.042583
H -8.547610 4.419622 5.576918
H -2.543742 -1.567371 10.561137
H -3.521324 0.030862 8.934280
H -2.594196 4.747601 7.073451

C -3.476310 3.157630 8.275634
H -4.318524 4.418161 6.725217
C -8.695762 2.280668 5.864119
C -3.561781 4.482806 7.520358
H -9.302773 1.571914 6.452474
H -2.313377 2.145869 9.797280
C -7.211494 2.025270 6.149729
O -4.707777 2.824990 8.853490
C -2.419627 3.149992 9.365326
H -6.590155 2.676725 5.510862
H -1.450561 3.509347 8.999674
H -8.909621 2.080169 4.800851
H -3.857601 5.272740 8.225901
H -6.928450 0.991514 5.902069
H -2.758687 3.835150 10.155731

Compound $TS2_{sCy}$

74

Potential Energy = -1646.657574542500Hrt, B3LYP+D3/def2-SVP
H -6.546628 -2.244702 2.427898
H -7.085745 -4.030448 4.087704
C -5.911915 -2.390077 3.305449
C -6.212610 -3.390097 4.235996
C -4.805462 -1.561011 3.502797
H -4.571626 -0.764723 2.793569
C -5.391505 -3.572872 5.355015
H -5.621451 -4.355739 6.081980
C -3.996120 -1.742898 4.631525
C -4.273708 -2.759757 5.552244
N -2.886914 -0.869253 4.805868
H -3.637479 -2.882161 6.427361
C -2.497666 0.075129 5.773248

C -1.862692 -0.508846 3.916773
O -1.576603 -0.923192 2.826424
H -2.585962 2.075628 3.868896
H -1.440373 2.751562 5.034717
H -0.668167 3.246980 2.735061
C -1.556626 1.987803 4.253930
C -0.529518 2.231593 3.140728
C -1.355145 0.595811 4.884210
H -0.712609 1.527409 2.312936
H 1.119044 2.825552 4.414202
C 0.902483 2.049885 3.656338
H 0.245356 1.113591 6.224771
H 1.622937 2.201072 2.835925
C 0.076934 0.404173 5.397231
C 1.097766 0.664199 4.281302
H 0.193392 -0.609592 5.812977
H 0.986150 -0.108332 3.502353
H 2.117175 0.563238 4.687898
H -7.328632 4.221115 7.266070
H -5.794069 2.815558 8.676967
C -7.352478 3.123001 7.167786
H -8.157237 2.759763 7.829243
C -6.014298 2.537692 7.637606
N -3.889932 -0.549750 7.496413
H -5.194179 2.943678 7.023568
N -2.977902 0.272968 6.945188
C -6.400298 -3.247542 11.313199
H -7.233950 -3.890921 11.606772
C -6.604870 -2.258606 10.347478
C -5.141213 -3.420647 11.898167
H -7.583631 -2.113372 9.889256
C -7.664099 2.719856 5.722382
H -8.638870 3.129694 5.409636

H -4.986782 -4.200384 12.648216
O -8.004508 0.213513 8.870079
C -5.535063 -1.431965 9.973786
C -6.844339 0.264473 8.542811
C -4.082399 -2.584475 11.523373
N -5.724814 -0.429752 9.004846
C -6.003740 1.016064 7.489797
C -4.274136 -1.584787 10.569020
C -4.834155 0.166076 8.088614
H -6.904326 3.161450 5.050488
H -3.099048 -2.704573 11.985709
H -3.473767 -0.902039 10.287995
H -1.303547 2.756741 7.046566
C -2.589455 1.454422 8.243911
H -3.062664 3.057776 6.850831
C -7.651927 1.196061 5.563388
C -2.273441 2.775831 7.560531
H -8.479710 0.762337 6.149869
H -1.153842 1.352072 9.834538
C -6.330016 0.592236 6.047838
O -3.704203 1.421892 8.926700
C -1.396073 0.785152 8.920601
H -5.500177 0.935733 5.405447
H -1.664163 -0.239898 9.212751
H -7.823788 0.911655 4.511767
H -2.224422 3.550504 8.342184
H -6.348839 -0.505620 5.970342
H -0.504062 0.757010 8.278690

Compound **[Ph-6-sCy*N]$_2$**

64

Potential Energy = -1453.660652992700 Hrt, B3LYP+D3/def2-SVP

H -3.593273 -1.352866 13.908374
H -1.642278 -2.224825 15.212011
C -2.601727 -1.404890 13.451500
C -1.510868 -1.890469 14.179934
C -2.442532 -0.976985 12.131721
H -3.285268 -0.595227 11.558371
C -0.247899 -1.945017 13.578057
H 0.610824 -2.322440 14.139401
C -1.171127 -1.033727 11.538965
C -0.067090 -1.519175 12.261069
N -0.996773 -0.605196 10.209126
H 0.912921 -1.559794 11.786094
C -1.854088 -0.029366 9.260365
C 0.102000 -0.615798 9.323189
O 1.236898 -0.988052 9.466857
H -1.263720 -1.903980 7.317139
H -1.833336 -0.484942 6.419565
H 0.066097 -1.571414 5.207939
C -0.977538 -0.893583 6.981638
C 0.269751 -0.944817 6.091751
C -0.754502 0.017263 8.198762
H 1.091254 -1.431532 6.645290
H -0.080173 0.906144 5.027544
C 0.706175 0.461172 5.664831
H -1.145905 1.889583 7.237039
H 1.619472 0.407024 5.049663
C -0.304675 1.424913 7.777271
C 0.938393 1.363066 6.881825
H -0.115550 2.040293 8.672111
H 1.787474 0.973963 7.469597
H 1.212535 2.381833 6.561852
H -6.411511 4.646107 5.490525
H -5.400600 3.769160 7.622461

C -6.650493 3.626724 5.835635
H -7.582812 3.699148 6.421622
C -5.526906 3.115015 6.744161
N -3.613854 0.821712 8.208328
H -4.566503 3.116856 6.199955
N -3.082385 0.284180 9.382303
C -7.139051 -0.114331 12.496360
H -7.966973 -0.721308 12.871276
C -7.011796 0.103274 11.121661
C -6.209745 0.433542 13.386541
H -7.731379 -0.321020 10.419708
C -6.871830 2.692348 4.641289
H -7.706063 3.058667 4.020461
H -6.307640 0.256289 14.460476
O -8.022138 1.759032 8.478732
C -5.948591 0.878268 10.639458
C -6.844445 1.566875 8.336497
C -5.154904 1.213507 12.897409
N -5.859759 1.127976 9.247619
C -5.795479 1.672777 7.202833
C -5.025365 1.446332 11.527818
C -4.839812 1.151576 8.270999
H -5.972095 2.702461 3.998491
H -4.424893 1.643316 13.587467
H -4.204964 2.049608 11.138840
C -7.146962 1.256771 5.102280
H -8.101760 1.227900 5.654892
C -6.027317 0.735585 6.010644
H -5.078930 0.671650 5.449166
H -7.261474 0.587108 4.234155
H -6.254347 -0.280865 6.372108

Compound **Ph-10-Me$_2$**

22

Potential Energy = -442.138880483400 Hrt, B3LYP+D3/def2-SVP

H -1.272776 -0.171575 7.091121

H -2.252578 0.858886 9.142390

C -0.830817 0.735481 7.512320

C -1.381217 1.313647 8.664359

C 0.280703 1.311837 6.897840

H 0.714600 0.866374 5.998824

C -0.811438 2.473583 9.199092

H -1.235978 2.929008 10.097552

C 0.853530 2.478038 7.434364

C 0.302257 3.055070 8.586664

N 1.982103 3.119013 6.859722

H 0.762874 3.961341 8.985958

C 2.581555 2.726530 5.856024

C 3.286996 2.392322 4.793622

H 4.673104 1.161260 5.943426

C 4.494332 1.486415 4.908224

H 2.022754 3.541142 3.437043

C 2.918393 2.903393 3.417216

H 4.365148 0.586971 4.280435

H 2.728352 2.060885 2.728799

H 5.400643 2.005914 4.548552

H 3.750688 3.490546 2.989226

Compound CO

2

Potential Energy = -113.229581903400 Hrt, B3LYP+D3/def2-SVP

C 3.177683 4.056551 6.573062

O 2.748746 5.089559 6.410858

Compound $TS3_{Me2}$

24

Potential Energy = -555.318365457300 Hrt, B3LYP+D3/def2-SVP

H -1.774550 -0.606796 6.547284

H -2.761500 0.478758 8.566551

C -1.293121 0.270476 6.987614

C -1.847565 0.879891 8.121317

C -0.127058 0.775748 6.413806

H 0.317444 0.314083 5.529852

C -1.227351 2.001958 8.680467

H -1.654741 2.480989 9.565055

C 0.494062 1.904564 6.977728

C -0.058992 2.515887 8.112105

N 1.674848 2.450237 6.445495

H 0.441728 3.389243 8.535360

C 2.367704 2.158502 5.438659

C 3.101321 4.015687 6.566774

C 3.614297 2.888953 5.323433

O 2.826605 5.134069 6.418903

H 4.609064 1.617569 6.782804

C 4.819393 2.091277 5.812787

H 2.976029 4.229168 3.727577

C 3.873294 3.680380 4.049172

H 5.073404 1.307236 5.083210

H 4.182090 3.000150 3.240611

H 5.689203 2.757735 5.920383

H 4.687722 4.401907 4.216372

Compound $TS3_{Cl2}$

18

Potential Energy = -1395.663316676100 Hrt, B3LYP+D3/def2-SVP

H -1.622591 -0.774897 6.590361
H -2.625263 0.277110 8.618004
C -1.184565 0.135154 7.008025
C -1.747450 0.726626 8.147298
C -0.064831 0.700938 6.400876
H 0.386715 0.256169 5.512065
C -1.184932 1.890475 8.682079
H -1.620937 2.352634 9.571026
C 0.494772 1.872395 6.941388
C -0.062916 2.466535 8.082853
N 1.624142 2.478645 6.372723
H 0.392955 3.373498 8.484928
C 2.292488 2.281300 5.331811
C 2.926755 4.060041 6.740016
C 3.484021 3.106906 5.271813
O 2.676809 5.184211 6.808859
Cl 4.979626 2.239868 5.630854
Cl 3.641695 4.195617 3.896372

Compound **Ph-10-Cl_2**

16
Potential Energy = -1282.485901783900 Hrt, B3LYP+D3/def2-SVP
H -1.211796 -0.593033 6.163245
H -2.720772 0.121511 8.016341
C -0.963664 0.286902 6.762175
C -1.811313 0.689221 7.804104
C 0.197485 1.005983 6.485508
H 0.864540 0.700293 5.675407
C -1.493682 1.814999 8.570968
H -2.153450 2.129566 9.383262
C 0.514902 2.137460 7.257522
C -0.331012 2.540434 8.300115

N 1.676026 2.910739 7.029620
H -0.059916 3.422458 8.883978
C 2.527593 2.734059 6.166015
C 3.498375 2.641194 5.280497
Cl 4.928815 1.693430 5.594821
Cl 3.397940 3.441906 3.733845

Compound $TS3_{Ph2}$

38
Potential Energy = -938.576322134600 Hrt, B3LYP+D3/def2-SVP
H -1.305363 -0.895591 7.068213
H -2.374802 0.487564 8.849293
C -0.908148 0.095215 7.304268
C -1.508490 0.871504 8.304711
C 0.198006 0.577014 6.605209
H 0.678095 -0.013629 5.822536
C -0.995239 2.137651 8.604571
H -1.458813 2.746868 9.384682
C 0.709173 1.850883 6.908492
C 0.112509 2.629837 7.910259
N 1.827370 2.379023 6.240362
H 0.527019 3.615360 8.132588
C 2.500516 1.989831 5.247625
C 3.048990 3.970708 6.279404
C 3.687861 2.800085 5.023410
O 2.716048 5.075176 6.169863
C 3.818591 3.552221 3.729396
C 2.669335 3.849401 2.973933
C 2.762518 4.609727 1.809221
H 1.697576 3.472571 3.301793
H 1.861368 4.826836 1.229906
C 4.006693 5.090307 1.380599

C 5.061688 4.034214 3.290411
C 5.153630 4.800831 2.124874
H 4.080014 5.685511 0.466694
H 5.963247 3.809779 3.862595
H 6.128736 5.169419 1.795987
C 5.827356 2.796685 6.391853
C 6.995609 2.167932 6.832616
H 5.602904 3.815489 6.715122
H 7.675342 2.700681 7.502800
C 4.941582 2.129318 5.533080
C 7.290721 0.863411 6.424204
C 5.244984 0.821073 5.123722
C 6.410318 0.191677 5.569651
H 8.202280 0.371290 6.773075
H 4.558699 0.300046 4.452955
H 6.630207 -0.829685 5.247834

Compound **Ph-10-Ph$_2$**

36
Potential Energy = -825.408660490400 Hrt, B3LYP+D3/def2-SVP
H -0.752344 -0.522412 6.585759
H -2.358736 0.360080 8.278804
C -0.567897 0.443043 7.064169
C -1.469753 0.938802 8.015630
C 0.568198 1.176518 6.723511
H 1.278187 0.796571 5.984387
C -1.229222 2.172981 8.627926
H -1.929665 2.562532 9.371003
C 0.806519 2.417333 7.338752
C -0.091771 2.912509 8.292689
N 1.942610 3.207289 7.033200
H 0.117647 3.877808 8.758384

C 2.797759 2.985370 6.188556
C 3.773854 2.834117 5.294858
C 3.572324 3.376490 3.923965
C 2.289800 3.412203 3.343404
C 2.093088 3.959922 2.074153
H 1.441431 2.993619 3.891609
H 1.088705 3.975470 1.642431
C 3.176251 4.471770 1.351276
C 4.653454 3.899857 3.189784
C 4.456176 4.437252 1.915726
H 3.024687 4.892449 0.353899
H 5.654234 3.893936 3.626207
H 5.309622 4.839187 1.362837
C 5.482918 2.169215 7.022028
C 6.625318 1.464768 7.406351
H 4.947222 2.777544 7.755623
H 6.972263 1.521968 8.441692
C 5.014165 2.116443 5.695312
C 7.331764 0.700587 6.470739
C 5.728962 1.339101 4.764256
C 6.877332 0.643111 5.148625
H 8.230664 0.155177 6.769465
H 5.375670 1.274247 3.733301
H 7.417491 0.045565 4.409129

Compound $TS3_{sCy}$

31
Potential Energy = -671.995898899100 Hrt, B3LYP+D3/def2-SVP
H -2.094778 -0.401900 5.884610
H -3.123708 0.689510 7.879384
C -1.605405 0.456885 6.351829
C -2.183544 1.069577 7.471862

C -0.405654 0.935116 5.826158
H 0.057939 0.470159 4.953788
C -1.553176 2.167882 8.065895
H -1.998869 2.649381 8.940055
C 0.225792 2.039968 6.425068
C -0.351044 2.654270 7.546139
N 1.441133 2.556230 5.945559
H 0.158899 3.507952 7.997553
C 2.151856 2.271555 4.949272
C 2.923566 4.058377 6.162200
O 2.675239 5.185062 6.027858
H 4.285790 1.592665 6.354896
H 4.747482 1.253720 4.675459
H 6.691712 2.194338 5.882257
C 4.586184 2.064025 5.406166
C 5.878821 2.871285 5.572799
C 3.433785 2.933589 4.891212
H 5.748768 3.602644 6.390224
H 6.476925 2.872998 3.492599
C 6.252200 3.611080 4.284537
H 3.915764 3.020886 2.816464
H 7.171656 4.200201 4.435628
C 3.801642 3.740628 3.644713
C 5.110092 4.521500 3.822875
H 2.970662 4.412081 3.377592
H 4.957736 5.324750 4.563206
H 5.367267 5.016332 2.872342

Compound **Ph-10-sCy**

29

Potential Energy = -558.816009447000 Hrt, B3LYP+D3/def2-SVP

H -1.385401 -0.487377 5.316618

H -3.022069 0.164854 7.084239

C -1.181319 0.372526 5.960412

C -2.100988 0.738382 6.952653

C -0.004037 1.100566 5.787897

H 0.716764 0.820144 5.015423

C -1.836421 1.840833 7.771692

H -2.550116 2.132452 8.546614

C 0.263164 2.208020 6.611501

C -0.659732 2.575030 7.600975

N 1.436037 2.996034 6.491798

H -0.433187 3.440834 8.227077

C 2.374005 2.761461 5.725906

H 4.535943 1.462267 6.426324

H 4.779102 0.945140 4.745006

H 6.805574 2.133173 5.521974

C 4.654979 1.831086 5.396425

C 5.904849 2.717987 5.269534

C 3.420910 2.594559 4.946772

H 5.837083 3.538088 6.006116

H 6.190292 2.488243 3.136634

C 6.030294 3.309491 3.859945

H 3.571610 2.412903 2.812775

H 6.919261 3.959196 3.798054

C 3.511597 3.221738 3.565889

C 4.774152 4.093039 3.459460

H 2.607985 3.807620 3.339628

H 4.660682 4.967283 4.124597

H 4.875478 4.481458 2.431881

Compound $TS4_{Me2}$

48

Potential Energy = -1110.726623993700 Hrt, B3LYP+D3/def2-SVP

C 3.911367 -1.458255 4.978779

C 2.663770 -1.911918 4.540934

C 4.587162 -0.457121 4.276500

C 0.150976 -1.524496 1.799833

C 2.103867 -1.353835 3.385192

C 4.012808 0.095803 3.126085

C -1.017471 -2.446883 2.260126

C 2.773245 -0.351042 2.673094

C -0.166560 -2.658364 3.525515

H 4.354552 -1.892508 5.878483

H 2.122936 -2.682698 5.089858

H 5.558761 -0.101369 4.627392

H 4.531547 0.887000 2.580410

H 2.308303 0.072937 1.782689

C -2.346161 -1.731513 2.501396

C -1.177819 -3.697761 1.394137

N 0.832927 -1.799817 2.931879

O -0.213831 -3.215049 4.580411

H -2.199803 -0.791228 3.053211

H -1.871361 -4.406903 1.873766

H -0.216820 -4.209774 1.229023

H -1.591826 -3.414899 0.413375

H -3.028904 -2.386824 3.065805

H -2.817259 -1.489477 1.535734

H -0.643225 0.491236 0.287660

H -1.399976 1.798917 -0.688405

C -1.443356 1.245242 0.263302

H 4.082435 3.927345 4.606498

C 3.249342 3.226693 4.704407

O 0.770809 3.622503 0.962404

H 2.312178 3.852523 2.849354

C 2.259160 3.195527 3.717529

H -2.407430 0.715356 0.312427
C 3.178265 2.370519 5.805843
C 0.039394 2.905314 1.575700
H 3.956618 2.396993 6.572288
C 1.192962 2.298269 3.851947
N 0.175862 2.255908 2.859977
C -1.330102 2.217037 1.436750
C 2.109701 1.473433 5.923389
C 1.113223 1.433802 4.950745
C -0.985634 1.568361 2.810947
H 2.056244 0.792956 6.776054
H 0.278423 0.735768 5.019603
H -2.601629 3.756146 0.579311
C -2.519478 3.177230 1.513106
H -3.448296 2.600928 1.649177
H -2.425502 3.883715 2.352854

Compound [**Ph-6-Me$_2$**]$_2$

48

Potential Energy = -1110.887075211700 Hrt, B3LYP+D3/def2-SVP
C 3.565493 -2.420051 4.852605
C 2.224254 -2.348399 4.469792
C 4.522125 -1.598812 4.245958
C -0.310397 -0.393069 2.426167
C 1.840708 -1.449772 3.463807
C 4.133118 -0.715649 3.232225
C -1.352376 -1.457521 2.028678
C 2.799112 -0.646427 2.829818
C -0.415646 -2.421687 2.801089
H 3.862194 -3.117798 5.640022
H 1.470616 -2.977761 4.944356
H 5.567626 -1.648148 4.559810

H 4.873326 -0.072553 2.749753
H 2.494508 0.035107 2.034735
C -2.744807 -1.313202 2.638270
C -1.401128 -1.810915 0.539604
N 0.487267 -1.388147 3.071999
O -0.394731 -3.596384 3.062291
H -2.691673 -1.029006 3.700362
H -1.899218 -2.784200 0.405691
H -0.390397 -1.874118 0.108081
H -1.971358 -1.053127 -0.018336
H -3.284747 -2.269898 2.558429
H -3.325651 -0.547870 2.101966
H 0.013301 1.557269 -0.338984
H -1.250972 2.815449 -0.458500
C -0.985812 1.855890 0.013175
H 4.332020 3.655143 4.940515
C 3.489918 2.959050 4.974007
O 0.299758 4.141217 2.025585
H 2.759362 3.519305 3.009484
C 2.614940 2.889079 3.887671
H -1.712352 1.100179 -0.321494
C 3.302338 2.137435 6.090852
C 0.052738 2.965786 2.102887
H 3.998822 2.185188 6.931466
C 1.537382 1.992493 3.925992
N 0.650734 1.931485 2.830352
C -1.019956 2.001988 1.532804
C 2.214693 1.257653 6.128010
C 1.324587 1.189666 5.055710
C -0.254464 0.937741 2.343483
H 2.056675 0.615346 6.997902
H 0.473306 0.508734 5.085628
H -2.732117 3.325114 1.639326

C -2.415262 2.355344 2.054556
H -3.146099 1.593043 1.745806
H -2.427359 2.425527 3.153052

Compound TS4$_{Cl2}$

36
Potential Energy = -2791.424878139800 Hrt, B3LYP+D3/def2-SVP
C -3.886215 1.626170 -1.672803
C -2.637095 1.862291 -1.093862
C 0.016040 2.044446 0.480986
C 1.334196 1.241025 0.552956
C -5.516342 -2.311999 0.773262
C -4.216503 0.354382 -2.150506
C -1.722849 0.805564 -1.004471
C -4.199941 -2.686649 1.057164
C -5.898198 -0.968887 0.837754
C 0.590732 0.164682 -0.289345
C -3.289766 -0.690615 -2.057877
C -2.036868 -0.470831 -1.488867
C -0.819512 -1.295348 1.766161
C -3.275806 -1.695481 1.407166
C -4.961161 0.009627 1.192486
C -0.007919 -2.553493 2.180221
C -3.647198 -0.347572 1.485555
C -1.335989 -3.330127 2.023390
H -4.608562 2.443010 -1.741988
H -2.377871 2.846404 -0.703005
H -6.245324 -3.078425 0.499513
H -5.200930 0.173456 -2.588296
H -3.893157 -3.731579 1.010015
H -6.928210 -0.682077 0.612220
H -3.548736 -1.687410 -2.422127

H -1.302293 -1.271728 -1.396084

H -5.255214 1.060108 1.241489

H -2.906637 0.400396 1.769972

Cl 1.875195 0.763815 2.164627

Cl 2.651204 1.991059 -0.396857

Cl 1.268514 -3.052784 1.064285

Cl 0.561186 -2.444220 3.863698

N -0.464610 1.008374 -0.385746

N -1.931879 -2.057471 1.690859

O -0.483282 3.019198 0.942797

O -1.761810 -4.435159 2.090452

Compound [**Ph-6-Cl$_2$**]$_2$

36

Potential Energy = -2791.584136591700 Hrt, B3LYP+D3/def2-SVP

C -3.251280 2.613916 -2.316264

C -2.130575 2.698275 -1.486896

C 0.783925 2.487092 -0.367196

C 1.594092 1.527676 0.568386

C -4.903154 -1.464086 0.770283

C -3.687426 1.373518 -2.794197

C -1.443394 1.526618 -1.145958

C -3.556777 -1.796556 0.938071

C -5.325289 -0.135413 0.886756

C 0.320504 0.692151 0.574005

C -2.983849 0.211216 -2.458238

C -1.853679 0.282774 -1.643695

C -0.065903 -0.382016 1.263891

C -2.634673 -0.784016 1.232059

C -4.396784 0.865354 1.194671

C 0.687127 -1.420819 2.084814

C -3.051897 0.545309 1.380031

C -0.691772 -2.161447 2.138168
H -3.792232 3.525488 -2.581965
H -1.789111 3.659264 -1.100024
H -5.625123 -2.250845 0.538005
H -4.572776 1.312177 -3.431486
H -3.216666 -2.828247 0.839775
H -6.377559 0.120083 0.741172
H -3.314510 -0.760313 -2.833010
H -1.295237 -0.619530 -1.390112
H -4.719167 1.904556 1.293266
H -2.328515 1.321667 1.633259
Cl 2.071467 2.240742 2.124615
Cl 2.986496 0.799101 -0.275136
Cl 1.971143 -2.308198 1.235442
Cl 1.216686 -0.885693 3.701479
N -0.303765 1.611756 -0.313297
N -1.273204 -1.118098 1.412713
O 0.978568 3.519476 -0.935344
O -1.121118 -3.171635 2.608209

Compound $TS4_{Ph2}$

76
Potential Energy = -1877.248091227300 Hrt, B3LYP+D3/def2-SVP
C 3.188383 -3.986498 -2.595846
C 2.852125 -5.342064 -2.487637
C 2.640279 -3.054086 -1.708968
C -3.952125 -0.047578 -0.598432
C -2.592540 -0.367830 -0.572129
C 1.964999 -5.759989 -1.491350
C -4.894095 -0.890053 0.000452
C 0.370602 -1.327805 -0.405970
C 1.743434 -3.471619 -0.708915

C -2.189846 -1.548778 0.061853
C 1.410744 -4.828384 -0.607241
C -4.475391 -2.069858 0.627699
C 1.168399 -2.463364 0.285412
C -3.123714 -2.407915 0.654921
C -0.233118 -2.862862 0.909753
C 2.182858 -2.055625 1.327073
C 2.670828 -3.035265 2.205279
C 2.625396 -0.736183 1.461139
C 3.574694 -2.695982 3.210387
C 3.534538 -0.395023 2.470828
C 4.007918 -1.369732 3.350672
H 3.881342 -3.654293 -3.374292
H 3.280133 -6.070377 -3.181295
H 2.905076 -1.996329 -1.789627
H -4.274402 0.877355 -1.082891
H -1.848969 0.291174 -1.017935
H -5.954524 -0.626835 -0.020130
H 1.695191 -6.815686 -1.400817
H -5.205593 -2.732140 1.099702
H 0.709633 -5.146661 0.166953
H 2.329657 -4.067800 2.103370
H -2.771639 -3.323147 1.133325
H 2.269090 0.032401 0.773701
H 3.944965 -3.466885 3.891583
H 3.874843 0.639672 2.562352
H 4.718358 -1.102804 4.137501
N -0.821121 -1.890476 0.152994
O 0.561218 -0.357852 -1.081163
C -0.959630 -4.386083 7.841796
C 0.389232 -4.099738 8.089405
C -1.503670 -4.159979 6.573744
C -0.941766 1.737245 1.833956

C -0.754553 0.473171 2.390969
C 1.188997 -3.584791 7.065071
C -2.218758 2.310092 1.799426
C -2.478314 -2.439569 4.135295
C -0.701773 -3.638185 5.542641
C -1.858431 -0.221287 2.901442
C 0.645081 -3.351376 5.797458
C -3.309992 1.611451 2.325473
C -1.284950 -3.429570 4.146649
C -3.140244 0.340796 2.880071
C -0.560056 -2.334000 3.257798
C -1.478636 -4.738060 3.416078
C -0.344998 -5.504084 3.104521
C -2.739995 -5.189925 3.017183
C -0.471372 -6.694491 2.391396
C -2.867257 -6.386400 2.299345
C -1.736270 -7.139214 1.980383
H -1.589923 -4.788333 8.640122
H 0.813958 -4.276703 9.080913
H -2.554470 -4.387763 6.375923
H -0.084605 2.277528 1.424368
H 0.232129 0.008914 2.433833
H -2.362867 3.300552 1.360769
H 2.242197 -3.357087 7.250760
H -4.309029 2.053023 2.295241
H 1.262329 -2.942213 4.995114
H 0.642101 -5.155185 3.415434
H -3.987376 -0.219615 3.272547
H -3.631791 -4.614033 3.268603
H 0.419229 -7.281196 2.150758
H -3.859553 -6.729197 1.995299
H -1.836908 -8.073304 1.421432
N -1.651246 -1.527335 3.402319

O -3.610039 -2.371488 4.518220

Compound [**Ph-6-Ph$_2$]$_2$**

76

Potential Energy = -1877.415125001200 Hrt, B3LYP+D3/def2-SVP

C 0.718483 -4.908147 -1.721010

C 1.412559 -5.992092 -1.174290

C 0.654507 -3.694865 -1.032291

C -2.709013 1.629097 -0.847314

C -1.574025 1.002242 -0.328884

C 2.047995 -5.848076 0.061596

C -3.936942 0.958947 -0.880236

C 0.757873 -1.030092 0.101284

C 1.281035 -3.544609 0.215385

C -1.672056 -0.310611 0.151438

C 1.985975 -4.632288 0.747006

C -4.020918 -0.357625 -0.413411

C 1.116861 -2.254331 1.008776

C -2.890037 -1.001872 0.089685

C -0.318364 -2.047434 1.549077

C 2.266935 -1.904225 1.937112

C 2.058313 -1.383011 3.219944

C 3.581771 -2.004256 1.451975

C 3.141013 -1.003774 4.018086

C 4.663734 -1.623012 2.247767

C 4.447362 -1.126667 3.538513

H 0.222817 -5.005114 -2.690380

H 1.458286 -6.943562 -1.709993

H 0.104242 -2.862061 -1.476266

H -2.634244 2.654623 -1.217849

H -0.615183 1.519897 -0.286046

H -4.825827 1.460142 -1.270882

H 2.593524 -6.687621 0.500205
H -4.974422 -0.890700 -0.440785
H 2.490983 -4.525468 1.706359
H 1.045156 -1.284008 3.605156
H -2.950229 -2.030504 0.446960
H 3.756668 -2.384529 0.443585
H 2.956266 -0.619619 5.023707
H 5.680775 -1.711688 1.856728
H 5.294621 -0.834604 4.164511
N -0.518996 -0.947117 0.661568
O 1.301729 -0.396957 -0.760866
C 1.088594 -2.801829 6.419648
C 2.261327 -3.546699 6.260118
C 0.038753 -2.939597 5.509254
C -3.151424 1.330152 3.235954
C -2.407608 0.153871 3.136840
C 2.370114 -4.435584 5.187861
C -4.537117 1.277593 3.423911
C -2.344856 -3.409115 3.919064
C 0.145016 -3.820414 4.420949
C -3.061791 -1.084377 3.198761
C 1.317857 -4.573336 4.279167
C -5.177251 0.036938 3.519255
C -0.957211 -3.883960 3.371018
C -4.446766 -1.147555 3.403413
C -1.057998 -2.602558 2.511129
C -1.102199 -5.213932 2.651537
C -1.332747 -5.294981 1.272781
C -1.109602 -6.396742 3.409935
C -1.530498 -6.533165 0.655834
C -1.309822 -7.634043 2.794783
C -1.514945 -7.707616 1.411976
H 0.988173 -2.108161 7.258373

H 3.085594 -3.434819 6.968916
H -0.867965 -2.346803 5.651301
H -2.642162 2.294377 3.165222
H -1.326829 0.191618 2.996307
H -5.115898 2.201394 3.498222
H 3.280794 -5.024721 5.052474
H -6.258188 -0.011422 3.673768
H 1.406994 -5.273246 3.449233
H -1.341777 -4.387971 0.671531
H -4.938167 -2.119440 3.461659
H -0.955384 -6.345867 4.489664
H -1.687151 -6.573859 -0.424373
H -1.308105 -8.545123 3.398993
H -1.664564 -8.676827 0.929034
N -2.322281 -2.282867 3.092542
O -3.137735 -3.798179 4.731975

Compound $TS4_{sCy}$

62

Potential Energy = -1344.088698972200 Hrt, B3LYP+D3/def2-SVP
C -3.613551 -4.779586 2.881770
C -2.302605 -4.488311 2.499616
C -4.332665 -3.891731 3.687117
C 0.412047 -1.946041 2.898733
C -1.714624 -3.296575 2.939397
C -3.732363 -2.703951 4.120579
C 1.563442 -2.526028 2.035997
C -2.420277 -2.404122 3.756041
C 0.546031 -3.611486 1.645198
H -4.079190 -5.704090 2.532207
H -5.361842 -4.121952 3.973565
H -4.290530 -1.999101 4.741318

N -0.387608 -2.979914 2.549923
O 0.439914 -4.557352 0.922508
H -1.935499 -1.486672 4.090412
H -1.743265 -5.164703 1.854839
C 2.733079 -3.063346 2.880232
C 2.069260 -1.628652 0.901242
C 3.105430 -2.350234 0.032056
C 3.768771 -3.783459 2.007513
C 4.271041 -2.881569 0.874552
H 3.309871 -4.689547 1.575245
H 4.609275 -4.122261 2.635864
H 4.986150 -3.430147 0.239366
H 4.822727 -2.027528 1.308553
H 3.475027 -1.664934 -0.748612
H 2.618226 -3.192040 -0.490519
H 3.196594 -2.198748 3.386666
H 2.354050 -3.732998 3.669762
H 1.219095 -1.257660 0.309790
H 2.524056 -0.740322 1.374274
C -4.555224 -3.292314 0.026843
C -4.102376 -2.105901 0.607800
C -3.703159 -4.065305 -0.767196
C -1.103375 0.117045 0.857474
C -2.783032 -1.697918 0.378636
C -2.386434 -3.645200 -0.988230
C -1.597407 1.271401 1.768943
C -1.922776 -2.458049 -0.423068
C -2.890678 0.446679 1.887055
H -5.581512 -3.619926 0.209285
H -4.062426 -4.997628 -1.209888
H -1.710664 -4.249200 -1.598260
N -2.300984 -0.501696 0.970275
O -3.943695 0.459403 2.451998

H -0.901358 -2.114087 -0.585721
H -4.752791 -1.507950 1.244696
C -1.790445 2.591760 1.000499
C -0.806159 1.492934 3.062514
C -1.445099 2.575248 3.940446
C -2.429884 3.668528 1.885902
C -1.622193 3.888055 3.169266
H -3.456183 3.358265 2.149182
H -2.519425 4.608766 1.316857
H -2.112104 4.644413 3.804726
H -0.626916 4.291337 2.906580
H -0.826725 2.734055 4.839193
H -2.430921 2.222333 4.290758
H -0.793409 2.922337 0.660611
H -2.396218 2.417908 0.096130
H -0.693598 0.541610 3.603271
H 0.213135 1.798901 2.767021

Compound [**Ph-6-sCy]**$_2$

62
Potential Energy = -1344.245144934500 Hrt, B3LYP+D3/def2-SVP
C -3.756207 -4.973823 1.629814
C -2.723608 -4.090894 1.305950
C -4.745966 -4.604177 2.547014
C -1.461356 -0.526917 1.710261
C -2.679405 -2.827801 1.913902
C -4.685474 -3.347926 3.160574
C 0.068436 -0.682974 1.640565
C -3.650163 -2.463063 2.857828
C -0.258234 -2.188955 1.472778
H -3.791665 -5.956198 1.151750
H -5.559774 -5.293226 2.785687

H -5.450462 -3.051210 3.882440
N -1.629021 -1.942702 1.596445
O 0.359039 -3.214446 1.323614
H -3.598021 -1.487000 3.341570
H -1.953632 -4.368859 0.585564
C 0.834846 -0.406031 2.945405
C 0.778704 -0.050213 0.436975
C 2.234758 -0.524298 0.340835
C 2.292902 -0.871848 2.845573
C 2.997381 -0.240316 1.639739
H 2.314476 -1.969932 2.747161
H 2.824751 -0.623175 3.778833
H 4.032463 -0.612640 1.564867
H 3.064666 0.853365 1.789795
H 2.728112 -0.030805 -0.512726
H 2.249245 -1.608191 0.137506
H 0.804684 0.680684 3.132047
H 0.322048 -0.891620 3.791626
H 0.223254 -0.279124 -0.487118
H 0.758322 1.045102 0.560472
C -7.051879 -0.860226 0.862450
C -6.056582 -0.066744 1.436889
C -6.731903 -1.790866 -0.132326
C -2.308863 0.505317 1.692676
C -4.729698 -0.200792 1.003332
C -5.407822 -1.908033 -0.569572
C -2.197177 2.016796 1.962196
C -4.407580 -1.108112 -0.016093
C -3.741601 1.986731 1.834149
H -8.084873 -0.757047 1.204709
H -7.511852 -2.420444 -0.567425
H -5.147459 -2.628861 -1.348580
N -3.730547 0.612879 1.577249

O -4.645364 2.784207 1.875792
H -3.376785 -1.193061 -0.361756
H -6.296568 0.652615 2.220521
C -1.544487 2.864993 0.857858
C -1.687345 2.432932 3.348692
C -1.910836 3.930852 3.592913
C -1.771346 4.362122 1.103156
C -1.266685 4.778116 2.489485
H -2.849133 4.580083 1.024795
H -1.265948 4.946113 0.316378
H -1.467552 5.848038 2.664273
H -0.168622 4.652120 2.529564
H -1.506632 4.210020 4.580011
H -2.994590 4.133024 3.621408
H -0.462749 2.652453 0.861475
H -1.930275 2.559376 -0.128285
H -2.181179 1.827055 4.125758
H -0.609402 2.205083 3.399805

Compound $TS5_{Me2}$

34
Potential Energy = -748.388376114200 Hrt, B3LYP+D3/def2-SVP
O -2.551898 1.030384 1.052700
C -2.443438 -0.061022 0.585996
N -1.273011 -0.840717 0.260354
C -3.308496 -1.282057 0.220961
C -1.934938 -1.994027 0.044839
O -1.423408 -2.567683 -2.371079
C -1.463677 -3.408584 -1.445627
C -2.678989 -4.322628 -1.311826
C -0.182988 -4.032020 -0.902611
C 0.097459 -0.460120 0.203453

C -4.173687 -1.819428 1.357987

C -4.096020 -1.080721 -1.078737

H -3.598664 -1.931712 2.290355

H -4.582519 -2.803577 1.081257

H -5.014565 -1.134230 1.548415

H -2.511577 -5.190023 -1.974915

H -3.591548 -3.814181 -1.647047

H -2.807137 -4.692406 -0.284582

H -0.343563 -4.494177 0.081751

H 0.629059 -3.298612 -0.836764

H 0.125458 -4.820692 -1.612884

H -3.428900 -0.845785 -1.919854

H -4.822510 -0.263823 -0.943745

H -4.652638 -1.994612 -1.330970

C 0.833620 -0.719138 -0.959409

C 2.165664 -0.302694 -1.020681

H 0.346636 -1.238502 -1.788797

C 2.745974 0.375476 0.057589

H 2.751242 -0.503578 -1.921337

C 1.991725 0.644103 1.205363

H 3.787801 0.700957 0.001465

C 0.660003 0.229065 1.283907

H 2.441348 1.178483 2.045846

H 0.059511 0.434649 2.171827

Compound **Ph-11-Me$_2$**

34

Potential Energy = -748.464140487400 Hrt, B3LYP+D3/def2-SVP

O -1.955668 1.276963 -0.200328

C -1.986255 0.101639 0.072111

N -0.976742 -0.818922 0.312607

C -3.091777 -0.948011 0.311941

C -1.901630 -1.903510 0.599650
O -1.721418 -3.176354 0.042864
C -1.766392 -3.051790 1.505725
C -3.002040 -3.672244 2.118730
C -0.481772 -3.339350 2.246214
C 0.418098 -0.722268 0.135299
C -3.996699 -0.587778 1.487692
C -3.868333 -1.310608 -0.950953
H -3.417893 -0.456729 2.415433
H -4.762258 -1.358828 1.660526
H -4.512359 0.361184 1.270643
H -2.855748 -4.759896 2.222923
H -3.887884 -3.501715 1.493204
H -3.189129 -3.256191 3.121012
H -0.568385 -2.973946 3.282084
H 0.391225 -2.864218 1.785236
H -0.309499 -4.427445 2.286872
H -3.190773 -1.542959 -1.786654
H -4.517584 -0.473153 -1.250015
H -4.499579 -2.194402 -0.767204
C 1.150187 -1.800594 -0.389832
C 2.529273 -1.675274 -0.570028
H 0.629847 -2.721824 -0.652741
C 3.181735 -0.477310 -0.256946
H 3.096043 -2.519477 -0.971573
C 2.442989 0.600960 0.242003
H 4.260019 -0.382953 -0.407359
C 1.066129 0.483746 0.446274
H 2.942531 1.543141 0.482468
H 0.484099 1.320774 0.833403

Compound $TS5_{Cl2}$

28

Potential Energy = -1588.733950664900 Hrt, B3LYP+D3/def2-SVP

O -2.297907 1.032996 1.209399

C -2.281200 -0.009284 0.645939

N -1.217787 -0.793049 0.074042

C -3.229745 -1.182444 0.297473

C -1.937986 -1.910809 -0.156614

O -1.744019 -2.331898 -2.572534

C -1.759882 -3.210485 -1.669979

C -3.030121 -4.035478 -1.462948

C -0.487260 -3.977073 -1.319400

C 0.145210 -0.449124 -0.147947

Cl -4.005279 -1.957726 1.689822

Cl -4.380432 -0.773003 -0.988309

H -3.005573 -4.864433 -2.191551

H -3.922765 -3.429874 -1.664166

H -3.085409 -4.461740 -0.450814

H -0.580564 -4.497718 -0.355323

H 0.390090 -3.319094 -1.298130

H -0.328982 -4.731054 -2.110775

C 0.718449 -0.720158 -1.396363

C 2.047200 -0.350426 -1.615169

H 0.107836 -1.204063 -2.163846

C 2.780137 0.286773 -0.607172

H 2.510196 -0.556468 -2.583304

C 2.183823 0.566596 0.627966

H 3.819634 0.572589 -0.786453

C 0.856542 0.203713 0.865716

H 2.754315 1.069212 1.412558

H 0.379038 0.417931 1.823224

Compound **Ph-11-Cl$_2$**

28

Potential Energy = -1588.814560896300 Hrt, B3LYP+D3/def2-SVP

O -2.044854 1.482903 -0.253158

C -2.064122 0.300559 -0.053379

N -1.080092 -0.636470 0.198127

C -3.163120 -0.791235 0.057449

C -1.986578 -1.766139 0.329803

O -1.764940 -2.960103 -0.335959

C -1.927883 -2.994933 1.130173

C -3.210022 -3.666803 1.564805

C -0.703200 -3.353733 1.935476

C 0.326961 -0.512854 0.157181

Cl -4.268288 -0.505442 1.426893

Cl -4.035772 -1.122635 -1.446389

H -3.071703 -4.759834 1.538357

H -4.046788 -3.401870 0.905365

H -3.465278 -3.375089 2.594502

H -0.874968 -3.089884 2.990891

H 0.198370 -2.835170 1.591201

H -0.529366 -4.440506 1.881997

C 1.110892 -1.523640 -0.421027

C 2.498572 -1.374528 -0.462611

H 0.627187 -2.408627 -0.835304

C 3.103854 -0.217835 0.041753

H 3.109665 -2.164667 -0.906249

C 2.312406 0.794891 0.594005

H 4.189725 -0.103629 -0.001550

C 0.924618 0.651568 0.661094

H 2.777404 1.704005 0.983573

H 0.300927 1.436349 1.090921

Compound $TS5_{Ph2}$

48

Potential Energy = -1131.649845374200 Hrt, B3LYP+D3/def2-SVP

C -0.852872 -4.947638 -0.505359

C 0.005419 -5.857435 0.118106

C -0.773824 -3.585382 -0.199447

C -3.754045 2.188353 2.648378

C -2.729827 1.240613 2.586384

C 0.945152 -5.396891 1.047591

C -4.518292 2.485224 1.513937

C -0.534045 -0.665156 0.192174

C 0.169980 -3.115560 0.726146

C -2.493549 0.586911 1.370339

C 1.027496 -4.035993 1.348583

C -4.257429 1.834147 0.303024

C 0.202348 -1.653488 1.130313

C -3.239803 0.881169 0.221743

C -0.949571 -1.291593 2.116381

C 1.576886 -1.104780 1.483440

C 1.803791 -0.341270 2.637397

C 2.628182 -1.313274 0.574905

C 3.077326 0.185458 2.885575

C 3.893706 -0.780778 0.824866

C 4.123112 -0.031352 1.985390

H -1.588125 -5.296011 -1.235135

H -0.055412 -6.922558 -0.119766

H -1.451387 -2.888794 -0.698968

H -3.950665 2.703269 3.592076

H -2.112308 0.993975 3.456398

H -5.316096 3.230041 1.571398

H 1.619188 -6.101629 1.541246

H -4.848527 2.067894 -0.585734

H 1.761719 -3.681638 2.073815

H 0.999153 -0.162611 3.357751

H -3.023045 0.373498 -0.718731
H 2.451390 -1.898765 -0.330385
H 3.246313 0.769912 3.793740
H 4.704110 -0.950714 0.111247
H 5.115178 0.382608 2.184098
N -1.438721 -0.363004 1.279453
O -0.514850 -0.300055 -0.940043
O -0.810026 -0.289978 4.535241
H -2.893648 -2.703013 3.653013
C -2.664125 -1.801790 4.240452
C -1.184112 -1.432386 4.162410
H -3.304341 -0.973712 3.910187
H -2.898232 -2.006022 5.300794
H -0.538490 -3.501137 3.805197
C -0.257836 -2.623914 4.405039
H 0.788921 -2.360534 4.202839
H -0.339282 -2.887789 5.474108

Compound **Ph-11-Ph$_2$**

48

Potential Energy = -1131.726035671000 Hrt, B3LYP+D3/def2-SVP

C -0.939893 -5.034173 0.514114
C 0.218388 -5.809497 0.648014
C -0.895439 -3.661579 0.760812
C -3.829932 2.381364 2.172761
C -2.787181 1.460768 2.294427
C 1.415774 -5.196770 1.025236
C -4.565999 2.469513 0.985601
C -0.499971 -0.725656 0.387387
C 0.302996 -3.037917 1.148758
C -2.495169 0.600699 1.222976
C 1.458644 -3.819534 1.271092

C	-4.254951	1.624242	-0.084835
C	0.301879	-1.542732	1.442655
C	-3.227622	0.684693	0.028878
C	-0.844048	-1.087312	2.406892
C	1.676580	-0.957053	1.698817
C	2.190196	-0.807398	2.995294
C	2.485472	-0.619722	0.601087
C	3.490505	-0.329075	3.188779
C	3.783067	-0.141029	0.796710
C	4.291089	0.004160	2.092347
H	-1.881574	-5.501001	0.213382
H	0.185967	-6.884882	0.454848
H	-1.809020	-3.070229	0.656213
H	-4.059615	3.044831	3.010632
H	-2.195255	1.397940	3.207842
H	-5.375211	3.198178	0.893817
H	2.327090	-5.791468	1.129994
H	-4.820866	1.689966	-1.017752
H	2.398772	-3.352406	1.567178
H	1.566689	-1.048618	3.854941
H	-2.979455	0.022777	-0.801388
H	2.089992	-0.735817	-0.409783
H	3.877337	-0.215732	4.205062
H	4.399930	0.118957	-0.067615
H	5.306891	0.377843	2.246167
N	-1.437195	-0.327329	1.319578
O	-0.395157	-0.509765	-0.794394
O	-0.737635	-0.556699	3.692917
H	-3.480285	-2.588118	3.282772
C	-2.993122	-1.708259	3.732242
C	-1.502736	-1.800599	3.509922
H	-3.431913	-0.806576	3.291425
H	-3.211741	-1.715757	4.812373

H -1.286507 -3.933407 3.724150
C -0.843198 -3.011643 4.129112
H 0.236100 -3.038451 3.932801
H -1.004645 -2.998209 5.219554

Compound TS5$_{sCy}$

41
Potential Energy = -865.066932945300 Hrt, B3LYP+D3/def2-SVP
C -4.602364 -4.468508 2.063995
C -3.399148 -3.873265 1.675149
C -4.720435 -5.066005 3.323217
C -0.136529 -2.628523 2.864089
C -2.315952 -3.884354 2.561509
C -3.625638 -5.080728 4.196198
C 0.780444 -2.568302 1.609682
C -2.416715 -4.497448 3.815761
C -0.391499 -3.283101 0.910489
H -5.453469 -4.459393 1.378776
H -5.665273 -5.525497 3.623871
H -3.711424 -5.555941 5.176616
N -1.090876 -3.285715 2.169591
O -0.705499 -3.705666 -0.160946
H -1.548869 -4.511805 4.477249
H -3.296894 -3.401225 0.697014
C 2.021960 -3.463977 1.784975
C 1.157363 -1.210383 1.002676
C 1.916251 -1.395438 -0.317372
C 2.791343 -3.632339 0.469522
C 3.158093 -2.274631 -0.137924
H 2.168556 -4.197952 -0.245099
H 3.696497 -4.235836 0.648088
H 3.672224 -2.415374 -1.102968

H 3.873723 -1.760660 0.529984

H 2.198019 -0.407352 -0.717147

H 1.243976 -1.856634 -1.061663

H 2.665275 -2.982773 2.542007

H 1.727607 -4.444988 2.192815

H 0.254531 -0.598762 0.875469

H 1.792492 -0.677392 1.728813

H -1.864738 -0.589100 5.651387

H 0.755923 0.136368 5.342150

C -1.620691 -1.436115 4.984993

C 0.827721 -0.751040 4.687819

C -0.533934 -0.955239 4.031754

O -0.855126 -0.282634 3.028743

H -2.529880 -1.703590 4.430656

H 1.599838 -0.553492 3.932150

H -1.294000 -2.281391 5.605977

H 1.120564 -1.615459 5.300515

Compound **Ph-11-sCy**

41

Potential Energy = -865.142630744400 Hrt, B3LYP+D3/def2-SVP C -4.608446 -3.488906 3.515167

C -3.371877 -2.940748 3.168169

C -4.831684 -4.866311 3.406294

C -0.293166 -2.103925 2.771820

C -2.336369 -3.784876 2.732814

C -3.804313 -5.698235 2.948434

C 0.668299 -2.360256 1.581305

C -2.555749 -5.166611 2.617282

C -0.253342 -3.563111 1.296559

H -5.409108 -2.829697 3.860867

H -5.804172 -5.288309 3.671658

H -3.971575 -6.774376 2.853677
N -1.084306 -3.253766 2.362250
O -0.289477 -4.463011 0.489287
H -1.750357 -5.809296 2.260341
H -3.195423 -1.866487 3.227150
C 2.112569 -2.764481 1.904464
C 0.619495 -1.281624 0.492675
C 1.361693 -1.738021 -0.770198
C 2.838125 -3.238556 0.637993
C 2.800619 -2.161909 -0.452852
H 2.351223 -4.154711 0.265490
H 3.879838 -3.503159 0.883923
H 3.299343 -2.526691 -1.366115
H 3.373405 -1.278937 -0.112636
H 1.353464 -0.928544 -1.518611
H 0.820892 -2.591055 -1.213017
H 2.641919 -1.892321 2.320122
H 2.119236 -3.547365 2.680841
H -0.427061 -1.025712 0.261897
H 1.087851 -0.367844 0.898744
H -1.058240 -1.232868 6.003814
H 0.980800 0.019975 5.144459
C -0.812528 -2.064562 5.323246
C 1.194477 -0.788545 4.426202
C -0.082287 -1.536343 4.111116
O -0.902805 -0.881251 3.084830
H -1.738705 -2.591022 5.068201
H 1.632347 -0.341044 3.524703
H -0.156195 -2.762176 5.867872
H 1.934433 -1.463862 4.883376

Compound **MAC**(Singlet)

66

Free Energy = -1234.546532 Hrt, B3LYP+D3(BJ)/def2-TZVP

N	3.8199943	0.4229726	-0.7073279
C	4.3129572	-0.7762796	-0.1856774
C	3.0818914	-1.4838496	0.3466257
N	2.0300397	-0.5197553	0.0408922
C	2.4506977	0.5850111	-0.5712015
C	0.6703351	-0.7654996	0.4128536
C	4.6677315	1.4249391	-1.2856901
C	5.0077707	2.5422664	-0.5140791
C	5.8391099	3.4982898	-1.0941479
C	6.3112510	3.3393899	-2.3881160
C	5.9563877	2.2237008	-3.1317888
C	5.1249634	1.2430361	-2.5955789
C	4.5185058	2.6813129	0.9159956
H	6.1218644	4.3759540	-0.5296901
H	6.9589457	4.0915154	-2.8213448
H	6.3302658	2.1176849	-4.1404204
C	4.6697652	0.0509461	-3.4176213
C	4.2860334	4.1308637	1.3446482
H	5.2201066	4.6917840	1.4170351
H	3.8183358	4.1533587	2.3309233
H	3.6288381	4.6505389	0.6457655
C	5.4786406	1.9720794	1.8830481
H	3.5522603	2.1805988	0.9759157
H	5.6385528	0.9307747	1.6027766
H	5.0867654	2.0017281	2.9027835
H	6.4539945	2.4646110	1.8805456
C	3.3003748	0.3430360	-4.0501982
H	2.9283518	-0.5343562	-4.5849702
H	3.3796985	1.1670422	-4.7632824
H	2.5666264	0.6253099	-3.2948741
C	5.6807738	-0.3881513	-4.4776210

H	4.5480000	-0.7918305	-2.7357406
H	6.6647457	-0.5629824	-4.0399380
H	5.7858481	0.3534303	-5.2722240
H	5.3462530	-1.3163519	-4.9449502
C	-0.1665583	-1.4380054	-0.4848313
C	0.2306974	-0.3333998	1.6716036
C	-1.0913584	-0.5971806	2.0224605
C	-1.4818261	-1.6789818	-0.0904240
C	-1.9390874	-1.2663041	1.1513991
C	1.1386027	0.4644620	2.5898395
C	0.9891961	0.0897638	4.0665025
H	1.7395827	0.6143229	4.6611085
H	1.1193706	-0.9828689	4.2218621
H	0.0104576	0.3703614	4.4598802
C	0.9122539	1.9680596	2.3714067
H	2.1711205	0.2535755	2.3070334
H	-0.1098898	2.2432337	2.6416081
H	1.0726025	2.2357052	1.3266648
H	1.5969605	2.5528025	2.9900679
C	0.3079419	-1.8294378	-1.8715302
C	-0.2187652	-0.8266918	-2.9078693
H	-1.3104303	-0.8472021	-2.9433848
H	0.1593872	-1.0686779	-3.9033451
H	0.0945782	0.1869947	-2.6568750
C	-0.0643192	-3.2674215	-2.2449107
H	1.3960423	-1.7594360	-1.8871921
H	0.2988743	-3.9786390	-1.5004778
H	0.3737488	-3.5295768	-3.2100490
H	-1.1447477	-3.3965915	-2.3312605
H	-1.4660192	-0.2717918	2.9834780
H	-2.9639676	-1.4620687	1.4411446
H	-2.1575836	-2.1881857	-0.7647272
H	3.1717478	-1.6733290	1.4178906

H 2.9270907 -2.4385028 -0.1601027
O 5.4573618 -1.1517034 -0.1715932

Compound **MAC**(Triplet)

66
Free Energy = -1234.443864 Hrt, B3LYP+D3(BJ)/def2-TZVP
N 3.709358 0.562634 -0.632635
C 4.156451 -0.623463 -0.062273
C 3.045624 -1.085403 0.876317
N 1.938574 -0.158221 0.585777
C 2.444741 0.871847 -0.168613
C 0.581354 -0.609211 0.532373
C 4.572380 1.482660 -1.315505
C 5.423153 2.292371 -0.555725
C 6.278163 3.153439 -1.243081
C 6.274673 3.206188 -2.627069
C 5.410890 2.400657 -3.356308
C 4.541869 1.522111 -2.715206
C 5.455452 2.234703 0.959300
H 6.955000 3.787981 -0.686102
H 6.946854 3.880478 -3.143152
H 5.416620 2.456268 -4.435921
C 3.625588 0.599084 -3.494075
C 5.279814 3.619946 1.591558
H 6.114473 4.281709 1.352105
H 5.230759 3.532522 2.679036
H 4.360933 4.095765 1.244982
C 6.740424 1.552102 1.448325
H 4.610751 1.630288 1.290317
H 6.837050 0.554439 1.019933
H 6.734528 1.464919 2.537409
H 7.620357 2.133384 1.162929

C	3.130004 1.197389 -4.811482
H	2.379359 0.541592 -5.256219
H	3.936651 1.304836 -5.539442
H	2.676235 2.178350 -4.659791
C	4.307797 -0.757089 -3.730057
H	2.747759 0.425578 -2.872164
H	4.649690 -1.202765 -2.796274
H	5.179260 -0.634840 -4.377788
H	3.619794 -1.451730 -4.218041
C	0.099770 -1.315463 -0.579360
C	-0.233899 -0.332374 1.642897
C	-1.554194 -0.773257 1.616476
C	-1.228358 -1.741181 -0.559105
C	-2.048532 -1.472674 0.524269
C	0.308811 0.475597 2.807622
C	-0.326273 0.118092 4.152701
H	0.195771 0.637940 4.958208
H	-0.272823 -0.954276 4.349230
H	-1.374865 0.419143 4.201679
C	0.168268 1.978010 2.519317
H	1.376673 0.260642 2.879355
H	-0.884455 2.248673 2.407042
H	0.690528 2.253107 1.602361
H	0.586137 2.567177 3.338944
C	0.973123 -1.641605 -1.775309
C	0.393302 -1.059851 -3.069892
H	-0.577088 -1.501481 -3.304134
H	1.061030 -1.261750 -3.909159
H	0.260672 0.020034 -2.989938
C	1.203710 -3.152789 -1.907287
H	1.945526 -1.178570 -1.622242
H	1.635062 -3.570509 -0.995952
H	1.887907 -3.361733 -2.732041

H 0.267448 -3.679628 -2.102815
H -2.205037 -0.572239 2.456093
H -3.077070 -1.811577 0.520746
H -1.623205 -2.293767 -1.402168
H 3.400432 -1.023182 1.915369
H 2.747630 -2.117206 0.684417
O 5.238534 -1.146544 -0.235394

Compound **CAAC**(Singlet)

74
Free Energy = -1147.941544 Hrt, B3LYP+D3(BJ)/def2-TZVP
C -5.4377191 -3.4266879 1.9083129
C -4.0472365 -3.5117871 1.9741778
C -6.0627175 -2.3758103 1.2590343
C -1.0878464 -2.0292929 2.1704196
C -3.2905172 -2.5186461 1.3333755
C -5.2990337 -1.3604168 0.7060119
C 0.3471437 -2.2348084 1.7259306
C -3.9066103 -1.4023751 0.7417403
C 0.2944074 -3.2787909 0.5713895
H -6.0379844 -4.1858537 2.3924698
H -7.1436720 -2.3299883 1.2132296
H -5.7926346 -0.5113583 0.2525917
N -1.8495386 -2.6284113 1.2951100
C -3.1147443 -0.1970286 0.2695498
C -3.5906061 0.3640397 -1.0725260
H -2.0803119 -0.4999332 0.1487193
C -3.1264045 0.8807270 1.3640171
H -4.5929601 0.7907065 -1.0040923
H -3.6064803 -0.4055916 -1.8462018
H -2.9214243 1.1622062 -1.4008904
H -2.7128133 0.4806499 2.2901041

H	-4.1439767	1.2283850	1.5573387
H	-2.5248993	1.7402904	1.0579278
C	-3.4204544	-4.5841857	2.8463925
C	-3.6771321	-4.2520245	4.3244424
H	-3.1596240	-4.9638976	4.9710214
H	-4.7442545	-4.2984531	4.5521286
H	-3.3228793	-3.2499942	4.5651533
C	-3.9069401	-5.9996427	2.5204467
H	-2.3441665	-4.5543679	2.6975206
H	-3.7538659	-6.2486766	1.4703040
H	-4.9699281	-6.1169860	2.7393265
H	-3.3666259	-6.7305585	3.1260468
C	1.3562953	-2.5533893	2.8771663
C	0.7521474	-0.8393393	1.1622504
C	2.1782611	-0.7739098	0.6139971
C	2.7665735	-2.6166793	2.2651339
C	3.1723087	-1.2731720	1.6592961
H	2.8147797	-3.4001513	1.5020409
H	3.5008702	-2.8840055	3.0219294
H	4.1718116	-1.3409025	1.2191813
H	3.2368694	-0.5324656	2.4657180
H	2.2462163	-1.4400419	-0.2536532
C	2.5141268	0.6372163	0.1374644
H	0.0532896	-0.5450490	0.3760380
H	0.6341857	-0.1042303	1.9628811
C	-1.1765984	-3.3697914	0.1387326
C	-1.4441656	-2.6640435	-1.1926342
C	-1.6707996	-4.8083834	0.0246569
H	-1.0265057	-1.6597145	-1.2164187
H	-2.5130541	-2.6024071	-1.3986099
H	-0.9809688	-3.2367892	-1.9978342
H	-1.4850701	-5.3759556	0.9333938
H	-1.1407917	-5.3027358	-0.7915036

H -2.7375307 -4.8447142 -0.1984919
H 0.6476939 -4.2498783 0.8999748
H 0.9172408 -2.9914978 -0.2733719
H 1.8183172 0.9721213 -0.6356812
H 3.5242376 0.6833714 -0.2766305
H 2.4597458 1.3490762 0.9656614
C 1.0418703 -3.7153532 3.8829881
H 1.3370736 -1.6590679 3.5113012
C -0.1793782 -3.3467839 4.7362035
H 0.0228829 -2.4554369 5.3346556
H -0.4245412 -4.1654917 5.4177778
H -1.0503983 -3.1187868 4.1271729
C 0.7912504 -5.0808782 3.2299984
H -0.1441972 -5.1009057 2.6734571
H 0.7204852 -5.8483155 4.0047321
H 1.6005869 -5.3696689 2.5562819
C 2.2230525 -3.8799269 4.8595446
H 2.5384688 -2.9161355 5.2668286
H 3.0884837 -4.3543570 4.3959096
H 1.9165713 -4.5096497 5.6975348

Compound **CAAC**(Triplet)

74

Free Energy = -1147.866249 Hrt, B3LYP+D3(BJ)/def2-TZVP

C -5.494606 -3.332888 1.952924
C -4.105304 -3.451210 1.971371
C -6.117665 -2.280125 1.303443
C -1.064769 -2.372489 2.252731
C -3.337992 -2.489813 1.284209
C -5.351190 -1.296297 0.699607
C 0.360837 -2.280179 1.838405
C -3.960004 -1.371669 0.693836

C 0.288522 -3.286467 0.630935
H -6.096777 -4.071170 2.466899
H -7.198457 -2.210693 1.293194
H -5.840669 -0.447069 0.240531
N -1.920809 -2.613072 1.202858
C -3.152053 -0.202649 0.164956
C -3.601286 0.277103 -1.217061
H -2.121547 -0.533786 0.087208
C -3.172649 0.945884 1.184379
H -4.608393 0.698291 -1.193010
H -3.597730 -0.537727 -1.942666
H -2.930488 1.058675 -1.580818
H -2.796018 0.609316 2.151421
H -4.186994 1.325305 1.328876
H -2.545868 1.772865 0.841923
C -3.473858 -4.546552 2.810362
C -3.671116 -4.240364 4.302442
H -3.151213 -4.979636 4.915894
H -4.729828 -4.260193 4.570567
H -3.279028 -3.254252 4.553027
C -3.997876 -5.945491 2.471376
H -2.403127 -4.528223 2.626507
H -3.877901 -6.173064 1.411918
H -5.056919 -6.047571 2.716572
H -3.455060 -6.700052 3.045212
C 1.430849 -2.608879 2.924563
C 0.696075 -0.856520 1.304569
C 2.094382 -0.724279 0.694525
C 2.807043 -2.632726 2.236538
C 3.153692 -1.258109 1.658823
H 2.827602 -3.390624 1.447373
H 3.585593 -2.908617 2.944698
H 4.126548 -1.291122 1.159170

H 3.257100 -0.551400 2.491478
H 2.131805 -1.338570 -0.212148
C 2.381219 0.717563 0.281121
H -0.053654 -0.563959 0.567444
H 0.597646 -0.154450 2.138490
C -1.186547 -3.360783 0.131898
C -1.360806 -2.674326 -1.224776
C -1.652307 -4.811721 0.000623
H -0.950668 -1.666274 -1.224711
H -2.414523 -2.621464 -1.501662
H -0.840129 -3.250265 -1.992566
H -1.510212 -5.363837 0.926719
H -1.076907 -5.313241 -0.780612
H -2.706800 -4.858605 -0.275735
H 0.615766 -4.270266 0.946651
H 0.940359 -2.978080 -0.183905
H 1.640378 1.079288 -0.435981
H 3.367803 0.809261 -0.179812
H 2.355723 1.381469 1.149708
C 1.163587 -3.775306 3.936132
H 1.444930 -1.721046 3.569526
C 0.028741 -3.362240 4.886878
H 0.308862 -2.476471 5.462463
H -0.191190 -4.167237 5.592714
H -0.882868 -3.134354 4.334245
C 0.801279 -5.115773 3.285972
H -0.164959 -5.068871 2.786950
H 0.737182 -5.890879 4.053850
H 1.554382 -5.434789 2.562236
C 2.411512 -3.999380 4.809998
H 2.787652 -3.057689 5.217871
H 3.222093 -4.481719 4.262630
H 2.155669 -4.646453 5.651799

Compound **CArAmC**(Singlet)

43

Free Energy = -904.238344 Hrt, B3LYP+D3(BJ)/def2-TZVP

N	3.7096117	0.4357527	-0.5308405
C	3.9826737	-0.9867166	-0.2926750
C	2.7470463	-1.4669224	0.3641325
C	1.8920633	-0.3674750	0.4597116
C	2.5158913	0.8620461	-0.1194222
C	4.7000125	1.2620390	-1.1676535
C	5.6410571	1.9148417	-0.3657033
C	6.5770268	2.7264244	-1.0033965
C	6.5763332	2.8639346	-2.3832706
C	5.6450660	2.1853760	-3.1548436
C	4.6895314	1.3626034	-2.5621765
C	5.6087247	1.7865101	1.1458657
H	7.3169875	3.2533471	-0.4170133
H	7.3122062	3.4983477	-2.8615894
H	5.6650997	2.2943741	-4.2303547
C	3.6418591	0.6455930	-3.3932265
C	4.7205980	2.8840503	1.7506487
H	5.1356133	3.8709743	1.5321213
H	4.6590593	2.7730044	2.8359089
H	3.7119833	2.8415131	1.3370894
C	6.9999448	1.7850794	1.7834774
H	5.1504582	0.8253674	1.3876529
H	7.6451019	1.0319745	1.3282025
H	6.9181325	1.5660059	2.8498511
H	7.4909453	2.7556657	1.6900406
C	2.4094198	1.5432191	-3.5788224
H	1.6335386	1.0179537	-4.1409552
H	2.6781737	2.4466535	-4.1315902

H 1.9984125 1.8457114 -2.6146158
C 4.1727604 0.1478372 -4.7394810
H 3.3222611 -0.2367811 -2.8350299
H 5.0713090 -0.4582234 -4.6130635
H 4.4107632 0.9734155 -5.4130152
H 3.4146140 -0.4632045 -5.2331333
O 4.9962830 -1.5453370 -0.5925261
C 0.6445510 -0.5065409 1.0363464
C 2.4018766 -2.7179996 0.8276478
C 1.1377496 -2.8574537 1.4116130
C 0.2751398 -1.7698112 1.5135642
H 3.0806778 -3.5573571 0.7432342
H 0.8258619 -3.8227901 1.7891009
H -0.0228907 0.3423279 1.1133771
H -0.6970186 -1.9063612 1.9700736

Compound **CArAmC**(Triplet)

43
Free Energy = -904.206843 Hrt, B3LYP+D3(BJ)/def2-TZVP
N 3.327287 0.533054 -0.619469
C 3.738541 -0.827990 -0.365461
C 2.566229 -1.446568 0.245089
C 1.518858 -0.470865 0.336861
C 2.057277 0.703739 -0.208082
C 4.172033 1.521216 -1.221830
C 4.967531 2.319229 -0.390874
C 5.800500 3.254134 -1.001769
C 5.837540 3.381225 -2.382176
C 5.042485 2.573761 -3.180776
C 4.192099 1.624384 -2.617421
C 4.977639 2.138574 1.114843
H 6.432192 3.885383 -0.391386

H 6.493423 4.112193 -2.838548
H 5.087143 2.678073 -4.256663
C 3.367548 0.706520 -3.498963
C 4.915450 3.468035 1.872874
H 5.815631 4.065993 1.718111
H 4.828442 3.282065 2.945207
H 4.056589 4.063867 1.558870
C 6.200668 1.310976 1.538462
H 4.087410 1.571573 1.389263
H 6.223733 0.352602 1.019288
H 6.180809 1.124284 2.614633
H 7.124707 1.845009 1.304103
C 2.602335 1.466487 -4.587038
H 1.956302 0.780275 -5.138302
H 3.277984 1.929848 -5.308580
H 1.978392 2.252059 -4.157121
C 4.258178 -0.389672 -4.102544
H 2.625532 0.215163 -2.868869
H 4.773911 -0.947313 -3.320494
H 5.012186 0.046777 -4.761995
H 3.657878 -1.087758 -4.690489
O 4.840377 -1.260684 -0.638665
C 0.281651 -0.823348 0.891954
C 2.367336 -2.742002 0.701898
C 1.136373 -3.073799 1.248545
C 0.107642 -2.119029 1.339781
H 3.165520 -3.469519 0.627233
H 0.961906 -4.078506 1.610194
H -0.515394 -0.095648 0.966225
H -0.842207 -2.407852 1.771308

Compound **Dipp-6-Cl$_2$**(Singlet)

36

Free Energy = -1631.691653 Hrt, B3LYP+D3(BJ)/def2-TZVP

C	-4.8442147	-2.7763937	0.6592089
C	-3.4702580	-2.6733870	0.8638163
C	-5.7165127	-1.8456636	1.2041444
C	-0.8451590	-1.9722594	2.8663192
C	-3.0233904	-1.6037532	1.6469276
C	-5.2366532	-0.7845140	1.9576739
C	0.3925243	-1.2911191	2.2002760
C	-3.8719427	-0.6347552	2.1928966
C	-0.6016540	-0.7821554	1.1253857
H	-5.2367558	-3.5887173	0.0641065
H	-6.7811024	-1.9434679	1.0324134
H	-5.9314446	-0.0625399	2.3627873
Cl	1.1034310	-0.0129275	3.1909504
Cl	1.5830294	-2.4471111	1.5942081
N	-1.6149205	-1.4797336	1.8826287
O	-0.6295127	-0.1387017	0.1378914
C	-3.3270788	0.4980528	3.0431758
C	-4.1331291	1.7925206	2.9166173
H	-2.3169038	0.7224870	2.6925686
C	-3.2189413	0.0568592	4.5108466
H	-5.1263418	1.6971103	3.3594581
H	-4.2512969	2.0899729	1.8734797
H	-3.6200403	2.5979179	3.4446337
H	-2.5876612	-0.8263174	4.6159696
H	-4.2078555	-0.1848804	4.9072889
H	-2.7933637	0.8581846	5.1184280
C	-2.5001750	-3.6987348	0.3063862
C	-2.2590687	-4.8160349	1.3330018
H	-1.5336683	-5.5358826	0.9482752
H	-3.1910688	-5.3469377	1.5404928
H	-1.8806653	-4.4168174	2.2747907

C	-2.9372937	-4.2762281	-1.0415319
H	-1.5446457	-3.1966196	0.1371294
H	-3.1524504	-3.4873735	-1.7640195
H	-3.8256739	-4.9033314	-0.9455016
H	-2.1418732	-4.9029616	-1.4480284

Compound **Dipp-6-Cl$_2$**(Triplet)

36

Free Energy = -1631.625485 Hrt, B3LYP+D3(BJ)/def2-TZVP

C	-4.8638477	-2.8151214	0.6823151
C	-3.4895484	-2.7057184	0.8831456
C	-5.7325488	-1.8501110	1.1668664
C	-0.7065890	-2.2341893	2.6167081
C	-3.0298851	-1.5982562	1.6091662
C	-5.2474948	-0.7536183	1.8659743
C	0.4561660	-1.3988575	2.2406068
C	-3.8852785	-0.6040759	2.1101147
C	-0.6606249	-0.6086650	1.4047679
H	-5.2575374	-3.6563942	0.1289481
H	-6.7968568	-1.9482673	0.9934794
H	-5.9405893	-0.0085213	2.2288841
Cl	1.2021353	-0.4105495	3.5163478
Cl	1.6979665	-2.1415162	1.2050572
N	-1.6342576	-1.4791917	1.8531799
O	-0.6500756	0.3455060	0.6922579
C	-3.3422612	0.5490793	2.9358647
C	-4.2464876	1.7824323	2.9374701
H	-2.3956470	0.8595291	2.4919709
C	-3.0611556	0.0848740	4.3744062
H	-5.1816739	1.6020545	3.4719805
H	-4.4873738	2.1041002	1.9230716
H	-3.7392678	2.6063814	3.4417009

H	-2.3704495	-0.7584489	4.4007372
H	-3.9889366	-0.2226743	4.8624571
H	-2.6214522	0.8968597	4.9569350
C	-2.5319119	-3.7603331	0.3616101
C	-2.3931608	-4.9054828	1.3759672
H	-1.6658361	-5.6412573	1.0270485
H	-3.3516340	-5.4101776	1.5178514
H	-2.0603805	-4.5386036	2.3490456
C	-2.9219151	-4.2980357	-1.0178061
H	-1.5510061	-3.2936556	0.2559156
H	-3.0618010	-3.4878740	-1.7349931
H	-3.8426695	-4.8833305	-0.9819568
H	-2.1350793	-4.9542244	-1.3939724

Compound **Dipp-6-Ph$_2$(**Singlet)

56

Free Energy = -1174.420011 Hrt, B3LYP+D3(BJ)/def2-TZVP

C	2.8271939	-3.0467307	-0.3020068
C	2.8084992	-3.6168440	0.9665966
C	2.0947306	-1.8957709	-0.5612686
C	-4.7125259	1.5481511	-1.0848599
C	-3.3537980	1.4095412	-0.8156311
C	2.0506631	-3.0310929	1.9734167
C	-5.5707092	0.4634609	-0.9798245
C	-0.3787859	-0.1215069	-1.0681567
C	1.3343433	-1.3048024	0.4470633
C	-2.8937593	0.1417603	-0.4302069
C	1.3139334	-1.8807169	1.7158433
C	-5.0822232	-0.7787071	-0.6080722
C	0.5447294	-0.0476983	0.1686537
C	-3.7297030	-0.9753616	-0.3266328

C	-0.7965657	0.0782309	0.9789529
C	1.3731229	1.2159394	0.2462069
C	1.9175819	1.7967713	-0.8977116
C	1.5992199	1.8091539	1.4880954
C	2.6832474	2.9532120	-0.7993158
C	2.3640292	2.9641490	1.5831812
C	2.9088341	3.5395446	0.4401167
H	3.4122910	-3.4997803	-1.0923817
H	3.3796353	-4.5143083	1.1675370
H	2.1062311	-1.4576425	-1.5509130
H	-5.1047649	2.5102252	-1.3823535
C	-2.4055439	2.5950239	-0.8776583
H	-6.6246859	0.5863657	-1.1955981
H	2.0281782	-3.4717202	2.9622352
H	-5.7568866	-1.6229645	-0.5360745
H	0.7132286	-1.4293295	2.4939582
H	1.7430807	1.3463611	-1.8659745
C	-3.2819594	-2.3654126	0.0980966
H	1.1643344	1.3673423	2.3743436
H	3.1024670	3.3955250	-1.6942828
H	2.5317717	3.4178562	2.5518757
H	3.5045242	4.4403490	0.5155427
N	-1.4926034	0.0149175	-0.1650220
O	-0.3264387	-0.2394808	-2.2473883
C	-2.8191467	3.6578212	-1.8970957
H	-1.4283102	2.2267937	-1.1956566
C	-2.2321491	3.2135821	0.5180421
C	-2.1010221	-2.9467072	-0.6932514
H	-4.1398706	-3.0083396	-0.1170362
C	-3.0341977	-2.4439388	1.6119769
H	-2.1805471	-2.7260747	-1.7584488
H	-2.0855111	-4.0315985	-0.5721120
H	-1.1394315	-2.5809853	-0.3379911

H	-3.1842339	3.6129814	0.8755992
H	-1.5067175	4.0290761	0.4848112
H	-1.8833338	2.4757982	1.2415486
H	-2.9807219	3.2235621	-2.8850571
H	-2.0331427	4.4102927	-1.9802541
H	-3.7322984	4.1759720	-1.5974229
H	-2.2084255	-1.7975405	1.9116216
H	-2.7919292	-3.4698172	1.8992797
H	-3.9199541	-2.1319120	2.1682317

Compound **Dipp-6-Ph$_2$**(Triplet)

56

Free Energy = -1174.354638 Hrt, B3LYP+D3(BJ)/def2-TZVP

C	2.4470042	-3.2088028	-0.8693233
C	2.9368752	-3.7131492	0.3272868
C	1.6971394	-2.0356908	-0.8835342
C	-4.6948865	1.6461641	-0.7713350
C	-3.3339478	1.4712851	-0.5237205
C	2.6759983	-3.0384240	1.5171536
C	-5.5711790	0.5757786	-0.7216834
C	-0.4049217	0.0793108	-0.8668960
C	1.4321604	-1.3600414	0.3047352
C	-2.8808966	0.1744167	-0.2386215
C	1.9303029	-1.8694356	1.5041853
C	-5.0984166	-0.6952170	-0.4264807
C	0.6288246	-0.0809791	0.3333316
C	-3.7469264	-0.9297896	-0.1847641
C	-0.6722725	-0.1512280	1.1241899
C	1.4639259	1.1673065	0.5039806
C	2.6256047	1.3302263	-0.2521564
C	1.0663780	2.1918851	1.3609610
C	3.3666109	2.4999871	-0.1584486

C	1.8115396	3.3606944	1.4588325
C	2.9627287	3.5197434	0.6971036
H	2.6445327	-3.7273341	-1.7990939
H	3.5179142	-4.6265576	0.3366112
H	1.3196613	-1.6452434	-1.8188713
H	-5.0697949	2.6344523	-1.0032404
C	-2.3977500	2.6638641	-0.5776330
H	-6.6255328	0.7285992	-0.9154996
H	3.0530937	-3.4259530	2.4552324
H	-5.7873484	-1.5303316	-0.3921039
H	1.7261041	-1.3445553	2.4295423
H	2.9460722	0.5395810	-0.9170855
C	-3.3097710	-2.3515592	0.1292007
H	0.1707209	2.0633281	1.9557679
H	4.2628866	2.6154040	-0.7547672
H	1.4935988	4.1447402	2.1346207
H	3.5452779	4.4290484	0.7733055
N	-1.4887802	-0.0107271	-0.0200141
O	-0.3011142	0.2441429	-2.0526748
C	-2.2369539	3.1716444	-2.0164533
H	-1.4150378	2.3436747	-0.2368007
C	-2.8471433	3.7845697	0.3672741
C	-2.2194927	-2.9042154	-0.7994849
H	-4.2012711	-2.9612589	-0.0429295
C	-2.9338313	-2.5305873	1.6065189
H	-2.4277223	-2.6647441	-1.8432248
H	-2.1746400	-3.9909948	-0.7021599
H	-1.2326439	-2.5192989	-0.5525075
H	-3.8040415	4.2124317	0.0617366
H	-2.1093465	4.5894853	0.3681159
H	-2.9544694	3.4182957	1.3899872
H	-1.8672462	2.3785758	-2.6662586
H	-1.5255683	3.9996146	-2.0476205

H	-3.1897377	3.5294185	-2.4138847
H	-2.0287391	-1.9717685	1.8565993
H	-2.7399677	-3.5840769	1.8208455
H	-3.7378953	-2.1892891	2.2608397

Compound **Dipp-6-Spiro-Cy**(Singlet)

49

Free Energy = -907.756179 Hrt, B3LYP+D3(BJ)/def2-TZVP

C	-4.4358914	-5.0705527	1.7206764
C	-3.1782722	-4.5309433	1.9804892
C	-5.2444985	-4.5479279	0.7220299
C	-1.0098473	-2.0901922	2.4037840
C	-2.7722739	-3.4429153	1.1965292
C	-4.8153969	-3.4703132	-0.0379448
C	0.4006016	-2.0859875	1.7458057
C	-3.5671889	-2.8931444	0.1838253
C	-0.2397294	-2.9933162	0.6781893
H	-4.7894262	-5.9086027	2.3043663
H	-6.2182846	-4.9835516	0.5359015
H	-5.4599204	-3.0744627	-0.8098789
N	-1.4770835	-2.8783162	1.4199811
O	0.0294736	-3.5792965	-0.3200458
C	-3.1040997	-1.6706174	-0.5880202
C	-3.6917189	-1.5739405	-1.9967100
H	-2.0215899	-1.7452246	-0.7076889
C	-3.3997283	-0.3959842	0.2178965
H	-4.7658932	-1.3792055	-1.9766527
H	-3.5203654	-2.4897343	-2.5645860
H	-3.2238736	-0.7477474	-2.5349700
H	-2.9422553	-0.4326975	1.2074476
H	-4.4771024	-0.2744545	0.3518431
H	-3.0207060	0.4846906	-0.3059274

C	-2.2591698	-5.1204818	3.0356111
C	-3.0024115	-5.8052336	4.1834065
H	-2.2926179	-6.0914265	4.9615993
H	-3.5089581	-6.7160796	3.8571070
H	-3.7452891	-5.1422251	4.6296140
C	-1.2562353	-6.0870893	2.3866187
H	-1.6971730	-4.2933769	3.4727431
H	-0.6880602	-5.6103468	1.5872768
H	-1.7795539	-6.9412885	1.9505520
H	-0.5507849	-6.4641205	3.1305519
C	1.4872720	-2.7597107	2.5845245
C	0.8461257	-0.7146272	1.2365974
C	2.1793875	-0.7937741	0.4889706
C	2.8188025	-2.8327213	1.8328933
C	3.2612840	-1.4516714	1.3468829
H	2.7107525	-3.5015790	0.9735794
H	3.5812915	-3.2709642	2.4814484
H	4.1926454	-1.5324058	0.7813350
H	3.4730338	-0.8141009	2.2131199
H	2.4897894	0.2095725	0.1872512
H	2.0435776	-1.3734061	-0.4292980
H	1.6038012	-2.1740986	3.5021906
H	1.1624001	-3.7583096	2.8877921
H	0.0715545	-0.2810488	0.5986859
H	0.9398881	-0.0565333	2.1065227

Compound **Dipp-6-Spiro-Cy**(Triplet)

49

Free Energy = -907.690734 Hrt, B3LYP+D3(BJ)/def2-TZVP

C	-4.2231546	-5.2663268	1.4223534
C	-2.9975265	-4.6613233	1.6974082
C	-5.1533869	-4.6560112	0.5976654

C	-1.0772215	-1.6125654	1.9963518
C	-2.7314725	-3.4248350	1.0921323
C	-4.8714914	-3.4267641	0.0178395
C	0.3985322	-1.8310954	1.6928735
C	-3.6538893	-2.7918302	0.2416368
C	-0.1800927	-3.1139508	0.9888596
H	-4.4529854	-6.2257958	1.8668621
H	-6.1021540	-5.1398477	0.4018118
H	-5.6045743	-2.9642747	-0.6287983
N	-1.4837803	-2.7947286	1.3351154
O	0.2921062	-4.0377428	0.3748721
C	-3.3444072	-1.4479744	-0.3900931
C	-3.8593227	-1.3238459	-1.8265914
H	-2.2585591	-1.3475986	-0.4273760
C	-3.8827908	-0.3068013	0.4854753
H	-4.9500259	-1.3023201	-1.8688790
H	-3.5124933	-2.1528090	-2.4454977
H	-3.5001118	-0.3930028	-2.2698487
H	-3.4691915	-0.3537859	1.4942974
H	-4.9711803	-0.3641356	0.5630780
H	-3.6208507	0.6633700	0.0573473
C	-2.0059564	-5.3438454	2.6198309
C	-2.6343554	-5.6933502	3.9743707
H	-1.8765213	-6.1055760	4.6439456
H	-3.4238957	-6.4403772	3.8718228
H	-3.0665889	-4.8106319	4.4489447
C	-1.3950593	-6.5818031	1.9499228
H	-1.1943129	-4.6437224	2.8159835
H	-0.9027278	-6.3110260	1.0164402
H	-2.1650431	-7.3265927	1.7346743
H	-0.6550360	-7.0434233	2.6076043
C	1.3025266	-2.1446902	2.8853742
C	1.0540089	-0.8472951	0.7233872

C	2.4395544	-1.3291958	0.2820857
C	2.6860179	-2.6217566	2.4329817
C	3.3395492	-1.6158781	1.4846198
H	2.5837816	-3.5842812	1.9243536
H	3.3175373	-2.7877213	3.3092571
H	4.3085533	-1.9917499	1.1475360
H	3.5345911	-0.6812979	2.0241567
H	2.8958568	-0.5775178	-0.3667491
H	2.3284986	-2.2405259	-0.3117555
H	1.4026600	-1.2311404	3.4810682
H	0.8243450	-2.8893345	3.5255988
H	0.4043060	-0.6913482	-0.1407122
H	1.1427915	0.1179507	1.2330876

Compound **Ph-6-Ph$_2$**(Singlet)

38

Free Energy = -938.751428 Hrt, B3LYP+D3(BJ)/def2-TZVP

C	2.5327799	-2.0733726	-2.5281639
C	2.8884616	-3.2021792	-1.8017524
C	1.8121512	-1.0487508	-1.9219996
C	-4.6786257	1.1921766	-1.3841536
C	-3.2997676	1.1168266	-1.2258352
C	2.5208076	-3.3047106	-0.4632992
C	-5.5196456	0.3846122	-0.6291040
C	-0.2609512	0.7647301	-0.7961624
C	1.4457253	-1.1463875	-0.5817684
C	-2.7746442	0.2200308	-0.3001546
C	1.8037592	-2.2837497	0.1437449
C	-4.9790045	-0.5080424	0.2928493
C	0.6826577	-0.0479290	0.1195846
C	-3.6060697	-0.5962516	0.4619744
C	-0.6456566	-0.5915438	0.7535505

C	1.5248410	0.8081603	1.0411025
C	2.8738382	1.0383177	0.7750481
C	0.9433395	1.4117369	2.1568137
C	3.6250219	1.8593624	1.6058910
C	1.6970586	2.2257812	2.9928397
C	3.0399122	2.4548242	2.7177817
H	2.8159353	-1.9852058	-3.5694512
H	3.4480695	-3.9993160	-2.2744427
H	1.5467715	-0.1673971	-2.4909070
H	-5.0933557	1.8875639	-2.1023237
H	-2.6384415	1.7414012	-1.8080316
H	-6.5924462	0.4481333	-0.7574364
H	2.7918012	-4.1836373	0.1079706
H	-5.6305467	-1.1404287	0.8820609
H	1.5113239	-2.3616966	1.1824699
H	3.3383415	0.5672161	-0.0806238
H	-3.1631990	-1.2821284	1.1706120
H	-0.1006652	1.2329392	2.3822009
H	4.6712641	2.0307779	1.3860957
H	1.2342045	2.6800130	3.8598053
H	3.6277064	3.0910162	3.3672308
N	-1.3682445	0.1308750	-0.1236834
O	-0.2120693	1.5916263	-1.6472882

Compound **Ph-6-Ph$_2$**(Triplet)

38

Free Energy = -938.689112 Hrt, B3LYP+D3(BJ)/def2-TZVP

C	1.8687362	-2.7127814	-2.3688780
C	3.0194342	-3.2156367	-1.7706514
C	1.1213626	-1.7338897	-1.7301835
C	-4.5703952	1.4307784	-1.5682917
C	-3.2087931	1.2560837	-1.3708251

C	3.4164881	-2.7287120	-0.5316105
C	-5.4979377	0.7069514	-0.8243482
C	-0.2531279	0.6605682	-0.7296076
C	1.5144775	-1.2361067	-0.4861023
C	-2.7750778	0.3390549	-0.4098497
C	2.6716982	-1.7428007	0.1041385
C	-5.0544369	-0.2031886	0.1291866
C	0.6729134	-0.2053334	0.2330238
C	-3.6967366	-0.3921114	0.3410670
C	-0.6971519	-0.6838147	0.7093708
C	1.4404679	0.6760834	1.1871698
C	2.4251823	1.5282475	0.6835446
C	1.1858859	0.6634938	2.5548940
C	3.1376103	2.3562344	1.5387754
C	1.9069808	1.4870261	3.4130320
C	2.8830528	2.3357415	2.9071180
H	1.5540425	-3.0809626	-3.3370893
H	3.6032874	-3.9786921	-2.2694311
H	0.2296012	-1.3516670	-2.2103721
H	-4.9091903	2.1411748	-2.3115173
H	-2.4828807	1.8149905	-1.9431119
H	-6.5578936	0.8514369	-0.9867706
H	4.3108270	-3.1131904	-0.0576815
H	-5.7684396	-0.7706968	0.7122439
H	2.9927513	-1.3623297	1.0641670
H	2.6213345	1.5451671	-0.3805470
H	-3.3419804	-1.0995161	1.0800135
H	0.4206012	0.0035680	2.9435967
H	3.8926324	3.0201789	1.1372151
H	1.7041794	1.4639121	4.4763180
H	3.4433151	2.9790166	3.5737066
N	-1.4143112	0.1373129	-0.1859096
O	-0.0411010	1.4636096	-1.5957567

Compound **Ph-6-Me₂**(Singlet)

24

Free Energy = -555.440582 Hrt, B3LYP+D3(BJ)/def2-TZVP

H -2.014909 -0.037235 7.503421

H -2.536307 1.650636 9.240236

C -1.360265 0.812663 7.647740

C -1.653421 1.761365 8.624053

C -0.231389 0.947316 6.854635

H 0.016818 0.223927 6.090523

C -0.809449 2.849776 8.804642

H -1.032079 3.590387 9.561865

C 0.606371 2.042940 7.045721

C 0.326473 2.999299 8.017544

N 1.763119 2.181298 6.233482

H 0.988149 3.842442 8.151375

C 2.233404 1.402559 5.240108

C 2.815783 3.175952 6.184842

C 3.442043 2.361869 5.040827

O 3.008059 4.164936 6.815383

H 4.764620 1.222748 6.342995

C 4.786763 1.727955 5.376017

H 2.476449 3.509052 3.461620

C 3.447610 3.066852 3.689803

H 5.042724 0.989978 4.613877

H 3.685963 2.348310 2.903756

H 5.570828 2.487035 5.403057

H 4.197756 3.859818 3.675879

Compound **Ph-6-Me₂**(Triplet)

24

Free Energy = -555.378191 Hrt, B3LYP+D3(BJ)/def2-TZVP
H -2.046923 -0.021186 7.528122
H -2.554014 1.669600 9.267604
C -1.387674 0.825314 7.672172
C -1.672775 1.773861 8.648470
C -0.259156 0.954413 6.875959
H -0.029646 0.220582 6.113573
C -0.817338 2.858002 8.824034
H -1.033360 3.600010 9.582116
C 0.592503 2.045127 7.059837
C 0.315736 3.004443 8.037907
N 1.722444 2.162540 6.253475
H 0.984282 3.842892 8.168210
C 2.254000 1.376757 5.209991
C 2.762585 3.081393 6.152130
C 3.450232 2.290694 4.980357
O 3.019879 4.093786 6.747649
H 4.776277 1.191871 6.304273
C 4.815847 1.734971 5.359860
H 2.487042 3.464637 3.427102
C 3.472722 3.068344 3.671707
H 5.174019 1.054316 4.585251
H 3.796788 2.421124 2.854578
H 5.535001 2.550886 5.457245
H 4.174337 3.901694 3.746642

Compound **Dipp-6-Me$_2$**(Singlet)

42
Free Energy = -791.106103 Hrt, B3LYP+D3(BJ)/def2-TZVP
C -4.5443825 -2.1973240 -0.3445365
C -3.1764641 -2.2642311 -0.0938092
C -5.3132742 -1.1702449 0.1798893

C	-0.1478972	-0.7907870	0.3024185
C	-2.6044727	-1.2566188	0.6980414
C	-4.7242810	-0.1979463	0.9715119
C	0.8413577	-1.5745053	1.2198744
C	-3.3586284	-0.2163970	1.2564909
C	-0.4327566	-2.1552935	1.8667384
H	-5.0146016	-2.9557984	-0.9540660
H	-6.3761363	-1.1317390	-0.0232382
H	-5.3312659	0.5968769	1.3872566
C	1.6695923	-2.6028886	0.4583989
C	1.6944960	-0.6942891	2.1220298
N	-1.1941815	-1.3405468	0.9420194
O	-0.7699607	-2.9293440	2.7002707
H	1.0489202	-3.2209381	-0.1922706
H	2.2176937	-1.2997040	2.8646914
H	1.0971816	0.0511740	2.6481828
H	2.4365834	-0.1667119	1.5201978
H	2.1974496	-3.2589665	1.1531454
H	2.4051119	-2.0911842	-0.1645887
C	-2.8040735	0.8943271	2.1347368
C	-2.0924703	0.4018323	3.4013801
H	-3.6864741	1.4408282	2.4789474
C	-1.9478067	1.8966592	1.3480630
H	-2.6720999	-0.3696604	3.9097531
H	-1.9556885	1.2370072	4.0915181
H	-1.1058566	-0.0056794	3.1930082
H	-1.0501385	1.4323990	0.9392898
H	-1.6469705	2.7215009	1.9985842
H	-2.5106227	2.3115109	0.5100573
C	-2.3211101	-3.3600642	-0.7056811
C	-3.0651109	-4.6836209	-0.8957551
H	-1.5008579	-3.5654819	-0.0157651
C	-1.7101487	-2.8787395	-2.0304743

H	-2.4995127	-2.6650930	-2.7549296
H	-1.0596011	-3.6478048	-2.4537317
H	-1.1259044	-1.9677699	-1.8924422
H	-3.5392248	-5.0117990	0.0305148
H	-2.3629886	-5.4574112	-1.2112856
H	-3.8340601	-4.6122132	-1.6673930

Compound **Dipp-6-Me$_2$**(Triplet)

42

Free Energy = -791.040546 Hrt, B3LYP+D3(BJ)/def2-TZVP

C	-4.5618353	-2.2253828	-0.3015090
C	-3.1908065	-2.2754316	-0.0630738
C	-5.3353346	-1.1913057	0.2022475
C	-0.0820030	-0.6819452	0.3485542
C	-2.6166390	-1.2472199	0.7048620
C	-4.7482456	-0.1949000	0.9649247
C	0.9137872	-1.5151016	1.1462631
C	-3.3813359	-0.2002403	1.2414345
C	-0.4210111	-2.1240262	1.7185172
H	-5.0318490	-3.0028367	-0.8867362
H	-6.4001049	-1.1669681	0.0065402
H	-5.3584589	0.6040890	1.3679443
C	1.7216487	-2.5352567	0.3557197
C	1.7466624	-0.7837743	2.1877630
N	-1.2133428	-1.2982658	0.9397713
O	-0.6761183	-2.9896916	2.5145374
H	1.1027518	-3.0511446	-0.3784479
H	2.1683061	-1.4998420	2.8964003
H	1.1492950	-0.0597354	2.7416048
H	2.5710363	-0.2526997	1.7078319
H	2.1480866	-3.2774985	1.0339396
H	2.5405638	-2.0423003	-0.1716574

C	-2.8264913	0.9206477	2.1057738
C	-2.1667454	0.4293727	3.4004704
H	-3.7029331	1.5001368	2.4098232
C	-1.9197417	1.8830277	1.3277507
H	-2.8173402	-0.2650824	3.9336180
H	-1.9634037	1.2788489	4.0562811
H	-1.2229494	-0.0784006	3.2145405
H	-0.9932528	1.3986999	1.0120509
H	-1.6529730	2.7365438	1.9554566
H	-2.4243621	2.2601861	0.4364252
C	-2.3331959	-3.3786365	-0.6583211
C	-3.0841245	-4.6951937	-0.8671635
H	-1.5304397	-3.5903653	0.0479569
C	-1.6951403	-2.9040973	-1.9733640
H	-2.4677083	-2.6865504	-2.7147321
H	-1.0405549	-3.6773708	-2.3823975
H	-1.1031051	-1.9997783	-1.8265696
H	-3.5799021	-5.0207298	0.0485356
H	-2.3809836	-5.4742708	-1.1669716
H	-3.8356741	-4.6176745	-1.6556621

V. References

[1] A. R. Chianese, X. Li, M. C. Janzen, J. W. Faller, R. H. Crabtree, Rhodium and Iridium Complexes of *N*-Heterocyclic Carbenes via Transmetalation: Structure and Dynamics *Organometallics* **2003**, *22*, 1663.

[2] R. A. Kelly Iii, H. Clavier, S. Giudice, N. M. Scott, E. D. Stevens, J. Bordner, I. Samardjiev, C. D. Hoff, L. Cavallo, S. P. Nolan, Determination of *N*-Heterocyclic Carbene (NHC) Steric and Electronic Parameters using the [(NHC)Ir$(CO)_2$Cl] System *Organometallics* **2008**, *27*, 202.

[3] S. Wolf, H. Plenio, Synthesis of (NHC)Rh(cod)Cl and (NHC)RhCl$(CO)_2$ complexes – Translation of the Rh- into the Ir-scale for the electronic properties of NHC ligands *J. Organomet. Chem.* **2009**, *694*, 1487.

[4] G. Sheldrick, SHELXT - Integrated space-group and crystal-structure determination *Acta Crystallogr. A* **2015**, *71*, 3.

[5] O. V. Dolomanov, L. J. Bourhis, R. J. Gildea, J. A. K. Howard, H. Puschmann, OLEX2: a complete structure solution, refinement and analysis program *J. Appl. Crystallogr.* **2009**, *42*, 339.

[6] G. Sheldrick, Crystal structure refinement with SHELXL *Acta Crystallogr. C* **2015**, *71*, 3.

[7] M. Shimizu, Y. Gama, T. Takagi, M. Shibakami, I. Shibuya, A Convenient Synthesis of Ketenimines from Thioamides with Haloiminium Salts *Synthesis* **2000**, *2000*, 517.

[8] J. Kästner, J. M. Carr, T. W. Keal, W. Thiel, A. Wander, P. Sherwood, DL-FIND: An Open-Source Geometry Optimizer for Atomistic Simulations *J. Phys. Chem. A* **2009**, *113*, 11856.

[9] S. Metz, J. Kästner, A. A. Sokol, T. W. Keal, P. Sherwood, ChemShell—a modular software package for QM/MM simulations *WIREs Comput. Mol. Sci.* **2014**, *4*, 101.

[10] A. D. Becke, Density-functional thermochemistry. III. The role of exact exchange *J. Chem. Phys.* **1993**, *98*, 5648.

[11] C. Lee, W. Yang, R. G. Parr, Development of the Colle-Salvetti correlation-energy formula into a functional of the electron density *Phys. Rev. B* **1988**, *37*, 785.

[12] S. Grimme, S. Ehrlich, L. Goerigk, Effect of the damping function in dispersion corrected density functional theory *J. Comput. Chem.* **2011**, *32*, 1456.

[13] S. Grimme, J. Antony, S. Ehrlich, H. Krieg, A consistent and accurate ab initio parametrization of density functional dispersion correction (DFT-D) for the 94 elements H-Pu *J. Chem. Phys.* **2010**, *132*.

[14] A. Klamt, G. Schüürmann, COSMO: a new approach to dielectric screening in solvents with explicit expressions for the screening energy and its gradient *Perkin Trans. 2* **1993**, 799.

[15] P. G. Smith, M. N. Patel, J. Kim, T. E. Milner, K. P. Johnston, Effect of Surface Hydrophilicity on Charging Mechanism of Colloids in Low-Permittivity Solvents *J. Phys. Chem. C* **2007**, *111*, 840.

[16] S. Seritan, C. Bannwarth, B. S. Fales, E. G. Hohenstein, C. M. Isborn, S. I. L. Kokkila-Schumacher, X. Li, F. Liu, N. Luehr, J. W. Snyder Jr., C. Song, A. V. Titov, I. S. Ufimtsev, L.-P. Wang, T. J. Martínez, TeraChem: A graphical processing unit-accelerated electronic structure package for large-scale ab initio molecular dynamics *WIREs Comput. Mol. Sci.* **2021**, *11*, e1494.

[17] A. Schäfer, C. Huber, R. Ahlrichs, Fully optimized contracted Gaussian basis sets of triple zeta valence quality for atoms Li to Kr *J. Chem. Phys.* **1994**, *100*, 5829.

[18] G. Bussi, M. Parrinello, Accurate sampling using Langevin dynamics *Phys. Rev.* **2007**, *75*, 056707.

[19] J. S. Binkley, J. A. Pople, W. J. Hehre, Self-consistent molecular orbital methods. 21. Small split-valence basis sets for first-row elements *J. Am. Chem. Soc.* **1980**, *102*, 939.

[20] C. Bannwarth, S. Ehlert, S. Grimme, GFN2-xTB—An Accurate and Broadly Parametrized Self-Consistent Tight-Binding Quantum Chemical Method with Multipole Electrostatics and Density-Dependent Dispersion Contributions *J. Chem. Theory Comput.* **2019**, *15*, 1652.

[21] X. Hu, W. Yang, Accelerating self-consistent field convergence with the augmented Roothaan–Hall energy function *J. Chem. Phys.* **2010**, *132*.
